# Neutrophil-enriched gene signature correlates with teplizumab therapy resistance in different stages of type 1 diabetes

**DOI:** 10.1172/JCI176403

**Published:** 2025-09-30

**Authors:** Gabriele Sassi, Pierre Lemaitre, Laia Fernández Calvo, Francesca Lodi, Álvaro Cortés Calabuig, Samal Bissenova, Amber Wouters, Laure Degroote, Marijke Viaene, Niels van Damme, Lauren Higdon, Peter S. Linsley, S. Alice Long, Chantal Mathieu, Conny Gysemans

**Affiliations:** 1 Leuven Diabetes Lab, Clinical and Experimental Endocrinology (CEE), Campus Gasthuisberg O&N1 and; 2 VIB Laboratory of Translational Genetics, Leuven, Belgium;; 3 Genomics Core Leuven, Centre for Human Genetics, KU Leuven, Leuven, Belgium.; 4 VIB Single Cell Core, Leuven, Ghent, Belgium.; 5 UCSF Diabetes Center, Immune Tolerance Network (ITN), San Francisco, California, USA.; 6 Center for Systems Immunology, Benaroya Research Institute, Seattle, Washington, USA.

**Keywords:** Autoimmunity, Endocrinology, Diabetes, Immunotherapy, Neutrophils

## Abstract

Teplizumab, a humanized anti-CD3 mAb, represents a breakthrough in autoimmune type 1 diabetes (T1D) treatment, by delaying clinical onset in stage 2 and slowing progression in early stage 3 of the disease. However, therapeutic responses are heterogeneous. To better understand this variability, we applied single-cell transcriptomics to paired peripheral blood and pancreas samples from anti–mouse CD3–treated nonobese diabetic (NOD) mice and identified distinct gene signatures associated with the therapy outcome, with consistent patterns across compartments. Success-associated signatures were enriched in NK or CD8^+^ T cells and other immune cell types, whereas resistance signatures were predominantly expressed by neutrophils. The immune cell communities underlying these response signatures were confirmed in human whole blood sequencing data from the Autoimmunity-blocking Antibody for Tolerance (AbATE) study at 6 months, which assessed teplizumab therapy in individuals with stage 3 T1D. Furthermore, baseline expression profiling in the human TrialNet Anti-CD3 Prevention (TN10) (stage 2) and AbATE (stage 3) cohorts identified immune signatures predictive of therapy response, T cell–enriched signatures in responders, and neutrophil-enriched signatures in nonresponders, highlighting the roles of both adaptive and innate immunity in determining teplizumab treatment outcomes. Using an elastic net logistic regression model, we developed a 26-gene blood-based signature predicting the response to teplizumab (AUC = 0.97). These findings demonstrate the predictive potential of immune gene signatures and the value of transcriptomics profiling in guiding individualized treatment strategies with teplizumab in individuals with T1D.

## Abstract 2

Teplizumab therapy in type 1 diabetes shows variable patient responses, with specific immune gene signatures offering a powerful tool to predict treatment success or resistance.

## Introduction

The approval of teplizumab, a humanized anti-CD3 mAb, as the first therapy to delay the onset of symptomatic stage 3 type 1 diabetes (T1D) in high-risk, autoantibody-positive individuals with stage 2 disease represents a significant milestone in the prevention and early intervention for T1D (1, 2). An integrated analysis of 5 teplizumab trials in individuals with stage 3 T1D also confirmed the consistency in preserving endogenous β cell function for at least 2 years, as measured by C-peptide levels (3), which is now considered a valid surrogate endpoint for clinical benefit of disease-modifying therapies in individuals with T1D (4). A major challenge remains the variability in teplizumab outcomes and duration on an individual basis. Post hoc and meta-analyses of treated cohorts revealed that a requirement of younger age and lower hemoglobin A1c (HbA1c) and insulin levels at disease onset (5–7) were linked to therapeutic success, but reliable biomarkers for predicting, or at least understanding, therapy outcomes are needed. Yet, the heterogeneous population of individuals with stage 2 or 3 T1D, with differences in age, genetics, disease phenotype/endotype, and clinical course (8), will necessitate a personalized approach to treatment. Understanding the underlying mechanisms of action that may determine teplizumab treatment outcomes will be crucial for avoiding futile treatment burden.

Recent research has demonstrated that teplizumab treatment during stage 2 T1D induces dynamic transcriptional changes in CD4^+^ and CD8^+^ T cells, characterized by gene expression profiles associated with T cell activation (9). Notably, sustained expression of eomesodermin (*EOMES*) correlated with delayed progression to stage 3 disease, and trajectory analyses revealed differentiation toward immunoregulatory and exhausted T cell phenotypes. In stage 3 T1D, individuals who responded to teplizumab also showed increased frequencies of KLRG1^+^TIGIT^+^CD8^+^ T cells expressing an *EOMES*-associated transcriptional signature (10). Differential expression of *IL10* and *KLRC1* (encoding NKG2A) further implicated modulation of Treg-associated pathways (11, 12). Additionally, baseline data from the TrialNet Anti-CD3 Prevention (TN10) (stage 2) and AbATE (stage 3) trials revealed that individuals seropositive for EBV had higher frequencies of partially exhausted CD8^+^ T cells and Tregs, along with stronger clinical responses to teplizumab compared with EBV-seronegative participants (13). Despite growing insights into the mechanisms of teplizumab success, the basis of therapy resistance remains poorly understood. Currently, no validated biomarkers, apart from metabolic parameters (e.g., baseline C-peptide), exist to guide patient selection or enable personalized teplizumab therapy in stage 2 or 3 T1D, underscoring the urgent need to identify resistance mechanisms and predictive markers to improve clinical outcomes.

In this study, we used unbiased single-cell transcriptomics on paired peripheral blood and pancreatic samples from nonobese diabetic (NOD) mice treated with anti–mouse CD3, enabling the identification of gene expression signatures and immune cell networks associated with either therapeutic success or resistance. Building on these findings, we extended our analysis to human datasets, linking transcriptional signatures to metabolic outcomes by integrating whole blood single-cell RNA-Seq (sc-RNA-Seq) with bulk RNA-Seq data from the TN10 and AbATE randomized, controlled trials of teplizumab treatment of individuals with stage 2 or stage 3 T1D, respectively (1, 10). From these human trial datasets, we derived a predictive 26-gene blood-based signature that effectively stratified responders (Rs) and nonresponders (NRs) to teplizumab.

## Results

### Low-dose anti–mouse CD3 stably reverts recent-onset T1D in NOD mice.

**Figure 1:**
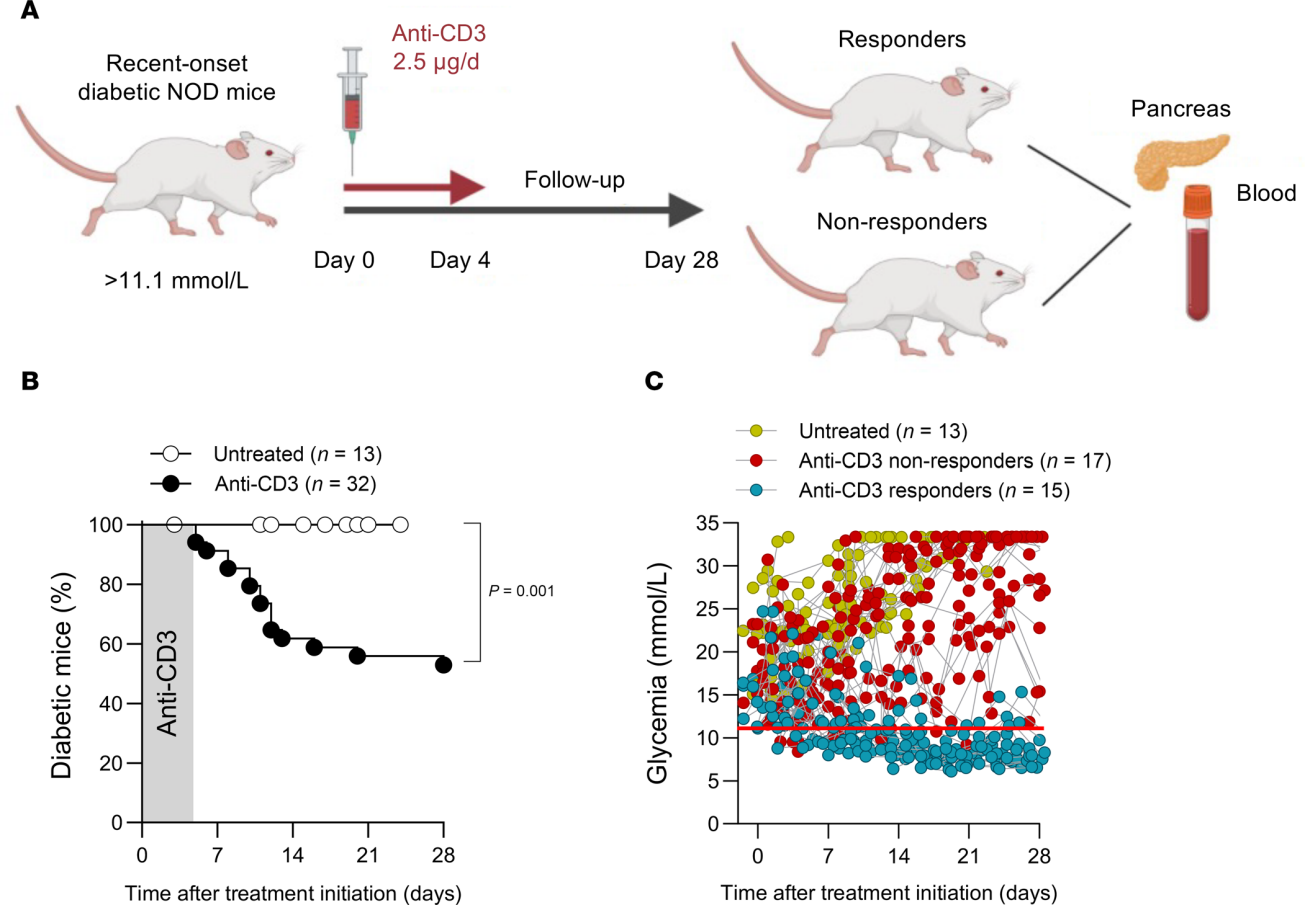
Anti–mouse CD3 study design. (**A**) RO NOD mice with blood glucose levels above 11.1 mmol/L were treated with anti–mouse CD3 antibody (2.5 μg/day) for 5 consecutive days. Blood glucose concentrations were monitored 3 times per week for 28 days following treatment initiation. At day 28, mice were classified as Rs or NRs on the basis of glycemic control. Pancreas and peripheral blood samples were collected at this time point for further analysis. (**B**) Kaplan-Meier survival curve shows the percentage of remission from disease of RO NOD mice after anti–mouse CD3 therapy. Black and white symbols indicate treated and untreated samples, respectively. (**C**) Individual glycemia levels of untreated mice, anti–mouse CD3 R mice, and anti–mouse CD3 NR mice during the study follow-up are shown in yellow, blue, and red, respectively. Normoglycemia threshold (11.1 mmol/L) is depicted by the red line. In **B, the** Mantel-Cox log-rank test was used for statistical comparison between groups.

Five consecutive injections of low-dose anti–mouse CD3 mAb (2.5 μg – clone 145-2C11) were given to a cohort of 32 NOD mice with recent-onset (RO) diabetes, resulting in an overall disease remission rate of 47% compared with untreated diabetic control mice (*n* = 13), which remained hyperglycemic and were euthanized at humane endpoints (*P* = 0.001) (Figure 1, A and B). In this partial response treatment model, the R mice were characterized by glycemic levels that returned to normal (11.1 mmol/L) within 2 weeks of therapy initiation, whereas NRs and untreated diabetic mice exhibited oscillating glycemic values that did not return to normoglycemic levels (Figure 1C).

### Low-dose anti–mouse CD3 modulates innate and adaptive immune subsets in peripheral blood and the pancreas.

To explore the mechanism of action of anti–mouse CD3 therapy and its effect on peripheral blood and pancreas-infiltrating immune cells, we exploited cellular indexing of transcriptomes and epitopes sequencing (CITE-Seq) technology. Using a large panel of 190 DNA-barcoded antibodies, we performed multimodal immune cell phenotyping by simultaneously examining the complete transcriptome and surface proteome at the single-cell level (Supplemental Table 1; supplemental material available online with this article; https://doi.org/10.1172/JCI176403DS1). We obtained high-quality data from FACS-sorted CD45^+^ leukocytes isolated from paired peripheral blood and pancreas samples obtained from RO NOD mice (*n* = 4) as well as from R (*n* = 4) and NR (*n* = 3) mice 28 days after anti–mouse CD3 therapy (Supplemental Figure 1A). The CITE-Seq dataset comprised 111,907 single cells, with a total of 24,795 genes and was balanced across the 3 biological conditions (Supplemental Figure 1B).

Applying unsupervised principal component analysis (PCA) based on RNA and protein expression, clusters in the resulting 2D uniform manifold approximation and projection (UMAP) representation consisted of different cell types, identified as the major immune cell populations present in peripheral blood and pancreas (Supplemental Figure 2A). Cell type identities were assigned based on the differential expression of signature genes and proteins (Supplemental Figure 3, A and B, and Supplemental Table 2). We observed a clear distinction in immune cell distribution between peripheral blood and pancreas, with myeloid lineage cells predominantly present in peripheral blood and lymphocytes primarily infiltrating the pancreas, underscoring the divergent compositions of immune cells in these 2 compartments (Supplemental Figure 2, B and C). We observed the most significant differences in immune cell proportions when comparing paired peripheral blood and pancreas specimens from individual mice, regardless of outcome (Supplemental Figure 2E). Next, we analyzed the proportions of immune cell types in peripheral blood and pancreas, taking into consideration the metabolic outcome. Notably, we observed similar proportions of all major cell types across the RO, R, and NR samples, indicating their presence in all conditions. However, minor variations were detected, specifically, in peripheral blood, where NK or CD8^+^ T cells (NK/CD8^+^ T cells) had a higher prevalence in anti–mouse CD3–treated R and NR samples compared with RO samples. In the pancreas, the frequency of DCs was lower in R compared with RO samples (Supplemental Figure 2D). These findings highlight the consistent presence of the major cell types across the analyzed biological conditions. However, large variations existed in their proportions in peripheral blood and pancreas, indicating the importance of comparing both tissues when studying the therapy outcome.

### A gene signature enriched in NK/CD8^+^ T and innate immune cells in both peripheral blood and pancreas correlates with anti–mouse CD3 therapy success in NOD mice.

**Figure 2:**
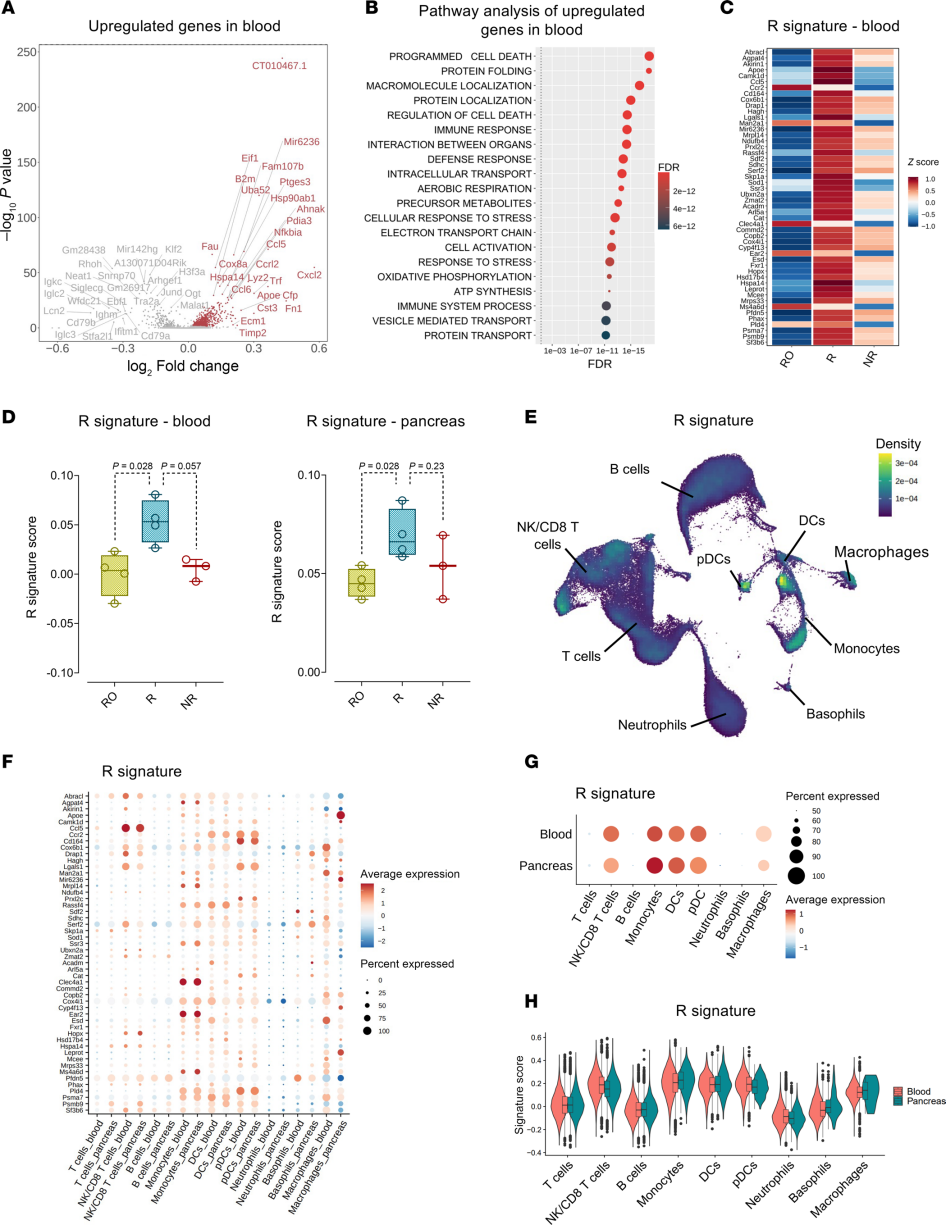
Blood-based R signature in anti–mouse CD3 R mice and its cellular origin. (**A**) Volcano plot illustrating DEGs comparing the peripheral blood leukocytes of R and NR samples. Red dots and labels indicate upregulated genes, while gray dots and labels indicate downregulated genes that were statistically significant (*P* < 0.05). The log-transformed FC values are reported on the *x* axis, and the –log_10_-transformed values of the adjusted *P* values are shown on the *y* axis. *P* values were obtained by Wilcoxon rank-sum test with Bonferroni correction (Seurat function FindMarkers). (**B**) hypeR pathway analysis performed on upregulated DEGs in circulating leukocytes of Rs. Assessment of relevant pathways was done utilizing the hallmark gene sets from the MSigDB as a reference. (**C**) Heatmap presenting the normalized expression levels of the top 50 genes comprising the R signature, represented using the *z* score. (**D**) Box plots representing the R signature score calculated in peripheral blood (left) and pancreas (right) RO, R, and NR samples. The mean expression of the genes comprising the R signature in each individual cell was calculated using the Seurat function AddModuleScore. Boxes represent the median (center line) and extend from the 25th to 75th percentiles (bottom and top lines, respectively); whiskers extend from the minimum to the maximum value. (**E**) UMAP plot displaying the expression of the R signature in the main immune cell types in peripheral blood and pancreas. (**F**) Dot plot showing the expression of individual genes comprising the R signature in the main immune cell types in peripheral blood and pancreas. (**G**) Dot plot showing the average expression of the R signature in the main immune cell types in peripheral blood and pancreas. (**H**) Violin plots comparing the expression levels of the R signature in the main immune cell types in peripheral blood and pancreas. In **D**, the Mann-Whitney *U* test was used for statistical comparison.

To identify a blood-based gene signature associated with an anti–mouse CD3 therapy response, we performed a differentially expressed gene (DEG) analysis comparing the gene profiles of peripheral blood leukocytes between R and NR samples. The volcano plot displayed the upregulated (633 genes) and downregulated (174 genes) genes in R compared with NR samples following anti–mouse CD3 therapy, as determined using the Wilcoxon rank-sum test with Bonferroni correction (*P* < 0.05) (Figure 2A and Supplemental Table 3). The upregulated and downregulated DEGs were then separately assessed to elucidate their unique biological processes. Gene Ontology (GO) pathway analysis of the upregulated genes showed their involvement in diverse regulatory networks, including immunological processes, apoptosis, cell death, cell activation, and intracellular protein and electron transport (Figure 2B and Supplemental Table 3).

To narrow down the selection from the initial pool of 633 upregulated genes, a ranking analysis, determined through the Wilcoxon rank-sum test with Bonferroni correction (*P* < 0.05), identified the top 50 upregulated genes with the highest discriminatory capacity in terms of the anti–mouse CD3 therapy response in R versus NR samples, constituting the “R signature” (Figure 2C). To evaluate the efficacy of the R signature in identifying the anti–mouse CD3 therapy response, we calculated signature scores by combining all single cells (pseudobulk) for each biological replicate in peripheral blood samples. As expected, the R samples exhibited significantly higher scores than did the NR samples, whereas the RO samples had scores resembling those of the NR samples. Interestingly, a similar trend was observed when scoring the peripheral blood gene signature to pseudobulk immune cells in the pancreas (Figure 2D). After establishing the R signature, we further investigated the cell types contributing to its expression. In peripheral blood, we found that, apart from NK/CD8^+^ T cells, innate immune cells like monocytes, DCs, and plasmacytoid DCs (pDCs) expressed the R signature, as evidenced by their high scores (Figure 2, E and H). CD8^+^ T cells had a memory/cytotoxic phenotype with traits of exhaustion characterized by the expression of *Tigit*, *Eomes*, and *Klrg1* (Supplemental Figure 3C), which was consistent with the gene signature described in the human AbATE study (10). The same cell types — NK/CD8^+^ T cells, monocytes, DCs, and pDCs — also expressed the R signature in the pancreas, demonstrating uniformity across tissues (Figure 2, F–H). Additionally, when examining individual genes comprising the R signature, we observed a concordant expression pattern between peripheral blood and the pancreas for each cell type (Figure 2G).

### A gene signature enriched in neutrophils in peripheral blood and pancreas correlates with anti–mouse CD3 therapy resistance in NOD mice.

**Figure 3:**
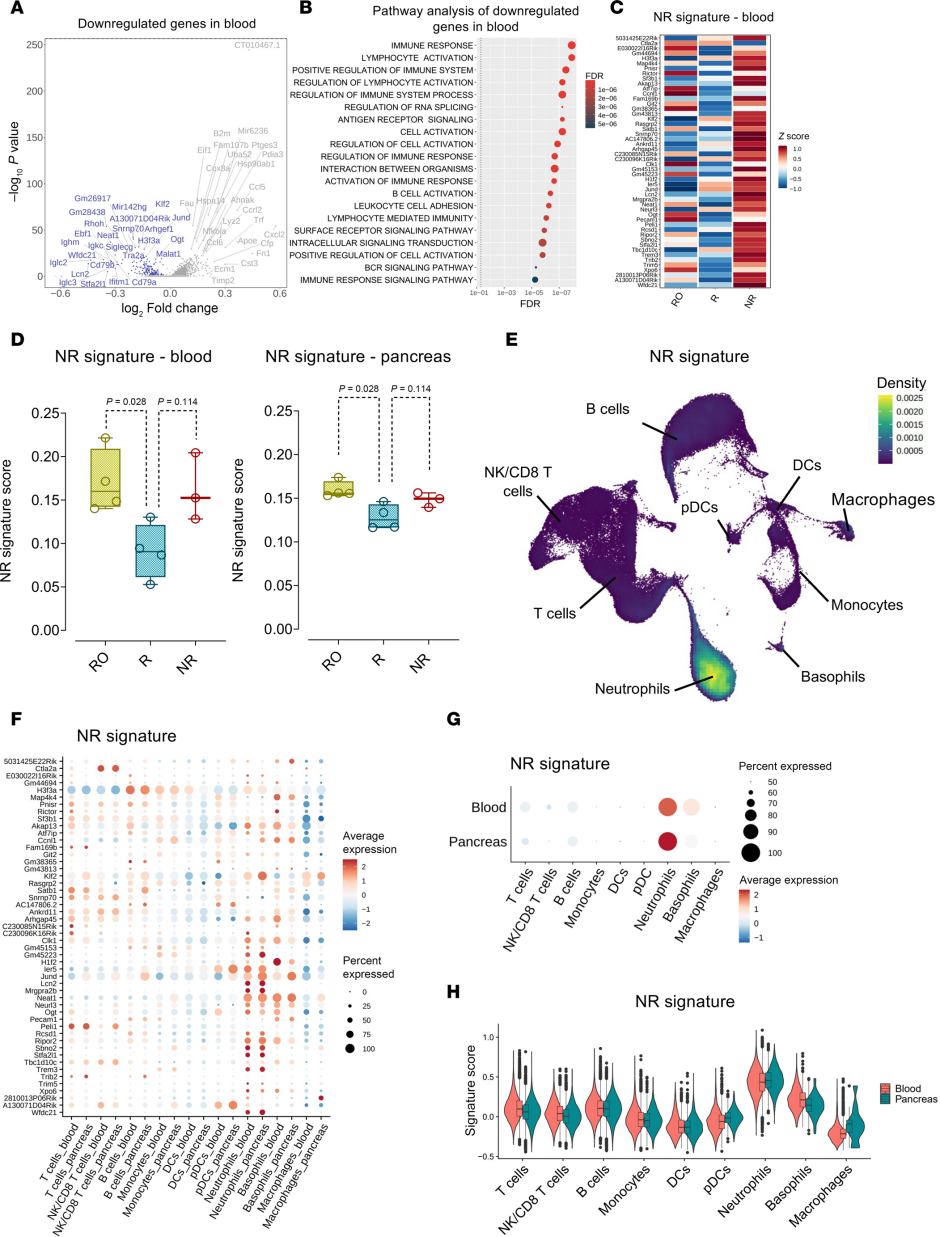
Blood-based NR signature in anti–mouse CD3 R mice and its cellular origin. (**A**) Volcano plot illustrating DEGs comparing peripheral blood leukocytes of R and NR samples. Blue dots and labels indicate downregulated genes, while gray dots and labels indicate upregulated genes that were statistically significant (*P* < 0.05). The log-transformed FC values are reported on the *x* axis, and –log_10_-transformed values of the adjusted *P* values are shown on the *y* axis. *P* values were obtained by Wilcoxon rank-sum test with Bonferroni correction. (**B**) hypeR pathway analysis performed on downregulated DEGs in peripheral blood leukocytes of R samples. Assessment of relevant pathways was conducted utilizing the hallmark gene sets from the MSigDB as a reference. (**C**) Heatmap presenting normalized expression levels of the 50 genes comprising the NR signature, represented using the *z* score. (**D**) Box plots representing the NR signature score calculated in peripheral blood (left) and pancreas (right) of RO, R, and NR samples. Mean expression of the genes comprising the NR signature in each individual cell was calculated using the Seurat function AddModuleScore. Boxes represent the median (center line) and extend from the 25th to 75th percentiles (bottom and top lines, respectively); whiskers extend from the minimum to the maximum value. (**E**) UMAP plot displaying the expression of the NR signature in the main immune cell types in peripheral blood and pancreas. (**F**) Dot plot showing the expression of individual genes comprising the NR signature in the main immune cell types in peripheral blood and pancreas. (**G**) Dot plot showing the average expression of the NR signature in the main immune cell types in peripheral blood and pancreas. (**H**) Violin plots comparing the expression levels of the NR signature in the main immune cell types in peripheral blood and pancreas. In **D**, the Mann-Whitney *U* test was used for statistical comparison.

GO pathway analysis identified that the genes downregulated in R compared with NR samples were linked to various pathways, including lymphocyte cell activation, positive regulation of cell activation, and lymphocyte-mediated immunity (Figure 3, A and B, and Supplemental Table 3). A ranking analysis, as determined through the Wilcoxon rank-sum test with Bonferroni correction (*P* < 0.05), of the downregulated genes in R compared with NR samples led to the selection of the top 50 downregulated genes with the highest discriminatory capacity in terms of anti–mouse CD3 therapy resistance, constituting the NR signature (Figure 3C). The signature score was calculated as described above. In both tissues, R samples had significantly lower scores compared with RO samples. R samples also had a lower score, yet not reaching statistical significance, when compared with NR samples (Figure 3D). The downregulated genes associated with anti–mouse CD3 therapy resistance were also downregulated in the pancreas samples. We identified neutrophils as the main contributors to the NR signature (Figure 3E). Both peripheral blood and pancreas-infiltrating neutrophils predominantly expressed these genes, with a similar expression level for most of them across both compartments (Figure 3G). The UMAP projection reflected these results, with a strong and uniform expression in neutrophils in both peripheral blood and pancreas and a mild expression of the NR signature in basophils in peripheral blood only (Figure 3, F–H).

### NK/CD8^+^ T cell– and neutrophil-enriched gene signatures correlate with C-peptide kinetics 6 months following teplizumab therapy in stage 3 T1D.

**Figure 4:**
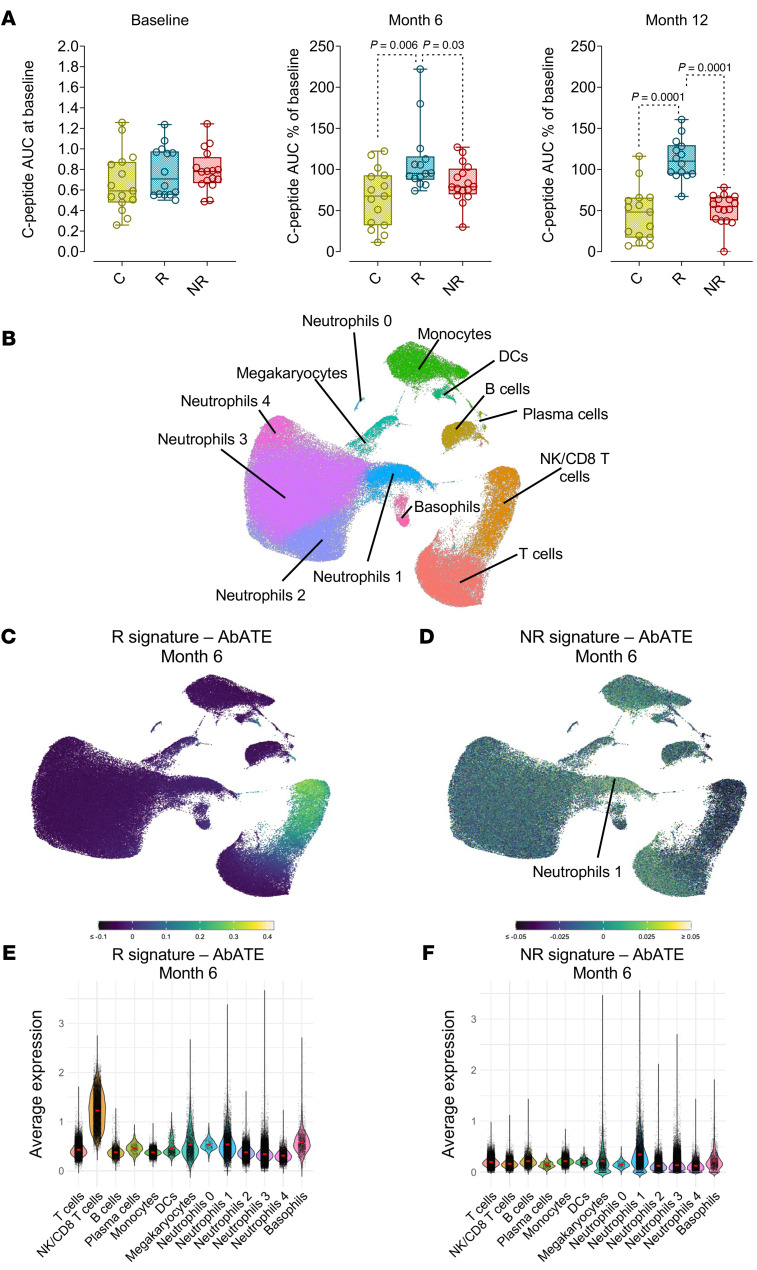
R signature and NR signature 6 months after teplizumab therapy in stage 3 T1D and their cellular origin. (**A**) Box plots representing the C-peptide AUC as absolute values at baseline (left panel), the percentage relative to baseline at 6 months (middle panel), and 12 months (right panel) after teplizumab therapy. Plots show data for untreated controls (C) (yellow, *n* = 15), R (blue, *n* = 14), and NR (red, *n* = 16). Boxes represent the median (center line) and extend from the 25th to 75th percentiles (bottom and top lines, respectively); whiskers extend from the minimum to the maximum value. (**B**) UMAP plot of immune cells from the peripheral blood of stage 3 T1D donors (*n* = 4). Clusters are color coded to define the different cell types identified using top variable genes and unsupervised clustering. (**C**) UMAP plot displaying the expression of the R signature – AbATE Month 6 in peripheral blood immune subsets. (**D**) UMAP plot displaying the expression of the NR signature – AbATE Month 6 in peripheral blood immune subsets. (**E**) Violin plot showing the average expression of the R signature – AbATE Month 6 in peripheral blood immune subsets. (**F**) Violin plot showing the average expression of the NR signature – AbATE Month 6 in peripheral blood immune subsets. In **A**, the Mann-Whitney *U* test was used for statistical comparison.

To explore whether parallel immune communities linked to anti–mouse CD3 therapy outcomes were present in humans, we analyzed published bulk whole blood RNA-Seq data from the AbATE study, in which two 14-day courses of teplizumab, 12 months apart, were evaluated in individuals with stage 3 T1D (10). We first assessed the kinetics of the C-peptide AUC at baseline, 6 months, and 12 months after the start of teplizumab therapy, but prior to the administration of the second course of teplizumab. While the C-peptide AUC was comparable between the groups at baseline, the clinical outcome was already evident 6 months after teplizumab therapy, with a significant difference between Rs (*n* = 14) and both NRs (*n* = 16) and untreated controls (Cs) (*n* = 15) (Figure 4A). A continuous decrease in C-peptide levels persisting until 24 months after therapy initiation was observed in the Cs as well as in the NRs, despite a second teplizumab regimen initiated 12 months after the first regimen in the latter group (Supplemental Figure 4).

We next analyzed at the patient level the correlation between gene expression and the C-peptide AUC percentage of baseline, 6 months after teplizumab therapy. This analysis unveiled 274 genes positively correlated with the C-peptide AUC at 6 months, constituting the R signature – AbATE Month 6. The key genes associated with teplizumab response within the R signature – AbATE Month 6 (Supplemental Table 4) overlapped with the previously described *EOMES*-associated signature identified in the AbATE study (10). We cross-connected the R signature – AbATE Month 6 with whole blood scRNA-Seq data from individuals with stage 3 T1D (Figure 4B). Cell type identities were determined on the basis of the differential expression of signature genes (Supplemental Figure 5 and Supplemental Table 5). Substantiating previous findings, NK/CD8^+^ cytotoxic T cells with characteristics of exhaustion, defined by the expression of *TIGIT*, *EOMES*, and *KLRG1* (Supplemental Figure 6A), were associated with clinical response to teplizumab 6 months after therapy, as also indicated by their elevated scores for the R signature – AbATE Month 6 (Figure 4, C–E).

We also discovered 112 genes that were negatively correlated with the C-peptide AUC percentage of baseline 6 months after teplizumab therapy, forming the “NR signature – AbATE Month 6” (Supplemental Table 4). We cross-connected the NR signature – AbATE Month 6 with whole blood scRNA-Seq data from individuals with stage 3 T1D and exposed neutrophils as the main contributors to the NR signature – AbATE Month 6. We annotated 5 neutrophil subsets on the basis of key DEGs: neutrophils 0 (expressing *CAMP*, *LNC2*, *MMP9*, *PAD4, S100A12*); neutrophils 1 (expressing *ADGRG1, ADGRG3, SMPD3*); neutrophils 2 (expressing *ADGRG3*, *CSF3R*, *CXCR2*, *IL1B, MMP9*, *PAD4*, *S100A12*); neutrophils 3 (expressing *CSF3R*, *CXCR2*, *IL1B*); and neutrophils 4 (expressing *IFIT1*, *HERC5*, *IL1B, RSAD2*). These neutrophil subsets were further characterized according to select markers of maturation (*CFS2RB*, *CSF3R*, *CXCR1*, *CXCR2*); inflammation (*CXCL8, SOD2*, *IL-1B*, *TLR1*, *TNFAIP2*); IFN signaling (*HERC5*, *IFIT1-5*, *IFI44, ISG15*); and antigen presentation (*HLA-DRA*, *HLA-DRB1*, *HLA-DM*, *CD74*) (14–16) (Supplemental Figure 6B). On the basis of this classification, we found that the neutrophils 1 subcluster expressing antigen-presenting markers, typically expressed by DCs, was the main cell type contributing to the expression of the NR signature – AbATE Month 6 (Figure 4, D–F).

### A neutrophil-enriched baseline gene signature is linked to teplizumab resistance in stage 3 T1D.

**Figure 5:**
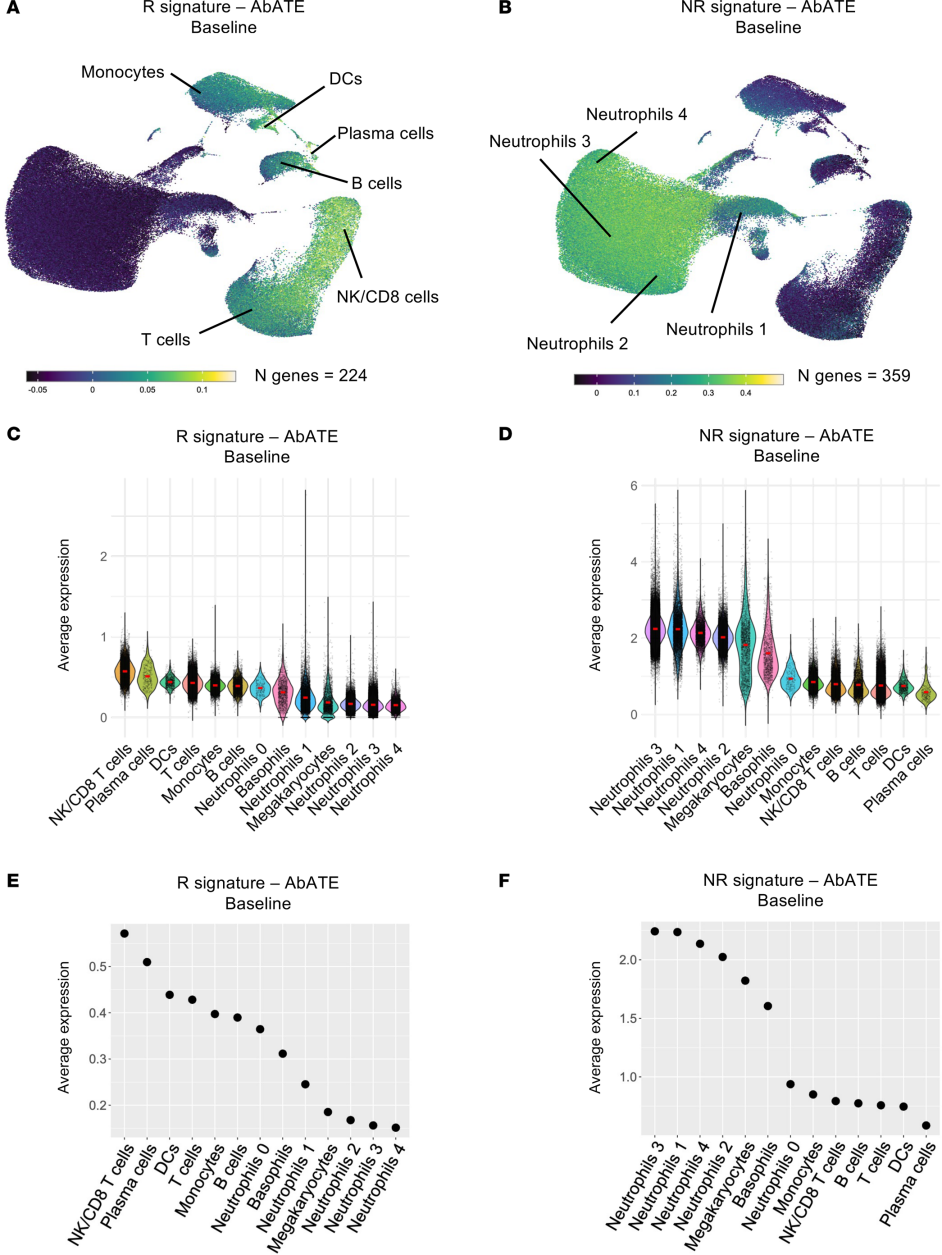
R signature and NR signature prior to teplizumab therapy in stage 3 T1D and their cellular origin. (**A**) UMAP plot displaying the expression of the R signature – AbATE Baseline in peripheral blood immune subsets. (**B**) UMAP plot displaying the expression of the NR signature – AbATE Baseline in peripheral blood immune subsets. (**C**) Violin plot showing the average expression of the R signature – AbATE Baseline in peripheral blood immune subsets. (**D**) Violin plot showing the average expression of the NR signature – AbATE Baseline in peripheral blood immune subsets. (**E**) Dot plot illustrating the average expression of the R signature – AbATE Baseline across peripheral blood immune subsets, ranked from highest to lowest expression. (**F**) Dot plot illustrating the average expression of the NR signature – AbATE Baseline across peripheral blood immune subsets, ranked from highest to lowest expression.

Despite the success of teplizumab in stage 3 T1D, its efficacy remains restricted to a subset of patients, highlighting the need for predictive markers in clinical trial design. Therefore, we reanalyzed bulk whole blood RNA-Seq data collected from the participants treated in the AbATE trial prior to the start of therapy. We correlated gene expression at baseline with changes in the C-peptide AUC percentage of baseline at 12 months after teplizumab therapy. This analysis revealed 224 genes positively correlated and 359 genes negatively correlated at baseline with the C-peptide AUC, representing the “R signature – AbATE Baseline” and the “NR signature – AbATE Baseline,” respectively (Supplemental Table 6). Notably, NK/CD8^+^ T cells and plasma cells, followed to a lesser extent by T cells, B cells, monocytes, and DCs, emerged as the primary contributors to the R signature – AbATE Baseline (Figure 5, A–C). Interestingly, neutrophils exhibited the highest expression of the NR signature – AbATE Baseline (Figure 5B), particularly the neutrophil subclusters 1–4 (Figure 5D and Supplemental Figure 5B). These patterns were further underscored when we ranked the immune subsets according to baseline expression of the R signature – AbATE Baseline and NR signature – AbATE Baseline (Figure 5, E and F).

### A neutrophil-enriched baseline gene signature is linked to teplizumab resistance in stage 2 T1D.

**Figure 6:**
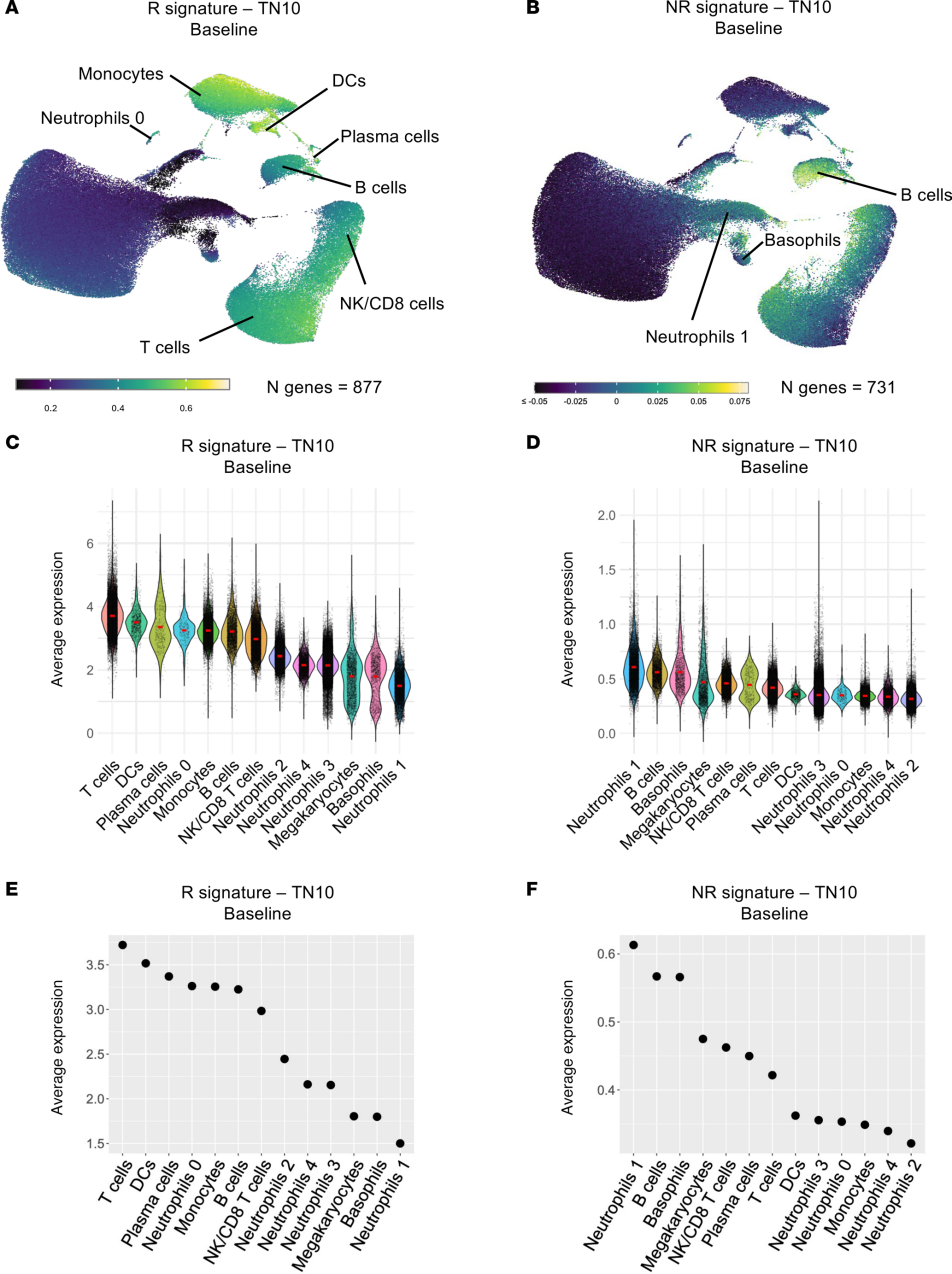
R signature and NR signature prior to teplizumab therapy in stage 2 T1D and their cellular origin. (**A**) UMAP plot displaying the expression of the R signature – TN10 Baseline in peripheral blood immune subsets. (**B**) UMAP plot displaying the expression of the NR signature – TN10 Baseline in peripheral blood immune subsets. (**C**) Violin plot showing the average expression of the R signature – TN10 Baseline in peripheral blood immune subsets. (**D**) Violin plot showing the average expression of the NR signature – TN10 Baseline in peripheral blood immune subsets. (**E**) Dot plot illustrating the average expression of the R signature – TN10 Baseline across peripheral blood immune subsets, ranked from highest to lowest expression. (**F**) Dot plot illustrating the average expression of the NR signature – TN10 Baseline across peripheral blood immune subsets, ranked from highest to lowest expression.

As teplizumab is presently only approved to delay the onset of stage 3 T1D in individuals with stage 2 disease, we also reanalyzed bulk whole blood RNA-Seq data from the TN10 study, in which teplizumab was evaluated in individuals with stage 2 T1D (1), correlating baseline gene expression profiles with time to clinical diagnosis. Given the variability in disease progression rates among placebo-treated individuals, we excluded genes correlated with disease progression in the placebo group. This identified 877 genes positively and 731 genes negatively correlated with time to diagnosis in teplizumab-treated participants, defining the “R signature – TN10 Baseline” and “NR signature – TN10 Baseline,” respectively (Supplemental Table 7). As in the AbATE study, the R signature was enriched in T cells, B cells, monocytes, and DCs (Figure 6, A–C). Interestingly, the NR signature was predominantly expressed by the neutrophils 1 subcluster, as well as by B cells and basophils (Figure 6, B–F).

### Cross-study evidence reveals a neutrophil-enriched baseline gene signature that is associated with teplizumab resistance across different stages of T1D.

**Figure 7:**
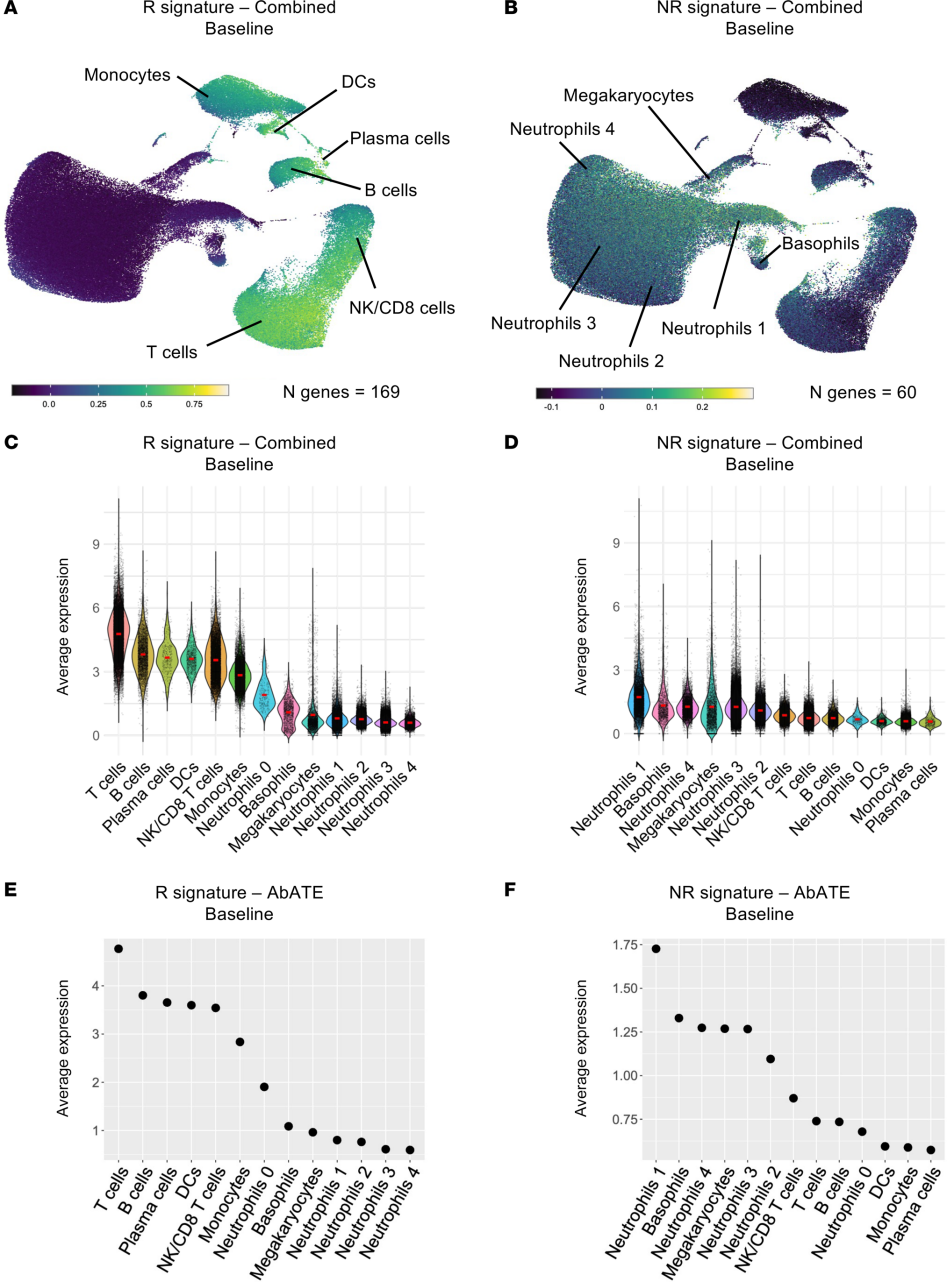
Combined R and NR signatures prior to teplizumab therapy in stage 2 and 3 T1D and their cellular origin. (**A**) UMAP plot displaying the expression of the R signature – Combined Baseline in peripheral blood immune subsets. (**B**) UMAP plot displaying the expression of the NR signature – Combined Baseline in peripheral blood immune subsets. (**C**) Violin plot showing the average expression of the R signature – Combined Baseline in peripheral blood immune subsets. (**D**) Violin plot showing the average expression of the NR signature – Combined Baseline in peripheral blood immune subsets. (**E**) Dot plot illustrating the average expression of the R signature – Combined Baseline across peripheral blood immune subsets, ranked from highest to lowest expression. (**F**) Dot plot illustrating the average expression of the NR signature – Combined Baseline across peripheral blood immune subsets, ranked from highest to lowest expression.

We next investigated whether the baseline response signatures from the stage 2 and stage 3 T1D studies shared common DEGs. We found 169 overlapping genes positively correlated and 60 genes negatively correlated at baseline with outcomes, representing the “R signature – Combined Baseline” and “NR signature – Combined Baseline,” respectively. Apart from NK/CD8^+^ T cells, other T cells, B cells, monocytes, and DCs also expressed the R signature – Combined Baseline (Figure 7, A and C), whereas neutrophils were the main cell types expressing the NR signature – Combined Baseline (Figure 7, B and D). Further support for these patterns came from ranking immune cell subsets according to their baseline expression of the 2 signatures (Figure 7, E and F).

### A robust feature selection and predictive elastic net logistic regression modeling pipeline identifies a 26-gene predictive signature to teplizumab response in stage 2 and 3 T1D.

**Figure 8:**
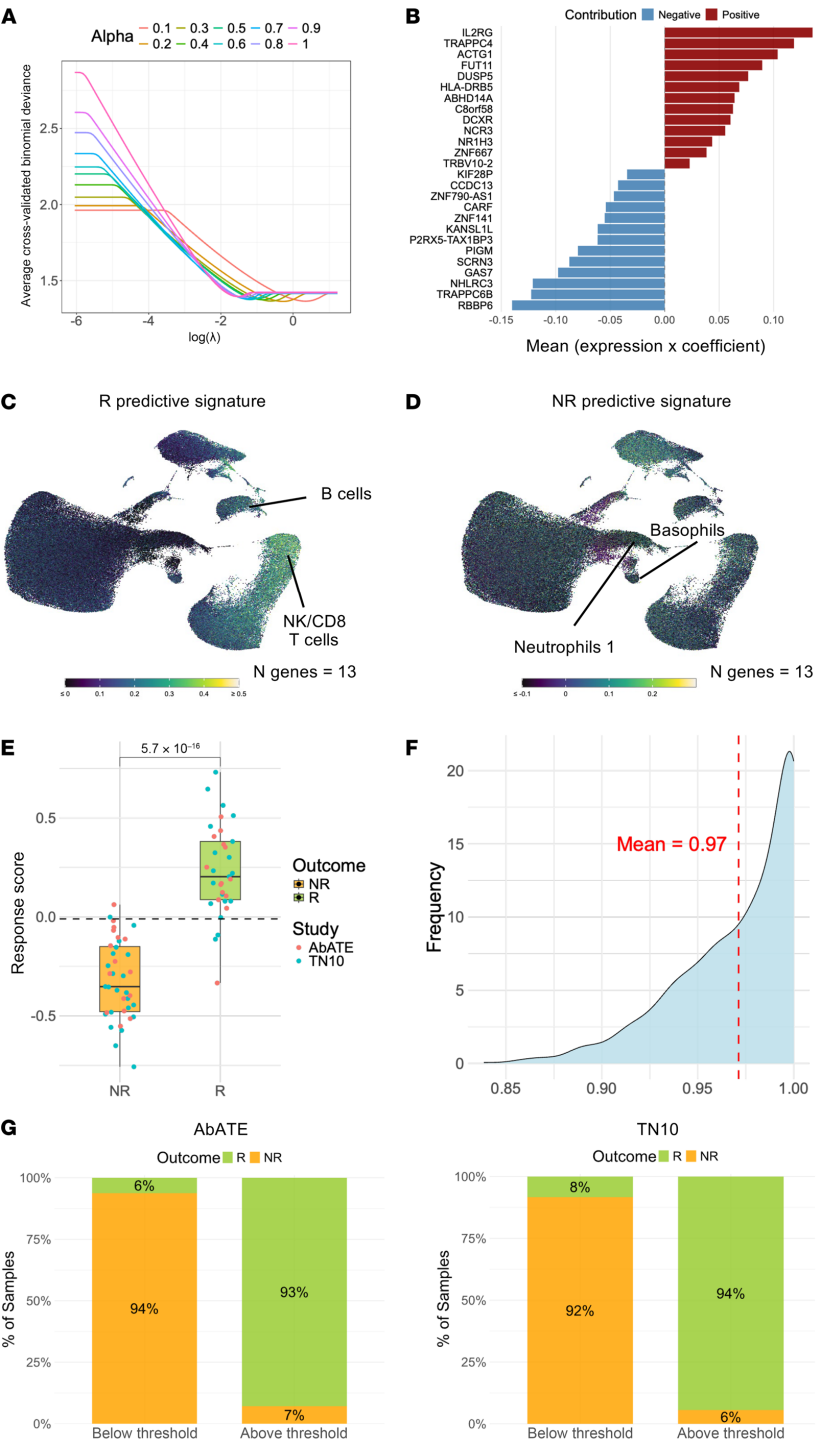
Baseline predictive response score associated with a clinical response to teplizumab outcome in stage 2 and 3 T1D. (**A**) Average cross-validated deviance changes with λ for different α values across all resampling. (**B**) Effective weight of the response score features. Each bar represents the mean absolute contribution of an individual gene to the overall score across all samples, reflecting the gene’s relative importance in the predictive model. (**C**) UMAP plot displaying the expression of the R predictive signature in peripheral blood immune subsets. (**D**) UMAP plot displaying the expression of the NR predictive signature in peripheral blood immune subsets. (**E**) Box plots depicting the response score at baseline in Rs (green box, *n* = 33) and NRs (yellow box, *n* = 39) from the AbATE (red dots, *n* = 30) and TN10 (cyan dots, *n* = 42) studies. Boxes represent the median (center line) and extend from the 25th to 75th percentiles (bottom and top lines, respectively); whiskers extend from the minimum to the maximum value. (**F**) Distribution of the AUC from 1,000 samplings of 20 random individuals from the full dataset to evaluate the predictive performance of the response score. (**G**) Stacked bar charts showing the proportion of Rs and NRs with a response score below and above threshold in the AbATE and TN10 studies. In **E**, the Wilcoxon rank-sum test was used for statistical comparison.

To identify a predictive gene signature for teplizumab response in different stages of T1D, we built a pipeline based on elastic net logistic regression modeling. Briefly, both datasets were integrated, and the integrated count matrix was used to train elastic net logistic regression models. The models were trained on 70% of the samples, with 10 different penalties (α) and a regularization parameter (λ) calculated for each model to give the minimum mean cross-validated error (Figure 8A). Each model was run 100 times with a bootstrap resampling strategy to assess feature selection stability. Moreover, the training dataset was randomly resampled 200 times to assess modeling stability and to select the features that were the least sensitive to the sampling variability. The pipeline identified 26 features that were stably selected across all α and resampling, and a median coefficient was calculated for each feature (Supplemental Figure 7A and Supplemental Table 8). A “response score” could then be calculated for each sample, with 13 genes positively (*ZNF667*, *TRBV10-2*, *C8orf58*, *DUSP5*, *NR1H3*, *ABHD14A*, *FUT11*, *TRAPPC4*, *NCR3*, *DCXR*, *HLA-DRB5*, *IL2RG*, *ACTG1*) and 13 genes negatively (*GAS7*, *RBBP6*, *TRAPPC6B*, *PIGM*, *NHLRC3*, *ZNF141*, *CARF*, *KANSL1L*, *SCRN3*, *KIF28P*, *P2RX5-TAX1BP3*, *ZNF790-AS1*, *CCDC13*) influencing the score (Figure 8B) and constituting an “R predictive signature” and an “NR predictive signature,” respectively. Most genes in the R predictive signature were predominantly expressed by NK/CD8^+^ T cells and B cells (Figure 8C and Supplemental Figure 7B). In contrast, genes in the NR predictive signature showed broader expression across cell types, with the highest expression observed in the neutrophils 1 cluster and basophils (Figure 8D and Supplemental Figure 7C). The response score effectively distinguished R from NR to teplizumab treatment (Figure 8E). This score was not significantly influenced by sex or age (Supplemental Figure 7, D and E). Notably, we observed a stronger response to teplizumab in individuals who were HLA-DR4^+^ (Supplemental Figure 8G) or had anti-GAD65 antibodies (Supplemental Figure 7I). In contrast, the presence or absence of other HLA types (Supplemental Figure 7, F–H) or additional autoantibodies (Supplemental Figure 7, J–L) was not associated with treatment response.

To obtain a less biased and more robust estimate of the predictive performance of the response score on future samples, we performed 1,000 iterative receiver operating characteristic (ROC) curve analyses. In each iteration, we randomly selected 20 samples from both studies (AbATE and TN10), reshuffling the sample set each time to assess variability and generalizability across different subsets (Supplemental Figure 7M). The AUC was consistent across these 1,000 samples, and the average AUC was 0.97 (Figure 8F). Overall, the mean sensitivity was 93%, and the mean specificity was 98%. Finally, we applied our predictive response score to the AbATE and TN10 studies separately with a threshold of –0.01 and observe a 6% and 8% false-negative rate and a 7% and 6% false-positive rate, respectively (Figure 8G).

## Discussion

While prior studies have significantly advanced our understanding of how anti-CD3 therapies such as teplizumab modulate T cell responses in T1D, they have largely focused on mechanisms of clinical response (9–12, 17). These investigations have described exhausted or regulatory phenotypes in CD8^+^ and CD4^+^ T cells, reductions in T cell activation signatures over time, and prevention of autoreactive T cell expansion. However, comparatively little is known about the mechanisms of teplizumab resistance or how to identify predictive biomarkers to guide patient selection.

Using the NOD mouse model and single-cell transcriptomics, we identified 2 distinct blood-based gene signatures associated with responses to anti–mouse CD3 therapy. The R signature was enriched in NK/CD8^+^ T cells displaying transcriptional features of exhaustion, suggestive of an active immune regulatory response. In contrast, the NR signature was predominantly associated with neutrophils, indicating a distinct, potentially proinflammatory profile. Importantly, both signatures were also detected within pancreatic immune infiltrates, underscoring their potential translational relevance as biomarkers for monitoring and predicting therapeutic outcomes in autoimmune T1D.

Reanalysis of the AbATE trial involving individuals with stage 3 T1D further validated the association between NK/CD8^+^ T cell–enriched gene expression profiles and clinical response to teplizumab (10, 11). Key transcripts, such as *KLRC1*, *NKG7*, and *CD160* (expressed by NK cells), as well as *GZMA*, *GZMB*, *TIGIT*, *EOMES*, and *KLRG1* (expressed by CD8^+^ T cells), were elevated in Rs 6 months after therapy initiation. A similar population of exhausted CD8^+^ T cells was detected in the pancreatic immune infiltrates of anti–mouse CD3–treated R mice, suggesting localized engagement of inhibitory pathways that modulate pathogenic effector T cell activity at the site of autoimmune inflammation. Transcriptomics profiling from the TN10 study of individuals with stage 2 T1D revealed an early activation of CD8^+^ T cells following teplizumab administration, which progressively transitioned into regulatory and exhausted effector phenotypes by 18 months (9). Although NK cells have traditionally been seen as cytotoxic “immune enforcers,” emerging data highlight their context-dependent immunoregulatory roles, including inducing CD8^+^ T cell exhaustion and suppressing autoreactive responses within pancreatic tissue (18, 19). Also in multiple sclerosis, specific cytotoxic NK cell responses, in particular NKG2C^+^ and NKG2D^+^ NK cell populations have been shown to correlate with the control of autoreactive cells (20). Additionally, a recent study described NK-like CD57^+^ and PD-1^+^CD8^+^ exhausted-like T cell populations in T1D, revealing shared epigenetic features but also distinct characteristics, such as unique chromatin accessibility in CD57^+^ cells, particularly for iKIR (inhibitory killer cell immunoglobulin-like receptor) genes (21). Similar exhausted-like phenotypes have also been associated with preserved C-peptide levels in alefacept-treated T1D patients (22), and low-dose ATG (anti–thymocyte globulin) therapy has been shown to induce CD4^+^ T cell exhaustion (23), further supporting T cell exhaustion as a shared mechanism of immune modulation across distinct immunotherapies in T1D.

In contrast to the well-studied mechanisms underlying teplizumab success, no studies have systematically addressed teplizumab resistance. Our identification of a second gene signature associated with nonresponse, predominantly enriched in neutrophils, adds a new perspective on alternative immune pathways. This signature was especially prominent in the human neutrophils 1 subcluster characterized by high expression of adhesion GPCRs (*ADGRG1* [EMR1, F4/80]*, ADGRG3* [EMR3] and *ADGRG5* [CD97]) and antigen-presenting markers (*HLA-DRA*, *HLA-DRB1*, *HLA-DM*, and *CD74*), indicative of “hybrid” neutrophils with both innate and adaptive features (24, 25). These cells, known for heightened cytokine production, phagocytic activity, and T cell–stimulatory potential, may act as antigen transporters to draining lymph nodes, fostering effector T cell responses. Although it remains unclear whether these cells actively drive therapy resistance or emerge because of treatment, to our knowledge, this is the first study to link peripheral neutrophils to teplizumab resistance in stage 3 T1D.

While post-treatment analyses have provided key insights into immune changes associated with teplizumab response, the identification of predictive biomarkers remains essential for determining which individuals are most likely to benefit prior to therapy initiation. Using baseline data from the TN10 (stage 2) and AbATE (stage 3) clinical trials, we identified blood-based gene expression signatures that distinguished teplizumab Rs from NRs. The R signature – Combined Baseline was enriched not only in NK/CD8^+^ T cells, but also in other T cell subsets, B cells, monocytes, and DCs. Given that CD8^+^ T cell exhaustion is linked to slower disease progression (26) and positively modulated by teplizumab (9, 10), this signature could serve as a tool for stratifying patients based on their immune phenotypes, thereby optimizing treatment strategies to delay T1D progression. In contrast, the NR signature – Combined Baseline was predominantly expressed by neutrophils. These neutrophil-associated signatures exhibited disease stage-specific variation. The neutrophils cluster 1, defined by elevated expression of genes associated with MHC class II–mediated antigen presentation, exhibited gene signatures linked to teplizumab resistance in both stage 2 and stage 3 T1D. As mentioned earlier, these cells exhibit hybrid features seen in other diseases, including DC-like morphology, cytokine production, and antigen presentation to naive CD4^+^ T cells (15, 24, 27). In contrast, clusters 2–4 were more prominent in stage 3 T1D and resembled mature, inflammatory neutrophils expressing IFN-stimulated genes. Previous studies have shown that peripheral neutrophils from individuals at risk for, or newly diagnosed with, T1D overexpress genes downstream of both type I IFN-α and type II IFN-γ signaling pathways (28). Evidence from human and mouse studies demonstrated that IFN-α can enhance HLA class I expression in islets, promoting β cell antigen presentation and CD8^+^ T cell–mediated cytotoxicity in T1D (29, 30). An IFN-associated neutrophil phenotype may undermine the immunomodulatory effects of teplizumab by sustaining antigen presentation and inflammatory signaling in the islets, thereby maintaining cytotoxic T cell activity and β cell destruction despite therapy.

Next, we developed a robust elastic net logistic regression pipeline that identified a 26-gene signature predictive of teplizumab response in stage 2 and 3 T1D. This gene signature includes 2 distinct sets of genes positively and negatively associated with treatment response. The predictive response score accurately distinguished teplizumab treatment Rs from NRs with high sensitivity and specificity, independent of sex or age. Notably, individuals positive for HLA-DR4 demonstrated an enhanced response to teplizumab, consistent with prior studies reporting improved outcomes in HLA-DR4^+^ and HLA-DR3^–^ participants (1), which we hypothesize reflects the role of MHC molecules in shaping the T cell repertoire and activation patterns. Furthermore, the presence of anti-GAD65 antibodies, previously linked to lower risk in autoantibody-positive populations (31), was associated with variations in the response score.

While the immune communities associated with therapy success and resistance in both the mouse model and human studies were largely comparable, their underlying gene signatures, particularly those expressed by various neutrophil subsets, differed significantly. Although the hamster anti–mouse 145-2C11 clone may trigger immune responses distinct from humanized anti-CD3 antibodies, both share the ability to initially activate T cells. Our data suggest that the pancreatic microenvironment harbors distinct neutrophil states, indicating that broad depletion strategies may eliminate both detrimental and potentially beneficial subsets. It remains unclear whether these neutrophils actively drive resistance or merely reflect an underlying immune state. A deeper understanding of the mechanisms that drive the acquisition of specific neutrophil states will be essential for selectively manipulating these populations and obtaining a more nuanced picture of their role in immunotherapy. Importantly, our predictive model has not yet been validated in independent cohorts. Future work should prioritize external validation and refine neutrophil subset characterization using specific markers to improve patient stratification and clarify the functional role of these neutrophil subsets in resistance. Together, these efforts will support the development of a robust, personalized framework for predicting teplizumab responsiveness in individuals with T1D.

In this study, we elucidate mechanisms underlying the clinical response to teplizumab by identifying transcriptional signatures predictive of therapeutic outcome and mapping them to specific immune cell populations. Gene expression profiles associated with Rs were predominantly enriched in NK/CD8^+^ T cells, whereas NR signatures were primarily enriched in neutrophils. These findings underscore a potential role for innate immune pathways in mediating resistance to teplizumab and suggest that enhancing teplizumab efficacy may require complementary approaches targeting additional immune subsets, particularly in NRs. Evaluating this hypothesis in clinical settings will also necessitate a shift in sampling strategies that opt for whole blood over isolated PBMCs to more comprehensively capture the role of the granulocyte lineage in treatment response. Ultimately, our data support the development of precision, multitargeted immunotherapies for T1D and lay the groundwork for identifying likely Rs before treatment initiation.

## Methods

### Sex as a biological variable.

Our study exclusively examined male mice. Although male and female NOD mice have a different rate of disease incidence, being more frequent in females than in males, the response to anti–mouse CD3 has been shown to be similar in both sexes (32, 33). Published human data are from both male and female participants.

### Animals.

NOD mice have been inbred at the KU Leuven animal facility (Leuven, Belgium) since 1989 and are kept under semi-barrier conditions. Mice were screened for diabetes onset 3 times a week, checked for glucosuria (Diastix Reagent Strips, Bayer) and nonfasting glycemia values (AccuCheck, F. Hoffmann-La Roche) and were considered diabetic if glycemia values exceeded 11.1 mmol/L for 2 consecutive days.

### Anti–mouse CD3 therapy in RO NOD mice.

RO NOD mice were bled via the submandibular vein and treated for 5 consecutive days with 2.5 μg/d hamster anti–mouse CD3 mAb (clone 145-2C11, BioXCell) and monitored for a period of 28 days (CITE-Seq and follow-up) (Figure 1A). Mice that returned to normoglycemia until 28 days after therapy initiation were considered Rs. Mice that did not return to normoglycemia after therapy initiation were considered NRs. All mice were sacrificed according to humane endpoints (i.e., 20% weight loss or 3 consecutive maximum blood glucose measurements of 33.3 mmol/L).

### Immune subset staining and sorting.

Single-cell suspensions from NOD pancreases were prepared in digestion medium (RPMI medium plus 20% FCS plus 1% nonessential amino acids solution plus 1 mM Na-pyruvate plus 2 mM MgCl_2_ plus 2 mM CaCl_2_; MilliporeSigma/Thermo Fisher Scientific) for 20 minutes in a shaking incubator at 250 rpm at 37°C. Peripheral blood was diluted with PBS, and RBCs were lysed with NH_4_Cl. The cells from peripheral blood and pancreas were incubated for 10 minutes with Fc block to block nonspecific antibody binding and then stained with a pool of 190 barcoded TotalSeq A antibodies (BioLegend) for 30 minutes at 4°C, mixed with an anti-CD45 antibody (clone 30-F11, Thermo Fisher Scientific). After labeling, cells were washed with FACS buffer, and CD45^+^ immune cells were sorted with the BD FACS Aria II cell sorter (BD Biosciences). Cells were sorted, spun down, and resuspended in PBS with 0.04% BSA at an estimated final concentration of 1,000 cells/μL.

### CITE-Seq of peripheral blood and pancreatic immune cells from NOD mice.

CD45^+^ sorted immune cells from NOD samples (target recovery of 10,000 cells) were loaded onto a GemCode Single-cell Instrument (10x Genomics) to generate single-cell Gel Bead-in-EMulsions (GEMs). scRNA-Seq libraries were prepared using GemCode Single-cell 3′ V3.1 Gel Bead and the Library Kit (10x Genomics) according to the manufacturer’s instructions. The cDNA amplification mix was modified to include ADT PCR additive primer (5′-CCTTGGCACCCGAGAATT*C*C-3′, where the asterisk indicates a phosphonothioate bond) and HTO PCR additive primer (5′-GTGACTGGAGTTCAGACGTGTGC*T*C-3′). Size selection via a SPRIselect Reagent kit (Beckman Coulter) was used to separate the amplified cDNA molecules for 3′ gene expression and cell-surface protein library construction. Sequencing libraries were loaded on an Illumina HiSeq 4000 or an Illumina NovaSeq 6000 flow cell at VIB Nucleomics Core (Leuven, Belgium), with the sequencing settings recommended by 10x Genomics (read 1: 26 cycles; read 2: 98 cycles; index i7: eight cycles; index i5: no cycles, pooled at a 75:20:5 ratio with a 115 pM loading concentration plus 1% PhiX for the combined 3′ gene expression and cell-surface/hashing libraries, respectively). Raw sequencing reads were demultiplexed using the Cell Ranger pipeline (10x Genomics,) and gene expression and cell-surface protein matrices were created. These matrices were processed with Seurat, version 4.1.0. Cells expressing more than 200 genes were retained. Subsequent filtering based on the number of features, the unique molecular identifiers (UMIs), and the percentage of mitochondrial DNA was applied. As a result from the Cell Ranger, the average of the mean reads per cell across all the libraries was 72,793, with an average sequencing saturation of 53%. Overall, a CITE-Seq dataset of 43,706 single cells, 190 surface markers, and 24,987 different genes was analyzed in this study.

### scRNA-Seq of peripheral blood in recent-onset T1D.

Peripheral blood samples from newly diagnosed T1D donors were collected in EDTA-coated tubes. RBCs were lysed using RBC Lysis buffer (Roche, 11814389001) according to the manufacturer’s instructions. Briefly, 2 mL whole blood was resuspended in 4 mL RBC Lysis buffer and incubated on a shaker (700 rpm) for 15 minutes. After centrifugation (5 minutes at 500*g*), the pellet was resuspended in 4 mL RBC Lysis buffer and spun down again. Cells were resuspended in PBS with 1 mM EDTA (Corning, 46-034-Cl). All procedures were conducted at room temperature and under sterile conditions. Isolated cells were then kept on ice before proceeding to the preparation for single-cell sequencing. Samples were counted on a LUNA-FL Automated Cell Counter (Logos Biosystems) and were loaded onto a BD Rhapsody cartridge (BD Biosciences) with a targeted capture of 25,000–40,000 cells. Reverse transcription, cDNA amplification, and library construction were performed following the manufacturer’s instructions (BD Biosciences, catalog 23-24117[02]). Libraries were sequenced on a NovaSeq 6000 or a NextSeq 2000 flow cell (Illumina). The BD Rhapsody Sequence Analysis Pipeline (BD, version 1.12) was used to map the FASTQ files to the human reference genome (GRCh38).

### Immune subset profiling using the Seurat R toolkit.

The CITE-Seq data from mouse samples and the scRNA-Seq data from human samples, both free of unwanted cells, were processed to construct a Seurat object using Seurat, version 4.1.0 (34). Data were normalized using natural-log and centered-log ratio normalization for mRNA-based and ADT-based clustering, respectively. For the scRNA-Seq data of human samples, only the natural-log normalization for mRNA-based clustering was performed. To identify the main cell types of the dataset, we clustered the cells with PCA linear dimensional reduction and UMAP nonlinear dimensional reduction for visualization. No batch correction was performed at this stage. The different immune cell types were annotated on the basis of differential expression of genes and surface proteins, performing the Wilcoxon rank-sum test using the Seurat function “FindAllMarkers” with a default log fold change (FC) threshold of 0.25, testing only the genes and proteins that were detected in a minimum of 25% of cells. *P* value adjustment was performed using Bonferroni correction. Clustering based on protein and gene expression data revealed different lymphoid and myeloid lineage immune cells in both datasets.

### Differential expression and pathway analysis of CITE-Seq data.

DEGs comparing peripheral blood leukocytes in Rs and NRs were identified by performing the Wilcoxon rank-sum test using the Seurat function FindMarkers without a logFC threshold, testing only the genes that were detected in a minimum of 25% of cells. *P* value adjustment was performed using Bonferroni correction. Mitochondrial and ribosomal genes were filtered out from the analysis to minimize potential noise from housekeeping functions and enhance the focus on genes specifically associated with immunological pathways. The R package hypeR, version 1.14.0, was used to perform the pathway analysis (35). Only the upregulated and downregulated DEGs in peripheral blood of R mice with the highest significant adjusted *P* value and absolute log_2_FC of 0.5 or greater were included in the analysis. Furthermore, mitochondrial and ribosomal genes were filtered out from the selected DEGs. The Molecular Signatures Database (MSigDB) hallmark gene sets were used as a reference to identify the pathways in which the DEGs are involved.

### Gene signatures in NOD mice.

DEGs comparing peripheral blood leukocytes in Rs and NRs were identified using the Wilcoxon rank-sum test and the Seurat function FindMarkers without a logFC threshold, testing only the genes that were detected in a minimum of 25% of cells. Bonferroni correction was applied to adjust the *P* values obtained from the differential gene expression analysis to account for multiple testing. A *P* value of 0.05 or less was used as the significance threshold. The DEGs showing significant upregulation were used to create the R signature, whereas the genes with significant downregulation were used for the NR signature. The Wilcoxon nonparametric rank-sum test was used to rank the DEGs according to their discriminatory power between Rs and NRs. The top 50 ranked genes were selected for both the R signature and the NR signature, as they exhibited the most pronounced differences in expression levels.

### The AbATE study of stage 3 T1D.

Bulk whole blood RNA-Seq data from patients enrolled in the AbATE study (ClinicalTrials.gov NCT00129259) were downloaded from ITN TrialShare, a clinical trial research portal supported by the National Institute of Allergy and Infectious Disease (NIAID), NIH. Briefly, in this study, individuals with new-onset T1D (according to the American Diabetes Association [ADA] criteria) within 8 weeks prior to study entry were randomized to receive teplizumab or standard diabetes management alone. The experimental group was treated with teplizumab for the first 14 days of the study and again 1 year later. Assessment of the therapy response was based on the criterion of a 40% reduction in baseline C-peptide levels, as measured by the mean AUC during a 4-hour mixed-meal tolerance test (MMTT), 2 years after therapy initiation. Samples were collected at timed visits during the study and stored frozen until use. For RNA extraction, whole blood samples were collected into Tempus tubes (Thermo Fisher Scientific), and RNA was prepared by commercial vendors (Expression Analysis and Thermo Fisher Scientific) as previously described (10). In this study, we analyzed samples collected at baseline, 6 months, and 12 months after therapy, with a total of 136 samples, 93 of which were used for bulk whole blood RNA-Seq analysis, divided by controls (*n* = 30), Rs to teplizumab therapy (*n* = 30), and NRs to teplizumab therapy (*n* = 33).

### The TN10 study for stage 2 T1D.

In this placebo-controlled, phase 2 clinical trial (ClinicalTrials.gov: NCT01030861), nondiabetic individuals with a T1D relative from the TN01 study (ClinicalTrials.gov: NCT00097292), aged at least 8 years at the time of randomization, with at least 2 diabetes-related autoantibodies and demonstrated abnormal glucose tolerance (stage 2 T1D) were randomized 1:1 to receive either placebo or teplizumab for 14 days, with the primary outcome being the progression toward overt diabetes onset (1). Whole blood was collected for bulk whole blood RNA-Seq at baseline, day 20, and month 12. In this study, we analyzed baseline samples from a total of 73 individuals, of whom 31 received a placebo and 42 received teplizumab. We classified participants as Rs or NRs to teplizumab therapy according to their progression to stage 3 T1D within the first 12 months of the study. Rs were those who did not progress to stage 3 T1D, while NRs were those who did.

### Bulk whole blood RNA-Seq preprocessing.

The raw count matrix from the publicly available dataset on bulk whole blood RNA-Seq from the AbATE study was used to generate gene signatures comparing teplizumab therapy Rs versus NRs. The bulk whole blood RNA-Seq raw count matrix from the TN10 study was obtained following a request to the TrialNet publications committee along with the metadata. “Combat_seq” from the sva package, version 3.50.0 (36), was used to correct a batch effect that was present in the samples collected at baseline in the AbATE dataset and was also used to integrate both datasets together. The gene expression matrix was normalized using the “estimateSizeFactors” function from the DESeq2 package, version 1.42.1 (37), to account for differences in sequencing depth across samples. The genes expressed at least 10 times in a minimum 40% of the samples were kept, and the rest were excluded from the matrix.

### Gene signatures from teplizumab-treated patients with stage 3 T1D.

The raw count matrix from a publicly available bulk whole-blood RNA-Seq dataset from the AbATE study were utilized to generate gene expression signatures comparing R versus NR to teplizumab therapy. Gene signatures at 6 months were identified using Pearson correlation analysis, correlating gene expression at month 6 after teplizumab therapy with C-peptide levels, expressed as the AUC percentage of baseline at month 6. Genes showing a positive, statistically significant correlation (*P* < 0.05) were used to create the R signature – AbATE Month 6, whereas genes showing a negative statistically significant correlation (*P* < 0.05) were used to create the R signature – AbATE Month 6.

Baseline gene signatures were identified similarly, with Pearson correlation analysis correlating gene expression at baseline with the C-peptide AUC percentage of baseline at month 12 after teplizumab therapy. Genes showing a positive, statistically significant correlation (*P* < 0.01) were used to create the R signature – AbATE Baseline, and genes showing a negative statistically significant correlation (*P* < 0.01) were used to create the NR signature – AbATE Baseline.

### Gene signatures from teplizumab-treated patients with stage 2 T1D.

The raw count matrix from the TN10 study was used to generate signatures comparing R versus NR to teplizumab therapy. Baseline gene signatures were identified with Pearson correlation analysis correlating gene expression at baseline with time before progression to stage 3 T1D. The gene signatures linked to teplizumab therapy were generated by excluding genes that correlated with time before progression to stage 3 T1D in the placebo group. Genes showing a positive, statistically significant correlation (*P* < 0.05) were used to create the R signature – TN10 Baseline, and genes showing a negative statistically significant correlation (*P* < 0.05) were used to create the NR signature – TN10 Baseline.

### Baseline gene signatures from teplizumab-treated patients with stage 2 or 3 T1D.

Baseline gene signatures from both AbATE and TN10 studies were compared. The genes that significantly correlated with a positive outcome following teplizumab therapy in both studies were used to create the “R signature – Combined Baseline,” and the genes that significantly correlated with a negative outcome following teplizumab therapy in both studies were used to create the NR signature – Combined Baseline.

### Predictive model building and features selection.

To identify genes capable of reliably predicting positive or negative outcomes following teplizumab therapy in stage 2 and 3 T1D, we used elastic net logistic regression with bootstrap resampling. The analysis was conducted on baseline expression data from the combined dataset, which was randomly partitioned 200 times into training sets comprising 70% of the samples. Each partition used a unique random seed to ensure variability across the splits. Within each of the 200 training sets, we trained elastic net logistic regression models using the glmnet package (38), systematically varying the regularization mixing parameter α from 0 (pure Ridge regression) to 1 (pure Lasso regression) in increments of 0.1. For each α value and corresponding training partition, the optimal regularization strength (λ) was selected via 5-fold cross-validation using the cv.glmnet function. The objective was to minimize the binomial deviance, as the response variable was binary (R versus NR). We selected the λ value corresponding to lambda.min, defined as the value producing the minimum mean cross-validated error. Following selection of the optimal α-λ combination for each training partition, a bootstrap resampling procedure was conducted, involving 100 bootstrap iterations per combination. In each iteration, an elastic net logistic regression model was fit using the current training data, α, and λ. Genes with non-zero regression coefficients were considered selected in that bootstrap iteration. For every α and training set combination, we computed the selection frequency of each gene across the 100 bootstrap iterations. The top 33 most frequently selected genes in each scenario were defined as the stable feature set for that specific α and training partition. To derive a robust and generalizable predictive gene signature, we identified an “overlapping features” set, comprising genes that consistently appeared among the top 33 features across all α values (except α = 0, which was excluded because of consistently poor performance). Finally, to summarize the relative contribution of each gene in this robust signature, we computed the median of all non-zero coefficient values observed across all bootstrap iterations, α, and training partitions.

### Predictive signature validation.

To evaluate the predictive performance of the gene signature described above, we computed a response score for each individual. This score was calculated as the weighted sum of the expression levels of the signature genes, where each gene was multiplied by its corresponding model coefficient. To assess the signature’s ability to distinguish between Rs and NRs, we implemented a robust resampling strategy. Specifically, we randomly selected a subset of 20 individuals from the full cohort 1,000 times. For each resampled subset, we performed ROC curve analysis and recorded the AUC, along with the sensitivity and specificity values. To determine the optimal classification threshold within each subset, we identified the point on the ROC curve that maximized Youden’s J index (defined as sensitivity + specificity − 1). The corresponding threshold value was recorded for each iteration, allowing us to evaluate the stability and robustness of the classification performance across different subsets of the data.

### Statistics.

Box plots were generated and their statistical analysis performed using GraphPad Prism 9.0 (GraphPad Software). Survival curves were created by Kaplan-Meier survival analysis combined with the log-rank Mantel-Cox test. Statistical analysis of CITE-Seq and scRNA-Seq DEGs was performed with the Wilcoxon rank-sum test using the Seurat function “FindAllMarkers,” testing only the genes and proteins that were detected in a minimum of 25% of cells. *P* value adjustment was performed using Bonferroni correction. An adjusted *P* value of 0.05 or less was considered significant. Pearson correlation analysis was performed using the “rcorr” function from the R package Hmisc (version 5.1-2). Differential expression analysis of bulk-whole blood RNA-Seq data was performed with the R package DESeq2 (version 1.42.1), with statistical significance determined via the Benjamini-Hochberg procedure to control the FDR, using a threshold of an adjusted *P* value of less than 0.1.

### Study approval.

All animal protocols were approved by the KU Leuven Animal Care and Use Committee (project number P068/2019). All experiments complied with the EU Directive 2010/63/EU for animal experiments.

### Data availability.

All datasets generated and analyzed in this study are available from the corresponding authors on reasonable request. Mouse CITE-Seq raw data and gene-cell count matrices were deposited in the NCBI’s Gene Expression Omnibus (GEO) database (GEO GSE307823). Human scRNA-Seq raw data and gene-cell count matrices are available on the Zenodo repository (https://www.doi.org/10.5281/zenodo.17035450). Bulk-whole blood RNA-Seq data from the AbATE study are deposited in the GEO database (GEO GSE85531), and the raw data for the TN10 study are deposited in the Zenodo repository (https://www.doi.org/10.5281/zenodo.17063348). The source code and documentation are available through GitHub (github.com/Leuven-Diabetes-Lab/Teplizumab_Neutrophil_signature). All values presented in the figures are provided in the Supporting Data Values file.

## Author contributions

All authors contributed substantially to this study. CG designed the study. GS, PL, and CG wrote the manuscript. AW, LD, and MV assisted in mouse treatment and follow-up. GS performed the CITE-Seq experiment and analyzed the data. FL and PL assisted with CITE-Seq analysis. LFC, PL, and ACC performed the bulk-whole blood RNA-Seq analysis. PL designed the predictive feature selection pipeline. NVD assisted with the sorting of immune cells from peripheral blood and pancreas and the CITE-Seq experiment. SB collected, optimized, and processed human peripheral blood for Sc-RNA-Seq. PSL, SAL, and LH helped with the AbATE data access, analysis, and interpretation. All authors critically revised and edited the manuscript. CM and CG conceptualized the research goals, acquired major funding, and discussed the data.

## Funding support

This work is the result of NIH funding, in whole or in part, and is subject to the NIH Public Access Policy. Through acceptance of this federal funding, the NIH has been given a right to make the work publicly available in PubMed Central.

- Flemish Research Foundation (FWO Vlaanderen, grants G.0C63.19N, G.0667.21N, and G.0316.25N, to CM and CG).
- KU Leuven C1 funding (C1/18/006 and C16/18/006).
- Research infrastructure grant (KA/20/077).
- Gifts for the Hippo & Friends Type 1 Diabetes fund and the Carpe Diem fund for diabetes research.
- Scholarship from the Flemish Research Foundation (FWO Vlaanderen, grant 1.1A02.20N, to SB).
- Cooperative agreements between the National Institute of Diabetes and Digestive and Kidney Diseases (NIDDK), NIH, the National Institute of Allergy and Infectious Diseases (NIAID), NIH, and the Eunice Kennedy Shriver National Institute of Child Health and Human Development (U01 DK061010, U01 DK061034, U01 DK061042, U01 DK061058, U01 DK085453, U01 DK085461, U01 DK085465, U01 DK085466, U01 DK085476, U01 DK085499, U01 DK085504, U01 DK085509, U01 DK103153, U01 DK103180, U01 DK103266, U01 DK103282, U01 DK106984, U01 DK106994, U01 DK107013, U01 DK107014, UC4 DK097835, and U01 DK106993).
- Breakthrough T1D (formerly JDRF).
- American Diabetes Association.
- National Center for Research Resources through Clinical Translational Science (UL1TR000142, UL1TR002366, UL1TR000445, UL1TR000064, UL1TR002537, UL1TR001082, UL1TR000114, UL1TR001857, UL1TR002529, UL1TR001872).
- Immune Tolerance Network (ITN) (UM1 AI09565).
- MacroGenics donated the study agents and provided funds for additional site monitoring.

## Supplementary Material

Supplemental data

Supplemental table 2

Supplemental table 3

Supplemental table 4

Supplemental table 5

Supplemental table 6

Supplemental table 7

Supplemental table 8

Supporting data values

## Version 1 09/30/2025

In-Press Preview

## Version 2 12/01/2025

Electronic publication
