# Supplementary material for: Neutrophil-enriched gene signature correlates with teplizumab therapy resistance in different stages of type 1 diabetes
Source: J Clin Invest. 2025 Sep 30;135(23):e176403. doi: 10.1172/JCI176403 (PMC12646666; doi:10.1172/JCI176403)
Supplement: Supplemental data [file jci-135-176403-s289.pdf]

Supplemental Figure 1

A

CITE-seq sample dataset

Recent-Onset (RO)	n = 4
anti-CD3 Responders (R)	n = 4
anti-CD3 Non-Responders (NR)	n = 3
total	n = 11

B

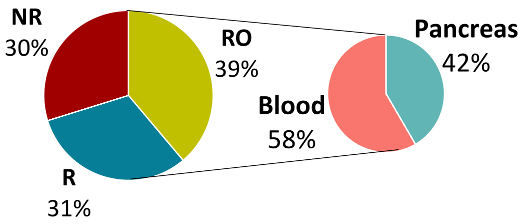

Supplemental Figure 1. CITE-sequencing dataset composition. (**A**) Biological condition and number of samples used for CITE-sequencing. (**B**) Diagram showing the proportion of cells derived from both blood and pancreas across RO (Recent-Onset), R (Responder), and NR (Non-Responder) mice. All proportions are normalized to the total cell count in the dataset.

Supplemental Figure 2

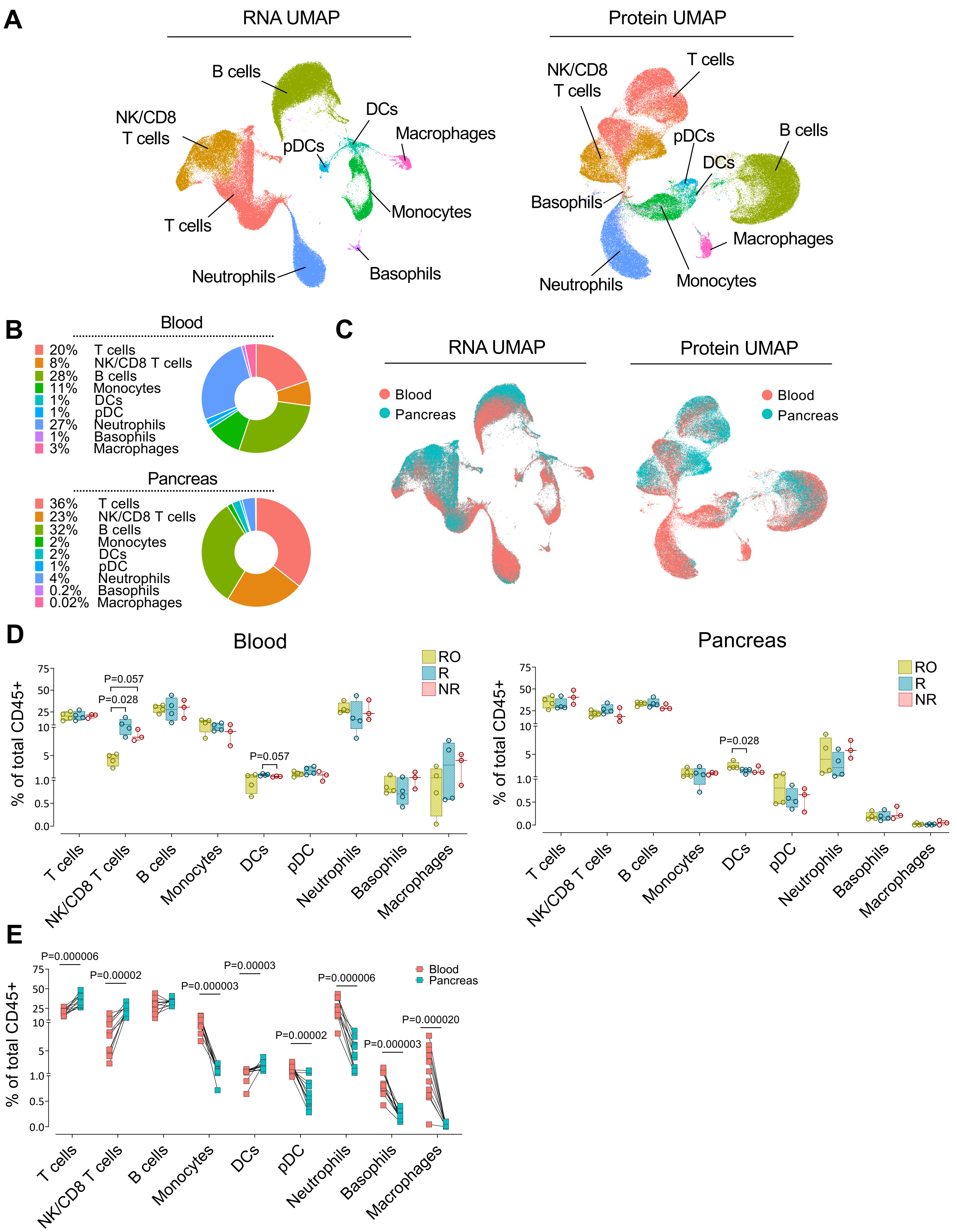

Supplemental Figure 2. Immune subset profiling in peripheral blood and pancreas of NOD mice. (**A**) UMAP (Uniform Manifold Approximation and Projection) visualizations of immune cell populations computed using RNA (left) or proteins (right). Cell types were identified using top variable genes and proteins. (**B**) Diagrams showing the proportions of the immune cell populations in peripheral blood and pancreas. (**C**) UMAP plot of immune cells colored according to their tissue of origin, with peripheral blood cells represented in red and pancreas cells in green. (**D**) Box plots showing the frequency of major immune cell types in RO (Recent-Onset; yellow), R (Responder; blue), NR (Non-Responder; red) samples, normalized on total counts of $CD45^{+}$ leukocytes. Boxes represent the median (center line) and extend from the 25th to 75th percentiles (bottom and top lines, respectively); whiskers extend from the minimum to the maximum value. (**E**) Slope chart showing the frequencies of each cell type in paired peripheral blood (red) and pancreas (green) samples for each mouse of the CITE-seq dataset. In (**D, E**), the Mann-Whitney U test was used for statistical comparison.

Supplemental Figure 3

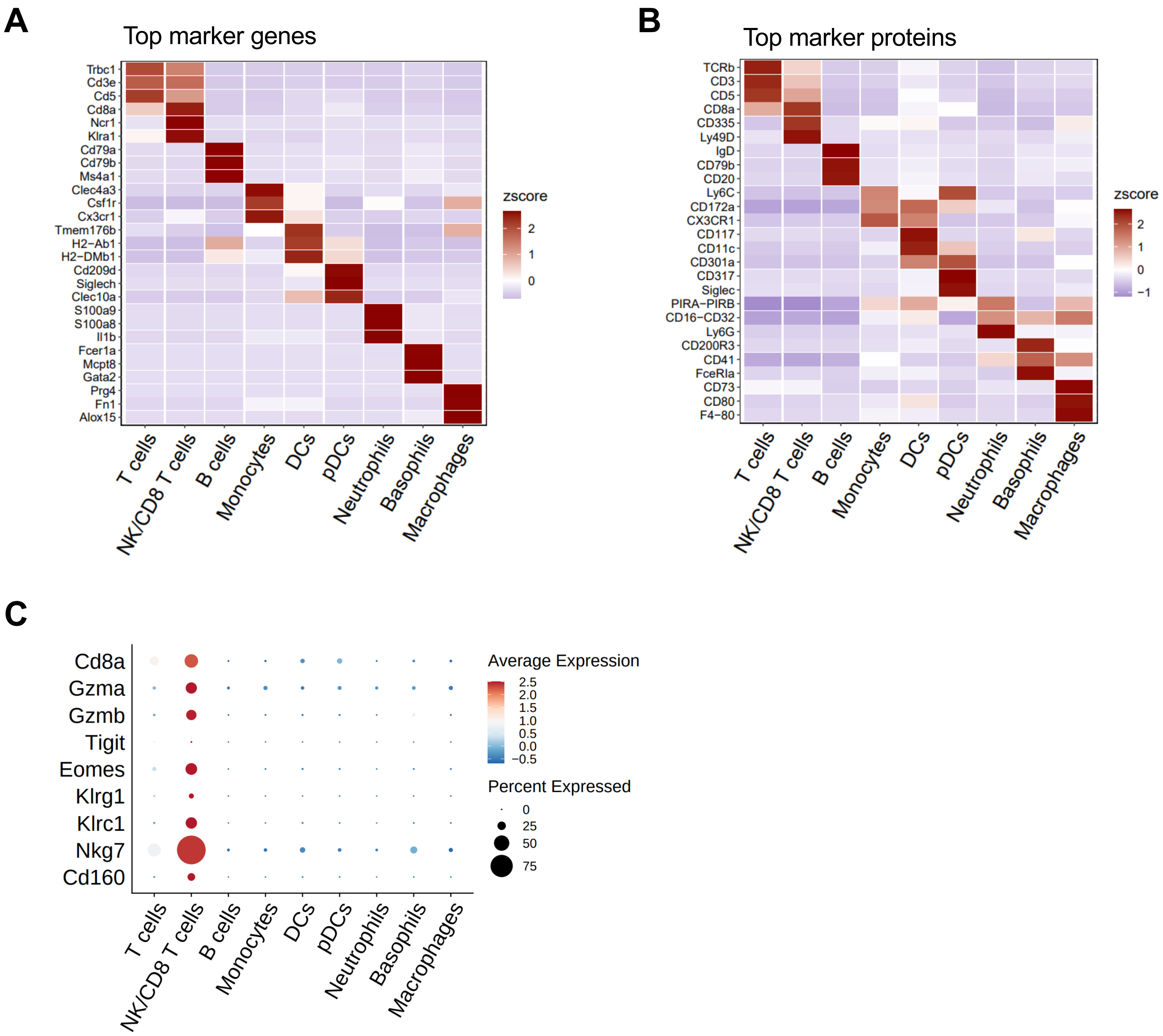

Supplemental Figure 3. Unsupervised immune subset clustering based on RNA and protein markers in mouse peripheral blood and pancreas samples. Heat map showing the expression of top marker genes (**A**) or proteins (**B**) in the main immune cell types in both peripheral blood and pancreas. (**C**) Dot plot illustrating the expression of genes associated with partial exhaustion phenotype.

Supplemental Figure 4

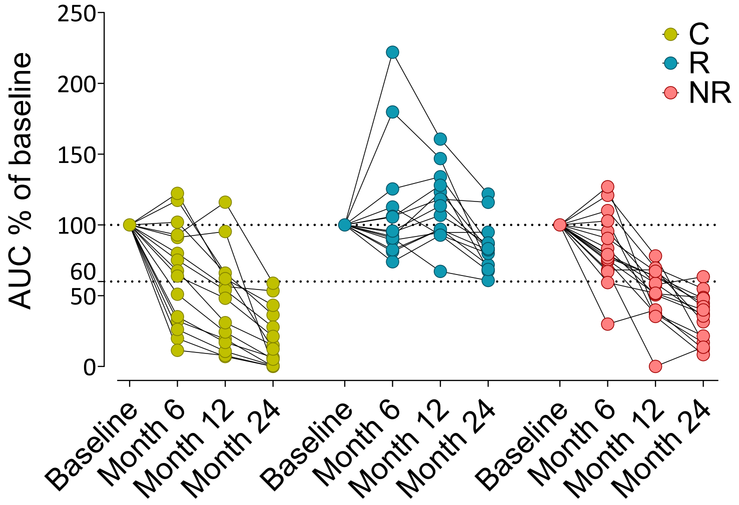

Supplemental Figure 4. C-peptide AUC percentage after teplizumab therapy in new-onset type 1 diabetes. Dot plot illustrating the percentage of C-peptide levels, measured as the mean AUC (Area Under the Curve), at baseline, and 6, 12, and 24 months after teplizumab therapy. Untreated Controls are labelled as C (yellow, n=15), Responders as R (blue, n=14), and Non-Responders as NR (red, n=16).

Supplemental Figure 5

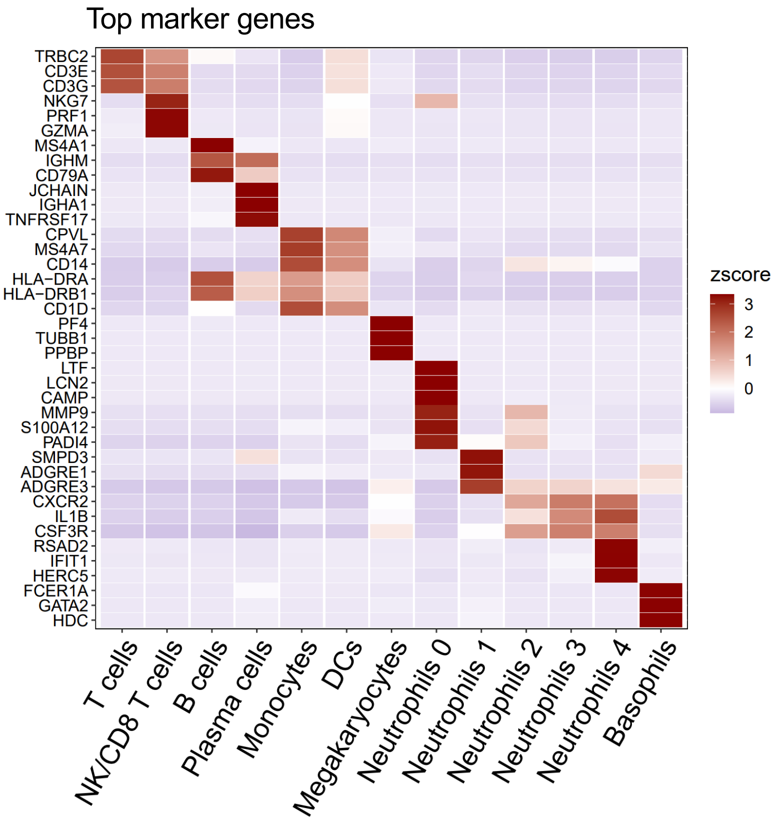

Supplemental Figure 5. Unsupervised immune subset clustering based on RNA markers in whole blood samples from stage 3 type 1 diabetic individuals. Heat map showing the expression of top marker genes in immune subsets of peripheral blood of stage 3 type 1 diabetic individuals.

Supplemental Figure 6

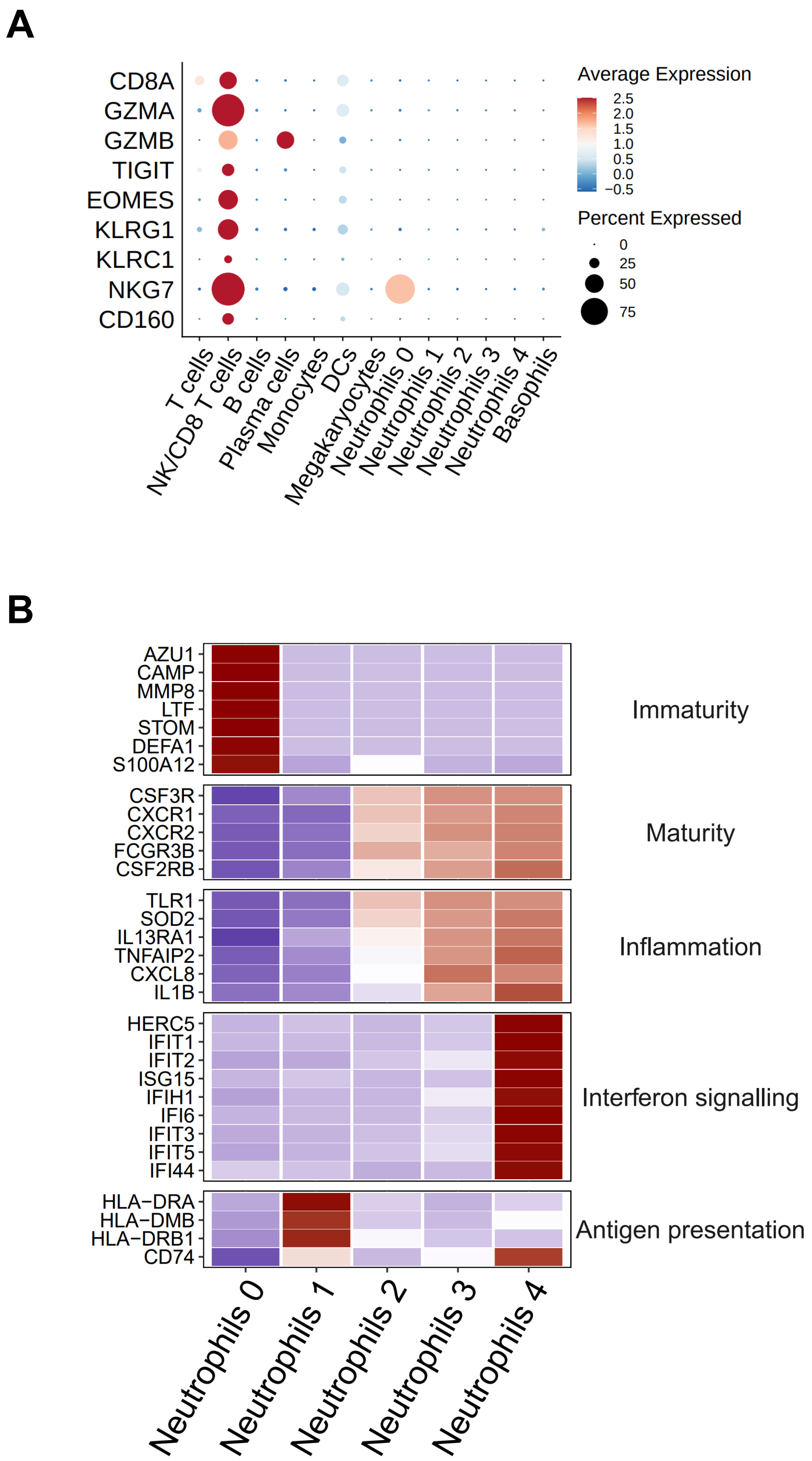

Supplemental Figure 6. Functional gene expression in immune cells from stage 3 type 1 diabetic individuals. (**A**) Dot plot illustrating the expression of genes associated with partial exhaustion phenotype. (**B**) Heat map displaying the expression of functional markers across different neutrophil subtypes.

Supplemental Figure 7

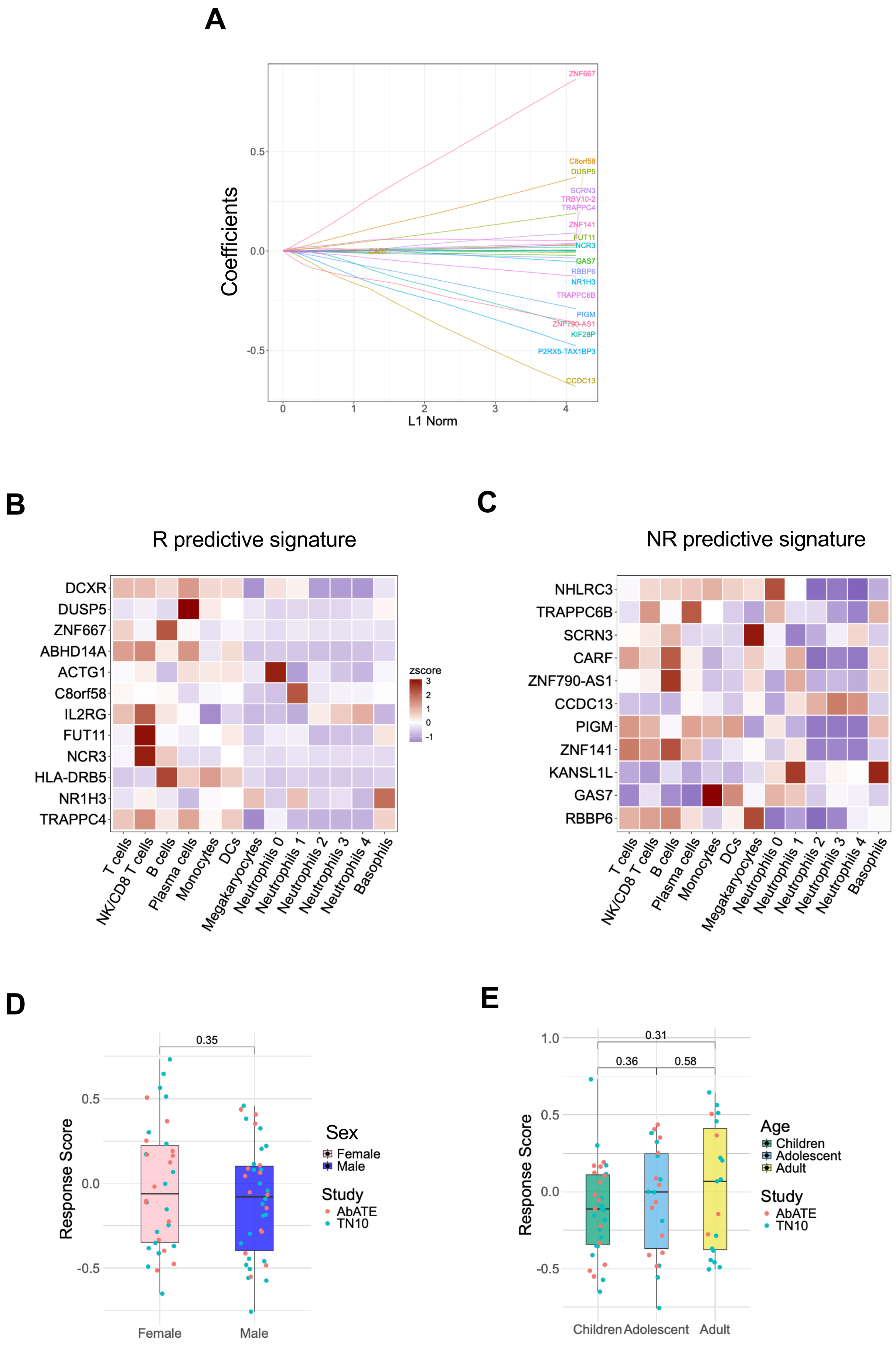

Supplemental Figure 7

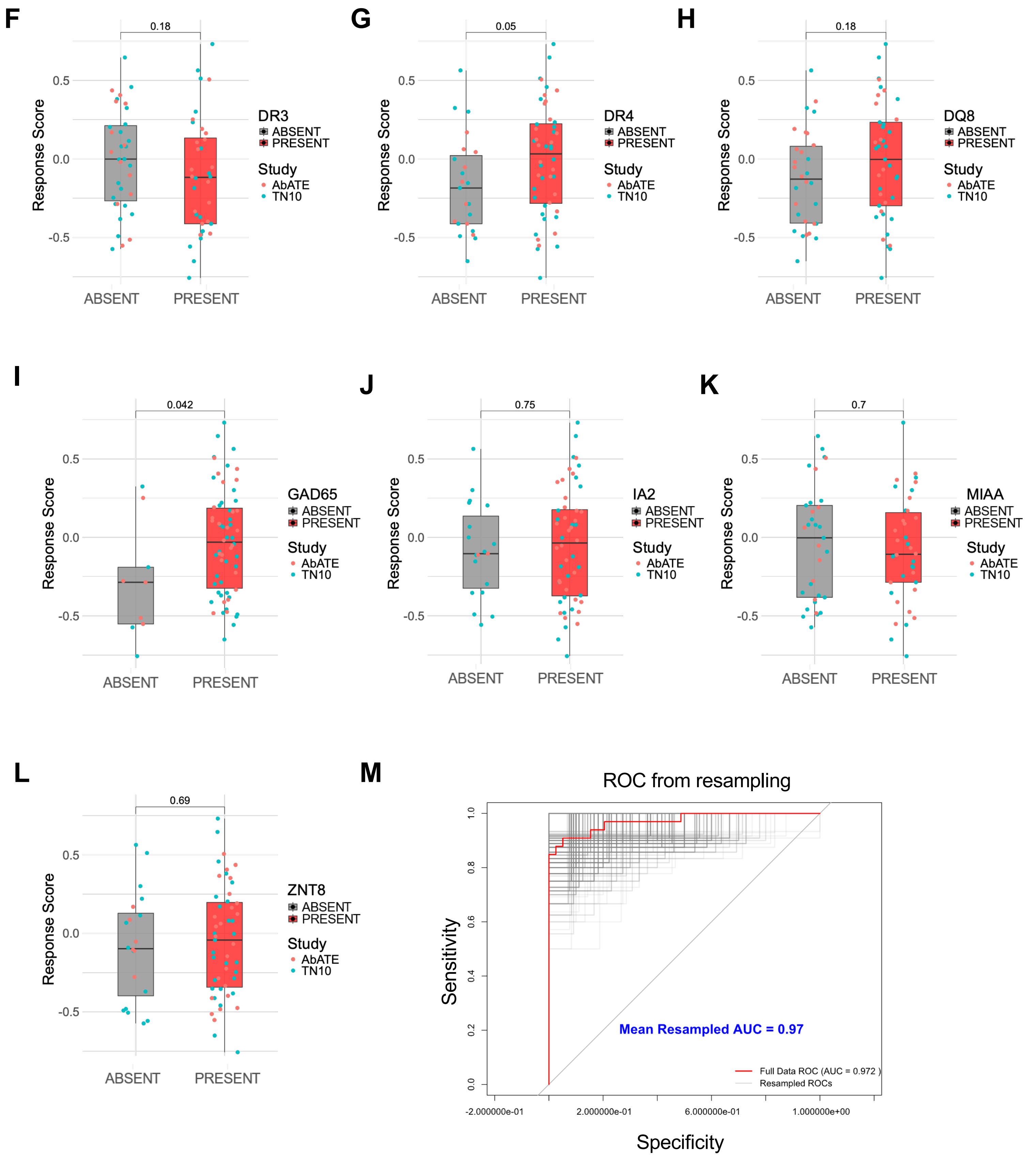

Supplemental Figure 7. Baseline predictive response score modelling, validation and covariates. (**A**) Coefficient paths showing changes in feature coefficients across different regularization strengths for the final selected features. (**B**) Heatmap presenting the expression levels of the individual genes with a positive coefficient comprising the “Response Score” at baseline in peripheral blood immune subsets, represented using the z-score. (**C**) Heatmap presenting the expression levels of the individual genes with a negative coefficient comprising the “Response Score” at baseline in peripheral blood immune subsets, represented using the z-score. **(D–L)** Box plots depicting baseline “Response Score” across various clinical and demographic subgroups: (**D**) Female (pink box, *n*=34) vs. male (blue box, *n*=38) participants; (**E**) Children (<12 years, green box, *n*=31), adolescents (12–18 years, blue box, *n*=22), and adults (≥18 years, yellow box, *n*=19); (**F**) Presence (red box, *n*=36) vs. absence (grey box, *n*=35) of the HLA-DR3 haplotype; (**G**) Presence (red box, *n*=48) vs. absence (grey box, *n*=23) of the HLA-DR4 haplotype; (**H**) Presence (red box, *n*=41) vs. absence (grey box, *n*=30) of the HLA-DQ8 haplotype; (**I–L**) Presence (red box) vs. absence (grey box) of autoantibodies against GAD65 (*n*=63/9), I-A2 (*n*=53/19), insulin (mIAA; *n*=38/34), and ZnT8 (*n*=51/21), respectively. Data points represent samples from the AbATE (red dots, *n*=30) and TN10 (cyan dots, *n*=42) studies. Boxes represent the median (center line) and extend from the 25th to 75th percentiles (bottom and top lines, respectively); whiskers extend from the minimum to the maximum value. The Wilcoxon Rank Sum test was used for statistical comparison. (**M**) ROC curve (AUC) overlay from 1,000 sampling of 20 random subjects from the full dataset to evaluate the predictive performance of the response score and ROC AUC of the full dataset (red curve).

Supplementary Table 1. List of the 190 surface protein markers (antibody-derived tag or ADT) used for CITE-sequencing on CD45^{+} leukocytes sorted from peripheral blood or pancreas specimen of recent-onset diabetic and anti-CD3 treated NOD mice.

CD301b	TCRγδ	Ly49D	CD150	CD93
CD163	IgG1-Rat-l	CD169	CD195	CD69
TCRg2	IgG1-Rat-k	CD39	Ly6G	NK1-1
CD304	CD49b	CD200R3	MERTK	CD27-A0191
CD11b-mh	CD103	IgG2b-Rat-k	CD102	CD204
CD201	TER119	CD49f	CD80	CD107a
CD11c	CD41	CD106	CD34	JAML
CD14	CD21-CD35	CD105	IgG2a-Rat-k	FceRIα
CD8a	CD19	CD3e	CD172a	TCRβ
Ly49H	TCRγ	IL33Ra	CD54	CD270
CD137	Folate	CD309-A0553	CD274	P2X7R
CD138	CD115	IgG2b-Mouse-k	CD28	CD279
CD4	CD62E	CD45	CD152	CD3
Integrin	CD23	CD71	CD55	TLR4
TCRb5	CD272	CD45-A0178	IRF4	CD122
P2RY12	CD278	Streptavidin-A0955	CD223	CD22
CD117	ENPP1	CD62L	CD16-CD32	IgM
Ly6C	CLEC4F	IgG2a-Mouse-k	CD124	CD300LG
CD335	Ly6A-Ly6E	GABRB3	CD193	Mac2
CD192	CD38	KCC2	CD197	CD326
TIGIT	CD90-A0075	CD68	CD185	CD9
KLRG1-mh	Siglec	CD300c-d	CD25	IA-IE
Tim4	MAdCAM1	F4-80	CD135	CD314
XCR1	RORγ	CX3CR1	4-1BB	CD20
ReceptorD4	CD24	ESAM	CXCR4	CD45R-B220
CD5	CD1d	CD83	CD196	CD95
CD8b	CD317	CD86	CD140a	CD127
CD79b	Pan endothelial	Streptavidin-A0952	CD370	CD26
TCRg3	CD200R	CD226	CD43	CD200
VSIG4	ERK1	CD64	Notch	CD49d
CD90	IgG2c-Rat-k	CD357	CD309-A0554	CD183
CD36	IgG-Hamster	CD49a	IgG2a	CD309
PIRA-PIRB	H2Kb	CD44-mh	CD2	CD309.1
CD73	CD15-mh	IgG1-Mouse-k	CD134	Biotin
DR3	CD206	CD62P	CD29	CD146
CD371	DLL1	CD366	CD11a	Streptavidin-A0953
IgD	CD207-mh	CD48	TCRβ8	Streptavidin-A0951
Ly6G-Ly6C	CD301a	CD63	CD31	CD278.1
