# Supplementary material for: Neutrophil-enriched gene signature correlates with teplizumab therapy resistance in different stages of type 1 diabetes
Source: J Clin Invest. 2025 Sep 30;135(23):e176403. doi: 10.1172/JCI176403 (PMC12646666; doi:10.1172/JCI176403)
Supplement: Supplemental table 2 [file jci-135-176403-s290.pdf]

Supplemental Table 2 : Marker genes of each cell type in mouse CITE-seq data

p_val	avg_log2FC	pct.1	pct.2	p_val_adj	cluster	gene
0	2.0880292	0.9	0.195	0	T cells	Cd3g
0	2.40899367	0.869	0.169	0	T cells	Cd3e
0	2.5702606	0.915	0.232	0	T cells	Trbc2
0	2.95194241	0.787	0.123	0	T cells	Trac
0	1.95478481	0.905	0.265	0	T cells	Ms4a4b
0	2.24143681	0.873	0.235	0	T cells	Cd3d
0	2.56140754	0.757	0.151	0	T cells	Lat
0	3.75914493	0.655	0.076	0	T cells	Tcf7
0	3.30376667	0.68	0.11	0	T cells	Il7r
0	2.8453508	0.677	0.109	0	T cells	Bcl11b
0	2.27195543	0.739	0.173	0	T cells	Skap1
0	4.31356731	0.634	0.069	0	T cells	Lef1
0	2.05759445	0.73	0.192	0	T cells	Lck
0	2.49136814	0.692	0.157	0	T cells	Txk
0	2.85776187	0.634	0.121	0	T cells	Cd28
0	1.82448276	0.621	0.155	0	T cells	Thy1
0	1.90478204	0.846	0.383	0	T cells	Ms4a6b
0	2.26240735	0.571	0.149	0	T cells	Cd27
0	2.63891935	0.572	0.151	0	T cells	Trbc1
0	2.53254362	0.523	0.104	0	T cells	Itk
0	2.01650759	0.746	0.333	0	T cells	Atp1b3
0	1.96322626	0.661	0.271	0	T cells	S1pr1
0	0.8876058	0.628	0.245	0	T cells	Ly6a
0	2.78718599	0.452	0.078	0	T cells	Cd247
0	1.16413607	0.745	0.371	0	T cells	Emb
0	2.53041677	0.461	0.094	0	T cells	Prkcq
0	1.46023614	0.631	0.266	0	T cells	Ccnd2
0	0.82893324	0.778	0.426	0	T cells	Fyb
0	2.16064652	0.513	0.163	0	T cells	Dgka
0	1.38025631	0.6	0.259	0	T cells	Arl4c
0	1.86226852	0.518	0.178	0	T cells	Rgs10
0	2.89117608	0.4	0.06	0	T cells	Cd5
0	1.78688655	0.562	0.231	0	T cells	Chd3
0	2.58115197	0.404	0.079	0	T cells	Gm2682
0	1.70257973	0.584	0.259	0	T cells	Srpk1
0	1.40622687	0.583	0.261	0	T cells	Gimap3
0	1.12546994	0.754	0.433	0	T cells	Ablim1
0	1.9276493	0.477	0.162	0	T cells	Inpp4b
0	2.22519289	0.443	0.13	0	T cells	Utrn
0	1.7362169	0.565	0.252	0	T cells	Vps37b
0	1.87387763	0.434	0.125	0	T cells	Fam189b
0	1.30875434	0.696	0.388	0	T cells	Pdcd4
0	1.10966399	0.668	0.361	0	T cells	Sept1
0	1.06989551	0.843	0.537	0	T cells	Ets1
0	1.05601482	0.76	0.457	0	T cells	Npc2
0	2.08083755	0.516	0.216	0	T cells	Smc4
0	5.41128126	0.313	0.013	0	T cells	Dapl1
0	4.45416931	0.322	0.023	0	T cells	Cd4

0	1.96932267	0.381	0.082	0	T cells	Cd6
0	3.59656536	0.346	0.055	0	T cells	Dusp10
0	1.97436396	0.438	0.148	0	T cells	Tecpr1
0	1.7528213	0.455	0.169	0	T cells	Grap2
0	2.25458083	0.391	0.108	0	T cells	Ramp1
0	2.51169512	0.34	0.061	0	T cells	Themis
0	0.94018563	0.682	0.403	0	T cells	H2-Q7
0	0.83363971	0.647	0.372	0	T cells	Cd2
0	1.33491143	0.618	0.345	0	T cells	Rapgef6
0	1.27539773	0.619	0.347	0	T cells	Peli1
0	2.63182763	0.348	0.078	0	T cells	Kbtbd11
0	0.80294359	0.56	0.291	0	T cells	Hcst
0	0.96286107	0.701	0.441	0	T cells	Gimap6
0	2.27548415	0.344	0.084	0	T cells	Klk8
0	1.22238399	0.461	0.203	0	T cells	Rinl
0	1.15385238	0.527	0.272	0	T cells	Gm8369
0	2.90476248	0.301	0.049	0	T cells	Sidt1
0	3.45403573	0.282	0.031	0	T cells	Nsg2
0	1.25972304	0.491	0.243	0	T cells	Saraf
0	1.60730462	0.433	0.186	0	T cells	Gramd3
0	1.62158367	0.344	0.099	0	T cells	Sh2d1a
0	1.20104644	0.601	0.357	0	T cells	Itgb7
0	1.0460179	0.546	0.303	0	T cells	Smad7
0	1.4517663	0.456	0.213	0	T cells	Tnfaip3
0	1.88389635	0.382	0.141	0	T cells	Gtf2i
0	1.6051384	0.517	0.276	0	T cells	Bcl2
0	0.7534356	0.552	0.312	0	T cells	Ccr7
0	1.16694789	0.632	0.392	0	T cells	Satb1
0	2.26392786	0.326	0.087	0	T cells	Fam78a
0	0.99799889	0.455	0.217	0	T cells	Spn
0	0.99548725	0.604	0.366	0	T cells	Gimap1
0	1.98181713	0.341	0.104	0	T cells	Ehd3
0	1.97785411	0.314	0.079	0	T cells	Zap70
0	4.56894552	0.27	0.036	0	T cells	Igfbp4
0	1.96819207	0.3	0.067	0	T cells	Gm12840
0	2.79408146	0.299	0.067	0	T cells	Kcnn4
0	1.03517542	0.477	0.248	0	T cells	Leprotl1
0	0.91044418	0.65	0.422	0	T cells	Ifi203
0	1.20191128	0.538	0.312	0	T cells	Slamf6
0	1.08213895	0.555	0.329	0	T cells	Atp11b
0	1.02190782	0.463	0.237	0	T cells	Ptpn22
0	2.08115773	0.305	0.079	0	T cells	Rnf125
0	1.01470253	0.633	0.409	0	T cells	Gm4759
0	2.14178464	0.315	0.091	0	T cells	A930005H10R
0	1.24297632	0.453	0.23	0	T cells	Gimap9
0	1.37670486	0.414	0.192	0	T cells	Ifi47
0	1.09535346	0.713	0.492	0	T cells	Crlf3
0	1.67864982	0.291	0.074	0	T cells	Itga6
0	2.05155744	0.31	0.094	0	T cells	Il27ra
0	1.31178976	0.435	0.221	0	T cells	Rnf138

0	0.7326693	0.574	0.361	0	T cells	Gimap4
0	1.19305432	0.456	0.244	0	T cells	Itpkb
0	2.46266511	0.269	0.061	0	T cells	Pik3ip1
0	0.85279439	0.455	0.248	0	T cells	Fyn
0	2.46722688	0.272	0.065	0	T cells	Rgcc
0	0.60556181	0.646	0.442	0	T cells	Rhoh
0	0.96601727	0.839	0.635	0	T cells	Gm10260
0	0.79554106	0.624	0.421	0	T cells	Il2rg
0	1.38047077	0.292	0.089	0	T cells	Cd8b1
0	1.67086094	0.35	0.149	0	T cells	Tnfrsf18
0	1.86292053	0.294	0.093	0	T cells	Abhd8
0	0.95059033	0.521	0.323	0	T cells	Evl
0	0.69969684	0.877	0.68	0	T cells	Mbnl1
0	0.39776036	0.712	0.515	0	T cells	Ptprcap
0	0.46361468	0.945	0.751	0	T cells	mt-Nd2
0	0.93364123	0.558	0.365	0	T cells	Sec11a
0	0.96252597	0.634	0.441	0	T cells	Tubb5
0	1.93116041	0.307	0.116	0	T cells	Bzw2
0	0.3835356	0.956	0.766	0	T cells	mt-Nd1
0	0.87813042	0.665	0.476	0	T cells	Ifi27l2a
0	1.23363636	0.377	0.188	0	T cells	Tes
0	1.34474298	0.36	0.172	0	T cells	Traf1
0	1.25451345	0.365	0.177	0	T cells	Rasal3
0	1.22539687	0.365	0.179	0	T cells	Pitpnc1
0	0.88253764	0.829	0.643	0	T cells	Rpl22l1
0	0.85384013	0.82	0.634	0	T cells	Npm1
0	0.74274405	0.579	0.394	0	T cells	Prdx6
0	0.5777176	0.876	0.692	0	T cells	Ltb
0	0.77559725	0.738	0.556	0	T cells	Gas5
0	1.88522241	0.274	0.093	0	T cells	Mllt3
0	0.89638111	0.53	0.35	0	T cells	Emg1
0	1.43259459	0.328	0.148	0	T cells	Rasgrp1
0	1.54098133	0.297	0.117	0	T cells	Fam102a
0	0.99662638	0.51	0.331	0	T cells	Gm8730
0	0.79277545	0.73	0.551	0	T cells	Prrc2c
0	0.61170125	0.97	0.791	0	T cells	mt-Cytb
0	0.73375096	0.746	0.568	0	T cells	Rpl13a
0	1.42784768	0.343	0.165	0	T cells	Aebp2
0	2.05007965	0.274	0.096	0	T cells	Igtp
0	1.6340517	0.283	0.105	0	T cells	Tnik
0	0.85889474	0.276	0.099	0	T cells	Cd8a
0	0.73566127	0.771	0.595	0	T cells	Rpl15
0	0.65762964	0.546	0.37	0	T cells	Acp5
0	0.83693516	0.515	0.339	0	T cells	Mif
0	1.28730574	0.304	0.128	0	T cells	Prkch
0	1.29017357	0.276	0.101	0	T cells	Sh2d2a
0	0.55113878	0.328	0.153	0	T cells	Il2rb
0	1.17782494	0.361	0.186	0	T cells	Gm37033
0	0.46542459	0.385	0.211	0	T cells	Rgs1
0	0.67599454	0.634	0.46	0	T cells	Ldha

0	0.73328486	0.585	0.412	0	T cells	Mndal
0	1.4436536	0.955	0.782	0	T cells	Tmsb10
0	0.46751717	0.935	0.763	0	T cells	mt-Nd4
0	0.99688468	0.361	0.19	0	T cells	Otulinl
0	0.83653572	0.937	0.767	0	T cells	Rpl3
0	0.95544278	0.935	0.765	0	T cells	Rps18
0	0.82308425	0.9	0.731	0	T cells	Rpl36a
0	0.73936371	0.586	0.417	0	T cells	Psme2
0	0.6653616	0.72	0.552	0	T cells	Eef1d
0	0.75035852	0.307	0.14	0	T cells	Ipcef1
0	0.65670907	0.899	0.733	0	T cells	Rpl4
0	1.02568287	0.386	0.22	0	T cells	Foxo1
0	0.66547755	0.931	0.766	0	T cells	Rps19
0	0.75731904	0.931	0.766	0	T cells	Rpl10a
0	0.64129967	0.7	0.535	0	T cells	Eif3e
0	0.81274183	0.511	0.347	0	T cells	Snhg8
0	0.75555907	0.918	0.754	0	T cells	Shisa5
0	1.34908682	0.263	0.099	0	T cells	Slco3a1
0	0.47439365	0.495	0.331	0	T cells	Pycard
0	0.911061	0.477	0.313	0	T cells	Snrpd3
0	0.6668067	0.881	0.718	0	T cells	Rpl35
0	0.59469616	0.806	0.643	0	T cells	mt-Co1
0	0.95598205	0.449	0.287	0	T cells	Zc3hav1
0	0.71977846	0.646	0.484	0	T cells	Uba52
0	1.38522243	0.309	0.147	0	T cells	Gbp7
0	1.29665894	0.27	0.109	0	T cells	Il18r1
0	0.8596983	0.457	0.297	0	T cells	Hp1bp3
0	0.67374996	0.771	0.611	0	T cells	Rpl24
0	0.57972063	0.793	0.635	0	T cells	Rpl31
0	0.82243819	0.916	0.758	0	T cells	Rpl5
0	1.13528675	0.329	0.171	0	T cells	Esyt2
0	1.39715925	0.274	0.117	0	T cells	C230085N15R
0	1.10961335	0.367	0.21	0	T cells	H2-Q4
0	0.69721098	0.582	0.425	0	T cells	Tomm20
0	0.65108738	0.71	0.554	0	T cells	Psme1
0	0.7307705	0.443	0.288	0	T cells	Sept6
0	0.70661297	0.531	0.376	0	T cells	Gm11478
0	0.73899872	0.428	0.273	0	T cells	Grap
0	0.61953718	0.535	0.381	0	T cells	Clec2d
0	1.69371486	0.254	0.1	0	T cells	Trib2
0	1.41216387	0.336	0.183	0	T cells	Nrip1
0	1.05355972	0.284	0.131	0	T cells	Hsd11b1
0	1.07583102	0.417	0.264	0	T cells	Fkbp1a
0	0.7503147	0.609	0.456	0	T cells	Gm47283
0	0.60772938	0.754	0.602	0	T cells	Ptpn18
0	0.50148207	0.803	0.651	0	T cells	S100a10
0	0.85212468	0.475	0.323	0	T cells	Clint1
0	0.73042026	0.866	0.715	0	T cells	Rps6
0	0.68516978	0.939	0.788	0	T cells	Rpl14
0	0.6723888	0.887	0.737	0	T cells	Rps17

0	0.61157484	0.645	0.495	0	T cells	Elf1
0	0.60507185	0.754	0.604	0	T cells	Eef1g
0	0.66392713	0.94	0.79	0	T cells	Rpl22
0	0.91311601	0.939	0.789	0	T cells	Rps28
0	0.57360621	0.682	0.533	0	T cells	Snrpg
0	0.71710152	0.865	0.717	0	T cells	Rpl23a
0	1.03795704	0.377	0.229	0	T cells	Snhg1
0	0.6923206	0.953	0.805	0	T cells	H2-K1
0	0.69931077	0.411	0.264	0	T cells	Rflnb
0	0.69346146	0.888	0.742	0	T cells	Rpl27a
0	0.65550283	0.812	0.666	0	T cells	Tle5
0	0.79339639	0.907	0.761	0	T cells	Rps2
0	1.17657232	0.309	0.163	0	T cells	Fam53b
0	0.76890926	0.962	0.817	0	T cells	Rps20
0	0.57078683	0.764	0.619	0	T cells	Eif3h
0	1.0235407	0.366	0.221	0	T cells	Pop5
0	0.83868237	0.426	0.281	0	T cells	Znrf2
0	0.54140325	0.938	0.793	0	T cells	Ppia
0	0.75748943	0.471	0.327	0	T cells	Ndufv3
0	0.43470858	0.69	0.546	0	T cells	Selenow
0	0.98884914	0.343	0.2	0	T cells	Sesn3
0	1.32297177	0.274	0.131	0	T cells	Gm26917
0	1.13670866	0.97	0.828	0	T cells	Rpl12
0	0.32835318	0.887	0.745	0	T cells	Hsp90ab1
0	0.62328371	0.564	0.422	0	T cells	Hspe1
0	0.77019622	0.428	0.287	0	T cells	Traf3ip3
0	0.49833548	0.642	0.501	0	T cells	Eif4a1
0	0.86795684	0.958	0.818	0	T cells	Rps24-ps3
0	0.99748453	0.912	0.772	0	T cells	Rplp0
0	0.6491895	0.901	0.761	0	T cells	Naca
0	0.6127344	0.859	0.719	0	T cells	Rpl27
0	1.00136135	0.395	0.256	0	T cells	Stat1
0	0.54277275	0.894	0.755	0	T cells	Rpl7a
0	0.63155983	0.552	0.414	0	T cells	Eif3m
0	0.4515093	0.757	0.619	0	T cells	Serbp1
0	0.84567467	0.969	0.831	0	T cells	Rpsa
0	0.74709791	0.352	0.214	0	T cells	Gimap5
0	0.57411899	0.69	0.552	0	T cells	Gm10076
0	0.67855728	0.956	0.819	0	T cells	Rack1
0	0.7382592	0.934	0.797	0	T cells	Rpl36
0	0.60245547	0.939	0.803	0	T cells	Rpl6
0	0.74774967	0.389	0.254	0	T cells	Srpk2
0	0.62813384	0.463	0.328	0	T cells	Arl6ip5
0	0.72424305	0.412	0.277	0	T cells	Ugcg
0	0.57788058	0.806	0.672	0	T cells	Limd2
0	0.45821652	0.808	0.674	0	T cells	Atp5h
0	0.88403263	0.385	0.251	0	T cells	Churc1
0	0.90319905	0.328	0.195	0	T cells	Sipa1l1
0	0.67351642	0.903	0.771	0	T cells	Rpl30
0	0.45549044	0.695	0.564	0	T cells	Ncl

0	1.17191856	0.267	0.136	0	T cells	Aak1
0	0.56076277	0.8	0.669	0	T cells	Rpl35a
0	1.00519726	0.301	0.17	0	T cells	Stim1
0	1.21484474	0.274	0.144	0	T cells	Tagap
0	0.92082923	0.383	0.253	0	T cells	Rexo2
0	0.67654339	0.937	0.807	0	T cells	Rpl38
0	0.52549997	0.611	0.481	0	T cells	Nsa2
0	0.58966077	0.548	0.419	0	T cells	Ikzf1
0	0.84890177	0.96	0.831	0	T cells	Rps24
0	0.80100549	0.943	0.814	0	T cells	Rps7
0	0.56628707	0.314	0.185	0	T cells	Tmem71
0	0.60732507	0.477	0.348	0	T cells	Phf20l1
0	0.61880655	0.352	0.223	0	T cells	Lfng
0	0.86941019	0.337	0.209	0	T cells	Plaat3
0	0.52110089	0.561	0.434	0	T cells	Park7
0	0.6078696	0.404	0.277	0	T cells	Atp1a1
0	0.52931993	0.569	0.442	0	T cells	Nsd3
0	0.94874368	0.33	0.204	0	T cells	Klhl6
0	0.51897237	0.921	0.795	0	T cells	Rpl7
0	0.58503362	0.908	0.782	0	T cells	Rpl34
0	0.50036588	0.601	0.475	0	T cells	Spcs2
0	0.63776166	0.694	0.568	0	T cells	Gm8995
0	0.84860705	0.959	0.834	0	T cells	Rps15a
0	0.84348203	0.321	0.196	0	T cells	S1pr4
0	0.72610838	0.89	0.765	0	T cells	Rps10
0	1.1914423	0.263	0.139	0	T cells	Gpr132
0	0.51638164	0.615	0.491	0	T cells	Tra2b
0	0.79083427	0.342	0.219	0	T cells	Tespa1
0	0.582232	0.546	0.424	0	T cells	Srsf7
0	0.93043009	0.916	0.794	0	T cells	Eef1b2
0	0.62986497	0.488	0.366	0	T cells	Cnot6l
0	0.65599103	0.448	0.326	0	T cells	Cbx3
0	0.46878556	0.63	0.508	0	T cells	Klf13
0	0.57432454	0.47	0.348	0	T cells	Rps27rt
0	0.55732588	0.549	0.428	0	T cells	Stk4
0	0.65238987	0.968	0.848	0	T cells	Rps4x
0	0.60823888	0.473	0.353	0	T cells	S100a13
0	0.55599991	0.48	0.361	0	T cells	1810026B05R
0	0.69174602	0.394	0.275	0	T cells	Slc25a4
0	0.48013337	0.779	0.66	0	T cells	Cox7a2l
0	0.71783202	0.369	0.25	0	T cells	Ppp1r14b
0	0.67364129	0.934	0.816	0	T cells	Rpl21
0	0.80756808	0.31	0.192	0	T cells	Gramd1a
0	0.43460478	0.871	0.753	0	T cells	Eef2
0	0.6886327	0.941	0.823	0	T cells	Rps26
0	0.41899323	0.791	0.673	0	T cells	Psmb8
0	0.98811188	0.284	0.167	0	T cells	Snhg12
0	0.79733029	0.344	0.227	0	T cells	Npm3
0	0.5361222	0.66	0.544	0	T cells	Tpr
0	0.78178801	0.97	0.854	0	T cells	Rpl32

0	0.65173801	0.962	0.846	0	T cells	Rps5
0	0.71028143	0.384	0.268	0	T cells	Idnk
0	0.84700453	0.279	0.164	0	T cells	Pfkp
0	0.74944825	0.382	0.267	0	T cells	Cct6a
0	0.40587793	0.688	0.573	0	T cells	Cnbp
0	0.83141047	0.942	0.827	0	T cells	Rpl18
0	0.73024706	0.39	0.275	0	T cells	Bola2
0	0.88404621	0.29	0.175	0	T cells	Arl5c
0	0.63078892	0.413	0.299	0	T cells	Dnajc3
0	0.6642692	0.439	0.325	0	T cells	Pnn
0	0.48690958	0.551	0.438	0	T cells	Atp5mpl
0	0.81716576	0.263	0.15	0	T cells	D16Ertd472e
0	0.77748015	0.956	0.843	0	T cells	Rps23
0	0.56152252	0.593	0.48	0	T cells	Ndfip1
0	0.63445128	0.272	0.16	0	T cells	Znrf1
0	0.45507667	0.611	0.499	0	T cells	Snrpe
0	0.67922891	0.431	0.319	0	T cells	Nt5c
0	0.99213557	0.303	0.191	0	T cells	Cblb
0	0.9940872	0.266	0.155	0	T cells	Kif1b
0	0.81304453	0.277	0.166	0	T cells	Il21r
0	0.84801323	0.33	0.219	0	T cells	Kcnq1ot1
0	0.94636734	0.283	0.173	0	T cells	B4galt1
0	0.44357671	0.824	0.714	0	T cells	Uqcrh
0	0.80689637	0.298	0.188	0	T cells	Cdipt
0	0.34003593	0.797	0.688	0	T cells	Hspa8
0	0.90288195	0.261	0.152	0	T cells	Gimap8
0	0.86175447	0.312	0.203	0	T cells	Prrc2b
0	0.71794127	0.378	0.269	0	T cells	Akr1b3
0	0.70942911	0.363	0.254	0	T cells	Tnrc6c
0	0.7034257	0.963	0.855	0	T cells	Rps15
0	0.68760031	0.968	0.86	0	T cells	Rpl13
0	0.71259474	0.316	0.21	0	T cells	4932438A13R
0	0.87694793	0.285	0.179	0	T cells	Dgkz
0	0.38554564	0.776	0.67	0	T cells	Srrm2
0	0.75538478	0.311	0.206	0	T cells	Rps10-ps1
0	0.73566124	0.339	0.234	0	T cells	Nap1l4
0	0.49438494	0.954	0.849	0	T cells	Rpl26
0	0.96276815	0.26	0.156	0	T cells	Ldb1
0	0.68045923	0.956	0.852	0	T cells	Rpl11
0	0.80908543	0.968	0.865	0	T cells	Rps21
0	0.38689847	0.691	0.588	0	T cells	Hnrnpa0
0	0.6044353	0.254	0.153	0	T cells	C230096K16R
0	0.44596348	0.829	0.728	0	T cells	Hnrnpf
0	0.7991563	0.971	0.872	0	T cells	Rpl19
0	0.55880828	0.952	0.855	0	T cells	Rpl37a
0	0.58336785	0.948	0.852	0	T cells	Rpl28
0	0.69101608	0.954	0.858	0	T cells	Rps13
0	0.35877727	0.81	0.715	0	T cells	Hnrnpa2b1
0	0.63734064	0.965	0.87	0	T cells	Rps11
0	0.91318294	0.96	0.866	0	T cells	Rplp2

0	0.6840844	0.972	0.88	0	T cells	Rps8
0	0.36469139	0.835	0.744	0	T cells	Pabpc1
0	0.30622115	0.933	0.845	0	T cells	Rpl10
0	0.73058262	0.958	0.871	0	T cells	Rpl39
0	1.30169343	0.97	0.889	0	T cells	Rplp1
0	0.43614241	0.913	0.833	0	T cells	Rps25
0	0.70891167	0.968	0.894	0	T cells	Rpl8
0	0.64729214	0.974	0.901	0	T cells	Eef1a1
0	0.56129424	0.939	0.87	0	T cells	Rpl37
0	0.77887932	0.968	0.901	0	T cells	Rps3
0	0.74827289	0.973	0.908	0	T cells	Rpl23
0	0.5375455	0.958	0.901	0	T cells	Rpl41
0	0.63989965	0.976	0.92	0	T cells	Rps3a1
0	0.74606358	0.975	0.919	0	T cells	Rps29
0	0.69199825	0.959	0.903	0	T cells	Rpl17
0	0.54751423	0.96	0.907	0	T cells	Rps12
0	0.71915601	0.949	0.896	0	T cells	mt-Rnr1
0	0.57073951	0.969	0.921	0	T cells	Rpl18a
0	0.73482541	0.98	0.937	0	T cells	Rps14
0	0.55763761	0.968	0.928	0	T cells	Rpl9
0	0.96027554	0.971	0.932	0	T cells	Rps16
0	0.63312803	0.97	0.937	0	T cells	Rps27a
0	0.64733989	0.97	0.938	0	T cells	Rps27
0	0.77970468	0.987	0.975	0	T cells	Tpt1
0	0.33367342	0.967	0.957	0	T cells	B2m
5.355E-308	0.44830454	0.59	0.478	1.328E-303	T cells	Abracl
3.375E-307	0.77430951	0.286	0.186	8.367E-303	T cells	Setx
1.133E-305	0.47623281	0.58	0.47	2.809E-301	T cells	Ran
8.838E-305	0.70100018	0.371	0.263	2.191E-300	T cells	Cited2
2.018E-304	0.53172757	0.481	0.373	5.003E-300	T cells	Nme1
1.547E-303	0.53425964	0.549	0.428	3.836E-299	T cells	Ifngr1
4.117E-302	0.51531856	0.501	0.389	1.021E-297	T cells	Nop10
3.804E-298	0.35257374	0.729	0.629	9.432E-294	T cells	Psmb1
1.225E-296	0.44731867	0.664	0.543	3.037E-292	T cells	mt-Nd5
1.672E-296	0.84698871	0.278	0.184	4.145E-292	T cells	Mrpl58
7.301E-296	0.64866267	0.342	0.232	1.81E-291	T cells	Kif21b
7.343E-293	0.82102522	0.27	0.176	1.821E-288	T cells	Ski
9.988E-293	0.80020017	0.284	0.189	2.477E-288	T cells	Tpt1-ps3
2.807E-291	0.92871676	0.259	0.168	6.961E-287	T cells	Tet3
3.464E-286	0.4343129	0.638	0.529	8.589E-282	T cells	Arhgef1
1.443E-284	0.3698653	0.676	0.568	3.578E-280	T cells	Atp5j2
2.098E-284	0.37416572	0.362	0.246	5.201E-280	T cells	Lcp2
4.764E-283	0.56178907	0.442	0.335	1.181E-278	T cells	Sh3kbp1
1.847E-281	0.74670363	0.274	0.179	4.579E-277	T cells	Selenop
4.382E-279	0.60420098	0.392	0.285	1.087E-274	T cells	Vgll4
3.568E-278	0.41680148	0.579	0.463	8.846E-274	T cells	Cox7b
1.209E-275	0.71201248	0.33	0.231	2.998E-271	T cells	Taf1d
1.18E-268	0.49410442	0.46	0.356	2.927E-264	T cells	Scp2
1.236E-268	0.5014132	0.48	0.377	3.066E-264	T cells	Lsm4
2.99E-267	0.29249961	0.979	0.981	7.415E-263	T cells	mt-Rnr2

3.605E-267	0.53634345	0.496	0.396	8.938E-263	T cells	Xrn2
4.295E-265	0.67320483	0.322	0.226	1.065E-260	T cells	Tbrg1
1.192E-264	0.48807308	0.536	0.429	2.955E-260	T cells	Arhgap15
1.644E-262	0.50174633	0.344	0.232	4.076E-258	T cells	Stat4
1.191E-258	0.72334218	0.275	0.185	2.954E-254	T cells	Egln1
1.364E-258	0.38027765	0.335	0.226	3.381E-254	T cells	Il6ra
1.004E-257	0.74321727	0.293	0.204	2.488E-253	T cells	Rpl10a-ps1
7.172E-257	0.64369606	0.316	0.222	1.778E-252	T cells	Ssbp4
7.196E-256	0.68308936	0.344	0.248	1.784E-251	T cells	Adcy7
8.538E-253	0.30645046	0.725	0.626	2.117E-248	T cells	Tma7
1.819E-252	0.46977858	0.496	0.394	4.509E-248	T cells	Tbca
4.271E-248	0.67060845	0.293	0.2	1.059E-243	T cells	Rbl2
1.256E-246	0.68378001	0.324	0.233	3.115E-242	T cells	Trp53
1.503E-246	0.46928715	0.357	0.252	3.727E-242	T cells	Ifi27
7.549E-245	0.36434211	0.654	0.549	1.872E-240	T cells	Hnrnpa3
2.143E-243	0.54809612	0.395	0.3	5.313E-239	T cells	Sugt1
3.769E-243	0.34395842	0.698	0.599	9.345E-239	T cells	Atp5b
7.062E-242	0.78278039	0.257	0.173	1.751E-237	T cells	Rpl29
2.789E-241	0.55592968	0.408	0.312	6.916E-237	T cells	Jun
3.094E-241	0.5988303	0.416	0.32	7.672E-237	T cells	Birc6
4.413E-241	0.60965447	0.41	0.314	1.094E-236	T cells	Sptbn1
1.773E-239	0.57679614	0.487	0.39	4.396E-235	T cells	Sdf4
1.563E-237	0.2568344	0.82	0.739	3.874E-233	T cells	Btf3
2.534E-237	0.31238593	0.446	0.338	6.283E-233	T cells	Anxa6
4.516E-237	0.52158559	0.467	0.369	1.12E-232	T cells	Fubp1
1.839E-236	0.6158154	0.315	0.223	4.559E-232	T cells	Sept9
6.02E-234	0.51020355	0.454	0.36	1.493E-229	T cells	Atp5g3
1.163E-233	0.5518435	0.329	0.235	2.883E-229	T cells	Tpst2
8.91E-233	0.67783664	0.281	0.195	2.209E-228	T cells	Skp1a
1.369E-232	0.44271225	0.514	0.415	3.394E-228	T cells	Rpl36al
6.066E-231	0.42053798	0.407	0.301	1.504E-226	T cells	Pik3cd
1.946E-229	0.45173117	0.49	0.394	4.824E-225	T cells	Eif3i
4.545E-229	0.70599691	0.292	0.206	1.127E-224	T cells	Chordc1
1.65E-228	0.7471392	0.281	0.196	4.09E-224	T cells	Ubac2
1.578E-227	0.34507724	0.65	0.549	3.913E-223	T cells	Cox7c
3.239E-227	0.50725763	0.44	0.341	8.032E-223	T cells	Zfp292
5.998E-227	0.45861376	0.397	0.296	1.487E-222	T cells	Psip1
4.382E-226	0.73064663	0.264	0.183	1.087E-221	T cells	Ccdc85b
1.371E-224	0.69157078	0.306	0.219	3.399E-220	T cells	Tap1
2.072E-224	0.38863546	0.553	0.45	5.138E-220	T cells	Hnrnpa1
3.732E-224	0.49016526	0.409	0.312	9.254E-220	T cells	Fkbp3
4.1E-224	0.75932095	0.251	0.17	1.017E-219	T cells	Exosc8
6.039E-224	0.26022966	0.786	0.726	1.497E-219	T cells	Pnrc1
2.464E-222	0.454219	0.556	0.463	6.111E-218	T cells	Snrnp70
1.772E-221	0.57248184	0.364	0.276	4.393E-217	T cells	Snrpf
4.623E-221	0.38213008	0.579	0.482	1.146E-216	T cells	Dnaja1
3.372E-220	0.38747855	0.565	0.468	8.36E-216	T cells	Nop53
1.494E-218	0.76365993	0.268	0.189	3.704E-214	T cells	Itm2c
5.318E-218	0.35550858	0.478	0.36	1.319E-213	T cells	Tbc1d10c
2.006E-217	0.62944909	0.317	0.231	4.974E-213	T cells	Gm15500

6.33E-211	0.46022664	0.35	0.255	1.57E-206	T cells	Runx3
3.92E-209	0.53419343	0.41	0.321	9.721E-205	T cells	Ddx39b
1.1E-207	0.60385423	0.284	0.199	2.727E-203	T cells	Prkacb
1.123E-206	0.42032131	0.506	0.413	2.784E-202	T cells	Cct2
1.169E-206	0.28414538	0.688	0.58	2.898E-202	T cells	Ndufa4
1.126E-202	0.6888108	0.269	0.191	2.793E-198	T cells	C1qbp
7.349E-202	0.64696205	0.298	0.217	1.822E-197	T cells	Thoc7
3.593E-200	0.54668973	0.388	0.301	8.909E-196	T cells	Bod1l
3.974E-200	0.30952444	0.715	0.626	9.854E-196	T cells	Rbm3
5.077E-200	0.57373256	0.33	0.244	1.259E-195	T cells	Numa1
1.002E-198	0.71793715	0.272	0.194	2.485E-194	T cells	Efr3a
5.77E-198	0.50663936	0.405	0.315	1.431E-193	T cells	Tgfbr2
5.893E-198	0.51527589	0.383	0.297	1.461E-193	T cells	Cops9
2.172E-197	0.41678089	0.491	0.4	5.386E-193	T cells	Uqcr10
3.92E-197	0.27523587	0.58	0.462	9.72E-193	T cells	4930523C07R
1.37E-195	0.45331933	0.468	0.374	3.397E-191	T cells	Kmt2a
6.11E-194	0.55730398	0.305	0.221	1.515E-189	T cells	Sh3gl1
4.455E-191	0.33891675	0.611	0.518	1.105E-186	T cells	Cox6c
3.591E-189	0.43017636	0.436	0.349	8.905E-185	T cells	Micos13
1.455E-186	0.29850283	0.534	0.415	3.609E-182	T cells	Cd69
1.118E-185	0.30986272	0.652	0.552	2.772E-181	T cells	Eif3k
2.148E-185	0.62874765	0.306	0.228	5.325E-181	T cells	Lsm8
7.946E-185	0.47042803	0.434	0.351	1.97E-180	T cells	Cct7
1.923E-184	0.61526264	0.311	0.23	4.768E-180	T cells	Tle4
2.863E-184	0.41922426	0.257	0.178	7.1E-180	T cells	Clip1
2.493E-182	0.26498292	0.413	0.307	6.182E-178	T cells	Lgals1
7.022E-182	0.51849884	0.426	0.346	1.741E-177	T cells	Srsf2
8.659E-181	0.55373121	0.375	0.295	2.147E-176	T cells	Emc10
1.026E-180	0.49199394	0.423	0.341	2.545E-176	T cells	Pura
1.126E-178	0.64387712	0.288	0.214	2.793E-174	T cells	Fbl
4.446E-178	0.41993745	0.443	0.353	1.102E-173	T cells	Ankrd12
1.294E-177	0.46701243	0.434	0.35	3.209E-173	T cells	Top2b
1.3E-177	0.38499471	0.514	0.425	3.224E-173	T cells	Atp5o.1
4.388E-177	0.32929032	0.587	0.498	1.088E-172	T cells	Ndufa2
6.651E-177	0.44718745	0.36	0.274	1.649E-172	T cells	Aprt
1.593E-176	0.49992728	0.363	0.276	3.951E-172	T cells	Ccdc88c
1.784E-176	0.45350108	0.392	0.304	4.424E-172	T cells	Cd164
1.633E-173	0.58610103	0.322	0.246	4.048E-169	T cells	Tap2
4.505E-170	0.38456906	0.436	0.349	1.117E-165	T cells	Gm28438
1.384E-168	0.28526687	0.675	0.59	3.432E-164	T cells	Ndufa13
2.965E-168	0.57236553	0.328	0.253	7.352E-164	T cells	Mrpl24
4.531E-168	0.52298891	0.338	0.26	1.123E-163	T cells	Ddx21
4.862E-168	0.40135722	0.509	0.425	1.206E-163	T cells	Ddx24
1.394E-166	0.34207524	0.59	0.503	3.456E-162	T cells	Anp32b
3.196E-166	0.32441442	0.381	0.293	7.924E-162	T cells	Slc9a3r1
3.681E-165	0.57038334	0.281	0.205	9.126E-161	T cells	Rictor
5.437E-165	1.18229905	0.174	0.254	1.348E-160	T cells	Hbb-bs
3.162E-163	0.29727123	0.635	0.542	7.839E-159	T cells	Atp5a1
1.971E-162	0.60578887	0.303	0.228	4.886E-158	T cells	Plec
3.131E-162	0.44819524	0.271	0.194	7.763E-158	T cells	Arl2bp

1.03E-161	0.30770337	0.593	0.508	2.554E-157	T cells	Set
2.203E-160	0.58531583	0.298	0.226	5.463E-156	T cells	Rsl1d1
5.19E-160	0.6380313	0.284	0.213	1.287E-155	T cells	Tpp2
5.599E-160	0.31259244	0.578	0.493	1.388E-155	T cells	Tomm7
1.141E-159	0.44207551	0.4	0.322	2.829E-155	T cells	Bcas2
5.778E-159	0.46382417	0.362	0.284	1.433E-154	T cells	Pole4
5.954E-159	0.39738335	0.498	0.416	1.476E-154	T cells	Smchd1
1.854E-157	0.42961967	0.43	0.351	4.597E-153	T cells	Mrpl30
1.153E-156	0.29874341	0.61	0.521	2.86E-152	T cells	Sp100
5.868E-156	0.51917135	0.339	0.265	1.455E-151	T cells	Tcof1
1.178E-155	0.37245132	0.502	0.42	2.922E-151	T cells	Srp9
6.299E-154	0.51229467	0.366	0.293	1.562E-149	T cells	Imp3
2.102E-153	0.56535212	0.29	0.216	5.212E-149	T cells	Fryl
1.965E-149	0.54001901	0.319	0.248	4.872E-145	T cells	Ndufa10
1.985E-149	0.48156314	0.36	0.286	4.923E-145	T cells	Cct3
1.139E-145	0.28724032	0.452	0.368	2.823E-141	T cells	Ppp1r15a
9.588E-143	0.41346318	0.434	0.36	2.377E-138	T cells	Rtf1
1.765E-141	0.61862941	0.286	0.219	4.376E-137	T cells	Asxl2
2.163E-141	0.62547852	0.332	0.267	5.364E-137	T cells	Tspan32
1.661E-140	0.38982255	0.58	0.5	4.118E-136	T cells	CT010467.1
1.684E-140	0.53938462	0.288	0.221	4.176E-136	T cells	Adh5
3.361E-140	0.39648498	0.449	0.375	8.333E-136	T cells	Nucks1
1.132E-138	0.48852282	0.348	0.28	2.808E-134	T cells	Banf1
7.707E-136	0.32039121	0.527	0.45	1.911E-131	T cells	Psmb9
1.091E-135	0.59379839	0.278	0.214	2.704E-131	T cells	Maz
1.648E-135	0.31487318	0.551	0.474	4.087E-131	T cells	Csnk1a1
2.065E-135	0.45815073	0.345	0.273	5.119E-131	T cells	Wdr33
2.988E-135	0.31053395	0.572	0.489	7.408E-131	T cells	Dad1
1.639E-134	0.26419101	0.796	0.727	4.064E-130	T cells	Zfp36l2
1.914E-134	0.40331755	0.388	0.314	4.746E-130	T cells	Mat2b
1.136E-132	0.26672129	0.664	0.586	2.817E-128	T cells	Arhgap45
3.983E-132	0.34820666	0.452	0.376	9.876E-128	T cells	Pebp1
5.741E-132	0.41676094	0.294	0.218	1.424E-127	T cells	Arap2
5.424E-131	0.58265121	0.254	0.191	1.345E-126	T cells	Synj2bp
1.161E-130	0.57554587	0.265	0.202	2.878E-126	T cells	Mrps26
3.684E-129	0.38235238	0.401	0.329	9.135E-125	T cells	Eif4ebp2
5.326E-129	0.30706461	0.601	0.519	1.321E-124	T cells	Macf1
2.177E-128	0.50598177	0.354	0.286	5.399E-124	T cells	Zfp644
1.234E-127	0.51566562	0.334	0.268	3.061E-123	T cells	Huwe1
2.322E-127	0.53405343	0.299	0.234	5.757E-123	T cells	Cflar
6.476E-127	0.40246977	0.448	0.374	1.606E-122	T cells	Ttc14
2.063E-126	0.2677181	0.628	0.549	5.115E-122	T cells	Atp5j
4.164E-126	0.58165956	0.257	0.196	1.033E-121	T cells	Snrpa1
6.165E-126	0.33742737	0.509	0.438	1.529E-121	T cells	Sec62
9.334E-126	0.42287833	0.395	0.327	2.314E-121	T cells	Cdc37
1.445E-125	0.32672446	0.491	0.413	3.584E-121	T cells	Tmed9
2.757E-125	0.48069521	0.327	0.263	6.836E-121	T cells	Timm23
2.948E-124	0.2618012	0.661	0.567	7.31E-120	T cells	Hint1
3.666E-122	0.41877213	0.362	0.295	9.09E-118	T cells	Tomm5
1.12E-121	0.35182463	0.473	0.402	2.776E-117	T cells	Acin1

5.544E-121	0.36884496	0.409	0.338	1.375E-116	T cells	Atp5g1
5.596E-121	0.312528	0.364	0.285	1.387E-116	T cells	P2ry10
1.099E-120	0.33287969	0.479	0.411	2.724E-116	T cells	Raly
7.438E-120	0.39512195	0.271	0.203	1.844E-115	T cells	Nabp1
8.072E-120	0.25174928	0.659	0.585	2.002E-115	T cells	Tmem50a
8.928E-117	0.25887481	0.594	0.514	2.214E-112	T cells	Uqcrq
2.018E-116	0.47276788	0.279	0.218	5.004E-112	T cells	Uqcc3
2.061E-116	0.53514642	0.294	0.231	5.111E-112	T cells	Dhx36
2.814E-116	0.38267991	0.413	0.341	6.977E-112	T cells	Map4k4
5.728E-116	0.28815995	0.5	0.427	1.42E-111	T cells	Nol7
7.813E-116	0.40643432	0.331	0.266	1.937E-111	T cells	Ubn1
1.102E-115	0.50066384	0.298	0.235	2.733E-111	T cells	Snrnp48
1.779E-115	0.29576077	0.553	0.484	4.411E-111	T cells	Psma2
9.257E-114	0.38550713	0.332	0.265	2.295E-109	T cells	Gltp
9.555E-114	0.48998356	0.289	0.229	2.369E-109	T cells	Sars
2.875E-113	0.36109739	0.428	0.36	7.128E-109	T cells	G3bp1
7.322E-113	0.28070984	0.52	0.451	1.816E-108	T cells	Mbd2
1.686E-112	0.44656798	0.365	0.302	4.18E-108	T cells	Thrap3
1.568E-111	0.43730643	0.285	0.225	3.887E-107	T cells	Dnajc15
3.246E-111	0.49867007	0.273	0.213	8.049E-107	T cells	Scamp3
4.663E-110	0.32104789	0.458	0.39	1.156E-105	T cells	Ndufb5
3.026E-109	0.41841338	0.378	0.313	7.502E-105	T cells	Celf1
1.722E-108	0.43140071	0.308	0.245	4.27E-104	T cells	Tnrc6a
2.28E-108	0.2636418	0.615	0.545	5.652E-104	T cells	Rbm25
3.474E-108	0.43410195	0.302	0.239	8.615E-104	T cells	Plgrkt
9.105E-107	0.30404157	0.469	0.401	2.258E-102	T cells	Cct5
1.271E-106	0.50897294	0.261	0.204	3.151E-102	T cells	Pa2g4
1.131E-105	0.28442144	0.654	0.594	2.803E-101	T cells	Arl6ip1
6.053E-105	0.45356101	0.302	0.242	1.501E-100	T cells	Commd8
6.109E-105	0.48272199	0.262	0.205	1.515E-100	T cells	Glrx5
1.057E-104	0.32994396	0.438	0.371	2.62E-100	T cells	Cct4
1.08E-104	0.39127363	0.428	0.367	2.678E-100	T cells	Psmb10
1.286E-104	0.33655165	0.473	0.41	3.19E-100	T cells	Ube2s
3.243E-104	0.39366643	0.36	0.299	8.04E-100	T cells	Ranbp1
4.29E-104	0.32114863	0.439	0.374	1.064E-99	T cells	Timm13
1.474E-103	0.36255571	0.384	0.32	3.655E-99	T cells	Rnf187
1.597E-103	0.4051094	0.35	0.289	3.96E-99	T cells	Hspd1
3.8E-103	0.41377307	0.349	0.289	9.421E-99	T cells	Smc1a
3.457E-102	0.42921457	0.31	0.25	8.5714E-98	T cells	Fkbp4
3.604E-102	0.50852214	0.277	0.222	8.9351E-98	T cells	Nudt21
8.664E-102	0.49289649	0.338	0.278	2.1483E-97	T cells	Esyt1
1.389E-101	0.39852996	0.345	0.287	3.4439E-97	T cells	Hdgf
2.23E-101	0.36300428	0.393	0.329	5.5293E-97	T cells	Spcs1
2.791E-101	0.34244411	0.498	0.436	6.9215E-97	T cells	Atrx
6.434E-101	0.40289735	0.335	0.273	1.5954E-96	T cells	Zdhhc20
7.772E-101	0.48677544	0.259	0.203	1.9271E-96	T cells	Epc1
1.387E-100	0.54576163	0.269	0.215	3.4394E-96	T cells	Uhrf2
3.123E-99	0.43871056	0.28	0.222	7.7435E-95	T cells	Ppp4r2
1.1559E-98	0.32483333	0.428	0.363	2.8662E-94	T cells	Cct8
4.0947E-98	0.52190734	0.26	0.205	1.0153E-93	T cells	Tlk1

5.6159E-98	0.32287777	0.451	0.39	1.3925E-93	T cells	Hnrnpab
6.2705E-98	0.38265917	0.347	0.289	1.5548E-93	T cells	Mrpl18
3.613E-97	0.33869591	0.375	0.313	8.9585E-93	T cells	Wapl
9.3034E-97	0.44474595	0.295	0.24	2.3068E-92	T cells	Ndufa5
2.0425E-96	0.48966521	0.289	0.234	5.0643E-92	T cells	Eny2
2.0541E-96	0.56565931	0.258	0.205	5.0931E-92	T cells	Ptbp1
3.5991E-96	0.44251706	0.266	0.21	8.9239E-92	T cells	Usp3
7.8905E-96	0.38583909	0.337	0.28	1.9564E-91	T cells	Rwdd1
2.3999E-94	0.43270976	0.324	0.269	5.9507E-90	T cells	Ndufs4
3.2485E-94	0.27413525	0.512	0.45	8.0546E-90	T cells	Sept7
5.7879E-94	0.41827457	0.342	0.285	1.4351E-89	T cells	Aimp1
1.251E-93	0.38817605	0.334	0.273	3.1018E-89	T cells	Cyld
8.96E-93	0.31745331	0.458	0.398	2.2216E-88	T cells	Bzw1
9.3262E-93	0.30826677	0.427	0.36	2.3124E-88	T cells	Ndufa1
9.2288E-92	0.37164628	0.298	0.239	2.2883E-87	T cells	Itpr2
4.5607E-90	0.2678744	0.991	0.992	1.1308E-85	T cells	Gm42418
1.9487E-89	0.26049363	0.479	0.412	4.8318E-85	T cells	Tnrc6b
3.3798E-89	0.35734167	0.347	0.29	8.3802E-85	T cells	Smc3
5.3152E-89	0.51368448	0.256	0.206	1.3179E-84	T cells	Hadhb
7.718E-89	0.29298957	0.456	0.394	1.9137E-84	T cells	Ndufa3
1.0759E-88	0.42031897	0.343	0.288	2.6678E-84	T cells	Hnrnpr
2.1468E-88	0.28067798	0.515	0.454	5.323E-84	T cells	Bclaf1
1.1967E-87	0.30387824	0.439	0.379	2.9672E-83	T cells	G3bp2
4.0595E-87	0.40061897	0.279	0.223	1.0066E-82	T cells	Cyth1
4.598E-87	0.39761145	0.417	0.359	1.1401E-82	T cells	Prpf4b
5.3169E-87	0.29180981	0.471	0.412	1.3183E-82	T cells	Ssb
1.0288E-86	0.32346011	0.324	0.261	2.551E-82	T cells	Arhgef18
1.518E-84	0.2541886	0.541	0.476	3.764E-80	T cells	Psma3
1.8571E-84	0.5063975	0.277	0.226	4.6047E-80	T cells	Tnks2
2.3803E-84	0.32659102	0.419	0.362	5.902E-80	T cells	Tsn
3.1066E-84	0.36735391	0.348	0.293	7.7028E-80	T cells	Ewsr1
1.2772E-83	0.33741914	0.385	0.329	3.1668E-79	T cells	Mrps14
1.8962E-83	0.38221348	0.336	0.283	4.7016E-79	T cells	Eif3g
9.9865E-81	0.47780524	0.273	0.224	2.4761E-76	T cells	Gspt1
4.8429E-79	0.39488587	0.356	0.302	1.2008E-74	T cells	Ddx46
8.5143E-79	0.48639272	0.265	0.218	2.1111E-74	T cells	Rex1bd
2.0743E-78	0.30656979	0.421	0.366	5.1433E-74	T cells	Atp5k
2.8367E-78	0.36929904	0.313	0.256	7.0335E-74	T cells	Chd2
3.1465E-78	0.45255391	0.261	0.212	7.8018E-74	T cells	Nudcd3
3.6585E-78	0.32712489	0.346	0.288	9.0712E-74	T cells	Nfatc3
5.1454E-77	0.41800475	0.29	0.237	1.2758E-72	T cells	Tcf12
9.9151E-77	0.41822438	0.302	0.253	2.4584E-72	T cells	Rpp21
4.8821E-76	0.50533241	0.305	0.256	1.2105E-71	T cells	Tuba1b
6.9546E-76	0.43815277	0.295	0.247	1.7244E-71	T cells	Anapc5
1.1085E-75	0.38993013	0.309	0.259	2.7484E-71	T cells	Suclg1
2.3753E-75	0.36198846	0.29	0.24	5.8896E-71	T cells	Zfas1
3.3132E-75	0.37963068	0.384	0.335	8.215E-71	T cells	Smap1
3.7973E-75	0.38384596	0.318	0.266	9.4154E-71	T cells	Sf3b3
4.9283E-73	0.45354345	0.271	0.225	1.222E-68	T cells	Dhx15
4.459E-72	0.31044282	0.387	0.334	1.1056E-67	T cells	Sltm

4.5215E-72	0.34035114	0.318	0.263	1.1211E-67	T cells	Birc2
7.2671E-72	0.39036318	0.343	0.294	1.8019E-67	T cells	Srsf6
1.0579E-71	0.31974543	0.452	0.398	2.6231E-67	T cells	Arglu1
1.0743E-71	0.38007972	0.326	0.276	2.6637E-67	T cells	R3hdm1
3.0597E-71	0.25434308	0.457	0.4	7.5866E-67	T cells	Ctcf
3.355E-71	0.36347128	0.319	0.272	8.3187E-67	T cells	Cuta
6.1571E-71	0.29628906	0.398	0.344	1.5267E-66	T cells	Ndufb3
3.3025E-69	0.37201767	0.312	0.264	8.1885E-65	T cells	Abcf1
1.1026E-68	0.4202775	0.272	0.227	2.7339E-64	T cells	Mbd3
5.7905E-68	0.27536311	0.458	0.406	1.4358E-63	T cells	H2az2
3.5321E-67	0.36584149	0.299	0.251	8.7578E-63	T cells	Gpatch8
3.687E-67	0.38189719	0.295	0.247	9.1419E-63	T cells	Gtpbp4
5.9425E-67	0.36381188	0.3	0.252	1.4734E-62	T cells	Rdx
7.3897E-67	0.27640807	0.407	0.355	1.8323E-62	T cells	Smc6
9.2046E-67	0.3698607	0.295	0.247	2.2823E-62	T cells	Rnaset2a
2.2375E-66	0.25247063	0.427	0.374	5.5478E-62	T cells	Dnaja2
6.0195E-66	0.3585453	0.296	0.25	1.4925E-61	T cells	Txn2
1.0298E-65	0.28126134	0.41	0.359	2.5533E-61	T cells	Eif4b
1.6773E-65	0.33425801	0.342	0.295	4.159E-61	T cells	Uqcrc2
3.4435E-63	0.41394106	0.277	0.233	8.5382E-59	T cells	Eif2s1
3.6632E-62	0.45647853	0.257	0.215	9.0828E-58	T cells	Pds5a
1.5966E-61	0.30353244	0.407	0.358	3.9588E-57	T cells	Cdc42se2
2.713E-61	0.41101174	0.273	0.228	6.727E-57	T cells	Dhx9
3.2454E-61	0.38429572	0.315	0.272	8.0469E-57	T cells	Sptssa
3.925E-61	0.30271926	0.361	0.314	9.732E-57	T cells	Rrp1
6.1333E-61	0.39574437	0.322	0.278	1.5208E-56	T cells	Luc7l3
6.8412E-61	0.31891599	0.361	0.313	1.6963E-56	T cells	Brd1
1.1894E-60	0.31780132	0.299	0.254	2.949E-56	T cells	Lsm5
1.2365E-60	0.35887134	0.305	0.262	3.0658E-56	T cells	Rnf7
7.2777E-60	0.43498902	0.259	0.218	1.8045E-55	T cells	Eprs
7.7753E-59	0.37944605	0.294	0.251	1.9279E-54	T cells	Polr2m
3.8651E-58	0.34145387	0.253	0.211	9.5834E-54	T cells	Smad4
1.5584E-56	0.346068	0.312	0.269	3.8641E-52	T cells	Ndufc2
3.6507E-56	0.30352769	0.263	0.218	9.0518E-52	T cells	Gpbp1l1
4.6349E-56	0.25218094	0.367	0.32	1.1492E-51	T cells	Cacybp
6.4164E-56	0.3543783	0.28	0.237	1.5909E-51	T cells	Phf14
8.6607E-55	0.35503132	0.296	0.256	2.1474E-50	T cells	Chchd1
8.9403E-55	0.27257617	0.381	0.337	2.2167E-50	T cells	Snu13
1.2549E-54	0.30331626	0.342	0.297	3.1115E-50	T cells	Zc3h15
2.6162E-54	0.35741101	0.304	0.263	6.4868E-50	T cells	Anp32e
3.7118E-54	0.40633307	0.263	0.223	9.2033E-50	T cells	Selenoh
5.6514E-54	0.361639	0.256	0.215	1.4013E-49	T cells	Pkn2
5.8684E-54	0.30178653	0.317	0.271	1.4551E-49	T cells	Krit1
6.9466E-54	0.32512006	0.298	0.257	1.7224E-49	T cells	Ssrp1
1.1208E-53	0.43248214	0.29	0.252	2.7791E-49	T cells	Nek7
1.3177E-53	0.31024028	0.305	0.26	3.2673E-49	T cells	Taf15
3.0294E-53	0.28739726	0.332	0.288	7.5114E-49	T cells	Magoh
1.6172E-52	0.28393874	0.371	0.328	4.0099E-48	T cells	Khdrbs1
5.2765E-52	0.27718146	0.299	0.255	1.3083E-47	T cells	Csnk1g2
1.1659E-51	0.44180506	0.278	0.24	2.8909E-47	T cells	Syncrip

1.5977E-51	0.26643343	0.327	0.281	3.9614E-47	T cells	Fnbp1
5.1524E-51	0.27118605	0.359	0.314	1.2775E-46	T cells	Mdh1
2.2193E-50	0.34975798	0.301	0.263	5.5027E-46	T cells	Psmc3
2.8806E-49	0.30077872	0.305	0.266	7.1423E-45	T cells	Mrpl57
4.3547E-49	0.26375138	0.394	0.345	1.0798E-44	T cells	Dock10
6.0671E-49	0.31956054	0.374	0.331	1.5043E-44	T cells	Gm1966
8.3018E-49	0.26181902	0.391	0.35	2.0584E-44	T cells	Nono
2.2945E-48	0.31456393	0.258	0.218	5.6891E-44	T cells	Suz12
2.4183E-48	0.3170986	0.275	0.234	5.9961E-44	T cells	Rnps1
4.4504E-48	0.29962049	0.317	0.278	1.1035E-43	T cells	Srp19
9.5193E-48	0.33572606	0.271	0.232	2.3603E-43	T cells	Prmt1
1.9329E-47	0.27505786	0.297	0.255	4.7925E-43	T cells	Add1
7.3979E-46	0.30253306	0.292	0.254	1.8343E-41	T cells	Nudc
9.3506E-46	0.26570718	0.512	0.471	2.3185E-41	T cells	Dock2
3.5184E-45	0.300708	0.336	0.298	8.7238E-41	T cells	Snw1
6.1596E-45	0.31244809	0.281	0.244	1.5273E-40	T cells	Zbtb20
8.6497E-45	0.30101425	0.307	0.268	2.1447E-40	T cells	Pum1
1.9069E-44	0.4336258	0.261	0.229	4.7282E-40	T cells	Hif1a
2.8703E-42	0.31169512	0.304	0.268	7.117E-38	T cells	Jtb
3.2131E-42	0.27680466	0.328	0.291	7.9669E-38	T cells	Eif3d
6.1396E-42	0.25629598	0.304	0.265	1.5223E-37	T cells	Polr2g
2.4653E-41	0.30854032	0.293	0.257	6.1126E-37	T cells	Thoc2
2.7255E-41	0.37127562	0.257	0.225	6.7578E-37	T cells	Tmem208
4.5844E-41	0.36011982	0.292	0.257	1.1367E-36	T cells	6820431F20Ri
7.7561E-41	0.29627939	0.316	0.28	1.9231E-36	T cells	Polr2f
9.2397E-41	0.30325815	0.304	0.266	2.291E-36	T cells	Kdm5a
2.8145E-40	0.35708736	0.265	0.231	6.9787E-36	T cells	Zranb2
3.2246E-40	0.32135409	0.316	0.279	7.9953E-36	T cells	Usp34
8.3621E-40	0.32581637	0.302	0.267	2.0734E-35	T cells	Eif4g1
2.4254E-38	0.33440656	0.27	0.239	6.0138E-34	T cells	Psmd7
2.8129E-38	0.30751852	0.284	0.25	6.9745E-34	T cells	Srp72
4.6041E-38	0.29429158	0.325	0.29	1.1416E-33	T cells	U2surp
4.9086E-38	0.28372501	0.331	0.296	1.2171E-33	T cells	Hnrnpl
5.9647E-38	0.25939248	0.329	0.294	1.4789E-33	T cells	1110004F10Ri
1.479E-37	0.28548666	0.259	0.225	3.6672E-33	T cells	Utp3
1.4908E-37	0.26326787	0.31	0.277	3.6964E-33	T cells	Marc2
1.5491E-37	0.33167205	0.253	0.221	3.841E-33	T cells	Ssbp1
1.7287E-37	0.2630285	0.279	0.243	4.2863E-33	T cells	Cntrl
2.7368E-36	0.25362271	0.371	0.337	6.7858E-32	T cells	Pbrm1
2.7984E-36	0.29426201	0.32	0.288	6.9385E-32	T cells	Snrpd1
4.0143E-36	0.26711595	0.301	0.268	9.9534E-32	T cells	U2af1
7.6886E-36	0.25896645	0.331	0.298	1.9064E-31	T cells	Ndufv2
1.0576E-35	0.29452769	0.267	0.235	2.6223E-31	T cells	Anapc16
1.2213E-35	0.25620424	0.358	0.322	3.0283E-31	T cells	Mrpl52
1.1349E-34	0.32900284	0.261	0.229	2.8141E-30	T cells	Ep400
1.7711E-34	0.29865741	0.279	0.248	4.3914E-30	T cells	Ankrd17
4.056E-34	0.3190397	0.274	0.243	1.0057E-29	T cells	Ociad1
4.5807E-34	0.28342922	0.251	0.22	1.1358E-29	T cells	Anapc11
6.6786E-34	0.27113189	0.253	0.222	1.6559E-29	T cells	Mcmbp
9.425E-34	0.2676479	0.343	0.311	2.3369E-29	T cells	Tcp1

1.4294E-33	0.28777838	0.326	0.295	3.5442E-29	T cells	Ube3a
4.4834E-33	0.28021444	0.278	0.245	1.1117E-28	T cells	Srsf1
1.8468E-32	0.27750849	0.281	0.25	4.5791E-28	T cells	Cenpx
3.4256E-32	0.28308997	0.302	0.27	8.4937E-28	T cells	Ndufc1
1.0902E-31	0.27734154	0.28	0.249	2.7031E-27	T cells	Psmc5
1.8785E-31	0.26520444	0.304	0.273	4.6577E-27	T cells	Dcun1d5
4.1207E-30	0.31447913	0.325	0.297	1.0217E-25	T cells	Vcp
6.7653E-29	0.29060694	0.26	0.232	1.6775E-24	T cells	Wdr83os
4.0444E-28	0.28616648	0.294	0.266	1.0028E-23	T cells	Ctbp1
6.9027E-28	0.26084993	0.296	0.267	1.7115E-23	T cells	Trip12
1.9323E-27	0.26980416	0.273	0.246	4.791E-23	T cells	Chrac1
2.0202E-27	0.28464403	0.297	0.27	5.0092E-23	T cells	Metap2
1.6269E-26	0.6525556	0.36	0.341	4.034E-22	T cells	Itgb1
6.2606E-23	0.27192547	0.281	0.255	1.5523E-18	T cells	Rpn2
1.6847E-22	0.29051058	0.29	0.266	4.1772E-18	T cells	Ddrgk1
3.1931E-21	0.28470148	0.269	0.247	7.9173E-17	T cells	Cyc1
3.4301E-12	0.34275676	0.232	0.264	8.5049E-08	T cells	S100a4
0	4.26150937	0.986	0.165	0	NK/CD8 T cells	Nkg7
0	5.30830796	0.982	0.236	0	NK/CD8 T cells	Ccl5
0	3.92559166	0.852	0.11	0	NK/CD8 T cells	Ctsw
0	5.47380467	0.683	0.035	0	NK/CD8 T cells	Klrk1
0	3.76511162	0.737	0.105	0	NK/CD8 T cells	Klrd1
0	1.95225653	0.976	0.345	0	NK/CD8 T cells	Ms4a4b
0	2.67427911	0.884	0.258	0	NK/CD8 T cells	Ly6a
0	3.1902554	0.702	0.095	0	NK/CD8 T cells	Ctla2a
0	3.32875113	0.715	0.115	0	NK/CD8 T cells	Il2rb
0	3.27244627	0.979	0.382	0	NK/CD8 T cells	AW112010
0	2.59165096	0.792	0.238	0	NK/CD8 T cells	Id2
0	4.12296875	0.599	0.047	0	NK/CD8 T cells	Cxcr6
0	1.77784744	0.802	0.257	0	NK/CD8 T cells	Lck
0	2.45001679	0.803	0.259	0	NK/CD8 T cells	Lgals1
0	1.32423345	0.873	0.337	0	NK/CD8 T cells	Trbc2
0	3.68987978	0.601	0.072	0	NK/CD8 T cells	Ccl4
0	3.00879202	0.707	0.184	0	NK/CD8 T cells	Rgs1
0	5.90781045	0.531	0.015	0	NK/CD8 T cells	Klre1
0	1.58588038	0.759	0.251	0	NK/CD8 T cells	Skap1
0	1.37160905	0.805	0.311	0	NK/CD8 T cells	Ahnak
0	3.36141554	0.594	0.101	0	NK/CD8 T cells	Cd7
0	1.85613848	0.798	0.313	0	NK/CD8 T cells	Cd3g
0	4.81511953	0.509	0.024	0	NK/CD8 T cells	Fasl
0	6.34447852	0.497	0.023	0	NK/CD8 T cells	Xcl1
0	4.37859249	0.534	0.063	0	NK/CD8 T cells	Prf1
0	1.90498195	0.762	0.297	0	NK/CD8 T cells	Hcst
0	2.03375511	0.672	0.213	0	NK/CD8 T cells	Thy1
0	2.01948841	0.685	0.233	0	NK/CD8 T cells	Ptpn22
0	1.53980842	0.738	0.291	0	NK/CD8 T cells	Cd3e
0	2.74978106	0.525	0.091	0	NK/CD8 T cells	Cxcr3
0	1.0419851	0.875	0.446	0	NK/CD8 T cells	Ctsd
0	1.42236956	0.714	0.286	0	NK/CD8 T cells	Itgb1
0	1.37098759	0.658	0.239	0	NK/CD8 T cells	Txk

0	1.60853083	0.835	0.418	0	NK/CD8 T cells	H2-Q7
0	2.14464084	0.638	0.222	0	NK/CD8 T cells	Runx3
0	2.40523003	0.565	0.153	0	NK/CD8 T cells	Gimap7
0	1.72304209	0.62	0.215	0	NK/CD8 T cells	Rinl
0	1.18577324	0.746	0.347	0	NK/CD8 T cells	Cd3d
0	4.61982012	0.418	0.023	0	NK/CD8 T cells	Ifng
0	1.40008909	0.781	0.387	0	NK/CD8 T cells	Sept1
0	1.26798595	0.781	0.39	0	NK/CD8 T cells	Cd2
0	1.81469556	0.638	0.247	0	NK/CD8 T cells	Fyn
0	2.40170227	0.487	0.097	0	NK/CD8 T cells	Il18r1
0	1.24725891	0.596	0.207	0	NK/CD8 T cells	Trbc1
0	1.51983054	0.677	0.292	0	NK/CD8 T cells	Gimap3
0	1.28517575	0.64	0.257	0	NK/CD8 T cells	Lat
0	1.89789167	0.725	0.342	0	NK/CD8 T cells	Dusp2
0	6.38344817	0.385	0.013	0	NK/CD8 T cells	Ncr1
0	1.39699204	0.659	0.288	0	NK/CD8 T cells	Bcl2
0	3.37902597	0.425	0.055	0	NK/CD8 T cells	Cst7
0	1.15360934	0.885	0.515	0	NK/CD8 T cells	Ptprcap
0	0.50239623	0.85	0.481	0	NK/CD8 T cells	S100a6
0	4.95709502	0.387	0.018	0	NK/CD8 T cells	Trgc2
0	4.73326106	0.388	0.025	0	NK/CD8 T cells	Itga1
0	2.74058066	0.468	0.107	0	NK/CD8 T cells	Hopx
0	5.49663095	0.369	0.011	0	NK/CD8 T cells	Klrc2
0	2.24590758	0.469	0.114	0	NK/CD8 T cells	Sh2d1a
0	1.50238611	0.478	0.123	0	NK/CD8 T cells	Ccr2
0	5.60155378	0.358	0.011	0	NK/CD8 T cells	Klrc1
0	1.37251528	0.551	0.208	0	NK/CD8 T cells	S100a4
0	1.77306771	0.499	0.159	0	NK/CD8 T cells	Fam189b
0	1.81218824	0.535	0.197	0	NK/CD8 T cells	Dusp5
0	4.07788339	0.369	0.032	0	NK/CD8 T cells	Eomes
0	1.18445323	0.705	0.37	0	NK/CD8 T cells	Gimap4
0	3.68991373	0.37	0.035	0	NK/CD8 T cells	Sytl3
0	3.02263531	0.433	0.099	0	NK/CD8 T cells	Cd8a
0	1.23729911	0.595	0.263	0	NK/CD8 T cells	Efhd2
0	2.40444034	0.431	0.101	0	NK/CD8 T cells	Sh2d2a
0	1.32447602	0.56	0.234	0	NK/CD8 T cells	Spn
0	2.58436716	0.421	0.096	0	NK/CD8 T cells	Dok2
0	0.51828866	0.93	0.607	0	NK/CD8 T cells	Crip1
0	1.27889728	0.534	0.215	0	NK/CD8 T cells	Cd27
0	1.23468373	0.965	0.646	0	NK/CD8 T cells	S100a10
0	2.63468375	0.399	0.083	0	NK/CD8 T cells	Cd226
0	3.87988156	0.353	0.04	0	NK/CD8 T cells	Klra1
0	0.99909471	0.523	0.212	0	NK/CD8 T cells	Cd28
0	2.56119248	0.404	0.093	0	NK/CD8 T cells	Gm19585
0	3.8359061	0.331	0.023	0	NK/CD8 T cells	Trdc
0	2.55810285	0.407	0.1	0	NK/CD8 T cells	Cd8b1
0	0.4745839	0.876	0.57	0	NK/CD8 T cells	Selplg
0	1.5732685	0.511	0.207	0	NK/CD8 T cells	Gimap5
0	1.18214885	0.622	0.32	0	NK/CD8 T cells	Ccnd2
0	1.22588128	0.643	0.342	0	NK/CD8 T cells	S100a13

0	5.30094045	0.318	0.017	0	NK/CD8 T cells	Gzmb
0	5.03455984	0.314	0.013	0	NK/CD8 T cells	Gzmk
0	1.02348758	0.553	0.256	0	NK/CD8 T cells	Trac
0	1.6514978	0.568	0.271	0	NK/CD8 T cells	Ugcg
0	3.36809503	0.352	0.057	0	NK/CD8 T cells	Ccr5
0	1.90683634	0.425	0.133	0	NK/CD8 T cells	Prkch
0	1.00943616	0.719	0.428	0	NK/CD8 T cells	Gm4759
0	5.87021966	0.352	0.065	0	NK/CD8 T cells	Gzma
0	2.32750493	0.391	0.105	0	NK/CD8 T cells	Bhlhe40
0	1.26621991	0.459	0.174	0	NK/CD8 T cells	Itk
0	0.98479254	0.714	0.429	0	NK/CD8 T cells	Pdcd4
0	0.95615154	0.593	0.309	0	NK/CD8 T cells	Arl4c
0	0.89443785	0.749	0.465	0	NK/CD8 T cells	Ms4a6b
0	2.2256643	0.38	0.098	0	NK/CD8 T cells	Gm15472
0	1.2342534	0.551	0.269	0	NK/CD8 T cells	Saraf
0	1.02731097	0.752	0.471	0	NK/CD8 T cells	Ndfip1
0	1.66127831	0.368	0.088	0	NK/CD8 T cells	Il18rap
0	1.04869335	0.661	0.383	0	NK/CD8 T cells	Clec2d
0	1.0589871	0.497	0.22	0	NK/CD8 T cells	Bcl11b
0	1.89105649	0.409	0.133	0	NK/CD8 T cells	Hsd11b1
0	1.30394302	0.498	0.223	0	NK/CD8 T cells	Stat4
0	3.78981839	0.304	0.03	0	NK/CD8 T cells	Ifitm10
0	1.32118533	0.425	0.152	0	NK/CD8 T cells	Prkcq
0	4.04844069	0.303	0.03	0	NK/CD8 T cells	Serpinb9
0	1.22522098	0.63	0.358	0	NK/CD8 T cells	Cd48
0	1.92267337	0.372	0.103	0	NK/CD8 T cells	Zap70
0	4.31406059	0.29	0.024	0	NK/CD8 T cells	Serpinb6b
0	1.19406951	0.576	0.311	0	NK/CD8 T cells	Smap1
0	1.19139412	0.592	0.327	0	NK/CD8 T cells	Arl6ip5
0	0.26254716	0.745	0.482	0	NK/CD8 T cells	Fyb
0	4.00145545	0.28	0.019	0	NK/CD8 T cells	Spry2
0	0.55138426	0.742	0.481	0	NK/CD8 T cells	Ablim1
0	0.89939826	0.831	0.572	0	NK/CD8 T cells	Ndufa4
0	4.27568311	0.277	0.018	0	NK/CD8 T cells	Tbx21
0	4.00008323	0.277	0.018	0	NK/CD8 T cells	Trgc4
0	2.76306403	0.318	0.061	0	NK/CD8 T cells	Slamf7
0	0.50253667	0.691	0.434	0	NK/CD8 T cells	Itgb2
0	3.559379	0.286	0.032	0	NK/CD8 T cells	Camk2n1
0	1.11660693	0.507	0.253	0	NK/CD8 T cells	Gimap9
0	1.22118804	0.468	0.215	0	NK/CD8 T cells	Ifi47
0	0.378751	0.607	0.356	0	NK/CD8 T cells	Itgal
0	1.59727521	0.504	0.253	0	NK/CD8 T cells	BC018473
0	1.93732243	0.376	0.125	0	NK/CD8 T cells	Cd6
0	0.77182122	0.643	0.393	0	NK/CD8 T cells	Gimap1
0	1.67204664	0.37	0.12	0	NK/CD8 T cells	Chsy1
0	0.94661972	0.523	0.273	0	NK/CD8 T cells	Leprotl1
0	1.3320731	0.443	0.193	0	NK/CD8 T cells	Pitpnc1
0	1.3949998	0.475	0.225	0	NK/CD8 T cells	Tpst2
0	0.77199875	0.674	0.425	0	NK/CD8 T cells	Ifngr1
0	1.42952736	0.456	0.208	0	NK/CD8 T cells	Plaat3

0	1.65026486	0.453	0.206	0	NK/CD8 T cells	Dnajc15
0	0.73442264	0.843	0.596	0	NK/CD8 T cells	Tspo
0	2.65767264	0.309	0.063	0	NK/CD8 T cells	St6galnac2
0	0.84842819	0.575	0.333	0	NK/CD8 T cells	Anxa6
0	0.89780157	0.513	0.274	0	NK/CD8 T cells	Prex1
0	1.89143798	0.351	0.113	0	NK/CD8 T cells	AU020206
0	1.40645625	0.518	0.28	0	NK/CD8 T cells	Epsti1
0	3.44323572	0.265	0.028	0	NK/CD8 T cells	Osbpl3
0	1.32023841	0.369	0.132	0	NK/CD8 T cells	Gm2682
0	0.98638305	0.395	0.159	0	NK/CD8 T cells	Sema4a
0	0.59421509	0.804	0.569	0	NK/CD8 T cells	Emp3
0	0.96116761	0.879	0.645	0	NK/CD8 T cells	H2az1
0	0.74721918	0.932	0.699	0	NK/CD8 T cells	Mbnl1
0	0.78334344	0.907	0.677	0	NK/CD8 T cells	Atp5h
0	0.91179476	0.515	0.286	0	NK/CD8 T cells	Chd3
0	0.99728405	0.474	0.245	0	NK/CD8 T cells	Tnfaip3
0	1.14696574	0.491	0.262	0	NK/CD8 T cells	Esyt1
0	1.28722009	0.417	0.189	0	NK/CD8 T cells	Nabp1
0	0.830816	0.506	0.282	0	NK/CD8 T cells	Cd82
0	0.83180914	0.897	0.673	0	NK/CD8 T cells	Tle5
0	1.33566834	0.357	0.133	0	NK/CD8 T cells	St3gal6
0	0.72858258	0.788	0.565	0	NK/CD8 T cells	Atp5j2
0	1.12773115	0.43	0.207	0	NK/CD8 T cells	Tes
0	1.73823167	0.267	0.045	0	NK/CD8 T cells	Ccl3
0	0.85663162	0.558	0.336	0	NK/CD8 T cells	Smad7
0	0.7491016	0.715	0.494	0	NK/CD8 T cells	Macroh2a1
0	1.17590848	0.379	0.159	0	NK/CD8 T cells	Gbp7
0	0.73343486	0.863	0.643	0	NK/CD8 T cells	Sumo2
0	0.31327798	0.819	0.6	0	NK/CD8 T cells	Vim
0	0.69366817	0.744	0.525	0	NK/CD8 T cells	Cox5a
0	3.06672327	0.267	0.049	0	NK/CD8 T cells	Nr4a2
0	1.02007858	0.462	0.246	0	NK/CD8 T cells	Pde7a
0	1.0197834	0.499	0.283	0	NK/CD8 T cells	Vgll4
0	0.99604111	0.504	0.289	0	NK/CD8 T cells	Znrf2
0	0.7694566	0.677	0.463	0	NK/CD8 T cells	Cox7b
0	1.51258305	0.344	0.13	0	NK/CD8 T cells	Peak1
0	0.80268778	0.888	0.674	0	NK/CD8 T cells	Psmb8
0	0.27236309	0.799	0.588	0	NK/CD8 T cells	Ets1
0	1.14589638	0.407	0.197	0	NK/CD8 T cells	Rasal3
0	1.46469263	0.331	0.122	0	NK/CD8 T cells	Tnik
0	1.33262044	0.374	0.165	0	NK/CD8 T cells	Pfkp
0	0.77763558	0.64	0.432	0	NK/CD8 T cells	Ppp1r12a
0	0.72684442	0.677	0.47	0	NK/CD8 T cells	Ran
0	0.66885734	0.791	0.584	0	NK/CD8 T cells	Ndufa13
0	0.66418701	0.83	0.623	0	NK/CD8 T cells	Tma7
0	0.79602156	0.742	0.535	0	NK/CD8 T cells	Myl12a
0	1.11746932	0.472	0.265	0	NK/CD8 T cells	Slc3a2
0	2.19782251	0.271	0.065	0	NK/CD8 T cells	Atp2b4
0	1.05770773	0.354	0.148	0	NK/CD8 T cells	Cd247
0	0.963206	0.418	0.212	0	NK/CD8 T cells	Ikzf3

0	0.62570405	0.832	0.626	0	NK/CD8 T cells	Serbp1
0	0.67849937	0.568	0.362	0	NK/CD8 T cells	Tbc1d10c
0	0.81443761	0.627	0.422	0	NK/CD8 T cells	Drap1
0	0.89733885	0.476	0.271	0	NK/CD8 T cells	Itpkb
0	1.37409702	0.355	0.151	0	NK/CD8 T cells	D16Ertd472e
0	1.12670294	0.478	0.274	0	NK/CD8 T cells	Pik3r1
0	1.08937717	0.432	0.229	0	NK/CD8 T cells	Aplp2
0	1.97006338	0.264	0.063	0	NK/CD8 T cells	Rora
0	0.69701686	0.723	0.522	0	NK/CD8 T cells	Jpt1
0	0.63751603	0.715	0.514	0	NK/CD8 T cells	Cox6c
0	0.55612119	0.654	0.454	0	NK/CD8 T cells	Ifi203
0	1.27421811	0.394	0.194	0	NK/CD8 T cells	Prkacb
0	0.65626167	0.742	0.542	0	NK/CD8 T cells	Atp5j
0	0.39064602	0.681	0.482	0	NK/CD8 T cells	Gimap6
0	0.78508337	0.679	0.48	0	NK/CD8 T cells	Abracl
0	1.83525459	0.295	0.096	0	NK/CD8 T cells	Lax1
0	0.7636905	0.614	0.416	0	NK/CD8 T cells	1810037I17Ri
0	0.66432936	0.671	0.476	0	NK/CD8 T cells	Ndufb7
0	0.74600848	0.632	0.439	0	NK/CD8 T cells	Psmb3
0	0.69747993	0.672	0.48	0	NK/CD8 T cells	Dnaja1
0	0.53056159	0.88	0.689	0	NK/CD8 T cells	Sub1
0	1.49530312	0.34	0.149	0	NK/CD8 T cells	Dnajc1
0	0.9451079	0.399	0.208	0	NK/CD8 T cells	Otulinl
0	0.41149638	0.813	0.622	0	NK/CD8 T cells	Ppib
0	0.86208641	0.492	0.302	0	NK/CD8 T cells	Sept6
0	0.67893496	0.707	0.517	0	NK/CD8 T cells	Sp100
0	0.67938731	0.585	0.395	0	NK/CD8 T cells	Lrrfip1
0	0.58356686	0.671	0.482	0	NK/CD8 T cells	Spcs2
0	0.54366288	0.637	0.448	0	NK/CD8 T cells	Il2rg
0	1.15923863	0.371	0.182	0	NK/CD8 T cells	H2-Q6
0	0.93534205	0.574	0.385	0	NK/CD8 T cells	Ywhaq
0	0.58031979	0.615	0.427	0	NK/CD8 T cells	A130071D04R
0	0.87970411	0.527	0.339	0	NK/CD8 T cells	Ndufv3
0	0.81497473	0.418	0.23	0	NK/CD8 T cells	Lfng
0	0.53984062	0.737	0.549	0	NK/CD8 T cells	Cox7c
0	0.91284609	0.498	0.311	0	NK/CD8 T cells	Fkbp3
0	0.6501624	0.637	0.451	0	NK/CD8 T cells	Krtcap2
0	1.03489321	0.436	0.25	0	NK/CD8 T cells	Rnaseh2c
0	0.41257886	0.942	0.756	0	NK/CD8 T cells	Hsp90ab1
0	0.59546329	0.767	0.583	0	NK/CD8 T cells	Ccnd3
0	0.92561413	0.451	0.267	0	NK/CD8 T cells	Fnbp1
0	1.25786509	0.386	0.204	0	NK/CD8 T cells	Med10
0	1.92097987	0.303	0.121	0	NK/CD8 T cells	Rcbtb2
0	0.59486862	0.619	0.437	0	NK/CD8 T cells	Ppp1r18
0	0.58715369	0.728	0.546	0	NK/CD8 T cells	Cox7a2
0	1.47878706	0.31	0.129	0	NK/CD8 T cells	Pld3
0	0.54390062	0.987	0.806	0	NK/CD8 T cells	Ppia
0	1.45420689	0.353	0.173	0	NK/CD8 T cells	Ybx3
0	0.76390727	0.861	0.681	0	NK/CD8 T cells	Jak1
0	1.04771795	0.385	0.207	0	NK/CD8 T cells	Prrc2b

0	0.57122265	0.663	0.486	0	NK/CD8 T cells	Dad1
0	0.97061714	0.397	0.22	0	NK/CD8 T cells	Inpp4b
0	0.74162261	0.469	0.292	0	NK/CD8 T cells	Slc9a3r1
0	0.62623775	0.657	0.481	0	NK/CD8 T cells	Edf1
0	1.00071069	0.397	0.221	0	NK/CD8 T cells	Irf2bpl
0	1.64275312	0.267	0.091	0	NK/CD8 T cells	Socs1
0	0.68996752	0.618	0.442	0	NK/CD8 T cells	Sept7
0	0.92857398	0.363	0.187	0	NK/CD8 T cells	Bcl2a1b
0	0.48146282	0.735	0.559	0	NK/CD8 T cells	Selenow
0	0.73296519	0.525	0.35	0	NK/CD8 T cells	Evl
0	1.00286487	0.375	0.2	0	NK/CD8 T cells	Rbl2
0	0.5059196	0.791	0.617	0	NK/CD8 T cells	Ptpn18
0	0.57334523	0.68	0.506	0	NK/CD8 T cells	Set
0	0.50398587	0.745	0.571	0	NK/CD8 T cells	Psme1
0	0.82392168	0.895	0.721	0	NK/CD8 T cells	Zfp36l2
0	1.0977313	0.33	0.156	0	NK/CD8 T cells	Gimap8
0	1.30300092	0.288	0.114	0	NK/CD8 T cells	Ttc39b
0	0.60428747	0.589	0.415	0	NK/CD8 T cells	Vps28
0	0.67436918	0.567	0.393	0	NK/CD8 T cells	Uqcrb
0	1.39053529	0.283	0.11	0	NK/CD8 T cells	Themis
0	1.0026019	0.317	0.145	0	NK/CD8 T cells	Spata13
0	1.46742373	0.252	0.08	0	NK/CD8 T cells	Gata3
0	0.68930811	0.605	0.433	0	NK/CD8 T cells	Gabarapl2
0	0.95147413	0.369	0.198	0	NK/CD8 T cells	Traf1
0	0.56810496	0.504	0.333	0	NK/CD8 T cells	Zdhhc18
0	0.72409911	0.523	0.353	0	NK/CD8 T cells	Ankrd12
0	0.69922085	0.974	0.804	0	NK/CD8 T cells	Tmsb10
0	0.4909033	0.744	0.575	0	NK/CD8 T cells	Ncl
0	0.87095264	0.99	0.821	0	NK/CD8 T cells	H2-K1
0	1.28385548	0.319	0.15	0	NK/CD8 T cells	Rnf166
0	0.45970626	0.764	0.596	0	NK/CD8 T cells	Cox6b1
0	0.8636532	0.452	0.284	0	NK/CD8 T cells	Pdcd6ip
0	0.5673445	0.659	0.492	0	NK/CD8 T cells	Tomm7
0	0.51747775	0.715	0.548	0	NK/CD8 T cells	Srp14
0	0.4490956	0.716	0.549	0	NK/CD8 T cells	Snrpg
0	0.52442262	0.941	0.774	0	NK/CD8 T cells	Shisa5
0	1.03858221	0.389	0.223	0	NK/CD8 T cells	Ssbp4
0	0.82397289	0.422	0.256	0	NK/CD8 T cells	Ifi27
0	1.1415022	0.312	0.147	0	NK/CD8 T cells	Aak1
0	0.79830609	0.496	0.331	0	NK/CD8 T cells	Bag1
0	0.82405723	0.419	0.254	0	NK/CD8 T cells	Rnf138
0	0.46999943	0.747	0.583	0	NK/CD8 T cells	Cnn2
0	1.35715306	0.334	0.17	0	NK/CD8 T cells	Sept11
0	0.55948507	0.583	0.419	0	NK/CD8 T cells	Prdx6
0	0.78817181	0.342	0.179	0	NK/CD8 T cells	Tnfrsf18
0	0.48052133	0.675	0.512	0	NK/CD8 T cells	Uqcrq
0	0.76731841	0.499	0.336	0	NK/CD8 T cells	Mapre1
0	1.22843039	0.278	0.116	0	NK/CD8 T cells	Rnf125
0	0.84533852	0.413	0.251	0	NK/CD8 T cells	Adcy7
0	0.5880317	0.624	0.462	0	NK/CD8 T cells	Prkar1a

0	0.51389745	0.689	0.527	0	NK/CD8 T cells	Cox5b
0	0.47012755	0.703	0.542	0	NK/CD8 T cells	Atp5d
0	0.42415482	0.888	0.728	0	NK/CD8 T cells	Gnas
0	0.335779	0.894	0.734	0	NK/CD8 T cells	Rpl27
0	0.54979339	0.599	0.439	0	NK/CD8 T cells	Psme2
0	0.46133918	0.746	0.587	0	NK/CD8 T cells	Hsp90aa1
0	0.56868414	0.556	0.398	0	NK/CD8 T cells	H2az2
0	0.3910594	0.977	0.819	0	NK/CD8 T cells	Rpl38
0	0.50736579	0.657	0.499	0	NK/CD8 T cells	Ndufa2
0	1.33158415	0.283	0.125	0	NK/CD8 T cells	Ppp3cc
0	0.47507194	0.494	0.336	0	NK/CD8 T cells	Cyth4
0	1.04856554	0.337	0.18	0	NK/CD8 T cells	B4galt1
0	0.34946429	0.731	0.574	0	NK/CD8 T cells	Ostf1
0	0.52378583	0.469	0.312	0	NK/CD8 T cells	Vps37b
0	0.65120082	0.512	0.356	0	NK/CD8 T cells	Ndufa1
0	0.72744198	0.517	0.361	0	NK/CD8 T cells	Psmb10
0	0.49675256	0.81	0.654	0	NK/CD8 T cells	Calm2
0	0.45167859	0.659	0.504	0	NK/CD8 T cells	Anp32b
0	0.76886625	0.404	0.249	0	NK/CD8 T cells	Slbp
0	0.41609689	0.507	0.352	0	NK/CD8 T cells	Pycard
0	1.20099629	0.337	0.182	0	NK/CD8 T cells	Ccdc85b
0	1.00415205	0.391	0.236	0	NK/CD8 T cells	Npm3
0	0.72693077	0.365	0.21	0	NK/CD8 T cells	Gm37033
0	0.41659467	0.735	0.581	0	NK/CD8 T cells	Cnbp
0	0.54325238	0.548	0.394	0	NK/CD8 T cells	Sec11a
0	0.67534969	0.518	0.364	0	NK/CD8 T cells	Mif
0	0.47042965	0.725	0.571	0	NK/CD8 T cells	Elob
0	0.38081918	0.882	0.728	0	NK/CD8 T cells	Clic1
0	0.47832569	0.59	0.436	0	NK/CD8 T cells	Mndal
0	0.47241182	0.614	0.46	0	NK/CD8 T cells	Reep5
0	0.48070745	0.668	0.515	0	NK/CD8 T cells	Ndufb11
0	0.52223492	0.634	0.481	0	NK/CD8 T cells	Psma2
0	1.33170013	0.282	0.129	0	NK/CD8 T cells	Tapbpl
0	0.55238884	0.431	0.278	0	NK/CD8 T cells	Arhgap9
0	0.68982508	0.343	0.191	0	NK/CD8 T cells	Utrn
0	1.20775397	0.276	0.124	0	NK/CD8 T cells	Abhd8
0	0.69990541	0.459	0.307	0	NK/CD8 T cells	Gng2
0	0.68321053	0.532	0.38	0	NK/CD8 T cells	Nme1
0	0.44432431	0.584	0.432	0	NK/CD8 T cells	Bin2
0	0.49213599	0.665	0.514	0	NK/CD8 T cells	Rbx1
0	0.60852072	0.931	0.78	0	NK/CD8 T cells	Cox8a
0	0.74846537	0.381	0.23	0	NK/CD8 T cells	Tespa1
0	0.62632422	0.52	0.37	0	NK/CD8 T cells	Dnajb6
0	0.58287203	0.598	0.448	0	NK/CD8 T cells	Mbd2
0	1.35362229	0.276	0.127	0	NK/CD8 T cells	Bex3
0	0.49127498	0.635	0.486	0	NK/CD8 T cells	Cox6a1
0	0.97555578	0.378	0.229	0	NK/CD8 T cells	Ech1
0	1.45076233	0.266	0.118	0	NK/CD8 T cells	Nsmaf
0	0.50970723	0.629	0.481	0	NK/CD8 T cells	Ube2i
0	1.0014094	0.361	0.213	0	NK/CD8 T cells	Mxd4

0	0.65765866	0.456	0.309	0	NK/CD8 T cells	Dnajc3
0	0.4117851	0.762	0.615	0	NK/CD8 T cells	Ubl5
0	0.56594306	0.455	0.308	0	NK/CD8 T cells	Cyfip2
0	1.1740942	0.277	0.13	0	NK/CD8 T cells	Iqgap2
0	0.84554817	0.374	0.227	0	NK/CD8 T cells	Plec
0	0.57542409	0.569	0.422	0	NK/CD8 T cells	Ywhab
0	0.80921053	0.94	0.793	0	NK/CD8 T cells	Sh3bgrl3
0	0.5444846	0.559	0.413	0	NK/CD8 T cells	Tmed9
0	1.13074456	0.265	0.12	0	NK/CD8 T cells	Mllt3
0	0.58130962	0.496	0.351	0	NK/CD8 T cells	Rsbn1l
0	0.55758638	0.546	0.401	0	NK/CD8 T cells	Tbca
0	0.58505209	0.423	0.278	0	NK/CD8 T cells	Ccdc88c
0	0.59121791	0.536	0.392	0	NK/CD8 T cells	Atp5md
0	0.72536924	0.459	0.316	0	NK/CD8 T cells	Rnf187
0	0.57193791	0.542	0.399	0	NK/CD8 T cells	Sfr1
0	0.78268942	0.37	0.227	0	NK/CD8 T cells	Sept9
0	0.70285227	0.367	0.224	0	NK/CD8 T cells	Grap2
0	0.7618575	0.374	0.231	0	NK/CD8 T cells	H2-Q4
0	0.64161753	0.268	0.126	0	NK/CD8 T cells	Rgs3
0	1.0665201	0.275	0.133	0	NK/CD8 T cells	Klk8
0	0.42758476	0.732	0.59	0	NK/CD8 T cells	Arl6ip1
0	0.8034119	0.394	0.252	0	NK/CD8 T cells	Taf15
0	0.55485358	0.549	0.408	0	NK/CD8 T cells	Ssb
0	0.64495505	0.501	0.361	0	NK/CD8 T cells	Atp5k
0	0.57452826	0.527	0.387	0	NK/CD8 T cells	Dek
0	0.9144602	0.357	0.218	0	NK/CD8 T cells	Cetn2
0	0.66993667	0.411	0.272	0	NK/CD8 T cells	Cited2
0	0.39597197	0.793	0.655	0	NK/CD8 T cells	Serf2
0	0.35750872	0.889	0.751	0	NK/CD8 T cells	Chchd2
0	1.17379982	0.283	0.145	0	NK/CD8 T cells	Usp48
0	0.51854915	0.589	0.451	0	NK/CD8 T cells	Psmb9
0	0.35579786	0.816	0.678	0	NK/CD8 T cells	Srrm2
0	1.23492165	0.265	0.128	0	NK/CD8 T cells	H2-T10
0	0.90434683	0.345	0.208	0	NK/CD8 T cells	Nab1
0	0.76471931	0.365	0.228	0	NK/CD8 T cells	Med30
0	1.0609637	0.297	0.161	0	NK/CD8 T cells	Lrrc8c
0	0.95680576	0.306	0.17	0	NK/CD8 T cells	Rhof
0	0.62647592	0.341	0.206	0	NK/CD8 T cells	Tecpr1
0	0.76401516	0.422	0.287	0	NK/CD8 T cells	Med28
0	0.98621091	0.34	0.206	0	NK/CD8 T cells	Higd1a
0	0.68231277	0.376	0.242	0	NK/CD8 T cells	Il10rb
0	0.82375103	0.399	0.265	0	NK/CD8 T cells	Cuta
0	1.03391277	0.323	0.19	0	NK/CD8 T cells	Mrpl58
0	0.65288157	0.471	0.338	0	NK/CD8 T cells	Atp5g1
0	0.84581957	0.328	0.196	0	NK/CD8 T cells	Arl2bp
0	0.74244264	0.326	0.194	0	NK/CD8 T cells	Acap1
0	0.67312839	0.456	0.325	0	NK/CD8 T cells	Mrps14
0	0.3740761	0.905	0.774	0	NK/CD8 T cells	Atp5e
0	0.87926581	0.34	0.21	0	NK/CD8 T cells	Scamp3
0	0.75365633	0.965	0.835	0	NK/CD8 T cells	Myl6

0	0.75561864	0.417	0.288	0	NK/CD8 T cells	Dpm3
0	1.0185682	0.283	0.154	0	NK/CD8 T cells	Ly9
0	0.78928994	0.276	0.148	0	NK/CD8 T cells	Ehd3
0	1.26076863	0.252	0.126	0	NK/CD8 T cells	Ptpn7
0	0.96643219	0.258	0.132	0	NK/CD8 T cells	Fam78a
0	0.97625518	0.317	0.191	0	NK/CD8 T cells	Egln1
0	0.32734191	0.957	0.831	0	NK/CD8 T cells	Calm1
0	0.81022937	0.309	0.184	0	NK/CD8 T cells	Fam53b
0	0.78792346	0.304	0.179	0	NK/CD8 T cells	Def6
0	1.25664764	0.261	0.137	0	NK/CD8 T cells	Cmc1
0	0.95589359	0.254	0.132	0	NK/CD8 T cells	Cd5
0	0.89451526	0.319	0.198	0	NK/CD8 T cells	Efr3a
0	0.90908824	0.273	0.154	0	NK/CD8 T cells	Rassf1
0	0.51031563	0.961	0.843	0	NK/CD8 T cells	Rac2
0	0.46484364	0.952	0.837	0	NK/CD8 T cells	Cfl1
0	0.35294359	0.962	0.855	0	NK/CD8 T cells	Oaz1
0	0.91292271	0.991	0.906	0	NK/CD8 T cells	Cd52
0	0.64582828	0.992	0.934	0	NK/CD8 T cells	Pfn1
0	0.40048143	0.995	0.954	0	NK/CD8 T cells	B2m
0	0.43573137	0.998	0.968	0	NK/CD8 T cells	H2-D1
1.797E-302	0.67323629	0.415	0.286	4.455E-298	NK/CD8 T cells	Fkbp1a
2.44E-301	0.4109691	0.739	0.583	6.049E-297	NK/CD8 T cells	Dynll1
7.992E-301	0.55058145	0.512	0.368	1.982E-296	NK/CD8 T cells	Capns1
9.587E-297	0.50791495	0.563	0.429	2.377E-292	NK/CD8 T cells	Sumo1
7.908E-296	0.5121918	0.379	0.24	1.961E-291	NK/CD8 T cells	Cdc42ep3
3.196E-295	0.85375697	0.357	0.238	7.924E-291	NK/CD8 T cells	Ndufa5
9.999E-294	0.64623674	0.317	0.192	2.479E-289	NK/CD8 T cells	Lasp1
2.127E-293	0.59768687	0.408	0.273	5.274E-289	NK/CD8 T cells	Akna
2.772E-292	0.86024233	0.306	0.188	6.874E-288	NK/CD8 T cells	Gtf2i
3.801E-292	0.39240688	0.709	0.567	9.425E-288	NK/CD8 T cells	Atp6v1f
1.206E-291	0.50125586	0.357	0.223	2.99E-287	NK/CD8 T cells	Arrb2
1.58E-291	0.25981401	0.976	0.81	3.917E-287	NK/CD8 T cells	Rpl36
3.192E-291	0.39025957	0.835	0.739	7.915E-287	NK/CD8 T cells	Btg2
7.103E-291	0.75042051	0.373	0.249	1.761E-286	NK/CD8 T cells	Lsm5
8.731E-291	0.42945254	0.604	0.469	2.165E-286	NK/CD8 T cells	Snrnp70
6.595E-289	0.50692378	0.567	0.43	1.635E-284	NK/CD8 T cells	Atp5o.1
4.809E-288	0.3007072	0.859	0.72	1.192E-283	NK/CD8 T cells	Hnrnpa2b1
1.732E-287	0.62315406	0.47	0.343	4.294E-283	NK/CD8 T cells	Tmem160
3.681E-287	0.27944172	0.669	0.522	9.127E-283	NK/CD8 T cells	Vasp
6.623E-286	0.77922449	0.317	0.199	1.642E-281	NK/CD8 T cells	Ndfip2
2.165E-285	0.89279804	0.306	0.191	5.368E-281	NK/CD8 T cells	Cox20
1.623E-283	0.40529906	0.621	0.471	4.025E-279	NK/CD8 T cells	Tubb5
5.413E-283	0.30584341	0.876	0.722	1.342E-278	NK/CD8 T cells	Uqcrh
9.845E-282	0.53071657	0.444	0.308	2.441E-277	NK/CD8 T cells	Cd164
7.319E-281	0.78910922	0.358	0.241	1.815E-276	NK/CD8 T cells	Tbcb
1.253E-280	0.47407672	0.466	0.325	3.107E-276	NK/CD8 T cells	Srpk1
1.765E-280	0.36944098	0.752	0.604	4.375E-276	NK/CD8 T cells	Atp5b
2.438E-280	0.52730077	0.551	0.42	6.045E-276	NK/CD8 T cells	Eif3c
7.106E-280	0.83763566	0.292	0.178	1.762E-275	NK/CD8 T cells	4930453N24R
4.274E-277	0.3609096	0.724	0.585	1.06E-272	NK/CD8 T cells	Tmem50a

6.709E-275	0.45722034	0.571	0.439	1.664E-270	NK/CD8 T cells	Sec62
2.408E-274	0.67646969	0.407	0.287	5.971E-270	NK/CD8 T cells	Mrpl18
2.669E-274	0.71265489	0.398	0.279	6.619E-270	NK/CD8 T cells	Rwdd1
7.407E-273	0.39201082	0.544	0.399	1.836E-268	NK/CD8 T cells	Peli1
1.368E-272	0.50269029	0.446	0.316	3.392E-268	NK/CD8 T cells	Tgoln1
3.738E-271	0.37001232	0.641	0.504	9.27E-267	NK/CD8 T cells	Tra2b
9.496E-271	0.63482478	0.443	0.322	2.355E-266	NK/CD8 T cells	Mien1
2.323E-270	0.84371741	0.275	0.166	5.759E-266	NK/CD8 T cells	Dctn6
3.343E-270	0.289787	0.892	0.739	8.29E-266	NK/CD8 T cells	Btf3
5.945E-270	0.79080678	0.286	0.175	1.474E-265	NK/CD8 T cells	Azi2
2.335E-269	0.32540763	0.714	0.581	5.79E-265	NK/CD8 T cells	Sri
4.499E-269	0.5498864	0.51	0.382	1.116E-264	NK/CD8 T cells	Tmem258
1.048E-267	0.57338414	0.481	0.357	2.597E-263	NK/CD8 T cells	Psmb6
1.662E-265	0.61378103	0.404	0.281	4.12E-261	NK/CD8 T cells	Idnk
9.046E-265	0.94314139	0.294	0.188	2.243E-260	NK/CD8 T cells	Cdk4
1.294E-264	0.52847176	0.506	0.378	3.208E-260	NK/CD8 T cells	Pebp1
1.782E-264	0.49837743	0.512	0.384	4.42E-260	NK/CD8 T cells	Tecr
8.042E-264	0.33737528	0.845	0.695	1.994E-259	NK/CD8 T cells	Hspa8
2.628E-263	0.64485825	0.344	0.221	6.517E-259	NK/CD8 T cells	Arap2
1.321E-262	0.40133181	0.509	0.369	3.275E-258	NK/CD8 T cells	Atp11b
8.488E-262	0.66521277	0.414	0.296	2.105E-257	NK/CD8 T cells	Tomm5
3.437E-261	0.68531209	0.33	0.216	8.523E-257	NK/CD8 T cells	Tpp2
2.255E-259	0.53501885	0.505	0.38	5.591E-255	NK/CD8 T cells	Ndufb10
6.071E-259	0.49873222	0.52	0.397	1.505E-254	NK/CD8 T cells	Mrps24
5.598E-258	0.44429185	0.627	0.499	1.388E-253	NK/CD8 T cells	Taf10
1.86E-257	0.65825904	0.397	0.28	4.613E-253	NK/CD8 T cells	Hprt
3.046E-257	0.49821224	0.523	0.399	7.553E-253	NK/CD8 T cells	Sf3b5
1.104E-256	0.56442919	0.434	0.304	2.736E-252	NK/CD8 T cells	Psip1
1.711E-256	0.73541094	0.368	0.256	4.241E-252	NK/CD8 T cells	Suclg1
6.284E-256	0.86467716	0.261	0.157	1.558E-251	NK/CD8 T cells	Ppm1b
9.652E-256	0.33952675	0.727	0.576	2.393E-251	NK/CD8 T cells	Eef1d
2.35E-255	0.47371825	0.546	0.414	5.828E-251	NK/CD8 T cells	H2-T23
3.058E-251	0.39292635	0.66	0.519	7.582E-247	NK/CD8 T cells	Eif4a1
6.2E-250	0.67056246	0.334	0.22	1.537E-245	NK/CD8 T cells	Fryl
9.899E-250	0.51522842	0.463	0.338	2.454E-245	NK/CD8 T cells	Pnn
1.755E-249	0.79287693	0.336	0.228	4.351E-245	NK/CD8 T cells	Commd7
4.364E-248	0.6533444	0.33	0.213	1.082E-243	NK/CD8 T cells	S1pr4
9.153E-248	0.71388925	0.314	0.203	2.27E-243	NK/CD8 T cells	Ubac2
6.69E-247	0.51992565	0.498	0.38	1.659E-242	NK/CD8 T cells	Pdcd10
6.946E-247	0.39985663	0.658	0.529	1.722E-242	NK/CD8 T cells	Ost4
8.03E-246	0.50290562	0.513	0.391	1.991E-241	NK/CD8 T cells	Snrpd2
3.354E-245	0.48689988	0.466	0.341	8.317E-241	NK/CD8 T cells	Dock10
3.426E-244	0.40700543	0.575	0.435	8.495E-240	NK/CD8 T cells	Pdia3
7.336E-244	0.66923377	0.402	0.289	1.819E-239	NK/CD8 T cells	Pdcd5
2.588E-243	0.30853263	0.724	0.577	6.416E-239	NK/CD8 T cells	Tln1
3.611E-243	0.447815	0.578	0.45	8.953E-239	NK/CD8 T cells	Park7
1.902E-242	0.37697765	0.644	0.51	4.716E-238	NK/CD8 T cells	Snrpe
3.111E-242	0.47202519	0.44	0.306	7.715E-238	NK/CD8 T cells	Lamtor4
2.455E-241	0.59856658	0.397	0.278	6.086E-237	NK/CD8 T cells	Sash3
3.486E-241	0.58962907	0.446	0.333	8.643E-237	NK/CD8 T cells	Nt5c

8.128E-239	0.6206765	0.277	0.172	2.015E-234	NK/CD8 T cells	Rab8b
1.39E-238	0.70570976	0.29	0.18	3.446E-234	NK/CD8 T cells	Il21r
3.491E-238	0.32412843	0.716	0.593	8.655E-234	NK/CD8 T cells	Ankrd11
6.588E-238	0.61530587	0.423	0.309	1.634E-233	NK/CD8 T cells	Sugt1
2.831E-237	0.54017831	0.473	0.356	7.02E-233	NK/CD8 T cells	Cct7
3.743E-237	0.506573	0.492	0.374	9.282E-233	NK/CD8 T cells	Cwc15
3.857E-236	0.48596481	0.529	0.407	9.564E-232	NK/CD8 T cells	Uqcr10
9.327E-234	0.56181911	0.441	0.326	2.313E-229	NK/CD8 T cells	Bcas2
6.589E-233	0.49791742	0.463	0.341	1.634E-228	NK/CD8 T cells	Cbx3
1.575E-232	0.27498232	0.877	0.751	3.905E-228	NK/CD8 T cells	Pabpc1
4.852E-232	0.70930131	0.337	0.232	1.203E-227	NK/CD8 T cells	Vps29
1.245E-231	0.49634784	0.499	0.377	3.086E-227	NK/CD8 T cells	Nucks1
7.155E-231	0.52554261	0.476	0.361	1.774E-226	NK/CD8 T cells	Tsn
1.927E-230	0.37875662	0.629	0.499	4.777E-226	NK/CD8 T cells	Snrpb
2.334E-228	0.7621215	0.288	0.186	5.787E-224	NK/CD8 T cells	Ppil2
3.66E-228	0.53862941	0.436	0.321	9.074E-224	NK/CD8 T cells	Ubxn4
4.287E-228	0.670888	0.253	0.15	1.063E-223	NK/CD8 T cells	Fam102a
1.263E-227	0.37167231	0.563	0.435	3.131E-223	NK/CD8 T cells	Ikzf1
3.09E-227	0.77807617	0.318	0.214	7.661E-223	NK/CD8 T cells	Chordc1
4.098E-227	0.54345448	0.462	0.347	1.016E-222	NK/CD8 T cells	Ndufa11
8.976E-226	0.47738725	0.513	0.397	2.225E-221	NK/CD8 T cells	Bzw1
1.071E-225	0.46155457	0.492	0.367	2.656E-221	NK/CD8 T cells	Bsg
1.128E-225	0.55615714	0.319	0.208	2.796E-221	NK/CD8 T cells	Gramd1a
1.963E-225	0.72051606	0.353	0.248	4.868E-221	NK/CD8 T cells	Polr2m
1.541E-223	0.39506247	0.563	0.439	3.821E-219	NK/CD8 T cells	Srsf7
2.095E-221	0.29278854	0.706	0.581	5.194E-217	NK/CD8 T cells	Prrc2c
2.102E-221	0.29421232	0.732	0.598	5.211E-217	NK/CD8 T cells	Srsf3
2.501E-220	0.73259394	0.341	0.238	6.202E-216	NK/CD8 T cells	Ift20
4.4E-219	0.85173916	0.267	0.171	1.091E-214	NK/CD8 T cells	B3gat3
1.706E-218	0.79626102	0.264	0.167	4.23E-214	NK/CD8 T cells	Ankrd10
2.244E-218	0.48192855	0.51	0.394	5.564E-214	NK/CD8 T cells	Ndufa3
4.6E-217	0.30001124	0.773	0.636	1.141E-212	NK/CD8 T cells	Psmb1
1.282E-216	0.51732548	0.46	0.347	3.18E-212	NK/CD8 T cells	Swi5
2.195E-216	0.65933946	0.356	0.251	5.444E-212	NK/CD8 T cells	Rpp21
7.402E-216	0.44387623	0.51	0.392	1.835E-211	NK/CD8 T cells	Micos10
1.094E-215	0.3578558	0.54	0.42	2.712E-211	NK/CD8 T cells	Nktr
3.754E-215	0.60000854	0.399	0.289	9.307E-211	NK/CD8 T cells	Pole4
6.835E-215	0.6012194	0.395	0.289	1.695E-210	NK/CD8 T cells	Trappc4
1.725E-213	0.34294789	0.762	0.635	4.276E-209	NK/CD8 T cells	Eif5a
2.234E-213	0.29117673	0.788	0.668	5.539E-209	NK/CD8 T cells	Ppp1ca
9.532E-213	0.73913929	0.263	0.167	2.363E-208	NK/CD8 T cells	Xrn1
6.242E-211	0.59312735	0.342	0.237	1.548E-206	NK/CD8 T cells	Tle4
1.222E-210	0.61827928	0.396	0.292	3.029E-206	NK/CD8 T cells	Ndufs6
1.229E-210	0.76393447	0.29	0.192	3.047E-206	NK/CD8 T cells	Rbbp7
6.362E-210	0.48296061	0.437	0.323	1.577E-205	NK/CD8 T cells	Hp1bp3
8.097E-210	0.48128358	0.481	0.369	2.008E-205	NK/CD8 T cells	Atp5g3
1.496E-209	0.3204606	0.701	0.57	3.71E-205	NK/CD8 T cells	Atp5pb
2.428E-208	0.57992855	0.386	0.281	6.021E-204	NK/CD8 T cells	Sp110
8.669E-208	0.55903955	0.27	0.168	2.149E-203	NK/CD8 T cells	Ramp1
2.194E-207	0.57954741	0.39	0.285	5.44E-203	NK/CD8 T cells	Magoh

3.865E-207	0.38040522	0.587	0.469	9.584E-203	NK/CD8 T cells	Hmgb1
8.685E-207	0.41824126	0.57	0.451	2.153E-202	NK/CD8 T cells	Atp5mpl
1.625E-206	0.5752736	0.417	0.311	4.03E-202	NK/CD8 T cells	Mdh1
6.228E-206	0.30990443	0.687	0.559	1.544E-201	NK/CD8 T cells	Hnrnpa3
7.753E-202	0.5332342	0.357	0.251	1.922E-197	NK/CD8 T cells	Tap2
2.02E-201	0.50132014	0.458	0.352	5.009E-197	NK/CD8 T cells	Ndufs7
2.894E-201	0.66789531	0.333	0.234	7.176E-197	NK/CD8 T cells	Denr
3.842E-201	0.28793673	0.717	0.579	9.527E-197	NK/CD8 T cells	Capzb
5.65E-201	0.42520226	0.527	0.413	1.401E-196	NK/CD8 T cells	Raly
1.057E-200	0.57307436	0.395	0.29	2.622E-196	NK/CD8 T cells	Smc3
1.478E-200	0.69559579	0.266	0.172	3.666E-196	NK/CD8 T cells	Ss18
3.983E-200	0.45396819	0.303	0.198	9.877E-196	NK/CD8 T cells	Esyt2
4.584E-200	0.36507559	0.525	0.404	1.137E-195	NK/CD8 T cells	Itgb7
7.506E-200	0.45505851	0.473	0.365	1.861E-195	NK/CD8 T cells	Tmem167
1.378E-199	0.53532128	0.435	0.33	3.417E-195	NK/CD8 T cells	Ppig
3.849E-199	0.41687201	0.518	0.406	9.542E-195	NK/CD8 T cells	Xrn2
4.124E-197	0.42834795	0.34	0.231	1.023E-192	NK/CD8 T cells	Heca
4.406E-196	0.73621006	0.262	0.17	1.092E-191	NK/CD8 T cells	Gatad2a
2.718E-195	0.38986818	0.452	0.339	6.739E-191	NK/CD8 T cells	Cox17
8.43E-195	0.32476807	0.678	0.549	2.09E-190	NK/CD8 T cells	Atp5a1
1.319E-194	0.50990563	0.424	0.317	3.269E-190	NK/CD8 T cells	Arf6
3.481E-194	0.74926199	0.252	0.16	8.63E-190	NK/CD8 T cells	Nmi
4.269E-194	0.67074248	0.305	0.209	1.058E-189	NK/CD8 T cells	Sap30l
4.583E-194	0.68774557	0.34	0.245	1.136E-189	NK/CD8 T cells	Cenpx
2.092E-193	0.52829612	0.439	0.336	5.187E-189	NK/CD8 T cells	Bax
7.082E-193	0.84784792	0.258	0.17	1.756E-188	NK/CD8 T cells	Mrps36
7.18E-193	0.5581925	0.398	0.297	1.78E-188	NK/CD8 T cells	Csnk2b
2.717E-191	0.25307129	0.707	0.584	6.737E-187	NK/CD8 T cells	Gm8995
5.867E-191	0.56988182	0.387	0.287	1.455E-186	NK/CD8 T cells	Higd2a
8.974E-191	0.31010732	0.62	0.505	2.225E-186	NK/CD8 T cells	Tsc22d4
9.41E-191	0.60466394	0.35	0.251	2.333E-186	NK/CD8 T cells	Rdx
1.137E-190	0.4740467	0.437	0.331	2.819E-186	NK/CD8 T cells	Spcs1
2.393E-190	0.32639931	0.488	0.371	5.933E-186	NK/CD8 T cells	Wipf1
6.153E-190	0.58726349	0.385	0.285	1.526E-185	NK/CD8 T cells	Snrpf
7.096E-190	0.63598789	0.332	0.235	1.759E-185	NK/CD8 T cells	Lsm8
1.589E-189	0.64626725	0.355	0.259	3.94E-185	NK/CD8 T cells	Psmc3
1.493E-188	0.44957112	0.416	0.31	3.702E-184	NK/CD8 T cells	Cast
5.001E-187	0.36953971	0.578	0.471	1.24E-182	NK/CD8 T cells	Trir
6.465E-187	0.63029156	0.301	0.207	1.603E-182	NK/CD8 T cells	Dap
9.902E-187	0.54793838	0.429	0.331	2.455E-182	NK/CD8 T cells	Cdc37
3.283E-186	0.55542933	0.321	0.223	8.141E-182	NK/CD8 T cells	Asxl2
3.93E-185	0.60132287	0.392	0.292	9.744E-181	NK/CD8 T cells	Slc25a4
5.712E-185	0.33843136	0.648	0.526	1.416E-180	NK/CD8 T cells	Arhgdia
9.492E-185	0.53614731	0.388	0.288	2.353E-180	NK/CD8 T cells	Tram1
1.347E-184	0.7147232	0.294	0.203	3.339E-180	NK/CD8 T cells	Svbp
1.491E-184	0.73562077	0.267	0.178	3.697E-180	NK/CD8 T cells	Tiprl
1.559E-184	0.46571811	0.451	0.348	3.865E-180	NK/CD8 T cells	Pura
3.561E-184	0.62648041	0.351	0.254	8.828E-180	NK/CD8 T cells	Ssrp1
6.806E-184	0.3245224	0.605	0.493	1.688E-179	NK/CD8 T cells	Pafah1b1
4.398E-183	0.59952786	0.388	0.291	1.091E-178	NK/CD8 T cells	Hspd1

4.907E-183	0.35894988	0.571	0.454	1.217E-178	NK/CD8 T cells	Selenof
5.002E-182	0.34495987	0.576	0.465	1.24E-177	NK/CD8 T cells	Sf3b6
6.14E-182	0.30999653	0.602	0.49	1.523E-177	NK/CD8 T cells	Wnk1
3.327E-181	0.32546101	0.59	0.477	8.249E-177	NK/CD8 T cells	Psma3
3.708E-181	0.33442577	0.588	0.479	9.195E-177	NK/CD8 T cells	Csnk1a1
2.931E-180	0.59768312	0.359	0.263	7.269E-176	NK/CD8 T cells	Abcf1
4.154E-180	0.44137212	0.46	0.347	1.03E-175	NK/CD8 T cells	Rps27l
4.197E-180	0.29051194	0.724	0.597	1.041E-175	NK/CD8 T cells	Rpl13a
9.06E-180	0.40872756	0.463	0.36	2.247E-175	NK/CD8 T cells	Prpf4b
1.104E-178	0.62915148	0.332	0.238	2.738E-174	NK/CD8 T cells	Tbrg1
4.358E-178	0.25730654	0.716	0.572	1.081E-173	NK/CD8 T cells	Hint1
8.132E-178	0.63471684	0.311	0.218	2.016E-173	NK/CD8 T cells	Maz
1.851E-177	0.58292704	0.369	0.275	4.589E-173	NK/CD8 T cells	Srp19
4E-177	0.38461715	0.473	0.367	9.918E-173	NK/CD8 T cells	Rab8a
6.283E-177	0.36352183	0.518	0.408	1.558E-172	NK/CD8 T cells	Tacc1
2.274E-175	0.29160911	0.677	0.565	5.639E-171	NK/CD8 T cells	Eif4g2
2.342E-175	0.71406304	0.317	0.229	5.807E-171	NK/CD8 T cells	Uqcc2
5.413E-175	0.58934065	0.37	0.277	1.342E-170	NK/CD8 T cells	Polr2f
6.436E-175	0.67179743	0.321	0.23	1.596E-170	NK/CD8 T cells	Prmt1
7.34E-175	0.38903033	0.537	0.427	1.82E-170	NK/CD8 T cells	Lamtor2
1.1E-174	0.27395664	0.719	0.598	2.728E-170	NK/CD8 T cells	Hnrnpa0
3.565E-173	0.57973485	0.251	0.162	8.839E-169	NK/CD8 T cells	Bcl2l11
7.741E-172	0.47618093	0.429	0.331	1.919E-167	NK/CD8 T cells	BC031181
4.101E-171	0.53026082	0.414	0.318	1.017E-166	NK/CD8 T cells	Mrpl52
1.766E-170	0.31571471	0.613	0.508	4.38E-166	NK/CD8 T cells	Rock1
3.815E-170	0.35254207	0.539	0.422	9.459E-166	NK/CD8 T cells	Smdt1
2.422E-169	0.32388395	0.577	0.465	6.005E-165	NK/CD8 T cells	Tmed2
2.773E-169	0.46484444	0.457	0.359	6.875E-165	NK/CD8 T cells	Trmt112
6.745E-169	0.39656333	0.496	0.394	1.672E-164	NK/CD8 T cells	Ndufb5
1.282E-168	0.73840108	0.265	0.18	3.179E-164	NK/CD8 T cells	Exosc8
1.626E-168	0.50328768	0.373	0.276	4.031E-164	NK/CD8 T cells	Hspa14
1.898E-168	0.26355502	0.36	0.244	4.706E-164	NK/CD8 T cells	Il7r
3.329E-168	0.36991219	0.528	0.426	8.253E-164	NK/CD8 T cells	Rab2a
5.361E-168	0.45496848	0.442	0.345	1.329E-163	NK/CD8 T cells	Ndufb3
6.707E-168	0.256203	0.372	0.261	1.663E-163	NK/CD8 T cells	Lcp2
1.008E-167	0.43949795	0.374	0.275	2.501E-163	NK/CD8 T cells	Cyld
5.957E-167	0.60285073	0.281	0.193	1.477E-162	NK/CD8 T cells	Pcna
1.987E-166	0.52349759	0.384	0.291	4.926E-162	NK/CD8 T cells	Cmpk1
4.126E-166	0.58892749	0.286	0.198	1.023E-161	NK/CD8 T cells	Itm2c
7.404E-166	0.35020784	0.554	0.451	1.836E-161	NK/CD8 T cells	Ubxn1
8.81E-166	0.42873983	0.426	0.329	2.185E-161	NK/CD8 T cells	Pdcd6
1.358E-165	0.62069985	0.335	0.247	3.366E-161	NK/CD8 T cells	Psmc2
1.574E-165	0.40251487	0.483	0.384	3.902E-161	NK/CD8 T cells	Emg1
1.981E-165	0.51494237	0.25	0.159	4.913E-161	NK/CD8 T cells	Lamb3
1.562E-164	0.34962683	0.357	0.253	3.873E-160	NK/CD8 T cells	Tm6sf1
1.563E-162	0.67174262	0.25	0.168	3.874E-158	NK/CD8 T cells	Tmem9b
1.844E-162	0.69542432	0.298	0.213	4.572E-158	NK/CD8 T cells	Pfdn1
3.042E-162	0.44225359	0.464	0.367	7.542E-158	NK/CD8 T cells	Cct8
4.451E-162	0.48539893	0.387	0.292	1.104E-157	NK/CD8 T cells	Ppm1g
6.639E-162	0.25239273	0.677	0.558	1.646E-157	NK/CD8 T cells	Tpr

2.563E-161	0.55512815	0.36	0.271	6.355E-157	NK/CD8 T cells	Ndufs4
8.335E-160	0.63917255	0.295	0.208	2.067E-155	NK/CD8 T cells	Aip
1.191E-159	0.4187611	0.457	0.361	2.954E-155	NK/CD8 T cells	Plekhj1
1.795E-159	0.45017049	0.426	0.331	4.45E-155	NK/CD8 T cells	Ddx39b
1.604E-158	0.35691107	0.374	0.275	3.978E-154	NK/CD8 T cells	Zdhhc20
3.593E-157	0.62181895	0.31	0.225	8.909E-153	NK/CD8 T cells	Gspt1
3.318E-156	0.44077011	0.443	0.349	8.226E-152	NK/CD8 T cells	Sarnp
1.791E-155	0.73358934	0.288	0.206	4.441E-151	NK/CD8 T cells	Stip1
1.016E-154	0.59990505	0.319	0.233	2.519E-150	NK/CD8 T cells	Sars
1.059E-154	0.41467721	0.422	0.327	2.625E-150	NK/CD8 T cells	Dctn3
1.404E-154	0.41977859	0.447	0.347	3.482E-150	NK/CD8 T cells	Uqcr11
3.082E-154	0.36956926	0.479	0.381	7.641E-150	NK/CD8 T cells	G3bp2
4.628E-154	0.53689197	0.286	0.2	1.147E-149	NK/CD8 T cells	Aebp2
1.097E-153	0.52953366	0.337	0.25	2.721E-149	NK/CD8 T cells	Dguok
1.129E-153	0.3816131	0.501	0.402	2.8E-149	NK/CD8 T cells	Psmb4
1.519E-153	0.40093278	0.495	0.4	3.767E-149	NK/CD8 T cells	Arf4
4.385E-153	0.54001144	0.371	0.285	1.087E-148	NK/CD8 T cells	Cct6a
5.343E-153	0.53941427	0.373	0.286	1.325E-148	NK/CD8 T cells	Akr1b3
5.631E-153	0.44141227	0.404	0.311	1.396E-148	NK/CD8 T cells	Puf60
1.032E-152	0.42827023	0.436	0.343	2.558E-148	NK/CD8 T cells	Snrpd3
2.355E-152	0.4240437	0.422	0.329	5.84E-148	NK/CD8 T cells	Rbbp4
3.859E-152	0.45416695	0.272	0.183	9.568E-148	NK/CD8 T cells	Rasgrp1
6.19E-151	0.41905611	0.437	0.347	1.535E-146	NK/CD8 T cells	Pfdn2
6.409E-151	0.44986592	0.318	0.225	1.589E-146	NK/CD8 T cells	Sesn3
3.898E-150	0.39516782	0.493	0.398	9.665E-146	NK/CD8 T cells	Dnajc8
6.299E-150	0.37769749	0.499	0.405	1.562E-145	NK/CD8 T cells	Nop10
5.147E-149	0.62060029	0.316	0.233	1.276E-144	NK/CD8 T cells	Rsl1d1
7.51E-149	0.63582899	0.264	0.182	1.862E-144	NK/CD8 T cells	Lsm1
7.962E-149	0.64935355	0.288	0.207	1.974E-144	NK/CD8 T cells	Mrps26
8.135E-148	0.49890313	0.393	0.307	2.017E-143	NK/CD8 T cells	Tcp1
1.695E-147	0.45006875	0.4	0.31	4.203E-143	NK/CD8 T cells	Fkbp8
3.066E-147	0.48212318	0.396	0.307	7.603E-143	NK/CD8 T cells	Cops9
8.175E-146	0.57598029	0.326	0.243	2.027E-141	NK/CD8 T cells	Txnl1
4.632E-145	0.68619719	0.266	0.187	1.149E-140	NK/CD8 T cells	Fundc2
1.838E-144	0.27062279	0.566	0.468	4.558E-140	NK/CD8 T cells	Dock2
5.667E-144	0.47314437	0.399	0.311	1.405E-139	NK/CD8 T cells	Txndc17
2.281E-143	0.47990813	0.379	0.293	5.655E-139	NK/CD8 T cells	Eif1b
2.314E-143	0.53622391	0.348	0.264	5.739E-139	NK/CD8 T cells	Mrpl57
3.345E-143	0.57072726	0.36	0.277	8.295E-139	NK/CD8 T cells	Cycs
1.048E-142	0.56150686	0.296	0.214	2.597E-138	NK/CD8 T cells	Nudcd3
3.158E-142	0.41932173	0.371	0.282	7.831E-138	NK/CD8 T cells	Lrp10
6.609E-142	0.56081814	0.319	0.238	1.639E-137	NK/CD8 T cells	Eny2
1.185E-141	0.47434298	0.329	0.238	2.938E-137	NK/CD8 T cells	Gramd3
7.717E-141	0.36819065	0.421	0.326	1.913E-136	NK/CD8 T cells	Sipa1
3.434E-140	0.52180717	0.355	0.272	8.514E-136	NK/CD8 T cells	Sptssa
5.458E-140	0.26419998	0.571	0.466	1.353E-135	NK/CD8 T cells	Ndufb9
2.857E-138	0.42353945	0.359	0.271	7.085E-134	NK/CD8 T cells	Nptn
2.993E-138	0.3909668	0.464	0.375	7.42E-134	NK/CD8 T cells	Dnaja2
4.19E-138	0.38991316	0.448	0.356	1.039E-133	NK/CD8 T cells	Ube2l3
8.581E-138	0.38121684	0.412	0.321	2.128E-133	NK/CD8 T cells	Mat2b

1.458E-137	0.58568401	0.252	0.175	3.615E-133	NK/CD8 T cells	Riok1
6.837E-137	0.49025298	0.386	0.304	1.695E-132	NK/CD8 T cells	Psmb5
7.21E-136	0.31348163	0.521	0.428	1.788E-131	NK/CD8 T cells	Srp9
1.151E-135	0.36200463	0.498	0.403	2.855E-131	NK/CD8 T cells	Gm11478
1.241E-135	0.47190072	0.339	0.254	3.077E-131	NK/CD8 T cells	Csnk1g2
2.295E-135	0.48109359	0.388	0.303	5.69E-131	NK/CD8 T cells	Ranbp1
2.889E-135	0.42519185	0.421	0.334	7.163E-131	NK/CD8 T cells	Pbrm1
5.256E-135	0.29607624	0.311	0.22	1.303E-130	NK/CD8 T cells	Pstpip1
8.01E-134	0.42031106	0.39	0.302	1.986E-129	NK/CD8 T cells	Zbtb7a
1.191E-133	0.51667344	0.276	0.196	2.952E-129	NK/CD8 T cells	Dgkz
2.637E-133	0.32011197	0.401	0.306	6.538E-129	NK/CD8 T cells	Tab2
6.929E-133	0.38353565	0.415	0.326	1.718E-128	NK/CD8 T cells	Esd
3.416E-132	0.47141768	0.381	0.298	8.469E-128	NK/CD8 T cells	Zc3h15
4.143E-132	0.38250981	0.466	0.379	1.027E-127	NK/CD8 T cells	Timm13
1.295E-131	0.44827927	0.325	0.241	3.21E-127	NK/CD8 T cells	Cntrl
1.615E-131	0.39006069	0.45	0.362	4.003E-127	NK/CD8 T cells	Rtraf
2.397E-131	0.39848475	0.27	0.184	5.943E-127	NK/CD8 T cells	Ppp1r16b
7.254E-131	0.51803995	0.347	0.269	1.799E-126	NK/CD8 T cells	Timm23
7.569E-130	0.61319191	0.292	0.217	1.877E-125	NK/CD8 T cells	Anapc11
4.131E-129	0.42069485	0.397	0.314	1.024E-124	NK/CD8 T cells	Eloc
4.274E-129	0.47534102	0.373	0.292	1.06E-124	NK/CD8 T cells	Ube2m
1.32E-128	0.39002248	0.444	0.36	3.274E-124	NK/CD8 T cells	Micos13
2.704E-128	0.65674212	0.259	0.187	6.704E-124	NK/CD8 T cells	Psmd14
3.295E-127	0.55501078	0.272	0.197	8.169E-123	NK/CD8 T cells	Synj2bp
6.064E-127	0.58338132	0.295	0.22	1.504E-122	NK/CD8 T cells	Rex1bd
8.541E-127	0.3572087	0.473	0.386	2.118E-122	NK/CD8 T cells	Psmd4
4.109E-126	0.561835	0.269	0.194	1.019E-121	NK/CD8 T cells	Nucb1
7.783E-126	0.61638888	0.281	0.208	1.93E-121	NK/CD8 T cells	Ebna1bp2
9.753E-126	0.54240451	0.301	0.223	2.418E-121	NK/CD8 T cells	1110008P14R
1.961E-125	0.40469179	0.4	0.317	4.862E-121	NK/CD8 T cells	Ndufa8
4.207E-125	0.59039229	0.28	0.205	1.043E-120	NK/CD8 T cells	Casp8
7.058E-125	0.40998958	0.29	0.209	1.75E-120	NK/CD8 T cells	Cblb
7.181E-125	0.49820677	0.369	0.291	1.781E-120	NK/CD8 T cells	Hdgf
1.107E-124	0.41131005	0.373	0.292	2.744E-120	NK/CD8 T cells	Ube3a
1.566E-124	0.3440269	0.495	0.407	3.884E-120	NK/CD8 T cells	Eif3i
2.754E-124	0.36008401	0.483	0.397	6.829E-120	NK/CD8 T cells	Hnrnpd
3.34E-124	0.2844888	0.525	0.435	8.282E-120	NK/CD8 T cells	Ddx24
4.261E-124	0.52933951	0.345	0.268	1.057E-119	NK/CD8 T cells	Ndufc1
3.328E-123	0.57922781	0.288	0.215	8.252E-119	NK/CD8 T cells	Mlf2
8.973E-123	0.3118182	0.527	0.438	2.225E-118	NK/CD8 T cells	Eif3m
3.01E-122	0.45185349	0.4	0.321	7.464E-118	NK/CD8 T cells	Cacybp
3.966E-122	0.25031501	0.555	0.463	9.834E-118	NK/CD8 T cells	Top1
5.883E-122	0.42517354	0.271	0.194	1.459E-117	NK/CD8 T cells	Stim1
2.7E-121	0.53166083	0.316	0.241	6.694E-117	NK/CD8 T cells	Ociad1
2.749E-121	0.53655281	0.274	0.199	6.817E-117	NK/CD8 T cells	Os9
2.854E-121	0.52228337	0.289	0.214	7.076E-117	NK/CD8 T cells	Stag1
5.635E-121	0.44277048	0.306	0.227	1.397E-116	NK/CD8 T cells	Trip11
6.529E-121	0.40460837	0.418	0.337	1.619E-116	NK/CD8 T cells	Snu13
1.356E-120	0.65781212	0.272	0.202	3.361E-116	NK/CD8 T cells	Ndufb2
8.623E-120	0.41789718	0.373	0.294	2.138E-115	NK/CD8 T cells	Hnrnpl

8.955E-120	0.35006247	0.47	0.38	2.22E-115	NK/CD8 T cells	Eno1
1.007E-119	0.29781614	0.476	0.386	2.498E-115	NK/CD8 T cells	Kmt2a
2.8E-119	0.58366364	0.282	0.21	6.942E-115	NK/CD8 T cells	Glrx5
6.654E-119	0.3356057	0.462	0.38	1.65E-114	NK/CD8 T cells	Bcap31
1.062E-118	0.47253635	0.329	0.254	2.633E-114	NK/CD8 T cells	Bub3
1.217E-118	0.36782999	0.464	0.383	3.018E-114	NK/CD8 T cells	Ptges3
1.225E-118	0.28376414	0.543	0.454	3.038E-114	NK/CD8 T cells	Tomm20
1.496E-118	0.42810142	0.273	0.195	3.709E-114	NK/CD8 T cells	Epb41
4.057E-118	0.48262151	0.369	0.293	1.006E-113	NK/CD8 T cells	1110004F10Ri
9.437E-118	0.6028318	0.267	0.196	2.34E-113	NK/CD8 T cells	Dpy30
3.017E-117	0.55961063	0.309	0.237	7.481E-113	NK/CD8 T cells	Coa3
5.367E-117	0.35336136	0.478	0.394	1.331E-112	NK/CD8 T cells	Hnrnpab
6.106E-117	0.26708356	0.605	0.515	1.514E-112	NK/CD8 T cells	Tpm3
6.112E-117	0.52920002	0.3	0.227	1.515E-112	NK/CD8 T cells	Smim11
7.323E-117	0.52689055	0.259	0.187	1.816E-112	NK/CD8 T cells	Rps6ka1
1.5E-116	0.42098673	0.369	0.289	3.719E-112	NK/CD8 T cells	Ubtf
1.615E-116	0.29013707	0.54	0.447	4.004E-112	NK/CD8 T cells	Hspe1
3.253E-116	0.53920347	0.263	0.191	8.066E-112	NK/CD8 T cells	Ski
4.304E-116	0.47343838	0.253	0.18	1.067E-111	NK/CD8 T cells	Vps13a
1.59E-115	0.32802535	0.425	0.342	3.942E-111	NK/CD8 T cells	Gsk3b
2.203E-115	0.50598557	0.289	0.216	5.462E-111	NK/CD8 T cells	Actn4
6.122E-115	0.47199962	0.323	0.245	1.518E-110	NK/CD8 T cells	Dnajb1
2.015E-114	0.57485982	0.301	0.232	4.995E-110	NK/CD8 T cells	Glrx3
2.874E-113	0.53904115	0.279	0.209	7.126E-109	NK/CD8 T cells	Hadhb
5.311E-113	0.49464652	0.332	0.258	1.317E-108	NK/CD8 T cells	Stub1
1.263E-112	0.46630849	0.302	0.23	3.132E-108	NK/CD8 T cells	Tmem128
1.822E-112	0.36788873	0.423	0.342	4.517E-108	NK/CD8 T cells	Psmb2
5.266E-112	0.50648692	0.302	0.23	1.306E-107	NK/CD8 T cells	Aurkaip1
5.749E-112	0.4604182	0.359	0.286	1.425E-107	NK/CD8 T cells	Eif6
5.805E-112	0.48514988	0.303	0.23	1.439E-107	NK/CD8 T cells	Zranb2
6.316E-112	0.45012999	0.36	0.286	1.566E-107	NK/CD8 T cells	Snrpd1
2.321E-111	0.56886583	0.254	0.185	5.755E-107	NK/CD8 T cells	Pgp
4.412E-111	0.3844293	0.402	0.324	1.094E-106	NK/CD8 T cells	Psma4
1.179E-110	0.30749431	0.366	0.287	2.923E-106	NK/CD8 T cells	Tmem30a
1.842E-110	0.31264053	0.287	0.209	4.566E-106	NK/CD8 T cells	Nfatc1
1.866E-110	0.33862843	0.443	0.359	4.627E-106	NK/CD8 T cells	Ndufb8
1.968E-110	0.46688691	0.315	0.241	4.88E-106	NK/CD8 T cells	Snrnp48
5.283E-110	0.52925751	0.312	0.241	1.31E-105	NK/CD8 T cells	Pdap1
8.103E-110	0.48997733	0.291	0.222	2.009E-105	NK/CD8 T cells	Ebp
8.293E-110	0.47129066	0.31	0.24	2.056E-105	NK/CD8 T cells	Syncrip
1.241E-109	0.62661479	0.266	0.2	3.078E-105	NK/CD8 T cells	Ciao2b
1.248E-109	0.45895433	0.355	0.281	3.093E-105	NK/CD8 T cells	Cope
1.194E-108	0.43014827	0.37	0.295	2.961E-104	NK/CD8 T cells	Cct3
2.32E-108	0.35362303	0.434	0.354	5.752E-104	NK/CD8 T cells	Prelid1
2.484E-108	0.38197617	0.342	0.266	6.159E-104	NK/CD8 T cells	Fip1l1
3.525E-108	0.47332731	0.327	0.257	8.741E-104	NK/CD8 T cells	Ndufa10
1.907E-107	0.34493034	0.448	0.369	4.728E-103	NK/CD8 T cells	Rtf1
3.176E-107	0.32968714	0.33	0.25	7.874E-103	NK/CD8 T cells	Kif21b
5.214E-107	0.41046134	0.376	0.302	1.293E-102	NK/CD8 T cells	Imp3
8.223E-107	0.36630741	0.367	0.29	2.039E-102	NK/CD8 T cells	Slc50a1

1.04E-106	0.46840079	0.339	0.268	2.579E-102	NK/CD8 T cells	Zcrb1
1.574E-106	0.40186919	0.308	0.233	3.903E-102	NK/CD8 T cells	Sh3gl1
2.002E-106	0.53931916	0.274	0.205	4.965E-102	NK/CD8 T cells	Ngdn
2.375E-106	0.39132961	0.357	0.276	5.889E-102	NK/CD8 T cells	Dnajc9
2.663E-106	0.50918277	0.287	0.219	6.602E-102	NK/CD8 T cells	Ntan1
5.486E-106	0.58861238	0.272	0.207	1.36E-101	NK/CD8 T cells	Ndufb6
9.319E-106	0.45036852	0.347	0.276	2.311E-101	NK/CD8 T cells	Trappc6b
1.089E-105	0.3896137	0.367	0.292	2.7E-101	NK/CD8 T cells	Hnrnpr
2.619E-105	0.54853299	0.297	0.229	6.495E-101	NK/CD8 T cells	Mbd3
2.93E-105	0.56274319	0.261	0.194	7.264E-101	NK/CD8 T cells	Hdac1
3.191E-105	0.26822949	0.4	0.318	7.913E-101	NK/CD8 T cells	Zc3hav1
7.114E-105	0.40733816	0.363	0.29	1.764E-100	NK/CD8 T cells	Rheb
1.044E-104	0.43717568	0.361	0.289	2.589E-100	NK/CD8 T cells	U2surp
1.959E-104	0.34257238	0.426	0.35	4.857E-100	NK/CD8 T cells	Nono
1.09E-103	0.31430115	0.509	0.426	2.703E-99	NK/CD8 T cells	Snx3
1.156E-103	0.25906924	0.545	0.458	2.867E-99	NK/CD8 T cells	Eif3a
8.674E-103	0.57218772	0.28	0.214	2.1508E-98	NK/CD8 T cells	Commd4
1.128E-102	0.26378637	0.508	0.426	2.7979E-98	NK/CD8 T cells	Arpp19
5.573E-102	0.60384268	0.276	0.213	1.3818E-97	NK/CD8 T cells	Cops6
6.555E-102	0.41094079	0.334	0.264	1.6252E-97	NK/CD8 T cells	Dync1i2
9.481E-102	0.31103491	0.397	0.32	2.3509E-97	NK/CD8 T cells	Ggnbp2
2.406E-101	0.45368801	0.341	0.271	5.9647E-97	NK/CD8 T cells	Dcun1d5
1.493E-100	0.37544764	0.304	0.232	3.7017E-96	NK/CD8 T cells	Tap1
2.057E-100	0.51613994	0.274	0.208	5.101E-96	NK/CD8 T cells	Ccdc59
2.213E-100	0.3743789	0.277	0.206	5.486E-96	NK/CD8 T cells	Mob3a
4.139E-100	0.49838464	0.31	0.243	1.0262E-95	NK/CD8 T cells	Cyc1
2.004E-99	0.34613147	0.37	0.297	4.9694E-95	NK/CD8 T cells	Ewsr1
2.795E-99	0.58028347	0.267	0.203	6.9303E-95	NK/CD8 T cells	Glmp
6.197E-99	0.43220512	0.358	0.288	1.5365E-94	NK/CD8 T cells	Banf1
8.912E-99	0.44056625	0.353	0.285	2.2096E-94	NK/CD8 T cells	Mrps33
1.1184E-98	0.44870496	0.335	0.267	2.7731E-94	NK/CD8 T cells	U2af1
2.0093E-98	0.35389611	0.426	0.353	4.9822E-94	NK/CD8 T cells	Sh3kbp1
5.3933E-98	0.45001071	0.313	0.246	1.3373E-93	NK/CD8 T cells	Ssr2
1.6255E-97	0.51527972	0.318	0.253	4.0304E-93	NK/CD8 T cells	Txn2
1.685E-97	0.31345215	0.469	0.394	4.178E-93	NK/CD8 T cells	Lsm4
6.882E-97	0.47331371	0.296	0.229	1.7064E-92	NK/CD8 T cells	Thoc7
1.7852E-96	0.41949896	0.297	0.228	4.4263E-92	NK/CD8 T cells	Klhl6
7.871E-96	0.31675205	0.441	0.367	1.9516E-91	NK/CD8 T cells	G3bp1
1.1113E-93	0.37356796	0.301	0.231	2.7554E-89	NK/CD8 T cells	Skil
1.4285E-93	0.53421873	0.277	0.214	3.542E-89	NK/CD8 T cells	Alyref
1.5196E-93	0.43954962	0.296	0.23	3.7678E-89	NK/CD8 T cells	Srpr
2.7616E-93	0.25371023	0.3	0.228	6.8474E-89	NK/CD8 T cells	4932438A13R
2.9928E-93	0.36578584	0.316	0.246	7.4207E-89	NK/CD8 T cells	Plgrkt
3.6787E-93	0.29238626	0.303	0.231	9.1214E-89	NK/CD8 T cells	Dennd1b
4.7709E-93	0.38889357	0.372	0.303	1.183E-88	NK/CD8 T cells	Polr2e
1.6147E-92	0.35535612	0.358	0.287	4.0036E-88	NK/CD8 T cells	Aprt
1.8299E-92	0.5063605	0.282	0.22	4.5373E-88	NK/CD8 T cells	Copb2
2.3128E-92	0.42062031	0.319	0.252	5.7345E-88	NK/CD8 T cells	Nap1l4
3.443E-92	0.42546436	0.328	0.264	8.5369E-88	NK/CD8 T cells	Ddrgk1
3.7078E-92	0.27549071	0.502	0.427	9.1936E-88	NK/CD8 T cells	Cct2

4.4824E-92	0.55044244	0.253	0.191	1.1114E-87	NK/CD8 T cells	Zcchc9
6.2803E-92	0.51656956	0.275	0.212	1.5572E-87	NK/CD8 T cells	Psmd12
7.9409E-92	0.27821383	0.43	0.357	1.969E-87	NK/CD8 T cells	Srsf2
8.7593E-92	0.32249654	0.42	0.35	2.1719E-87	NK/CD8 T cells	Tomm22
1.2969E-91	0.53051255	0.284	0.222	3.2156E-87	NK/CD8 T cells	Ssna1
1.6753E-91	0.42446648	0.316	0.25	4.1539E-87	NK/CD8 T cells	Pop5
1.9608E-91	0.32741072	0.291	0.22	4.8618E-87	NK/CD8 T cells	Sipa1l1
5.3849E-91	0.45383665	0.332	0.269	1.3352E-86	NK/CD8 T cells	Metap2
1.1994E-90	0.37303818	0.323	0.255	2.9738E-86	NK/CD8 T cells	Usp25
1.9988E-90	0.42870862	0.343	0.278	4.956E-86	NK/CD8 T cells	Lsm6
3.1266E-90	0.59156901	0.258	0.198	7.7524E-86	NK/CD8 T cells	Ndufaf8
3.4223E-90	0.34784271	0.389	0.322	8.4856E-86	NK/CD8 T cells	Rab10
3.7892E-90	0.48502311	0.278	0.216	9.3953E-86	NK/CD8 T cells	Rab1b
1.4227E-89	0.40016225	0.304	0.239	3.5277E-85	NK/CD8 T cells	Dhx36
3.5503E-89	0.51484506	0.288	0.228	8.803E-85	NK/CD8 T cells	Psmc4
5.1107E-89	0.37028785	0.372	0.307	1.2672E-84	NK/CD8 T cells	Emc10
6.3568E-89	0.4261041	0.32	0.255	1.5762E-84	NK/CD8 T cells	Eif4e2
8.7443E-89	0.4775335	0.298	0.236	2.1681E-84	NK/CD8 T cells	Eif2s1
1.0418E-88	0.52953004	0.263	0.203	2.5831E-84	NK/CD8 T cells	Tex261
1.0811E-88	0.54301589	0.255	0.196	2.6805E-84	NK/CD8 T cells	Polr2c
1.3993E-88	0.42701531	0.327	0.264	3.4696E-84	NK/CD8 T cells	Rnf7
2.5226E-88	0.4076687	0.362	0.298	6.2547E-84	NK/CD8 T cells	Uqcrc2
5.6927E-88	0.34673957	0.384	0.315	1.4115E-83	NK/CD8 T cells	Ap2s1
6.2761E-88	0.48209772	0.304	0.241	1.5561E-83	NK/CD8 T cells	Eif4a3
1.7715E-87	0.42198514	0.295	0.229	4.3923E-83	NK/CD8 T cells	Ddost
2.2723E-87	0.36112248	0.304	0.239	5.6343E-83	NK/CD8 T cells	Phf14
4.5419E-87	0.3408836	0.387	0.319	1.1262E-82	NK/CD8 T cells	Smarca5
5.1998E-87	0.42347798	0.315	0.251	1.2893E-82	NK/CD8 T cells	Rnaset2a
5.7995E-87	0.34662441	0.394	0.327	1.438E-82	NK/CD8 T cells	Map2k2
1.3061E-86	0.27191231	0.363	0.294	3.2384E-82	NK/CD8 T cells	Zfp644
1.4422E-86	0.33433999	0.269	0.203	3.576E-82	NK/CD8 T cells	Zfp652
6.9528E-86	0.49152686	0.267	0.207	1.7239E-81	NK/CD8 T cells	Ccdc124
8.407E-86	0.26074028	0.34	0.27	2.0845E-81	NK/CD8 T cells	Cab39
1.1933E-85	0.32599698	0.37	0.304	2.9588E-81	NK/CD8 T cells	Gtf2h5
1.5753E-85	0.53322363	0.263	0.204	3.906E-81	NK/CD8 T cells	Snrpa1
1.7194E-85	0.47090595	0.273	0.213	4.2631E-81	NK/CD8 T cells	Mrpl32
2.4584E-85	0.52050498	0.274	0.215	6.0955E-81	NK/CD8 T cells	Eif1ax
4.9266E-85	0.43348169	0.289	0.225	1.2216E-80	NK/CD8 T cells	Selenoh
7.0111E-85	0.37195124	0.353	0.288	1.7384E-80	NK/CD8 T cells	Zmat2
1.2308E-84	0.43846764	0.311	0.248	3.0519E-80	NK/CD8 T cells	Trp53
2.9384E-84	0.25718891	0.466	0.39	7.2858E-80	NK/CD8 T cells	Gnb1
3.3249E-84	0.46989608	0.278	0.216	8.2442E-80	NK/CD8 T cells	Zc3h13
3.6641E-84	0.48608785	0.303	0.244	9.0852E-80	NK/CD8 T cells	Romo1
3.929E-84	0.559504	0.264	0.207	9.742E-80	NK/CD8 T cells	Etfb
6.1448E-84	0.42242279	0.315	0.253	1.5236E-79	NK/CD8 T cells	Cetn3
8.7808E-84	0.31914436	0.376	0.309	2.1772E-79	NK/CD8 T cells	Thrap3
1.2081E-83	0.33751351	0.363	0.298	2.9954E-79	NK/CD8 T cells	Ier3ip1
1.2707E-83	0.53983304	0.265	0.207	3.1507E-79	NK/CD8 T cells	Mrpl42
1.4626E-83	0.39347689	0.36	0.298	3.6266E-79	NK/CD8 T cells	Ndufv2
1.6612E-83	0.35162598	0.36	0.295	4.1191E-79	NK/CD8 T cells	Vcp

2.9644E-83	0.42559871	0.292	0.23	7.3502E-79	NK/CD8 T cells	Dnttip2
1.1903E-82	0.36198166	0.34	0.272	2.9514E-78	NK/CD8 T cells	Ppp1r14b
2.0513E-82	0.36898686	0.268	0.205	5.0862E-78	NK/CD8 T cells	Ccndbp1
2.1455E-82	0.42188764	0.329	0.268	5.3197E-78	NK/CD8 T cells	Eif4g1
2.4712E-82	0.30175463	0.402	0.335	6.1274E-78	NK/CD8 T cells	Rer1
8.9862E-82	0.46265336	0.286	0.226	2.2281E-77	NK/CD8 T cells	Snrnp27
1.6338E-81	0.45924766	0.29	0.229	4.0511E-77	NK/CD8 T cells	Ppp4r2
2.9909E-81	0.47881222	0.317	0.258	7.4161E-77	NK/CD8 T cells	Fkbp4
3.5308E-81	0.32994007	0.29	0.226	8.7545E-77	NK/CD8 T cells	Ifnar1
5.0476E-81	0.41003593	0.284	0.222	1.2516E-76	NK/CD8 T cells	Vps36
5.592E-81	0.29113445	0.286	0.221	1.3865E-76	NK/CD8 T cells	Gpbp1l1
8.1694E-81	0.41254135	0.297	0.235	2.0256E-76	NK/CD8 T cells	Otulin
9.1558E-81	0.35904857	0.277	0.214	2.2702E-76	NK/CD8 T cells	Atp2a3
1.0411E-80	0.30169947	0.34	0.273	2.5814E-76	NK/CD8 T cells	Tnrc6c
1.128E-80	0.42604558	0.325	0.265	2.797E-76	NK/CD8 T cells	Ctbp1
3.5105E-80	0.38462473	0.342	0.281	8.7042E-76	NK/CD8 T cells	Ube2n
6.937E-80	0.27913383	0.425	0.359	1.72E-75	NK/CD8 T cells	Papola
7.9959E-80	0.4861414	0.263	0.205	1.9826E-75	NK/CD8 T cells	Vti1b
1.071E-79	0.37581134	0.328	0.267	2.6554E-75	NK/CD8 T cells	Polr2g
1.9523E-79	0.54937254	0.257	0.202	4.8408E-75	NK/CD8 T cells	Samm50
3.3232E-79	0.42886973	0.303	0.243	8.2399E-75	NK/CD8 T cells	Krcc1
4.6185E-79	0.27140443	0.402	0.336	1.1452E-74	NK/CD8 T cells	Birc6
9.4058E-79	0.31954821	0.376	0.315	2.3322E-74	NK/CD8 T cells	Polr2j
1.5518E-78	0.37131651	0.332	0.269	3.8476E-74	NK/CD8 T cells	Cbx1
2.1819E-78	0.33508638	0.362	0.299	5.4101E-74	NK/CD8 T cells	Snw1
5.1446E-78	0.34943332	0.278	0.217	1.2756E-73	NK/CD8 T cells	Pkn2
5.9206E-78	0.52158689	0.259	0.203	1.468E-73	NK/CD8 T cells	Mrpl12
1.5095E-77	0.36001935	0.359	0.296	3.7428E-73	NK/CD8 T cells	Smc1a
3.7867E-77	0.43832111	0.284	0.227	9.389E-73	NK/CD8 T cells	M6pr
4.1025E-77	0.41615693	0.297	0.238	1.0172E-72	NK/CD8 T cells	Sra1
7.5234E-77	0.49196778	0.254	0.198	1.8654E-72	NK/CD8 T cells	Ifi35
2.632E-76	0.35612154	0.376	0.315	6.526E-72	NK/CD8 T cells	Bod1l
1.1823E-75	0.39188336	0.33	0.273	2.9315E-71	NK/CD8 T cells	Ndufc2
1.3086E-75	0.27945928	0.389	0.325	3.2448E-71	NK/CD8 T cells	Zfand6
6.9434E-75	0.4083778	0.31	0.252	1.7216E-70	NK/CD8 T cells	Ufc1
3.4953E-74	0.30370252	0.303	0.239	8.6665E-70	NK/CD8 T cells	Pcmtd1
3.7885E-74	0.39381231	0.303	0.246	9.3936E-70	NK/CD8 T cells	Psmc6
5.2405E-74	0.50195107	0.259	0.205	1.2994E-69	NK/CD8 T cells	Ndufab1
1.6019E-73	0.48523165	0.274	0.22	3.972E-69	NK/CD8 T cells	0610012G03R
1.9391E-73	0.48951769	0.269	0.215	4.808E-69	NK/CD8 T cells	Rab18
7.2574E-73	0.38007166	0.349	0.293	1.7995E-68	NK/CD8 T cells	Eif3d
1.2083E-72	0.29465339	0.335	0.274	2.996E-68	NK/CD8 T cells	Ubn1
4.7368E-72	0.47954526	0.263	0.209	1.1745E-67	NK/CD8 T cells	Psmd2
4.1053E-71	0.2863758	0.345	0.284	1.0179E-66	NK/CD8 T cells	Wdr33
1.0618E-70	0.43759212	0.273	0.219	2.6328E-66	NK/CD8 T cells	Pfdn6
1.549E-70	0.39287954	0.287	0.231	3.8409E-66	NK/CD8 T cells	Ogdh
8.0169E-70	0.4012288	0.329	0.273	1.9878E-65	NK/CD8 T cells	Ddx21
9.5754E-70	0.32168223	0.308	0.25	2.3742E-65	NK/CD8 T cells	Actr10
1.6635E-69	0.26776105	0.413	0.353	4.1246E-65	NK/CD8 T cells	Chic2
5.4456E-69	0.49240257	0.256	0.205	1.3502E-64	NK/CD8 T cells	Dnajc19

5.5438E-69	0.40073644	0.306	0.251	1.3746E-64	NK/CD8 T cells	Srp72
9.0728E-69	0.40753342	0.262	0.207	2.2496E-64	NK/CD8 T cells	Fam204a
9.7419E-69	0.42728075	0.289	0.236	2.4155E-64	NK/CD8 T cells	Grpel1
1.0236E-68	0.44578012	0.284	0.231	2.5379E-64	NK/CD8 T cells	Adh5
1.387E-68	0.31367811	0.314	0.256	3.439E-64	NK/CD8 T cells	Gpatch8
1.4544E-68	0.26725337	0.441	0.38	3.6062E-64	NK/CD8 T cells	Cct4
2.5584E-68	0.35774107	0.323	0.268	6.3434E-64	NK/CD8 T cells	Lamtor5
2.9656E-68	0.26818554	0.411	0.349	7.3531E-64	NK/CD8 T cells	Ostc
2.2481E-67	0.39966414	0.259	0.205	5.5741E-63	NK/CD8 T cells	Ppp2r2a
2.4162E-67	0.34798322	0.304	0.247	5.9909E-63	NK/CD8 T cells	Rad21
3.2091E-67	0.32368048	0.299	0.242	7.9569E-63	NK/CD8 T cells	Ppp6r1
3.6425E-67	0.31717408	0.343	0.287	9.0315E-63	NK/CD8 T cells	Mrpl20
5.9176E-67	0.37803246	0.322	0.268	1.4673E-62	NK/CD8 T cells	Ghitm
6.3547E-67	0.43822865	0.264	0.213	1.5756E-62	NK/CD8 T cells	Nars
2.4216E-66	0.35472741	0.323	0.266	6.0043E-62	NK/CD8 T cells	Anp32e
3.4904E-66	0.42758466	0.267	0.215	8.6543E-62	NK/CD8 T cells	Snx6
3.812E-66	0.31615609	0.334	0.278	9.4519E-62	NK/CD8 T cells	Dynlrb1
7.8787E-66	0.43226457	0.261	0.209	1.9535E-61	NK/CD8 T cells	Commd6
9.2881E-66	0.41325894	0.274	0.223	2.303E-61	NK/CD8 T cells	Sod2
1.1103E-65	0.40903283	0.301	0.249	2.753E-61	NK/CD8 T cells	Uqcrc1
2.1006E-65	0.26850587	0.345	0.288	5.2085E-61	NK/CD8 T cells	Snrpc
2.1641E-65	0.37506915	0.274	0.22	5.3659E-61	NK/CD8 T cells	Vbp1
5.0108E-65	0.31774956	0.374	0.318	1.2424E-60	NK/CD8 T cells	Rrp1
1.9705E-64	0.4083698	0.277	0.226	4.8859E-60	NK/CD8 T cells	Tmem208
2.6524E-64	0.40317763	0.251	0.198	6.5767E-60	NK/CD8 T cells	H2-M3
5.7925E-64	0.32250275	0.376	0.323	1.4363E-59	NK/CD8 T cells	Mrps16
9.3501E-64	0.41414227	0.256	0.205	2.3184E-59	NK/CD8 T cells	Ppp2r1a
2.4265E-63	0.33184372	0.322	0.269	6.0165E-59	NK/CD8 T cells	Psmd11
3.1323E-63	0.32302606	0.329	0.274	7.7666E-59	NK/CD8 T cells	Dnajb11
3.3019E-63	0.25331921	0.259	0.203	8.1872E-59	NK/CD8 T cells	Swt1
5.7606E-63	0.43691739	0.251	0.201	1.4283E-58	NK/CD8 T cells	Rbck1
9.7438E-63	0.46043194	0.258	0.209	2.416E-58	NK/CD8 T cells	Exoc3
2.1277E-62	0.29282332	0.387	0.331	5.2756E-58	NK/CD8 T cells	Khdrbs1
3.7207E-62	0.36162418	0.275	0.221	9.2255E-58	NK/CD8 T cells	Suz12
7.9738E-62	0.45111858	0.255	0.206	1.9771E-57	NK/CD8 T cells	Trappc2l
3.8873E-61	0.35106205	0.317	0.264	9.6387E-57	NK/CD8 T cells	Supt16
2.0139E-60	0.32692784	0.325	0.273	4.9933E-56	NK/CD8 T cells	Snf8
2.2196E-60	0.31772669	0.303	0.25	5.5036E-56	NK/CD8 T cells	Psmd13
2.2505E-60	0.30506294	0.338	0.284	5.58E-56	NK/CD8 T cells	Ap2m1
2.3226E-60	0.29679904	0.375	0.321	5.759E-56	NK/CD8 T cells	St13
2.4826E-60	0.31387594	0.302	0.249	6.1556E-56	NK/CD8 T cells	Baz1b
2.0434E-59	0.29694071	0.32	0.268	5.0666E-55	NK/CD8 T cells	Mapk1
4.586E-59	0.25208666	0.346	0.291	1.1371E-54	NK/CD8 T cells	Sirt7
1.1279E-58	0.37183805	0.299	0.25	2.7967E-54	NK/CD8 T cells	Aamp
2.2993E-58	0.38492805	0.283	0.234	5.7011E-54	NK/CD8 T cells	Txnl4a
4.0174E-58	0.27134731	0.297	0.244	9.9612E-54	NK/CD8 T cells	Zfp148
4.4048E-57	0.3636503	0.308	0.26	1.0922E-52	NK/CD8 T cells	Chchd1
7.4576E-57	0.37017283	0.306	0.257	1.8491E-52	NK/CD8 T cells	Nudc
9.0303E-57	0.34197579	0.288	0.238	2.2391E-52	NK/CD8 T cells	Sap18
9.5777E-57	0.25333479	0.335	0.282	2.3748E-52	NK/CD8 T cells	Akap9

1.3413E-56	0.25074625	0.338	0.283	3.3258E-52	NK/CD8 T cells	Cdk11b
2.5881E-56	0.35922902	0.303	0.253	6.4172E-52	NK/CD8 T cells	Gtpbp4
2.7167E-56	0.33129269	0.349	0.298	6.7361E-52	NK/CD8 T cells	Bola2
2.7478E-56	0.32907373	0.319	0.27	6.8132E-52	NK/CD8 T cells	Jtb
2.6973E-55	0.30314095	0.331	0.28	6.6881E-51	NK/CD8 T cells	Mrps21
1.6358E-54	0.28455091	0.278	0.227	4.056E-50	NK/CD8 T cells	Vezf1
2.573E-54	0.33526328	0.267	0.217	6.3797E-50	NK/CD8 T cells	Fam133b
8.7051E-54	0.27857527	0.282	0.232	2.1584E-49	NK/CD8 T cells	Tnks2
9.0323E-54	0.38098422	0.259	0.212	2.2396E-49	NK/CD8 T cells	Uba2
5.3532E-53	0.32705157	0.281	0.233	1.3273E-48	NK/CD8 T cells	Uba1
6.3936E-53	0.43012208	0.259	0.214	1.5853E-48	NK/CD8 T cells	Ergic3
6.7475E-53	0.33395092	0.266	0.219	1.6731E-48	NK/CD8 T cells	Pcmt1
9.7718E-53	0.27146977	0.327	0.277	2.4229E-48	NK/CD8 T cells	Lsm14a
3.6977E-51	0.32895408	0.265	0.218	9.1684E-47	NK/CD8 T cells	Sike1
3.9012E-50	0.2525461	0.36	0.31	9.6731E-46	NK/CD8 T cells	Lamtor1
7.644E-50	0.33566501	0.32	0.275	1.8953E-45	NK/CD8 T cells	Emc6
1.417E-49	0.26502612	0.305	0.255	3.5134E-45	NK/CD8 T cells	Rpn2
2.7183E-49	0.36465068	0.257	0.212	6.7399E-45	NK/CD8 T cells	Dazap1
3.7864E-49	0.37184746	0.267	0.223	9.3883E-45	NK/CD8 T cells	Eprs
5.897E-49	0.3023745	0.3	0.254	1.4622E-44	NK/CD8 T cells	Phf5a
6.9301E-49	0.29106653	0.263	0.218	1.7183E-44	NK/CD8 T cells	Med21
1.0486E-48	0.27944267	0.305	0.257	2.6001E-44	NK/CD8 T cells	Zmiz1
1.3933E-48	0.25059926	0.365	0.317	3.4547E-44	NK/CD8 T cells	Nsmce4a
1.7156E-47	0.36263782	0.255	0.213	4.2537E-43	NK/CD8 T cells	Sar1a
2.3715E-47	0.32000807	0.298	0.253	5.88E-43	NK/CD8 T cells	Wtap
3.7956E-47	0.33161825	0.254	0.209	9.4111E-43	NK/CD8 T cells	Smndc1
4.8228E-47	0.30676356	0.264	0.218	1.1958E-42	NK/CD8 T cells	Lsm3
3.8309E-45	0.2664795	0.344	0.299	9.4988E-41	NK/CD8 T cells	Psma6
7.5835E-45	0.34990339	0.272	0.231	1.8803E-40	NK/CD8 T cells	Nudt21
1.6029E-44	0.34232516	0.283	0.241	3.9743E-40	NK/CD8 T cells	Psmd7
1.6683E-44	0.32894234	0.294	0.251	4.1365E-40	NK/CD8 T cells	Psmc5
3.5775E-44	0.27900053	0.323	0.28	8.8703E-40	NK/CD8 T cells	Marc2
4.4026E-44	0.25167689	0.4	0.355	1.0916E-39	NK/CD8 T cells	Eif5b
4.9863E-44	0.36776737	0.268	0.228	1.2363E-39	NK/CD8 T cells	Mrpl34
6.23E-44	0.39286375	0.254	0.214	1.5447E-39	NK/CD8 T cells	Pa2g4
7.4604E-44	0.25703092	0.296	0.252	1.8498E-39	NK/CD8 T cells	Nrbp1
8.4564E-44	0.37797174	0.255	0.214	2.0968E-39	NK/CD8 T cells	Ahsa1
1.0463E-43	0.315807	0.29	0.248	2.5944E-39	NK/CD8 T cells	Ik
1.0833E-43	0.36302576	0.266	0.225	2.6859E-39	NK/CD8 T cells	Akirin2
1.2627E-42	0.26802231	0.255	0.212	3.1308E-38	NK/CD8 T cells	Setd5
1.838E-42	0.28654509	0.272	0.229	4.5572E-38	NK/CD8 T cells	Pcif1
2.8507E-42	0.28885463	0.332	0.291	7.0682E-38	NK/CD8 T cells	Eif3g
3.5969E-42	0.26311384	0.323	0.28	8.9185E-38	NK/CD8 T cells	Churc1
3.0071E-41	0.31775637	0.254	0.213	7.4561E-37	NK/CD8 T cells	Ptbp1
4.1556E-41	0.36601487	0.263	0.224	1.0304E-36	NK/CD8 T cells	Ssbp1
5.9625E-41	0.3060287	0.293	0.252	1.4784E-36	NK/CD8 T cells	Commd8
6.3253E-41	0.36706665	0.251	0.212	1.5683E-36	NK/CD8 T cells	Skp1a
1.5964E-40	0.31223168	0.274	0.234	3.9583E-36	NK/CD8 T cells	Wdr83os
1.6986E-40	0.2521441	0.254	0.212	4.2117E-36	NK/CD8 T cells	Cnot3
1.0145E-39	0.26114522	0.335	0.295	2.5155E-35	NK/CD8 T cells	Aimp1

1.234E-39	0.28487806	0.263	0.222	3.0597E-35	NK/CD8 T cells	Sbds
2.1078E-39	0.29039621	0.271	0.232	5.2264E-35	NK/CD8 T cells	Dhx15
5.0149E-39	0.29592226	0.306	0.267	1.2435E-34	NK/CD8 T cells	Ciao2a
1.5194E-37	0.31668616	0.266	0.229	3.7674E-33	NK/CD8 T cells	Prpf8
2.7374E-37	0.30909511	0.267	0.229	6.7874E-33	NK/CD8 T cells	Mcrip1
4.744E-37	0.28299217	0.293	0.255	1.1763E-32	NK/CD8 T cells	Psma5
8.0579E-37	0.29560861	0.257	0.218	1.998E-32	NK/CD8 T cells	Gps2
1.7254E-36	0.30327576	0.251	0.213	4.2781E-32	NK/CD8 T cells	Spg21
4.1193E-36	0.27312138	0.281	0.243	1.0214E-31	NK/CD8 T cells	Strap
6.6218E-36	0.36605303	0.252	0.216	1.6419E-31	NK/CD8 T cells	Nhp2
1.9385E-35	0.30606337	0.265	0.23	4.8065E-31	NK/CD8 T cells	Emc7
2.6109E-35	0.25120879	0.276	0.238	6.4738E-31	NK/CD8 T cells	Alkbh5
2.7878E-35	0.28500986	0.264	0.227	6.9123E-31	NK/CD8 T cells	U2af2
5.7483E-35	0.2540008	0.271	0.235	1.4253E-30	NK/CD8 T cells	Txndc9
1.3021E-34	0.30797355	0.263	0.228	3.2285E-30	NK/CD8 T cells	Psmb7
1.8428E-33	0.31075336	0.259	0.225	4.5691E-29	NK/CD8 T cells	Tmco1
3.6281E-33	0.28231915	0.277	0.241	8.996E-29	NK/CD8 T cells	Fxr1
2.0307E-32	0.31228591	0.251	0.217	5.035E-28	NK/CD8 T cells	Hadha
4.6145E-32	0.25053474	0.269	0.234	1.1442E-27	NK/CD8 T cells	Copb1
1.1643E-28	0.30942091	0.259	0.229	2.8868E-24	NK/CD8 T cells	Mrps18c
3.3123E-24	0.27382598	0.258	0.23	8.2129E-20	NK/CD8 T cells	Utp3
3.0931E-19	0.25150052	0.274	0.25	7.6693E-15	NK/CD8 T cells	Chrac1
0	6.82984746	0.948	0.023	0	B cells	Ebf1
0	6.56867052	0.98	0.08	0	B cells	Cd79a
0	6.70472492	0.915	0.027	0	B cells	Ms4a1
0	5.93174089	0.959	0.073	0	B cells	Cd79b
0	5.37572648	0.917	0.072	0	B cells	Iglc2
0	5.67399028	0.871	0.05	0	B cells	H2-DMb2
0	3.85735719	0.981	0.167	0	B cells	H2-Eb1
0	3.58088007	0.983	0.187	0	B cells	H2-Ab1
0	5.95857857	0.834	0.043	0	B cells	Bank1
0	5.93807039	0.818	0.038	0	B cells	Ighd
0	3.95438138	0.923	0.148	0	B cells	Mef2c
0	4.53806193	0.822	0.049	0	B cells	Iglc3
0	3.76365103	0.988	0.222	0	B cells	H2-Aa
0	6.44238208	0.723	0.018	0	B cells	Fcmr
0	6.06184894	0.713	0.022	0	B cells	Scd1
0	5.01953618	0.691	0.038	0	B cells	Siglecg
0	4.14983875	0.733	0.081	0	B cells	Ralgps2
0	5.89313192	0.661	0.016	0	B cells	Pax5
0	2.68405107	0.842	0.207	0	B cells	Napsa
0	2.9399716	0.785	0.162	0	B cells	H2-DMa
0	3.38043055	0.791	0.173	0	B cells	Gm49980
0	4.00743496	0.998	0.398	0	B cells	Cd74
0	5.22822688	0.623	0.029	0	B cells	H2-Ob
0	5.05041972	0.61	0.027	0	B cells	Fcrla
0	2.70501145	0.723	0.142	0	B cells	Ly86
0	5.62859113	0.99	0.419	0	B cells	Igkc
0	5.43764561	0.587	0.026	0	B cells	Tnfrsf13c
0	3.76832954	0.961	0.41	0	B cells	Ighm

0	3.4980546	0.637	0.1	0	B cells	Cd55
0	5.53524183	0.549	0.016	0	B cells	Cd19
0	6.01935374	0.547	0.014	0	B cells	Fcer2a
0	1.78562186	0.758	0.23	0	B cells	Pou2f2
0	3.8548469	0.597	0.078	0	B cells	Pkig
0	1.63303715	0.708	0.199	0	B cells	Ctsh
0	6.56342287	0.516	0.012	0	B cells	Gm31243
0	3.57331485	0.562	0.06	0	B cells	Bcl11a
0	3.52398603	0.582	0.091	0	B cells	Chchd10
0	2.84156574	0.582	0.092	0	B cells	Cd81
0	5.87333923	0.483	0.011	0	B cells	Vpreb3
0	2.96677831	0.54	0.075	0	B cells	Serpinb1a
0	4.38475668	0.492	0.029	0	B cells	Blnk
0	3.65626302	0.524	0.07	0	B cells	Swap70
0	5.12741408	0.471	0.018	0	B cells	Cd22
0	3.93257033	0.509	0.059	0	B cells	Cd83
0	4.77115517	0.466	0.019	0	B cells	Ly6d
0	3.3094441	0.556	0.113	0	B cells	Fchsd2
0	1.75022471	0.685	0.244	0	B cells	Ccr7
0	3.27623829	0.541	0.102	0	B cells	Lmo2
0	3.83810653	0.483	0.045	0	B cells	Cd72
0	2.02738169	0.759	0.331	0	B cells	Smim14
0	4.41125696	0.465	0.038	0	B cells	H2-Oa
0	1.01658134	0.598	0.172	0	B cells	Irf8
0	2.06754457	0.62	0.197	0	B cells	BE692007
0	1.28532048	0.723	0.301	0	B cells	Syk
0	2.11030739	0.589	0.181	0	B cells	Ifi30
0	2.18771027	0.582	0.183	0	B cells	Stap1
0	1.54262687	0.834	0.436	0	B cells	Serp1
0	4.09708759	0.435	0.039	0	B cells	Ciita
0	1.5987621	0.503	0.108	0	B cells	Tcf4
0	2.50546351	0.471	0.077	0	B cells	H2-DMb1
0	3.5147549	0.464	0.072	0	B cells	Hvcn1
0	1.55379599	0.6	0.216	0	B cells	Unc93b1
0	2.02933791	0.931	0.549	0	B cells	Cd37
0	1.33816152	0.553	0.171	0	B cells	Cd24a
0	1.6508866	0.773	0.395	0	B cells	Zfp36l1
0	1.61839971	0.659	0.287	0	B cells	Cmah
0	1.95813524	0.575	0.206	0	B cells	Filip1l
0	3.5247679	0.394	0.031	0	B cells	Spib
0	1.94186294	0.629	0.266	0	B cells	Pold4
0	5.99768106	0.368	0.007	0	B cells	Cr2
0	4.39270216	0.388	0.027	0	B cells	Gga2
0	2.60501417	0.473	0.117	0	B cells	Pxk
0	4.5679568	0.372	0.016	0	B cells	Pxdc1
0	1.61879156	0.862	0.509	0	B cells	Foxp1
0	2.06666574	0.552	0.201	0	B cells	Ptp4a3
0	0.86648903	0.65	0.3	0	B cells	Ctsz
0	3.01971691	0.432	0.082	0	B cells	Mtss1
0	1.02497986	0.613	0.268	0	B cells	Slamf6

0	2.21055571	0.552	0.209	0	B cells	Man1a
0	4.07582919	0.366	0.023	0	B cells	Fcrl1
0	4.47468485	0.363	0.021	0	B cells	Snn
0	1.81930588	0.526	0.187	0	B cells	Sh3bp5
0	2.06389737	0.527	0.191	0	B cells	Snx2
0	1.61861698	0.639	0.305	0	B cells	Hmgn1
0	1.18143022	0.678	0.347	0	B cells	Cd69
0	0.55710274	0.7	0.37	0	B cells	Ctss
0	3.41162136	0.367	0.04	0	B cells	Sdc4
0	1.89850846	0.523	0.197	0	B cells	Tmem243
0	2.80792931	0.417	0.092	0	B cells	Tnfrsf13b
0	1.57778098	0.843	0.518	0	B cells	Gdi2
0	1.51237274	0.67	0.348	0	B cells	Nap1l1
0	1.58556792	0.648	0.326	0	B cells	Ppdpf
0	2.22903113	0.494	0.173	0	B cells	Gpr171
0	3.46423622	0.36	0.042	0	B cells	B3gnt5
0	1.61543284	0.541	0.224	0	B cells	Prkcb
0	3.3074893	0.372	0.055	0	B cells	Btla
0	3.34983294	0.37	0.059	0	B cells	Pgap1
0	3.19606705	0.373	0.063	0	B cells	Cd38
0	2.64410028	0.411	0.102	0	B cells	Zfp318
0	1.40502575	0.412	0.106	0	B cells	Rnase6
0	2.31788726	0.423	0.117	0	B cells	1110059E24R
0	1.03365418	0.82	0.515	0	B cells	Serinc3
0	2.90552384	0.38	0.076	0	B cells	Pml
0	4.42290492	0.316	0.014	0	B cells	Blk
0	0.33675003	0.533	0.233	0	B cells	Plac8
0	4.84416283	0.315	0.015	0	B cells	Ccr6
0	2.22488907	0.438	0.138	0	B cells	Dmxl1
0	1.62696002	0.549	0.25	0	B cells	Snx5
0	2.60501693	0.379	0.084	0	B cells	Plekho1
0	1.67370045	0.51	0.217	0	B cells	Gpr183
0	1.67136913	0.496	0.203	0	B cells	Rel
0	4.40342021	0.312	0.02	0	B cells	Cxcr5
0	2.3641151	0.42	0.129	0	B cells	Parp1
0	3.07670718	0.351	0.06	0	B cells	Dennd5b
0	1.99580048	0.42	0.13	0	B cells	Lat2
0	5.63635413	0.298	0.01	0	B cells	Trim7
0	1.15775506	0.694	0.407	0	B cells	4930523C07R
0	1.06835519	0.697	0.41	0	B cells	Rhoh
0	4.32548355	0.304	0.017	0	B cells	Pou2af1
0	1.86915282	0.464	0.178	0	B cells	Csnk1g3
0	2.17924654	0.422	0.136	0	B cells	Acadl
0	5.16540844	0.309	0.023	0	B cells	Iglc1
0	4.30443803	0.312	0.028	0	B cells	Cd40
0	1.8591373	0.437	0.154	0	B cells	Mif4gd
0	1.91888423	0.393	0.111	0	B cells	March1
0	2.09225095	0.406	0.124	0	B cells	Abhd17b
0	5.21509784	0.29	0.009	0	B cells	Bend5
0	1.8249681	0.42	0.141	0	B cells	Ms4a4c

0	1.46083367	0.619	0.34	0	B cells	Arpc5l
0	1.22782183	0.658	0.382	0	B cells	Malt1
0	2.63827299	0.339	0.065	0	B cells	Pmf1
0	0.55267475	0.759	0.485	0	B cells	Ptprcap
0	5.43719416	0.283	0.01	0	B cells	Gm45745
0	2.15321727	0.384	0.112	0	B cells	Blvrb
0	3.40074339	0.321	0.05	0	B cells	Pkib
0	5.37803931	0.278	0.008	0	B cells	Chst3
0	5.17684657	0.286	0.016	0	B cells	Rasgrp3
0	2.25578785	0.368	0.099	0	B cells	Bach2
0	1.20314877	0.67	0.403	0	B cells	Smap2
0	1.6245303	0.427	0.162	0	B cells	Ikzf3
0	1.51896896	0.484	0.22	0	B cells	Tmem123
0	1.96446831	0.409	0.146	0	B cells	Bmyc
0	1.80071783	0.381	0.122	0	B cells	Hhex
0	2.0229156	0.392	0.134	0	B cells	Lmbrd1
0	1.22346365	0.465	0.207	0	B cells	Tspan32
0	1.45984108	0.551	0.294	0	B cells	Tmod3
0	0.46387382	0.543	0.287	0	B cells	Lyn
0	1.43927661	0.436	0.182	0	B cells	Fam111a
0	0.59188404	0.688	0.434	0	B cells	Gimap6
0	1.24237183	0.575	0.322	0	B cells	Lbh
0	1.46043889	0.48	0.229	0	B cells	Camk2d
0	1.35896445	0.481	0.232	0	B cells	P2ry10
0	1.37250081	0.507	0.26	0	B cells	Cnp
0	2.03976948	0.318	0.072	0	B cells	Cyb561a3
0	1.06176988	0.598	0.354	0	B cells	Tut4
0	1.38711162	0.483	0.239	0	B cells	Etnk1
0	2.37023183	0.326	0.082	0	B cells	Myo1c
0	1.35955049	0.489	0.247	0	B cells	Rabgap1l
0	1.48547622	0.47	0.229	0	B cells	Bin1
0	2.62046168	0.322	0.082	0	B cells	Cd86
0	0.88115033	0.969	0.731	0	B cells	mt-Nd2
0	2.8178319	0.288	0.053	0	B cells	Txndc16
0	0.87430764	0.504	0.269	0	B cells	Gm8369
0	0.91654117	0.98	0.746	0	B cells	mt-Nd1
0	2.44934197	0.296	0.064	0	B cells	Cxxc5
0	2.06469892	0.331	0.1	0	B cells	Strbp
0	0.38870079	0.78	0.549	0	B cells	Ets1
0	2.01623391	0.333	0.104	0	B cells	Lamb3
0	0.82473159	0.736	0.507	0	B cells	mt-Nd5
0	1.72239732	0.381	0.153	0	B cells	Plekha2
0	0.53376193	0.667	0.44	0	B cells	Ly6e
0	3.20449049	0.259	0.033	0	B cells	Rapgef4
0	3.47495455	0.257	0.033	0	B cells	Abca1
0	2.22261507	0.301	0.078	0	B cells	Trp53i11
0	0.88610159	0.691	0.468	0	B cells	Cxcr4
0	1.47943362	0.36	0.139	0	B cells	Pecam1
0	1.36812322	0.429	0.209	0	B cells	Cd2ap
0	0.58810268	0.914	0.695	0	B cells	Rpl35

0	1.79789108	0.365	0.146	0	B cells	Eml4
0	0.71352443	0.961	0.743	0	B cells	mt-Nd4
0	3.2297087	0.253	0.036	0	B cells	Myo1e
0	1.33991657	0.433	0.218	0	B cells	Map3k1
0	0.81653673	0.403	0.19	0	B cells	Ncf1
0	0.55287109	0.925	0.714	0	B cells	Rpl4
0	1.02395171	0.552	0.341	0	B cells	Pgls
0	0.51230797	0.923	0.713	0	B cells	Rpl36a
0	2.81833407	0.256	0.046	0	B cells	Lrrk2
0	2.01184946	0.287	0.077	0	B cells	Wdfy4
0	2.98350696	0.265	0.057	0	B cells	Cd180
0	1.24762479	0.377	0.169	0	B cells	Sypl
0	0.74623551	0.984	0.776	0	B cells	mt-Cytb
0	0.62475728	0.789	0.582	0	B cells	Eef1g
0	1.2168173	0.42	0.213	0	B cells	Arhgap17
0	0.83978918	0.954	0.747	0	B cells	Rps19
0	2.65710082	0.273	0.067	0	B cells	Snx9
0	2.48468615	0.268	0.063	0	B cells	Sbk1
0	0.32294292	0.661	0.457	0	B cells	Ablim1
0	1.86731593	0.344	0.141	0	B cells	Txndc5
0	2.75816995	0.253	0.051	0	B cells	Gm15987
0	0.44737832	0.951	0.749	0	B cells	Rpl10a
0	2.13291958	0.288	0.087	0	B cells	Nrm
0	0.86138468	0.681	0.481	0	B cells	Macf1
0	1.87672615	0.303	0.103	0	B cells	Lpgat1
0	1.98942371	0.276	0.076	0	B cells	Btk
0	0.58714408	0.827	0.627	0	B cells	mt-Co1
0	0.9926962	0.403	0.204	0	B cells	Baz2b
0	0.40053806	0.915	0.717	0	B cells	Rps17
0	0.36086524	0.95	0.752	0	B cells	Rpl3
0	0.26325295	0.948	0.75	0	B cells	Rps18
0	2.76442199	0.255	0.058	0	B cells	Snx30
0	1.26302698	0.392	0.195	0	B cells	Med13
0	0.43458087	0.937	0.741	0	B cells	Rpl5
0	0.3731487	0.829	0.633	0	B cells	Rpl22l1
0	1.06312333	0.493	0.299	0	B cells	Dnajc7
0	0.7650845	0.551	0.359	0	B cells	Acp5
0	0.50820975	0.589	0.398	0	B cells	Satb1
0	1.14281928	0.408	0.218	0	B cells	Dgkd
0	0.38921922	0.809	0.62	0	B cells	Rpl31
0	0.26606282	0.821	0.632	0	B cells	Gm10260
0	0.83212924	0.507	0.32	0	B cells	Ppp3ca
0	0.59237634	0.576	0.389	0	B cells	Erp29
0	0.94801048	0.965	0.779	0	B cells	Ptma
0	0.470429	0.68	0.495	0	B cells	Crlf3
0	1.0382206	0.417	0.232	0	B cells	Dnajc9
0	1.73406855	0.324	0.14	0	B cells	Clec2i
0	0.36308725	0.883	0.7	0	B cells	Rps6
0	1.18334888	0.349	0.166	0	B cells	Birc3
0	0.51295932	0.956	0.774	0	B cells	Rpl14

0	1.38106459	0.323	0.143	0	B cells	Ppp1r16b
0	0.40921592	0.397	0.217	0	B cells	Aldh2
0	0.72417303	0.518	0.339	0	B cells	Klhl24
0	0.54244194	0.955	0.776	0	B cells	Rpl22
0	0.57393857	0.866	0.688	0	B cells	Eif3f
0	1.49787942	0.323	0.146	0	B cells	Ifi209
0	1.86348102	0.301	0.124	0	B cells	Fh1
0	0.47099671	0.828	0.651	0	B cells	Rpl35a
0	0.31309383	0.923	0.747	0	B cells	Rps2
0	0.32866746	0.952	0.776	0	B cells	Rps28
0	1.47405344	0.321	0.146	0	B cells	Ehd4
0	0.80426724	0.569	0.394	0	B cells	Nol7
0	1.39944332	0.337	0.164	0	B cells	Uchl3
0	2.16418809	0.25	0.077	0	B cells	Cerk
0	0.38651151	0.901	0.729	0	B cells	Rpl27a
0	1.75205676	0.27	0.098	0	B cells	AC147806.2
0	0.85943083	0.469	0.297	0	B cells	Baz1a
0	0.79648378	0.582	0.41	0	B cells	Zfp706
0	0.74739379	0.616	0.445	0	B cells	Csk
0	0.84602421	0.336	0.166	0	B cells	Gm45153
0	0.6279017	0.947	0.777	0	B cells	Rpl7
0	0.85683248	0.522	0.353	0	B cells	Scaf11
0	0.79975415	0.974	0.805	0	B cells	Rps20
0	0.45716929	0.933	0.765	0	B cells	Rpl34
0	0.91105736	0.406	0.239	0	B cells	Tcp11l2
0	0.56462381	0.956	0.789	0	B cells	Rpl6
0	0.272843	0.914	0.748	0	B cells	Naca
0	0.2614033	0.873	0.707	0	B cells	Rpl23a
0	0.39202647	0.729	0.566	0	B cells	Rpl13a
0	0.43940818	0.92	0.757	0	B cells	Rpl30
0	0.93129184	0.486	0.323	0	B cells	Gm28438
0	0.43634086	0.898	0.736	0	B cells	Eef2
0	1.03158393	0.369	0.207	0	B cells	Uvrag
0	0.49900756	0.863	0.702	0	B cells	Gnas
0	1.48755582	0.331	0.17	0	B cells	Stt3b
0	1.73597719	0.252	0.091	0	B cells	St6gal1
0	0.82901734	0.487	0.326	0	B cells	Zcchc7
0	0.76328135	0.407	0.246	0	B cells	Ctsc
0	0.78675205	0.497	0.336	0	B cells	Ppp1r11
0	0.39222389	0.968	0.807	0	B cells	Rps24-ps3
0	0.34035453	0.946	0.785	0	B cells	Rpl36
0	0.32734371	0.978	0.818	0	B cells	Rpl12
0	1.0214583	0.418	0.258	0	B cells	Slc38a1
0	0.83418636	0.442	0.283	0	B cells	Inpp5d
0	0.37345664	0.966	0.808	0	B cells	Rack1
0	0.30157507	0.538	0.381	0	B cells	Gimap1
0	1.67354347	0.263	0.106	0	B cells	Map4k1
0	1.13353088	0.292	0.135	0	B cells	Plcg2
0	0.32789892	0.751	0.595	0	B cells	Rpl15
0	0.80228684	0.437	0.281	0	B cells	Samd9l

0	1.04573567	0.295	0.139	0	B cells	Irf5
0	0.50674749	0.693	0.537	0	B cells	Selenow
0	0.43244634	0.957	0.801	0	B cells	Rps7
0	0.56032994	0.825	0.669	0	B cells	Sub1
0	0.30807232	0.865	0.71	0	B cells	Rpl27
0	1.17195189	0.344	0.189	0	B cells	Ccdc50
0	0.6872691	0.511	0.357	0	B cells	Arid4b
0	1.11656732	0.275	0.122	0	B cells	Pik3ap1
0	0.5006652	0.615	0.462	0	B cells	Grb2
0	0.30671699	0.904	0.752	0	B cells	Rps10
0	0.63647507	0.539	0.387	0	B cells	H2-T23
0	0.77339553	0.446	0.294	0	B cells	Sipa1
0	1.23317099	0.3	0.148	0	B cells	Gpr18
0	0.61030032	0.307	0.156	0	B cells	Dok3
0	0.51576893	0.96	0.809	0	B cells	Rps26
0	0.35242277	0.972	0.822	0	B cells	Rpsa
0	0.85373783	0.408	0.258	0	B cells	Herpud1
0	0.27717008	0.946	0.796	0	B cells	Rpl38
0	0.71782638	0.451	0.302	0	B cells	Cirbp
0	0.73137638	0.445	0.296	0	B cells	Itsn2
0	0.93461322	0.342	0.194	0	B cells	Arap2
0	0.39315968	0.969	0.821	0	B cells	Rps24
0	1.31875207	0.289	0.141	0	B cells	Tcf3
0	0.99411762	0.376	0.228	0	B cells	Yeats4
0	0.52073871	0.407	0.259	0	B cells	Rflnb
0	1.53271998	0.269	0.122	0	B cells	Ctso
0	0.50437457	0.95	0.803	0	B cells	Rpl21
0	0.34359958	0.969	0.823	0	B cells	Rps15a
0	0.62069456	0.569	0.423	0	B cells	Add3
0	0.6044385	0.577	0.434	0	B cells	Hnrnpa1
0	1.02105493	0.329	0.187	0	B cells	Aff4
0	0.92335838	0.318	0.176	0	B cells	Mylip
0	1.14596841	0.295	0.153	0	B cells	Tmem131l
0	1.13038362	0.294	0.152	0	B cells	Cenpa
0	0.60243852	0.593	0.451	0	B cells	Nop53
0	0.77736033	0.437	0.296	0	B cells	Rp9
0	0.7035825	0.358	0.218	0	B cells	Pde4b
0	1.30535633	0.252	0.113	0	B cells	Card11
0	0.41157982	0.669	0.531	0	B cells	Snrpg
0	0.89863866	0.385	0.247	0	B cells	Limd1
0	0.85054521	0.364	0.226	0	B cells	Arid5b
0	0.4074861	0.682	0.545	0	B cells	Calr
0	0.4224351	0.477	0.34	0	B cells	St8sia4
0	0.59446088	0.975	0.839	0	B cells	Rps4x
0	1.13212379	0.313	0.177	0	B cells	Nsmce1
0	0.79490477	0.434	0.298	0	B cells	Fli1
0	0.48144449	0.695	0.56	0	B cells	Eif4a2
0	0.77092727	0.341	0.207	0	B cells	Slc12a6
0	0.71184319	0.382	0.249	0	B cells	Tubb4b
0	0.52916674	0.97	0.837	0	B cells	Rps5

0	0.35267123	0.671	0.538	0	B cells	Eif3e
0	0.40007482	0.681	0.549	0	B cells	Gm10076
0	0.67345349	0.455	0.323	0	B cells	Grk6
0	0.33840286	0.976	0.845	0	B cells	Rpl32
0	0.57363526	0.561	0.431	0	B cells	Bptf
0	0.72577615	0.367	0.237	0	B cells	Pde7a
0	0.83267205	0.392	0.262	0	B cells	Slc50a1
0	0.43971628	0.626	0.496	0	B cells	Elf1
0	0.59909523	0.968	0.838	0	B cells	Rpl26
0	0.75603715	0.355	0.225	0	B cells	Foxo1
0	1.19127173	0.253	0.124	0	B cells	Gpr174
0	0.49328783	0.652	0.523	0	B cells	Hsp90b1
0	0.40263868	0.705	0.577	0	B cells	Hnrnpa0
0	0.49967767	0.943	0.816	0	B cells	Rps25
0	0.37745788	0.968	0.842	0	B cells	Rpl11
0	0.39249968	0.615	0.489	0	B cells	Uba52
0	0.57243054	0.503	0.377	0	B cells	Ctcf
0	1.17021603	0.253	0.127	0	B cells	Slc4a7
0	0.73768588	0.401	0.275	0	B cells	Foxn3
0	0.34673669	0.956	0.831	0	B cells	Rpl10
0	0.50298683	0.976	0.851	0	B cells	Rpl13
0	0.38622013	0.562	0.438	0	B cells	Il2rg
0	0.3700587	0.631	0.507	0	B cells	Sp100
0	0.78300073	0.368	0.244	0	B cells	Avl9
0	1.05161085	0.322	0.198	0	B cells	Rtcb
0	0.75155497	0.365	0.241	0	B cells	Arhgef18
0	0.51687564	0.964	0.841	0	B cells	Rpl28
0	0.44919534	0.966	0.844	0	B cells	Rpl37a
0	0.49602716	0.969	0.847	0	B cells	Rps15
0	1.0423946	0.286	0.164	0	B cells	Map2k1
0	1.0188145	0.292	0.17	0	B cells	Rfx7
0	0.4960742	0.453	0.332	0	B cells	Jmjd1c
0	0.56936075	0.469	0.348	0	B cells	Ikbkb
0	0.51014975	0.463	0.343	0	B cells	Phip
0	0.57194067	0.478	0.359	0	B cells	Nucks1
0	0.48433075	0.521	0.402	0	B cells	Smchd1
0	0.39373928	0.965	0.848	0	B cells	Rps13
0	0.30926966	0.787	0.671	0	B cells	Ybx1
0	1.00521271	0.31	0.194	0	B cells	Gm37474
0	0.54446212	0.395	0.279	0	B cells	Grap
0	0.54377516	0.46	0.346	0	B cells	Rps27rt
0	0.80149914	0.318	0.204	0	B cells	Cyth1
0	0.33024388	0.682	0.569	0	B cells	Cnbp
0	1.12276731	0.275	0.162	0	B cells	Rfc1
0	0.46881063	0.599	0.486	0	B cells	Paip2
0	0.36664067	0.677	0.565	0	B cells	Ncl
0	0.89992184	0.35	0.238	0	B cells	Lrmp
0	0.35999371	0.977	0.865	0	B cells	Rpl19
0	0.5295743	0.486	0.375	0	B cells	Snrpd2
0	0.30425667	0.973	0.862	0	B cells	Rps11

0	0.91899984	0.259	0.149	0	B cells	4632427E13R[illegible]
0	0.39761293	0.969	0.86	0	B cells	Rps21
0	0.6296077	0.308	0.199	0	B cells	Stx7
0	0.4509967	0.492	0.384	0	B cells	Gm11478
0	1.01981989	0.26	0.153	0	B cells	Rftn1
0	0.39223558	0.605	0.498	0	B cells	Set
0	0.85544265	0.292	0.185	0	B cells	Tbc1d1
0	0.61785909	0.414	0.307	0	B cells	Sptbn1
0	0.46019956	0.555	0.449	0	B cells	Srsf11
0	1.03083119	0.256	0.15	0	B cells	Ddx41
0	0.40070205	0.968	0.862	0	B cells	Rpl39
0	0.88242231	0.289	0.183	0	B cells	Fam204a
0	0.64950872	0.268	0.163	0	B cells	Capg
0	0.73209835	0.333	0.228	0	B cells	Cpsf6
0	0.53875562	0.978	0.873	0	B cells	Rps8
0	0.35699006	0.678	0.574	0	B cells	Luc7l2
0	0.59172983	0.395	0.292	0	B cells	Srsf9
0	0.66667819	0.361	0.258	0	B cells	Wbp11
0	0.47254236	0.518	0.416	0	B cells	Sfpq
0	0.85967516	0.277	0.175	0	B cells	Rfc2
0	0.79514782	0.294	0.192	0	B cells	Atp2a3
0	0.71776167	0.356	0.254	0	B cells	Tcof1
0	0.57865839	0.429	0.328	0	B cells	Dock10
0	0.68175668	0.279	0.18	0	B cells	Rbm38
0	0.58436872	0.393	0.294	0	B cells	Nsmce4a
0	0.80235847	0.258	0.159	0	B cells	H1f4
0	0.79247046	0.273	0.175	0	B cells	Pip4p1
0	0.75945821	0.334	0.236	0	B cells	Cdv3
0	0.28700867	0.928	0.831	0	B cells	Stk17b
0	0.73161935	0.334	0.237	0	B cells	Galnt1
0	0.7063331	0.343	0.247	0	B cells	Abhd17a
0	0.84672945	0.267	0.171	0	B cells	Cux1
0	0.83533587	0.263	0.168	0	B cells	Dapp1
0	0.89217	0.272	0.178	0	B cells	Ddx54
0	0.31462173	0.954	0.86	0	B cells	Rpl37
0	0.75840158	0.27	0.177	0	B cells	Rest
0	0.76768022	0.308	0.216	0	B cells	Otulin
0	0.43465847	0.975	0.888	0	B cells	Rpl8
0	0.31785405	0.978	0.896	0	B cells	Eef1a1
0	0.32492955	0.974	0.895	0	B cells	Rps3
0	0.33013546	0.978	0.903	0	B cells	Rpl23
0	0.31526233	0.972	0.899	0	B cells	Rps12
0	0.42938794	0.968	0.896	0	B cells	Rpl17
0	0.35517541	0.966	0.895	0	B cells	Rpl41
0	0.27183634	0.965	0.898	0	B cells	Cd52
0	0.40595126	0.981	0.914	0	B cells	Rps29
0	0.46746015	0.981	0.915	0	B cells	Rps3a1
0	0.52829285	0.976	0.916	0	B cells	Rpl18a
0	0.46035585	0.977	0.922	0	B cells	Rpl9
0	0.25412986	0.983	0.934	0	B cells	Rps14

0	0.59463861	0.978	0.933	0	B cells	Rps27
0	1.12881573	0.974	0.935	0	B cells	H3f3a
0	0.36476335	0.966	0.941	0	B cells	Ddx5
0	0.27487941	0.904	0.88	0	B cells	Jund
0	0.51738682	0.988	0.978	0	B cells	mt-Rnr2
0	0.53437882	0.988	0.981	0	B cells	Fau
0	0.25326292	0.983	0.979	0	B cells	Rps9
5.152E-307	0.25991201	0.518	0.374	1.278E-302	B cells	Gimap4
3.681E-303	0.33403429	0.688	0.576	9.126E-299	B cells	Hsp90aa1
9.096E-302	0.40705354	0.554	0.449	2.255E-297	B cells	2410006H16R
1.117E-301	0.46906766	0.471	0.368	2.77E-297	B cells	Kmt2a
1.378E-300	0.6122708	0.373	0.274	3.417E-296	B cells	Nfatc3
1.96E-300	0.72684316	0.274	0.183	4.859E-296	B cells	Trim26
9.768E-299	0.38261565	0.574	0.463	2.422E-294	B cells	Gm47283
7.688E-296	0.27909799	0.779	0.654	1.906E-291	B cells	Cox7a2l
1.002E-295	0.48861843	0.464	0.366	2.484E-291	B cells	Dbnl
3.469E-294	0.3213515	0.691	0.585	8.602E-290	B cells	Srsf3
3.905E-284	0.45892849	0.476	0.381	9.683E-280	B cells	Hnrnpd
7.673E-284	0.40359385	0.548	0.447	1.903E-279	B cells	Krtcap2
4.057E-283	0.26303376	0.757	0.614	1.006E-278	B cells	Eif3h
5.245E-281	0.56675563	0.395	0.303	1.301E-276	B cells	Mrps16
2.066E-280	0.69020549	0.314	0.224	5.123E-276	B cells	Tcf12
2.046E-278	0.61034664	0.341	0.25	5.072E-274	B cells	Cbx1
1.179E-277	0.65217587	0.274	0.184	2.922E-273	B cells	Senp7
4.66E-275	0.36980337	0.598	0.501	1.156E-270	B cells	Polr1d
2.434E-273	0.45506006	0.486	0.395	6.035E-269	B cells	Use1
3.651E-269	0.38854761	0.458	0.348	9.052E-265	B cells	Cmtm7
5.47E-269	0.34391571	0.578	0.478	1.356E-264	B cells	Irf2
3.184E-265	0.74581992	0.264	0.181	7.894E-261	B cells	H2-M3
1.983E-264	0.65509122	0.341	0.253	4.916E-260	B cells	Sf3b3
2.1E-262	0.48014329	0.46	0.367	5.207E-258	B cells	Fubp1
7.593E-262	0.52346478	0.424	0.335	1.883E-257	B cells	Eif5b
1.334E-261	0.61329202	0.336	0.248	3.308E-257	B cells	Anp32e
5.396E-261	0.42328742	0.499	0.405	1.338E-256	B cells	Mtdh
8.338E-259	0.35715725	0.586	0.485	2.067E-254	B cells	Nsa2
4.477E-257	0.41989507	0.436	0.34	1.11E-252	B cells	Smc6
1.975E-255	0.4182218	0.5	0.411	4.898E-251	B cells	Cct2
2.588E-255	0.66009637	0.264	0.181	6.416E-251	B cells	Cybc1
8.285E-255	0.58954038	0.351	0.262	2.054E-250	B cells	R3hdm1
1.633E-252	0.80827274	0.252	0.173	4.05E-248	B cells	Hexb
6.733E-252	0.58048784	0.365	0.278	1.669E-247	B cells	Zfp644
1.813E-247	0.46681266	0.319	0.222	4.496E-243	B cells	Tespa1
1.913E-246	0.42060729	0.464	0.372	4.744E-242	B cells	Rab21
4.296E-243	0.32824731	0.592	0.495	1.065E-238	B cells	Tra2b
1.432E-241	0.53303274	0.354	0.267	3.551E-237	B cells	Srsf10
1.352E-238	0.54454717	0.282	0.198	3.352E-234	B cells	Far1
5.821E-237	0.44530095	0.433	0.345	1.443E-232	B cells	Cdc42se2
7.453E-237	0.30227436	0.647	0.547	1.848E-232	B cells	Hnrnpa3
7.685E-237	0.54459758	0.307	0.219	1.906E-232	B cells	Gm43362
4.098E-236	0.53215436	0.457	0.368	1.016E-231	B cells	Manf

7.719E-234	0.4917869	0.388	0.304	1.914E-229	B cells	Smarca5
4.605E-233	0.39243866	0.486	0.395	1.142E-228	B cells	Xrn2
1.203E-232	0.52337122	0.332	0.245	2.982E-228	B cells	Chd2
2.336E-232	0.70737976	0.253	0.176	5.793E-228	B cells	BC004004
6.035E-232	0.6485904	0.258	0.18	1.496E-227	B cells	Tia1
1.019E-230	0.5017446	0.429	0.347	2.527E-226	B cells	Psma1
2.038E-230	0.3365163	0.422	0.324	5.053E-226	B cells	Rcsd1
3.88E-229	0.61486267	0.309	0.229	9.621E-225	B cells	Zfas1
3.124E-227	0.67942208	0.304	0.229	7.745E-223	B cells	Ndufs8
3.934E-227	0.25147388	0.73	0.624	9.755E-223	B cells	Psmb1
7.611E-227	0.40476575	0.477	0.395	1.887E-222	B cells	Eif3i
1.34E-226	0.28752869	0.62	0.534	3.322E-222	B cells	Kmt2e
1.342E-224	0.30803783	0.511	0.412	3.327E-220	B cells	Rasgrp2
2.487E-223	0.32331612	0.5	0.41	6.166E-219	B cells	9930111J21Ri
4.789E-222	0.53831197	0.387	0.299	1.187E-217	B cells	Pdia6
1.161E-221	0.35630977	0.53	0.443	2.879E-217	B cells	Wasf2
2.413E-220	0.41711365	0.388	0.297	5.983E-216	B cells	Traf3ip3
5.622E-219	0.4122973	0.45	0.368	1.394E-214	B cells	Sys1
9.851E-219	0.29782014	0.625	0.538	2.443E-214	B cells	Rbm25
6.623E-217	0.37390128	0.475	0.388	1.642E-212	B cells	Stk24
1.711E-215	0.44361569	0.424	0.34	4.242E-211	B cells	Matr3
5.485E-215	0.43258967	0.433	0.354	1.36E-210	B cells	G3bp1
1.951E-212	0.38837623	0.422	0.335	4.837E-208	B cells	Polr2a
6.989E-211	0.53721012	0.36	0.282	1.733E-206	B cells	Ppm1g
9.49E-211	0.42107841	0.448	0.367	2.353E-206	B cells	Bcap31
2.81E-208	0.47165255	0.383	0.302	6.968E-204	B cells	Ddx50
1.924E-206	0.40277049	0.35	0.266	4.771E-202	B cells	Ptk2b
9.546E-205	0.50850713	0.378	0.305	2.367E-200	B cells	Phb2
1.799E-204	0.40432748	0.441	0.357	4.46E-200	B cells	Rbbp6
6.265E-204	0.50687755	0.335	0.254	1.553E-199	B cells	Birc2
6.987E-204	0.33712994	0.536	0.453	1.732E-199	B cells	Chd4
5.236E-203	0.44299055	0.39	0.31	1.298E-198	B cells	Gpbp1
1.992E-202	0.34485959	0.521	0.433	4.94E-198	B cells	Hspe1
4.807E-202	0.40661472	0.43	0.35	1.192E-197	B cells	Prpf4b
4.915E-202	0.64188673	0.265	0.193	1.219E-197	B cells	Tcea1
3.629E-201	0.36468259	0.251	0.173	8.997E-197	B cells	Fosb
1.286E-200	0.65861549	0.273	0.202	3.188E-196	B cells	Ak2
7.862E-200	0.55614549	0.304	0.224	1.949E-195	B cells	Kcnq1ot1
4.828E-197	0.34921347	0.494	0.415	1.197E-192	B cells	Eif3c
5.412E-197	0.49886353	0.319	0.241	1.342E-192	B cells	Mdm4
2.818E-196	0.33402045	0.448	0.365	6.988E-192	B cells	Ppp1r15a
1.521E-192	0.56188991	0.291	0.218	3.772E-188	B cells	Dhx9
5.33E-189	0.32716228	0.542	0.461	1.321E-184	B cells	Hmgb1
1.047E-184	0.3299419	0.49	0.412	2.596E-180	B cells	Arid1a
3.845E-184	0.30911269	0.514	0.432	9.534E-180	B cells	Srsf7
2.4E-183	0.51640382	0.336	0.266	5.951E-179	B cells	Lsm6
7.608E-183	0.2833526	0.542	0.46	1.886E-178	B cells	Pnisr
1.674E-182	0.5305028	0.3	0.228	4.15E-178	B cells	Cebpz
1.383E-181	0.56982671	0.309	0.239	3.429E-177	B cells	Gtpbp4
1.947E-181	0.31040608	0.533	0.457	4.828E-177	B cells	Ube2d2a

2.246E-181	0.39543559	0.448	0.371	5.57E-177	B cells	Ttc14
1.307E-180	0.66177971	0.258	0.19	3.24E-176	B cells	Ilf2
1.979E-179	0.26604225	0.588	0.503	4.906E-175	B cells	Snrpe
7.064E-179	0.33044393	0.479	0.404	1.752E-174	B cells	Tmem134
8.66E-179	0.45130241	0.319	0.243	2.147E-174	B cells	Sgms1
2.335E-178	0.45622779	0.357	0.282	5.79E-174	B cells	Pole4
2.876E-175	0.40658079	0.412	0.341	7.13E-171	B cells	Sarnp
8.993E-175	0.51280099	0.33	0.26	2.23E-170	B cells	Ddx21
1.638E-172	0.4702402	0.373	0.306	4.06E-168	B cells	Rrp1
1.993E-172	0.31549746	0.5	0.425	4.943E-168	B cells	Ddx24
6.721E-170	0.63819255	0.271	0.206	1.666E-165	B cells	Tob2
3.75E-169	0.42505345	0.394	0.32	9.299E-165	B cells	Gm1966
1.165E-168	0.32584107	0.443	0.369	2.888E-164	B cells	Atf4
2.572E-167	0.37881812	0.412	0.34	6.378E-163	B cells	Ubap2l
8.448E-166	0.32152747	0.384	0.307	2.095E-161	B cells	BC005624
8.671E-166	0.32521916	0.273	0.198	2.15E-161	B cells	Sik1
1.563E-164	0.5704718	0.278	0.215	3.877E-160	B cells	Selenoh
9.51E-164	0.55486533	0.299	0.235	2.358E-159	B cells	Gm15500
2.357E-161	0.30891525	0.524	0.447	5.843E-157	B cells	Bclaf1
2.379E-161	0.41923806	0.315	0.244	5.898E-157	B cells	Safb2
4.057E-157	0.31842839	0.467	0.394	1.006E-152	B cells	Rbm5
4.344E-157	0.28713181	0.54	0.466	1.077E-152	B cells	Csde1
8.562E-155	0.49383587	0.28	0.216	2.123E-150	B cells	Gm9843
7.555E-152	0.43801217	0.363	0.3	1.873E-147	B cells	Tcp1
3.243E-151	0.30046459	0.49	0.419	8.042E-147	B cells	Rpl36al
8.984E-149	0.37238299	0.394	0.329	2.228E-144	B cells	Snu13
7.687E-148	0.51845601	0.289	0.227	1.906E-143	B cells	Rnps1
9.492E-148	0.42958594	0.299	0.232	2.353E-143	B cells	Cdk13
2.847E-147	0.34147594	0.404	0.337	7.06E-143	B cells	Hnrnpdl
3.875E-147	0.46379556	0.314	0.25	9.608E-143	B cells	Cisd2
5.189E-147	0.31984488	0.428	0.36	1.287E-142	B cells	Mknk2
6.556E-147	0.36277368	0.355	0.285	1.626E-142	B cells	Tor1aip1
1.687E-146	0.41998932	0.312	0.247	4.182E-142	B cells	Mef2d
2.459E-146	0.43161449	0.291	0.226	6.097E-142	B cells	Setd2
3.631E-146	0.31120598	0.461	0.392	9.003E-142	B cells	Prpf38b
1.288E-144	0.43241137	0.348	0.283	3.194E-140	B cells	Hnrnpr
1.79E-144	0.34705431	0.417	0.353	4.438E-140	B cells	Eif4b
3.882E-144	0.43638902	0.344	0.28	9.626E-140	B cells	U2surp
7.573E-143	0.55724039	0.268	0.21	1.878E-138	B cells	Idh3g
1.485E-142	0.4518831	0.287	0.225	3.681E-138	B cells	Gtf2a2
5.082E-142	0.44044084	0.312	0.251	1.26E-137	B cells	Znrd1
1.422E-141	0.4195064	0.376	0.316	3.526E-137	B cells	Llph
1.958E-140	0.36234163	0.331	0.265	4.854E-136	B cells	Cib1
1.316E-138	0.39219923	0.353	0.29	3.262E-134	B cells	Zc3h15
7.224E-138	0.45002213	0.261	0.199	1.791E-133	B cells	Trappc5
7.729E-135	0.34816135	0.417	0.353	1.916E-130	B cells	Top2b
1.319E-134	0.47851609	0.321	0.264	3.271E-130	B cells	Emc6
7.474E-134	0.28344948	0.467	0.401	1.853E-129	B cells	Srrm1
8.26E-134	0.32385391	0.337	0.267	2.048E-129	B cells	Atpif1
1.645E-133	0.31246769	0.462	0.399	4.078E-129	B cells	Nop10

1.656E-130	0.40631634	0.292	0.229	4.106E-126	B cells	Pcmtd1
1.728E-130	0.4876649	0.266	0.207	4.284E-126	B cells	Fam133b
2.18E-130	0.34322784	0.461	0.401	5.405E-126	B cells	Sfr1
5.29E-130	0.37554043	0.359	0.298	1.312E-125	B cells	Ddx46
1.658E-129	0.43141357	0.26	0.199	4.112E-125	B cells	Elk4
2.199E-129	0.29187555	0.416	0.347	5.451E-125	B cells	Zfp292
5.913E-129	0.34984665	0.419	0.359	1.466E-124	B cells	Tsn
9.217E-129	0.48835021	0.278	0.221	2.285E-124	B cells	Cetn2
3.267E-127	0.33240042	0.283	0.218	8.102E-123	B cells	Sesn3
4.153E-127	0.36993032	0.301	0.241	1.03E-122	B cells	Epn1
3.757E-126	0.36212657	0.36	0.301	9.316E-122	B cells	Thrap3
2.084E-120	0.35850052	0.322	0.261	5.167E-116	B cells	Smg1
1.356E-119	0.50616153	0.262	0.209	3.362E-115	B cells	Lsm3
2.11E-119	0.50102352	0.267	0.213	5.231E-115	B cells	Ssbp1
1.374E-116	0.4752451	0.3	0.248	3.407E-112	B cells	Nudc
2.404E-115	0.43978498	0.299	0.246	5.96E-111	B cells	Cetn3
5.178E-115	0.30733743	0.316	0.254	1.284E-110	B cells	Nedd9
1.023E-114	0.27727675	0.439	0.379	2.537E-110	B cells	Brd2
5.704E-114	0.2651732	0.402	0.336	1.414E-109	B cells	Nfkb1
1.362E-113	0.46036487	0.305	0.252	3.377E-109	B cells	Ssrp1
2.338E-113	0.41426413	0.334	0.281	5.797E-109	B cells	Eif3g
4.371E-113	0.29196143	0.381	0.319	1.084E-108	B cells	Ash1l
5.271E-113	0.2771357	0.453	0.395	1.307E-108	B cells	Arglu1
6.559E-112	0.36259453	0.336	0.281	1.626E-107	B cells	Zmat2
1.449E-110	0.3068453	0.46	0.405	3.592E-106	B cells	Eif2s2
4.843E-109	0.41712616	0.336	0.285	1.201E-104	B cells	Aimp1
1.489E-108	0.28033135	0.459	0.402	3.693E-104	B cells	Cct5
2.054E-108	0.31879081	0.377	0.319	5.093E-104	B cells	N4bp2l2
3.346E-108	0.27677114	0.445	0.39	8.296E-104	B cells	Dek
5.586E-108	0.40004802	0.272	0.218	1.385E-103	B cells	1110008P14R
1.757E-105	0.40191985	0.29	0.235	4.356E-101	B cells	Snrnp48
2.063E-105	0.3723878	0.275	0.221	5.115E-101	B cells	Spag9
1.164E-104	0.33706419	0.287	0.233	2.886E-100	B cells	Ncoa3
8.459E-103	0.30378918	0.387	0.331	2.0974E-98	B cells	Sltm
1.104E-102	0.29030194	0.377	0.321	2.7364E-98	B cells	Fkbp3
9.043E-102	0.25032007	0.291	0.233	2.2423E-97	B cells	Tmbim4
2.873E-100	0.42040965	0.32	0.271	7.1225E-96	B cells	Marc2
6.22E-100	0.49392067	0.263	0.216	1.5422E-95	B cells	Erp44
9.834E-100	0.30870468	0.407	0.357	2.4383E-95	B cells	Mrpl30
1.354E-98	0.2802864	0.428	0.376	3.3571E-94	B cells	Timm13
4.1621E-96	0.30697225	0.349	0.295	1.032E-91	B cells	Kansl1
7.4656E-96	0.4094876	0.296	0.249	1.8511E-91	B cells	Hspa4
1.351E-95	0.26758118	0.486	0.425	3.3499E-91	B cells	Sec11c
1.5045E-93	0.32625012	0.357	0.308	3.7303E-89	B cells	Mat2a
3.9062E-92	0.35653495	0.326	0.274	9.6854E-88	B cells	Akap9
4.5713E-92	0.28049514	0.407	0.357	1.1335E-87	B cells	Trmt112
2.2397E-91	0.32398094	0.347	0.298	5.5532E-87	B cells	Polr2e
4.2071E-91	0.3984953	0.252	0.203	1.0432E-86	B cells	Setd5
6.5622E-91	0.28172619	0.376	0.322	1.6271E-86	B cells	Tgfbr2
7.5704E-91	0.35749484	0.312	0.262	1.8771E-86	B cells	Fip1l1

2.0847E-90	0.30435925	0.303	0.251	5.169E-86	B cells	Rlim
4.8456E-90	0.32573443	0.308	0.256	1.2015E-85	B cells	Taf15
2.1369E-89	0.28645176	0.374	0.325	5.2984E-85	B cells	Khdrbs1
1.3087E-88	0.45065257	0.258	0.214	3.2448E-84	B cells	Rpl10a-ps1
1.4626E-88	0.32853893	0.312	0.261	3.6266E-84	B cells	Kdm5a
3.1494E-88	0.39458988	0.251	0.205	7.8089E-84	B cells	Emd
4.135E-88	0.25987166	0.38	0.329	1.0253E-83	B cells	Nsd1
1.6985E-87	0.28295876	0.396	0.349	4.2115E-83	B cells	Ndufa11
4.9411E-87	0.36368898	0.311	0.265	1.2251E-82	B cells	Pum1
1.4275E-86	0.33463207	0.318	0.272	3.5395E-82	B cells	Trappc6b
8.175E-86	0.34222637	0.338	0.292	2.027E-81	B cells	Cct3
1.1081E-85	0.35373523	0.314	0.266	2.7475E-81	B cells	Ranbp2
4.5972E-85	0.41638352	0.28	0.234	1.1399E-80	B cells	Dhx36
9.3477E-85	0.38867183	0.297	0.253	2.3178E-80	B cells	Lsm5
1.1405E-84	0.32493603	0.358	0.309	2.8278E-80	B cells	Bod1l
3.2788E-83	0.34494733	0.329	0.285	8.1298E-79	B cells	Banf1
9.3518E-83	0.40686242	0.254	0.21	2.3188E-78	B cells	Gemin7
4.7088E-82	0.36864213	0.325	0.284	1.1675E-77	B cells	Snrpd1
1.4535E-81	0.44605224	0.26	0.216	3.6039E-77	B cells	Eprs
1.0116E-79	0.33859192	0.318	0.272	2.5083E-75	B cells	Huwe1
3.2962E-79	0.26967376	0.404	0.362	8.1728E-75	B cells	Rtraf
1.7535E-78	0.42200099	0.309	0.27	4.3478E-74	B cells	Dnajb11
4.5588E-78	0.31320966	0.359	0.316	1.1304E-73	B cells	St13
1.9442E-77	0.31631951	0.299	0.252	4.8206E-73	B cells	6820431F20Ri
2.3261E-77	0.31416757	0.361	0.319	5.7676E-73	B cells	Mrpl52
2.515E-77	0.35085149	0.269	0.225	6.2361E-73	B cells	Chd1
4.0953E-77	0.40177011	0.26	0.215	1.0154E-72	B cells	Uhrf2
6.9628E-77	0.33054731	0.271	0.225	1.7264E-72	B cells	Ccnt1
3.1504E-76	0.57360281	0.283	0.242	7.8114E-72	B cells	Zbtb20
9.0099E-75	0.32290159	0.295	0.251	2.234E-70	B cells	Gpatch8
2.3068E-74	0.33168652	0.322	0.28	5.7197E-70	B cells	Ube2k
1.121E-73	0.2510152	0.419	0.375	2.7795E-69	B cells	Cct4
9.366E-72	0.43156166	0.25	0.21	2.3223E-67	B cells	Hadha
1.5569E-70	0.30704955	0.354	0.314	3.8602E-66	B cells	Uqcrfs1
4.0073E-70	0.35639399	0.299	0.26	9.9362E-66	B cells	Supt16
1.8911E-69	0.35168155	0.308	0.269	4.6891E-65	B cells	Dcun1d5
4.3844E-68	0.37150822	0.286	0.248	1.0871E-63	B cells	Anapc5
4.9637E-68	0.28371323	0.359	0.32	1.2307E-63	B cells	Cacybp
3.524E-67	0.31384788	0.33	0.292	8.7377E-63	B cells	1110004F10Ri
1.004E-66	0.38272003	0.256	0.217	2.4894E-62	B cells	Ntan1
4.7915E-66	0.36602876	0.269	0.231	1.1881E-61	B cells	Prmt1
4.9336E-66	0.25087158	0.309	0.267	1.2233E-61	B cells	Eif2s3y
9.6138E-65	0.32691285	0.286	0.248	2.3837E-60	B cells	Caprin1
1.7552E-64	0.25598144	0.37	0.33	4.352E-60	B cells	Rbbp4
3.8321E-64	0.31556622	0.257	0.217	9.5017E-60	B cells	Suz12
5.6064E-64	0.29591742	0.351	0.315	1.3901E-59	B cells	Mdh1
2.3771E-62	0.40155087	0.256	0.222	5.894E-58	B cells	Mrpl54
3.2149E-62	0.26000998	0.339	0.301	7.9714E-58	B cells	Imp3
2.2948E-61	0.30704207	0.255	0.216	5.6899E-57	B cells	Vbp1
2.6769E-61	0.29538862	0.302	0.265	6.6374E-57	B cells	Mrpl43

8.6535E-61	0.25062295	0.374	0.334	2.1456E-56	B cells	Pbrm1
6.0142E-60	0.35045526	0.26	0.224	1.4912E-55	B cells	Prpf8
8.3692E-60	0.263057	0.325	0.288	2.0751E-55	B cells	Mpc1
8.3947E-60	0.28141943	0.253	0.214	2.0815E-55	B cells	Dcaf8
1.2093E-59	0.30110182	0.277	0.239	2.9986E-55	B cells	Eif4a3
3.9635E-59	0.28067948	0.296	0.258	9.8275E-55	B cells	Ccar1
1.1789E-58	0.30519932	0.301	0.265	2.9231E-54	B cells	Psmd11
1.996E-57	0.35705156	0.28	0.248	4.949E-53	B cells	Psmc5
2.7884E-57	0.29575269	0.298	0.263	6.9139E-53	B cells	Ctbp1
2.8723E-55	0.28477727	0.328	0.292	7.122E-51	B cells	Ube3a
8.5827E-55	0.34898875	0.289	0.257	2.1281E-50	B cells	Chchd1
6.4392E-54	0.28467091	0.283	0.246	1.5966E-49	B cells	Taf1d
7.1358E-54	0.29564909	0.29	0.256	1.7693E-49	B cells	Fkbp4
2.0066E-52	0.26475105	0.33	0.298	4.9754E-48	B cells	Srsf6
1.014E-51	0.32648868	0.255	0.222	2.5142E-47	B cells	U2af2
3.3015E-49	0.26163928	0.338	0.305	8.1861E-45	B cells	Ranbp1
8.2385E-48	0.29537803	0.284	0.254	2.0427E-43	B cells	Polr2m
1.133E-47	0.25127556	0.292	0.259	2.8092E-43	B cells	Stub1
2.8742E-47	0.27930304	0.271	0.238	7.1265E-43	B cells	Ubn2
2.5856E-45	0.28958669	0.311	0.281	6.4109E-41	B cells	Hspa14
3.9965E-45	0.26905751	0.279	0.247	9.9094E-41	B cells	Baz1b
3.1067E-43	0.29207887	0.275	0.245	7.703E-39	B cells	Ik
4.3628E-40	0.33095927	0.262	0.237	1.0817E-35	B cells	Eif2s1
4.4447E-40	0.3182845	0.271	0.245	1.1021E-35	B cells	Cyc1
1.1916E-39	0.26479705	0.261	0.231	2.9547E-35	B cells	Zranb2
2.7365E-34	0.29136467	0.251	0.226	6.7852E-30	B cells	Rps10-ps1
1.0574E-33	0.25371659	0.264	0.238	2.6218E-29	B cells	Fxr1
7.1889E-33	0.25488782	0.274	0.249	1.7825E-28	B cells	Trp53
1.4906E-32	0.3202303	0.268	0.247	3.6959E-28	B cells	Chrac1
6.907E-30	0.27998477	0.267	0.246	1.7126E-25	B cells	Romo1
4.5575E-29	0.26790577	0.254	0.232	1.13E-24	B cells	Mbd3
8.6979E-26	0.26809593	0.251	0.23	2.1566E-21	B cells	Psmc4
0	5.94476261	0.892	0.034	0	Monocytes	Clec4a3
0	3.88070416	0.904	0.092	0	Monocytes	Csf1r
0	6.16256243	0.818	0.02	0	Monocytes	Clec4a1
0	4.57410654	0.883	0.088	0	Monocytes	Ms4a6c
0	4.05408928	0.958	0.172	0	Monocytes	Ifitm3
0	6.59008742	0.77	0.014	0	Monocytes	Ear2
0	3.42282275	0.856	0.1	0	Monocytes	Trf
0	3.64003919	0.969	0.226	0	Monocytes	Cybb
0	4.93042013	0.777	0.035	0	Monocytes	Lrp1
0	4.97375366	0.756	0.031	0	Monocytes	Cx3cr1
0	3.21158952	0.865	0.143	0	Monocytes	Pld4
0	3.82509721	0.785	0.069	0	Monocytes	Ifitm6
0	3.10277847	0.819	0.104	0	Monocytes	Mpeg1
0	3.0149249	0.902	0.19	0	Monocytes	Zeb2
0	2.77259516	0.828	0.117	0	Monocytes	Sirpa
0	3.66926289	0.818	0.107	0	Monocytes	Tnfrsf1b
0	2.30663948	0.859	0.153	0	Monocytes	Cd300a
0	1.87342773	0.897	0.193	0	Monocytes	Lst1

0	1.43677537	0.944	0.25	0	Monocytes	Ifitm2
0	3.47314704	0.778	0.086	0	Monocytes	Cd68
0	6.63437832	0.709	0.02	0	Monocytes	Adgre4
0	2.72218394	0.759	0.102	0	Monocytes	Cd300c2
0	3.04456213	0.926	0.271	0	Monocytes	Ctsb
0	0.76271809	0.878	0.224	0	Monocytes	Ccl6
0	2.56872445	0.797	0.144	0	Monocytes	Metrnl
0	3.54274768	0.69	0.045	0	Monocytes	Gpr141
0	2.24675508	0.966	0.325	0	Monocytes	Fcer1g
0	2.7105493	0.749	0.109	0	Monocytes	Plbd1
0	1.18832678	0.893	0.255	0	Monocytes	Alox5ap
0	2.54785984	0.771	0.147	0	Monocytes	Hck
0	7.76184732	0.632	0.008	0	Monocytes	Ace
0	3.32907163	0.729	0.108	0	Monocytes	Klf4
0	2.72309277	0.843	0.23	0	Monocytes	Nr4a1
0	2.66551367	0.846	0.237	0	Monocytes	Smpdl3a
0	3.75666381	0.68	0.072	0	Monocytes	Ccdc88a
0	1.62270063	0.741	0.133	0	Monocytes	Emilin2
0	0.94834331	0.786	0.187	0	Monocytes	Pla2g7
0	3.82630108	0.767	0.169	0	Monocytes	Gngt2
0	3.03093149	0.878	0.282	0	Monocytes	Plac8
0	3.07938706	0.993	0.403	0	Monocytes	Lyz2
0	1.66096528	0.756	0.168	0	Monocytes	Fgr
0	3.36290242	0.668	0.087	0	Monocytes	Rassf4
0	1.82881123	0.676	0.096	0	Monocytes	Sirpb1c
0	1.84860034	0.825	0.245	0	Monocytes	Nadk
0	3.67256683	0.663	0.087	0	Monocytes	Rras
0	1.11444125	0.971	0.397	0	Monocytes	Tyrobp
0	5.43905673	0.589	0.015	0	Monocytes	Treml4
0	1.93062273	0.787	0.217	0	Monocytes	S100a4
0	6.48805414	0.577	0.008	0	Monocytes	Ear1
0	4.71246532	0.606	0.039	0	Monocytes	Plxnb2
0	2.81982094	0.613	0.052	0	Monocytes	Ccl9
0	2.03086978	0.786	0.23	0	Monocytes	Prkcd
0	2.82691434	0.606	0.052	0	Monocytes	Adgre1
0	1.27109388	0.828	0.276	0	Monocytes	Spi1
0	1.288364	0.718	0.168	0	Monocytes	Itgam
0	2.52484613	0.671	0.122	0	Monocytes	Trps1
0	1.09873882	0.736	0.188	0	Monocytes	Nfam1
0	2.5270762	0.695	0.148	0	Monocytes	Irf5
0	1.63580315	0.668	0.125	0	Monocytes	Igsf6
0	1.11909992	0.707	0.164	0	Monocytes	Fcgr3
0	2.389252	0.882	0.34	0	Monocytes	Lamp1
0	5.2367141	0.565	0.025	0	Monocytes	Ptpro
0	2.17848057	0.656	0.117	0	Monocytes	Csf2ra
0	6.12445888	0.555	0.017	0	Monocytes	Grk3
0	3.95796306	0.59	0.052	0	Monocytes	Acer3
0	2.32506462	0.678	0.143	0	Monocytes	Ap1s2
0	2.57063874	0.735	0.2	0	Monocytes	Myo1g
0	1.66371993	0.773	0.239	0	Monocytes	Ptpre

0	1.87554653	0.814	0.291	0	Monocytes	Ctsa
0	2.02425998	0.93	0.408	0	Monocytes	Gm2a
0	1.76340659	0.71	0.19	0	Monocytes	Creg1
0	2.40111672	0.632	0.115	0	Monocytes	Bach1
0	1.67098234	0.736	0.22	0	Monocytes	Il6ra
0	0.89604743	0.807	0.292	0	Monocytes	Ncf2
0	0.65366702	0.804	0.29	0	Monocytes	Tpd52
0	2.27259624	0.867	0.353	0	Monocytes	Pou2f2
0	4.57923301	0.54	0.027	0	Monocytes	Pid1
0	1.87538873	0.617	0.104	0	Monocytes	Grn
0	4.76054208	0.533	0.024	0	Monocytes	Tifab
0	1.90477624	0.959	0.45	0	Monocytes	Cebpb
0	2.0516697	0.605	0.097	0	Monocytes	App
0	2.34408446	0.941	0.434	0	Monocytes	Ctss
0	1.60687676	0.836	0.329	0	Monocytes	Lyn
0	1.48057571	0.73	0.228	0	Monocytes	Lamp2
0	2.09820002	0.9	0.399	0	Monocytes	Lgals3
0	1.33259916	0.67	0.171	0	Monocytes	Tnfrsf1a
0	0.3466008	0.728	0.229	0	Monocytes	Marcks
0	5.59376572	0.516	0.017	0	Monocytes	Gm21188
0	1.9559571	0.669	0.17	0	Monocytes	Selenop
0	2.84567677	0.599	0.101	0	Monocytes	Fam49a
0	2.49427497	0.562	0.066	0	Monocytes	Fgd4
0	1.61168046	0.864	0.371	0	Monocytes	Ctsz
0	1.63245603	0.591	0.099	0	Monocytes	Mgst1
0	0.77813522	0.828	0.336	0	Monocytes	Cd44
0	4.22839674	0.519	0.029	0	Monocytes	Nupr1
0	1.39542148	0.714	0.225	0	Monocytes	Myo1f
0	2.92854086	0.565	0.076	0	Monocytes	Plin2
0	1.79273038	0.688	0.2	0	Monocytes	Xbp1
0	2.0353623	0.762	0.277	0	Monocytes	Atp1a1
0	1.93701485	0.843	0.358	0	Monocytes	Itgal
0	1.43477519	0.805	0.321	0	Monocytes	Il17ra
0	2.90357628	0.536	0.053	0	Monocytes	Cd177
0	2.60522037	0.594	0.112	0	Monocytes	Ptprj
0	2.18977243	0.646	0.164	0	Monocytes	Sh3bgrl
0	2.16497095	0.69	0.209	0	Monocytes	Anxa5
0	4.36156367	0.507	0.027	0	Monocytes	Cd300ld
0	5.48004796	0.501	0.021	0	Monocytes	Hfe
0	1.8235436	0.906	0.427	0	Monocytes	Ifngr1
0	1.88602407	0.779	0.3	0	Monocytes	Laptm4a
0	2.00192206	0.666	0.188	0	Monocytes	Mapkapk2
0	2.86558932	0.541	0.065	0	Monocytes	Adssl1
0	1.49946174	0.737	0.262	0	Monocytes	Rnf130
0	2.95564836	0.56	0.087	0	Monocytes	Camkk2
0	2.56052479	0.581	0.108	0	Monocytes	BC028528
0	7.1838853	0.477	0.005	0	Monocytes	Zfyve9
0	2.34108362	0.562	0.092	0	Monocytes	Cdkn1a
0	1.79228178	0.885	0.416	0	Monocytes	Flna
0	1.84350692	0.734	0.265	0	Monocytes	Glud1

0	1.69037875	0.642	0.174	0	Monocytes	Hexa
0	1.83956255	0.816	0.348	0	Monocytes	Ahnak
0	1.75081915	0.726	0.259	0	Monocytes	Mef2a
0	4.69553125	0.5	0.033	0	Monocytes	Ms4a6d
0	2.73419542	0.568	0.104	0	Monocytes	Cbfa2t3
0	1.48402952	0.561	0.098	0	Monocytes	Gm50022
0	3.87408517	0.503	0.04	0	Monocytes	Krt80
0	1.49392848	0.617	0.154	0	Monocytes	Fam129a
0	2.31805078	0.935	0.477	0	Monocytes	Ly6e
0	2.28261908	0.613	0.156	0	Monocytes	Sgk1
0	1.64783676	0.69	0.234	0	Monocytes	Pip4k2a
0	0.72868961	0.821	0.365	0	Monocytes	Napsa
0	3.09301225	0.516	0.061	0	Monocytes	Plod3
0	1.35729678	0.734	0.279	0	Monocytes	Efhd2
0	1.29900592	0.695	0.241	0	Monocytes	Tmcc1
0	1.14841617	0.773	0.32	0	Monocytes	Ctsh
0	1.61421033	0.653	0.201	0	Monocytes	Nfe2l2
0	4.53949497	0.488	0.037	0	Monocytes	Fcgr4
0	1.28884081	0.691	0.24	0	Monocytes	Aldh2
0	1.8370255	0.542	0.093	0	Monocytes	Cd93
0	2.24644288	0.549	0.1	0	Monocytes	Zfp710
0	1.34718933	0.608	0.16	0	Monocytes	Tgfbi
0	3.00034025	0.986	0.539	0	Monocytes	Psap
0	2.09646264	0.586	0.14	0	Monocytes	Soat1
0	6.29554004	0.454	0.009	0	Monocytes	Ldlrad3
0	2.36478127	0.513	0.07	0	Monocytes	Slc11a1
0	1.14924035	0.664	0.222	0	Monocytes	Rara
0	1.52184342	0.618	0.176	0	Monocytes	Ifngr2
0	1.80557396	0.652	0.215	0	Monocytes	Tent5a
0	2.34092113	0.498	0.061	0	Monocytes	Clec4a2
0	1.85438338	0.758	0.321	0	Monocytes	Stk10
0	1.88933789	0.605	0.169	0	Monocytes	Ehd4
0	5.7367893	0.448	0.013	0	Monocytes	Arhgef10l
0	1.7592314	0.587	0.152	0	Monocytes	Plekho2
0	1.5644291	0.723	0.29	0	Monocytes	Tab2
0	2.31786649	0.483	0.053	0	Monocytes	Il13ra1
0	5.84478765	0.448	0.018	0	Monocytes	Slc12a2
0	1.7936997	0.58	0.153	0	Monocytes	Nrros
0	1.43843284	0.665	0.239	0	Monocytes	Tm6sf1
0	1.35999062	0.534	0.108	0	Monocytes	Lrrc25
0	2.25813582	0.521	0.097	0	Monocytes	Apobec1
0	1.99162822	0.502	0.078	0	Monocytes	Atp1a3
0	1.2765658	0.865	0.441	0	Monocytes	Itgb2
0	1.45304541	0.778	0.355	0	Monocytes	Ikbkb
0	5.47764681	0.446	0.023	0	Monocytes	Eno3
0	7.520352	0.427	0.004	0	Monocytes	Cd300e
0	1.54943995	0.66	0.238	0	Monocytes	Samsn1
0	4.44915222	0.451	0.03	0	Monocytes	Nav1
0	1.42319974	0.911	0.49	0	Monocytes	Fyb
0	4.29254002	0.443	0.022	0	Monocytes	Tnfrsf21

0	4.75617922	0.443	0.023	0	Monocytes	Serpinb10
0	1.58831868	0.69	0.271	0	Monocytes	Dbi
0	1.71381886	0.57	0.153	0	Monocytes	Mapk3
0	2.11055208	0.541	0.127	0	Monocytes	Il10ra
0	0.53066881	0.802	0.389	0	Monocytes	Anxa2
0	5.41238912	0.435	0.022	0	Monocytes	Tmem51
0	3.76984484	0.449	0.037	0	Monocytes	Tppp3
0	2.24496485	0.509	0.098	0	Monocytes	Asah1
0	1.54340242	0.813	0.402	0	Monocytes	Atox1
0	1.00311855	0.624	0.216	0	Monocytes	Skap2
0	1.48306833	0.644	0.236	0	Monocytes	Syngr2
0	4.31689012	0.433	0.027	0	Monocytes	Cd302
0	2.53589417	0.48	0.076	0	Monocytes	Naaa
0	1.23808915	0.627	0.224	0	Monocytes	Dleu2
0	1.35689611	0.572	0.17	0	Monocytes	Rnase6
0	1.44399595	0.671	0.271	0	Monocytes	Degs1
0	2.99818302	0.456	0.056	0	Monocytes	Lair1
0	1.15920486	0.616	0.216	0	Monocytes	Ttc7
0	0.62651159	0.719	0.319	0	Monocytes	Itgb1
0	0.52086988	0.624	0.225	0	Monocytes	Myadm
0	6.96472472	0.402	0.004	0	Monocytes	Hpgd
0	1.12885725	0.847	0.451	0	Monocytes	Itga4
0	0.71730492	0.706	0.311	0	Monocytes	Pglyrp1
0	2.25423217	0.475	0.08	0	Monocytes	Nfil3
0	0.90016007	0.683	0.289	0	Monocytes	Ly86
0	1.47563875	0.6	0.208	0	Monocytes	Otulinl
0	1.11712387	0.661	0.269	0	Monocytes	Mbp
0	1.78058237	0.483	0.094	0	Monocytes	Atf3
0	1.29885136	0.718	0.329	0	Monocytes	Nfkb1
0	2.73304943	0.422	0.034	0	Monocytes	Gm9733
0	1.66132514	0.537	0.15	0	Monocytes	Sgk3
0	0.9034297	0.569	0.182	0	Monocytes	Eif4ebp1
0	1.58189525	0.9	0.514	0	Monocytes	Sat1
0	3.82037995	0.426	0.04	0	Monocytes	Rbpms
0	1.31309312	0.821	0.435	0	Monocytes	Atp2b1
0	1.71518051	0.491	0.106	0	Monocytes	Dstn
0	2.45662935	0.485	0.1	0	Monocytes	Hip1
0	1.2315381	0.665	0.28	0	Monocytes	Tmpo
0	0.88721401	0.557	0.173	0	Monocytes	Pirb
0	3.25851062	0.45	0.066	0	Monocytes	Bcl2a1d
0	1.04693933	0.908	0.525	0	Monocytes	Adgre5
0	2.34232288	0.939	0.557	0	Monocytes	Gpx1
0	0.73549568	0.591	0.211	0	Monocytes	Cyp4f18
0	3.5557619	0.409	0.03	0	Monocytes	Gpr35
0	4.38626691	0.407	0.028	0	Monocytes	Scarb1
0	1.27323944	0.669	0.291	0	Monocytes	Rrbp1
0	1.01183052	0.541	0.163	0	Monocytes	Gm45223
0	1.22870071	0.64	0.262	0	Monocytes	Qk
0	0.53819093	0.816	0.439	0	Monocytes	Rgs2
0	2.44046335	0.442	0.066	0	Monocytes	Csgalnact2

0	1.19855113	0.802	0.429	0	Monocytes	Atp6v0b
0	7.52539131	0.376	0.003	0	Monocytes	Lilra5
0	0.69743429	0.787	0.415	0	Monocytes	Man2b1
0	1.2466648	0.679	0.307	0	Monocytes	Tkt
0	1.42559562	0.849	0.477	0	Monocytes	Rap1a
0	1.31491696	0.633	0.262	0	Monocytes	Sppl2a
0	1.8442679	0.46	0.09	0	Monocytes	Fn1
0	0.92765824	0.673	0.304	0	Monocytes	Vsir
0	0.86825162	0.659	0.29	0	Monocytes	Id2
0	5.28177343	0.383	0.014	0	Monocytes	F13a1
0	0.85857295	0.674	0.306	0	Monocytes	Unc93b1
0	5.85229393	0.38	0.012	0	Monocytes	Chil3
0	1.14466697	0.538	0.17	0	Monocytes	Svil
0	4.42928402	0.403	0.036	0	Monocytes	Apoe
0	2.54294078	0.462	0.095	0	Monocytes	Abhd12
0	1.48419592	0.577	0.211	0	Monocytes	Ssr3
0	1.54770949	0.563	0.197	0	Monocytes	P4hb
0	3.45151347	0.394	0.029	0	Monocytes	Ppfia4
0	1.18036928	0.602	0.237	0	Monocytes	Atp6ap1
0	0.82335114	0.612	0.247	0	Monocytes	Rassf3
0	1.17079672	0.553	0.189	0	Monocytes	Lmo4
0	3.64636637	0.396	0.032	0	Monocytes	Cfh
0	1.02410373	0.744	0.38	0	Monocytes	Mtpn
0	2.52347128	0.412	0.048	0	Monocytes	C3
0	1.28443194	0.705	0.341	0	Monocytes	BC005537
0	1.19050599	0.785	0.422	0	Monocytes	Pomp
0	1.03810372	0.746	0.383	0	Monocytes	Ubl3
0	1.08049123	0.732	0.37	0	Monocytes	Bri3
0	1.19695012	0.5	0.138	0	Monocytes	Dennd5a
0	2.49310635	0.468	0.107	0	Monocytes	Abi3
0	3.74747655	0.387	0.026	0	Monocytes	Hebp1
0	2.79219989	0.418	0.057	0	Monocytes	Rap2a
0	1.20600039	0.78	0.42	0	Monocytes	Erp29
0	2.73867241	0.401	0.041	0	Monocytes	Clec12a
0	1.11687166	0.516	0.156	0	Monocytes	Cd244a
0	1.14826109	0.532	0.174	0	Monocytes	Atp6v1b2
0	0.66096183	0.5	0.142	0	Monocytes	Ogfrl1
0	2.83890721	0.411	0.053	0	Monocytes	Gm37420
0	2.23589982	0.413	0.056	0	Monocytes	6430548M08R
0	2.03357787	0.469	0.113	0	Monocytes	Bri3bp
0	1.68501006	0.479	0.123	0	Monocytes	Lyl1
0	1.30329961	0.546	0.191	0	Monocytes	Akt1
0	2.20609798	0.453	0.098	0	Monocytes	Atf6
0	2.85775555	0.405	0.051	0	Monocytes	Rap1gap2
0	1.08982148	0.646	0.292	0	Monocytes	Filip1l
0	1.32693585	0.551	0.197	0	Monocytes	Sik1
0	2.82558217	0.414	0.06	0	Monocytes	Elmo2
0	0.61062866	0.498	0.144	0	Monocytes	Wfdc17
0	1.40067887	0.562	0.209	0	Monocytes	Lims1
0	0.48690837	0.773	0.42	0	Monocytes	Picalm

0	1.18454215	0.67	0.317	0	Monocytes	Canx
0	3.76509012	0.383	0.03	0	Monocytes	Tlr7
0	1.09638293	0.471	0.118	0	Monocytes	Fes
0	0.72513112	0.632	0.279	0	Monocytes	Plek
0	0.87922814	0.479	0.127	0	Monocytes	Csf2rb
0	1.26435614	0.664	0.313	0	Monocytes	Tmed5
0	1.75543745	0.621	0.27	0	Monocytes	Ctsc
0	1.42332638	0.509	0.158	0	Monocytes	Plcg2
0	1.62333125	0.534	0.184	0	Monocytes	Magt1
0	0.86657303	0.761	0.411	0	Monocytes	Ncf4
0	1.23890075	0.59	0.24	0	Monocytes	Zmiz1
0	1.75476008	0.465	0.116	0	Monocytes	Ehbp1l1
0	0.39596025	0.752	0.403	0	Monocytes	Syk
0	1.51957128	0.632	0.284	0	Monocytes	Tgfb1
0	1.26200109	0.838	0.49	0	Monocytes	Capza2
0	1.69712538	0.604	0.256	0	Monocytes	Spn
0	1.80590221	0.482	0.134	0	Monocytes	Susd3
0	1.02464696	0.679	0.332	0	Monocytes	Mob1a
0	1.60654526	0.465	0.118	0	Monocytes	5031439G07R
0	2.21171385	0.441	0.094	0	Monocytes	Inpp5f
0	3.82689411	0.378	0.031	0	Monocytes	S1pr5
0	4.53196717	0.364	0.017	0	Monocytes	Bcl2a1a
0	1.24366084	0.571	0.224	0	Monocytes	Sept9
0	1.58213756	0.518	0.172	0	Monocytes	Jarid2
0	0.73996725	0.846	0.5	0	Monocytes	Pim1
0	0.92119213	0.677	0.332	0	Monocytes	Fmnl1
0	1.44899687	0.53	0.185	0	Monocytes	Slk
0	0.75144271	0.894	0.549	0	Monocytes	Zfp36
0	1.34195879	0.542	0.198	0	Monocytes	Fam174a
0	0.84807068	0.706	0.363	0	Monocytes	Kdm7a
0	2.25342578	0.436	0.094	0	Monocytes	Tgfbr1
0	1.37261175	0.511	0.169	0	Monocytes	Fcho2
0	1.23802961	0.842	0.5	0	Monocytes	Prdx5
0	1.55308401	0.483	0.142	0	Monocytes	Ppp1r15b
0	3.8607439	0.367	0.026	0	Monocytes	Mafb
0	4.73471832	0.371	0.03	0	Monocytes	Cd36
0	1.9420803	0.45	0.11	0	Monocytes	Gusb
0	0.90721959	0.62	0.28	0	Monocytes	Ifi30
0	1.02293414	0.622	0.283	0	Monocytes	Zfand5
0	1.85033762	0.427	0.089	0	Monocytes	G6pdx
0	1.70278389	0.432	0.095	0	Monocytes	Cers6
0	1.2815686	0.892	0.555	0	Monocytes	Samhd1
0	3.0003619	0.405	0.069	0	Monocytes	Agpat4
0	1.38412427	0.539	0.204	0	Monocytes	Nab1
0	1.99063296	0.416	0.081	0	Monocytes	Rnpep
0	1.50316088	0.488	0.153	0	Monocytes	Adam17
0	1.09424635	0.454	0.12	0	Monocytes	Pilra
0	0.90253206	0.657	0.323	0	Monocytes	Cbl
0	1.23815036	0.526	0.192	0	Monocytes	Evi2a
0	1.03371266	0.516	0.182	0	Monocytes	Mapk14

0	2.13048409	0.399	0.065	0	Monocytes	Xdh
0	1.14012701	0.469	0.136	0	Monocytes	Themis2
0	0.96440157	0.783	0.451	0	Monocytes	Ptpn1
0	1.22500378	0.468	0.136	0	Monocytes	Dusp6
0	1.21315265	0.46	0.128	0	Monocytes	St3gal4
0	1.003793	0.572	0.24	0	Monocytes	Asap1
0	0.86603254	0.663	0.332	0	Monocytes	Rab5if
0	0.74220069	0.699	0.371	0	Monocytes	Rbms1
0	2.03480068	0.376	0.049	0	Monocytes	Tlr13
0	0.96586716	0.523	0.196	0	Monocytes	N4bp1
0	0.98140441	0.506	0.179	0	Monocytes	Dok3
0	2.10657128	0.412	0.085	0	Monocytes	Lrrc8d
0	2.17937055	0.41	0.083	0	Monocytes	Lgmn
0	1.06810611	0.55	0.224	0	Monocytes	Nckap1l
0	5.02721386	0.347	0.021	0	Monocytes	Smpdl3b
0	0.383072	0.431	0.106	0	Monocytes	Gm5150
0	0.90369663	0.691	0.366	0	Monocytes	Capns1
0	4.09945638	0.344	0.019	0	Monocytes	Sowahc
0	0.87109905	0.827	0.502	0	Monocytes	Prr13
0	1.10177122	0.682	0.358	0	Monocytes	Akr1a1
0	0.50987368	0.683	0.359	0	Monocytes	Rhog
0	1.05111927	0.624	0.3	0	Monocytes	Srsf9
0	0.88610651	0.737	0.413	0	Monocytes	Tmed10
0	1.62965867	0.458	0.134	0	Monocytes	Siva1
0	1.93701122	0.433	0.11	0	Monocytes	Ddi2
0	3.28766983	0.361	0.038	0	Monocytes	Dusp16
0	1.89690507	0.416	0.093	0	Monocytes	Gsr
0	1.25090491	0.493	0.171	0	Monocytes	Wsb1
0	1.76423196	0.384	0.064	0	Monocytes	Rab32
0	1.55666187	0.522	0.202	0	Monocytes	Ms4a4c
0	0.86810455	0.69	0.37	0	Monocytes	Stk38
0	1.84074996	0.392	0.073	0	Monocytes	Sqor
0	0.76319266	0.647	0.328	0	Monocytes	Stat3
0	1.92494615	0.384	0.066	0	Monocytes	Ralb
0	2.14447008	0.397	0.079	0	Monocytes	Rp2
0	0.59918605	0.54	0.223	0	Monocytes	Ppt1
0	0.89687279	0.532	0.215	0	Monocytes	Slc6a6
0	1.71524468	0.406	0.089	0	Monocytes	Sap30
0	3.50497151	0.342	0.025	0	Monocytes	Msr1
0	1.05998716	0.564	0.247	0	Monocytes	Bnip2
0	1.66210872	0.447	0.131	0	Monocytes	Ap2a2
0	0.65760134	0.807	0.492	0	Monocytes	Ptpn6
0	0.96769413	0.599	0.284	0	Monocytes	Tor1aip1
0	1.32417885	0.447	0.132	0	Monocytes	Arap1
0	2.67190668	0.382	0.067	0	Monocytes	Naga
0	0.90465113	0.576	0.261	0	Monocytes	Nptn
0	0.77868995	0.783	0.469	0	Monocytes	Ppp2r5a
0	0.89598267	0.604	0.29	0	Monocytes	Mia2
0	1.28850196	0.492	0.178	0	Monocytes	Cnpy3
0	5.06654529	0.33	0.016	0	Monocytes	Colec12

0	1.94394526	0.411	0.098	0	Monocytes	Gyg
0	3.87581592	0.33	0.018	0	Monocytes	Dna2
0	1.05215489	0.554	0.243	0	Monocytes	Ppp2r5c
0	1.35145027	0.452	0.142	0	Monocytes	Hsd17b11
0	1.21089885	0.523	0.213	0	Monocytes	Rnh1
0	1.15374733	0.886	0.577	0	Monocytes	Tln1
0	1.60832329	0.428	0.119	0	Monocytes	Tet2
0	2.06248074	0.367	0.058	0	Monocytes	Tmem38b
0	1.17980286	0.559	0.251	0	Monocytes	Ciao2a
0	1.61231877	0.462	0.154	0	Monocytes	Tmem256
0	2.37982022	0.361	0.053	0	Monocytes	Bmpr2
0	2.74220664	0.344	0.036	0	Monocytes	Gstm1
0	4.54373698	0.324	0.016	0	Monocytes	P2ry6
0	1.0259442	0.59	0.282	0	Monocytes	Pgam1
0	0.82473354	0.413	0.106	0	Monocytes	Hacd4
0	0.40083381	0.589	0.282	0	Monocytes	Kctd12
0	1.31326411	0.486	0.179	0	Monocytes	Nin
0	0.78107297	0.734	0.427	0	Monocytes	Diaph1
0	1.09075411	0.802	0.496	0	Monocytes	Prdx1
0	1.49559189	0.467	0.161	0	Monocytes	Ywhag
0	2.61180645	0.366	0.06	0	Monocytes	Dusp3
0	1.31088877	0.427	0.121	0	Monocytes	Synj1
0	1.00353277	0.559	0.254	0	Monocytes	Cd2ap
0	1.00524849	0.542	0.238	0	Monocytes	Epn1
0	1.69740816	0.451	0.147	0	Monocytes	Pik3ap1
0	4.62204594	0.321	0.017	0	Monocytes	Ifi202b
0	0.71528423	0.47	0.166	0	Monocytes	Fosl2
0	1.14881536	0.523	0.22	0	Monocytes	Eif4g3
0	1.18697309	0.518	0.215	0	Monocytes	Ccdc50
0	1.33482424	0.466	0.163	0	Monocytes	Ifnar2
0	0.85482863	0.721	0.418	0	Monocytes	Smdt1
0	2.16700857	0.38	0.078	0	Monocytes	Gm37531
0	2.16616467	0.379	0.077	0	Monocytes	Diaph2
0	0.80227397	0.644	0.342	0	Monocytes	Rps27l
0	1.06336409	0.436	0.134	0	Monocytes	Mfsd14b
0	5.83832351	0.309	0.007	0	Monocytes	Slc8a1
0	0.71796902	0.635	0.334	0	Monocytes	Ddx3x
0	0.98631507	0.588	0.287	0	Monocytes	Pgk1
0	0.85652623	0.648	0.349	0	Monocytes	Wdr26
0	0.85918998	0.554	0.255	0	Monocytes	Herc4
0	3.92105104	0.321	0.022	0	Monocytes	Slc7a7
0	0.80856816	0.84	0.541	0	Monocytes	Hsp90b1
0	1.00649717	0.568	0.269	0	Monocytes	Slc44a2
0	2.41872002	0.375	0.077	0	Monocytes	Htra2
0	1.0375396	0.592	0.294	0	Monocytes	Zbtb7a
0	2.29835463	0.353	0.055	0	Monocytes	Milr1
0	1.05223493	0.52	0.223	0	Monocytes	Xiap
0	2.49163459	0.331	0.034	0	Monocytes	Ltbr
0	1.94812894	0.449	0.152	0	Monocytes	Ccr2
0	1.7109846	0.396	0.099	0	Monocytes	Slc43a2

0	0.90329562	0.511	0.214	0	Monocytes	Nfkbiz
0	0.98747827	0.499	0.202	0	Monocytes	Cnih4
0	4.56928476	0.312	0.015	0	Monocytes	Havcr2
0	1.0466916	0.801	0.505	0	Monocytes	Psma7
0	1.42911656	0.448	0.152	0	Monocytes	Atp2c1
0	0.81003858	0.374	0.078	0	Monocytes	Sirpb1b
0	0.9597751	0.601	0.305	0	Monocytes	Pdia6
0	1.1405481	0.446	0.151	0	Monocytes	Mpp1
0	0.77070815	0.656	0.361	0	Monocytes	Cmtm7
0	0.65072245	0.551	0.256	0	Monocytes	Lcp2
0	1.41113439	0.429	0.134	0	Monocytes	Hps3
0	0.76943783	0.597	0.302	0	Monocytes	Spop
0	5.59977974	0.3	0.005	0	Monocytes	Itgb5
0	4.15392974	0.31	0.015	0	Monocytes	Aif1
0	1.33730513	0.572	0.277	0	Monocytes	Aprt
0	0.82965698	0.791	0.497	0	Monocytes	Snrpb
0	1.04103455	0.519	0.225	0	Monocytes	Rbm7
0	2.65227915	0.344	0.05	0	Monocytes	Adap1
0	1.47820198	0.434	0.14	0	Monocytes	Lipa
0	0.68267599	0.533	0.24	0	Monocytes	Cdc42ep3
0	1.1235098	0.483	0.19	0	Monocytes	Itm2c
0	1.76365953	0.404	0.112	0	Monocytes	Ncor2
0	0.91322953	0.504	0.212	0	Monocytes	Stx7
0	0.92148719	0.529	0.237	0	Monocytes	Aplp2
0	1.65886007	0.425	0.133	0	Monocytes	Chmp2b
0	1.97130744	0.387	0.096	0	Monocytes	B4galt5
0	1.32112562	0.54	0.249	0	Monocytes	Cd9
0	0.84052098	0.596	0.305	0	Monocytes	Ap2s1
0	1.66056537	0.418	0.127	0	Monocytes	Fam168a
0	0.61937158	0.876	0.586	0	Monocytes	Serinc3
0	0.64272035	0.811	0.521	0	Monocytes	Sh3glb1
0	1.88485747	0.38	0.09	0	Monocytes	Trafd1
0	2.01178706	0.381	0.091	0	Monocytes	Ddhd1
0	0.88721791	0.773	0.483	0	Monocytes	Pitpna
0	2.70972815	0.333	0.043	0	Monocytes	Naip2
0	4.00487677	0.316	0.026	0	Monocytes	Tcf7l2
0	0.79008098	0.67	0.381	0	Monocytes	Gnb1
0	0.89033601	0.552	0.263	0	Monocytes	Gltp
0	1.20144129	0.806	0.517	0	Monocytes	Npc2
0	0.43068895	0.771	0.484	0	Monocytes	Mrpl33
0	0.8888089	0.547	0.26	0	Monocytes	Aup1
0	0.74585272	0.619	0.332	0	Monocytes	Rassf5
0	1.62695652	0.408	0.121	0	Monocytes	Slc15a4
0	0.88579637	0.561	0.275	0	Monocytes	Taok3
0	0.45296921	0.569	0.283	0	Monocytes	Plaur
0	0.71993799	0.702	0.416	0	Monocytes	Ywhae
0	0.74488783	0.677	0.392	0	Monocytes	Arpc4
0	1.1881011	0.422	0.137	0	Monocytes	Map7d1
0	1.08577946	0.361	0.076	0	Monocytes	Rasgrp4
0	2.56850219	0.332	0.048	0	Monocytes	Idh1

0	1.2636295	0.442	0.158	0	Monocytes	Tpi1
0	1.24105023	0.465	0.181	0	Monocytes	Map2k1
0	0.74951772	0.65	0.366	0	Monocytes	Fermt3
0	1.47371349	0.403	0.12	0	Monocytes	Ethe1
0	0.57157982	0.791	0.508	0	Monocytes	Dazap2
0	1.16304079	0.458	0.175	0	Monocytes	Tmed7
0	0.86950043	0.568	0.285	0	Monocytes	Cmpk1
0	0.86465744	0.589	0.306	0	Monocytes	Lamtor4
0	3.61090797	0.309	0.026	0	Monocytes	Plxnd1
0	5.02448394	0.294	0.012	0	Monocytes	Fcgr1
0	1.46205839	0.356	0.074	0	Monocytes	Pilrb2
0	0.60219196	0.716	0.434	0	Monocytes	Bin2
0	0.83577566	0.531	0.25	0	Monocytes	H13
0	0.30316479	0.467	0.186	0	Monocytes	Pgd
0	1.89695933	0.378	0.098	0	Monocytes	Bak1
0	0.72349724	0.703	0.423	0	Monocytes	Ywhab
0	4.69295583	0.295	0.015	0	Monocytes	Nectin1
0	1.050627	0.428	0.15	0	Monocytes	Rnf19b
0	1.61629579	0.368	0.09	0	Monocytes	Snx10
0	0.7614373	0.581	0.303	0	Monocytes	Rab5c
0	2.23346078	0.348	0.07	0	Monocytes	Dusp22
0	1.14167903	0.474	0.196	0	Monocytes	Mrpl14
0	1.23793144	0.443	0.166	0	Monocytes	Api5
0	0.97890348	0.522	0.245	0	Monocytes	Sema4d
0	1.00204979	0.382	0.106	0	Monocytes	Camk1
0	1.69786312	0.381	0.105	0	Monocytes	Cyfip1
0	0.87185194	0.548	0.272	0	Monocytes	Rel
0	3.23244219	0.31	0.034	0	Monocytes	Tubb6
0	0.42305909	0.596	0.32	0	Monocytes	AB124611
0	0.74565086	0.826	0.55	0	Monocytes	Actr2
0	2.252453	0.342	0.067	0	Monocytes	L1cam
0	0.72223271	0.363	0.088	0	Monocytes	Cd300lb
0	1.03309795	0.446	0.172	0	Monocytes	Rsu1
0	0.68468529	0.509	0.235	0	Monocytes	Notch2
0	3.71535294	0.303	0.029	0	Monocytes	Cln8
0	1.27623003	0.359	0.085	0	Monocytes	Rab31
0	0.82829155	0.856	0.583	0	Monocytes	Emp3
0	0.87069641	0.848	0.575	0	Monocytes	Fam49b
0	0.51171525	0.569	0.296	0	Monocytes	Snx20
0	0.66396438	0.609	0.336	0	Monocytes	Rtn4
0	0.73888129	0.594	0.321	0	Monocytes	Cyb5a
0	0.65334526	0.687	0.415	0	Monocytes	Mtdh
0	0.30146569	0.392	0.12	0	Monocytes	Rnf149
0	1.4284873	0.352	0.08	0	Monocytes	Apobr
0	0.75735071	0.527	0.256	0	Monocytes	Arhgap17
0	0.64998566	0.714	0.443	0	Monocytes	Zfp706
0	0.74296711	0.82	0.549	0	Monocytes	Rac1
0	0.90806394	0.85	0.58	0	Monocytes	Capzb
0	0.65146236	0.615	0.345	0	Monocytes	Arl6ip5
0	0.78146136	0.792	0.522	0	Monocytes	Klf13

0	1.70809412	0.362	0.092	0	Monocytes	Gch1
0	0.68474334	0.746	0.476	0	Monocytes	Ccdc12
0	0.83593059	0.486	0.216	0	Monocytes	Rab1a
0	1.32716757	0.401	0.131	0	Monocytes	Atp6v1a
0	0.72207495	0.576	0.306	0	Monocytes	Mpc2
0	4.23037325	0.284	0.014	0	Monocytes	Ceacam2
0	0.63837118	0.59	0.32	0	Monocytes	Ddx17
0	0.79179029	0.35	0.081	0	Monocytes	Trem3
0	0.53631194	0.789	0.521	0	Monocytes	Vamp8
0	0.72617885	0.52	0.252	0	Monocytes	Atp6v1e1
0	1.0666755	0.448	0.18	0	Monocytes	Ybx3
0	0.84429042	0.437	0.17	0	Monocytes	Chp1
0	1.11712983	0.398	0.131	0	Monocytes	Gm37558
0	1.10810842	0.414	0.147	0	Monocytes	Plekhf2
0	1.09964761	0.438	0.171	0	Monocytes	Edem1
0	0.77192688	0.535	0.268	0	Monocytes	Cltc
0	0.69309458	0.703	0.436	0	Monocytes	Pdia3
0	3.32614637	0.291	0.024	0	Monocytes	Fabp4
0	1.36220118	0.408	0.142	0	Monocytes	Ube2f
0	0.69743489	0.443	0.177	0	Monocytes	March1
0	1.00925659	0.49	0.224	0	Monocytes	Ssr1
0	0.62641978	0.692	0.426	0	Monocytes	Ssr4
0	0.92531174	0.467	0.202	0	Monocytes	Nabp1
0	0.6884695	0.536	0.271	0	Monocytes	Abi1
0	1.77224421	0.351	0.086	0	Monocytes	Dram2
0	2.13240504	0.305	0.04	0	Monocytes	Svip
0	0.81952143	0.434	0.169	0	Monocytes	Ugp2
0	3.33083447	0.297	0.032	0	Monocytes	Pot1b
0	0.45637851	0.605	0.34	0	Monocytes	Cyth4
0	1.06747123	0.548	0.284	0	Monocytes	Stap1
0	2.5200645	0.306	0.042	0	Monocytes	Adam15
0	1.11004195	0.421	0.157	0	Monocytes	Cebpg
0	1.41713368	0.392	0.128	0	Monocytes	Polb
0	2.11540878	0.323	0.06	0	Monocytes	Tifa
0	0.74490391	0.485	0.222	0	Monocytes	Atp6v0d1
0	1.59061511	0.457	0.194	0	Monocytes	Bcl2a1b
0	0.96807241	0.451	0.188	0	Monocytes	Arl5c
0	0.82211277	0.527	0.265	0	Monocytes	Ppp1cb
0	3.65506721	0.296	0.034	0	Monocytes	Ldlr
0	0.94823138	0.381	0.119	0	Monocytes	Pqlc1
0	0.75629272	0.583	0.321	0	Monocytes	Esd
0	0.50166669	0.671	0.41	0	Monocytes	Hcls1
0	0.9663056	0.478	0.217	0	Monocytes	Emc7
0	1.73536792	0.37	0.109	0	Monocytes	Pltp
0	0.62100197	0.536	0.275	0	Monocytes	Tnfaip8l2
0	0.93826257	0.415	0.154	0	Monocytes	Plekho1
0	0.98002403	0.398	0.137	0	Monocytes	Tnrc18
0	0.70101861	0.687	0.427	0	Monocytes	B4galnt1
0	1.45714133	0.373	0.113	0	Monocytes	Fam117b
0	0.6294175	0.626	0.366	0	Monocytes	Scp2

0	1.93626147	0.331	0.071	0	Monocytes	Etv6
0	0.67142995	0.624	0.364	0	Monocytes	Psmd8
0	0.29021298	0.891	0.631	0	Monocytes	Taldo1
0	0.89707822	0.356	0.096	0	Monocytes	Lpcat2
0	2.90919539	0.293	0.033	0	Monocytes	Gm37472
0	0.79990181	0.341	0.081	0	Monocytes	Nfe2
0	0.82778189	0.514	0.255	0	Monocytes	Itch
0	1.71547483	0.354	0.095	0	Monocytes	Slc35c2
0	2.22449844	0.32	0.061	0	Monocytes	Dnajb14
0	1.38534257	0.379	0.12	0	Monocytes	Ipo7
0	1.5373155	0.34	0.081	0	Monocytes	Tmem50b
0	1.30101217	0.4	0.141	0	Monocytes	Ilrun
0	2.7121229	0.294	0.036	0	Monocytes	Cebpa
0	0.61101755	0.8	0.542	0	Monocytes	Atp6v0c
0	0.51817097	0.793	0.536	0	Monocytes	Pkm
0	0.99158084	0.408	0.151	0	Monocytes	Crk
0	0.76760064	0.565	0.308	0	Monocytes	Uqcrfs1
0	1.4305447	0.369	0.113	0	Monocytes	Tmem131
0	4.81271133	0.265	0.009	0	Monocytes	Tgm2
0	1.96686119	0.31	0.054	0	Monocytes	Aldh3b1
0	0.96135454	0.815	0.559	0	Monocytes	Slc25a5
0	2.29001506	0.313	0.058	0	Monocytes	Plagl2
0	0.3139779	0.348	0.093	0	Monocytes	Trem1
0	2.70545163	0.304	0.049	0	Monocytes	Gsto1
0	1.01772918	0.466	0.212	0	Monocytes	Tmco1
0	1.27868136	0.392	0.138	0	Monocytes	Tor1a
0	3.98481125	0.267	0.013	0	Monocytes	Thbd
0	1.79709255	0.337	0.083	0	Monocytes	Abcd1
0	0.66617891	0.792	0.538	0	Monocytes	Cox5a
0	0.71895776	0.525	0.271	0	Monocytes	Limd1
0	0.7259015	0.493	0.239	0	Monocytes	Derl1
0	0.45535641	0.535	0.282	0	Monocytes	Arhgap9
0	0.30533171	0.412	0.159	0	Monocytes	D1Ertd622e
0	2.43582898	0.303	0.05	0	Monocytes	Klhl5
0	1.17121504	0.415	0.162	0	Monocytes	Fndc3a
0	2.45753736	0.302	0.05	0	Monocytes	Ggta1
0	1.07581838	0.388	0.136	0	Monocytes	Sh2b3
0	0.7546278	0.821	0.569	0	Monocytes	Calr
0	0.80726005	0.389	0.137	0	Monocytes	Arrdc3
0	1.3790838	0.342	0.09	0	Monocytes	Bcl6
0	2.47021891	0.3	0.048	0	Monocytes	Tiam1
0	0.6145721	0.605	0.353	0	Monocytes	Dock8
0	2.75122859	0.988	0.737	0	Monocytes	Cst3
0	2.45267096	0.282	0.031	0	Monocytes	Sort1
0	0.96294308	0.469	0.218	0	Monocytes	M6pr
0	1.12159983	0.383	0.132	0	Monocytes	Gsdmd
0	1.26773773	0.419	0.168	0	Monocytes	Sdf2l1
0	4.00917241	0.272	0.022	0	Monocytes	Stom
0	1.90943816	0.294	0.044	0	Monocytes	I830077J02Rik
0	0.81117674	0.877	0.627	0	Monocytes	Ptp4a2

0	2.42612521	0.317	0.067	0	Monocytes	Cyth3
0	1.41468435	0.337	0.087	0	Monocytes	Abca7
0	1.00589176	0.4	0.15	0	Monocytes	Ppp1r9b
0	1.17936227	0.349	0.099	0	Monocytes	Zbtb7b
0	0.88325698	0.419	0.169	0	Monocytes	Rab8b
0	1.64980896	0.336	0.087	0	Monocytes	Gsap
0	1.05425416	0.398	0.149	0	Monocytes	Sh3bp1
0	0.61051229	0.509	0.26	0	Monocytes	Iscu
0	0.64724915	0.575	0.326	0	Monocytes	Lman2
0	0.71253116	0.53	0.282	0	Monocytes	Sdhb
0	3.28649155	0.27	0.022	0	Monocytes	Sulf2
0	2.92705964	0.268	0.02	0	Monocytes	AI839979
0	1.56584986	0.342	0.094	0	Monocytes	Gm15987
0	0.81001168	0.454	0.206	0	Monocytes	Far1
0	0.74365809	0.863	0.615	0	Monocytes	Gnb2
0	2.29391301	0.288	0.04	0	Monocytes	Nhsl2
0	2.68938298	0.286	0.039	0	Monocytes	Stx3
0	0.39608944	0.486	0.24	0	Monocytes	Orai1
0	1.21660882	0.901	0.655	0	Monocytes	Ucp2
0	0.80757918	0.488	0.242	0	Monocytes	Serinc1
0	6.64156821	0.25	0.004	0	Monocytes	Vcan
0	2.99709468	0.274	0.029	0	Monocytes	Slc16a10
0	1.35626099	0.378	0.133	0	Monocytes	Tbpl1
0	0.46069966	0.35	0.105	0	Monocytes	Slc16a3
0	1.75656311	0.318	0.073	0	Monocytes	Cpt1a
0	0.63057201	0.544	0.3	0	Monocytes	Lamtor1
0	0.95847142	0.432	0.188	0	Monocytes	Surf4
0	0.81436582	0.883	0.639	0	Monocytes	Cytip
0	0.83664868	0.445	0.201	0	Monocytes	Hook3
0	0.86500753	0.431	0.187	0	Monocytes	Unc119
0	1.69845613	0.31	0.066	0	Monocytes	Klf10
0	2.56858107	0.289	0.045	0	Monocytes	Arhgap39
0	2.81242665	0.284	0.04	0	Monocytes	Vwa5a
0	1.3477187	0.338	0.095	0	Monocytes	Arhgap26.1
0	0.56860448	0.796	0.553	0	Monocytes	Apbb1ip
0	1.28880757	0.345	0.102	0	Monocytes	Ptpn12
0	1.01723164	0.411	0.168	0	Monocytes	Dlst
0	1.30281314	0.362	0.12	0	Monocytes	Scarb2
0	0.59574516	0.783	0.541	0	Monocytes	Brk1
0	2.68880537	0.277	0.036	0	Monocytes	Slc8b1
0	0.65089174	0.482	0.241	0	Monocytes	Mbnl2
0	3.47076199	0.258	0.018	0	Monocytes	E230029C05R
0	1.05616756	0.397	0.157	0	Monocytes	Rnf166
0	1.3350657	0.36	0.12	0	Monocytes	Capn2
0	0.76244374	0.417	0.177	0	Monocytes	Ctdsp2
0	0.91290163	0.397	0.158	0	Monocytes	Cop1
0	1.04553541	0.385	0.146	0	Monocytes	Camk1d
0	0.46814655	0.368	0.129	0	Monocytes	Trib1
0	1.49710201	0.342	0.104	0	Monocytes	Vps13c
0	3.45350393	0.261	0.023	0	Monocytes	Gatm

0	0.94026395	0.439	0.201	0	Monocytes	Hdlbp
0	0.66785762	0.498	0.26	0	Monocytes	Lamtor5
0	2.49541362	0.283	0.045	0	Monocytes	Hpse
0	1.09905257	0.416	0.178	0	Monocytes	Man2a1
0	0.53283756	0.807	0.57	0	Monocytes	Fos
0	1.63918617	0.326	0.089	0	Monocytes	Mfsd1
0	1.19993507	0.378	0.141	0	Monocytes	Hopx
0	1.85281354	0.282	0.045	0	Monocytes	Tlr2
0	1.16547121	0.915	0.678	0	Monocytes	Rap1b
0	0.45152926	0.817	0.58	0	Monocytes	Ostf1
0	1.38099409	0.326	0.09	0	Monocytes	Alcam
0	1.33965973	0.375	0.139	0	Monocytes	Atp2a2
0	0.95857924	0.463	0.228	0	Monocytes	Dusp5
0	0.75053362	0.817	0.582	0	Monocytes	Hspa5
0	0.78472322	0.51	0.275	0	Monocytes	Ptk2b
0	0.34233901	0.469	0.234	0	Monocytes	Ptms
0	1.36690827	0.337	0.103	0	Monocytes	Mafg
0	1.52399639	0.333	0.099	0	Monocytes	Tspan14
0	0.81395819	0.874	0.64	0	Monocytes	Ywhaz
0	1.56495838	0.317	0.084	0	Monocytes	Ids
0	0.73332968	0.495	0.262	0	Monocytes	Mkln1
0	1.19932591	0.337	0.105	0	Monocytes	Foxn2
0	0.7487666	0.488	0.256	0	Monocytes	Nedd9
0	0.98716201	0.41	0.178	0	Monocytes	Fam120a
0	0.52354627	0.807	0.576	0	Monocytes	Hint1
0	1.56608517	0.276	0.045	0	Monocytes	Cdc42ep2
0	1.16890142	0.382	0.151	0	Monocytes	Pon2
0	0.50284368	0.482	0.251	0	Monocytes	Fuca1
0	0.6858791	0.444	0.214	0	Monocytes	Arpc1a
0	1.21366823	0.317	0.087	0	Monocytes	Arid3a
0	0.81002196	0.377	0.147	0	Monocytes	Nfic
0	0.84560313	0.379	0.149	0	Monocytes	Myo9b
0	1.74244197	0.289	0.059	0	Monocytes	Itgax
0	0.78515781	0.466	0.237	0	Monocytes	AI662270
0	0.55421655	0.461	0.232	0	Monocytes	Slc12a6
0	0.94636559	0.399	0.17	0	Monocytes	Necap2
0	3.6630681	0.252	0.023	0	Monocytes	Cdk14
0	0.75914285	0.326	0.097	0	Monocytes	Tcn2
0	0.71446342	0.475	0.246	0	Monocytes	Rpn2
0	0.62364667	0.43	0.201	0	Monocytes	Gm45153
0	1.8164681	0.316	0.088	0	Monocytes	Tmem109
0	1.568661	0.307	0.079	0	Monocytes	Nuak2
0	1.32973106	0.346	0.118	0	Monocytes	Leprot
0	2.68604052	0.259	0.032	0	Monocytes	Rnd3
0	0.9753719	0.383	0.156	0	Monocytes	Gpr132
0	1.14788053	0.371	0.145	0	Monocytes	Cept1
0	0.89856849	0.315	0.089	0	Monocytes	Stx11
0	0.83224594	0.458	0.232	0	Monocytes	Cstb
0	2.26414947	0.268	0.042	0	Monocytes	Hmox1
0	2.9212784	0.264	0.038	0	Monocytes	Hes1

0	0.71978564	0.302	0.076	0	Monocytes	Lilra6
0	1.04157708	0.372	0.147	0	Monocytes	Znhit1
0	0.96497696	0.378	0.153	0	Monocytes	Casp1
0	0.74122325	0.382	0.157	0	Monocytes	Hpcal1
0	0.86854164	0.408	0.183	0	Monocytes	2900097C17R
0	1.59560826	0.295	0.07	0	Monocytes	Tmem176b
0	0.84408288	0.409	0.184	0	Monocytes	Vps35
0	1.905426	0.279	0.055	0	Monocytes	Arrb1
0	2.02814464	0.288	0.064	0	Monocytes	Itgav
0	1.1485174	0.343	0.12	0	Monocytes	Pdlim5
0	1.72575947	0.315	0.092	0	Monocytes	Gfpt1
0	1.77063253	0.288	0.065	0	Monocytes	Pcnx
0	0.77586883	0.404	0.182	0	Monocytes	Rps6ka1
0	0.61376544	0.461	0.239	0	Monocytes	Rala
0	1.40950344	0.316	0.094	0	Monocytes	Cep170
0	2.60514672	0.253	0.031	0	Monocytes	Olfm1
0	2.1156585	0.256	0.034	0	Monocytes	Ebi3
0	2.38279008	0.269	0.048	0	Monocytes	Mospd2
0	2.14776308	0.271	0.05	0	Monocytes	Daglb
0	0.61517608	0.316	0.095	0	Monocytes	Chil1
0	1.67370242	0.304	0.083	0	Monocytes	Dok1
0	0.96746316	0.361	0.14	0	Monocytes	Tm2d2
0	0.99386546	0.369	0.149	0	Monocytes	Aph1a
0	0.57906574	0.426	0.206	0	Monocytes	Stat6
0	0.67502888	0.424	0.205	0	Monocytes	Cers2
0	0.73872042	0.426	0.207	0	Monocytes	Runx1
0	0.64014146	0.84	0.621	0	Monocytes	Arpc5
0	2.41429175	0.288	0.069	0	Monocytes	Ddit4
0	1.11842996	0.35	0.132	0	Monocytes	St3gal1
0	1.44597072	0.294	0.076	0	Monocytes	Map3k11
0	1.09735641	0.342	0.125	0	Monocytes	Elk3
0	1.9070295	0.281	0.064	0	Monocytes	Nkiras2
0	0.95077586	0.393	0.176	0	Monocytes	Nmt1
0	1.35790275	0.319	0.102	0	Monocytes	Abca3
0	0.72118283	0.427	0.21	0	Monocytes	Dync1h1
0	0.86024697	0.39	0.174	0	Monocytes	Dipk1a
0	1.74984825	0.272	0.057	0	Monocytes	Sh3bp2
0	0.3158448	0.364	0.149	0	Monocytes	Pxn
0	0.57216923	0.422	0.207	0	Monocytes	Chmp3
0	1.02433683	0.359	0.145	0	Monocytes	Usp8
0	1.27928132	0.314	0.1	0	Monocytes	Brpf1
0	0.97227514	0.367	0.153	0	Monocytes	Trappc1
0	1.21132857	0.332	0.119	0	Monocytes	Rb1
0	0.85364198	0.404	0.191	0	Monocytes	Me2
0	1.12493337	0.347	0.134	0	Monocytes	Snx1
0	2.32780626	0.256	0.044	0	Monocytes	Frrs1
0	0.84341559	0.394	0.182	0	Monocytes	Hexb
0	0.72566417	0.428	0.216	0	Monocytes	Mcmbp
0	0.70444382	0.42	0.208	0	Monocytes	Scamp2
0	0.50664453	0.408	0.196	0	Monocytes	Xpr1

0	1.29425978	0.27	0.059	0	Monocytes	Dgkh
0	0.46795442	0.358	0.147	0	Monocytes	Csnk1e
0	0.70581245	0.388	0.178	0	Monocytes	Eps15
0	2.55967029	0.254	0.044	0	Monocytes	Dse
0	1.01770972	0.354	0.144	0	Monocytes	Vamp4
0	1.46550115	0.301	0.091	0	Monocytes	Calhm2
0	1.03735797	0.336	0.127	0	Monocytes	Plekhm3
0	0.83060544	0.335	0.126	0	Monocytes	Xylt1
0	1.2471184	0.327	0.118	0	Monocytes	Ppm1h
0	1.61183651	0.267	0.058	0	Monocytes	Dock5
0	1.7677177	0.272	0.063	0	Monocytes	Ppt2
0	0.6681679	0.419	0.21	0	Monocytes	Rab1b
0	2.16162721	0.261	0.053	0	Monocytes	Asph
0	1.83725745	0.254	0.046	0	Monocytes	Plekhg3
0	1.39766627	0.311	0.103	0	Monocytes	Isoc1
0	0.79649779	0.36	0.153	0	Monocytes	Tmed3
0	0.82207505	0.409	0.202	0	Monocytes	Stx16
0	0.37953251	0.384	0.178	0	Monocytes	Mettl9
0	0.90449075	0.388	0.182	0	Monocytes	Mgat2
0	0.57666808	0.823	0.617	0	Monocytes	Arf5
0	0.85046512	0.335	0.13	0	Monocytes	Ythdf3
0	1.23817171	0.335	0.13	0	Monocytes	Parl
0	0.61383604	0.382	0.177	0	Monocytes	Acap2
0	0.80727449	0.376	0.171	0	Monocytes	Kmt5a
0	0.76275614	0.37	0.165	0	Monocytes	Cic
0	1.40205674	0.277	0.073	0	Monocytes	Qsox1
0	0.98660683	0.332	0.128	0	Monocytes	Ncoa1
0	0.81434464	0.326	0.122	0	Monocytes	Pik3cg
0	1.5909808	0.292	0.088	0	Monocytes	Rhoq
0	0.7406939	0.385	0.182	0	Monocytes	Med8
0	1.18057234	0.305	0.102	0	Monocytes	Pitpnm1
0	1.20743538	0.312	0.11	0	Monocytes	Coro1c
0	0.77886383	0.389	0.187	0	Monocytes	Fkbp2
0	1.29649916	0.319	0.118	0	Monocytes	Tm9sf4
0	0.96305636	0.331	0.13	0	Monocytes	Lats2
0	1.52650477	0.295	0.094	0	Monocytes	Lrrk1
0	1.12765072	0.298	0.097	0	Monocytes	Zswim6
0	1.75551052	0.263	0.063	0	Monocytes	Ggh
0	0.72954831	0.383	0.183	0	Monocytes	Copz1
0	0.71237695	0.364	0.164	0	Monocytes	Sptlc2
0	0.31692667	0.342	0.143	0	Monocytes	Abr
0	0.71732146	0.305	0.106	0	Monocytes	Sec14l1
0	1.75852219	0.261	0.062	0	Monocytes	Tcirg1
0	1.45805155	0.288	0.089	0	Monocytes	Map3k14
0	0.76724885	0.863	0.664	0	Monocytes	H2az1
0	1.72649887	0.282	0.083	0	Monocytes	Fcgrt
0	1.99258921	0.26	0.062	0	Monocytes	Fam102b
0	1.08987228	0.303	0.105	0	Monocytes	Vps26a
0	1.32417225	0.289	0.091	0	Monocytes	Tmem14c
0	0.8834995	0.32	0.123	0	Monocytes	Tbc1d10b

0	1.16888653	0.327	0.13	0	Monocytes	Chd9
0	2.02229749	0.272	0.075	0	Monocytes	Smagp
0	0.73227269	0.922	0.726	0	Monocytes	Cd47
0	1.71354822	0.263	0.067	0	Monocytes	Atp13a2
0	1.46164296	0.272	0.076	0	Monocytes	Usp12
0	0.93744733	0.31	0.114	0	Monocytes	Mvp
0	0.5428917	0.279	0.083	0	Monocytes	Pfkfb4
0	0.91587856	0.336	0.14	0	Monocytes	9530068E07R
0	0.71829579	0.37	0.175	0	Monocytes	Usp9x
0	1.68921905	0.252	0.057	0	Monocytes	Apaf1
0	1.18781047	0.303	0.108	0	Monocytes	Ctnna1
0	1.03007968	0.325	0.13	0	Monocytes	Acsl5
0	1.32339189	0.292	0.097	0	Monocytes	Acot9
0	0.67609583	0.366	0.171	0	Monocytes	Osbpl8
0	0.70643295	0.279	0.085	0	Monocytes	Pdlim4
0	0.6755653	0.346	0.152	0	Monocytes	Vamp3
0	0.84947525	0.888	0.695	0	Monocytes	Sem1
0	0.34245617	0.341	0.148	0	Monocytes	Snx18
0	2.1001208	0.25	0.057	0	Monocytes	Abcc5
0	0.76922284	0.363	0.17	0	Monocytes	Trim8
0	1.00363384	0.338	0.146	0	Monocytes	Bscl2
0	1.29114145	0.261	0.07	0	Monocytes	Vcl
0	0.75974808	0.368	0.178	0	Monocytes	Snrpb2
0	0.93560494	0.328	0.138	0	Monocytes	Elovl1
0	1.15962678	0.253	0.063	0	Monocytes	Itpripl2
0	0.91192866	0.294	0.104	0	Monocytes	Sgpl1
0	0.97929482	0.297	0.107	0	Monocytes	Lrrfip2
0	0.86327961	0.261	0.071	0	Monocytes	Slc15a3
0	0.86112074	0.359	0.169	0	Monocytes	Rpn1
0	0.63747905	0.345	0.155	0	Monocytes	Ap3s1
0	0.81644231	0.323	0.134	0	Monocytes	Rcbtb2
0	0.84456331	0.344	0.155	0	Monocytes	Napg
0	1.25806348	0.278	0.09	0	Monocytes	Slc25a28
0	0.82482813	0.317	0.129	0	Monocytes	Phyh
0	1.15477026	0.277	0.09	0	Monocytes	Pacsin2
0	1.44351156	0.261	0.074	0	Monocytes	Wls
0	0.68927971	0.313	0.126	0	Monocytes	Map3k3
0	0.6989828	0.307	0.12	0	Monocytes	Kctd20
0	1.27695175	0.287	0.1	0	Monocytes	Cndp2
0	0.96176971	0.33	0.144	0	Monocytes	Spcs3
0	0.8489573	0.328	0.142	0	Monocytes	Myo1c
0	0.58153391	0.275	0.089	0	Monocytes	Cpd
0	1.09398204	0.298	0.112	0	Monocytes	Lpar6
0	0.73235655	0.306	0.12	0	Monocytes	Tfeb
0	0.88437935	0.285	0.099	0	Monocytes	Mtmr6
0	0.58494755	0.783	0.598	0	Monocytes	Sec61b
0	0.73556443	0.336	0.151	0	Monocytes	Nipa2
0	1.06648791	0.313	0.13	0	Monocytes	Cat
0	0.74135525	0.3	0.117	0	Monocytes	Stk40
0	0.78715284	0.331	0.149	0	Monocytes	Tmem219

0	1.0553222	0.302	0.12	0	Monocytes	Dr1
0	1.33788143	0.263	0.082	0	Monocytes	Mfsd5
0	0.56435322	0.799	0.619	0	Monocytes	Clta
0	1.27717318	0.276	0.096	0	Monocytes	Blvra
0	0.91122611	0.321	0.142	0	Monocytes	Pigt
0	1.20472761	0.25	0.071	0	Monocytes	Tfe3
0	1.03599517	0.25	0.071	0	Monocytes	Tmem164
0	1.58051064	0.251	0.072	0	Monocytes	Dpy19l1
0	1.15546215	0.266	0.088	0	Monocytes	Bckdha
0	0.96760422	0.261	0.083	0	Monocytes	Cyb5r1
0	1.52907648	0.261	0.083	0	Monocytes	Mink1
0	0.73793004	0.281	0.104	0	Monocytes	Gpr137b-ps
0	0.93962951	0.28	0.104	0	Monocytes	Sec24b
0	0.85410098	0.271	0.095	0	Monocytes	Atp8b4
0	0.71371424	0.314	0.139	0	Monocytes	Dcaf12
0	0.74524593	0.256	0.081	0	Monocytes	Gm45222
0	0.64131566	0.3	0.125	0	Monocytes	Trpv2
0	0.80793866	0.299	0.125	0	Monocytes	Map1lc3a
0	0.99919778	0.276	0.103	0	Monocytes	Prkx
0	0.82764871	0.272	0.099	0	Monocytes	Fuca2
0	0.94034446	0.317	0.144	0	Monocytes	Pnp
0	0.91174147	0.309	0.136	0	Monocytes	Mpp6
0	1.05533272	0.791	0.619	0	Monocytes	Vim
0	0.98041163	0.962	0.79	0	Monocytes	Laptm5
0	1.27763522	0.259	0.088	0	Monocytes	Ncstn
0	0.91753438	0.304	0.133	0	Monocytes	Uggt1
0	0.87649536	0.305	0.134	0	Monocytes	Zbp1
0	0.65679388	0.305	0.134	0	Monocytes	Nfkb2
0	0.9312755	0.274	0.104	0	Monocytes	Pmaip1
0	0.63533392	0.298	0.128	0	Monocytes	Fem1c
0	1.35175764	0.252	0.083	0	Monocytes	Wdr3
0	0.89953603	0.3	0.131	0	Monocytes	Anxa7
0	0.52015068	0.277	0.109	0	Monocytes	Mid1ip1
0	1.26686769	0.261	0.093	0	Monocytes	P4ha1
0	0.76071752	0.297	0.129	0	Monocytes	Fkbp15
0	1.24587599	0.253	0.086	0	Monocytes	Agps
0	0.64011351	0.288	0.122	0	Monocytes	Glul
0	0.95995969	0.256	0.091	0	Monocytes	Parp8
0	1.02196494	0.275	0.111	0	Monocytes	Sel1l
0	0.66860308	0.264	0.103	0	Monocytes	Comt
0	0.88389271	0.271	0.11	0	Monocytes	Map3k8
0	0.53962659	0.865	0.705	0	Monocytes	Hspa8
0	0.61564129	0.251	0.091	0	Monocytes	Map3k5
0	0.87228582	0.263	0.104	0	Monocytes	Gga1
0	0.79104743	0.272	0.114	0	Monocytes	Rap2b
0	1.14464181	0.255	0.097	0	Monocytes	Gns
0	1.11898734	0.269	0.112	0	Monocytes	Elovl5
0	0.65335749	0.265	0.109	0	Monocytes	Snx27
0	0.72748676	0.251	0.095	0	Monocytes	Oaz2
0	0.98348493	0.264	0.109	0	Monocytes	Tmem259

0	0.91607572	0.264	0.109	0	Monocytes	Mcur1
0	0.63867446	0.261	0.106	0	Monocytes	Runx2
0	0.96721325	0.252	0.097	0	Monocytes	Pisd
0	1.23367073	0.254	0.099	0	Monocytes	Snd1
0	0.49139865	0.881	0.727	0	Monocytes	Arpc3
0	0.91834996	0.259	0.105	0	Monocytes	Psen1
0	0.50575496	0.888	0.735	0	Monocytes	Gng5
0	0.88792827	0.259	0.107	0	Monocytes	Psme4
0	0.93649713	0.255	0.104	0	Monocytes	Atf6b
0	0.97152516	0.955	0.804	0	Monocytes	Cyba
0	0.74755414	0.924	0.778	0	Monocytes	Msn
0	0.70875196	0.92	0.776	0	Monocytes	Rhoa
0	0.43888255	0.919	0.801	0	Monocytes	Lsp1
0	0.81745057	0.929	0.823	0	Monocytes	Arpc1b
0	0.45692221	0.919	0.818	0	Monocytes	Cdc42
0	0.97352278	0.94	0.842	0	Monocytes	Calm1
0	0.50083341	0.905	0.809	0	Monocytes	Cox4i1
0	0.53747616	0.947	0.852	0	Monocytes	Itm2b
0	0.46045277	0.926	0.832	0	Monocytes	Gnai2
0	0.74659689	0.928	0.835	0	Monocytes	Arpc2
0	0.41645734	0.945	0.863	0	Monocytes	Rpl10
0	0.80719311	0.95	0.89	0	Monocytes	Actg1
0	1.20945089	0.963	0.912	0	Monocytes	Ftl1
0	0.32595298	0.978	0.929	0	Monocytes	Ptprc
0	0.61133037	0.952	0.919	0	Monocytes	Coro1a
0	0.75111284	0.983	0.958	0	Monocytes	B2m
0	0.327053	0.965	0.946	0	Monocytes	H3f3a
0	0.30068246	0.995	0.993	0	Monocytes	Actb
0	0.35756825	0.998	0.998	0	Monocytes	Tmsb4x
5.575E-307	0.70795843	0.374	0.181	1.382E-302	Monocytes	Golga7
1.438E-306	0.68070083	0.33	0.151	3.566E-302	Monocytes	Arl8a
8.484E-305	0.53421523	0.77	0.521	2.104E-300	Monocytes	Eif4a1
1.506E-303	0.79635179	0.292	0.129	3.735E-299	Monocytes	Rnf213
2.611E-303	0.75249921	0.334	0.157	6.473E-299	Monocytes	Tmem165
1.446E-302	0.66008395	0.348	0.164	3.586E-298	Monocytes	Elf4
1.531E-301	0.99817286	0.27	0.118	3.796E-297	Monocytes	Cyb5r3
5.871E-301	0.34924861	0.29	0.124	1.456E-296	Monocytes	Mfsd6
1.822E-300	0.91330921	0.273	0.118	4.519E-296	Monocytes	Lrpap1
5.845E-300	0.59629638	0.603	0.353	1.449E-295	Monocytes	Ndufb8
7.455E-299	0.62933258	0.523	0.292	1.848E-294	Monocytes	Pdcd6ip
1.075E-296	0.99749154	0.279	0.124	2.665E-292	Monocytes	Hsd17b12
1.357E-296	0.8084016	0.301	0.136	3.364E-292	Monocytes	Ptpn11
4.167E-296	0.47528334	0.592	0.344	1.033E-291	Monocytes	Pbxip1
4.314E-296	0.29800448	0.46	0.234	1.07E-291	Monocytes	Glipr1
5.335E-296	0.50714761	0.714	0.465	1.323E-291	Monocytes	Reep5
5.506E-295	0.72741036	0.359	0.175	1.365E-290	Monocytes	Ppp3r1
3.011E-294	0.78975195	0.279	0.122	7.466E-290	Monocytes	Epb41l2
5.084E-293	0.61058763	0.45	0.238	1.261E-288	Monocytes	Larp4b
6.199E-293	0.45066345	0.576	0.327	1.537E-288	Monocytes	Mkrn1
8.169E-293	0.48388184	0.49	0.263	2.025E-288	Monocytes	Rock2

1.704E-291	0.67004253	0.435	0.227	4.226E-287	Monocytes	Uqcc2
2.918E-291	0.60213586	0.35	0.167	7.235E-287	Monocytes	Ilk
3.375E-291	0.55743345	0.376	0.183	8.369E-287	Monocytes	N4bp2l1
2.753E-290	0.75301379	0.325	0.152	6.825E-286	Monocytes	Mapre2
1.17E-289	0.95461789	0.254	0.109	2.9E-285	Monocytes	Lrrc59
1.965E-288	0.46702524	0.411	0.206	4.871E-284	Monocytes	Rnf13
2.762E-287	0.73803971	0.294	0.133	6.847E-283	Monocytes	Ripk1
1.005E-286	0.70855767	0.284	0.125	2.492E-282	Monocytes	Ist1
4.471E-286	0.52988098	0.564	0.327	1.109E-281	Monocytes	Chmp4b
5.167E-285	0.55912529	0.368	0.18	1.281E-280	Monocytes	Adipor2
8.288E-285	0.66900307	0.33	0.156	2.055E-280	Monocytes	Cuedc2
9.476E-285	0.54673558	0.63	0.379	2.35E-280	Monocytes	Cd48
1.22E-283	0.60516919	0.474	0.255	3.025E-279	Monocytes	Vdac3
2.783E-283	0.87977151	0.27	0.12	6.901E-279	Monocytes	Tmc6
7.366E-283	0.61442356	0.389	0.196	1.826E-278	Monocytes	Ube2a
8.966E-283	0.49679715	0.691	0.441	2.223E-278	Monocytes	Rab14
1.545E-282	0.51796982	0.765	0.527	3.831E-278	Monocytes	Arhgdia
9.661E-282	0.70305918	0.298	0.136	2.395E-277	Monocytes	Usp4
8.828E-281	0.71394438	0.394	0.201	2.189E-276	Monocytes	Etfb
8.383E-280	0.38576921	0.31	0.141	2.079E-275	Monocytes	Ttyh3
1.271E-279	0.4516836	0.545	0.303	3.153E-275	Monocytes	Rasa3
5.821E-279	0.65800583	0.398	0.205	1.443E-274	Monocytes	Kxd1
8.373E-279	0.60596347	0.58	0.345	2.076E-274	Monocytes	Tmem160
1.3E-278	0.47950585	0.393	0.196	3.224E-274	Monocytes	Lasp1
4.297E-278	0.33927515	0.443	0.231	1.065E-273	Monocytes	Reep3
5.586E-278	0.50580186	0.581	0.34	1.385E-273	Monocytes	Wdr1
8.947E-278	0.37837525	0.854	0.703	2.218E-273	Monocytes	Gapdh
9.76E-278	0.35245033	0.283	0.125	2.42E-273	Monocytes	Dhx38
4.316E-277	0.74428503	0.284	0.128	1.07E-272	Monocytes	Pdlim1
7.836E-277	0.65950017	0.29	0.131	1.943E-272	Monocytes	Vcpip1
1.916E-276	0.6982951	0.378	0.191	4.751E-272	Monocytes	Crlf2
1.467E-274	0.61151889	0.794	0.642	3.637E-270	Monocytes	Crip1
1.541E-274	0.49149058	0.671	0.427	3.822E-270	Monocytes	Sec11c
6.317E-274	0.55946065	0.27	0.118	1.566E-269	Monocytes	Fry
1.103E-273	0.48075787	0.448	0.236	2.735E-269	Monocytes	Tmbim4
1.109E-273	0.3749713	0.401	0.2	2.75E-269	Monocytes	Rin3
1.981E-273	0.53904776	0.368	0.183	4.912E-269	Monocytes	Rilpl2
3.248E-273	0.64001811	0.51	0.287	8.053E-269	Monocytes	Tram1
8.841E-272	0.5045537	0.341	0.164	2.192E-267	Monocytes	Arhgef2
1.442E-271	0.75012885	0.253	0.11	3.574E-267	Monocytes	Gosr2
3.36E-271	0.94906919	0.337	0.168	8.332E-267	Monocytes	Lrrc8c
5.737E-271	0.27450657	0.336	0.155	1.423E-266	Monocytes	Gsn
4.871E-269	0.79576464	0.254	0.111	1.208E-264	Monocytes	Il6st
7.472E-269	0.2968007	0.785	0.557	1.853E-264	Monocytes	Cap1
1.455E-268	0.78112999	0.347	0.173	3.608E-264	Monocytes	Rftn1
2.057E-267	0.91649091	0.555	0.355	5.099E-263	Monocytes	Smc6
4.451E-267	0.57117771	0.647	0.405	1.104E-262	Monocytes	Lrrfip1
4.214E-266	0.6056016	0.323	0.155	1.045E-261	Monocytes	Ppp1r12c
4.516E-266	0.55916835	0.453	0.246	1.12E-261	Monocytes	Ufc1
8.262E-266	0.58518184	0.39	0.2	2.049E-261	Monocytes	Dctn2

5.098E-264	0.46918071	0.696	0.449	1.264E-259	Monocytes	Mrfap1
1.376E-263	0.62740248	0.63	0.398	3.412E-259	Monocytes	Stk24
6.662E-263	0.52693767	0.353	0.172	1.652E-258	Monocytes	Acly
8.589E-263	0.73056925	0.264	0.118	2.13E-258	Monocytes	Arl8b
1.835E-262	0.46139155	0.739	0.507	4.551E-258	Monocytes	Arhgap30
6.611E-262	0.60415727	0.449	0.244	1.639E-257	Monocytes	Tbcb
1.078E-261	0.4068728	0.252	0.111	2.674E-257	Monocytes	Cks2
1.506E-261	0.47246658	0.76	0.538	3.734E-257	Monocytes	Fis1
1.072E-259	0.50830463	0.326	0.156	2.657E-255	Monocytes	Fbrs
1.535E-259	0.82547722	0.253	0.112	3.806E-255	Monocytes	Prkd3
8.7E-259	0.74218201	0.278	0.127	2.157E-254	Monocytes	Usp47
1.729E-257	0.77186419	0.271	0.124	4.288E-253	Monocytes	Lactb
2.12E-256	0.56861575	0.564	0.334	5.256E-252	Monocytes	Bax
3.088E-256	0.57630087	0.394	0.206	7.655E-252	Monocytes	Spg21
4.273E-256	0.39142159	0.27	0.121	1.059E-251	Monocytes	Hmgcl
5.089E-256	0.49345302	0.407	0.213	1.262E-251	Monocytes	Eid1
8.742E-254	0.73558847	0.293	0.139	2.168E-249	Monocytes	Ehmt2
9.707E-254	0.4754414	0.416	0.219	2.407E-249	Monocytes	Taok1
2.934E-253	0.56794013	0.552	0.327	7.276E-249	Monocytes	Prpf40a
1.955E-251	0.47102237	0.763	0.541	4.847E-247	Monocytes	Arf1
9.479E-251	0.60385887	0.362	0.185	2.35E-246	Monocytes	Aftph
1.287E-249	0.44499198	0.61	0.369	3.19E-245	Monocytes	Lnpep
4.041E-249	0.99164984	0.258	0.121	1.002E-244	Monocytes	Slfn5
6.729E-249	0.67801804	0.282	0.133	1.669E-244	Monocytes	Coa5
1.087E-248	0.39278163	0.862	0.702	2.696E-244	Monocytes	Actr3
1.43E-246	0.63639968	0.354	0.181	3.547E-242	Monocytes	Acaa2
1.867E-246	0.73520262	0.304	0.148	4.63E-242	Monocytes	Etfa
2.298E-246	0.6837092	0.345	0.175	5.698E-242	Monocytes	Mydgf
3.812E-246	0.50466806	0.281	0.129	9.452E-242	Monocytes	Wdfy4
7.828E-246	0.51200343	0.368	0.188	1.941E-241	Monocytes	Flii
2.558E-245	0.30236787	0.261	0.116	6.344E-241	Monocytes	Borcs6
2.72E-244	0.81944327	0.26	0.12	6.743E-240	Monocytes	Ccdc115
8.939E-244	0.65674654	0.258	0.117	2.216E-239	Monocytes	Arl6ip6
1.052E-243	0.70949553	0.255	0.116	2.609E-239	Monocytes	Sgpp1
2.997E-243	0.56902067	0.671	0.448	7.432E-239	Monocytes	Ccnl1
5.435E-243	0.6479574	0.403	0.217	1.347E-238	Monocytes	Sod2
6.936E-243	0.42469245	0.462	0.256	1.72E-238	Monocytes	Sla
1.028E-242	0.58013271	0.332	0.165	2.549E-238	Monocytes	Usf1
3.293E-242	0.71281584	0.311	0.153	8.166E-238	Monocytes	Tmem147
1.682E-241	0.47156435	0.689	0.452	4.17E-237	Monocytes	Wasf2
6.473E-241	0.57515805	0.393	0.21	1.605E-236	Monocytes	Copa
7.522E-240	0.58150571	0.329	0.164	1.865E-235	Monocytes	Hcfc1r1
3.08E-239	0.53133673	0.309	0.151	7.637E-235	Monocytes	Stxbp2
4.328E-239	0.59868903	0.42	0.228	1.073E-234	Monocytes	Prdx2
1.715E-238	0.89152894	0.255	0.119	4.251E-234	Monocytes	Ube2g2
2.259E-238	0.72661431	0.314	0.158	5.602E-234	Monocytes	Spata13
3.003E-238	0.5361307	0.263	0.121	7.446E-234	Monocytes	Col4a3bp
8.545E-238	0.43147136	0.537	0.314	2.119E-233	Monocytes	Usf2
4.342E-236	0.61049709	0.301	0.147	1.077E-231	Monocytes	Vapb
2.444E-235	0.43461921	0.459	0.259	6.059E-231	Monocytes	Lars2

9.104E-235	0.35530001	0.382	0.198	2.257E-230	Monocytes	Cmip
1.48E-234	0.5090757	0.715	0.52	3.67E-230	Monocytes	Atp5c1
9.969E-234	0.5031932	0.54	0.32	2.472E-229	Monocytes	Psma4
1.235E-232	0.39806411	0.863	0.694	3.061E-228	Monocytes	Ybx1
3.157E-232	0.38826974	0.657	0.419	7.827E-228	Monocytes	Chmp2a
1.885E-231	0.47022373	0.659	0.434	4.673E-227	Monocytes	Pcbp1
4.06E-230	0.54109918	0.39	0.208	1.007E-225	Monocytes	Atp6ap2
4.171E-230	0.57196825	0.368	0.194	1.034E-225	Monocytes	Cybc1
1.27E-229	0.55705189	0.377	0.2	3.149E-225	Monocytes	Nras
3.311E-229	0.48430346	0.66	0.425	8.21E-225	Monocytes	Tapbp
3.373E-229	0.38523407	0.539	0.317	8.364E-225	Monocytes	Ep300
6.696E-228	0.34233343	0.518	0.298	1.66E-223	Monocytes	Epsti1
4.247E-227	0.36658527	0.345	0.175	1.053E-222	Monocytes	Stat5b
4.303E-227	0.45869943	0.451	0.252	1.067E-222	Monocytes	Sgms1
7.765E-227	0.61358338	0.3	0.148	1.925E-222	Monocytes	Faim
7.814E-227	0.54041586	0.32	0.161	1.938E-222	Monocytes	AI413582
1.88E-226	0.47722081	0.601	0.377	4.661E-222	Monocytes	Eno1
2.331E-226	0.5742742	0.463	0.267	5.78E-222	Monocytes	Ndufc2
2.415E-226	0.5637388	0.447	0.254	5.989E-222	Monocytes	Mef2d
3.856E-226	0.63270186	0.262	0.123	9.562E-222	Monocytes	Cyb5r4
4.119E-226	0.46827741	0.46	0.257	1.021E-221	Monocytes	Lrmp
2.9E-225	0.37182838	0.393	0.208	7.19E-221	Monocytes	Dap
5.47E-225	0.34872098	0.757	0.527	1.356E-220	Monocytes	Grk2
6.294E-224	0.49674052	0.376	0.199	1.561E-219	Monocytes	Adam10
9.832E-224	0.61287318	0.352	0.185	2.438E-219	Monocytes	Sec61a1
7.476E-223	0.33842484	0.567	0.338	1.854E-218	Monocytes	Rab7
9.64E-223	0.43106498	0.766	0.565	2.39E-218	Monocytes	Eif3k
1.05E-222	0.56703306	0.375	0.2	2.603E-218	Monocytes	Tmx3
1.078E-222	0.56097136	0.355	0.187	2.672E-218	Monocytes	Irak1
1.092E-222	0.59939563	0.346	0.181	2.707E-218	Monocytes	Kdelr2
4.445E-221	0.57377647	0.268	0.127	1.102E-216	Monocytes	Irf7
1.425E-220	0.75456533	0.253	0.12	3.534E-216	Monocytes	Scpep1
4.462E-220	0.51597901	0.754	0.535	1.106E-215	Monocytes	Jpt1
5.092E-220	0.55257717	0.256	0.119	1.263E-215	Monocytes	Ccdc71l
1.874E-219	0.58558004	0.454	0.26	4.646E-215	Monocytes	Adcy7
4.228E-219	0.40444674	0.365	0.192	1.048E-214	Monocytes	Cnppd1
2.351E-217	0.44311909	0.478	0.273	5.83E-213	Monocytes	Rexo2
4.792E-217	0.5494669	0.366	0.193	1.188E-212	Monocytes	Nucb1
9.375E-216	0.34999749	0.437	0.244	2.325E-211	Monocytes	Zfp106
1.766E-215	0.60909344	0.263	0.126	4.379E-211	Monocytes	Gak
6.219E-215	0.69304257	0.281	0.139	1.542E-210	Monocytes	Trappc6a
6.732E-215	0.38682934	0.725	0.492	1.669E-210	Monocytes	Abracl
1.141E-214	0.50287828	0.403	0.222	2.829E-210	Monocytes	Mcrip1
1.343E-214	0.4930394	0.357	0.189	3.33E-210	Monocytes	Mtch1
3.091E-214	0.4645787	0.284	0.139	7.664E-210	Monocytes	Rnf216
4.397E-214	0.70115957	0.324	0.17	1.09E-209	Monocytes	Naa50
1.943E-212	0.68284459	0.289	0.145	4.817E-208	Monocytes	Med15
7.016E-212	0.55255727	0.282	0.138	1.74E-207	Monocytes	Clk3
1.005E-211	0.30943947	0.317	0.16	2.491E-207	Monocytes	Zfand3
1.355E-210	0.43504312	0.314	0.159	3.361E-206	Monocytes	Rap2c

3.91E-210	0.64766445	0.297	0.151	9.695E-206	Monocytes	2610507B11R
4.089E-210	0.52213538	0.348	0.183	1.014E-205	Monocytes	Clec2i
4.802E-210	0.47251121	0.294	0.147	1.191E-205	Monocytes	Eloa
5.476E-210	0.61981509	0.271	0.133	1.358E-205	Monocytes	Lypla1
7.744E-210	0.50407772	0.286	0.142	1.92E-205	Monocytes	Pttg1ip
2.527E-209	0.4571027	0.554	0.344	6.266E-205	Monocytes	Ndufb3
8.624E-209	0.38541182	0.39	0.212	2.138E-204	Monocytes	Tbl1xr1
4.509E-208	0.50459507	0.407	0.227	1.118E-203	Monocytes	Arcn1
7.966E-207	0.72823007	0.29	0.148	1.975E-202	Monocytes	Stt3a
1.746E-206	0.49146009	0.455	0.264	4.329E-202	Monocytes	Cdc40
3.25E-206	0.50232981	0.328	0.171	8.059E-202	Monocytes	Atp13a3
1.148E-205	0.53674467	0.337	0.178	2.845E-201	Monocytes	Cs
2.615E-205	0.41707317	0.44	0.252	6.484E-201	Monocytes	Usp25
2.965E-205	0.57659198	0.373	0.205	7.353E-201	Monocytes	Psmd2
3.599E-205	0.49811621	0.27	0.132	8.923E-201	Monocytes	Actr1a
2.46E-204	0.44752568	0.261	0.125	6.101E-200	Monocytes	Mrtfa
4.59E-204	0.51105089	0.521	0.318	1.138E-199	Monocytes	Rab10
1.629E-203	0.35096593	0.439	0.248	4.039E-199	Monocytes	Rgs19
2.371E-203	0.45925062	0.524	0.317	5.879E-199	Monocytes	Eif4h
4.267E-203	0.48752141	0.418	0.237	1.058E-198	Monocytes	Ncoa3
2.563E-202	0.32223297	0.344	0.18	6.354E-198	Monocytes	Ypel5
3.706E-202	0.50269849	0.592	0.38	9.189E-198	Monocytes	Manf
6.083E-202	0.55918162	0.292	0.149	1.508E-197	Monocytes	Colgalt1
3.559E-201	0.65132901	0.477	0.291	8.824E-197	Monocytes	Camk2d
4.702E-201	0.54568368	0.335	0.179	1.166E-196	Monocytes	Cbfb
1.426E-200	0.50532986	0.336	0.179	3.537E-196	Monocytes	Brd7
3.908E-200	0.46964195	0.376	0.207	9.691E-196	Monocytes	Sdhd
9.33E-200	0.44628549	0.56	0.345	2.313E-195	Monocytes	Map4k4
3.074E-199	0.55287271	0.325	0.172	7.622E-195	Monocytes	C1d
6.394E-199	0.37725702	0.758	0.577	1.585E-194	Monocytes	Aldoa
3.723E-198	0.5104376	0.308	0.161	9.231E-194	Monocytes	Atxn10
2.421E-197	0.39366439	0.598	0.378	6.002E-193	Monocytes	Bnip3l
2.424E-197	0.38446433	0.799	0.606	6.009E-193	Monocytes	Cox6b1
7.418E-197	0.40236205	0.731	0.52	1.839E-192	Monocytes	Uqcrq
1.18E-196	0.5141308	0.457	0.269	2.927E-192	Monocytes	Dnajb11
2.308E-196	0.47854221	0.255	0.124	5.722E-192	Monocytes	Wdr45b
5.139E-196	0.32763813	0.582	0.361	1.274E-191	Monocytes	Bcl10
6.571E-195	0.36196813	0.473	0.275	1.629E-190	Monocytes	Twf2
7.474E-195	0.6372501	0.276	0.141	1.853E-190	Monocytes	Sdhc
1.089E-194	0.39519149	0.359	0.193	2.699E-190	Monocytes	Pip4p1
2.532E-194	0.53069595	0.282	0.143	6.278E-190	Monocytes	Ap3d1
4.476E-194	0.4554008	0.624	0.424	1.11E-189	Monocytes	Ndufb1-ps
1.544E-193	0.54232316	0.296	0.153	3.829E-189	Monocytes	Cnpy2
1.663E-193	0.44001652	0.676	0.48	4.123E-189	Monocytes	Ndufa6
6.273E-193	0.4067802	0.393	0.22	1.555E-188	Monocytes	Kdelr1
7.493E-193	0.5663383	0.294	0.152	1.858E-188	Monocytes	Mfsd14a
1.223E-192	0.49754112	0.363	0.2	3.032E-188	Monocytes	Glg1
3.988E-192	0.71662172	0.264	0.134	9.887E-188	Monocytes	Ormdl2
4.094E-192	0.6965213	0.257	0.129	1.015E-187	Monocytes	Prkcsh
4.162E-192	0.31978643	0.257	0.125	1.032E-187	Monocytes	Tbc1d23

1.85E-191	0.50215543	0.354	0.194	4.588E-187	Monocytes	Etf1
2.373E-191	0.43700307	0.571	0.357	5.884E-187	Monocytes	Tm9sf3
4.967E-191	0.42305439	0.314	0.166	1.231E-186	Monocytes	Spen
1.204E-190	0.27457697	0.328	0.173	2.984E-186	Monocytes	Cpne3
1.584E-190	0.50015879	0.293	0.152	3.929E-186	Monocytes	Rps6ka3
2.52E-190	0.42814605	0.27	0.136	6.248E-186	Monocytes	Tiparp
3.233E-190	0.44040297	0.684	0.466	8.016E-186	Monocytes	Tmed2
4.619E-190	0.43507721	0.373	0.206	1.145E-185	Monocytes	Erbin
1.008E-189	0.55106434	0.262	0.131	2.5E-185	Monocytes	Ankfy1
1.022E-189	0.5628551	0.327	0.175	2.535E-185	Monocytes	Nsf
3.211E-189	0.35473693	0.455	0.263	7.962E-185	Monocytes	Cdk9
6.09E-189	0.56057162	0.273	0.138	1.51E-184	Monocytes	Clcn4
6.884E-189	0.33779238	0.876	0.74	1.707E-184	Monocytes	Clic1
1.041E-188	0.39871692	0.369	0.205	2.581E-184	Monocytes	Pts
1.144E-188	0.37977665	0.717	0.511	2.837E-184	Monocytes	Macroh2a1
2.807E-188	0.43022668	0.364	0.201	6.96E-184	Monocytes	Tm9sf2
3.735E-188	0.41320133	0.513	0.309	9.26E-184	Monocytes	Txndc17
3.782E-188	0.4240632	0.349	0.189	9.379E-184	Monocytes	Cux1
6.438E-187	0.33053316	0.876	0.76	1.596E-182	Monocytes	Pabpc1
1.308E-186	0.31985499	0.525	0.316	3.243E-182	Monocytes	Inpp5d
7.188E-186	0.44125472	0.38	0.213	1.782E-181	Monocytes	Mlf2
7.97E-186	0.38949185	0.489	0.294	1.976E-181	Monocytes	Ier3ip1
3.58E-185	0.30531629	0.319	0.167	8.877E-181	Monocytes	Susd6
4E-185	0.56034885	0.284	0.148	9.919E-181	Monocytes	Tank
4.358E-185	0.4168727	0.345	0.188	1.08E-180	Monocytes	Vdac1
1.915E-184	0.49010744	0.289	0.149	4.749E-180	Monocytes	Rap1gds1
2.558E-184	0.47575383	0.438	0.259	6.342E-180	Monocytes	Ndufs2
4.178E-184	0.44368875	0.274	0.139	1.036E-179	Monocytes	Rbfa
7.678E-184	0.47326356	0.395	0.225	1.904E-179	Monocytes	Spag9
5.849E-183	0.47302118	0.374	0.209	1.45E-178	Monocytes	Ergic3
7.416E-183	0.48509591	0.33	0.179	1.839E-178	Monocytes	Pdha1
4.017E-182	0.46459201	0.362	0.202	9.96E-178	Monocytes	Mcts1
6.35E-182	0.47306223	0.332	0.181	1.574E-177	Monocytes	Ctdnep1
8.421E-182	0.36675709	0.82	0.62	2.088E-177	Monocytes	Tmbim6
5.769E-181	0.29641243	0.345	0.188	1.431E-176	Monocytes	Clip1
1.959E-180	0.4125146	0.453	0.269	4.856E-176	Monocytes	Ube2q1
1.826E-179	0.40362719	0.453	0.268	4.528E-175	Monocytes	Snf8
2.162E-179	0.41249412	0.42	0.243	5.362E-175	Monocytes	Uvrag
1.309E-178	0.45768889	0.462	0.278	3.245E-174	Monocytes	Selenos
1.734E-178	0.42629903	0.329	0.179	4.3E-174	Monocytes	Csnk1d
2.81E-178	0.50502913	0.358	0.201	6.968E-174	Monocytes	Clptm1l
1.355E-177	0.43357611	0.361	0.202	3.36E-173	Monocytes	Trappc2l
1.847E-177	0.48946807	0.272	0.141	4.579E-173	Monocytes	Rragc
1.917E-177	0.49903343	0.252	0.127	4.752E-173	Monocytes	Sppl3
1.217E-176	0.29606183	0.301	0.159	3.017E-172	Monocytes	Bin3
4.434E-174	0.33945344	0.293	0.155	1.099E-169	Monocytes	Azin1
4.908E-174	0.37256089	0.69	0.477	1.217E-169	Monocytes	Kif5b
7.764E-174	0.36014763	0.398	0.229	1.925E-169	Monocytes	Ensa
1.698E-173	0.32557304	0.432	0.254	4.211E-169	Monocytes	Ctnnb1
3.942E-173	0.4971449	0.376	0.215	9.775E-169	Monocytes	Eif3l

4.525E-173	0.390237	0.416	0.243	1.122E-168	Monocytes	Mia3
2.618E-172	0.31940652	0.769	0.629	6.491E-168	Monocytes	H2aj
6.215E-172	0.41884243	0.541	0.339	1.541E-167	Monocytes	Psmb2
6.839E-172	0.49418392	0.272	0.142	1.696E-167	Monocytes	Atp10d
6.943E-172	0.38228273	0.498	0.307	1.721E-167	Monocytes	Cops9
1.284E-171	0.36556165	0.318	0.172	3.183E-167	Monocytes	Atad2b
2.375E-171	0.45623967	0.304	0.164	5.889E-167	Monocytes	Cnot8
5.395E-171	0.41669357	0.459	0.276	1.338E-166	Monocytes	Cycs
5.81E-171	0.33773572	0.373	0.211	1.441E-166	Monocytes	Rnf115
1.117E-170	0.31641285	0.397	0.229	2.769E-166	Monocytes	Trpc4ap
1.318E-170	0.4322396	0.362	0.206	3.269E-166	Monocytes	Cul1
1.85E-169	0.27263728	0.277	0.144	4.586E-165	Monocytes	Fam241a
2.471E-169	0.36853042	0.671	0.455	6.126E-165	Monocytes	Eif3a
5.323E-169	0.39915284	0.695	0.481	1.32E-164	Monocytes	Ankrd44
7.615E-169	0.4897496	0.353	0.2	1.888E-164	Monocytes	Zc3h7a
1.263E-168	0.41351881	0.462	0.279	3.133E-164	Monocytes	Cope
1.057E-167	0.46260669	0.35	0.197	2.622E-163	Monocytes	Arl1
1.142E-167	0.3632248	0.987	0.971	2.832E-163	Monocytes	H2-D1
1.471E-167	0.43766429	0.329	0.182	3.649E-163	Monocytes	Mrpl4
1.669E-167	0.33046219	0.335	0.184	4.139E-163	Monocytes	Capg
1.691E-167	0.41005678	0.458	0.277	4.193E-163	Monocytes	Ube2n
1.058E-166	0.32898842	0.665	0.452	2.622E-162	Monocytes	Add3
4.286E-166	0.295152	0.267	0.139	1.063E-161	Monocytes	Dhx40
5.076E-166	0.34295349	0.376	0.215	1.259E-161	Monocytes	Tob2
2.457E-164	0.39644893	0.562	0.367	6.092E-160	Monocytes	Tmem167
2.605E-164	0.39794415	0.742	0.544	6.459E-160	Monocytes	Myl12b
1.053E-163	0.42864494	0.382	0.222	2.61E-159	Monocytes	Psmb7
1.75E-163	0.38446978	0.325	0.179	4.34E-159	Monocytes	Tbc1d20
2.688E-163	0.43544988	0.581	0.381	6.666E-159	Monocytes	Dbnl
3.1E-163	0.41441573	0.257	0.133	7.686E-159	Monocytes	Gxylt1
4.543E-163	0.28377442	0.366	0.206	1.126E-158	Monocytes	Vav1
6.112E-163	0.32030765	0.259	0.133	1.515E-158	Monocytes	Mtmr3
9.998E-163	0.48697704	0.306	0.168	2.479E-158	Monocytes	Zmiz2
2.936E-162	0.55389686	0.291	0.159	7.279E-158	Monocytes	Tgs1
2.946E-162	0.3538544	0.501	0.312	7.305E-158	Monocytes	Cast
3.784E-162	0.3855178	0.361	0.207	9.384E-158	Monocytes	Stt3b
4.488E-162	0.40459528	0.389	0.228	1.113E-157	Monocytes	Copb1
9.663E-162	0.57328042	0.262	0.139	2.396E-157	Monocytes	Pepd
1.345E-161	0.35523308	0.295	0.16	3.334E-157	Monocytes	2510039O18R
1.634E-161	0.37723919	0.296	0.161	4.051E-157	Monocytes	Strn3
1.701E-161	0.33745143	0.612	0.402	4.218E-157	Monocytes	Vapa
6.638E-161	0.39230119	0.305	0.165	1.646E-156	Monocytes	Prkch
1.003E-160	0.39909041	0.381	0.221	2.486E-156	Monocytes	Ebp
1.647E-160	0.36916369	0.295	0.16	4.084E-156	Monocytes	Golph3
1.998E-160	0.43553963	0.275	0.147	4.955E-156	Monocytes	Arrdc1
3.021E-160	0.33306128	0.349	0.197	7.49E-156	Monocytes	Sp1
5.883E-160	0.29718613	0.317	0.174	1.459E-155	Monocytes	Acaa1a
1.068E-159	0.32835978	0.486	0.299	2.649E-155	Monocytes	Zfp207
1.162E-159	0.289129	0.337	0.188	2.88E-155	Monocytes	Gmip
1.383E-159	0.38092398	0.393	0.229	3.429E-155	Monocytes	Skil

1.024E-158	0.29443511	0.301	0.163	2.538E-154	Monocytes	Ctdsp1
2.715E-158	0.35109096	0.391	0.228	6.732E-154	Monocytes	Morc3
3.148E-158	0.38035546	0.274	0.145	7.806E-154	Monocytes	Cd86
3.726E-158	0.28715962	0.473	0.288	9.24E-154	Monocytes	Stag2
4.687E-158	0.39752437	0.362	0.208	1.162E-153	Monocytes	Emd
5.948E-158	0.28838103	0.552	0.352	1.475E-153	Monocytes	Ralbp1
2.469E-157	0.44421538	0.66	0.516	6.123E-153	Monocytes	Ifi27l2a
4.255E-157	0.2943424	0.484	0.299	1.055E-152	Monocytes	Chmp5
2.192E-156	0.37566374	0.287	0.155	5.436E-152	Monocytes	Prxl2c
3.995E-156	0.31249536	0.62	0.409	9.905E-152	Monocytes	Tacc1
5.145E-156	0.3989694	0.286	0.154	1.276E-151	Monocytes	Gorasp2
7.207E-156	0.33975531	0.34	0.194	1.787E-151	Monocytes	Glrx2
7.412E-156	0.3746422	0.547	0.349	1.838E-151	Monocytes	Ndufa11
8.157E-156	0.40622252	0.35	0.201	2.022E-151	Monocytes	Snx17
1.423E-154	0.38006014	0.432	0.262	3.529E-150	Monocytes	Dync1i2
2.478E-154	0.42595358	0.403	0.242	6.145E-150	Monocytes	Chrac1
2.78E-154	0.31162855	0.676	0.494	6.893E-150	Monocytes	Gpi1
6.883E-154	0.42273408	0.317	0.178	1.707E-149	Monocytes	Szrd1
8.055E-154	0.42609262	0.359	0.209	1.997E-149	Monocytes	B230219D22R
1.456E-153	0.37075924	0.457	0.279	3.61E-149	Monocytes	Ap2m1
3.378E-153	0.41795064	0.39	0.227	8.376E-149	Monocytes	Ddost
6.998E-153	0.40513956	0.37	0.217	1.735E-148	Monocytes	Idh3g
8.235E-153	0.38776532	0.556	0.362	2.042E-148	Monocytes	Evl
1.052E-152	0.3180298	0.589	0.383	2.61E-148	Monocytes	Brd2
1.348E-152	0.41223234	0.419	0.255	3.342E-148	Monocytes	Galnt1
3.396E-152	0.38620889	0.41	0.245	8.42E-148	Monocytes	Uqcrc1
1.926E-150	0.45595129	0.285	0.157	4.775E-146	Monocytes	Tnpo1
3.45E-150	0.36511876	0.465	0.289	8.555E-146	Monocytes	Rheb
5.687E-150	0.41502258	0.271	0.146	1.41E-145	Monocytes	Sirt2
8.244E-150	0.30304789	0.656	0.447	2.044E-145	Monocytes	Cdkn1b
1.136E-149	0.27048219	0.333	0.188	2.816E-145	Monocytes	Msl2
1.614E-149	0.3012127	0.518	0.327	4.002E-145	Monocytes	Dctn3
3.101E-149	0.32194479	0.433	0.264	7.689E-145	Monocytes	Mrpl43
1.535E-148	0.35351915	0.602	0.402	3.805E-144	Monocytes	Psmb4
2.574E-148	0.33717328	0.653	0.448	6.382E-144	Monocytes	Psme2
2.779E-148	0.48209018	0.26	0.14	6.89E-144	Monocytes	Tpd52l2
3.902E-148	0.38223107	0.306	0.172	9.675E-144	Monocytes	Hmox2
2.487E-147	0.45862752	0.256	0.136	6.166E-143	Monocytes	Hltf
3.749E-147	0.36819534	0.318	0.18	9.296E-143	Monocytes	Poldip3
4.252E-147	0.42895701	0.304	0.171	1.054E-142	Monocytes	Tnpo3
2.517E-146	0.44238819	0.426	0.27	6.24E-142	Monocytes	Irf2bp2
6.424E-146	0.58096193	0.254	0.137	1.593E-141	Monocytes	Nus1
5.049E-145	0.49720693	0.255	0.138	1.252E-140	Monocytes	Sec31a
1.054E-144	0.34328381	0.455	0.283	2.613E-140	Monocytes	Mrpl20
3.501E-144	0.32414929	0.764	0.567	8.682E-140	Monocytes	Eif4g2
8.645E-144	0.45102757	0.252	0.136	2.144E-139	Monocytes	Triap1
2.157E-142	0.34253741	0.546	0.356	5.347E-138	Monocytes	Ube2l3
2.274E-142	0.32667506	0.487	0.307	5.639E-138	Monocytes	Rab11b
4.864E-142	0.34939015	0.389	0.236	1.206E-137	Monocytes	Fxr1
4.873E-142	0.27183153	0.344	0.2	1.208E-137	Monocytes	Setd1b

8.511E-142	0.3487833	0.47	0.295	2.11E-137	Monocytes	Ndufv2
3.03E-141	0.41856714	0.296	0.168	7.513E-137	Monocytes	Commd1
4.532E-141	0.33086153	0.33	0.19	1.124E-136	Monocytes	Smarcd2
1.019E-140	0.27773423	0.512	0.327	2.528E-136	Monocytes	Hnrnpul2
1.242E-140	0.37784448	0.429	0.265	3.08E-136	Monocytes	Eif4g1
1.016E-139	0.37427438	0.496	0.317	2.519E-135	Monocytes	Dnajc3
1.138E-139	0.30437505	0.326	0.187	2.82E-135	Monocytes	Arf3
1.297E-139	0.28832944	0.492	0.31	3.216E-135	Monocytes	Ppp4c
1.553E-139	0.29638714	0.428	0.263	3.85E-135	Monocytes	Trip12
2.123E-139	0.32386121	0.783	0.591	5.265E-135	Monocytes	Tmem50a
4.694E-139	0.28964041	0.821	0.664	1.164E-134	Monocytes	Serf2
4.703E-139	0.31215337	0.403	0.244	1.166E-134	Monocytes	Tmem179b
1.055E-138	0.30215225	0.407	0.247	2.615E-134	Monocytes	Psmd13
1.436E-138	0.38696907	0.484	0.311	3.56E-134	Monocytes	Mat2a
4.85E-138	0.38713586	0.315	0.183	1.203E-133	Monocytes	Morf4l2
9.306E-138	0.45595877	0.387	0.238	2.308E-133	Monocytes	Plec
6.227E-137	0.39225894	0.288	0.162	1.544E-132	Monocytes	Ogfr
8.222E-137	0.27318662	0.556	0.363	2.039E-132	Monocytes	Pkn1
3.393E-135	0.3594685	0.272	0.152	8.413E-131	Monocytes	Zzef1
9.166E-135	0.32350727	0.364	0.218	2.273E-130	Monocytes	0610012G03R
1.265E-134	0.27383225	0.382	0.23	3.138E-130	Monocytes	Uba1
2.195E-134	0.26309482	0.529	0.353	5.444E-130	Monocytes	Kpna4
2.447E-134	0.34099968	0.277	0.155	6.068E-130	Monocytes	Madd
3.284E-134	0.33670317	0.662	0.457	8.143E-130	Monocytes	Selenof
4.596E-134	0.28784326	0.442	0.276	1.139E-129	Monocytes	Mrps21
7.696E-134	0.35158095	0.424	0.262	1.908E-129	Monocytes	Rnf7
1.688E-133	0.45568074	0.251	0.138	4.184E-129	Monocytes	Ten1
2.191E-133	0.27592497	0.62	0.431	5.432E-129	Monocytes	Kras
2.468E-133	0.37441742	0.324	0.19	6.12E-129	Monocytes	Ptpra
5.102E-133	0.4216836	0.277	0.157	1.265E-128	Monocytes	Ap3b1
3.456E-132	0.31708438	0.413	0.253	8.57E-128	Monocytes	Eif4e2
4.986E-132	0.37099761	0.404	0.25	1.236E-127	Monocytes	Psma5
7.551E-132	0.37999019	0.272	0.153	1.872E-127	Monocytes	Mgat1
1.949E-131	0.30227553	0.255	0.14	4.833E-127	Monocytes	Arid1b
6.693E-130	0.2988964	0.434	0.273	1.66E-125	Monocytes	Ddx3y
9.84E-130	0.37956905	0.264	0.147	2.44E-125	Monocytes	Cdk2ap1
1.128E-129	0.3707705	0.315	0.183	2.796E-125	Monocytes	Rexo1
2.87E-129	0.28063659	0.537	0.352	7.117E-125	Monocytes	Prelid1
1.371E-128	0.27500491	0.346	0.205	3.398E-124	Monocytes	Baz2a
1.963E-128	0.35745893	0.365	0.222	4.868E-124	Monocytes	Tpp2
2.085E-127	0.27664876	0.755	0.574	5.171E-123	Monocytes	Atp6v1f
3.245E-127	0.30986468	0.626	0.429	8.045E-123	Monocytes	Lamtor2
3.813E-127	0.32026613	0.768	0.616	9.455E-123	Monocytes	Slc25a3
1.694E-126	0.35074762	0.288	0.165	4.199E-122	Monocytes	Stx8
7.353E-126	0.28723649	0.544	0.359	1.823E-121	Monocytes	Psma1
2.288E-125	0.32783445	0.276	0.157	5.672E-121	Monocytes	Sp140
4.13E-125	0.27480234	0.415	0.258	1.024E-120	Monocytes	Cisd2
1.984E-124	0.39608941	0.252	0.142	4.919E-120	Monocytes	Tm2d1
3.517E-124	0.36987856	0.323	0.193	8.721E-120	Monocytes	Unc50
1.829E-123	0.34737573	0.309	0.183	4.535E-119	Monocytes	Mrpl36

2.315E-123	0.33490401	0.286	0.165	5.74E-119	Monocytes	Rad23b
3.953E-123	0.41853577	0.265	0.15	9.802E-119	Monocytes	Aim2
1.725E-122	0.28534334	0.343	0.206	4.277E-118	Monocytes	Ccdc124
3.082E-121	0.26939612	0.477	0.305	7.643E-117	Monocytes	Rbm8a
3.451E-121	0.34221203	0.396	0.248	8.557E-117	Monocytes	Cenpx
4.931E-121	0.4378902	0.253	0.144	1.223E-116	Monocytes	Smarcc2
1.443E-120	0.25603906	0.3	0.175	3.577E-116	Monocytes	Ppp6r3
1.634E-120	0.30305234	0.366	0.224	4.051E-116	Monocytes	Mrps18c
2.201E-120	0.26625243	0.473	0.303	5.458E-116	Monocytes	Hsbp1
3.175E-120	0.45540087	0.253	0.144	7.873E-116	Monocytes	Ifrd1
4.824E-120	0.41149093	0.25	0.141	1.196E-115	Monocytes	Dpp8
2.438E-119	0.38789534	0.309	0.184	6.046E-115	Monocytes	Rnf4
3.26E-119	0.32676648	0.461	0.301	8.084E-115	Monocytes	Foxn3
4.328E-119	0.30044171	0.341	0.207	1.073E-114	Monocytes	Arhgef3
1.265E-118	0.35706836	0.293	0.172	3.138E-114	Monocytes	Ccny
1.606E-118	0.27139159	0.728	0.539	3.981E-114	Monocytes	Nedd8
1.79E-117	0.28105728	0.472	0.304	4.438E-113	Monocytes	Psmb5
2.402E-116	0.29868519	0.381	0.238	5.956E-112	Monocytes	Prrc2a
4.974E-116	0.27477103	0.554	0.371	1.233E-111	Monocytes	Psmb10
6.595E-116	0.31356641	0.447	0.288	1.635E-111	Monocytes	Sirt7
7.64E-116	0.29495357	0.396	0.248	1.894E-111	Monocytes	Nrbp1
8.928E-116	0.2660144	0.362	0.222	2.214E-111	Monocytes	Akirin2
9.436E-116	0.36399568	0.374	0.234	2.34E-111	Monocytes	Anapc16
1.154E-115	0.26670637	0.526	0.349	2.861E-111	Monocytes	Uqcr11
2.255E-115	0.26991495	0.456	0.294	5.592E-111	Monocytes	Bola2
2.841E-115	0.29218047	0.419	0.266	7.045E-111	Monocytes	Mrpl57
6.907E-115	0.29211382	0.328	0.198	1.713E-110	Monocytes	Elof1
1.185E-114	0.37652143	0.271	0.159	2.937E-110	Monocytes	Tmem33
4.119E-113	0.27783417	0.686	0.507	1.021E-108	Monocytes	Tmem234
7.872E-113	0.30121603	0.29	0.171	1.952E-108	Monocytes	Otub1
1.676E-112	0.30678709	0.572	0.396	4.157E-108	Monocytes	Ndufb5
1.731E-112	0.26575616	0.37	0.229	4.292E-108	Monocytes	Ogdh
2.632E-112	0.26339584	0.594	0.405	6.526E-108	Monocytes	Uqcrb
3.01E-112	0.30326361	0.357	0.221	7.463E-108	Monocytes	Erp44
3.214E-112	0.25563808	0.667	0.475	7.97E-108	Monocytes	Csde1
6.192E-112	0.3309163	0.388	0.247	1.535E-107	Monocytes	Baz1b
1.227E-111	0.29781337	0.286	0.169	3.044E-107	Monocytes	Zfp91.1
1.303E-111	0.35514139	0.263	0.153	3.23E-107	Monocytes	Tnip1
3.434E-111	0.37668937	0.257	0.148	8.514E-107	Monocytes	Mrps23
4.712E-111	0.25551271	0.343	0.211	1.168E-106	Monocytes	Vps4b
7.048E-111	0.32628796	0.392	0.249	1.748E-106	Monocytes	Srp72
7.764E-111	0.25611214	0.486	0.317	1.925E-106	Monocytes	Ndufa8
9.584E-111	0.27988218	0.348	0.213	2.376E-106	Monocytes	Rab18
4.031E-110	0.26615489	0.323	0.196	9.996E-106	Monocytes	Rnf6
8.17E-110	0.25338063	0.292	0.174	2.026E-105	Monocytes	Ccz1
1.519E-108	0.43078375	0.59	0.48	3.765E-104	Monocytes	Scand1
2.191E-108	0.36113867	0.265	0.156	5.432E-104	Monocytes	Sec13
5.017E-108	0.30020545	0.272	0.16	1.244E-103	Monocytes	Rnpepl1
2.276E-107	0.30428555	0.349	0.217	5.643E-103	Monocytes	Actn4
4.181E-107	0.35167077	0.29	0.175	1.037E-102	Monocytes	Emc2

6.254E-107	0.31980111	0.318	0.194	1.551E-102	Monocytes	March5
5.805E-106	0.2606356	0.278	0.165	1.439E-101	Monocytes	Polr2i
1.395E-105	0.2938965	0.381	0.243	3.458E-101	Monocytes	Romo1
2.976E-105	0.26716153	0.739	0.554	7.378E-101	Monocytes	Atp5a1
5.754E-105	0.30524799	0.266	0.158	1.427E-100	Monocytes	Smim7
7.477E-105	0.29592379	0.331	0.203	1.854E-100	Monocytes	Glmp
1.752E-104	0.34080802	0.263	0.156	4.343E-100	Monocytes	2610001J05Ri
2.248E-104	0.29624141	0.514	0.346	5.574E-100	Monocytes	Ostc
2.499E-104	0.29959458	0.494	0.335	6.195E-100	Monocytes	Birc6
3.749E-104	0.33924626	0.343	0.215	9.295E-100	Monocytes	Ak2
7.239E-104	0.28446378	0.376	0.239	1.795E-99	Monocytes	Ubn2
9.262E-103	0.27915484	0.637	0.459	2.2966E-98	Monocytes	Mycbp2
3.033E-102	0.25078278	0.279	0.167	7.5208E-98	Monocytes	Ubr2
2.249E-101	0.26548306	0.268	0.16	5.5765E-97	Monocytes	Sec22b
2.267E-101	0.3021286	0.315	0.195	5.6206E-97	Monocytes	Atraid
1.27E-100	0.31351482	0.312	0.194	3.1486E-96	Monocytes	Sumo3
2.196E-100	0.29353207	0.35	0.222	5.446E-96	Monocytes	Rex1bd
4.405E-100	0.29641262	0.292	0.178	1.0922E-95	Monocytes	Yipf3
3.691E-99	0.27635319	0.318	0.197	9.1527E-95	Monocytes	Stx4a
7.4E-99	0.2636519	0.392	0.254	1.8349E-94	Monocytes	Hspa4
3.2197E-98	0.27429658	0.382	0.246	7.9833E-94	Monocytes	Ssr2
6.0939E-98	0.2708267	0.372	0.241	1.511E-93	Monocytes	Syncrip
8.6548E-98	0.50497549	0.446	0.32	2.1459E-93	Monocytes	Cyfip2
1.2925E-97	0.29335927	0.61	0.442	3.2048E-93	Monocytes	Ikzf1
1.6134E-97	0.27081773	0.252	0.149	4.0005E-93	Monocytes	Oxa1l
1.883E-97	0.27494481	0.26	0.155	4.6689E-93	Monocytes	Hras
3.3034E-97	0.37958858	0.253	0.151	8.1907E-93	Monocytes	Slirp
1.0889E-96	0.30598835	0.305	0.188	2.6999E-92	Monocytes	Dapp1
2.2453E-96	0.30876955	0.263	0.159	5.5673E-92	Monocytes	Ddb1
5.8403E-96	0.26721917	0.296	0.183	1.4481E-91	Monocytes	Ino80d
1.1344E-95	0.3673553	0.255	0.154	2.8127E-91	Monocytes	Eri1
4.0465E-95	0.25485193	0.297	0.184	1.0033E-90	Monocytes	Ufm1
5.653E-95	0.25434764	0.739	0.577	1.4017E-90	Monocytes	Atp5pb
6.9292E-95	0.3261437	0.26	0.157	1.7181E-90	Monocytes	Usp1
2.2596E-94	0.29432887	0.363	0.234	5.6026E-90	Monocytes	Commd7
7.3639E-93	0.25269416	0.602	0.432	1.8259E-88	Monocytes	Vdac2
1.3636E-92	0.26501469	0.282	0.173	3.381E-88	Monocytes	Derl2
1.4906E-92	0.25591981	0.291	0.181	3.696E-88	Monocytes	Gapvd1
2.319E-91	0.26185072	0.691	0.504	5.75E-87	Monocytes	Taf10
4.3943E-89	0.25500719	0.353	0.229	1.0896E-84	Monocytes	Dhx15
1.01E-88	0.30540093	0.256	0.157	2.5042E-84	Monocytes	Ubr5
2.0606E-88	0.28347739	0.27	0.167	5.1093E-84	Monocytes	Immt
4.9085E-87	0.3040009	0.286	0.18	1.2171E-82	Monocytes	Bdp1
2.5371E-85	0.27828962	0.251	0.153	6.2908E-81	Monocytes	Ndufv1
2.7608E-75	0.27048639	0.255	0.16	6.8455E-71	Monocytes	Rnf220
3.0026E-73	0.73984998	0.285	0.199	7.4449E-69	Monocytes	Pecam1
2.6678E-69	0.26713232	0.257	0.165	6.6147E-65	Monocytes	Zfp800
2.0683E-68	0.25930504	0.253	0.164	5.1282E-64	Monocytes	Kpnb1
0	3.0233196	0.87	0.113	0	DCs	Rassf4
0	3.11132989	0.872	0.122	0	DCs	Cbfa2t3

0	3.22860146	0.925	0.182	0	DCs	H2-DMb1
0	3.0326883	0.842	0.1	0	DCs	Ccdc88a
0	2.89701674	0.876	0.139	0	DCs	Plbd1
0	2.78363462	0.873	0.16	0	DCs	Ccr2
0	3.84820629	0.782	0.073	0	DCs	Tmem176b
0	2.05738687	0.83	0.126	0	DCs	Grn
0	2.07452604	0.856	0.16	0	DCs	Plekho1
0	2.09110929	0.812	0.12	0	DCs	Cd68
0	2.28636666	0.914	0.229	0	DCs	Anxa5
0	2.3688509	0.891	0.214	0	DCs	Ifitm3
0	4.03909786	0.72	0.05	0	DCs	Pid1
0	4.1951494	0.7	0.036	0	DCs	Rnd3
0	4.58800871	0.696	0.034	0	DCs	Olfm1
0	3.94599513	0.72	0.06	0	DCs	Tmem176a
0	1.92978793	0.84	0.18	0	DCs	Pld4
0	5.42676025	0.674	0.019	0	DCs	Batf3
0	1.93135943	0.89	0.238	0	DCs	Ptms
0	1.81397066	0.854	0.203	0	DCs	Evi2a
0	0.68720193	0.937	0.287	0	DCs	Alox5ap
0	2.1237723	0.897	0.252	0	DCs	Syngr2
0	1.88123105	0.764	0.12	0	DCs	App
0	1.95314026	0.885	0.245	0	DCs	S100a4
0	2.64671144	0.771	0.131	0	DCs	Ms4a6c
0	2.6133994	0.731	0.093	0	DCs	Cfp
0	2.61204445	0.921	0.292	0	DCs	Ifi30
0	3.56444821	0.675	0.047	0	DCs	Nav1
0	2.09696824	0.962	0.337	0	DCs	H2-DMa
0	1.73566492	0.768	0.143	0	DCs	Csf2ra
0	1.60610999	0.818	0.194	0	DCs	Hexa
0	1.50071679	0.763	0.141	0	DCs	Trf
0	0.73158027	0.908	0.287	0	DCs	Ifitm2
0	1.73540905	0.795	0.175	0	DCs	Irf5
0	2.26458513	0.753	0.139	0	DCs	Klf4
0	0.64104006	0.915	0.303	0	DCs	Spi1
0	1.73514334	0.949	0.34	0	DCs	Ctsh
0	2.2039393	0.703	0.095	0	DCs	Alcam
0	0.79900754	0.965	0.358	0	DCs	Fcer1g
0	1.3482703	0.911	0.305	0	DCs	Ly86
0	1.62757861	0.788	0.187	0	DCs	Sh3bgrl
0	2.40209087	0.723	0.123	0	DCs	Rbpj
0	1.5583972	0.793	0.199	0	DCs	Itm2c
0	3.40137689	0.687	0.093	0	DCs	Naaa
0	1.39583182	0.883	0.292	0	DCs	Kctd12
0	3.19504157	0.637	0.048	0	DCs	Tifab
0	1.17489961	0.909	0.32	0	DCs	Unc93b1
0	1.10315491	0.891	0.305	0	DCs	Ctsb
0	0.84344256	0.82	0.234	0	DCs	Skap2
0	1.43644205	0.892	0.307	0	DCs	Rrbp1
0	2.14425714	0.717	0.137	0	DCs	Pepd
0	3.39044118	0.622	0.044	0	DCs	Ppfia4

0	0.57048683	0.82	0.242	0	DCs	Myadm
0	2.93497448	0.627	0.051	0	DCs	Ptpro
0	1.75108905	0.97	0.395	0	DCs	Ctsz
0	1.42361185	0.846	0.272	0	DCs	Gltp
0	1.73903373	0.721	0.147	0	DCs	Ciita
0	1.54316175	0.702	0.131	0	DCs	5031439G07R
0	3.70419684	0.603	0.033	0	DCs	Pak1
0	1.55501764	0.756	0.187	0	DCs	Rnase6
0	1.40649241	0.843	0.278	0	DCs	Atpif1
0	2.16003434	0.694	0.13	0	DCs	BC028528
0	1.46406301	0.787	0.224	0	DCs	Rnh1
0	1.93666204	0.699	0.138	0	DCs	Mpp6
0	1.92461004	0.735	0.175	0	DCs	Sdf2l1
0	1.13755437	0.717	0.158	0	DCs	Wfdc17
0	5.35878275	0.572	0.016	0	DCs	Clec4b1
0	1.07850232	0.808	0.255	0	DCs	Pip4k2a
0	1.71355654	0.696	0.143	0	DCs	Mpeg1
0	0.9468153	0.809	0.258	0	DCs	Prkcd
0	1.10635591	0.838	0.287	0	DCs	Glud1
0	1.28901001	0.739	0.189	0	DCs	Ehd4
0	1.49797406	0.722	0.172	0	DCs	Nrros
0	1.6171268	0.777	0.229	0	DCs	Zeb2
0	1.57978533	0.705	0.158	0	DCs	Tmed3
0	1.25467321	0.758	0.212	0	DCs	P4hb
0	1.36016396	0.745	0.199	0	DCs	Slk
0	1.08137328	0.843	0.3	0	DCs	Efhd2
0	1.23557561	0.894	0.353	0	DCs	Rps27l
0	1.44807202	0.699	0.158	0	DCs	Gsn
0	1.04651063	0.854	0.313	0	DCs	Srsf9
0	3.03826549	0.601	0.06	0	DCs	Tep1
0	1.71399035	0.656	0.117	0	DCs	Asah1
0	1.1113968	0.829	0.29	0	DCs	Dbi
0	3.26165002	0.619	0.081	0	DCs	Ccl9
0	1.97986003	0.645	0.108	0	DCs	Ifitm6
0	1.23460284	0.709	0.173	0	DCs	Ramp1
0	1.06143821	0.756	0.222	0	DCs	Stx7
0	2.09384704	0.622	0.09	0	DCs	Fgd2
0	1.01252504	0.789	0.259	0	DCs	H13
0	1.15112218	0.901	0.371	0	DCs	Akr1a1
0	0.56430457	0.719	0.189	0	DCs	Pirb
0	0.94690342	0.803	0.273	0	DCs	Nptn
0	1.23740998	0.801	0.272	0	DCs	Irf2bp2
0	1.50569636	0.692	0.165	0	DCs	Tmem256
0	2.59489768	0.605	0.079	0	DCs	Naga
0	1.84463944	0.896	0.37	0	DCs	Ahnak
0	1.6232364	0.775	0.25	0	DCs	Bst2
0	1.15087153	0.841	0.316	0	DCs	Pdia6
0	1.05099642	0.854	0.33	0	DCs	Cyb5a
0	1.36902024	0.959	0.436	0	DCs	Erp29
0	0.88451084	0.867	0.345	0	DCs	Rtn4

0	2.27032454	0.923	0.401	0	DCs	H2-Eb1
0	0.98867043	0.807	0.286	0	DCs	Taok3
0	2.66344416	0.608	0.087	0	DCs	Fcgrt
0	2.06053975	0.633	0.112	0	DCs	Ctnna1
0	2.43809129	0.936	0.416	0	DCs	H2-Ab1
0	0.67053971	0.803	0.285	0	DCs	Rnf130
0	1.90273487	0.952	0.435	0	DCs	Gm2a
0	2.19995179	0.956	0.442	0	DCs	H2-Aa
0	1.98463335	0.592	0.079	0	DCs	Acer3
0	0.95727512	0.899	0.387	0	DCs	Napsa
0	1.02022345	0.837	0.326	0	DCs	Psma4
0	1.58819736	0.638	0.128	0	DCs	Bri3bp
0	4.35996795	0.536	0.026	0	DCs	Aif1
0	2.32054709	0.621	0.112	0	DCs	Atf3
0	0.71195689	0.685	0.177	0	DCs	Fam129a
0	0.88203695	0.72	0.213	0	DCs	Cnih4
0	4.40729639	0.531	0.024	0	DCs	Shtn1
0	1.68396203	0.967	0.46	0	DCs	Ctss
0	1.01667493	0.648	0.142	0	DCs	St3gal4
0	5.6403797	0.519	0.015	0	DCs	Cd209a
0	1.74706674	0.924	0.421	0	DCs	Atox1
0	0.82000012	0.91	0.409	0	DCs	Anxa2
0	2.96118683	0.546	0.045	0	DCs	Cebpa
0	0.84192163	0.847	0.349	0	DCs	Cyth4
0	0.93479789	0.708	0.212	0	DCs	Mapkapk2
0	1.36666376	0.618	0.122	0	DCs	Zfp710
0	0.79899713	0.629	0.133	0	DCs	Fes
0	1.25935394	0.721	0.226	0	DCs	Otulinl
0	1.34631223	0.918	0.424	0	DCs	Lgals3
0	0.8756607	0.753	0.259	0	DCs	Tm6sf1
0	1.61504275	0.641	0.147	0	DCs	Pnp
0	1.57208347	0.643	0.15	0	DCs	Colgalt1
0	0.73291221	0.635	0.142	0	DCs	Csf2rb
0	0.88888516	0.826	0.333	0	DCs	Canx
0	1.47674203	0.682	0.19	0	DCs	Ybx3
0	0.97102386	0.71	0.218	0	DCs	Eid1
0	0.68013594	0.737	0.245	0	DCs	Ncf1
0	2.88273162	0.535	0.045	0	DCs	Hfe
0	1.67852777	0.605	0.115	0	DCs	Cyfip1
0	2.38712102	0.565	0.076	0	DCs	Dpy19l1
0	1.03417332	0.915	0.427	0	DCs	Ywhae
0	0.85536788	0.76	0.272	0	DCs	Ndufc2
0	3.74579314	0.523	0.036	0	DCs	Avpi1
0	0.59216614	0.735	0.248	0	DCs	Orai1
0	0.98577472	0.693	0.206	0	DCs	Etfb
0	1.41690091	0.631	0.144	0	DCs	Ap2a2
0	1.0021901	0.921	0.436	0	DCs	Ssr4
0	1.54242837	0.77	0.285	0	DCs	Ctsc
0	0.99373419	0.658	0.173	0	DCs	Plekho2
0	0.86332599	0.711	0.227	0	DCs	Ssr3

0	1.160549	0.921	0.438	0	DCs	Pomp
0	0.71923727	0.743	0.26	0	DCs	Bnip2
0	0.81302857	0.8	0.318	0	DCs	Ctsa
0	1.64950841	0.607	0.125	0	DCs	Gusb
0	3.14639322	0.526	0.045	0	DCs	Cmtm3
0	2.57210451	0.537	0.056	0	DCs	Tiam1
0	0.81378101	0.706	0.225	0	DCs	Xbp1
0	1.08434263	0.91	0.43	0	DCs	Smdt1
0	0.87714756	0.745	0.266	0	DCs	Cd2ap
0	2.31778589	0.522	0.045	0	DCs	Grk3
0	0.88182266	0.745	0.268	0	DCs	Cybb
0	0.8217344	0.689	0.212	0	DCs	Atp6ap2
0	0.50323064	0.669	0.193	0	DCs	Cd300a
0	0.56539438	0.704	0.228	0	DCs	Slc6a6
0	0.79550483	0.72	0.244	0	DCs	AI662270
0	3.96357027	0.504	0.028	0	DCs	Dapk1
0	0.99430628	0.663	0.188	0	DCs	Jarid2
0	2.08036814	0.544	0.07	0	DCs	Dnajb14
0	0.88095637	0.681	0.208	0	DCs	Lat2
0	0.99222293	0.719	0.246	0	DCs	Rala
0	1.08992599	0.664	0.192	0	DCs	Fkbp2
0	0.90371761	0.702	0.23	0	DCs	Ddost
0	1.0628522	0.616	0.146	0	DCs	Tm2d2
0	1.14826082	0.639	0.169	0	DCs	Zmiz2
0	0.85475612	0.743	0.273	0	DCs	Ppp1r14b
0	1.37589708	0.57	0.101	0	DCs	Plin2
0	0.89880128	0.651	0.183	0	DCs	Rsu1
0	0.83337824	0.695	0.227	0	DCs	Ccdc50
0	1.64343988	0.559	0.092	0	DCs	Rp2
0	2.67983874	0.517	0.051	0	DCs	Ltb4r1
0	1.03616029	0.835	0.369	0	DCs	Lamp1
0	1.19776858	0.619	0.154	0	DCs	Camk1d
0	0.86467517	0.678	0.215	0	DCs	Scamp2
0	1.02036596	0.637	0.174	0	DCs	Ywhag
0	3.66275828	0.493	0.031	0	DCs	Gatm
0	1.00549129	0.688	0.226	0	DCs	Fbl
0	0.87097519	0.628	0.168	0	DCs	Sgk3
0	0.94074968	0.739	0.28	0	DCs	Cycs
0	1.63086662	0.893	0.435	0	DCs	Lyz2
0	1.28032596	0.61	0.152	0	DCs	Ube2f
0	0.88915154	0.615	0.157	0	DCs	Sirpa
0	0.80384452	0.692	0.234	0	DCs	Ssr1
0	0.27949386	0.674	0.218	0	DCs	Creg1
0	0.7594597	0.695	0.239	0	DCs	Sept9
0	1.27301307	0.651	0.196	0	DCs	Unc119
0	0.93248716	0.686	0.233	0	DCs	Prdx2
0	1.02094204	0.686	0.233	0	DCs	Uqcc2
0	5.0794172	0.466	0.013	0	DCs	Flt3
0	1.43195829	0.579	0.126	0	DCs	Lactb
0	3.59760857	0.486	0.034	0	DCs	Eps8

0	1.53676768	0.584	0.132	0	DCs	Dpysl2
0	1.26765463	0.639	0.188	0	DCs	Hexb
0	1.31759767	0.594	0.143	0	DCs	Iqgap2
0	0.69181214	0.677	0.228	0	DCs	Myo1g
0	0.7406402	0.678	0.229	0	DCs	Spag9
0	3.48825337	0.487	0.038	0	DCs	Plxnd1
0	0.98692167	0.608	0.16	0	DCs	Cuedc2
0	2.07939443	0.531	0.085	0	DCs	Sigmar1
0	0.99322038	0.638	0.192	0	DCs	Mtch1
0	1.0629557	0.655	0.21	0	DCs	Trappc5
0	2.90022505	0.483	0.038	0	DCs	Trerf1
0	2.02404661	0.552	0.108	0	DCs	Pmaip1
0	1.20071867	0.705	0.261	0	DCs	Ccl6
0	0.72779909	0.595	0.151	0	DCs	Trps1
0	1.72181223	0.526	0.083	0	DCs	Clec4a2
0	0.89848136	0.669	0.227	0	DCs	M6pr
0	3.1442511	0.468	0.026	0	DCs	Fgfr1
0	0.81171655	0.658	0.217	0	DCs	Bloc1s2
0	2.35212089	0.504	0.063	0	DCs	Raph1
0	1.51492674	0.57	0.129	0	DCs	Capn2
0	1.25835673	0.567	0.126	0	DCs	Leprot
0	0.47488789	0.57	0.129	0	DCs	Lrrc25
0	1.1293329	0.613	0.174	0	DCs	Rpn1
0	0.83039872	0.638	0.2	0	DCs	Magt1
0	1.10995193	0.605	0.169	0	DCs	Fh1
0	2.85657282	0.484	0.05	0	DCs	Cfh
0	0.42845724	0.571	0.139	0	DCs	Cd300c2
0	0.57547361	0.637	0.206	0	DCs	Lmo4
0	0.86477742	0.937	0.506	0	DCs	Capza2
0	0.80824582	0.622	0.191	0	DCs	Vdac1
0	0.72314935	0.623	0.192	0	DCs	Cnpy3
0	1.66940169	0.536	0.106	0	DCs	Dpp4
0	0.86646879	0.607	0.178	0	DCs	Necap2
0	1.05649533	0.946	0.517	0	DCs	Psma7
0	0.71313713	0.637	0.21	0	DCs	Hdlbp
0	1.49171649	0.621	0.196	0	DCs	Selenop
0	1.85832814	0.495	0.071	0	DCs	Tifa
0	2.06612647	0.509	0.085	0	DCs	Bcl2a1d
0	0.44307773	0.622	0.198	0	DCs	Ifngr2
0	1.06026552	0.953	0.529	0	DCs	Npc2
0	1.18894345	0.508	0.085	0	DCs	Clec4a3
0	1.08536452	0.562	0.14	0	DCs	Csf1r
0	1.53384243	0.489	0.067	0	DCs	Clec4a1
0	1.93434146	0.492	0.07	0	DCs	Plxnb2
0	0.99501391	0.953	0.531	0	DCs	Eif4a1
0	1.18670013	0.579	0.158	0	DCs	Oxct1
0	0.81540466	0.608	0.189	0	DCs	Dapp1
0	0.88759944	0.587	0.168	0	DCs	Cebpg
0	0.82256981	0.635	0.217	0	DCs	Bmyc
0	2.26744573	0.486	0.068	0	DCs	Tubb2a

0	1.28374532	0.533	0.116	0	DCs	Coro1c
0	1.00247803	0.573	0.156	0	DCs	Osgep
0	1.83478469	0.985	0.569	0	DCs	Cd74
0	0.65668683	0.613	0.197	0	DCs	Crlf2
0	1.37990198	0.605	0.189	0	DCs	Fosb
0	3.25292783	0.445	0.029	0	DCs	P2ry6
0	1.92229057	0.978	0.562	0	DCs	Psap
0	1.03861753	0.571	0.156	0	DCs	Tmem147
0	1.61021087	0.493	0.079	0	DCs	Lrp1
0	2.65430501	0.441	0.028	0	DCs	Amz1
0	0.71549597	0.612	0.199	0	DCs	Me2
0	4.65562159	0.432	0.02	0	DCs	Slamf9
0	1.84170252	0.489	0.077	0	DCs	Elmo2
0	1.00357092	0.53	0.119	0	DCs	Borcs6
0	1.2504041	0.557	0.146	0	DCs	Cd86
0	1.28417016	0.516	0.106	0	DCs	Comt
0	4.41373309	0.435	0.025	0	DCs	Clec10a
0	0.87236148	0.541	0.132	0	DCs	Ehbp1l1
0	1.2281893	0.626	0.218	0	DCs	Tcf4
0	4.10347946	0.426	0.018	0	DCs	Cysltr1
0	1.10357088	0.509	0.102	0	DCs	Snx10
0	1.38433647	0.528	0.122	0	DCs	Scpep1
0	0.84654802	0.587	0.181	0	DCs	Srebf2
0	2.92144706	0.463	0.058	0	DCs	Tppp3
0	1.01353171	0.562	0.157	0	DCs	Ppm1m
0	1.42138268	0.922	0.518	0	DCs	Macroh2a1
0	1.88549633	0.467	0.064	0	DCs	1700025G04R
0	1.29617551	0.509	0.106	0	DCs	Slc35c2
0	1.79765188	0.479	0.076	0	DCs	Igsf8
0	0.72511745	0.586	0.184	0	DCs	Morf4l2
0	1.58292089	0.485	0.084	0	DCs	Plin3
0	0.93819784	0.519	0.12	0	DCs	Mvp
0	1.27287914	0.5	0.101	0	DCs	Lpcat1
0	1.07007325	0.569	0.17	0	DCs	Tpi1
0	0.68583758	0.533	0.135	0	DCs	Synj1
0	0.87052814	0.572	0.174	0	DCs	Vrk1
0	0.81342999	0.554	0.157	0	DCs	Aph1a
0	1.90435625	0.456	0.059	0	DCs	Clec12a
0	1.44656049	0.973	0.576	0	DCs	Gpx1
0	4.57674171	0.421	0.025	0	DCs	Mcub
0	1.16021427	0.508	0.113	0	DCs	Lrrfip2
0	1.01803989	0.536	0.142	0	DCs	Itpr1
0	0.74074941	0.537	0.143	0	DCs	Bach1
0	0.97419239	0.537	0.144	0	DCs	P2rx4
0	2.87222108	0.429	0.037	0	DCs	Ece1
0	1.9441771	0.466	0.074	0	DCs	Dusp3
0	1.22247091	0.535	0.144	0	DCs	Ahcyl2
0	2.32957564	0.43	0.04	0	DCs	Sdc3
0	0.84057044	0.508	0.118	0	DCs	Camk1
0	0.29547124	0.541	0.152	0	DCs	Themis2

0	2.49270625	0.456	0.067	0	DCs	Gas7
0	1.81500465	0.466	0.078	0	DCs	Nop16
0	2.68724176	0.436	0.049	0	DCs	Anxa4
0	1.33642409	0.481	0.095	0	DCs	Parp8
0	1.30572157	0.477	0.092	0	DCs	Prcp
0	1.64480755	0.467	0.082	0	DCs	Dusp22
0	1.32412885	0.499	0.114	0	DCs	Il6st
0	2.45844069	0.463	0.079	0	DCs	Lmna
0	0.84634197	0.539	0.156	0	DCs	Cnpy2
0	1.37528478	0.456	0.074	0	DCs	Klra17
0	0.98924385	0.528	0.146	0	DCs	Map4k1
0	1.4221997	0.501	0.119	0	DCs	Icam1
0	1.35010154	0.475	0.094	0	DCs	Abcd1
0	0.96374064	0.539	0.158	0	DCs	Cldnd1
0	0.69230788	0.539	0.159	0	DCs	Plekhf2
0	2.29191839	0.429	0.049	0	DCs	Slc31a2
0	2.9252427	0.413	0.033	0	DCs	Adgrg5
0	1.07164817	0.507	0.128	0	DCs	Hsd17b12
0	1.91100038	0.448	0.069	0	DCs	Fam102b
0	2.90819438	0.436	0.057	0	DCs	Emp1
0	3.07468901	0.406	0.027	0	DCs	Nectin1
0	0.82639213	0.526	0.148	0	DCs	Il10ra
0	0.79134941	0.522	0.145	0	DCs	Elovl1
0	0.97323421	0.955	0.579	0	DCs	Calr
0	1.47623861	0.508	0.133	0	DCs	Wdfy4
0	2.23675208	0.412	0.038	0	DCs	Zfp516
0	1.46794098	0.455	0.082	0	DCs	Etv6
0	1.39594527	0.474	0.101	0	DCs	Lrrk1
0	0.93647729	0.497	0.125	0	DCs	Ddi2
0	1.10795449	0.485	0.113	0	DCs	Kdm2b
0	2.17172513	0.429	0.057	0	DCs	Slc29a3
0	2.27481161	0.422	0.05	0	DCs	Vwa5a
0	1.1602225	0.483	0.111	0	DCs	Bak1
0	1.22741204	0.483	0.111	0	DCs	Isoc1
0	1.50362152	0.471	0.1	0	DCs	Clic4
0	1.17287836	0.44	0.069	0	DCs	Itpripl2
0	1.11946485	0.451	0.081	0	DCs	Ralb
0	1.08410745	0.918	0.549	0	DCs	Tagln2
0	3.45064221	0.403	0.034	0	DCs	Ccnd1
0	0.83269585	0.519	0.15	0	DCs	Spcs3
0	1.57193237	0.46	0.092	0	DCs	Ccr5
0	0.80438003	0.528	0.16	0	DCs	Jak2
0	0.79144462	0.514	0.149	0	DCs	Atp2a2
0	3.2234524	0.399	0.034	0	DCs	Mt1
0	0.53934496	0.489	0.124	0	DCs	Gnaq
0	0.87141881	0.503	0.139	0	DCs	Uggt1
0	0.74013999	0.519	0.155	0	DCs	Tmem219
0	8.03949177	0.365	0.002	0	DCs	Mrc1
0	1.19165129	0.454	0.091	0	DCs	Arhgap18
0	1.56391416	0.437	0.074	0	DCs	Cx3cr1

0	3.827603	0.379	0.016	0	DCs	Acvrl1
0	0.77618826	0.497	0.135	0	DCs	Plekhm3
0	0.68795327	0.488	0.126	0	DCs	Dstn
0	2.8884562	0.392	0.03	0	DCs	Zfp385a
0	0.84027082	0.514	0.153	0	DCs	Ptpa
0	1.11147776	0.429	0.068	0	DCs	Itgax
0	0.91734758	0.48	0.12	0	DCs	Lemd2
0	2.03029776	0.403	0.044	0	DCs	Tnfrsf21
0	1.35504833	0.445	0.086	0	DCs	Agpat4
0	1.62480465	0.435	0.077	0	DCs	Nenf
0	0.99654774	0.479	0.122	0	DCs	Cyb5r3
0	2.41441965	0.396	0.04	0	DCs	Ptprs
0	2.73192363	0.401	0.046	0	DCs	Ldlr
0	0.66616799	0.504	0.149	0	DCs	Tor1a
0	1.0205885	0.474	0.119	0	DCs	Pafah1b3
0	0.70869645	0.509	0.154	0	DCs	Lipa
0	0.9519596	0.457	0.103	0	DCs	Cep170
0	0.85138292	0.488	0.134	0	DCs	Slc15a4
0	1.96535586	0.414	0.06	0	DCs	Ank
0	1.48805919	0.446	0.092	0	DCs	Slc48a1
0	0.28459046	0.484	0.13	0	DCs	Pqlc1
0	1.57791558	0.442	0.088	0	DCs	Scd2
0	1.80746278	0.399	0.046	0	DCs	Rgs18
0	2.98177124	0.387	0.034	0	DCs	Marveld1
0	1.25472621	0.47	0.118	0	DCs	Cdkn1a
0	1.41263097	0.441	0.09	0	DCs	Pea15a
0	0.76057255	0.493	0.142	0	DCs	Snx1
0	2.00900907	0.4	0.05	0	DCs	Stx3
0	0.82976605	0.426	0.076	0	DCs	Il13ra1
0	4.28570054	0.369	0.019	0	DCs	Cd300lg
0	1.30069963	0.446	0.096	0	DCs	Irf4
0	1.36104824	0.482	0.132	0	DCs	Pdlim1
0	2.24841754	0.393	0.044	0	DCs	Ptov1
0	0.86315226	0.459	0.11	0	DCs	Sgpl1
0	0.67789152	0.493	0.144	0	DCs	Rragc
0	1.3298842	0.445	0.096	0	DCs	Tspan31
0	0.99184192	0.441	0.092	0	DCs	Tmem50b
0	0.91110963	0.471	0.122	0	DCs	Snx9
0	1.1122053	0.442	0.094	0	DCs	Ids
0	1.61541795	0.456	0.109	0	DCs	Fn1
0	0.93190852	0.463	0.116	0	DCs	Prkd3
0	2.42813362	0.38	0.033	0	DCs	Dna2
0	0.83528352	0.484	0.137	0	DCs	Anxa7
0	0.92164808	0.476	0.129	0	DCs	Fam49a
0	4.06070894	0.366	0.019	0	DCs	A530064D06R
0	2.47859124	0.392	0.047	0	DCs	Tubb6
0	0.84088263	0.481	0.136	0	DCs	Ormdl2
0	2.61119619	0.384	0.041	0	DCs	Ryr1
0	1.05388515	0.456	0.113	0	DCs	Lrrc59
0	1.02938093	0.446	0.104	0	DCs	Trafd1

0	1.95669388	0.965	0.625	0	DCs	Vim
0	1.36352959	0.424	0.086	0	DCs	Prkar2a
0	0.99855696	0.476	0.138	0	DCs	Txndc15
0	1.99544243	0.392	0.055	0	DCs	BC035044
0	0.91662341	0.434	0.097	0	DCs	Rnpep
0	0.402621	0.47	0.133	0	DCs	Ccrl2
0	2.89525507	0.369	0.033	0	DCs	F13a1
0	2.34499331	0.456	0.121	0	DCs	Pltp
0	2.49050744	0.389	0.054	0	DCs	Adam15
0	0.7999949	0.449	0.114	0	DCs	Coro2a
0	2.12412207	0.375	0.04	0	DCs	St3gal5
0	2.82159617	0.368	0.033	0	DCs	Stom
0	1.1358362	0.427	0.092	0	DCs	Plekha1
0	0.72292804	0.465	0.131	0	DCs	Acadm
0	0.81780274	0.459	0.125	0	DCs	Pkib
0	1.51540157	0.427	0.093	0	DCs	Fcgr2b
0	0.81224872	0.449	0.116	0	DCs	Atf6
0	5.37319281	0.34	0.007	0	DCs	Slamf8
0	2.58700085	0.366	0.033	0	DCs	Sulf2
0	3.27850081	0.349	0.016	0	DCs	Cadm1
0	3.49288823	0.355	0.022	0	DCs	9830107B12R
0	1.03388147	0.444	0.112	0	DCs	Arhgap5
0	0.93397852	0.412	0.08	0	DCs	Rab32
0	1.2101801	0.438	0.107	0	DCs	Marcksl1
0	0.69779799	0.463	0.132	0	DCs	Ipo7
0	1.32401514	0.417	0.086	0	DCs	Slc39a1
0	0.85548343	0.446	0.116	0	DCs	Tfg
0	1.39457396	0.419	0.089	0	DCs	Sfxn3
0	3.72328116	0.351	0.022	0	DCs	Abca9
0	0.91431063	0.443	0.115	0	DCs	Abhd12
0	1.69563691	0.382	0.054	0	DCs	S100a1
0	1.81470479	0.379	0.051	0	DCs	Nrp1
0	1.37344771	0.404	0.076	0	DCs	Rap2a
0	1.62692522	0.394	0.067	0	DCs	Bcat2
0	2.06591236	0.375	0.048	0	DCs	Zfhx3
0	1.20199642	0.425	0.098	0	DCs	Tmem109
0	1.51613089	0.385	0.059	0	DCs	Phactr2
0	0.6483695	0.42	0.094	0	DCs	Ptafr
0	1.74875988	0.387	0.062	0	DCs	Idh1
0	1.73279617	0.375	0.05	0	DCs	Gpr35
0	1.38256952	0.394	0.069	0	DCs	Milr1
0	2.67345764	0.36	0.035	0	DCs	Bcl2a1a
0	1.60106265	0.425	0.1	0	DCs	Lgmn
0	1.27927077	0.406	0.084	0	DCs	Aldh9a1
0	0.80548	0.427	0.105	0	DCs	Arhgap26.1
0	1.29844826	0.4	0.078	0	DCs	Hsd17b4
0	1.25742267	0.412	0.09	0	DCs	Dctpp1
0	3.44079033	0.344	0.023	0	DCs	Tnni2
0	0.88945508	0.437	0.117	0	DCs	Npepl1
0	1.73690156	0.375	0.055	0	DCs	Tax1bp3

0	0.89332475	0.403	0.083	0	DCs	Gpr141
0	2.12062935	0.367	0.048	0	DCs	Scarb1
0	3.47365506	0.334	0.016	0	DCs	Cd209d
0	3.04861894	0.344	0.026	0	DCs	Rasa4
0	2.16297918	0.36	0.043	0	DCs	Afdn
0	1.20467498	0.405	0.089	0	DCs	Lrrc58
0	3.53587347	0.333	0.017	0	DCs	Upb1
0	1.09135007	0.411	0.095	0	DCs	Hipk2
0	0.70521463	0.436	0.121	0	DCs	Rras
0	1.45878307	0.378	0.063	0	DCs	Pacs2
0	0.98166698	0.421	0.107	0	DCs	Yif1b
0	0.67697433	0.419	0.105	0	DCs	Sap30
0	0.96794551	0.439	0.125	0	DCs	Slfn5
0	0.76086822	0.428	0.115	0	DCs	Mbtd1
0	1.0667138	0.42	0.107	0	DCs	Cndp2
0	1.48120409	0.391	0.079	0	DCs	Nfkbie
0	1.0115783	0.405	0.094	0	DCs	Prdx4
0	1.05056614	0.403	0.092	0	DCs	Adssl1
0	2.28292649	0.958	0.647	0	DCs	Crip1
0	2.05907619	0.354	0.044	0	DCs	Basp1
0	2.04867788	0.358	0.049	0	DCs	Cd302
0	0.87424206	0.42	0.111	0	DCs	Abca3
0	1.14956902	0.415	0.108	0	DCs	Ass1
0	1.07052505	0.408	0.102	0	DCs	Gns
0	2.18401659	0.341	0.036	0	DCs	Hk2
0	1.36045211	0.395	0.09	0	DCs	Idh2
0	1.41263258	0.379	0.074	0	DCs	Nucb2
0	2.13421592	0.371	0.068	0	DCs	Fdps
0	1.29190949	0.387	0.084	0	DCs	Dnmt3a
0	0.83525792	0.412	0.11	0	DCs	Noc2l
0	0.92237492	0.416	0.114	0	DCs	Camkk2
0	0.97242404	0.392	0.091	0	DCs	Stard3
0	3.5983927	0.317	0.016	0	DCs	Stxbp6
0	0.84634634	0.405	0.105	0	DCs	Myo5a
0	2.23960351	0.332	0.033	0	DCs	Cdk14
0	1.02069919	0.393	0.094	0	DCs	Spr
0	3.28661286	0.323	0.024	0	DCs	Tbxas1
0	1.02502939	0.972	0.673	0	DCs	H2az1
0	0.83502635	0.403	0.105	0	DCs	Gch1
0	2.09743919	0.341	0.043	0	DCs	Ext1
0	1.31173376	0.378	0.08	0	DCs	Dhrs3
0	0.26681596	0.387	0.09	0	DCs	Rasgrp4
0	0.83884668	0.396	0.099	0	DCs	Ufsp2
0	2.27408444	0.341	0.044	0	DCs	Ahr
0	1.41807551	0.362	0.065	0	DCs	Kmo
0	5.99397148	0.304	0.007	0	DCs	Tnip3
0	1.2123112	0.364	0.067	0	DCs	Rnf141
0	1.09265857	0.365	0.069	0	DCs	Bmpr2
0	7.23296352	0.305	0.009	0	DCs	Mgl2
0	2.62372228	0.332	0.037	0	DCs	Frmd4a

0	1.03157633	0.399	0.104	0	DCs	Snd1
0	1.13116394	0.383	0.088	0	DCs	Ppp1r21
0	5.65536422	0.306	0.011	0	DCs	Rab7b
0	0.3895537	0.375	0.08	0	DCs	Ctnnbip1
0	0.97062437	0.377	0.083	0	DCs	Phf10
0	1.75146938	0.355	0.061	0	DCs	Gsto1
0	0.82922974	0.397	0.104	0	DCs	Mrps28
0	0.96163493	0.385	0.092	0	DCs	Ap1b1
0	0.83426415	0.391	0.098	0	DCs	Pacsin2
0	5.68216999	0.304	0.011	0	DCs	Dab2
0	1.86449266	0.341	0.05	0	DCs	Dnase1l1
0	1.58643356	0.347	0.056	0	DCs	Ccdc28b
0	1.43773033	0.363	0.072	0	DCs	Akr7a5
0	0.96750879	0.958	0.667	0	DCs	Ucp2
0	2.20336169	0.326	0.036	0	DCs	Sowahc
0	2.00037356	0.329	0.04	0	DCs	Prkra
0	0.97541458	0.367	0.078	0	DCs	Vcl
0	6.58335245	0.29	0.002	0	DCs	Tspan33
0	1.40261497	0.358	0.07	0	DCs	Arsb
0	1.22203903	0.379	0.092	0	DCs	Slamf7
0	2.09655023	0.34	0.053	0	DCs	Cyp51
0	1.96254981	0.329	0.042	0	DCs	Pdxk
0	1.30626941	0.329	0.042	0	DCs	Mctp1
0	0.88779821	0.378	0.092	0	DCs	Fundc1
0	0.97186341	0.374	0.088	0	DCs	Vrk2
0	3.56759012	0.311	0.025	0	DCs	Myof
0	1.09843406	0.357	0.072	0	DCs	Mtmr14
0	1.15578096	0.365	0.081	0	DCs	Ptpmt1
0	0.92892624	0.368	0.084	0	DCs	Tyk2
0	1.09398967	0.357	0.073	0	DCs	Erlin1
0	0.9693317	0.367	0.084	0	DCs	Fam98c
0	2.83608298	0.306	0.023	0	DCs	Ccdc102a
0	0.85405446	0.369	0.087	0	DCs	Plod3
0	1.96824842	0.337	0.055	0	DCs	Net1
0	0.8315405	0.368	0.086	0	DCs	Map3k11
0	2.65446926	0.311	0.029	0	DCs	Slc46a3
0	2.01634012	0.328	0.048	0	DCs	Mgst3
0	0.48789641	0.373	0.093	0	DCs	Apobr
0	1.92486647	0.308	0.028	0	DCs	Klrb1b
0	1.72485966	0.343	0.065	0	DCs	Rogdi
0	0.77781381	0.365	0.088	0	DCs	Arl3
0	1.49214015	0.354	0.078	0	DCs	Nedd4
0	1.66583911	0.356	0.08	0	DCs	Hmgcs1
0	2.35217165	0.306	0.031	0	DCs	Tbc1d8
0	2.28492327	0.321	0.046	0	DCs	Gm6377
0	2.19932018	0.314	0.039	0	DCs	Pros1
0	1.64208288	0.325	0.051	0	DCs	Abcc1
0	0.88551226	0.365	0.092	0	DCs	Eef1akmt1
0	0.88909154	0.362	0.089	0	DCs	Mfap3
0	1.17807797	0.347	0.074	0	DCs	Nkiras2

0	1.37791566	0.345	0.073	0	DCs	Pianp
0	1.71616407	0.317	0.046	0	DCs	Gna12
0	1.14418857	0.331	0.061	0	DCs	Itprid2
0	1.94929634	0.313	0.043	0	DCs	Nfix
0	1.08684102	0.337	0.067	0	DCs	Krt80
0	1.18458289	0.348	0.078	0	DCs	Fbrsl1
0	1.51584244	0.328	0.059	0	DCs	Apex1
0	1.03348639	0.338	0.069	0	DCs	Rarg
0	2.97531084	0.291	0.022	0	DCs	Cyp27a1
0	0.99550623	0.346	0.079	0	DCs	Khk
0	1.35127892	0.332	0.067	0	DCs	Plbd2
0	5.07664963	0.271	0.006	0	DCs	Celf4
0	2.20735017	0.301	0.037	0	DCs	Tmem141
0	1.85465735	0.306	0.042	0	DCs	Itpripl1
0	4.00325775	0.28	0.016	0	DCs	Sult1a1
0	1.50944703	0.313	0.049	0	DCs	Matk
0	0.9611354	0.317	0.053	0	DCs	Arl11
0	0.87381597	0.334	0.072	0	DCs	Inpp4a
0	4.23601719	0.275	0.013	0	DCs	Gm48582
0	1.73303217	0.321	0.059	0	DCs	H1f0
0	0.94352961	0.347	0.085	0	DCs	Mthfd2
0	1.39216614	0.33	0.069	0	DCs	Plxnc1
0	1.36100059	0.321	0.061	0	DCs	Tspan3
0	2.79529304	0.288	0.028	0	DCs	Tmem106a
0	1.3050566	0.318	0.059	0	DCs	Mospd2
0	1.56146421	0.319	0.06	0	DCs	Daglb
0	1.7485529	0.304	0.045	0	DCs	Plscr3
0	1.00619484	0.334	0.076	0	DCs	Ube2e3
0	0.56389031	0.34	0.082	0	DCs	Tsc22d1
0	1.11143536	0.334	0.077	0	DCs	Sdad1
0	3.16263251	0.279	0.022	0	DCs	Lacc1
0	1.11787958	0.332	0.076	0	DCs	Pmvk
0	1.27770968	0.312	0.056	0	DCs	I830077J02Rik
0	0.75866646	0.315	0.059	0	DCs	Arrdc4
0	1.29564321	0.331	0.075	0	DCs	Msmo1
0	1.6535587	0.3	0.045	0	DCs	Tmem51
0	1.0902749	0.308	0.053	0	DCs	Svip
0	1.70447053	0.297	0.043	0	DCs	Myo9a
0	5.64174012	0.265	0.011	0	DCs	Cxcl16
0	0.85353111	0.328	0.075	0	DCs	Plcb2
0	1.77115284	0.294	0.041	0	DCs	Mob3b
0	1.4396125	0.303	0.05	0	DCs	Hps5
0	1.31025176	0.313	0.061	0	DCs	Xpnpep1
0	1.60654326	0.296	0.045	0	DCs	Pgap2
0	2.34745954	0.298	0.047	0	DCs	Socs6
0	1.10670655	0.316	0.066	0	DCs	Eif4e3
0	1.02065908	0.3	0.05	0	DCs	Ltbr
0	1.0965007	0.317	0.067	0	DCs	Heatr5a
0	2.27261654	0.275	0.027	0	DCs	Chn2
0	4.97468368	0.268	0.02	0	DCs	Lpl

0	1.3348672	0.306	0.058	0	DCs	Nagpa
0	1.98786933	0.287	0.039	0	DCs	2810025M15R
0	2.31698331	0.283	0.035	0	DCs	Lifr
0	2.34754003	0.277	0.03	0	DCs	Ifi211
0	1.04031331	0.321	0.075	0	DCs	Samd1
0	2.22629875	0.995	0.75	0	DCs	Cst3
0	3.29188941	0.26	0.015	0	DCs	Plpp2
0	0.8223658	0.318	0.073	0	DCs	Mib1
0	1.28126608	0.299	0.056	0	DCs	Arf2
0	2.97794839	0.261	0.019	0	DCs	Tns3
0	1.24456088	0.297	0.055	0	DCs	Evi5
0	1.00791714	0.313	0.071	0	DCs	Tcirg1
0	1.47496877	0.289	0.048	0	DCs	Slc8b1
0	2.07927322	0.261	0.021	0	DCs	Aph1c
0	1.26970364	0.3	0.06	0	DCs	Piezo1
0	2.3949426	0.274	0.035	0	DCs	Rtl8a
0	2.04127446	0.267	0.028	0	DCs	Pik3cb
0	3.18602001	0.259	0.021	0	DCs	Cpq
0	1.09019567	0.309	0.071	0	DCs	Gba
0	0.84148189	0.295	0.057	0	DCs	Rell1
0	1.3872523	0.295	0.057	0	DCs	Tmem238
0	1.24760675	0.303	0.066	0	DCs	Adap1
0	0.99108107	0.303	0.067	0	DCs	Hlcs
0	1.87524089	0.28	0.045	0	DCs	Ckb
0	1.53908235	0.267	0.032	0	DCs	AI839979
0	1.3845182	0.296	0.061	0	DCs	Insig1
0	1.18224902	0.294	0.059	0	DCs	Fam91a1
0	1.85126524	0.295	0.06	0	DCs	Ms4a6d
0	3.19358283	0.26	0.026	0	DCs	Crip2
0	0.89277483	0.3	0.067	0	DCs	Arrb1
0	0.7794618	0.288	0.057	0	DCs	Tlr2
0	0.83659974	0.298	0.067	0	DCs	Slc25a45
0	8.80034502	0.255	0.027	0	DCs	Retnla
0	2.49079005	0.255	0.028	0	DCs	Spred1
0	2.17489163	0.262	0.035	0	DCs	Vat1
0	1.19454396	0.28	0.054	0	DCs	Fbxo6
0	1.3215639	0.276	0.05	0	DCs	Pnpo
0	1.88197316	0.262	0.037	0	DCs	Tle1
0	1.69680399	0.266	0.041	0	DCs	Tspan17
0	0.965023	0.278	0.054	0	DCs	Slc12a9
0	2.25692608	0.252	0.028	0	DCs	Luzp1
0	1.4907176	0.274	0.052	0	DCs	Zcchc24
0	1.58747593	0.258	0.037	0	DCs	Ctbp2
0	1.44595326	0.274	0.054	0	DCs	Hs6st1
0	3.05633369	0.265	0.045	0	DCs	Mafb
0	1.5483639	0.265	0.045	0	DCs	9930120I10Ri
0	0.79616273	0.279	0.059	0	DCs	Wdfy2
0	0.7041841	0.27	0.051	0	DCs	Rbm47
0	0.88106368	0.264	0.045	0	DCs	Ebi3
0	1.19735415	0.254	0.035	0	DCs	Pik3r6

0	1.87166071	0.266	0.048	0	DCs	Idi1
0	1.34515091	0.271	0.054	0	DCs	Lap3
0	1.26553015	0.271	0.054	0	DCs	Nagk
0	1.42869801	0.256	0.042	0	DCs	Mtmr4
0	1.16764274	0.267	0.054	0	DCs	Inppl1
0	1.49352412	0.264	0.052	0	DCs	Ecm1
0	1.36706616	0.259	0.048	0	DCs	Stn1
0	1.59664447	0.257	0.05	0	DCs	Cd36
0	0.93999789	0.251	0.045	0	DCs	Smim3
0	1.15943016	0.257	0.052	0	DCs	Trio
0	1.24741805	0.251	0.047	0	DCs	Crybg3
0	0.86697278	0.962	0.806	0	DCs	Lsp1
0	0.89276202	0.987	0.892	0	DCs	Actg1
4.812E-307	0.40078986	0.582	0.182	1.193E-302	DCs	Hck
5.594E-307	0.59085556	0.461	0.131	1.387E-302	DCs	Scarb2
6.447E-307	0.51139429	0.566	0.175	1.599E-302	DCs	Plcg2
2.257E-306	0.4204111	0.502	0.148	5.597E-302	DCs	Tnrc18
4.891E-306	0.6649902	0.551	0.17	1.213E-301	DCs	Commd1
5.2E-306	0.63622614	0.33	0.079	1.289E-301	DCs	Vav3
2.043E-305	0.74637878	0.572	0.183	5.066E-301	DCs	Mvb12a
2.572E-305	0.94224772	0.703	0.261	6.377E-301	DCs	Tuba1b
3.092E-305	0.92274552	0.321	0.076	7.667E-301	DCs	Pi4k2a
1.142E-304	0.44400901	0.691	0.238	2.831E-300	DCs	Reep3
1.316E-304	0.73992068	0.706	0.247	3.262E-300	DCs	Ssr2
1.612E-304	1.01716283	0.3	0.068	3.996E-300	DCs	Asl
4.842E-304	0.64638593	0.445	0.125	1.201E-299	DCs	Fam117b
6.55E-304	1.30010146	0.291	0.066	1.624E-299	DCs	Gbp3
1.732E-303	1.08376119	0.258	0.053	4.295E-299	DCs	Lrrc42
3.69E-303	2.33033182	0.267	0.059	9.15E-299	DCs	Tbc1d4
7.268E-303	0.75729581	0.577	0.184	1.802E-298	DCs	Ctdnep1
7.29E-303	0.81791671	0.363	0.093	1.807E-298	DCs	Bad
8.861E-303	0.79935583	0.412	0.112	2.197E-298	DCs	Slc35b1
2.247E-302	0.77648491	0.478	0.14	5.572E-298	DCs	Polb
2.551E-302	0.97163138	0.334	0.081	6.325E-298	DCs	L1cam
2.74E-302	0.9595331	0.94	0.604	6.794E-298	DCs	Sec61b
4.384E-302	0.85876815	0.876	0.387	1.087E-297	DCs	Cd48
7.923E-302	0.80173867	0.498	0.15	1.965E-297	DCs	Siva1
8.131E-302	0.72143094	0.57	0.18	2.016E-297	DCs	Mydgf
1.228E-301	0.83483192	0.363	0.093	3.045E-297	DCs	Htra2
1.358E-300	0.92319004	0.387	0.103	3.368E-296	DCs	Eci2
2.823E-300	0.81454281	0.344	0.085	7.001E-296	DCs	Cpt1a
3.502E-300	0.95285849	0.255	0.053	8.684E-296	DCs	Nhsl2
1.351E-299	0.97327872	0.437	0.126	3.35E-295	DCs	Hmgcr
1.667E-299	0.71452111	0.658	0.224	4.134E-295	DCs	Mrpl54
3.789E-299	0.70586802	0.47	0.137	9.395E-295	DCs	Acsl5
5.491E-299	0.69791093	0.492	0.147	1.361E-294	DCs	Mrpl17
5.895E-299	0.66338947	0.327	0.079	1.462E-294	DCs	Tfe3
7.865E-299	0.92842458	0.91	0.446	1.95E-294	DCs	Pdia3
8.668E-299	0.61060176	0.579	0.185	2.149E-294	DCs	Eps15
1.303E-298	0.6391657	0.623	0.205	3.23E-294	DCs	Dctn2

2.097E-298	0.81463268	0.354	0.09	5.2E-294	DCs	Papss1
3.081E-298	0.90350673	0.283	0.063	7.64E-294	DCs	Klhl5
3.152E-298	0.57159071	0.639	0.215	7.815E-294	DCs	Far1
1.524E-297	0.5276878	0.398	0.107	3.78E-293	DCs	G6pdx
1.639E-297	0.63830324	0.719	0.259	4.064E-293	DCs	Fuca1
2.194E-297	0.61775608	0.512	0.156	5.441E-293	DCs	Mfsd14a
1.47E-296	0.86425173	0.394	0.106	3.645E-292	DCs	Eif2a
2.522E-296	0.65221778	0.854	0.365	6.254E-292	DCs	Pycard
3.45E-296	0.27173123	0.586	0.185	8.554E-292	DCs	Sema4a
7.052E-296	0.87502938	0.353	0.09	1.748E-291	DCs	Wdr3
7.703E-296	0.66964127	0.585	0.189	1.91E-291	DCs	Mgat2
1.461E-295	0.32271798	0.609	0.197	3.621E-291	DCs	Mapk14
2.296E-295	0.65950152	0.558	0.182	5.693E-291	DCs	Metrnl
3.984E-295	0.70822682	0.529	0.163	9.877E-291	DCs	Naa38
2.425E-294	0.87894226	0.341	0.085	6.014E-290	DCs	Usp12
2.663E-294	0.70056328	0.411	0.114	6.603E-290	DCs	Slc43a2
3.953E-294	0.67841185	0.63	0.211	9.8E-290	DCs	Ube2e1
5.24E-294	0.38733304	0.464	0.134	1.299E-289	DCs	Fem1c
5.834E-294	1.10761709	0.262	0.056	1.446E-289	DCs	Fam234a
1.344E-293	0.66140658	0.474	0.141	3.334E-289	DCs	Lyl1
2.032E-292	1.04620184	0.283	0.064	5.039E-288	DCs	Casp6
2.554E-292	0.69093598	0.441	0.127	6.332E-288	DCs	Bmp2k
3.007E-292	0.74342368	0.894	0.385	7.455E-288	DCs	Bri3
1.264E-291	0.93836341	0.294	0.068	3.133E-287	DCs	Ptrhd1
1.576E-291	1.12413218	0.261	0.056	3.908E-287	DCs	Slc25a1
2.423E-291	0.67141031	0.43	0.121	6.008E-287	DCs	Plekhb2
3.944E-291	0.68100404	0.519	0.161	9.779E-287	DCs	Trappc1
5.263E-291	0.43480338	0.498	0.15	1.305E-286	DCs	Map7d1
7.897E-291	0.36775734	0.342	0.085	1.958E-286	DCs	Relt
1.229E-290	0.40191227	0.556	0.174	3.048E-286	DCs	Mapk3
3.073E-290	0.68633658	0.55	0.174	7.619E-286	DCs	Bcl7c
9.658E-290	0.72611546	0.383	0.102	2.395E-285	DCs	Meaf6
1.473E-289	0.96687025	0.701	0.271	3.652E-285	DCs	Tuba1a
1.637E-289	0.85722657	0.331	0.083	4.058E-285	DCs	Wls
2.789E-289	0.43143191	0.374	0.099	6.914E-285	DCs	Rab31
3.011E-289	0.62840712	0.632	0.214	7.466E-285	DCs	Fam174a
2.187E-288	0.73685106	0.481	0.144	5.423E-284	DCs	Sdhc
4.899E-288	0.86933742	0.357	0.093	1.215E-283	DCs	Dok1
1.086E-287	0.82748407	0.251	0.053	2.692E-283	DCs	Impa2
1.982E-287	0.62755079	0.62	0.209	4.914E-283	DCs	Stx16
2.511E-287	1.00790608	0.289	0.067	6.225E-283	DCs	Elp5
6.298E-287	0.47846492	0.559	0.177	1.562E-282	DCs	Acaa1a
8.526E-287	0.84608492	0.27	0.06	2.114E-282	DCs	Sirt3
2.311E-286	0.70250503	0.454	0.134	5.731E-282	DCs	Ethe1
7.849E-286	0.64428374	0.75	0.28	1.946E-281	DCs	Twf2
1.31E-285	0.73295019	0.625	0.214	3.249E-281	DCs	Nhp2
1.484E-285	0.69212049	0.51	0.158	3.68E-281	DCs	Mrps18a
2.582E-285	0.69466916	0.511	0.158	6.402E-281	DCs	Mrpl28
7.283E-285	0.66171055	0.641	0.222	1.806E-280	DCs	Arhgef6
1.079E-284	0.65846548	0.509	0.16	2.676E-280	DCs	Sh3bp1

1.292E-284	0.58338756	0.339	0.086	3.204E-280	DCs	Adgre1
2.569E-284	0.72578264	0.806	0.324	6.371E-280	DCs	Tkt
3.914E-283	0.57143651	0.472	0.141	9.705E-279	DCs	Fam168a
2.976E-282	0.77393542	0.762	0.295	7.38E-278	DCs	Herpud1
1.229E-281	0.29945851	0.705	0.247	3.046E-277	DCs	Il6ra
1.468E-281	0.61810135	0.625	0.211	3.639E-277	DCs	Spg21
1.184E-280	0.66072417	0.744	0.278	2.935E-276	DCs	Cltc
1.805E-280	0.77060488	0.495	0.153	4.476E-276	DCs	Bscl2
9.375E-280	0.77028	0.339	0.087	2.325E-275	DCs	9130401M01F
5.098E-279	0.71034022	0.719	0.262	1.264E-274	DCs	Vdac3
7.35E-279	0.69678247	0.392	0.108	1.823E-274	DCs	Mrto4
1.323E-278	0.63872873	0.569	0.187	3.279E-274	DCs	Fam120a
1.708E-278	0.64502286	0.555	0.183	4.234E-274	DCs	Edem1
4.04E-278	0.83963492	0.311	0.076	1.002E-273	DCs	Thyn1
3.006E-277	0.42824805	0.321	0.08	7.454E-273	DCs	Slc15a3
3.007E-277	0.6142683	0.555	0.18	7.455E-273	DCs	Larp1
3.31E-277	0.96783774	0.936	0.596	8.207E-273	DCs	Emp3
6.81E-277	0.72114641	0.603	0.205	1.688E-272	DCs	C1qbp
7.79E-277	0.83891379	0.343	0.089	1.931E-272	DCs	Hyou1
9.788E-277	0.55233707	0.749	0.279	2.427E-272	DCs	Qk
3.408E-276	0.58323223	0.563	0.185	8.45E-272	DCs	Tet3
7.132E-276	0.65198798	0.628	0.213	1.768E-271	DCs	Ergic3
1.053E-275	0.97443815	0.461	0.143	2.61E-271	DCs	Phf11b
1.555E-275	0.74258389	0.284	0.067	3.855E-271	DCs	Apaf1
1.585E-275	0.66971477	0.394	0.11	3.931E-271	DCs	Prkx
4.452E-275	0.70377711	0.665	0.24	1.104E-270	DCs	Cstb
7.305E-275	0.51215683	0.339	0.087	1.811E-270	DCs	Csgalnact2
1.901E-274	0.69670857	0.314	0.078	4.713E-270	DCs	Klf10
1.981E-274	0.7720733	0.924	0.446	4.911E-270	DCs	Atp6v0b
2.1E-274	0.75106053	0.326	0.082	5.206E-270	DCs	Gmpr2
2.242E-274	0.4907236	0.263	0.059	5.558E-270	DCs	Slc2a6
5.113E-274	0.73368178	0.293	0.07	1.268E-269	DCs	Trim41
1.105E-273	0.66888026	0.554	0.182	2.74E-269	DCs	Dipk1a
1.123E-273	0.96683055	0.307	0.076	2.784E-269	DCs	Atp13a2
2.989E-273	0.63302718	0.504	0.159	7.41E-269	DCs	Eif1a
4.687E-273	0.85289562	0.545	0.187	1.162E-268	DCs	Sept11
9.315E-273	0.59650035	0.561	0.183	2.31E-268	DCs	Psmg4
9.979E-273	0.91386934	0.375	0.104	2.474E-268	DCs	Creld2
1.355E-272	0.51364096	0.497	0.155	3.359E-268	DCs	Stxbp2
1.717E-272	0.47081361	0.524	0.166	4.257E-268	DCs	Hpcal1
1.906E-272	0.85322078	0.915	0.495	4.725E-268	DCs	Pitpna
2.362E-272	0.69708309	0.394	0.11	5.858E-268	DCs	Slc25a39
2.711E-272	0.54138154	0.475	0.146	6.721E-268	DCs	Dcaf12
1.633E-271	0.36577577	0.708	0.255	4.049E-267	DCs	Lamp2
2.468E-271	0.29164549	0.661	0.225	6.119E-267	DCs	Lmo2
2.618E-271	0.66437663	0.69	0.25	6.491E-267	DCs	Tbcb
5.089E-271	0.73803828	0.694	0.254	1.262E-266	DCs	Txn2
5.966E-271	0.80025642	0.413	0.12	1.479E-266	DCs	Ptcd2
1.084E-270	0.71156296	0.48	0.148	2.688E-266	DCs	Pigt
1.225E-270	0.78262709	0.863	0.397	3.038E-266	DCs	Ywhah

3.836E-270	0.73592411	0.757	0.29	9.51E-266	DCs	Sdhb
4.425E-270	0.67192291	0.63	0.219	1.097E-265	DCs	Eif3l
4.674E-270	0.55442087	0.473	0.146	1.159E-265	DCs	Pttg1ip
1.496E-269	0.63343019	0.467	0.142	3.71E-265	DCs	Stxbp3
2.161E-269	0.56740916	0.588	0.198	5.358E-265	DCs	Surf4
9.179E-269	1.1247582	0.419	0.135	2.276E-264	DCs	Egr1
1.09E-268	0.7781672	0.865	0.38	2.702E-264	DCs	Capns1
3.531E-268	0.51750956	0.505	0.159	8.754E-264	DCs	Vamp3
1.574E-267	0.66901129	0.671	0.242	3.902E-263	DCs	Ech1
4.717E-267	0.8655403	0.293	0.071	1.17E-262	DCs	Ado
5.959E-267	0.7431914	0.351	0.094	1.477E-262	DCs	Glod4
5.997E-267	0.75803563	0.336	0.087	1.487E-262	DCs	Unc119b
8.552E-267	0.49013418	0.668	0.239	2.12E-262	DCs	Nckap1l
1.14E-266	0.96391386	0.278	0.066	2.827E-262	DCs	Tlnrd1
1.446E-266	0.48569617	0.497	0.156	3.586E-262	DCs	Nfic
3.752E-265	0.72248275	0.391	0.111	9.302E-261	DCs	Polr2h
4.988E-265	0.65951534	0.371	0.103	1.237E-260	DCs	Cers5
6.799E-265	0.54541743	0.533	0.172	1.686E-260	DCs	Otub1
2.121E-264	0.81893189	0.35	0.094	5.26E-260	DCs	Naxe
3.325E-264	0.85575801	0.298	0.074	8.245E-260	DCs	Gna15
4.637E-264	0.77231385	0.437	0.132	1.15E-259	DCs	Atic
5.107E-264	0.53660465	0.574	0.193	1.266E-259	DCs	Vps35
1.124E-263	0.68120661	0.365	0.101	2.787E-259	DCs	Mfsd1
1.64E-263	0.77149754	0.269	0.062	4.065E-259	DCs	Vac14
2.168E-263	0.63319643	0.752	0.327	5.376E-259	DCs	Lgals1
2.43E-263	0.36692146	0.551	0.181	6.026E-259	DCs	Chp1
4.004E-263	0.71001707	0.362	0.099	9.928E-259	DCs	Stard3nl
5.453E-262	0.62082555	0.634	0.226	1.352E-257	DCs	Lims1
6.143E-262	0.44109007	0.544	0.18	1.523E-257	DCs	Rab8b
1.07E-261	0.49150132	0.399	0.114	2.652E-257	DCs	Ankle2
1.441E-261	0.49718059	0.532	0.172	3.573E-257	DCs	Cic
2.192E-261	0.51076481	0.497	0.157	5.434E-257	DCs	Arl8a
2.828E-261	0.5031628	0.585	0.198	7.012E-257	DCs	Pip4p1
1.353E-259	0.65753354	0.654	0.234	3.354E-255	DCs	Glrx3
1.488E-259	0.35021365	0.378	0.106	3.691E-255	DCs	Fuca2
2.144E-259	0.5910412	0.395	0.113	5.316E-255	DCs	Vps26a
1.236E-258	0.57442309	0.386	0.109	3.066E-254	DCs	Atf6b
1.277E-258	0.53286357	0.46	0.142	3.166E-254	DCs	Ptpn11
3.125E-258	0.43948588	0.431	0.129	7.749E-254	DCs	Pdlim5
3.481E-258	0.46893227	0.602	0.209	8.632E-254	DCs	Akt1
4.419E-258	0.66132111	0.463	0.144	1.096E-253	DCs	Nup210
4.905E-258	0.58237375	0.531	0.175	1.216E-253	DCs	Hmox2
6.45E-258	0.5142665	0.507	0.162	1.599E-253	DCs	Casp1
2.349E-257	0.58973236	0.414	0.123	5.824E-253	DCs	Arl8b
2.879E-257	0.73064269	0.417	0.124	7.139E-253	DCs	Ccdc115
3.374E-257	0.63871653	0.417	0.123	8.367E-253	DCs	Lrpap1
5.114E-257	0.48341252	0.568	0.19	1.268E-252	DCs	Copz1
2.787E-256	0.51951644	0.457	0.14	6.909E-252	DCs	Iah1
4.146E-256	0.56708338	0.491	0.156	1.028E-251	DCs	Nfu1
1.811E-255	0.51131745	0.426	0.127	4.489E-251	DCs	Cyb5r4

5.355E-255	0.67058477	0.602	0.215	1.328E-250	DCs	Runx1
8.243E-255	0.61759308	0.481	0.152	2.044E-250	DCs	Slirp
1.052E-254	0.42231769	0.555	0.187	2.61E-250	DCs	Ctdsp2
1.226E-254	0.67478309	0.351	0.096	3.041E-250	DCs	Gpr108
2.842E-254	1.22450313	0.43	0.14	7.046E-250	DCs	Bhlhe40
4.42E-254	0.49012727	0.563	0.19	1.096E-249	DCs	Arf3
7.196E-254	0.68506242	0.426	0.13	1.784E-249	DCs	Knop1
8.497E-254	0.54426737	0.46	0.143	2.107E-249	DCs	Rbfa
1.039E-253	0.63595788	0.46	0.143	2.577E-249	DCs	Paics
1.093E-253	0.5934859	0.746	0.29	2.71E-249	DCs	Degs1
2.4E-253	0.71812781	0.326	0.086	5.95E-249	DCs	Gipc1
2.523E-253	0.69007216	0.775	0.308	6.255E-249	DCs	Lamtor1
6.151E-253	0.70780311	0.712	0.276	1.525E-248	DCs	Sptssa
7.21E-253	0.59545063	0.362	0.101	1.788E-248	DCs	Tent2
7.283E-253	0.60831628	0.292	0.073	1.806E-248	DCs	Pdcd2l
8.712E-253	0.56185014	0.446	0.138	2.16E-248	DCs	Chd9
2.484E-252	0.36687924	0.382	0.109	6.159E-248	DCs	Casp3
3.241E-252	0.67549048	0.68	0.253	8.036E-248	DCs	Psma5
4.612E-252	1.00201106	0.254	0.059	1.143E-247	DCs	Nupr1
6.603E-252	0.71049361	0.873	0.399	1.637E-247	DCs	Ubl3
1.006E-251	0.66820629	0.359	0.1	2.494E-247	DCs	Siah2
2.293E-251	0.70760725	0.782	0.315	5.686E-247	DCs	Txndc17
2.178E-250	0.53324739	0.524	0.174	5.399E-246	DCs	Rraga
2.388E-250	0.48383458	0.481	0.154	5.922E-246	DCs	Usp8
2.497E-250	1.07663568	0.282	0.07	6.191E-246	DCs	Ifi204
4.81E-250	0.60120142	0.425	0.129	1.193E-245	DCs	Nfe2l1
5.266E-250	0.73971375	0.737	0.295	1.306E-245	DCs	Pgam1
1.219E-249	0.53714927	0.466	0.147	3.022E-245	DCs	Chmp2b
1.413E-249	0.31813143	0.472	0.147	3.505E-245	DCs	Sh2b3
3.464E-249	0.52458812	0.63	0.222	8.588E-245	DCs	Tmco1
5.482E-249	0.40895618	0.514	0.17	1.359E-244	DCs	Arhgef2
9.844E-249	0.74500019	0.62	0.238	2.441E-244	DCs	Tent5a
1.011E-248	0.44429257	0.489	0.157	2.506E-244	DCs	Nipa2
1.051E-248	0.5423323	0.38	0.109	2.606E-244	DCs	Gtf2e2
2.012E-248	0.6714871	0.385	0.113	4.988E-244	DCs	Tuba1c
2.812E-248	0.54223554	0.603	0.212	6.973E-244	DCs	Sdhd
6.067E-248	0.64583908	0.369	0.105	1.504E-243	DCs	Rabl6
8.451E-248	0.60659261	0.652	0.239	2.095E-243	DCs	Ssbp4
3.058E-247	0.26615022	0.525	0.173	7.583E-243	DCs	Ap1s2
6.91E-247	0.72169832	0.662	0.265	1.713E-242	DCs	Nr4a1
9.915E-247	0.56332602	0.554	0.191	2.459E-242	DCs	Rps6ka1
1.271E-246	0.59521448	0.45	0.14	3.151E-242	DCs	Psmd3
1.32E-246	0.59850933	0.663	0.244	3.273E-242	DCs	Ndufs8
1.852E-246	0.63519776	0.748	0.296	4.591E-242	DCs	Vcp
1.908E-246	0.53745242	0.559	0.192	4.731E-242	DCs	Ptpra
3.358E-246	0.70895978	0.253	0.059	8.326E-242	DCs	Slc25a24
3.38E-246	0.57748357	0.363	0.103	8.381E-242	DCs	Pisd
4.162E-246	0.89900104	0.274	0.067	1.032E-241	DCs	Bicd2
8.222E-246	0.59884177	0.311	0.082	2.039E-241	DCs	Mcee
8.808E-246	0.56105559	0.689	0.254	2.184E-241	DCs	Rpn2

1.726E-245	0.73518241	0.83	0.362	4.28E-241	DCs	Ndufb8
3.885E-245	0.79907414	0.935	0.555	9.632E-241	DCs	Hsp90b1
3.929E-245	0.97580168	0.261	0.063	9.742E-241	DCs	Fasn
7.63E-245	0.74748993	0.362	0.104	1.892E-240	DCs	Blvra
1.168E-244	0.7327549	0.321	0.087	2.895E-240	DCs	Rbbp8
1.323E-244	0.62549557	0.394	0.117	3.28E-240	DCs	Mtln
1.881E-244	0.41667805	0.552	0.188	4.664E-240	DCs	Tmed7
1.371E-243	0.58958505	0.974	0.828	3.399E-239	DCs	Arpc1b
2.812E-243	0.76726738	0.3	0.078	6.972E-239	DCs	Nelfe
3.564E-243	0.54517704	0.324	0.087	8.836E-239	DCs	Stx12
3.697E-243	0.52758976	0.51	0.169	9.168E-239	DCs	Hcfc1r1
1.044E-242	0.65743435	0.326	0.089	2.588E-238	DCs	Mtx1
1.169E-242	0.7260746	0.328	0.09	2.897E-238	DCs	Dhrs1
2.032E-242	0.68986287	0.534	0.189	5.038E-238	DCs	Plp2
3.905E-242	0.40957315	0.39	0.115	9.682E-238	DCs	Pip5k1c
6.53E-242	0.72084993	0.26	0.062	1.619E-237	DCs	Copg2
8.742E-242	0.628543	0.289	0.074	2.168E-237	DCs	Gm37420
1.561E-241	0.76662011	0.315	0.085	3.871E-237	DCs	Bcap29
2.89E-241	0.49695918	0.393	0.117	7.166E-237	DCs	Sel1l
3.596E-241	0.55473739	0.525	0.178	8.917E-237	DCs	Ifnar2
7.794E-241	0.48619049	0.483	0.157	1.932E-236	DCs	Hras
7.908E-241	0.52622612	0.467	0.15	1.961E-236	DCs	Cdk2ap1
8.103E-241	0.48659126	0.608	0.218	2.009E-236	DCs	Dync1h1
1.008E-240	0.65474081	0.667	0.249	2.498E-236	DCs	Uqcrc1
1.812E-240	0.54643776	0.562	0.195	4.493E-236	DCs	Sumo3
4.084E-239	0.6151033	0.285	0.072	1.013E-234	DCs	Mdfic
6.646E-239	0.49960487	0.486	0.159	1.648E-234	DCs	Ap3b1
1.057E-238	0.50862037	0.379	0.112	2.62E-234	DCs	Isyna1
1.777E-238	0.5642256	0.607	0.218	4.406E-234	DCs	Mlf2
2.431E-238	0.63919366	0.383	0.115	6.027E-234	DCs	Cd180
7.447E-238	0.56911514	0.472	0.156	1.846E-233	DCs	Rps6ka3
1.199E-237	0.38930387	0.49	0.161	2.973E-233	DCs	Ppp1r12c
1.199E-237	0.51810095	0.357	0.102	2.974E-233	DCs	Tmem168
5.34E-237	0.55478435	0.656	0.248	1.324E-232	DCs	Uvrag
5.741E-237	0.62866207	0.38	0.113	1.424E-232	DCs	Emc8
1.172E-236	0.94927569	0.362	0.107	2.906E-232	DCs	Ncoa7
4.561E-236	0.73816597	0.855	0.396	1.131E-231	DCs	Pgls
6.234E-236	0.55362861	0.751	0.302	1.546E-231	DCs	Atp1a1
7.335E-236	0.41515152	0.462	0.149	1.819E-231	DCs	Hps3
2.769E-235	0.52763696	0.44	0.14	6.866E-231	DCs	Nfkb2
1.97E-234	0.65965327	0.402	0.123	4.885E-230	DCs	Lman1
3.958E-234	0.55419207	0.34	0.096	9.814E-230	DCs	Chst12
5.291E-234	0.5230461	0.614	0.224	1.312E-229	DCs	Rex1bd
1.45E-233	0.62924195	0.34	0.096	3.596E-229	DCs	Cltb
2.8E-233	0.60133701	0.372	0.11	6.942E-229	DCs	Vma21
8.753E-233	0.99613923	0.25	0.06	2.17E-228	DCs	Nbdy
1.1E-232	0.37294051	0.35	0.1	2.727E-228	DCs	Gsap
1.241E-232	0.46872732	0.519	0.176	3.076E-228	DCs	C1d
3.134E-232	0.49011336	0.434	0.138	7.77E-228	DCs	Coa5
3.364E-232	0.63596655	0.793	0.33	8.342E-228	DCs	Esd

6.113E-232	0.52046319	0.441	0.14	1.516E-227	DCs	Mrps25
6.608E-232	0.81929152	0.3	0.08	1.638E-227	DCs	Tmem242
1.029E-231	0.25834587	0.586	0.207	2.551E-227	DCs	Rin3
1.56E-231	1.41258354	0.488	0.189	3.869E-227	DCs	Capg
1.8E-231	0.4539406	0.512	0.174	4.464E-227	DCs	Ilk
1.874E-231	0.45919975	0.671	0.255	4.646E-227	DCs	Asap1
2.509E-231	0.7861705	0.297	0.079	6.221E-227	DCs	Rtca
2.861E-231	0.54850759	0.31	0.084	7.093E-227	DCs	Agpat5
4.592E-231	0.53222455	0.711	0.28	1.139E-226	DCs	Sppl2a
5.346E-231	0.48902662	0.309	0.084	1.326E-226	DCs	Ckap4
7.679E-231	0.95508178	0.258	0.064	1.904E-226	DCs	Zfp422
2.285E-230	0.57262005	0.604	0.221	5.666E-226	DCs	0610012G03R
4.957E-230	0.34459909	0.488	0.162	1.229E-225	DCs	Ap3s1
5.266E-230	0.33339943	0.503	0.17	1.306E-225	DCs	Adam17
7.445E-230	0.56117546	0.53	0.184	1.846E-225	DCs	Snrpb2
1.322E-229	0.46147757	0.589	0.21	3.278E-225	DCs	Ndufb6
1.655E-229	0.79421766	0.293	0.078	4.104E-225	DCs	Slc35b2
1.759E-229	0.47192569	0.56	0.195	4.362E-225	DCs	Psmd6
1.885E-229	0.53431486	0.508	0.172	4.674E-225	DCs	Ndufa9
1.936E-229	0.6092446	0.439	0.143	4.799E-225	DCs	Got2
4.212E-229	0.60726044	0.934	0.566	1.044E-224	DCs	Zfp36
5.093E-229	0.48105635	0.536	0.185	1.263E-224	DCs	Mrpl36
6.1E-229	0.39661439	0.858	0.354	1.512E-224	DCs	Lyn
1.087E-228	0.41022601	0.683	0.26	2.694E-224	DCs	Rgs10
4.464E-228	0.5305212	0.435	0.139	1.107E-223	DCs	Parl
4.469E-228	0.25357151	0.48	0.158	1.108E-223	DCs	Hsd17b11
4.654E-228	0.46439757	0.545	0.192	1.154E-223	DCs	2900097C17R
8.48E-228	0.52580507	0.361	0.106	2.103E-223	DCs	Tpgs1
1.126E-227	0.44480816	0.255	0.063	2.791E-223	DCs	Zdhhc9
1.211E-227	0.66895809	0.809	0.358	3.003E-223	DCs	BC005537
1.375E-227	0.57566736	0.346	0.099	3.409E-223	DCs	Ero1lb
3.212E-227	0.45222298	0.579	0.208	7.963E-223	DCs	Mrpl14
4.155E-227	0.58410597	0.355	0.105	1.03E-222	DCs	Clptm1
4.883E-227	0.42228962	0.521	0.178	1.211E-222	DCs	Api5
1.583E-226	0.51681104	0.461	0.152	3.925E-222	DCs	Stt3a
3.333E-226	0.64933638	0.753	0.317	8.264E-222	DCs	Lamtor4
4.856E-226	0.6031157	0.351	0.102	1.204E-221	DCs	Tusc3
7.686E-226	0.38980387	0.498	0.167	1.906E-221	DCs	Rad23b
1.03E-225	0.51041817	0.343	0.099	2.555E-221	DCs	Mfsd10
1.414E-225	0.56281207	0.421	0.134	3.506E-221	DCs	Psmd1
1.386E-224	0.65948046	0.391	0.122	3.436E-220	DCs	Lpp
1.396E-224	0.64428452	0.325	0.092	3.461E-220	DCs	Smim10l1
1.724E-224	0.49144598	0.434	0.139	4.274E-220	DCs	Tsg101
1.757E-224	0.6552368	0.332	0.095	4.358E-220	DCs	Pih1d1
2.379E-224	0.32845795	0.514	0.178	5.898E-220	DCs	Cpne3
3.178E-224	0.53320113	0.428	0.137	7.879E-220	DCs	Hmg20b
1.673E-223	0.78369973	0.284	0.075	4.149E-219	DCs	Mrps6
1.85E-223	0.56547428	0.462	0.153	4.588E-219	DCs	Etfa
5.211E-223	0.43991512	0.617	0.225	1.292E-218	DCs	Ebp
9.48E-223	0.60541546	0.258	0.065	2.35E-218	DCs	Klhdc3
