# Supplementary material for: Neutrophil-enriched gene signature correlates with teplizumab therapy resistance in different stages of type 1 diabetes
Source: J Clin Invest. 2025 Sep 30;135(23):e176403. doi: 10.1172/JCI176403 (PMC12646666; doi:10.1172/JCI176403)
Supplement: Supplemental table 3 [file jci-135-176403-s291.pdf]

Supplemental Table 3: All data related to the gene signatures identified in the NOD mouse model.

p_val	avg_log2FC	pct.1	pct.2	p_val_adj
2.0996E-249	0.430676185	0.617	0.456	5.2058E-245
4.8525E-144	-0.45945439	0.96	0.967	1.2032E-139
3.7935E-125	0.324029578	0.559	0.465	9.4059E-121
4.41482E-80	-0.297116601	0.696	0.723	1.09466E-75
1.68425E-77	0.212122685	0.852	0.836	4.1761E-73
2.50582E-76	0.365430778	0.35	0.261	6.21319E-72
2.57881E-74	0.254993988	0.285	0.207	6.39416E-70
1.9298E-73	-0.269267263	0.712	0.735	4.78494E-69
1.72334E-72	0.176299672	0.848	0.826	4.27303E-68
1.05754E-71	0.108952118	0.99	0.99	2.62217E-67
2.63943E-71	0.208125176	0.672	0.588	6.54445E-67
9.32734E-67	0.222527536	0.738	0.701	2.31271E-62
6.87374E-60	0.577362511	0.181	0.126	1.70434E-55
8.47955E-60	0.123885146	0.983	0.979	2.10251E-55
1.44814E-59	0.176746339	0.918	0.905	3.59067E-55
5.73889E-59	-0.349512996	0.302	0.36	1.42296E-54
4.13151E-55	0.130158264	0.949	0.936	1.02441E-50
4.08893E-54	0.162207168	0.854	0.829	1.01385E-49
5.09612E-54	-0.232609308	0.718	0.737	1.26358E-49
1.28134E-53	0.193241048	0.742	0.719	3.17707E-49
2.68968E-53	-0.176275516	0.919	0.931	6.66907E-49
5.59227E-51	-0.217780202	0.751	0.77	1.3866E-46
2.21139E-47	-0.203340085	0.413	0.476	5.48314E-43
2.95602E-47	0.158279988	0.797	0.767	7.32945E-43
4.21312E-47	0.274554734	0.749	0.669	1.04464E-42
6.14768E-46	0.135855353	0.844	0.812	1.52432E-41
2.71319E-45	-0.201837266	0.141	0.19	6.72734E-41
9.33308E-44	0.181463691	0.759	0.728	2.31414E-39
6.76542E-43	0.143908692	0.299	0.238	1.67749E-38
1.52595E-42	0.364711017	0.113	0.074	3.7836E-38
4.71013E-42	-0.32595387	0.564	0.617	1.16788E-37
1.13791E-41	0.141088694	0.885	0.864	2.82146E-37
6.47966E-41	-0.207142819	0.579	0.615	1.60663E-36
1.33413E-40	0.125397124	0.948	0.934	3.30798E-36
3.05871E-40	0.190686757	0.665	0.633	7.58408E-36
1.63069E-39	0.135364233	0.92	0.905	4.0433E-35
7.44441E-37	-0.171904704	0.566	0.606	1.84584E-32
1.94366E-36	0.301041163	0.265	0.216	4.8193E-32
2.48109E-36	-0.191377376	0.452	0.492	6.15187E-32
3.5093E-36	-0.224634748	0.148	0.192	8.7013E-32
1.01693E-35	0.144893585	0.761	0.734	2.52147E-31
1.30644E-35	0.15573835	0.423	0.363	3.23931E-31
8.74217E-35	0.196726913	0.457	0.406	2.16762E-30
1.45601E-34	0.11533704	0.968	0.962	3.61018E-30
2.38082E-34	-0.142517054	0.962	0.968	5.90325E-30
3.21865E-33	0.193839058	0.428	0.374	7.98064E-29
1.36638E-32	0.179752552	0.552	0.504	3.38795E-28
2.06416E-32	-0.236501221	0.401	0.442	5.11809E-28

2.87473E-32	0.138235214	0.859	0.842	7.1279E-28
6.55972E-32	0.198383771	0.253	0.206	1.62648E-27
1.87797E-30	0.183881529	0.448	0.389	4.65642E-26
2.04741E-30	0.183699793	0.215	0.172	5.07654E-26
1.37702E-29	0.448436679	0.236	0.193	3.41432E-25
1.18275E-28	0.078521306	0.989	0.988	2.93264E-24
4.26686E-28	0.138339029	0.531	0.478	1.05797E-23
4.35343E-28	-0.300894359	0.217	0.254	1.07943E-23
4.75222E-28	0.179503136	0.685	0.632	1.17831E-23
1.32538E-27	0.133267098	0.605	0.551	3.28629E-23
2.48259E-27	0.171541142	0.303	0.256	6.15558E-23
2.4987E-27	0.149658034	0.627	0.563	6.19552E-23
1.05902E-26	0.119959121	0.701	0.667	2.62585E-22
1.37561E-26	-0.131886231	0.904	0.915	3.41082E-22
2.60925E-26	0.142899711	0.822	0.794	6.46963E-22
5.03032E-26	0.107955228	0.795	0.777	1.24727E-21
6.45517E-26	-0.208962927	0.846	0.848	1.60056E-21
1.46929E-25	0.141689299	0.644	0.609	3.64311E-21
2.33305E-25	-0.141656025	0.543	0.582	5.78479E-21
5.57532E-25	-0.153686667	0.429	0.465	1.3824E-20
5.8767E-25	0.114569643	0.87	0.851	1.45713E-20
6.27976E-25	0.141640133	0.626	0.564	1.55707E-20
6.82324E-25	0.104167191	0.85	0.838	1.69182E-20
1.02649E-24	0.107092217	0.593	0.541	2.54517E-20
1.15675E-24	0.156406389	0.606	0.565	2.86815E-20
2.36736E-24	-0.174118897	0.599	0.624	5.86987E-20
2.70298E-24	0.065014419	0.25	0.204	6.70204E-20
3.53724E-24	0.11386137	0.216	0.176	8.77059E-20
3.83657E-24	-0.048048505	0.992	0.995	9.51278E-20
4.54136E-24	0.117555314	0.818	0.788	1.12603E-19
5.05681E-24	-0.155140125	0.18	0.216	1.25384E-19
8.53362E-24	0.072885525	0.996	0.994	2.11591E-19
1.1585E-23	-0.078669434	0.505	0.546	2.8725E-19
1.36658E-23	0.2036579	0.208	0.172	3.38843E-19
1.58302E-23	0.113031415	0.803	0.755	3.92509E-19
1.64017E-23	0.117359393	0.367	0.318	4.06679E-19
1.67251E-23	-0.124651432	0.622	0.647	4.14698E-19
1.99507E-23	0.119513253	0.723	0.7	4.94678E-19
2.99101E-23	0.142413977	0.571	0.532	7.41621E-19
4.1378E-23	-0.36439657	0.119	0.151	1.02597E-18
5.67182E-23	-0.259882568	0.318	0.353	1.40633E-18
6.43456E-23	0.097297291	0.109	0.081	1.59545E-18
1.17186E-22	-0.434409858	0.303	0.335	2.90563E-18
1.26638E-22	0.113538498	0.652	0.614	3.13998E-18
1.72222E-22	0.108679812	0.86	0.848	4.27024E-18
3.0554E-22	0.091021863	0.121	0.092	7.57586E-18
3.37388E-22	-0.156641841	0.133	0.164	8.36553E-18
5.90407E-22	-0.185106027	0.489	0.514	1.46391E-17
1.05888E-21	-0.32416147	0.59	0.615	2.6255E-17
1.39084E-21	0.143426769	0.306	0.266	3.44858E-17

2.00259E-21	-0.163434174	0.462	0.491	4.96543E-17
2.17818E-21	0.120455111	0.615	0.572	5.4008E-17
2.19421E-21	-0.198351064	0.251	0.284	5.44055E-17
2.39166E-21	0.117627737	0.62	0.586	5.93012E-17
4.26841E-21	0.241748793	0.183	0.151	1.05835E-16
4.48253E-21	0.077785166	0.98	0.98	1.11144E-16
5.30198E-21	-0.320893557	0.543	0.57	1.31463E-16
6.60878E-21	-0.114404981	0.705	0.727	1.63865E-16
7.51951E-21	-0.129364866	0.138	0.169	1.86446E-16
8.30743E-21	0.076102675	0.679	0.632	2.05983E-16
1.00686E-20	0.103849497	0.222	0.184	2.4965E-16
1.123E-20	0.129361933	0.496	0.452	2.78448E-16
1.14892E-20	-0.142256386	0.148	0.18	2.84875E-16
1.30645E-20	0.089842297	0.821	0.802	3.23935E-16
1.4719E-20	-0.055376974	0.995	0.994	3.64959E-16
1.57054E-20	0.170217066	0.668	0.638	3.89414E-16
1.8804E-20	-0.290315403	0.266	0.299	4.66246E-16
2.27925E-20	0.086212861	0.192	0.157	5.65141E-16
2.54249E-20	0.155250556	0.237	0.199	6.3041E-16
3.16766E-20	-0.078528668	0.924	0.938	7.85422E-16
4.61524E-20	-0.20601212	0.138	0.167	1.14435E-15
5.59888E-20	-0.183532781	0.324	0.357	1.38824E-15
6.25614E-20	0.153486575	0.181	0.149	1.55121E-15
6.74733E-20	0.104264594	0.116	0.089	1.673E-15
9.95333E-20	-0.312308086	0.315	0.345	2.46793E-15
1.15362E-19	0.131178698	0.479	0.429	2.8604E-15
1.28485E-19	0.196188498	0.344	0.305	3.18579E-15
1.33474E-19	0.099403419	0.24	0.203	3.30948E-15
2.66289E-19	0.0611469	0.148	0.118	6.60263E-15
3.27524E-19	0.096233748	0.343	0.299	8.12096E-15
4.1551E-19	-0.265154174	0.124	0.152	1.03026E-14
4.52847E-19	0.166021009	0.34	0.302	1.12283E-14
7.65207E-19	0.065726819	0.22	0.184	1.89733E-14
8.75231E-19	-0.448181428	0.146	0.176	2.17014E-14
9.28348E-19	0.149332134	0.326	0.288	2.30184E-14
1.02673E-18	0.068962138	0.117	0.091	2.54577E-14
1.16799E-18	0.092450759	0.232	0.196	2.89604E-14
1.26712E-18	0.107291367	0.482	0.436	3.14182E-14
1.33849E-18	-0.086474885	0.637	0.665	3.31879E-14
1.64646E-18	0.099598873	0.714	0.684	4.0824E-14
1.74216E-18	-0.134416949	0.098	0.123	4.31969E-14
1.86596E-18	0.052433893	0.107	0.082	4.62664E-14
1.92972E-18	-0.141430411	0.195	0.226	4.78474E-14
2.05875E-18	0.129382188	0.162	0.132	5.10467E-14
2.38871E-18	0.126882122	0.254	0.216	5.92281E-14
2.45811E-18	0.148915155	0.368	0.33	6.09489E-14
2.49098E-18	-0.129773169	0.421	0.453	6.17637E-14
2.57655E-18	0.300373602	0.835	0.823	6.38856E-14
2.84831E-18	0.1069444	0.256	0.219	7.06237E-14
2.94912E-18	0.110881128	0.118	0.092	7.31233E-14

3.29287E-18	-0.089707714	0.765	0.78	8.16468E-14
3.56326E-18	0.100451623	0.545	0.501	8.8351E-14
4.72596E-18	0.088931429	0.125	0.098	1.1718E-13
4.78821E-18	-0.166230912	0.169	0.198	1.18724E-13
4.86613E-18	-0.289747449	0.268	0.298	1.20656E-13
5.73057E-18	0.116404509	0.473	0.433	1.4209E-13
7.2195E-18	0.085440376	0.503	0.45	1.79007E-13
7.67507E-18	-0.146212578	0.374	0.407	1.90303E-13
7.77876E-18	0.09026217	0.64	0.601	1.92874E-13
8.26021E-18	-0.283569666	0.332	0.358	2.04812E-13
9.31513E-18	0.084886067	0.8	0.773	2.30969E-13
1.15082E-17	0.181409948	0.303	0.265	2.85346E-13
1.53466E-17	-0.039271309	0.157	0.128	3.8052E-13
1.55202E-17	0.087285952	0.322	0.28	3.84823E-13
1.60705E-17	-0.143277328	0.135	0.163	3.98469E-13
2.30584E-17	0.077233755	0.213	0.179	5.71733E-13
2.88491E-17	0.111889528	0.147	0.12	7.15312E-13
3.35982E-17	0.13812933	0.174	0.144	8.33068E-13
3.40287E-17	0.099093216	0.741	0.716	8.43743E-13
3.82411E-17	0.124515421	0.48	0.443	9.48189E-13
4.10663E-17	0.192863754	0.381	0.345	1.01824E-12
4.35563E-17	0.084443943	0.22	0.187	1.07998E-12
4.41575E-17	0.086643366	0.171	0.14	1.09489E-12
4.68458E-17	0.058116118	0.142	0.114	1.16154E-12
5.11654E-17	0.087918103	0.106	0.083	1.26865E-12
7.81284E-17	0.072848189	0.315	0.274	1.93719E-12
8.40751E-17	0.050191902	0.103	0.079	2.08464E-12
8.84949E-17	0.075496508	0.132	0.105	2.19423E-12
9.92076E-17	0.10306212	0.397	0.353	2.45985E-12
1.02808E-16	0.09817821	0.209	0.176	2.54913E-12
1.34541E-16	-0.11682529	0.367	0.397	3.33595E-12
1.41803E-16	0.101935502	0.364	0.323	3.51601E-12
1.55252E-16	0.200236529	0.325	0.29	3.84946E-12
1.78623E-16	0.118852919	0.519	0.478	4.42895E-12
1.95755E-16	-0.126816776	0.551	0.577	4.85374E-12
2.08549E-16	-0.140025233	0.243	0.274	5.17096E-12
2.21319E-16	0.205814249	0.388	0.356	5.4876E-12
2.26153E-16	-0.200518727	0.335	0.366	5.60746E-12
2.36105E-16	0.079801239	0.211	0.178	5.85422E-12
3.13046E-16	-0.105384467	0.482	0.508	7.76197E-12
3.30984E-16	-0.200963333	0.149	0.176	8.20675E-12
3.38132E-16	0.090503779	0.44	0.398	8.38398E-12
3.83605E-16	0.144153968	0.203	0.172	9.51148E-12
5.00505E-16	0.076303936	0.563	0.511	1.241E-11
5.39814E-16	-0.185511115	0.204	0.232	1.33847E-11
5.80491E-16	0.060832716	0.16	0.131	1.43933E-11
8.19955E-16	-0.116568611	0.432	0.459	2.03308E-11
8.42968E-16	-0.122940251	0.115	0.139	2.09014E-11
9.36473E-16	0.081903548	0.739	0.719	2.32198E-11
9.75294E-16	0.082765775	0.862	0.845	2.41824E-11

1.05404E-15	0.039855555	0.156	0.127	2.6135E-11
1.14451E-15	-0.105924175	0.682	0.708	2.83781E-11
1.32512E-15	0.079195088	0.783	0.763	3.28563E-11
1.42316E-15	0.11335221	0.662	0.62	3.52871E-11
1.61075E-15	0.102756627	0.106	0.083	3.99387E-11
1.92428E-15	0.080698256	0.775	0.746	4.77126E-11
1.93333E-15	0.0907046	0.455	0.414	4.79368E-11
2.0494E-15	0.17622553	0.281	0.25	5.0815E-11
2.14121E-15	0.079733433	0.833	0.811	5.30914E-11
2.21497E-15	0.0640068	0.194	0.163	5.49201E-11
2.84316E-15	-0.102494677	0.153	0.181	7.04962E-11
3.09136E-15	-0.11100431	0.431	0.462	7.66503E-11
3.12639E-15	0.078126543	0.219	0.187	7.75188E-11
3.60277E-15	0.086343558	0.917	0.903	8.93306E-11
3.906E-15	0.106662552	0.179	0.151	9.68493E-11
4.15968E-15	0.095441032	0.836	0.821	1.03139E-10
4.50467E-15	-0.13776842	0.75	0.693	1.11693E-10
4.61939E-15	-0.178199292	0.196	0.223	1.14538E-10
4.72172E-15	-0.159154858	0.231	0.26	1.17075E-10
4.90479E-15	0.096774038	0.259	0.226	1.21614E-10
5.26489E-15	0.145675084	0.598	0.565	1.30543E-10
5.49118E-15	0.084267966	0.47	0.432	1.36154E-10
5.49498E-15	-0.164990452	0.234	0.263	1.36248E-10
6.76348E-15	0.145907784	0.396	0.357	1.677E-10
7.25988E-15	0.104551381	0.939	0.925	1.80009E-10
8.4489E-15	0.067333396	0.195	0.164	2.0949E-10
8.66542E-15	0.063027352	0.102	0.081	2.14859E-10
1.0466E-14	0.094104239	0.219	0.188	2.59505E-10
1.33564E-14	-0.138629348	0.358	0.389	3.31171E-10
1.35277E-14	0.05203131	0.113	0.09	3.35419E-10
1.43576E-14	0.126904366	0.588	0.56	3.55998E-10
1.49143E-14	0.072045532	0.292	0.254	3.698E-10
1.49181E-14	0.118090031	0.861	0.819	3.69894E-10
1.6484E-14	0.085931876	0.695	0.666	4.08721E-10
1.78891E-14	0.076080061	0.321	0.283	4.4356E-10
2.13211E-14	0.073495416	0.117	0.095	5.28656E-10
2.19543E-14	0.103602788	0.244	0.213	5.44356E-10
2.50338E-14	0.061034305	0.885	0.866	6.20714E-10
2.56239E-14	0.092498324	0.839	0.823	6.35344E-10
2.61148E-14	0.066372794	0.215	0.184	6.47517E-10
2.80678E-14	0.079636834	0.152	0.126	6.95942E-10
2.83927E-14	0.071647383	0.427	0.386	7.03997E-10
3.67191E-14	-0.120067551	0.606	0.616	9.1045E-10
4.09082E-14	0.08450833	0.131	0.107	1.01432E-09
5.54766E-14	0.081274557	0.862	0.847	1.37554E-09
5.9018E-14	0.083529784	0.344	0.305	1.46335E-09
6.33784E-14	0.087092884	0.406	0.367	1.57147E-09
6.7172E-14	0.081054371	0.231	0.199	1.66553E-09
7.31386E-14	-0.127283851	0.183	0.207	1.81347E-09
7.41282E-14	0.194018452	0.276	0.246	1.83801E-09

7.51611E-14	0.069280721	0.934	0.927	1.86362E-09
7.73E-14	0.081618453	0.101	0.08	1.91665E-09
8.44165E-14	0.088377043	0.658	0.632	2.09311E-09
8.90927E-14	0.063999275	0.318	0.28	2.20905E-09
8.92274E-14	0.044471677	0.149	0.123	2.21239E-09
9.23659E-14	0.086967958	0.218	0.189	2.29021E-09
9.53546E-14	0.173503668	0.295	0.259	2.36432E-09
9.55588E-14	-0.300057737	0.12	0.144	2.36938E-09
1.01113E-13	0.07761205	0.545	0.494	2.5071E-09
1.12049E-13	0.215392697	0.256	0.225	2.77825E-09
1.15165E-13	0.087260997	0.441	0.401	2.85551E-09
1.37106E-13	-0.015974071	0.167	0.14	3.39953E-09
1.40421E-13	0.121613136	0.158	0.133	3.48173E-09
1.42208E-13	0.065570527	0.134	0.11	3.52604E-09
1.4454E-13	0.103872643	0.467	0.431	3.58386E-09
1.69877E-13	-0.149345758	0.111	0.133	4.21211E-09
1.7299E-13	0.153022811	0.28	0.248	4.28928E-09
1.83313E-13	0.060164821	0.195	0.166	4.54526E-09
1.85409E-13	-0.144356521	0.14	0.164	4.59721E-09
1.85734E-13	0.137578106	0.619	0.59	4.60528E-09
1.90497E-13	-0.099155409	0.174	0.2	4.72338E-09
2.2652E-13	0.081349925	0.85	0.837	5.61656E-09
2.55042E-13	0.089811082	0.605	0.565	6.32377E-09
2.73031E-13	0.07406859	0.721	0.695	6.76981E-09
2.8748E-13	-0.123349987	0.669	0.689	7.12806E-09
3.24381E-13	0.070327902	0.104	0.083	8.04304E-09
3.27427E-13	0.115648516	0.143	0.119	8.11856E-09
4.41563E-13	-0.112484731	0.256	0.284	1.09485E-08
4.47518E-13	0.060302794	0.264	0.231	1.10962E-08
4.84248E-13	0.05232481	0.191	0.163	1.20069E-08
5.06371E-13	0.10405666	0.147	0.124	1.25555E-08
5.97575E-13	-0.095976227	0.481	0.503	1.48169E-08
5.98115E-13	0.057165289	0.118	0.096	1.48303E-08
6.17561E-13	-0.133592541	0.319	0.344	1.53124E-08
6.3404E-13	0.060195968	0.171	0.144	1.5721E-08
7.24475E-13	-0.189122894	0.257	0.286	1.79633E-08
8.0136E-13	0.065794916	0.103	0.083	1.98697E-08
8.77138E-13	0.086493518	0.303	0.269	2.17486E-08
9.52214E-13	0.134806437	0.122	0.101	2.36101E-08
9.82479E-13	0.048031652	0.238	0.207	2.43606E-08
1.05377E-12	0.074703598	0.407	0.372	2.61281E-08
1.05736E-12	0.037663473	0.185	0.157	2.62173E-08
1.12551E-12	0.076927816	0.363	0.328	2.7907E-08
1.13208E-12	0.072429635	0.301	0.267	2.80699E-08
1.14936E-12	0.135355691	0.235	0.207	2.84985E-08
1.16871E-12	0.080591034	0.597	0.557	2.89782E-08
1.35538E-12	0.067883017	0.379	0.344	3.36067E-08
1.47187E-12	0.120225536	0.226	0.197	3.64951E-08
1.61579E-12	0.078568481	0.21	0.183	4.00634E-08
1.97608E-12	0.070699276	0.645	0.616	4.89969E-08

2.1156E-12	-0.130907749	0.167	0.189	5.24564E-08
2.19376E-12	-0.099303868	0.296	0.32	5.43942E-08
2.21934E-12	-0.102655802	0.272	0.296	5.50286E-08
2.28325E-12	0.07319323	0.696	0.671	5.66131E-08
2.55035E-12	0.089692044	0.558	0.525	6.32358E-08
2.90672E-12	0.063633001	0.388	0.351	7.20722E-08
2.92395E-12	0.097165161	0.39	0.358	7.24994E-08
3.08444E-12	0.082452062	0.555	0.522	7.64786E-08
3.21216E-12	-0.122276438	0.49	0.507	7.96455E-08
3.32703E-12	0.072253372	0.886	0.872	8.24936E-08
3.55265E-12	0.066463233	0.944	0.937	8.80879E-08
4.53401E-12	-0.067506693	0.218	0.245	1.12421E-07
4.54318E-12	0.065517975	0.384	0.346	1.12648E-07
4.54935E-12	0.092943218	0.168	0.143	1.12801E-07
4.57641E-12	0.081514262	0.184	0.158	1.13472E-07
4.58854E-12	0.075080929	0.124	0.103	1.13773E-07
5.02281E-12	0.091223825	0.542	0.512	1.24541E-07
5.56351E-12	0.064585437	0.123	0.102	1.37947E-07
5.87392E-12	0.14637823	0.345	0.312	1.45644E-07
5.88096E-12	-0.098493314	0.375	0.397	1.45818E-07
5.94723E-12	0.064145032	0.133	0.111	1.47461E-07
6.11515E-12	0.090512819	0.24	0.211	1.51625E-07
6.12589E-12	0.04312318	0.128	0.106	1.51891E-07
6.14284E-12	0.012537145	0.643	0.611	1.52312E-07
7.12002E-12	0.055470266	0.13	0.108	1.76541E-07
8.02674E-12	0.055895059	0.311	0.277	1.99023E-07
8.99728E-12	0.074042418	0.419	0.383	2.23088E-07
1.0348E-11	0.123749457	0.437	0.409	2.56579E-07
1.04793E-11	-0.118672288	0.192	0.215	2.59833E-07
1.05585E-11	-0.130933496	0.384	0.408	2.61797E-07
1.08211E-11	0.058349268	0.287	0.254	2.68309E-07
1.23505E-11	0.074830179	0.248	0.219	3.06231E-07
1.2446E-11	0.057703519	0.119	0.099	3.08599E-07
1.24585E-11	-0.115135783	0.384	0.405	3.08908E-07
1.45603E-11	0.055875196	0.266	0.234	3.61023E-07
1.65334E-11	0.052581206	0.12	0.099	4.09946E-07
1.88072E-11	0.045932963	0.211	0.182	4.66326E-07
2.01833E-11	-0.151620429	0.165	0.186	5.00445E-07
2.07683E-11	0.077098344	0.642	0.61	5.14949E-07
2.10421E-11	0.108762919	0.982	0.982	5.21738E-07
2.23166E-11	0.080983422	0.619	0.585	5.5334E-07
2.36049E-11	0.135169738	0.183	0.158	5.85283E-07
2.57922E-11	0.083613027	0.76	0.749	6.39519E-07
2.58621E-11	0.155066502	0.317	0.291	6.41251E-07
2.62809E-11	0.070203205	0.237	0.208	6.51634E-07
2.63687E-11	-0.089776427	0.241	0.267	6.53811E-07
2.69202E-11	0.03946821	0.214	0.186	6.67487E-07
2.71325E-11	-0.083745894	0.634	0.648	6.7275E-07
2.7317E-11	-0.211261493	0.591	0.605	6.77324E-07
3.36834E-11	0.198838986	0.257	0.232	8.35179E-07

3.46679E-11	0.043181672	0.207	0.18	8.59591E-07
3.52024E-11	0.060829889	0.253	0.223	8.72842E-07
3.5576E-11	0.098177283	0.655	0.626	8.82108E-07
3.69076E-11	0.055708435	0.102	0.083	9.15124E-07
3.69937E-11	0.054743206	0.808	0.792	9.17258E-07
3.86552E-11	0.057589115	0.181	0.155	9.58456E-07
3.88777E-11	0.133015809	0.203	0.179	9.63971E-07
3.94982E-11	-0.116842461	0.335	0.355	9.79358E-07
4.13826E-11	0.039284137	0.187	0.16	1.02608E-06
4.20558E-11	-0.129597345	0.258	0.282	1.04277E-06
4.39726E-11	0.024665826	0.173	0.148	1.0903E-06
4.59423E-11	0.088226356	0.11	0.091	1.13914E-06
4.78352E-11	0.054333731	0.241	0.211	1.18607E-06
5.05009E-11	0.074426552	0.648	0.621	1.25217E-06
5.35267E-11	0.083262612	0.348	0.314	1.32719E-06
5.51477E-11	0.070550007	0.625	0.591	1.36739E-06
5.70989E-11	-0.07116586	0.083	0.101	1.41577E-06
5.77107E-11	0.079752291	0.144	0.122	1.43094E-06
5.77666E-11	-0.116852417	0.267	0.289	1.43232E-06
6.18087E-11	0.075437005	0.357	0.327	1.53255E-06
6.51762E-11	0.100598562	0.121	0.101	1.61604E-06
7.68705E-11	-0.119663192	0.22	0.241	1.906E-06
8.08226E-11	0.055716456	0.289	0.258	2.004E-06
8.10281E-11	0.080803266	0.828	0.817	2.00909E-06
8.12452E-11	0.069365198	0.312	0.279	2.01448E-06
8.13422E-11	0.069206728	0.238	0.21	2.01688E-06
8.15639E-11	0.061089145	0.958	0.954	2.02238E-06
8.33497E-11	0.088716623	0.602	0.56	2.06666E-06
8.79505E-11	0.068686227	0.523	0.489	2.18073E-06
9.00117E-11	-0.115041483	0.39	0.413	2.23184E-06
9.02552E-11	0.066366454	0.365	0.332	2.23788E-06
9.13801E-11	0.049395601	0.345	0.31	2.26577E-06
9.21222E-11	0.072585661	0.822	0.812	2.28417E-06
9.39843E-11	0.066113771	0.931	0.923	2.33034E-06
1.01839E-10	0.067130968	0.561	0.523	2.52509E-06
1.03366E-10	0.037473436	0.183	0.157	2.56295E-06
1.09595E-10	0.038444226	0.128	0.107	2.71741E-06
1.19365E-10	0.065264326	0.308	0.275	2.95965E-06
1.3225E-10	-0.130125963	0.292	0.314	3.27913E-06
1.35986E-10	0.066537399	0.333	0.3	3.37176E-06
1.40064E-10	0.068055299	0.643	0.615	3.4729E-06
1.50769E-10	0.056527834	0.166	0.142	3.73832E-06
1.52823E-10	0.054663417	0.226	0.199	3.78924E-06
1.67149E-10	0.074723964	0.268	0.238	4.14447E-06
1.67212E-10	0.0726787	0.581	0.547	4.14603E-06
1.76733E-10	0.048967557	0.211	0.184	4.38209E-06
1.80319E-10	0.081247192	0.677	0.644	4.471E-06
1.82184E-10	0.085060225	0.514	0.479	4.51725E-06
1.88929E-10	0.063956343	0.338	0.303	4.68449E-06
1.97711E-10	0.041960277	0.26	0.23	4.90223E-06

1.97874E-10	0.086141379	0.183	0.158	4.90629E-06
1.99758E-10	0.076024832	0.115	0.096	4.953E-06
2.04542E-10	-0.342092677	0.115	0.134	5.07163E-06
2.07564E-10	0.139802679	0.237	0.212	5.14655E-06
2.13181E-10	0.039103233	0.143	0.121	5.28583E-06
2.20524E-10	0.092158362	0.487	0.454	5.46789E-06
2.22211E-10	0.054093654	0.581	0.545	5.50972E-06
2.32043E-10	0.075511533	0.494	0.458	5.7535E-06
2.44392E-10	0.071423598	0.347	0.314	6.05971E-06
2.44997E-10	0.055002893	0.32	0.288	6.07471E-06
2.47153E-10	0.108517673	0.646	0.602	6.12816E-06
2.54854E-10	0.067801301	0.901	0.892	6.31911E-06
2.81017E-10	0.028813642	0.123	0.103	6.96782E-06
2.84057E-10	0.067519622	0.582	0.549	7.04319E-06
2.89645E-10	-0.07801713	0.197	0.22	7.18176E-06
2.93243E-10	0.034686218	0.238	0.209	7.27097E-06
3.14198E-10	0.054966836	0.331	0.299	7.79055E-06
3.15761E-10	0.075798839	0.564	0.531	7.8293E-06
3.19851E-10	0.056441088	0.305	0.274	7.93071E-06
3.31594E-10	0.067669106	0.324	0.292	8.22187E-06
3.44571E-10	0.062090967	0.23	0.203	8.54363E-06
3.89021E-10	0.066451193	0.35	0.32	9.64577E-06
3.95637E-10	0.06240188	0.143	0.122	9.80981E-06
4.40962E-10	0.050294213	0.362	0.329	1.09337E-05
4.69376E-10	0.051067489	0.151	0.129	1.16382E-05
4.76396E-10	-0.1015825	0.261	0.286	1.18122E-05
4.86528E-10	0.046629077	0.163	0.141	1.20635E-05
4.89331E-10	0.053380658	0.284	0.253	1.2133E-05
4.94011E-10	0.039510444	0.126	0.106	1.2249E-05
4.97191E-10	0.065587223	0.156	0.134	1.23279E-05
5.09323E-10	0.05779878	0.538	0.5	1.26287E-05
5.1278E-10	0.081346469	0.221	0.196	1.27144E-05
5.37054E-10	-0.168533264	0.312	0.328	1.33162E-05
5.41004E-10	0.033602818	0.101	0.083	1.34142E-05
5.55969E-10	0.054851065	0.12	0.101	1.37853E-05
5.69678E-10	-0.132088077	0.176	0.196	1.41252E-05
5.765E-10	-0.076795253	0.231	0.255	1.42943E-05
5.91656E-10	-0.087974571	0.303	0.324	1.46701E-05
6.35407E-10	0.066652858	0.223	0.197	1.57549E-05
6.87183E-10	0.087118872	0.822	0.801	1.70387E-05
6.9805E-10	0.031894887	0.347	0.311	1.73081E-05
7.20845E-10	0.061798377	0.355	0.322	1.78733E-05
7.21714E-10	0.027781895	0.187	0.163	1.78949E-05
7.90457E-10	0.069412752	0.173	0.15	1.95994E-05
8.0916E-10	0.076128426	0.755	0.731	2.00631E-05
8.76173E-10	0.094706718	0.559	0.523	2.17247E-05
8.97207E-10	0.045622046	0.335	0.301	2.22462E-05
9.42087E-10	0.047838392	0.308	0.276	2.3359E-05
1.02091E-09	0.08129033	0.117	0.099	2.53134E-05
1.02187E-09	0.044302521	0.215	0.189	2.53372E-05

1.0585E-09	0.036218306	0.237	0.209	2.62456E-05
1.10869E-09	-0.136169198	0.176	0.196	2.749E-05
1.14048E-09	0.042720992	0.243	0.215	2.82781E-05
1.19561E-09	-0.066085988	0.598	0.613	2.96452E-05
1.20366E-09	0.036590969	0.248	0.22	2.98446E-05
1.22301E-09	0.049523227	0.215	0.189	3.03247E-05
1.24552E-09	0.037415165	0.104	0.086	3.08827E-05
1.30092E-09	0.046947633	0.124	0.105	3.22563E-05
1.32332E-09	0.076748935	0.205	0.181	3.28118E-05
1.35223E-09	0.05411862	0.818	0.811	3.35285E-05
1.43512E-09	0.035444623	0.171	0.148	3.55837E-05
1.49597E-09	0.061950509	0.114	0.096	3.70926E-05
1.57634E-09	-0.125559488	0.325	0.347	3.90854E-05
1.59249E-09	0.118747529	0.331	0.305	3.94858E-05
1.6096E-09	-0.143984873	0.117	0.136	3.991E-05
1.63337E-09	0.050154922	0.237	0.209	4.04995E-05
1.65733E-09	0.076658363	0.367	0.34	4.10936E-05
1.66631E-09	0.076867279	0.589	0.554	4.13162E-05
1.81408E-09	0.065618715	0.236	0.209	4.49802E-05
2.01083E-09	0.051270292	0.763	0.748	4.98585E-05
2.05992E-09	0.034039893	0.108	0.09	5.10758E-05
2.09521E-09	0.075480499	0.605	0.579	5.19508E-05
2.18257E-09	0.052279785	0.124	0.105	5.41169E-05
2.24217E-09	0.065334395	0.141	0.122	5.55946E-05
2.37352E-09	0.131804136	0.33	0.304	5.88515E-05
2.46881E-09	0.051049987	0.134	0.114	6.12141E-05
2.75794E-09	0.031540717	0.152	0.13	6.8383E-05
2.77758E-09	-0.074722645	0.577	0.593	6.887E-05
2.84029E-09	0.056821828	0.189	0.165	7.04249E-05
2.92444E-09	0.041097213	0.26	0.232	7.25116E-05
2.93574E-09	0.050327305	0.14	0.12	7.27916E-05
3.03627E-09	0.037891808	0.125	0.106	7.52843E-05
3.04847E-09	-0.002150489	0.359	0.329	7.55868E-05
3.0914E-09	0.050175812	0.167	0.145	7.66513E-05
3.12565E-09	-0.127160422	0.242	0.264	7.75004E-05
3.14859E-09	-0.114535752	0.16	0.178	7.80693E-05
3.26484E-09	0.06943822	0.51	0.477	8.09517E-05
3.2686E-09	0.027958998	0.122	0.103	8.1045E-05
3.52632E-09	0.047983042	0.982	0.977	8.74352E-05
3.58309E-09	0.037863641	0.237	0.21	8.88427E-05
3.63033E-09	0.068003803	0.316	0.285	9.00141E-05
3.68421E-09	0.038527413	0.14	0.12	9.13501E-05
3.68751E-09	-0.081127669	0.366	0.386	9.14318E-05
3.70054E-09	-0.192750566	0.268	0.285	9.1755E-05
3.74856E-09	0.044412266	0.232	0.205	9.29455E-05
3.85097E-09	0.056272303	0.553	0.518	9.54847E-05
3.8871E-09	-0.095307278	0.368	0.387	9.63807E-05
3.97597E-09	0.02935704	0.18	0.157	9.85842E-05
4.04048E-09	0.067780564	0.498	0.466	0.000100184
4.28239E-09	0.056284063	0.336	0.305	0.000106182

4.86053E-09	0.033387446	0.108	0.091	0.000120517
5.12604E-09	0.052232673	0.237	0.211	0.0001271
5.18865E-09	0.027327688	0.139	0.118	0.000128653
5.2047E-09	0.061188754	0.24	0.214	0.000129051
5.25479E-09	-0.122921608	0.149	0.169	0.000130292
5.34431E-09	0.050712038	0.389	0.356	0.000132512
5.35112E-09	0.051220366	0.344	0.312	0.000132681
5.69719E-09	0.059045399	0.342	0.311	0.000141262
5.73012E-09	0.062655643	0.113	0.095	0.000142078
5.75282E-09	0.087197903	0.341	0.311	0.000142641
5.92362E-09	0.0480598	0.324	0.294	0.000146876
5.95894E-09	0.051408046	0.282	0.254	0.000147752
5.98533E-09	0.044360929	0.247	0.22	0.000148406
6.2253E-09	-0.078568713	0.105	0.121	0.000154356
6.42849E-09	-0.141604722	0.41	0.423	0.000159394
6.58404E-09	0.06461463	0.87	0.861	0.000163251
6.66106E-09	0.125004081	0.123	0.105	0.000165161
6.85004E-09	-0.095958618	0.118	0.135	0.000169847
7.0061E-09	0.039346902	0.252	0.224	0.000173716
7.13621E-09	0.06106153	0.762	0.748	0.000176942
7.29553E-09	0.051081279	0.371	0.339	0.000180893
7.3993E-09	0.061876368	0.611	0.582	0.000183466
7.45685E-09	0.066594286	0.169	0.147	0.000184893
7.71514E-09	0.066748295	0.11	0.093	0.000191297
7.91417E-09	0.064516235	0.283	0.255	0.000196232
8.33075E-09	0.048458907	0.187	0.164	0.000206561
8.41967E-09	0.06109373	0.409	0.376	0.000208766
8.46345E-09	0.039802632	0.32	0.289	0.000209851
8.73072E-09	0.061414422	0.309	0.281	0.000216478
8.86427E-09	0.044331406	0.246	0.219	0.000219789
8.91554E-09	0.062913841	0.155	0.135	0.000221061
9.79645E-09	-0.09806837	0.266	0.285	0.000242903
1.0586E-08	0.072857672	0.372	0.344	0.00026248
1.0778E-08	0.041336864	0.303	0.273	0.00026724
1.09681E-08	0.083310881	0.146	0.127	0.000271954
1.12874E-08	0.064360358	0.787	0.777	0.000279872
1.17195E-08	0.049446581	0.178	0.156	0.000290584
1.17522E-08	0.028316383	0.128	0.109	0.000291396
1.18286E-08	0.04867715	0.128	0.109	0.00029329
1.18656E-08	0.079294481	0.19	0.168	0.000294207
1.21197E-08	0.026425598	0.115	0.098	0.000300509
1.24772E-08	0.076491959	0.563	0.528	0.000309373
1.25412E-08	-0.095038907	0.153	0.171	0.00031096
1.35622E-08	0.081445461	0.556	0.524	0.000336276
1.48958E-08	-0.084744601	0.089	0.105	0.000369342
1.49809E-08	0.055022697	0.297	0.269	0.000371451
1.50351E-08	0.056014862	0.356	0.325	0.000372794
1.53924E-08	0.062290664	0.221	0.196	0.000381654
1.56443E-08	-0.075771694	0.349	0.37	0.000387901
1.57208E-08	0.043140124	0.16	0.139	0.000389798

1.6137E-08	0.072288126	0.595	0.564	0.000400116
1.64375E-08	0.070592233	0.342	0.313	0.000407569
1.74785E-08	-0.089351642	0.356	0.374	0.000433379
1.75239E-08	0.067613841	0.295	0.268	0.000434506
1.76161E-08	0.035491928	0.159	0.138	0.00043679
1.76335E-08	-0.102355174	0.397	0.414	0.000437222
1.81117E-08	0.052684515	0.109	0.092	0.000449079
1.81753E-08	-0.077661877	0.18	0.199	0.000450657
1.82651E-08	0.040952003	0.128	0.11	0.000452882
1.82727E-08	0.070940382	0.677	0.65	0.000453072
1.84226E-08	0.046789794	0.375	0.342	0.000456789
1.94722E-08	0.064857182	0.626	0.593	0.000482813
1.95342E-08	-0.099943611	0.192	0.211	0.00048435
1.97718E-08	0.062098507	0.106	0.09	0.000490242
2.01028E-08	0.086220595	0.13	0.113	0.000498449
2.15828E-08	0.045807227	0.123	0.106	0.000535145
2.18643E-08	0.084925218	0.29	0.264	0.000542124
2.27379E-08	0.060702824	0.115	0.098	0.000563787
2.28581E-08	-0.140078935	0.183	0.201	0.000566766
2.29078E-08	0.037912256	0.216	0.192	0.000567998
2.39792E-08	0.111006324	0.104	0.089	0.000594563
2.43364E-08	0.037273131	0.187	0.165	0.000603422
2.56993E-08	0.035905416	0.107	0.09	0.000637214
2.58671E-08	0.054753746	0.102	0.085	0.000641375
2.60284E-08	0.044888863	0.182	0.16	0.000645374
2.68024E-08	-0.09866775	0.128	0.145	0.000664566
2.82991E-08	0.044179684	0.293	0.265	0.000701677
2.89618E-08	0.044552505	0.221	0.197	0.000718109
3.00484E-08	0.034759654	0.211	0.187	0.000745051
3.01536E-08	0.034214479	0.301	0.272	0.000747659
3.07127E-08	0.054924484	0.816	0.806	0.000761521
3.09606E-08	-0.181441887	0.516	0.518	0.000767668
3.1623E-08	0.065253186	0.82	0.808	0.000784093
3.18457E-08	0.042726435	0.23	0.205	0.000789613
3.23911E-08	0.055297411	0.251	0.225	0.000803137
3.34892E-08	0.062407478	0.893	0.881	0.000830365
3.42816E-08	-0.1077515	0.333	0.351	0.000850011
3.47326E-08	0.049029868	0.265	0.238	0.000861194
3.50779E-08	0.052517879	0.867	0.862	0.000869756
3.57765E-08	0.036705195	0.14	0.121	0.000887079
3.58734E-08	0.036472644	0.101	0.085	0.000889481
3.59648E-08	0.028232665	0.147	0.127	0.000891748
3.69673E-08	0.093984297	0.501	0.468	0.000916605
3.87883E-08	-0.080010756	0.362	0.38	0.000961755
3.92121E-08	-0.048219836	0.732	0.741	0.000972265
3.95339E-08	0.024158816	0.1	0.084	0.000980243
3.98524E-08	0.09837589	0.276	0.252	0.000988141
4.0643E-08	0.036213804	0.24	0.214	0.001007744
4.15593E-08	0.079267492	0.395	0.366	0.001030462
4.17558E-08	0.1070704	0.135	0.118	0.001035334

4.27519E-08	0.050378602	0.212	0.189	0.001060033
4.2991E-08	0.080840766	0.659	0.629	0.001065962
4.31262E-08	0.04853306	0.343	0.313	0.001069314
4.33538E-08	0.047739658	0.223	0.199	0.001074958
4.52392E-08	0.050169843	0.319	0.29	0.001121707
4.56689E-08	0.057627547	0.529	0.496	0.001132361
4.80757E-08	0.041152949	0.115	0.098	0.001192038
4.91629E-08	0.063819171	0.469	0.44	0.001218995
5.04738E-08	0.035845348	0.601	0.572	0.001251498
5.5266E-08	-0.099729889	0.088	0.103	0.001370319
5.5516E-08	-0.068013406	0.41	0.43	0.00137652
5.7005E-08	0.030021998	0.135	0.116	0.001413438
5.91284E-08	0.075816782	0.258	0.233	0.001466089
6.08677E-08	0.204468295	0.109	0.094	0.001509215
6.1462E-08	0.060995414	0.31	0.283	0.00152395
6.17996E-08	0.059965443	0.22	0.197	0.001532321
6.23406E-08	0.068647392	0.156	0.137	0.001545736
6.76066E-08	0.042712558	0.158	0.138	0.001676305
6.77274E-08	-0.079484229	0.765	0.763	0.001679301
6.84774E-08	0.042602464	0.201	0.178	0.001697898
7.06995E-08	-0.101660387	0.466	0.483	0.001752994
7.11889E-08	0.039510015	0.233	0.208	0.001765128
7.17645E-08	0.039582435	0.293	0.265	0.001779402
7.22261E-08	-0.159912739	0.213	0.231	0.001790846
7.3176E-08	0.026040554	0.149	0.129	0.001814398
7.48864E-08	-0.227073613	0.55	0.5	0.001856809
7.61332E-08	0.059098153	0.554	0.519	0.001887723
7.72185E-08	0.056092836	0.857	0.842	0.001914632
7.90241E-08	0.058116765	0.179	0.159	0.001959404
8.1595E-08	0.040219595	0.12	0.103	0.002023148
8.22995E-08	0.073474909	0.516	0.487	0.002040617
8.24494E-08	0.046785352	0.756	0.733	0.002044332
8.32531E-08	0.048547809	0.19	0.169	0.00206426
8.47283E-08	0.019787452	0.145	0.126	0.002100837
8.6363E-08	0.051162188	0.375	0.344	0.002141369
8.92179E-08	0.048080225	0.22	0.198	0.002212159
8.96526E-08	0.073995741	0.18	0.16	0.002222937
9.42172E-08	0.024040415	0.162	0.142	0.002336115
9.5688E-08	0.034887865	0.102	0.086	0.002372583
9.67174E-08	0.096513258	0.255	0.232	0.002398107
9.71968E-08	0.038264417	0.12	0.104	0.002409995
9.74986E-08	0.062311986	0.35	0.322	0.002417477
9.82194E-08	-0.168598412	0.39	0.404	0.00243535
1.04885E-07	0.046150947	0.194	0.172	0.002600626
1.05632E-07	0.040051226	0.101	0.086	0.002619146
1.07143E-07	0.073512782	0.466	0.433	0.002656603
1.07233E-07	0.031835044	0.154	0.134	0.002658849
1.0756E-07	-0.150876072	0.273	0.292	0.002666962
1.08199E-07	0.035169752	0.141	0.122	0.002682785
1.09414E-07	0.045795061	0.37	0.34	0.002712927

1.14143E-07	0.097326927	0.333	0.305	0.002830179
1.20409E-07	-0.124554109	0.197	0.214	0.002985536
1.27195E-07	0.042744405	0.328	0.301	0.003153793
1.28112E-07	0.042121915	0.241	0.217	0.003176528
1.28135E-07	0.03747546	0.111	0.094	0.00317712
1.29464E-07	-0.093695421	0.179	0.196	0.003210053
1.3044E-07	0.027697258	0.136	0.118	0.00323427
1.31273E-07	0.086797774	0.132	0.114	0.003254921
1.31383E-07	0.034024406	0.256	0.231	0.00325765
1.33697E-07	0.039047613	0.172	0.152	0.003315017
1.34063E-07	0.0488903	0.231	0.208	0.003324087
1.37114E-07	0.0434455	0.269	0.243	0.003399751
1.37329E-07	0.038888389	0.214	0.191	0.003405068
1.42194E-07	0.063769402	0.585	0.566	0.003525689
1.45455E-07	0.063243348	0.278	0.253	0.003606565
1.52917E-07	0.016633757	0.192	0.17	0.003791588
1.55079E-07	0.086918216	0.191	0.171	0.003845173
1.55971E-07	0.020360073	0.172	0.151	0.00386731
1.56388E-07	0.039624228	0.15	0.131	0.003877649
1.58249E-07	0.11687311	0.169	0.152	0.003923789
1.60009E-07	-0.078358015	0.576	0.583	0.003967412
1.64364E-07	0.049128145	0.255	0.229	0.004075397
1.65685E-07	0.038681188	0.242	0.218	0.004108172
1.65695E-07	0.054082784	0.139	0.121	0.004108416
1.67958E-07	0.056310689	0.349	0.321	0.004164524
1.77525E-07	0.055659589	0.326	0.298	0.004401725
1.77687E-07	0.223945592	0.229	0.211	0.004405747
1.78108E-07	0.037698929	0.197	0.176	0.004416187
1.78842E-07	0.062859944	0.527	0.496	0.004434394
1.78936E-07	0.042566146	0.195	0.173	0.00443673
1.89419E-07	0.024260354	0.207	0.184	0.004696641
1.9261E-07	0.056111426	0.582	0.555	0.004775776
1.94649E-07	0.111861326	0.28	0.258	0.004826329
1.96751E-07	0.049709952	0.504	0.472	0.004878448
1.97202E-07	0.044909702	0.436	0.403	0.004889616
1.98863E-07	0.025693079	0.197	0.175	0.004930817
2.0067E-07	0.046683931	0.217	0.195	0.004975604
2.02529E-07	0.028138621	0.167	0.148	0.005021714
2.06175E-07	0.045646753	0.101	0.086	0.005112108
2.06208E-07	0.023235239	0.136	0.118	0.005112927
2.13204E-07	-0.138356333	0.273	0.288	0.005286386
2.13923E-07	0.082399481	0.297	0.273	0.005304226
2.15475E-07	0.056233717	0.672	0.643	0.005342693
2.15988E-07	0.057680884	0.791	0.783	0.005355428
2.2379E-07	0.051083062	0.652	0.629	0.005548864
2.24726E-07	0.035820986	0.15	0.131	0.005572081
2.33323E-07	0.05278945	0.863	0.845	0.005785242
2.36343E-07	0.03960485	0.523	0.489	0.005860136
2.38934E-07	0.079608065	0.554	0.527	0.00592436
2.41542E-07	0.04176265	0.418	0.386	0.005989037

2.41579E-07	0.058596448	0.574	0.545	0.005989954
2.42618E-07	-0.07802934	0.09	0.103	0.00601571
2.48541E-07	0.06886897	0.305	0.28	0.006162576
2.5371E-07	0.05857331	0.318	0.291	0.006290749
2.56808E-07	0.033360853	0.433	0.402	0.006367544
2.6017E-07	0.030745661	0.308	0.28	0.00645091
2.62437E-07	0.069818186	0.323	0.296	0.006507114
2.64577E-07	0.030153253	0.102	0.088	0.006560185
2.67817E-07	0.038037089	0.102	0.087	0.006640512
2.70249E-07	0.043247336	0.375	0.345	0.006700815
2.71356E-07	0.020663002	0.121	0.104	0.006728279
2.72654E-07	0.042356553	0.25	0.226	0.006760451
2.80908E-07	0.051994632	0.529	0.497	0.006965115
2.81822E-07	0.045564578	0.196	0.176	0.006987777
2.85021E-07	0.010839555	0.465	0.426	0.007067094
2.87068E-07	0.059356612	0.342	0.317	0.007117861
2.89898E-07	-0.062190013	0.268	0.286	0.007188032
2.96328E-07	0.051368572	0.217	0.194	0.007347463
2.96535E-07	0.075779634	0.183	0.163	0.007352573
3.0109E-07	0.051689777	0.252	0.229	0.007465518
3.01814E-07	0.078683607	0.326	0.301	0.007483486
3.05281E-07	-0.077879225	0.429	0.441	0.007569434
3.08157E-07	0.047328218	0.153	0.135	0.007640749
3.09557E-07	-0.108695584	0.426	0.44	0.007675477
3.09742E-07	0.039816395	0.266	0.24	0.007680044
3.16829E-07	0.015354666	0.279	0.252	0.00785578
3.20986E-07	0.087229526	0.468	0.439	0.007958843
3.29382E-07	0.058186667	0.441	0.411	0.008167026
3.36113E-07	0.065007821	0.372	0.344	0.00833393
3.51944E-07	0.045200208	0.34	0.311	0.008726441
3.60822E-07	0.037956961	0.341	0.312	0.008946574
3.62744E-07	-0.086660504	0.264	0.282	0.008994226
3.64366E-07	0.070983907	0.247	0.225	0.009034456
3.74856E-07	0.046925502	0.209	0.187	0.009294561
3.75912E-07	0.038976975	0.275	0.249	0.009320726
3.7851E-07	-0.079814688	0.19	0.207	0.009385154
3.86248E-07	0.024296604	0.103	0.088	0.009577014
3.93776E-07	0.031944825	0.235	0.211	0.009763683
3.94668E-07	0.053011168	0.461	0.43	0.009785783
3.97887E-07	0.041536384	0.289	0.262	0.009865604
4.01478E-07	0.054243285	0.211	0.19	0.009954654
4.02062E-07	0.043519426	0.282	0.258	0.009969116
4.05872E-07	0.031422286	0.327	0.298	0.010063594
4.11847E-07	0.024542542	0.228	0.204	0.010211737
4.13286E-07	0.037461044	0.26	0.236	0.010247416
4.37145E-07	0.039274036	0.202	0.181	0.01083901
4.74365E-07	0.075410755	0.291	0.266	0.011761875
4.75907E-07	0.049758706	0.559	0.525	0.011800114
4.86479E-07	0.02335324	0.373	0.341	0.012062252
4.90891E-07	0.05711016	0.158	0.14	0.012171644

4.92227E-07	-0.05005548	0.104	0.119	0.012204771
4.95121E-07	0.034807259	0.314	0.286	0.012276513
5.01577E-07	0.022275512	0.187	0.167	0.0124366
5.05638E-07	0.023491592	0.189	0.168	0.012537304
5.07384E-07	0.082380087	0.219	0.198	0.012580586
5.10305E-07	-0.018488567	0.103	0.089	0.012653024
5.28468E-07	0.024531838	0.211	0.189	0.013103363
5.40458E-07	0.045682151	0.636	0.609	0.013400647
5.49074E-07	-0.045150746	0.882	0.888	0.013614288
5.4962E-07	0.05963962	0.569	0.542	0.013627836
5.66107E-07	0.046426483	0.178	0.158	0.014036617
5.71299E-07	-0.093495489	0.271	0.287	0.014165351
5.78402E-07	0.050879053	0.349	0.322	0.014341486
5.80698E-07	0.068385431	0.4	0.375	0.014398416
5.89344E-07	0.114886678	0.947	0.948	0.01461278
5.896E-07	0.044301774	0.408	0.377	0.014619137
5.93859E-07	0.033166375	0.134	0.117	0.014724726
6.06276E-07	0.057442552	0.405	0.377	0.015032622
6.0687E-07	0.045267366	0.148	0.13	0.015047342
6.07495E-07	0.03440103	0.463	0.432	0.015062842
6.11448E-07	0.020682427	0.139	0.121	0.015160851
6.11847E-07	-0.085698249	0.141	0.157	0.015170747
6.14379E-07	0.025497729	0.21	0.188	0.015233527
6.20345E-07	0.033683173	0.226	0.204	0.015381444
6.2087E-07	0.031250887	0.113	0.097	0.015394463
6.31365E-07	0.040879747	0.144	0.127	0.015654688
6.48542E-07	0.032565646	0.186	0.166	0.016080592
6.79158E-07	-0.090753618	0.13	0.144	0.016839723
6.87968E-07	0.020857781	0.125	0.109	0.017058173
6.93931E-07	0.030989369	0.338	0.31	0.01720603
7.29053E-07	0.023848396	0.165	0.146	0.018076879
7.38281E-07	0.034350538	0.191	0.171	0.018305666
7.38722E-07	0.026789838	0.354	0.324	0.018316617
7.45183E-07	0.045237411	0.264	0.24	0.018476808
7.53134E-07	0.055228362	0.386	0.356	0.018673963
7.53887E-07	-0.070565613	0.233	0.25	0.018692616
7.73765E-07	0.054978722	0.263	0.239	0.019185505
7.74234E-07	0.047274756	0.13	0.114	0.019197133
7.92078E-07	-0.068724149	0.227	0.245	0.019639577
7.92169E-07	0.047320958	0.389	0.36	0.019641821
7.93718E-07	0.056588458	0.39	0.361	0.019680243
7.99438E-07	-0.050829879	0.896	0.908	0.019822074
8.02468E-07	0.057928279	0.652	0.626	0.019897183
8.10597E-07	0.040905345	0.1	0.086	0.020098762
8.11302E-07	-0.090496251	0.258	0.274	0.020116234
8.14511E-07	0.029488361	0.488	0.452	0.020195807
8.1913E-07	0.040914914	0.261	0.239	0.020310332
8.35673E-07	0.01979517	0.1	0.085	0.020720507
8.45311E-07	0.066154772	0.194	0.175	0.020959488
8.56345E-07	-0.095644411	0.15	0.165	0.021233084

8.75963E-07	-0.096935234	0.703	0.707	0.0217195
8.91263E-07	-0.084337365	0.216	0.232	0.022098867
8.93549E-07	0.033314873	0.294	0.269	0.022155538
8.95459E-07	0.040975974	0.495	0.464	0.022202909
8.96836E-07	0.047956348	0.231	0.209	0.022237039
9.0369E-07	0.016234778	0.161	0.143	0.022406986
9.18166E-07	0.044409471	0.317	0.29	0.022765933
9.25067E-07	0.024180074	0.231	0.208	0.022937048
9.48091E-07	0.079137807	0.414	0.388	0.023507907
9.53973E-07	-0.036340148	0.875	0.872	0.023653766
9.69301E-07	0.018097636	0.242	0.218	0.024033824
9.73783E-07	0.011345315	0.156	0.138	0.024144951
9.73848E-07	0.029467818	0.283	0.258	0.02414655
9.8911E-07	0.058860978	0.687	0.656	0.024524978
1.00373E-06	-0.101855303	0.154	0.169	0.024887475
1.00479E-06	0.035479243	0.253	0.23	0.024913809
1.00733E-06	0.025422445	0.101	0.086	0.024976854
1.00849E-06	0.037253953	0.234	0.211	0.025005514
1.0312E-06	-0.133278425	0.186	0.202	0.025568544
1.03386E-06	0.019201809	0.21	0.189	0.025634642
1.05512E-06	0.053163258	0.842	0.836	0.026161805
1.09606E-06	0.034463502	0.597	0.564	0.0271769
1.09878E-06	0.054750443	0.572	0.548	0.027244304
1.11678E-06	0.026350108	0.178	0.159	0.027690632
1.11983E-06	0.020999337	0.268	0.244	0.02776611
1.1292E-06	0.048196971	0.216	0.195	0.027998606
1.13146E-06	0.033979436	0.296	0.27	0.028054436
1.14852E-06	0.043196321	0.516	0.485	0.028477546
1.15216E-06	0.045729004	0.116	0.101	0.02856777
1.15891E-06	0.109408465	0.315	0.291	0.028735077
1.16929E-06	0.02807193	0.349	0.32	0.028992431
1.1802E-06	0.040664799	0.25	0.227	0.029263131
1.18183E-06	-0.081513897	0.201	0.217	0.029303518
1.2119E-06	0.027826716	0.191	0.17	0.030049014
1.25046E-06	0.029898501	0.155	0.137	0.031005249
1.2607E-06	0.03913316	0.22	0.199	0.031259179
1.26268E-06	0.020091346	0.201	0.18	0.031308264
1.2753E-06	0.036554572	0.258	0.234	0.031620997
1.29062E-06	-0.109897629	0.194	0.209	0.032000862
1.31363E-06	-0.10005774	0.576	0.58	0.032571359
1.31831E-06	0.075447227	0.175	0.158	0.032687423
1.32928E-06	-0.089889196	0.258	0.274	0.032959553
1.37062E-06	0.06843055	0.179	0.161	0.033984635
1.37109E-06	-0.095694266	0.129	0.143	0.033996264
1.3756E-06	0.037853656	0.719	0.708	0.034108081
1.38463E-06	0.049514735	0.382	0.353	0.034331815
1.41211E-06	0.038223642	0.281	0.257	0.03501334
1.41937E-06	0.0140246	0.146	0.129	0.035193199
1.44624E-06	0.025165294	0.207	0.186	0.035859518
1.48687E-06	0.059205012	0.103	0.089	0.036866921

1.50281E-06	0.054297382	0.397	0.369	0.037262259
1.51408E-06	0.04456962	0.575	0.545	0.037541718
1.51533E-06	0.031695323	0.252	0.229	0.037572719
1.5647E-06	0.085294716	0.1	0.086	0.03879672
1.59311E-06	-0.001638922	0.38	0.348	0.039501186
1.61967E-06	0.018551299	0.182	0.163	0.0401598
1.64787E-06	-0.114626646	0.144	0.158	0.040858856
1.66145E-06	0.02657009	0.16	0.141	0.041195739
1.66785E-06	0.025685749	0.142	0.126	0.041354418
1.71235E-06	0.053127858	0.222	0.201	0.042457668
1.77125E-06	0.045232352	0.436	0.407	0.043918064
1.79668E-06	-0.051165118	0.876	0.879	0.044548649
1.81028E-06	0.040151465	0.322	0.297	0.044885904
1.8549E-06	0.027600825	0.112	0.097	0.045992344
1.89701E-06	0.028338622	0.183	0.164	0.047036279
1.90862E-06	0.04943411	0.255	0.233	0.047324232
1.9105E-06	0.019383148	0.128	0.112	0.047370775
1.92539E-06	-0.082352207	0.3	0.316	0.047740085
1.97352E-06	0.042846888	0.387	0.358	0.048933365
1.99421E-06	0.025893586	0.134	0.118	0.049446358
2.00897E-06	0.034886187	0.147	0.13	0.049812424
2.01868E-06	0.053760331	0.349	0.323	0.050053235
2.01965E-06	0.056162256	0.377	0.352	0.05007714
2.04452E-06	0.015461638	0.178	0.159	0.050693863
2.13655E-06	0.027233604	0.245	0.221	0.052975706
2.1539E-06	0.099032442	0.625	0.596	0.053405945
2.20573E-06	0.028080513	0.182	0.163	0.054691068
2.22566E-06	0.017385827	0.918	0.897	0.055185212
2.28384E-06	0.055933495	0.184	0.166	0.056627791
2.35936E-06	0.032844202	0.387	0.359	0.058500349
2.36647E-06	0.033595676	0.275	0.25	0.058676692
2.37292E-06	0.098775361	0.512	0.488	0.058836623
2.39283E-06	-0.135775688	0.245	0.262	0.059330185
2.39474E-06	-0.077700591	0.505	0.514	0.059377689
2.43002E-06	0.037988658	0.198	0.178	0.060252464
2.47199E-06	-0.071166001	0.124	0.138	0.061293043
2.489E-06	0.055366413	0.154	0.137	0.061714747
2.60007E-06	0.044925748	0.266	0.244	0.064468763
2.64282E-06	0.034316601	0.102	0.088	0.065528834
2.67584E-06	0.061843094	0.379	0.352	0.066347433
2.7198E-06	0.072254731	0.269	0.247	0.067437458
2.72741E-06	0.030160959	0.223	0.202	0.06762609
2.77745E-06	0.052680096	0.587	0.56	0.068866866
2.80528E-06	0.035202555	0.323	0.298	0.069556941
2.85934E-06	0.034864456	0.183	0.164	0.070897332
2.92846E-06	-0.107151732	0.243	0.257	0.072611207
2.94365E-06	0.024442214	0.203	0.182	0.072987819
2.96413E-06	0.01850195	0.226	0.204	0.073495563
2.99787E-06	0.026187419	0.143	0.126	0.074332291
3.06313E-06	-0.073013447	0.118	0.132	0.075950387

3.11138E-06	0.020502591	0.235	0.213	0.077146715
3.20587E-06	0.025188352	0.277	0.252	0.07948958
3.2173E-06	0.053216926	0.619	0.592	0.079772893
3.23276E-06	0.040293021	0.221	0.2	0.080156333
3.23976E-06	0.029126974	0.154	0.137	0.080329867
3.27606E-06	0.043785562	0.267	0.244	0.081230013
3.28329E-06	0.038028376	0.49	0.46	0.081409238
3.29159E-06	0.04763025	0.169	0.152	0.081615078
3.34468E-06	0.058222579	0.529	0.499	0.082931288
3.44878E-06	0.043470967	0.172	0.154	0.085512563
3.49149E-06	0.093942822	0.182	0.166	0.086571453
3.50812E-06	-0.079102597	0.098	0.11	0.086983934
3.51903E-06	0.045302388	0.246	0.223	0.087254404
3.52364E-06	0.044827825	0.1	0.087	0.08736857
3.53278E-06	0.030475277	0.244	0.222	0.08759534
3.53323E-06	0.039709086	0.377	0.348	0.08760653
3.56478E-06	0.059414469	0.142	0.126	0.088388771
3.58985E-06	0.048861806	0.209	0.189	0.089010391
3.59592E-06	0.066719876	0.227	0.208	0.08916077
3.60312E-06	0.055763121	0.514	0.491	0.089339458
3.62822E-06	0.027571778	0.158	0.141	0.089961785
3.66112E-06	0.129973299	0.157	0.142	0.090777592
3.75689E-06	-0.062864817	0.099	0.112	0.093152146
3.81951E-06	0.049131984	0.302	0.279	0.094704654
3.86696E-06	-0.047930976	0.839	0.843	0.095881361
3.89262E-06	0.037286476	0.104	0.09	0.096517494
3.93673E-06	0.055226874	0.455	0.425	0.09761124
3.99955E-06	0.045395591	0.246	0.224	0.099168925
4.06992E-06	0.050371285	0.324	0.299	0.100913589
4.08144E-06	0.038755467	0.232	0.211	0.10119928
4.08815E-06	0.025844522	0.706	0.686	0.10136579
4.08975E-06	0.05755419	0.303	0.28	0.101405278
4.13989E-06	0.041680475	0.367	0.341	0.102648472
4.17368E-06	-0.089311815	0.304	0.318	0.103486423
4.25119E-06	-0.040760381	0.685	0.697	0.105408297
4.30479E-06	0.02477907	0.238	0.216	0.106737329
4.31924E-06	0.025411364	0.325	0.299	0.107095455
4.34426E-06	0.160028261	0.312	0.294	0.107715952
4.4301E-06	-0.080801301	0.286	0.299	0.109844451
4.43982E-06	0.026239405	0.129	0.114	0.110085379
4.46105E-06	0.020557432	0.277	0.253	0.110611644
4.63119E-06	0.058768229	0.603	0.573	0.114830281
4.67424E-06	0.049133111	0.238	0.217	0.11589769
4.68691E-06	0.04383956	0.133	0.118	0.116211923
4.70405E-06	-0.022581398	0.972	0.965	0.116637027
4.76117E-06	-0.083243756	0.266	0.28	0.118053181
4.79185E-06	0.034030949	0.223	0.202	0.118813814
4.84127E-06	0.02487505	0.402	0.373	0.12003918
4.97604E-06	0.048182169	0.335	0.311	0.123380825
5.0614E-06	0.068211975	0.163	0.146	0.125497321

5.09303E-06	0.031939906	0.24	0.218	0.126281699
5.11952E-06	0.035576382	0.259	0.237	0.126938587
5.21214E-06	0.02709307	0.198	0.178	0.129234983
5.40274E-06	0.038227586	0.195	0.176	0.133960957
5.42379E-06	0.182122718	0.266	0.245	0.134482877
5.44889E-06	0.053027333	0.306	0.283	0.135105216
5.56659E-06	0.054254506	0.503	0.478	0.13802368
5.61472E-06	0.023926617	0.103	0.09	0.139216879
5.70123E-06	0.038230971	0.418	0.388	0.141362061
5.78763E-06	0.037267301	0.142	0.127	0.143504383
5.84979E-06	0.016974836	0.283	0.259	0.145045483
5.85649E-06	0.037676764	0.437	0.407	0.145211621
5.95162E-06	0.022279263	0.115	0.101	0.147570432
5.99781E-06	0.035871254	0.273	0.251	0.148715756
6.17693E-06	0.075086905	0.141	0.127	0.153156908
6.26471E-06	0.077232916	0.415	0.389	0.155333467
6.38828E-06	-0.087011111	0.21	0.225	0.158397467
6.41926E-06	0.025885637	0.144	0.129	0.159165627
6.66236E-06	-0.072782834	0.154	0.168	0.16519321
6.76831E-06	0.028415588	0.19	0.171	0.167820339
6.80562E-06	0.043471513	0.824	0.81	0.168745439
6.81079E-06	0.039181086	0.273	0.251	0.168873613
6.86256E-06	0.015476708	0.136	0.12	0.170157122
6.86271E-06	0.048454004	0.374	0.347	0.17016095
6.86783E-06	0.049240117	0.106	0.093	0.170287869
7.01038E-06	-0.102890778	0.177	0.192	0.173822478
7.08278E-06	0.048410757	0.384	0.357	0.175617644
7.13772E-06	0.006657459	0.208	0.187	0.176979886
7.27681E-06	0.021352033	0.302	0.276	0.180428577
7.27765E-06	0.01883482	0.14	0.125	0.180449363
7.28243E-06	0.041778484	0.179	0.161	0.180567874
7.43082E-06	-0.073556168	0.107	0.12	0.184247254
7.49582E-06	0.043704321	0.298	0.276	0.185858902
7.52388E-06	0.023282757	0.143	0.127	0.186554562
7.56059E-06	-0.08329353	0.334	0.347	0.187464783
7.59671E-06	0.042797303	0.537	0.511	0.188360448
7.60724E-06	0.020904369	0.242	0.22	0.188621638
7.64541E-06	-0.050697603	0.174	0.19	0.189567905
7.81723E-06	0.032753201	0.187	0.168	0.193828334
7.88436E-06	0.031739923	0.361	0.335	0.195492711
7.98674E-06	0.02027468	0.136	0.12	0.198031194
8.25853E-06	0.020658851	0.266	0.246	0.204770203
8.44738E-06	0.043649581	0.659	0.634	0.209452852
8.52298E-06	0.027330484	0.478	0.447	0.211327398
8.55494E-06	0.026241156	0.203	0.184	0.212119834
8.6301E-06	0.029100083	0.217	0.197	0.213983442
8.64467E-06	0.037205808	0.112	0.098	0.214344706
8.76696E-06	-0.05211849	0.701	0.703	0.217376786
8.85372E-06	0.01433401	0.132	0.117	0.21952803
8.85653E-06	0.08121823	0.198	0.18	0.219597545

8.96135E-06	0.053744961	0.316	0.292	0.222196705
8.96395E-06	-0.09786822	0.748	0.749	0.222261048
8.99081E-06	0.007483209	0.128	0.113	0.222927239
9.26319E-06	0.013442102	0.128	0.112	0.229680866
9.27847E-06	0.023178674	0.175	0.157	0.23005976
9.3735E-06	0.046021159	0.414	0.388	0.23241604
9.40719E-06	0.048778832	0.19	0.172	0.233251158
9.41279E-06	0.035881138	0.25	0.228	0.233390167
9.58523E-06	0.016399242	0.16	0.143	0.237665799
9.59237E-06	0.042042192	0.343	0.318	0.237842918
9.624E-06	0.01584111	0.167	0.149	0.238626961
9.84372E-06	0.019967806	0.261	0.238	0.244074971
9.96215E-06	-0.086984708	0.096	0.108	0.247011465
1.01262E-05	0.038385241	0.312	0.287	0.251078368
1.01349E-05	0.101005702	0.289	0.27	0.25129583
1.01985E-05	0.018035659	0.121	0.106	0.252871629
1.02144E-05	-0.104892527	0.133	0.146	0.253265146
1.0487E-05	0.024766522	0.143	0.127	0.260026128
1.06277E-05	0.045157228	0.455	0.424	0.263514097
1.06338E-05	0.015501691	0.191	0.173	0.26366391
1.06493E-05	0.059200124	0.575	0.546	0.264048684
1.08616E-05	0.177493858	0.293	0.279	0.269314342
1.08753E-05	0.029600024	0.199	0.181	0.269653729
1.09291E-05	-0.150252383	0.24	0.253	0.270985942
1.09438E-05	0.023090804	0.134	0.119	0.271351433
1.09774E-05	0.050595305	0.225	0.206	0.272185465
1.12423E-05	0.042178631	0.528	0.501	0.278751803
1.12869E-05	0.063554017	0.494	0.469	0.279857672
1.13086E-05	0.037182509	0.191	0.173	0.280397104
1.13116E-05	0.046501634	0.338	0.314	0.280472232
1.13308E-05	0.046209571	0.132	0.117	0.280947484
1.14249E-05	0.036249059	0.329	0.304	0.28328009
1.14951E-05	0.049754639	0.418	0.393	0.285021224
1.15048E-05	0.044297473	0.524	0.493	0.285260601
1.15088E-05	0.049235285	0.758	0.75	0.285361712
1.15537E-05	0.056810525	0.257	0.236	0.286473271
1.15833E-05	0.022273684	0.13	0.115	0.287208447
1.17765E-05	0.089132236	0.731	0.708	0.291997286
1.19001E-05	0.063787351	0.462	0.434	0.295062438
1.20003E-05	0.049911427	0.222	0.203	0.29754814
1.21126E-05	0.009832772	0.179	0.161	0.300332773
1.22943E-05	-0.09241154	0.108	0.12	0.304836708
1.2322E-05	0.019770444	0.245	0.223	0.305524372
1.23544E-05	-0.110804855	0.18	0.193	0.306328079
1.23821E-05	-0.088182486	0.191	0.204	0.307014427
1.24264E-05	0.016719201	0.141	0.125	0.308112817
1.24731E-05	0.037980347	0.131	0.116	0.309269955
1.27831E-05	0.02884897	0.107	0.094	0.316958058
1.32033E-05	0.019721711	0.158	0.141	0.327375772
1.32071E-05	-0.075531108	0.097	0.109	0.32747121

1.32539E-05	0.01691463	0.178	0.16	0.328630711
1.32711E-05	0.038811849	0.501	0.475	0.329057059
1.33276E-05	0.027686744	0.371	0.345	0.330457326
1.34514E-05	0.019473411	0.184	0.165	0.333527811
1.36564E-05	0.022729604	0.224	0.204	0.338611261
1.36982E-05	0.038273925	0.611	0.581	0.339647283
1.37132E-05	0.022072113	0.288	0.265	0.340019104
1.37165E-05	0.012973727	0.301	0.276	0.340101308
1.37615E-05	0.011906219	0.215	0.195	0.341216085
1.37752E-05	0.031883788	0.381	0.356	0.341557193
1.38261E-05	0.040664968	0.221	0.201	0.34281874
1.40523E-05	0.016498846	0.131	0.116	0.348427871
1.43456E-05	0.057553068	0.105	0.093	0.355699325
1.43699E-05	0.014016602	0.388	0.359	0.356300709
1.44091E-05	0.04077621	0.549	0.519	0.357273764
1.45357E-05	0.006007152	0.191	0.172	0.360413233
1.45569E-05	0.031967485	0.243	0.223	0.360937867
1.45623E-05	0.024777986	0.261	0.238	0.361072714
1.45774E-05	0.053437728	0.57	0.545	0.361446552
1.46134E-05	0.01395302	0.115	0.101	0.362339125
1.4639E-05	0.040101824	0.339	0.314	0.362973679
1.48691E-05	-0.070841604	0.203	0.216	0.368678342
1.503E-05	0.029337853	0.423	0.393	0.372667874
1.54656E-05	0.011468542	0.102	0.089	0.383468471
1.5556E-05	0.022491031	0.125	0.11	0.385710887
1.576E-05	0.045016752	0.264	0.243	0.390769383
1.59275E-05	0.021785304	0.158	0.142	0.394922089
1.59993E-05	0.028034756	0.105	0.092	0.396702355
1.60581E-05	0.014354837	0.172	0.155	0.39816
1.60989E-05	0.081740483	0.212	0.195	0.399172892
1.61793E-05	-0.053591458	0.089	0.1	0.401164538
1.62455E-05	0.012940631	0.321	0.296	0.402807762
1.62771E-05	0.015833734	0.104	0.091	0.403589719
1.64352E-05	0.029494211	0.298	0.275	0.407511063
1.68825E-05	0.032913466	0.356	0.332	0.418602429
1.72112E-05	0.018426117	0.127	0.112	0.426750938
1.72161E-05	0.01377892	0.2	0.182	0.426872003
1.74288E-05	0.012714938	0.181	0.163	0.4321471
1.75319E-05	0.02741295	0.153	0.137	0.434702437
1.77079E-05	0.009663915	0.186	0.168	0.439068362
1.79441E-05	-0.099438266	0.249	0.263	0.444924684
1.79879E-05	0.026197873	0.145	0.13	0.44601035
1.80304E-05	0.032454414	0.137	0.122	0.447063529
1.83197E-05	0.047465358	0.547	0.519	0.454236166
1.84441E-05	-0.088307736	0.207	0.221	0.457322674
1.84609E-05	0.127838472	0.124	0.111	0.457738531
1.87347E-05	0.018289345	0.316	0.291	0.464527771
1.89911E-05	0.031645657	0.181	0.164	0.470885336
1.94758E-05	0.014262631	0.103	0.09	0.482902552
1.9489E-05	0.041283257	0.47	0.441	0.483229427

1.9553E-05	0.016718541	0.123	0.109	0.484816355
1.96803E-05	-0.127450612	0.195	0.208	0.487973209
1.97664E-05	0.010772439	0.211	0.191	0.490107926
1.98818E-05	0.019771949	0.153	0.136	0.492968502
1.99061E-05	0.022437581	0.211	0.192	0.493571218
2.02215E-05	0.02268562	0.721	0.707	0.501391125
2.02216E-05	0.015715401	0.112	0.099	0.501395025
2.02907E-05	0.027421559	0.309	0.287	0.50310725
2.03324E-05	0.018616817	0.131	0.116	0.504141982
2.037E-05	0.052965944	0.168	0.152	0.505074231
2.04688E-05	0.032596343	0.334	0.311	0.507524294
2.06922E-05	-0.111879597	0.274	0.285	0.513062219
2.0845E-05	0.064821217	0.347	0.325	0.516852533
2.09951E-05	0.027950786	0.136	0.121	0.520574489
2.11142E-05	0.043753285	0.228	0.209	0.523527596
2.12081E-05	-0.120477718	0.093	0.103	0.525854971
2.13741E-05	0.029386758	0.459	0.43	0.529970716
2.14162E-05	0.034252271	0.362	0.337	0.53101493
2.19358E-05	0.040717796	0.456	0.426	0.543898721
2.21084E-05	-0.154945607	0.392	0.4	0.548177611
2.22162E-05	0.023870321	0.889	0.883	0.550849527
2.23515E-05	0.016856779	0.277	0.254	0.554204676
2.28208E-05	0.076244302	0.493	0.477	0.565842223
2.30114E-05	-0.052767601	0.129	0.143	0.570567103
2.30629E-05	0.019617284	0.16	0.144	0.571844413
2.31258E-05	0.056234114	0.154	0.139	0.573403256
2.33987E-05	0.017541394	0.118	0.104	0.580171891
2.34915E-05	-0.104425703	0.147	0.159	0.58247079
2.37332E-05	0.023449271	0.14	0.126	0.588464943
2.39228E-05	0.04139055	0.115	0.102	0.593165387
2.39734E-05	0.017043876	0.108	0.095	0.594419869
2.45757E-05	0.043290372	0.357	0.333	0.609355641
2.4672E-05	0.021601903	0.115	0.102	0.611742591
2.47618E-05	0.0228883	0.101	0.089	0.613968543
2.48065E-05	0.010138231	0.203	0.184	0.615077394
2.49647E-05	0.036068853	0.399	0.374	0.618999103
2.49907E-05	0.034093591	0.108	0.095	0.619645636
2.50132E-05	0.024937513	0.303	0.28	0.620202782
2.51481E-05	0.011866469	0.105	0.092	0.623547901
2.54643E-05	0.011374941	0.129	0.114	0.631388313
2.56899E-05	0.036986943	0.159	0.144	0.636981968
2.62684E-05	-0.067507075	0.25	0.264	0.651325485
2.6311E-05	0.0112313	0.383	0.354	0.652382235
2.63125E-05	0.076351079	0.123	0.11	0.652417534
2.67613E-05	0.046888629	0.144	0.128	0.663546881
2.70317E-05	0.079206176	0.124	0.111	0.670252081
2.70638E-05	-0.08914021	0.105	0.116	0.671046912
2.71891E-05	0.023587635	0.188	0.171	0.674153666
2.74468E-05	0.036512637	0.448	0.419	0.680544556
2.80371E-05	0.015370966	0.148	0.132	0.695180673

2.8367E-05	-0.074867754	0.207	0.221	0.703359186
2.85552E-05	0.00997763	0.197	0.178	0.708026628
2.88328E-05	0.023181777	0.158	0.141	0.714909382
2.91181E-05	0.037408074	0.114	0.101	0.721983074
2.91549E-05	0.025689701	0.229	0.21	0.72289461
2.91703E-05	0.023033057	0.211	0.192	0.72327828
2.91925E-05	0.061079711	0.603	0.574	0.723828068
2.99468E-05	0.036431279	0.173	0.157	0.742530756
3.0053E-05	0.026206162	0.262	0.241	0.745163969
3.09201E-05	0.018907431	0.231	0.211	0.766664903
3.11812E-05	0.041609336	0.313	0.29	0.773136673
3.13841E-05	-0.064502848	0.09	0.101	0.778168421
3.1981E-05	-0.073260917	0.114	0.126	0.792970023
3.29323E-05	0.013067938	0.16	0.144	0.816557591
3.29769E-05	-0.107077933	0.319	0.33	0.817661726
3.30181E-05	-0.115083779	0.159	0.171	0.818683125
3.32234E-05	0.036165004	0.238	0.218	0.82377507
3.33088E-05	0.024577014	0.106	0.093	0.825890618
3.33452E-05	0.024002597	0.217	0.198	0.826794608
3.33776E-05	0.050221086	0.543	0.525	0.827596719
3.34245E-05	0.011240406	0.311	0.288	0.828761576
3.37277E-05	0.019655724	0.262	0.24	0.836279306
3.38271E-05	-0.079332429	0.275	0.287	0.83874358
3.38568E-05	0.029560732	0.113	0.1	0.839478944
3.44723E-05	0.032396382	0.165	0.15	0.854741444
3.45674E-05	-0.04547398	0.318	0.333	0.85709918
3.50567E-05	0.008569333	0.209	0.19	0.869229841
3.50718E-05	0.019782605	0.144	0.129	0.869605952
3.51628E-05	0.046800327	0.586	0.56	0.871861271
3.53561E-05	0.059062505	0.321	0.3	0.876655206
3.53602E-05	0.033470092	0.449	0.419	0.876755852
3.53773E-05	0.0169257	0.167	0.151	0.87717908
3.54471E-05	0.01702723	0.163	0.146	0.878911964
3.55263E-05	-0.098733087	0.336	0.345	0.880873489
3.5852E-05	0.043401246	0.375	0.349	0.888949267
3.59764E-05	0.019012505	0.209	0.191	0.892034529
3.60255E-05	0.0188796	0.229	0.21	0.893252248
3.61027E-05	-0.091727664	0.62	0.616	0.89516699
3.63053E-05	0.045097095	0.785	0.774	0.900189847
3.6355E-05	0.02299946	0.174	0.158	0.901420988
3.67967E-05	0.041572104	0.477	0.449	0.912373089
3.71594E-05	-0.090866451	0.151	0.163	0.921368203
3.71689E-05	0.02791007	0.121	0.108	0.92160231
3.72347E-05	0.017185559	0.327	0.301	0.9232343
3.76636E-05	0.025609674	0.139	0.124	0.933868787
3.82667E-05	0.028712258	0.135	0.121	0.948822601
3.83786E-05	-0.057405235	0.18	0.194	0.951597675
3.85172E-05	-0.002027415	0.16	0.144	0.955032857
3.90006E-05	0.015144967	0.137	0.122	0.967019565
3.90615E-05	0.024159314	0.12	0.106	0.968530636

3.97051E-05	0.041313016	0.375	0.352	0.984488795
4.00751E-05	0.020200797	0.141	0.126	0.993660969
0.36507791	-0.624500799	0.134	0.136	1
0.095026997	-0.595449189	0.296	0.284	1
0.161690696	-0.592570944	0.435	0.437	1
0.003094589	-0.313531287	0.126	0.133	1
0.000819928	-0.30152574	0.117	0.127	1
0.085685658	-0.180739923	0.104	0.108	1
0.565450369	-0.13768042	0.214	0.212	1
0.891986509	-0.136383591	0.329	0.319	1
9.93581E-05	-0.135922171	0.789	0.785	1
0.09255075	-0.128809947	0.144	0.148	1
0.000648185	-0.122369578	0.807	0.8	1
5.85678E-05	-0.122060046	0.666	0.663	1
7.40246E-05	-0.117964044	0.18	0.191	1
0.00086512	-0.116497831	0.199	0.208	1
0.000605619	-0.115315131	0.24	0.25	1
0.003628282	-0.113716498	0.131	0.138	1
0.000305517	-0.113643496	0.301	0.312	1
7.61189E-05	-0.113044181	0.417	0.428	1
0.001893099	-0.110050489	0.472	0.471	1
0.01627906	-0.109347341	0.13	0.136	1
0.105947423	-0.108143626	0.925	0.919	1
0.000390879	-0.107669688	0.329	0.334	1
4.25837E-05	-0.106793685	0.334	0.342	1
0.060505857	-0.106622556	0.217	0.218	1
0.73380621	-0.105998827	0.133	0.13	1
0.042104804	-0.102890411	0.249	0.251	1
0.000898671	-0.102530104	0.435	0.443	1
0.096204569	-0.101854348	0.251	0.253	1
0.000135378	-0.100903574	0.232	0.243	1
0.106790915	-0.099801214	0.279	0.279	1
6.52533E-05	-0.099566125	0.295	0.305	1
0.000187177	-0.098532308	0.163	0.174	1
0.000257372	-0.097533298	0.265	0.274	1
0.00365696	-0.097422206	0.156	0.163	1
0.077364421	-0.096180089	0.248	0.25	1
0.000326929	-0.092760006	0.2	0.209	1
0.004527567	-0.092387014	0.329	0.332	1
0.012560097	-0.091950679	0.221	0.227	1
0.000213055	-0.091788646	0.346	0.354	1
0.027200135	-0.091346311	0.202	0.207	1
0.612147952	-0.090782794	0.579	0.569	1
0.000250946	-0.090068846	0.183	0.194	1
0.000282521	-0.089528618	0.676	0.683	1
0.196104788	-0.089392363	0.21	0.202	1
0.000321329	-0.088736179	0.204	0.215	1
0.522981416	-0.087954416	0.247	0.243	1
0.000289215	-0.087509805	0.345	0.352	1
0.001564903	-0.087045667	0.121	0.13	1

0.002003768	-0.08694235	0.384	0.386	1
0.010209646	-0.08665939	0.13	0.137	1
0.001751257	-0.086250038	0.188	0.196	1
0.001979691	-0.08386507	0.455	0.452	1
0.003922295	-0.083633545	0.108	0.116	1
0.000113934	-0.08309442	0.193	0.204	1
0.000699949	-0.082964215	0.26	0.27	1
0.0015711	-0.08267393	0.125	0.134	1
0.010747429	-0.082639597	0.17	0.175	1
0.000855241	-0.082251087	0.095	0.103	1
0.002491346	-0.082087707	0.194	0.202	1
0.194533303	-0.081937718	0.387	0.376	1
0.137689315	-0.081511562	0.275	0.277	1
0.213906482	-0.081268144	0.244	0.245	1
0.018907689	-0.080027413	0.221	0.227	1
8.14518E-05	-0.079664569	0.333	0.345	1
0.00108488	-0.079420379	0.148	0.157	1
0.000108639	-0.079294522	0.204	0.216	1
0.294951666	-0.078491697	0.288	0.284	1
0.009928334	-0.078223605	0.387	0.391	1
0.015277945	-0.078135686	0.132	0.138	1
0.001919344	-0.077795487	0.169	0.176	1
0.000277124	-0.077362904	0.121	0.131	1
0.015410843	-0.076747096	0.176	0.182	1
0.000476483	-0.076691535	0.434	0.404	1
0.064221363	-0.076374761	0.301	0.3	1
0.255863281	-0.076260546	0.95	0.946	1
0.018531372	-0.075744484	0.128	0.119	1
0.009808247	-0.075700191	0.218	0.225	1
0.000949813	-0.07533688	0.135	0.145	1
0.006433452	-0.075134108	0.191	0.198	1
0.000916388	-0.07447457	0.393	0.402	1
0.033479987	-0.074442793	0.227	0.233	1
0.000312505	-0.074212016	0.282	0.293	1
0.003611401	-0.074021279	0.108	0.115	1
0.000285054	-0.07397613	0.217	0.229	1
0.000555289	-0.073560745	0.186	0.196	1
0.10933687	-0.073464359	0.344	0.344	1
0.003287316	-0.073427447	0.548	0.547	1
0.000703932	-0.073070702	0.614	0.609	1
0.000540723	-0.073003506	0.091	0.1	1
0.024001026	-0.072879872	0.308	0.31	1
0.016745277	-0.072647042	0.197	0.202	1
0.000871808	-0.072339011	0.202	0.211	1
0.000503804	-0.072193794	0.11	0.119	1
0.092417668	-0.072100302	0.312	0.313	1
0.02503601	-0.072031158	0.126	0.131	1
0.008040352	-0.071802765	0.207	0.214	1
0.031261208	-0.071703714	0.184	0.189	1
0.000186892	-0.071488652	0.404	0.411	1

0.001494271	-0.071395293	0.319	0.327	1
5.38367E-05	-0.071392275	0.108	0.12	1
0.000898729	-0.071219121	0.527	0.53	1
0.003524489	-0.070995183	0.197	0.205	1
0.947738623	-0.070829327	0.322	0.316	1
0.010762985	-0.070817179	0.103	0.109	1
0.011212397	-0.070790787	0.162	0.168	1
0.001449533	-0.07076598	0.408	0.412	1
0.000119259	-0.070667286	0.14	0.152	1
0.000920038	-0.070447102	0.502	0.503	1
8.99993E-05	-0.070447038	0.116	0.128	1
0.000893684	-0.070406473	0.296	0.305	1
0.004801549	-0.069991881	0.15	0.159	1
0.009162238	-0.069665306	0.225	0.231	1
0.003983966	-0.069508047	0.379	0.381	1
5.88746E-05	-0.069397773	0.137	0.149	1
0.029627211	-0.069291655	0.335	0.339	1
4.59456E-05	-0.069214915	0.482	0.484	1
0.000189262	-0.068982523	0.256	0.266	1
0.000566226	-0.068591479	0.122	0.132	1
0.024634895	-0.068448664	0.121	0.127	1
0.001742864	-0.068257807	0.161	0.17	1
0.091830636	-0.06787068	0.131	0.134	1
0.048693221	-0.06784727	0.265	0.267	1
8.07871E-05	-0.067798031	0.114	0.125	1
0.004555065	-0.06750789	0.172	0.181	1
0.001721419	-0.067496444	0.222	0.231	1
0.002209823	-0.067420822	0.214	0.223	1
0.040459974	-0.067080236	0.225	0.228	1
0.063189475	-0.067003962	0.15	0.154	1
0.000255032	-0.066773497	0.184	0.195	1
0.077105535	-0.066371765	0.433	0.427	1
0.012871907	-0.066222517	0.235	0.242	1
8.84303E-05	-0.066134791	0.172	0.185	1
0.002191505	-0.066034959	0.374	0.38	1
0.001046455	-0.065788675	0.299	0.309	1
0.001546087	-0.065704521	0.136	0.145	1
0.013004402	-0.065531286	0.203	0.209	1
0.313462264	-0.065388321	0.281	0.279	1
0.426429293	-0.065297183	0.25	0.24	1
0.043119437	-0.065104434	0.138	0.142	1
0.000205643	-0.064929029	0.304	0.317	1
0.014244759	-0.064694928	0.336	0.338	1
0.003507953	-0.064679121	0.438	0.442	1
0.007406271	-0.064613196	0.348	0.353	1
0.001097595	-0.064169977	0.143	0.152	1
0.002896427	-0.064146794	0.148	0.157	1
0.253664058	-0.064057905	0.303	0.299	1
0.100723704	-0.06380682	0.36	0.358	1
0.005611497	-0.063773295	0.188	0.195	1

0.001424663	-0.063524853	0.284	0.293	1
0.0006946	-0.063462162	0.183	0.192	1
0.004271757	-0.063292894	0.136	0.143	1
0.042386975	-0.063278036	0.313	0.314	1
0.000205343	-0.063189557	0.358	0.367	1
0.046574347	-0.063157774	0.145	0.149	1
0.004811449	-0.06313677	0.099	0.107	1
0.170562999	-0.063041891	0.174	0.177	1
0.007809238	-0.06290153	0.275	0.282	1
0.630753067	-0.06284619	0.315	0.312	1
0.041665378	-0.06283719	0.106	0.111	1
0.306173522	-0.062558096	0.376	0.371	1
0.007532238	-0.062551582	0.127	0.134	1
0.014466915	-0.062301101	0.198	0.205	1
0.000651586	-0.062300514	0.404	0.409	1
0.156571784	-0.062261884	0.132	0.135	1
0.003868298	-0.062235184	0.11	0.118	1
0.712890676	-0.062204567	0.111	0.112	1
0.000493944	-0.062111118	0.187	0.197	1
0.000855567	-0.062098874	0.143	0.153	1
0.472783517	-0.061899598	0.23	0.227	1
0.014734114	-0.061854313	0.229	0.236	1
0.001533381	-0.061714386	0.267	0.277	1
0.021883978	-0.061631294	0.114	0.12	1
0.129151938	-0.061331786	0.191	0.193	1
0.000568366	-0.061124098	0.332	0.342	1
0.000948776	-0.060765791	0.304	0.311	1
0.04840569	-0.060533293	0.249	0.252	1
0.060066116	-0.060466086	0.253	0.256	1
0.064424804	-0.060369233	0.245	0.247	1
0.000321104	-0.060303313	0.176	0.187	1
0.008910906	-0.060249423	0.124	0.131	1
0.598366285	-0.060242361	0.172	0.17	1
0.003775091	-0.060059202	0.201	0.21	1
0.008129525	-0.059413454	0.107	0.113	1
0.037619362	-0.059039602	0.105	0.11	1
0.052175285	-0.058651317	0.157	0.161	1
0.060726399	-0.058640842	0.174	0.179	1
0.025899901	-0.058587634	0.623	0.613	1
0.3942876	-0.058416176	0.383	0.378	1
0.351712433	-0.058287879	0.249	0.247	1
0.076285552	-0.058231639	0.185	0.188	1
0.085738957	-0.058060168	0.151	0.154	1
0.068947255	-0.058039665	0.127	0.131	1
0.000289822	-0.05793372	0.261	0.273	1
0.206355366	-0.057870413	0.124	0.127	1
0.00770509	-0.057792752	0.254	0.261	1
0.001600079	-0.057744296	0.153	0.162	1
0.002816449	-0.057601289	0.17	0.179	1
0.000220022	-0.057499508	0.164	0.178	1

0.008115101	-0.057489089	0.462	0.462	1
0.009298479	-0.057470269	0.18	0.188	1
0.016439939	-0.057262703	0.118	0.124	1
0.019923401	-0.057105639	0.204	0.21	1
8.03536E-05	-0.057020846	0.153	0.165	1
0.002356143	-0.056829258	0.275	0.284	1
0.021512986	-0.056682463	0.165	0.171	1
0.1206914	-0.056656878	0.256	0.258	1
0.000104511	-0.056656851	0.257	0.27	1
0.22641033	-0.056616572	0.127	0.13	1
0.500587172	-0.056514772	0.23	0.228	1
8.04811E-05	-0.056343135	0.093	0.104	1
0.003398055	-0.056309062	0.19	0.2	1
0.01282565	-0.056224729	0.304	0.309	1
0.119555275	-0.056214506	0.293	0.293	1
0.034023516	-0.056174844	0.152	0.157	1
0.039509804	-0.056025046	0.222	0.226	1
0.01788471	-0.055870241	0.378	0.381	1
0.119021379	-0.055652782	0.121	0.124	1
0.068203706	-0.055612825	0.13	0.134	1
0.000237957	-0.055569904	0.278	0.29	1
0.319183757	-0.055567876	0.319	0.318	1
0.001813147	-0.055473043	0.604	0.605	1
0.050885675	-0.055205782	0.179	0.184	1
0.377370423	-0.05518715	0.399	0.39	1
0.081578769	-0.055170111	0.2	0.205	1
0.526538156	-0.055164834	0.293	0.289	1
0.015304156	-0.055159587	0.254	0.259	1
0.021519298	-0.055096037	0.281	0.286	1
0.037414228	-0.055004724	0.288	0.291	1
0.045727603	-0.054989328	0.139	0.145	1
0.000313168	-0.054839552	0.135	0.145	1
0.029330001	-0.05481561	0.431	0.433	1
0.076200165	-0.054812945	0.758	0.763	1
0.044390139	-0.054745512	0.118	0.122	1
0.271958777	-0.054482657	0.252	0.251	1
0.002444325	-0.054364818	0.137	0.146	1
0.103061005	-0.054350178	0.198	0.201	1
0.307969483	-0.054256252	0.254	0.255	1
0.047977805	-0.054141062	0.115	0.12	1
0.000126317	-0.054080814	0.15	0.162	1
0.35679781	-0.053903014	0.42	0.418	1
0.005276214	-0.053742601	0.105	0.112	1
0.17846783	-0.053702464	0.239	0.24	1
0.205056876	-0.053675057	0.162	0.166	1
0.034264738	-0.053610955	0.137	0.142	1
0.683728826	-0.053444494	0.237	0.234	1
0.850533663	-0.053394439	0.232	0.226	1
0.048025115	-0.053351367	0.321	0.322	1
0.003583627	-0.05326098	0.149	0.158	1

0.648007755	-0.053112426	0.18	0.178	1
0.041739354	-0.052996196	0.245	0.249	1
0.000357074	-0.052947212	0.108	0.118	1
0.187653375	-0.052928395	0.114	0.117	1
0.035510063	-0.052812436	0.616	0.608	1
0.357138357	-0.052754123	0.133	0.133	1
0.005951767	-0.052751092	0.388	0.393	1
0.175442706	-0.05274054	0.179	0.182	1
0.001409039	-0.052721455	0.542	0.543	1
0.094952943	-0.052703591	0.246	0.248	1
0.007403502	-0.052686088	0.134	0.142	1
0.022076957	-0.052631825	0.119	0.125	1
0.007053634	-0.052620162	0.286	0.294	1
0.050797765	-0.052544846	0.322	0.325	1
0.15824173	-0.052502229	0.198	0.201	1
0.524965876	-0.052480057	0.223	0.221	1
0.005734759	-0.052460643	0.136	0.143	1
0.022221501	-0.05234742	0.158	0.164	1
0.179312319	-0.05228896	0.121	0.124	1
0.228215664	-0.05220789	0.187	0.188	1
0.281853915	-0.052206859	0.329	0.326	1
0.017850643	-0.052175672	0.216	0.222	1
0.100334394	-0.052171938	0.221	0.223	1
0.00220382	-0.052163621	0.311	0.32	1
0.130600127	-0.052103578	0.278	0.28	1
0.028427779	-0.052051249	0.401	0.401	1
0.010941301	-0.051685186	0.1	0.107	1
0.00060296	-0.051656327	0.477	0.481	1
0.280968539	-0.051640582	0.231	0.231	1
0.42848469	-0.051632946	0.304	0.301	1
0.295927006	-0.051540083	0.225	0.225	1
0.330157553	-0.051529968	0.169	0.17	1
0.000255894	-0.051429455	0.551	0.561	1
0.002774585	-0.051417446	0.316	0.324	1
0.004123934	-0.051366581	0.152	0.16	1
0.074320699	-0.051361994	0.174	0.178	1
0.010551539	-0.051254006	0.124	0.13	1
0.006495737	-0.051107155	0.099	0.106	1
0.090385532	-0.051017505	0.361	0.36	1
0.003321346	-0.050960136	0.116	0.124	1
0.006564566	-0.050920094	0.1	0.107	1
0.124009358	-0.050878141	0.207	0.209	1
0.000638961	-0.05081142	0.245	0.256	1
0.491000256	-0.05074492	0.197	0.191	1
0.009034441	-0.050694736	0.137	0.144	1
4.5456E-05	-0.050500444	0.366	0.379	1
0.032056085	-0.050394644	0.244	0.248	1
0.002399148	-0.05033563	0.544	0.504	1
9.5432E-05	-0.050282075	0.123	0.135	1
0.66161707	-0.050195143	0.129	0.126	1

0.120435274	-0.050170445	0.155	0.158	1
0.01434398	-0.050057594	0.41	0.413	1
0.029142746	-0.050032538	0.271	0.276	1
0.027572343	-0.049998405	0.321	0.325	1
0.002572065	-0.049940966	0.157	0.166	1
0.001574556	-0.049928792	0.14	0.15	1
0.001761728	-0.049883531	0.325	0.333	1
0.291055468	-0.049790693	0.371	0.366	1
0.167530679	-0.049628569	0.282	0.283	1
0.014307324	-0.049620823	0.181	0.188	1
0.000226173	-0.049585487	0.135	0.147	1
0.013971034	-0.049553604	0.132	0.139	1
0.099832897	-0.049521113	0.163	0.166	1
0.046202396	-0.049493166	0.114	0.119	1
0.012158201	-0.049491562	0.279	0.285	1
0.535876407	-0.049445211	0.115	0.115	1
0.027874719	-0.049409597	0.295	0.3	1
0.081037454	-0.049366175	0.101	0.105	1
0.898736522	-0.049324474	0.147	0.145	1
0.371911658	-0.049307325	0.147	0.148	1
0.024486517	-0.049122786	0.121	0.127	1
0.587919049	-0.049062926	0.152	0.152	1
0.004822299	-0.049047357	0.435	0.439	1
0.112502251	-0.048992966	0.096	0.1	1
0.052669029	-0.048953319	0.107	0.111	1
0.091156774	-0.048908465	0.366	0.366	1
0.682704682	-0.048830912	0.165	0.164	1
0.00078121	-0.048798918	0.415	0.425	1
0.034647498	-0.048797228	0.152	0.157	1
0.955111181	-0.048769085	0.151	0.148	1
0.002668977	-0.048695264	0.653	0.657	1
0.627347798	-0.048596683	0.336	0.328	1
0.045447217	-0.048586291	0.226	0.23	1
0.012090645	-0.048584267	0.307	0.313	1
0.16708695	-0.048488088	0.117	0.12	1
0.150099607	-0.048222545	0.226	0.228	1
0.55435798	-0.048046231	0.242	0.239	1
0.165524333	-0.048026028	0.325	0.324	1
0.111897899	-0.047982903	0.207	0.21	1
0.705265406	-0.047961711	0.239	0.235	1
0.530746756	-0.047905869	0.202	0.194	1
0.526628144	-0.047823451	0.292	0.289	1
0.00905942	-0.047807089	0.297	0.303	1
0.001266555	-0.047668572	0.185	0.195	1
0.142137467	-0.047646881	0.214	0.216	1
0.243386311	-0.047643714	0.313	0.313	1
0.044407813	-0.047637586	0.16	0.165	1
0.810395391	-0.047608817	0.16	0.159	1
0.10753505	-0.047598826	0.163	0.166	1
0.273459095	-0.047471855	0.196	0.198	1

0.240034114	-0.047386228	0.135	0.137	1
0.032096094	-0.047383637	0.125	0.131	1
0.586263323	-0.047354934	0.105	0.105	1
0.125885819	-0.047244518	0.127	0.13	1
0.258056863	-0.04723396	0.112	0.115	1
0.021416326	-0.047167849	0.321	0.325	1
0.344412693	-0.047137205	0.437	0.427	1
0.028808507	-0.047106829	0.283	0.284	1
0.065770861	-0.047041657	0.275	0.278	1
0.324460948	-0.04699206	0.262	0.261	1
0.006875958	-0.04698902	0.601	0.601	1
0.025326048	-0.046968637	0.21	0.215	1
0.250148765	-0.046962578	0.325	0.322	1
0.132490796	-0.046961858	0.282	0.283	1
0.072901292	-0.046805415	0.164	0.168	1
0.582662335	-0.046754319	0.15	0.146	1
0.023776636	-0.046739628	0.381	0.387	1
0.243915152	-0.046579741	0.171	0.173	1
0.002176683	-0.046549663	0.742	0.735	1
0.074081152	-0.046526442	0.253	0.256	1
0.06826298	-0.046442514	0.117	0.121	1
0.32836133	-0.046391091	0.24	0.24	1
0.37525624	-0.046384075	0.154	0.155	1
0.000815586	-0.046375192	0.151	0.161	1
0.223846927	-0.046331856	0.224	0.225	1
0.140710119	-0.046301709	0.143	0.146	1
0.099758811	-0.046261188	0.126	0.13	1
0.631973021	-0.046169875	0.196	0.195	1
0.447400121	-0.04591432	0.27	0.267	1
0.513771863	-0.045906637	0.199	0.198	1
0.406773098	-0.045891195	0.166	0.166	1
0.558109415	-0.045858194	0.311	0.306	1
0.342678405	-0.04578535	0.221	0.221	1
0.380113753	-0.045771488	0.205	0.205	1
0.188167384	-0.045687741	0.131	0.133	1
0.823678976	-0.045647861	0.247	0.243	1
0.044205506	-0.045635412	0.108	0.113	1
0.013818294	-0.045573416	0.479	0.478	1
0.232632328	-0.045550772	0.158	0.16	1
0.002375776	-0.045454688	0.094	0.102	1
0.205973793	-0.045443679	0.399	0.394	1
0.00649649	-0.045427355	0.118	0.125	1
8.87324E-05	-0.045315684	0.486	0.496	1
0.029569527	-0.045314247	0.157	0.163	1
0.057614913	-0.045261477	0.102	0.106	1
0.012138273	-0.045242083	0.126	0.133	1
0.262570985	-0.045146683	0.162	0.164	1
0.020352445	-0.04512017	0.154	0.16	1
0.005451061	-0.045053792	0.223	0.231	1
0.248085318	-0.045002134	0.259	0.257	1

0.190068489	-0.044969777	0.105	0.108	1
0.200757824	-0.044880664	0.184	0.187	1
0.957403708	-0.044878812	0.232	0.227	1
0.019349152	-0.044772937	0.214	0.221	1
0.088437732	-0.044757729	0.124	0.128	1
0.000846317	-0.044692951	0.335	0.346	1
0.2779638	-0.044621988	0.335	0.333	1
0.119270346	-0.044537539	0.141	0.145	1
0.747713	-0.044488777	0.263	0.258	1
0.012195847	-0.044452349	0.448	0.45	1
0.163160027	-0.044416232	0.202	0.204	1
0.016201571	-0.044402695	0.397	0.399	1
0.242101271	-0.044392527	0.396	0.39	1
0.221031733	-0.044387459	0.169	0.172	1
0.022178965	-0.044332426	0.093	0.1	1
0.567641514	-0.044316727	0.263	0.259	1
0.148750851	-0.044268399	0.253	0.254	1
0.541913174	-0.044213093	0.116	0.116	1
0.50724443	-0.044200416	0.311	0.307	1
0.007683824	-0.044024213	0.17	0.177	1
0.648766386	-0.044015924	0.307	0.301	1
0.121231622	-0.043948449	0.179	0.182	1
0.689183778	-0.043877307	0.22	0.213	1
0.083541016	-0.043860314	0.19	0.195	1
0.019909751	-0.043849489	0.162	0.168	1
0.06938094	-0.043780405	0.153	0.159	1
0.606931806	-0.043769206	0.118	0.118	1
0.168994608	-0.043763672	0.383	0.379	1
0.091901201	-0.043742333	0.259	0.263	1
0.251436775	-0.043729448	0.234	0.234	1
0.101137371	-0.043687831	0.306	0.306	1
0.066048532	-0.043682655	0.151	0.156	1
0.003288929	-0.04364036	0.19	0.199	1
0.105347506	-0.043535972	0.125	0.129	1
0.200604519	-0.043535796	0.126	0.128	1
0.506110921	-0.043486157	0.107	0.108	1
0.150203805	-0.043349598	0.504	0.499	1
0.039013846	-0.043346472	0.159	0.165	1
0.2309152	-0.043322258	0.216	0.217	1
0.000749301	-0.043307559	0.102	0.09	1
0.851342727	-0.043216198	0.162	0.16	1
0.060033489	-0.043083443	0.107	0.111	1
0.065886377	-0.042977225	0.106	0.111	1
0.172300732	-0.042939125	0.167	0.17	1
0.394809064	-0.042922631	0.132	0.133	1
0.277351343	-0.042875638	0.24	0.24	1
0.013351157	-0.042780549	0.279	0.285	1
0.07949554	-0.042731742	0.28	0.282	1
0.072616982	-0.042688901	0.138	0.143	1
0.844929069	-0.042686673	0.168	0.164	1

0.025058185	-0.042654695	0.135	0.141	1
0.173867329	-0.042574024	0.152	0.156	1
0.021806316	-0.042503225	0.212	0.218	1
0.023469166	-0.042485174	0.177	0.184	1
0.016995024	-0.042397344	0.104	0.111	1
0.695237457	-0.042289925	0.269	0.266	1
0.418337231	-0.042203681	0.182	0.182	1
0.039382771	-0.042189609	0.114	0.119	1
0.451320185	-0.042112543	0.14	0.141	1
0.631639946	-0.042047038	0.259	0.256	1
0.265753521	-0.042015644	0.106	0.108	1
0.533625855	-0.041983668	0.229	0.222	1
0.179105308	-0.041979132	0.111	0.114	1
0.714717086	-0.041816988	0.114	0.113	1
0.762500109	-0.04180481	0.147	0.146	1
0.005758258	-0.041794112	0.14	0.148	1
0.078120392	-0.041776042	0.196	0.2	1
0.123385001	-0.041738713	0.103	0.107	1
0.753604555	-0.041736633	0.167	0.163	1
0.468455603	-0.041654717	0.202	0.202	1
0.063497919	-0.041620647	0.128	0.133	1
0.075680961	-0.041593853	0.191	0.195	1
0.373801216	-0.041528614	0.402	0.395	1
0.058954065	-0.041477886	0.129	0.133	1
0.338583604	-0.041449915	0.177	0.178	1
0.724079708	-0.041431249	0.124	0.123	1
0.036808973	-0.041232035	0.122	0.127	1
0.19556907	-0.041217451	0.274	0.275	1
0.61520633	-0.041176698	0.185	0.184	1
0.530431946	-0.041170213	0.189	0.188	1
0.710604452	-0.041140143	0.195	0.193	1
0.221156425	-0.041129586	0.32	0.319	1
0.014404096	-0.041113691	0.38	0.358	1
0.018470011	-0.041088041	0.561	0.558	1
0.424656567	-0.04103841	0.104	0.105	1
0.008580286	-0.040985269	0.128	0.135	1
0.105017098	-0.040982098	0.152	0.156	1
0.676831566	-0.040857105	0.171	0.169	1
0.517833172	-0.040844253	0.655	0.645	1
0.752111272	-0.040828631	0.179	0.178	1
0.249273472	-0.040807412	0.193	0.194	1
0.217985007	-0.040782335	0.171	0.174	1
0.256736078	-0.040720433	0.131	0.134	1
0.006751388	-0.040650547	0.654	0.655	1
0.858583339	-0.040618249	0.133	0.132	1
0.828677838	-0.040617651	0.213	0.21	1
0.017757527	-0.040592404	0.21	0.217	1
0.152260251	-0.040470955	0.352	0.351	1
0.259515569	-0.040459605	0.215	0.216	1
0.182174025	-0.040432595	0.134	0.137	1

0.17748428	-0.040404318	0.176	0.178	1
0.7616103	-0.040321428	0.234	0.228	1
0.83477983	-0.040303603	0.224	0.22	1
0.33161537	-0.040283986	0.101	0.103	1
0.813743223	-0.040239644	0.167	0.166	1
0.034692892	-0.040204837	0.432	0.429	1
0.396727323	-0.040192212	0.212	0.212	1
0.027179587	-0.040192172	0.312	0.319	1
0.348400774	-0.040111942	0.17	0.17	1
0.088837717	-0.040110403	0.237	0.241	1
0.054432837	-0.03995265	0.173	0.178	1
0.763039284	-0.039922089	0.396	0.388	1
0.258126697	-0.039914603	0.342	0.341	1
0.551051068	-0.039872009	0.241	0.239	1
0.011795525	-0.039826525	0.619	0.615	1
0.889830045	-0.039801988	0.239	0.234	1
4.8288E-05	-0.039796593	0.12	0.133	1
0.431370331	-0.039767776	0.468	0.459	1
0.345784385	-0.039691752	0.182	0.183	1
0.125057085	-0.039620142	0.346	0.345	1
0.619788959	-0.039603727	0.101	0.101	1
0.776113805	-0.039513881	0.278	0.271	1
0.15381116	-0.039492589	0.131	0.134	1
0.917386766	-0.039466648	0.13	0.128	1
0.043076893	-0.039289184	0.392	0.393	1
0.062710251	-0.039288226	0.159	0.164	1
0.414476362	-0.039233606	0.126	0.127	1
0.978348436	-0.039233021	0.204	0.2	1
0.00886114	-0.039230964	0.1	0.107	1
0.645295418	-0.039209766	0.109	0.109	1
0.113984191	-0.039156149	0.273	0.275	1
0.830529987	-0.039153543	0.256	0.25	1
0.664701075	-0.039134071	0.108	0.108	1
0.938676414	-0.039124243	0.128	0.126	1
0.235851977	-0.039084717	0.136	0.139	1
0.30001115	-0.039055499	0.122	0.123	1
0.23417896	-0.039047453	0.101	0.103	1
0.907328548	-0.03902195	0.144	0.143	1
0.515985914	-0.039005134	0.193	0.193	1
0.888917068	-0.038995765	0.191	0.189	1
0.237394023	-0.038959265	0.312	0.312	1
0.381447098	-0.038927795	0.152	0.153	1
0.161656746	-0.038927339	0.097	0.101	1
0.062062559	-0.038783777	0.512	0.508	1
0.262433199	-0.038779832	0.134	0.136	1
0.526443366	-0.038767699	0.128	0.129	1
0.011241492	-0.038705751	0.14	0.147	1
0.004123113	-0.038671832	0.192	0.2	1
0.943612937	-0.038630929	0.132	0.13	1
0.566018124	-0.038619651	0.254	0.252	1

0.606898184	-0.038596518	0.201	0.2	1
0.798965364	-0.038523113	0.244	0.238	1
0.713927714	-0.03840322	0.244	0.241	1
0.005381073	-0.038391641	0.655	0.651	1
0.630629823	-0.0383764	0.113	0.113	1
0.781884771	-0.038355947	0.122	0.12	1
0.157937677	-0.038353766	0.133	0.136	1
0.637196818	-0.038337559	0.277	0.276	1
0.277799098	-0.038332883	0.149	0.151	1
0.049692163	-0.038323715	0.122	0.127	1
0.625111104	-0.038297221	0.198	0.196	1
0.118566474	-0.038228284	0.285	0.286	1
0.157161097	-0.038200668	0.151	0.154	1
0.62626452	-0.038176514	0.204	0.2	1
0.004781917	-0.038170898	0.133	0.142	1
0.69372076	-0.038155918	0.186	0.181	1
0.868763107	-0.038155633	0.281	0.276	1
0.017534657	-0.038137158	0.27	0.275	1
0.027821806	-0.038119003	0.196	0.202	1
0.413297789	-0.038104476	0.151	0.152	1
0.024025735	-0.038034537	0.103	0.109	1
0.976024568	-0.038019221	0.158	0.156	1
0.29496363	-0.03798264	0.58	0.572	1
0.002416929	-0.037846617	0.399	0.406	1
0.327431562	-0.037809025	0.161	0.155	1
0.507377956	-0.037769927	0.195	0.195	1
0.015424008	-0.037754719	0.645	0.641	1
0.293209209	-0.037752061	0.143	0.145	1
0.308068456	-0.03771942	0.124	0.126	1
0.607488849	-0.037712293	0.147	0.144	1
0.013486116	-0.037634803	0.177	0.185	1
0.138164058	-0.037599042	0.321	0.307	1
0.268224943	-0.037566471	0.167	0.161	1
0.693096095	-0.037529934	0.154	0.152	1
0.004201688	-0.03752147	0.562	0.567	1
0.032097241	-0.037499397	0.166	0.171	1
0.949904663	-0.03747439	0.245	0.24	1
0.442932802	-0.03744386	0.198	0.191	1
0.651822268	-0.037367087	0.257	0.25	1
0.106576143	-0.037355244	0.19	0.194	1
0.811451514	-0.037344218	0.39	0.38	1
0.032069759	-0.037326366	0.549	0.543	1
0.180291771	-0.037325688	0.107	0.11	1
0.097697636	-0.037315548	0.116	0.11	1
0.126159646	-0.0373147	0.124	0.128	1
0.965349305	-0.037283567	0.291	0.286	1
0.922912765	-0.03728314	0.146	0.144	1
0.025453074	-0.037279075	0.429	0.431	1
0.64758119	-0.037267129	0.158	0.158	1
0.57291605	-0.037234972	0.11	0.11	1

0.83259736	-0.037173821	0.106	0.106	1
0.202206091	-0.037173339	0.252	0.253	1
0.282430392	-0.037172089	0.257	0.258	1
0.129466617	-0.037159516	0.113	0.116	1
0.721239738	-0.03711835	0.162	0.161	1
0.083640163	-0.037103648	0.111	0.115	1
0.023349485	-0.037089439	0.319	0.324	1
0.064125077	-0.036907209	0.544	0.54	1
0.065028354	-0.036888676	0.264	0.267	1
0.504296168	-0.036853458	0.187	0.187	1
0.173811829	-0.036758018	0.22	0.223	1
0.224335499	-0.036730187	0.181	0.185	1
0.040804354	-0.036706432	0.133	0.139	1
0.206399537	-0.0366937	0.412	0.405	1
0.390493936	-0.03668825	0.106	0.102	1
0.068715623	-0.036658483	0.135	0.14	1
0.668088901	-0.036611762	0.407	0.398	1
0.618922264	-0.036598513	0.203	0.202	1
0.01518273	-0.036579348	0.339	0.346	1
0.551780194	-0.036502515	0.268	0.266	1
0.125764564	-0.036501424	0.195	0.198	1
0.019465258	-0.036494301	0.639	0.632	1
0.872855279	-0.036443301	0.281	0.276	1
0.448887147	-0.036399905	0.103	0.104	1
0.833253371	-0.036381275	0.157	0.154	1
0.857072646	-0.036358063	0.136	0.134	1
0.820546632	-0.036345519	0.149	0.148	1
0.592477955	-0.036342151	0.306	0.302	1
0.191072589	-0.036309565	0.11	0.113	1
0.591322521	-0.036306787	0.154	0.154	1
0.431627746	-0.036292487	0.263	0.261	1
0.078221373	-0.036266571	0.271	0.272	1
0.625297399	-0.036243547	0.173	0.172	1
0.571181697	-0.036170283	0.103	0.103	1
0.8354198	-0.036130996	0.199	0.197	1
0.003685958	-0.036062847	0.125	0.135	1
0.309003385	-0.036040438	0.292	0.28	1
0.002680189	-0.035988259	0.097	0.105	1
0.006783361	-0.035964062	0.543	0.541	1
0.06669047	-0.035939882	0.417	0.421	1
0.103095437	-0.035939749	0.236	0.239	1
0.82579965	-0.035914478	0.195	0.191	1
0.027552316	-0.035835795	0.094	0.1	1
0.542461885	-0.035801598	0.255	0.247	1
0.000851444	-0.035788815	0.144	0.155	1
0.310664943	-0.035753291	0.179	0.181	1
0.020448402	-0.035730097	0.8	0.799	1
0.286357452	-0.035678427	0.133	0.135	1
0.436156064	-0.03565446	0.156	0.151	1
0.612596044	-0.035644751	0.214	0.213	1

0.043025388	-0.035643379	0.104	0.109	1
0.334693249	-0.035642273	0.116	0.117	1
0.092041055	-0.035573881	0.232	0.236	1
0.982160332	-0.035531522	0.337	0.33	1
0.617213699	-0.035529347	0.223	0.217	1
0.004947855	-0.035520729	0.109	0.117	1
0.965127855	-0.035515161	0.181	0.178	1
0.272066131	-0.035509775	0.434	0.43	1
0.071925662	-0.035451316	0.112	0.117	1
0.943181966	-0.035438125	0.389	0.38	1
0.056656682	-0.035385874	0.227	0.232	1
0.884288224	-0.035343136	0.273	0.268	1
0.734614694	-0.035303435	0.218	0.216	1
0.060836168	-0.03523294	0.163	0.168	1
0.060866022	-0.035229237	0.708	0.704	1
0.606170189	-0.035133038	0.359	0.355	1
0.493964503	-0.035096392	0.161	0.162	1
0.363729773	-0.035008349	0.215	0.215	1
0.331417498	-0.034983591	0.122	0.124	1
0.619530368	-0.034953544	0.266	0.263	1
0.385594908	-0.034930268	0.311	0.308	1
0.354024999	-0.034872486	0.349	0.346	1
0.962560255	-0.034839373	0.249	0.244	1
0.354407761	-0.034773374	0.245	0.236	1
0.505346601	-0.034767097	0.187	0.186	1
0.265320898	-0.034750885	0.308	0.296	1
0.352311422	-0.034749111	0.283	0.273	1
0.430799913	-0.034651425	0.182	0.182	1
0.504736369	-0.034632713	0.128	0.129	1
0.56494102	-0.034599352	0.133	0.133	1
0.143416364	-0.03459864	0.144	0.148	1
0.667699038	-0.034564019	0.13	0.13	1
0.078690631	-0.03455515	0.29	0.293	1
0.925348256	-0.034541644	0.295	0.288	1
0.087890016	-0.034537309	0.598	0.585	1
0.069951835	-0.034499035	0.36	0.361	1
0.874889138	-0.03449545	0.22	0.216	1
0.243111385	-0.034491529	0.108	0.111	1
0.098108886	-0.034487482	0.249	0.251	1
0.144620281	-0.034435555	0.912	0.9	1
0.051917177	-0.034426606	0.206	0.212	1
0.048207538	-0.034387543	0.633	0.627	1
0.025322282	-0.034309269	0.182	0.189	1
0.106847297	-0.034279322	0.107	0.11	1
0.986922684	-0.034259974	0.283	0.277	1
0.930230806	-0.034167691	0.286	0.281	1
0.313807284	-0.03412602	0.225	0.225	1
0.158415011	-0.034026954	0.098	0.102	1
0.625748443	-0.034003473	0.188	0.183	1
0.6784292	-0.033974565	0.115	0.115	1

0.031052474	-0.03396795	0.105	0.111	1
0.494622292	-0.033964971	0.203	0.202	1
0.504090006	-0.033942436	0.249	0.247	1
0.503074109	-0.033932988	0.153	0.154	1
0.679987604	-0.033896146	0.149	0.149	1
0.026537256	-0.033857286	0.406	0.409	1
0.225104922	-0.033830684	0.154	0.156	1
0.668711549	-0.033828206	0.134	0.131	1
0.973859719	-0.033814691	0.305	0.298	1
0.953829994	-0.033799003	0.164	0.161	1
0.031370387	-0.033786232	0.154	0.16	1
0.188930482	-0.033775408	0.366	0.364	1
0.532476996	-0.033735749	0.226	0.226	1
0.916617788	-0.033698342	0.334	0.324	1
0.000270285	-0.033692911	0.634	0.636	1
0.721288795	-0.033692028	0.204	0.202	1
0.451645988	-0.033689261	0.236	0.228	1
0.175718837	-0.033654167	0.182	0.185	1
0.359088286	-0.033651013	0.134	0.136	1
0.017110328	-0.033621451	0.625	0.624	1
0.165310556	-0.033571775	0.116	0.119	1
0.931830507	-0.033559544	0.1	0.099	1
0.044626458	-0.033516803	0.112	0.117	1
0.123489279	-0.03350472	0.673	0.663	1
0.953479034	-0.033479568	0.159	0.156	1
0.496567673	-0.033450476	0.189	0.184	1
0.91908918	-0.033405616	0.304	0.297	1
0.319889282	-0.03337474	0.158	0.159	1
0.568909219	-0.033356389	0.261	0.259	1
0.214381752	-0.033336487	0.113	0.116	1
0.906206105	-0.033221222	0.112	0.111	1
0.917609453	-0.033183705	0.204	0.201	1
0.038685981	-0.033139153	0.287	0.273	1
0.767921037	-0.033138562	0.201	0.199	1
0.058468628	-0.033116784	0.237	0.241	1
0.018545803	-0.033045643	0.193	0.2	1
0.00658276	-0.033012759	0.146	0.154	1
0.163448433	-0.032991917	0.26	0.262	1
0.003379866	-0.032978176	0.239	0.25	1
0.871426282	-0.032937538	0.201	0.199	1
0.68979692	-0.032919497	0.133	0.132	1
0.34710096	-0.032909827	0.174	0.175	1
0.113262646	-0.032848185	0.162	0.165	1
0.408613441	-0.032847994	0.215	0.215	1
0.98564454	-0.032821863	0.192	0.188	1
0.078179935	-0.032796441	0.15	0.155	1
0.20553429	-0.032784691	0.232	0.234	1
0.054266034	-0.032783331	0.165	0.171	1
0.001779174	-0.032754909	0.695	0.661	1
0.882842273	-0.032727423	0.108	0.107	1

0.691352198	-0.032699131	0.265	0.262	1
0.225706705	-0.032686111	0.75	0.746	1
0.453761275	-0.032642551	0.413	0.406	1
0.801592103	-0.032624447	0.3	0.292	1
0.018726288	-0.032619519	0.176	0.183	1
0.032884778	-0.032580176	0.679	0.675	1
0.597941264	-0.032483911	0.16	0.156	1
0.534261406	-0.032423932	0.132	0.128	1
0.037015768	-0.032397521	0.387	0.389	1
0.171488215	-0.032372569	0.379	0.378	1
0.530781086	-0.03236088	0.266	0.258	1
0.306428056	-0.032305471	0.172	0.175	1
0.04084465	-0.032291993	0.202	0.208	1
0.212954841	-0.032291731	0.546	0.539	1
0.121862696	-0.032259733	0.266	0.267	1
0.887731532	-0.032242262	0.181	0.178	1
0.953719996	-0.032233904	0.112	0.111	1
0.911503104	-0.032199369	0.189	0.186	1
0.975466995	-0.032195897	0.153	0.151	1
0.879846661	-0.032150871	0.161	0.158	1
0.013336924	-0.032137899	0.281	0.289	1
0.977819637	-0.032137526	0.136	0.134	1
0.646944246	-0.032135181	0.22	0.214	1
0.768818947	-0.032054926	0.306	0.301	1
0.369269757	-0.032048198	0.098	0.1	1
0.510071499	-0.032020345	0.118	0.119	1
0.716018258	-0.03195842	0.345	0.335	1
0.027625872	-0.031847209	0.162	0.169	1
0.424591903	-0.031845679	0.169	0.163	1
0.056170011	-0.031780899	0.133	0.138	1
0.0555435	-0.031765822	0.13	0.135	1
0.940348022	-0.031757276	0.289	0.284	1
0.704216011	-0.031755611	0.18	0.179	1
0.689775651	-0.031693604	0.52	0.511	1
0.775608514	-0.03165397	0.119	0.119	1
0.43126613	-0.03163968	0.101	0.103	1
0.512231422	-0.031612608	0.272	0.269	1
0.594803435	-0.031594427	0.273	0.265	1
0.048827754	-0.031589079	0.227	0.232	1
0.146223092	-0.031565533	0.115	0.118	1
0.360536138	-0.031456731	0.446	0.441	1
0.251391609	-0.031364944	0.199	0.201	1
0.759582941	-0.031339023	0.388	0.379	1
0.154528924	-0.031337237	0.109	0.113	1
0.941227758	-0.031313387	0.169	0.167	1
0.871219906	-0.031257525	0.296	0.289	1
0.239124283	-0.031257454	0.192	0.194	1
0.510939823	-0.031228012	0.311	0.302	1
0.918287772	-0.031188759	0.118	0.118	1
0.454911344	-0.031171048	0.149	0.144	1

0.788193607	-0.031132822	0.144	0.143	1
0.722592651	-0.031120354	0.161	0.158	1
0.092881077	-0.031117177	0.098	0.102	1
0.897248279	-0.031108414	0.195	0.192	1
0.547570607	-0.031099364	0.131	0.127	1
0.384587852	-0.03107817	0.269	0.268	1
0.611059344	-0.031037684	0.431	0.429	1
0.273166315	-0.031030981	0.11	0.112	1
0.322295009	-0.0310005	0.314	0.315	1
0.327657013	-0.030993968	0.149	0.151	1
0.027226653	-0.030965767	0.138	0.144	1
0.798654519	-0.030935647	0.285	0.277	1
0.114846979	-0.030926917	0.118	0.122	1
0.962264265	-0.030815259	0.137	0.136	1
0.436957416	-0.03081187	0.227	0.219	1
0.488132802	-0.030800365	0.194	0.195	1
0.28450568	-0.030751166	0.245	0.235	1
0.891251769	-0.030696964	0.115	0.113	1
0.258688122	-0.030680229	0.152	0.154	1
0.767027684	-0.030595373	0.187	0.183	1
0.858792278	-0.030513057	0.123	0.122	1
0.777196924	-0.030493206	0.163	0.159	1
0.7176784	-0.03048779	0.156	0.155	1
0.842800775	-0.030462528	0.224	0.219	1
0.223812081	-0.030427418	0.152	0.155	1
0.789246061	-0.030422767	0.185	0.181	1
0.799676771	-0.030392183	0.505	0.493	1
0.417047051	-0.030356808	0.272	0.263	1
0.274965051	-0.030334827	0.228	0.219	1
0.386210224	-0.030330283	0.196	0.196	1
0.077059067	-0.030306007	0.141	0.146	1
0.865827041	-0.030300972	0.116	0.116	1
0.006595024	-0.030300499	0.211	0.22	1
0.434092375	-0.030274831	0.194	0.194	1
0.200729014	-0.030188477	0.154	0.147	1
0.448229676	-0.030162188	0.176	0.176	1
0.666927932	-0.030140026	0.134	0.133	1
0.783312886	-0.030138945	0.35	0.339	1
0.03846639	-0.030132906	0.164	0.171	1
0.07142839	-0.030086919	0.196	0.2	1
0.90687796	-0.030086123	0.157	0.154	1
0.471385656	-0.030078594	0.278	0.272	1
0.145597385	-0.030042734	0.212	0.202	1
0.233107453	-0.030018234	0.269	0.271	1
0.59099918	-0.029979537	0.326	0.321	1
0.251427744	-0.029968069	0.248	0.248	1
0.276142794	-0.029963694	0.233	0.224	1
0.267563867	-0.02983349	0.181	0.183	1
0.706586626	-0.029824573	0.417	0.411	1
0.463254789	-0.029815684	0.218	0.217	1

0.652659907	-0.029807148	0.153	0.152	1
0.182099183	-0.029785438	0.101	0.104	1
0.536018701	-0.029749797	0.122	0.123	1
0.125219174	-0.02971559	0.129	0.122	1
0.972799	-0.029670411	0.344	0.338	1
0.988491237	-0.029567629	0.297	0.291	1
0.904709734	-0.029506855	0.168	0.165	1
0.263045682	-0.029438522	0.276	0.276	1
0.027517399	-0.029430646	0.496	0.494	1
0.918312126	-0.029390121	0.135	0.134	1
0.723120971	-0.029373593	0.194	0.193	1
0.702574054	-0.029352867	0.108	0.105	1
0.753800329	-0.029301172	0.219	0.217	1
0.00412736	-0.029277335	0.411	0.422	1
0.896421171	-0.029275365	0.146	0.144	1
0.225992151	-0.029249682	0.102	0.105	1
0.172817493	-0.029239638	0.337	0.336	1
0.662221081	-0.029235289	0.12	0.117	1
0.878922033	-0.029228811	0.122	0.12	1
0.945492685	-0.029202801	0.161	0.159	1
0.683257598	-0.029197228	0.204	0.203	1
0.201663932	-0.029126991	0.136	0.129	1
0.776482558	-0.029123845	0.21	0.206	1
0.879904486	-0.029102514	0.212	0.208	1
0.017549298	-0.02909329	0.273	0.28	1
0.77558931	-0.029080723	0.14	0.139	1
0.572950812	-0.029047193	0.145	0.145	1
0.384527472	-0.029025305	0.124	0.126	1
0.077194278	-0.029007809	0.207	0.211	1
0.986932783	-0.028864884	0.107	0.106	1
0.497213133	-0.028849545	0.291	0.282	1
0.478542461	-0.028843943	0.105	0.106	1
0.77614956	-0.028841391	0.157	0.156	1
0.938947828	-0.028813149	0.15	0.149	1
0.493533803	-0.028775894	0.215	0.209	1
0.010828363	-0.028688564	0.322	0.331	1
0.189436873	-0.028649779	0.496	0.488	1
0.688176491	-0.028646882	0.157	0.153	1
0.679371369	-0.028612119	0.132	0.129	1
0.649241009	-0.028589721	0.286	0.278	1
0.502343333	-0.028581369	0.18	0.174	1
0.129127717	-0.02858119	0.385	0.369	1
0.410574259	-0.028554465	0.209	0.209	1
0.893719371	-0.028529002	0.136	0.135	1
0.638417949	-0.028516712	0.145	0.142	1
0.545620514	-0.028512744	0.406	0.399	1
0.588553133	-0.028500506	0.1	0.101	1
0.522018554	-0.028483352	0.165	0.16	1
0.600624529	-0.028455204	0.293	0.289	1
0.023135396	-0.028444401	0.135	0.142	1

0.599162793	-0.028426433	0.109	0.11	1
0.42109415	-0.028409649	0.195	0.189	1
0.406114617	-0.0284038	0.781	0.778	1
0.88957398	-0.028397459	0.139	0.137	1
0.067981209	-0.028388608	0.403	0.404	1
0.571732504	-0.028376936	0.187	0.182	1
0.403040804	-0.028359669	0.228	0.228	1
0.959317408	-0.028327086	0.141	0.139	1
0.531533855	-0.028323797	0.115	0.115	1
0.704839018	-0.028261261	0.134	0.131	1
0.533507442	-0.028225428	0.344	0.334	1
0.001662273	-0.028191503	0.226	0.236	1
0.196498451	-0.028161122	0.452	0.448	1
0.720755907	-0.028137139	0.118	0.116	1
0.520760116	-0.028113467	0.28	0.273	1
0.952018775	-0.028076764	0.143	0.141	1
0.559480491	-0.028074772	0.388	0.38	1
0.521044783	-0.028054048	0.234	0.234	1
0.872584338	-0.028041497	0.208	0.205	1
0.811817843	-0.028015074	0.195	0.193	1
0.130438192	-0.028012384	0.119	0.122	1
0.545206177	-0.028009858	0.126	0.127	1
0.451805016	-0.02800204	0.109	0.106	1
0.581975535	-0.027999443	0.157	0.153	1
0.248587136	-0.027964516	0.292	0.284	1
0.497204522	-0.027958086	0.155	0.156	1
0.909017245	-0.027945483	0.841	0.827	1
0.411354455	-0.027915126	0.145	0.146	1
0.302633658	-0.027885592	0.265	0.264	1
0.012806918	-0.027876239	0.364	0.345	1
0.685176791	-0.027864454	0.179	0.175	1
0.293684007	-0.027778998	0.162	0.163	1
0.56683088	-0.027739457	0.241	0.234	1
0.784999353	-0.02771377	0.113	0.113	1
0.797367522	-0.027676109	0.413	0.399	1
0.773810294	-0.027616229	0.118	0.118	1
0.871915913	-0.027584761	0.116	0.116	1
0.489911236	-0.027546973	0.585	0.575	1
0.234391084	-0.027424546	0.133	0.127	1
0.427338067	-0.027377587	0.272	0.27	1
0.584580775	-0.027370528	0.235	0.229	1
0.051849141	-0.027335508	0.15	0.141	1
0.845309235	-0.027334068	0.324	0.315	1
0.417575622	-0.027319149	0.215	0.216	1
0.785549195	-0.027286468	0.12	0.12	1
0.302303748	-0.027231234	0.137	0.139	1
0.505598096	-0.027201134	0.131	0.132	1
0.53896561	-0.027190669	0.436	0.423	1
0.217347242	-0.027156461	0.488	0.488	1
0.267225906	-0.027145559	0.354	0.341	1

0.13594615	-0.027134485	0.163	0.167	1
0.774311052	-0.02713034	0.412	0.403	1
0.340170407	-0.02710579	0.184	0.178	1
0.030547106	-0.027088229	0.114	0.12	1
0.4159787	-0.027077608	0.125	0.126	1
0.286680861	-0.027071697	0.168	0.17	1
0.420136884	-0.027067394	0.114	0.11	1
0.530524057	-0.027001177	0.181	0.176	1
0.536929259	-0.026861684	0.16	0.155	1
0.715211523	-0.026859726	0.333	0.324	1
0.743424317	-0.026840491	0.13	0.129	1
0.751490532	-0.026809931	0.173	0.172	1
0.854809115	-0.026807861	0.135	0.132	1
0.254002677	-0.026787116	0.478	0.473	1
0.77473655	-0.026716479	0.161	0.158	1
0.077204767	-0.026699454	0.165	0.17	1
0.52431819	-0.026673913	0.173	0.173	1
0.881603056	-0.026666739	0.119	0.117	1
0.313622622	-0.026524001	0.206	0.198	1
0.3611788	-0.026435811	0.207	0.21	1
0.439928017	-0.026433303	0.33	0.326	1
0.646005994	-0.026415591	0.173	0.172	1
0.595895291	-0.026387456	0.216	0.21	1
0.553721859	-0.026373856	0.153	0.149	1
0.512274613	-0.026371331	0.122	0.123	1
0.049917486	-0.02634201	0.193	0.198	1
0.007181194	-0.026284278	0.093	0.1	1
0.004485282	-0.02626613	0.111	0.12	1
0.521129246	-0.026242803	0.213	0.206	1
0.827609936	-0.026204006	0.292	0.287	1
0.399105693	-0.026195634	0.206	0.199	1
0.406937891	-0.026192911	0.292	0.283	1
0.128430668	-0.026174414	0.26	0.263	1
0.702791751	-0.026153274	0.189	0.184	1
0.33511062	-0.026135856	0.114	0.116	1
0.47093779	-0.026105127	0.181	0.182	1
0.187544012	-0.026098707	0.202	0.193	1
0.749269096	-0.02605053	0.107	0.107	1
0.480427369	-0.026029293	0.151	0.147	1
0.33729411	-0.026007926	0.513	0.499	1
0.730610702	-0.025997435	0.112	0.112	1
0.511568087	-0.025976115	0.107	0.108	1
0.455707134	-0.025852362	0.66	0.646	1
0.515997351	-0.025832623	0.344	0.334	1
0.010771559	-0.025750272	0.361	0.367	1
0.823939702	-0.025729043	0.127	0.125	1
0.850325382	-0.025684209	0.107	0.107	1
0.955019716	-0.025647143	0.122	0.12	1
0.625437599	-0.025628968	0.518	0.509	1
0.97218538	-0.025596518	0.146	0.144	1

0.817677035	-0.025546492	0.175	0.172	1
0.266785206	-0.025503346	0.22	0.211	1
0.497746776	-0.025500681	0.107	0.109	1
0.1223852	-0.025401484	0.236	0.225	1
0.661002467	-0.025134826	0.162	0.162	1
0.205617818	-0.025113696	0.135	0.129	1
0.406999838	-0.025084236	0.31	0.299	1
0.092978966	-0.025081896	0.232	0.221	1
0.917749266	-0.025069525	0.131	0.13	1
0.707315367	-0.025065221	0.101	0.101	1
0.680300506	-0.025059423	0.168	0.164	1
0.917685578	-0.025045742	0.262	0.258	1
0.6855665	-0.025040824	0.118	0.118	1
0.004061847	-0.025007831	0.411	0.418	1
0.155714538	-0.024991136	0.312	0.299	1
0.926058572	-0.024976544	0.119	0.118	1
0.266560006	-0.024952318	0.243	0.234	1
0.307695777	-0.024920429	0.229	0.221	1
0.17763339	-0.024901776	0.152	0.145	1
0.932513277	-0.024886486	0.308	0.301	1
0.040433773	-0.024880402	0.2	0.189	1
0.296592727	-0.024875299	0.149	0.152	1
0.897385432	-0.024835103	0.459	0.453	1
0.242368835	-0.024779969	0.268	0.258	1
0.346726756	-0.02476089	0.236	0.228	1
0.556538657	-0.024727784	0.21	0.21	1
0.636283727	-0.024713554	0.208	0.208	1
0.148471715	-0.024669674	0.142	0.135	1
0.36541898	-0.024667152	0.238	0.23	1
0.247120969	-0.024613721	0.178	0.18	1
0.38777431	-0.02461083	0.142	0.137	1
0.120755693	-0.024610222	0.191	0.182	1
0.818596336	-0.024551621	0.129	0.127	1
0.793388721	-0.024549229	0.104	0.102	1
0.293922245	-0.024522005	0.16	0.162	1
0.700456095	-0.024431919	0.14	0.14	1
0.848078732	-0.024401349	0.305	0.301	1
0.578289195	-0.024358909	0.404	0.399	1
0.728091432	-0.024350607	0.215	0.211	1
0.39702722	-0.024345528	0.285	0.286	1
0.052029263	-0.024341848	0.231	0.237	1
0.813830327	-0.024307184	0.167	0.164	1
0.884019981	-0.024243589	0.211	0.207	1
0.986833295	-0.02422969	0.109	0.108	1
0.027762467	-0.02420694	0.192	0.198	1
0.845936766	-0.024186359	0.216	0.214	1
0.767173875	-0.02408121	0.162	0.16	1
0.002073121	-0.024058375	0.118	0.107	1
0.4174602	-0.02405073	0.23	0.23	1
0.519356118	-0.024045038	0.148	0.149	1

0.340450049	-0.024009308	0.294	0.293	1
0.261167827	-0.023906317	0.218	0.21	1
0.544987701	-0.023882021	0.144	0.14	1
0.846628605	-0.02387875	0.217	0.215	1
0.381828744	-0.023860414	0.567	0.556	1
0.940539848	-0.023769967	0.206	0.204	1
0.330714061	-0.023762999	0.146	0.148	1
0.319456367	-0.023723034	0.105	0.108	1
0.431205167	-0.023706582	0.335	0.323	1
0.872493372	-0.023700582	0.178	0.176	1
0.025355634	-0.023684858	0.333	0.339	1
0.548928404	-0.023662082	0.153	0.154	1
0.93553856	-0.023659969	0.137	0.136	1
0.575386232	-0.023612441	0.146	0.147	1
0.610137239	-0.023513918	0.193	0.189	1
0.881051609	-0.02347081	0.111	0.11	1
0.542082728	-0.023438891	0.118	0.119	1
0.955704993	-0.023369914	0.384	0.373	1
0.576663982	-0.023298831	0.33	0.32	1
0.60732427	-0.023294745	0.134	0.131	1
0.479661661	-0.023285438	0.188	0.183	1
0.028353376	-0.023262233	0.356	0.337	1
0.962344943	-0.02324193	0.255	0.25	1
0.397530823	-0.023201644	0.124	0.12	1
0.42994601	-0.02317264	0.278	0.27	1
0.636099409	-0.023166144	0.182	0.178	1
0.128830243	-0.023149628	0.212	0.203	1
0.099727687	-0.023139404	0.252	0.256	1
0.223785957	-0.023085992	0.111	0.106	1
0.7196836	-0.023011141	0.762	0.756	1
0.846068394	-0.022987522	0.15	0.147	1
0.243962361	-0.022958666	0.266	0.267	1
0.535540226	-0.022952269	0.199	0.193	1
0.999644568	-0.022950301	0.153	0.152	1
0.97546149	-0.022906105	0.146	0.144	1
0.575317191	-0.02288854	0.105	0.106	1
0.283813897	-0.022859743	0.164	0.158	1
0.695519657	-0.02285969	0.152	0.149	1
0.972676225	-0.022838744	0.176	0.174	1
0.623299883	-0.022836676	0.132	0.133	1
0.186839899	-0.022819981	0.166	0.159	1
0.928445337	-0.022800248	0.249	0.245	1
0.793067394	-0.022780617	0.141	0.141	1
0.03762342	-0.022771361	0.209	0.197	1
0.848675771	-0.02276367	0.217	0.213	1
0.796210585	-0.022751675	0.107	0.107	1
0.71835192	-0.022707222	0.118	0.116	1
0.761655005	-0.022688211	0.283	0.28	1
0.370776555	-0.022678161	0.156	0.151	1
0.011958433	-0.022582406	0.516	0.491	1

0.559456845	-0.022572283	0.202	0.201	1
0.646711978	-0.022566154	0.25	0.243	1
0.957406856	-0.022533896	0.141	0.14	1
0.131988756	-0.022527934	0.188	0.18	1
0.455294093	-0.022497307	0.115	0.116	1
0.320563183	-0.022452105	0.173	0.175	1
0.182392008	-0.022378822	0.291	0.28	1
0.01193723	-0.022376902	0.137	0.127	1
0.606082098	-0.02237046	0.123	0.12	1
0.278090218	-0.02234775	0.189	0.182	1
0.772830352	-0.022307613	0.345	0.337	1
0.017758757	-0.022297195	0.147	0.137	1
0.557254678	-0.022272867	0.107	0.107	1
0.62425587	-0.022268451	0.173	0.169	1
0.311525975	-0.02219177	0.134	0.136	1
0.28369255	-0.02217119	0.154	0.149	1
0.619595941	-0.022164849	0.1	0.098	1
0.487103671	-0.022160027	0.178	0.173	1
0.291995024	-0.022105749	0.159	0.153	1
0.447042233	-0.022073035	0.106	0.108	1
0.723577854	-0.02203458	0.533	0.525	1
0.753835024	-0.02196633	0.109	0.109	1
0.889633154	-0.021947141	0.136	0.135	1
0.088978652	-0.021928359	0.102	0.096	1
0.956721355	-0.021886628	0.358	0.352	1
0.806941907	-0.021862311	0.147	0.146	1
0.684230877	-0.021853859	0.116	0.116	1
0.113990019	-0.021842527	0.429	0.411	1
0.077793183	-0.021841946	0.511	0.508	1
0.777675467	-0.021832468	0.119	0.119	1
0.525515041	-0.021832104	0.195	0.19	1
0.224020141	-0.021779003	0.11	0.105	1
0.123395053	-0.02177432	0.216	0.206	1
0.496510485	-0.021738449	0.141	0.137	1
0.545165642	-0.021725109	0.135	0.137	1
0.797609537	-0.021699659	0.118	0.116	1
0.70615207	-0.021677691	0.114	0.112	1
0.834889929	-0.021640778	0.167	0.165	1
0.073388672	-0.021615338	0.143	0.135	1
0.412953263	-0.021526519	0.099	0.1	1
0.476583205	-0.021459312	0.153	0.148	1
0.996015772	-0.021455623	0.176	0.174	1
0.967982786	-0.021454194	0.347	0.339	1
0.969312043	-0.021442801	0.104	0.103	1
0.222258126	-0.021430681	0.168	0.161	1
0.884570059	-0.021425811	0.126	0.126	1
0.122403758	-0.021402049	0.119	0.124	1
0.26452785	-0.021389846	0.184	0.177	1
0.119449372	-0.021386565	0.114	0.118	1
0.133766952	-0.02134039	0.135	0.128	1

0.898398624	-0.021326745	0.123	0.121	1
0.555742131	-0.021313704	0.276	0.268	1
0.989371516	-0.021289307	0.114	0.113	1
0.219837255	-0.021287822	0.471	0.463	1
0.339067288	-0.02126984	0.187	0.19	1
0.416182838	-0.021249618	0.103	0.105	1
0.203230816	-0.021235496	0.185	0.178	1
0.946304441	-0.021223948	0.195	0.193	1
0.348769009	-0.021214514	0.13	0.126	1
0.493404076	-0.021209035	0.152	0.147	1
0.098535914	-0.021206077	0.115	0.109	1
0.482722518	-0.02117363	0.256	0.248	1
0.119817228	-0.021171134	0.158	0.15	1
0.626444678	-0.021159637	0.235	0.229	1
0.18398316	-0.021158244	0.191	0.184	1
0.989494066	-0.021157251	0.1	0.099	1
0.242649878	-0.021098329	0.3	0.289	1
0.848013106	-0.021082284	0.164	0.163	1
0.755355003	-0.021075922	0.178	0.177	1
0.347179774	-0.021017075	0.165	0.159	1
0.56967315	-0.021014582	0.105	0.103	1
0.88836095	-0.02099199	0.439	0.429	1
0.993256161	-0.020990789	0.145	0.144	1
0.526788144	-0.020977051	0.267	0.259	1
0.986225143	-0.02097457	0.513	0.499	1
0.594226297	-0.020965517	0.112	0.109	1
0.914992895	-0.020933146	0.165	0.163	1
0.148074855	-0.020927035	0.097	0.101	1
0.339062232	-0.0209182	0.195	0.196	1
0.547398189	-0.020908634	0.127	0.128	1
0.904869568	-0.020857839	0.123	0.122	1
0.177067447	-0.020848981	0.215	0.207	1
0.733345157	-0.020843899	0.111	0.111	1
0.966214209	-0.020774907	0.131	0.13	1
0.296038263	-0.020750895	0.146	0.148	1
0.591250887	-0.020692111	0.409	0.404	1
0.253999555	-0.020650344	0.291	0.279	1
0.014815217	-0.020647026	0.181	0.169	1
0.972749474	-0.020645615	0.207	0.204	1
0.357824998	-0.020619967	0.177	0.171	1
0.302679651	-0.020580124	0.111	0.114	1
0.3367456	-0.020568227	0.122	0.118	1
0.329361954	-0.020549985	0.266	0.257	1
0.216943503	-0.020548689	0.161	0.155	1
0.046267216	-0.020538386	0.104	0.097	1
0.557427346	-0.020536994	0.104	0.105	1
0.667229971	-0.020533392	0.118	0.116	1
0.291518085	-0.020495708	0.116	0.119	1
0.277590587	-0.020481834	0.16	0.154	1
0.091679956	-0.020437459	0.104	0.098	1

0.720656177	-0.020432575	0.274	0.271	1
0.097887501	-0.02042738	0.2	0.19	1
0.240919384	-0.020420349	0.663	0.642	1
0.818724894	-0.020402258	0.125	0.125	1
0.146119481	-0.020385144	0.165	0.158	1
0.907154081	-0.02037444	0.182	0.18	1
0.650917168	-0.020368993	0.216	0.215	1
0.74225913	-0.020358265	0.269	0.263	1
0.820936347	-0.020343063	0.137	0.137	1
5.35941E-05	-0.020329945	0.566	0.529	1
0.505976938	-0.020252579	0.33	0.32	1
0.821958726	-0.020243434	0.405	0.396	1
0.52367342	-0.020238863	0.103	0.104	1
0.643478245	-0.020197555	0.269	0.262	1
0.274650381	-0.020154617	0.184	0.177	1
0.032552249	-0.020151685	0.13	0.122	1
0.72116672	-0.020151681	0.12	0.12	1
0.893708223	-0.020137705	0.173	0.17	1
0.232160537	-0.020126132	0.107	0.102	1
0.808066563	-0.02011991	0.148	0.148	1
0.167735656	-0.020110754	0.131	0.135	1
0.211741959	-0.020039529	0.28	0.268	1
0.033562655	-0.020036164	0.145	0.136	1
0.628334684	-0.020031537	0.115	0.112	1
0.548879382	-0.019998131	0.138	0.135	1
0.741933931	-0.019949751	0.147	0.144	1
0.801665511	-0.019936871	0.267	0.263	1
0.410770899	-0.019923505	0.136	0.131	1
0.396975241	-0.019823467	0.315	0.305	1
0.811832844	-0.019808982	0.113	0.112	1
0.290820821	-0.019807994	0.174	0.167	1
0.34300629	-0.019771669	0.233	0.225	1
0.837230365	-0.019750608	0.27	0.264	1
0.793419979	-0.01971045	0.201	0.197	1
0.020853243	-0.019691121	0.161	0.151	1
0.146535317	-0.019685206	0.786	0.784	1
0.186184616	-0.01968006	0.315	0.302	1
0.350378517	-0.019642625	0.164	0.159	1
0.728315908	-0.019639625	0.174	0.173	1
0.023675293	-0.019592214	0.147	0.154	1
0.541269761	-0.019556686	0.113	0.11	1
0.049999677	-0.019553946	0.145	0.136	1
0.728473247	-0.019511523	0.273	0.267	1
0.325930399	-0.019498527	0.299	0.288	1
0.609192793	-0.01946764	0.164	0.161	1
0.333746666	-0.019462074	0.193	0.186	1
0.982380347	-0.019454644	0.16	0.158	1
0.584230614	-0.019449099	0.285	0.279	1
0.766013278	-0.019439827	0.415	0.409	1
0.036270314	-0.019425841	0.138	0.129	1

0.299305291	-0.019405957	0.315	0.304	1
0.615743721	-0.019390382	0.183	0.179	1
0.882714973	-0.019390009	0.121	0.121	1
0.726531416	-0.019376641	0.89	0.883	1
0.637000026	-0.019356475	0.1	0.098	1
0.912164509	-0.019330472	0.106	0.105	1
0.356074518	-0.019280398	0.239	0.239	1
0.984000663	-0.019178948	0.131	0.131	1
0.803024674	-0.019116313	0.486	0.471	1
0.997471836	-0.019109103	0.153	0.151	1
0.035053563	-0.019104427	0.442	0.445	1
0.119301378	-0.01906822	0.222	0.212	1
0.194338956	-0.019061819	0.215	0.206	1
0.294441449	-0.0190515	0.194	0.187	1
0.935267619	-0.019016528	0.102	0.101	1
0.181243191	-0.0190013	0.166	0.159	1
0.09356117	-0.018985251	0.304	0.29	1
0.133041242	-0.018894022	0.102	0.097	1
0.638065115	-0.018887342	0.294	0.286	1
0.046999871	-0.018885461	0.101	0.094	1
0.111645973	-0.018861831	0.126	0.13	1
0.598522287	-0.018855339	0.13	0.131	1
0.707394029	-0.018848003	0.381	0.371	1
0.283467726	-0.018840885	0.174	0.167	1
0.181790163	-0.018813433	0.123	0.117	1
0.969290619	-0.01881181	0.264	0.26	1
0.55845411	-0.018793755	0.1	0.101	1
0.905079364	-0.018781556	0.125	0.124	1
0.817455871	-0.018712099	0.137	0.137	1
0.508125492	-0.01870481	0.143	0.139	1
0.975432048	-0.01868899	0.101	0.1	1
0.307870253	-0.018688105	0.43	0.413	1
0.785209712	-0.018656453	0.426	0.416	1
0.203538081	-0.018655181	0.136	0.131	1
0.813743966	-0.018643119	0.142	0.139	1
0.529545279	-0.018642658	0.183	0.183	1
0.04867384	-0.018636731	0.151	0.157	1
0.344062267	-0.018601861	0.132	0.128	1
0.184407702	-0.018572023	0.229	0.219	1
0.506107972	-0.018538061	0.283	0.274	1
0.577141623	-0.018523735	0.318	0.314	1
0.868142266	-0.018522836	0.122	0.12	1
0.324598193	-0.018515134	0.117	0.113	1
0.064991951	-0.018471966	0.542	0.52	1
0.172858367	-0.018447996	0.215	0.206	1
0.811066126	-0.018391083	0.221	0.216	1
0.001294636	-0.018390294	0.115	0.103	1
0.143952177	-0.01835356	0.193	0.184	1
0.413328364	-0.018305179	0.102	0.103	1
0.378868232	-0.018289798	0.176	0.171	1

0.157037861	-0.018285406	0.322	0.309	1
0.493108308	-0.018265304	0.245	0.243	1
0.64586215	-0.01826443	0.279	0.273	1
0.45789604	-0.018247563	0.121	0.117	1
0.956166407	-0.018228768	0.215	0.214	1
0.834631871	-0.018217483	0.239	0.236	1
0.046534459	-0.018210028	0.188	0.178	1
0.994984642	-0.01820517	0.277	0.274	1
0.620664491	-0.018161458	0.138	0.135	1
0.102296432	-0.018151214	0.104	0.098	1
0.74325461	-0.018124636	0.242	0.238	1
0.812624696	-0.018061566	0.389	0.378	1
0.125876508	-0.018060042	0.212	0.203	1
0.321169794	-0.018001805	0.11	0.106	1
0.657694501	-0.017997062	0.157	0.153	1
0.293405179	-0.017953437	0.219	0.221	1
0.976702212	-0.01793515	0.447	0.438	1
0.075613847	-0.017896565	0.241	0.23	1
0.139034941	-0.017868073	0.113	0.108	1
0.7263553	-0.017844738	0.362	0.356	1
0.123810898	-0.017839474	0.228	0.232	1
0.387012332	-0.01783408	0.139	0.134	1
0.062134223	-0.017776897	0.1	0.094	1
0.444709172	-0.01774164	0.363	0.35	1
0.138122775	-0.017733119	0.221	0.211	1
0.144971432	-0.0177069	0.149	0.143	1
0.843169734	-0.017702662	0.102	0.102	1
0.837211689	-0.017681929	0.198	0.196	1
0.531919767	-0.017634301	0.11	0.107	1
0.55873457	-0.017572608	0.218	0.212	1
0.059018301	-0.017562171	0.226	0.214	1
0.863397259	-0.017552588	0.409	0.4	1
0.167511545	-0.017549601	0.119	0.114	1
0.021219342	-0.017525938	0.169	0.158	1
0.529924333	-0.017515184	0.338	0.328	1
0.099943179	-0.017475782	0.212	0.202	1
0.031268916	-0.017445636	0.194	0.183	1
0.468754527	-0.017432432	0.267	0.267	1
0.647069862	-0.017425248	0.159	0.157	1
0.049928295	-0.01741983	0.214	0.202	1
0.516137429	-0.017386182	0.118	0.12	1
0.148631408	-0.017361235	0.156	0.15	1
0.95040967	-0.017360915	0.329	0.322	1
0.785270524	-0.017332152	0.126	0.124	1
0.127524235	-0.017325678	0.107	0.102	1
0.028010885	-0.017311074	0.112	0.118	1
0.768814899	-0.017229585	0.204	0.2	1
0.540473887	-0.017226545	0.103	0.1	1
0.90409611	-0.017224856	0.169	0.167	1
0.229972096	-0.017153982	0.14	0.135	1

0.033658453	-0.017041176	0.331	0.315	1
0.353518164	-0.017031613	0.11	0.106	1
0.33123192	-0.017021396	0.184	0.178	1
0.069971797	-0.01700295	0.301	0.287	1
0.009615238	-0.01699471	0.125	0.115	1
0.728092977	-0.016988442	0.153	0.15	1
0.64604997	-0.016944155	0.115	0.113	1
0.815052702	-0.016942284	0.141	0.139	1
0.12993062	-0.016888417	0.167	0.16	1
0.485871021	-0.016864256	0.161	0.157	1
0.645188538	-0.01684671	0.196	0.191	1
0.113097311	-0.016804673	0.126	0.12	1
0.09630651	-0.016801361	0.144	0.136	1
0.225735215	-0.016775255	0.138	0.133	1
0.80979088	-0.016767876	0.182	0.182	1
0.21956787	-0.016767201	0.244	0.235	1
0.860361398	-0.016758372	0.121	0.12	1
0.570430367	-0.016738885	0.1	0.098	1
0.985036195	-0.016702009	0.127	0.126	1
0.04442545	-0.016699537	0.195	0.184	1
0.208384558	-0.016695236	0.141	0.135	1
0.235335506	-0.016658489	0.168	0.161	1
0.343921354	-0.016638122	0.367	0.367	1
0.641196559	-0.01650245	0.122	0.12	1
0.899517316	-0.016491811	0.161	0.159	1
0.208654337	-0.016485266	0.12	0.115	1
0.195114739	-0.016482286	0.187	0.18	1
0.421979339	-0.016462305	0.225	0.218	1
0.576228439	-0.016455403	0.164	0.16	1
0.955857914	-0.016411798	0.136	0.135	1
0.925746887	-0.01635121	0.214	0.212	1
0.015138865	-0.016311307	0.101	0.093	1
0.110435625	-0.016299197	0.248	0.237	1
0.228660337	-0.01629056	0.119	0.114	1
0.822523596	-0.01628539	0.102	0.101	1
0.652428557	-0.016263088	0.12	0.118	1
0.103209322	-0.016245557	0.693	0.666	1
0.374037928	-0.016225582	0.123	0.119	1
0.594461874	-0.016206177	0.127	0.128	1
0.830912693	-0.01619063	0.173	0.169	1
0.803439124	-0.016173432	0.189	0.187	1
0.149972786	-0.016120489	0.117	0.111	1
0.235569351	-0.016105533	0.202	0.194	1
0.073243307	-0.016092621	0.23	0.219	1
0.393398746	-0.01609062	0.171	0.166	1
0.260663756	-0.016017907	0.101	0.097	1
0.4294347	-0.0160129	0.274	0.268	1
0.854596354	-0.015991718	0.14	0.138	1
0.276604328	-0.01598818	0.189	0.183	1
0.552874093	-0.015984537	0.271	0.264	1

0.437198078	-0.01597059	0.249	0.241	1
0.936886638	-0.015954732	0.183	0.181	1
0.121862423	-0.01592674	0.235	0.225	1
0.26394829	-0.015893476	0.161	0.155	1
0.822939673	-0.015860559	0.277	0.274	1
0.390013394	-0.015836186	0.101	0.098	1
0.020768049	-0.015826511	0.171	0.16	1
0.598921039	-0.015819477	0.116	0.117	1
0.050375528	-0.015811565	0.148	0.139	1
0.446210837	-0.015804117	0.434	0.42	1
0.623700981	-0.015770768	0.204	0.2	1
0.065898351	-0.015760731	0.131	0.123	1
0.265205485	-0.015750611	0.135	0.13	1
0.481212823	-0.015737234	0.147	0.148	1
0.704425163	-0.015709113	0.102	0.1	1
0.799021089	-0.015673655	0.163	0.161	1
0.682217504	-0.01566189	0.328	0.319	1
0.041450609	-0.015649971	0.241	0.228	1
0.47686147	-0.015602259	0.149	0.145	1
0.178848391	-0.015583628	0.274	0.263	1
0.683875363	-0.015515209	0.104	0.102	1
0.034807441	-0.015494143	0.191	0.181	1
0.097958003	-0.015468573	0.254	0.242	1
0.334284595	-0.015456648	0.414	0.399	1
0.633981305	-0.015450305	0.114	0.111	1
0.411196878	-0.015401866	0.177	0.172	1
0.934551466	-0.0153725	0.166	0.165	1
0.208912529	-0.015362595	0.174	0.167	1
0.134382576	-0.015350098	0.425	0.407	1
0.2448466	-0.015339829	0.102	0.098	1
0.89487581	-0.015334287	0.178	0.176	1
0.181509266	-0.015328902	0.196	0.188	1
0.843554217	-0.015298711	0.12	0.119	1
0.443164395	-0.015288967	0.113	0.109	1
0.203200862	-0.015279207	0.245	0.235	1
0.106042258	-0.015249349	0.17	0.162	1
0.645607419	-0.015204405	0.099	0.1	1
0.154982401	-0.015202407	0.178	0.171	1
0.232188481	-0.015194078	0.145	0.139	1
0.029748281	-0.015183793	0.114	0.106	1
0.021611217	-0.015182485	0.24	0.226	1
0.446095095	-0.015136421	0.377	0.373	1
0.590238651	-0.015086779	0.232	0.231	1
0.238773486	-0.01504965	0.112	0.107	1
0.386255887	-0.015041996	0.295	0.287	1
0.017154966	-0.015018509	0.201	0.189	1
0.166990467	-0.015007785	0.107	0.102	1
0.1247953	-0.014979189	0.187	0.179	1
0.006650343	-0.014974582	0.167	0.155	1
0.261972348	-0.014971768	0.175	0.168	1

0.361307018	-0.014942694	0.245	0.238	1
0.898022762	-0.014940144	0.163	0.161	1
0.053517725	-0.014860411	0.172	0.163	1
0.125866147	-0.014825645	0.192	0.184	1
0.249783814	-0.014806478	0.11	0.105	1
0.095143421	-0.014762717	0.15	0.142	1
0.078404512	-0.014762098	0.156	0.148	1
0.147529595	-0.014743533	0.21	0.201	1
0.355030941	-0.014701618	0.241	0.232	1
0.213693399	-0.014692943	0.13	0.125	1
0.202077667	-0.0146913	0.352	0.338	1
0.07805487	-0.014677825	0.174	0.165	1
0.843158878	-0.014648373	0.119	0.117	1
0.011054047	-0.014606806	0.122	0.113	1
0.976195266	-0.014603975	0.162	0.161	1
0.295503049	-0.014582585	0.165	0.159	1
0.066432842	-0.014558652	0.161	0.153	1
0.100264853	-0.014539118	0.205	0.195	1
0.33695811	-0.014535499	0.162	0.157	1
0.067934819	-0.014533268	0.779	0.778	1
0.295057831	-0.014516208	0.204	0.198	1
0.963881885	-0.014488368	0.125	0.124	1
0.656395852	-0.014443003	0.236	0.235	1
0.334769476	-0.014417759	0.959	0.958	1
0.379411366	-0.01438346	0.1	0.102	1
0.112338983	-0.014382496	0.154	0.147	1
0.952903212	-0.014382355	0.169	0.167	1
0.838086197	-0.014374812	0.147	0.146	1
0.29931299	-0.014363709	0.163	0.158	1
0.971397571	-0.014344301	0.172	0.171	1
0.267583044	-0.014342162	0.126	0.121	1
0.646249513	-0.014319104	0.126	0.124	1
0.401074344	-0.014300806	0.126	0.122	1
0.457345698	-0.014295249	0.196	0.19	1
0.369552738	-0.014277308	0.153	0.149	1
0.807502931	-0.01426835	0.283	0.277	1
0.362133467	-0.014251677	0.271	0.262	1
0.210423011	-0.014238347	0.157	0.16	1
0.8026781	-0.01421553	0.128	0.126	1
0.308513362	-0.014207107	0.113	0.109	1
0.295838037	-0.014173139	0.144	0.139	1
0.170073148	-0.014166945	0.141	0.134	1
0.661643024	-0.014143912	0.211	0.206	1
0.240064743	-0.014140914	0.167	0.16	1
0.381222122	-0.014092652	0.101	0.097	1
0.048799843	-0.014080754	0.103	0.097	1
0.081447515	-0.014078434	0.293	0.279	1
0.079355447	-0.014045799	0.106	0.099	1
0.284270663	-0.014040983	0.155	0.15	1
0.030831345	-0.01402764	0.155	0.145	1

0.475254363	-0.013991671	0.194	0.189	1
0.675851447	-0.013976993	0.109	0.109	1
0.320513052	-0.013965447	0.12	0.116	1
0.657760319	-0.013964539	0.24	0.235	1
0.280473646	-0.013953217	0.232	0.224	1
0.659065099	-0.013918962	0.111	0.109	1
0.318838351	-0.013903311	0.128	0.123	1
0.139209721	-0.013851577	0.147	0.14	1
0.322912588	-0.013848362	0.119	0.115	1
0.956567006	-0.013847409	0.115	0.115	1
0.041787563	-0.013813845	0.201	0.191	1
0.167052807	-0.013813184	0.194	0.186	1
0.782316531	-0.013766669	0.131	0.131	1
0.012410021	-0.01375689	0.123	0.114	1
0.754924758	-0.013743696	0.139	0.136	1
0.75284273	-0.013715674	0.111	0.109	1
0.106633098	-0.013694653	0.139	0.132	1
0.290774399	-0.013684642	0.165	0.167	1
0.57728243	-0.013674981	0.182	0.183	1
0.0153696	-0.01364841	0.113	0.104	1
0.536393182	-0.013616683	0.129	0.125	1
0.933145443	-0.013607583	0.101	0.1	1
0.058532402	-0.013601398	0.172	0.162	1
0.963823792	-0.013600808	0.609	0.599	1
0.166024171	-0.013587356	0.15	0.144	1
0.024193579	-0.01358079	0.219	0.206	1
0.210200305	-0.013517973	0.112	0.107	1
0.357176799	-0.013503278	0.143	0.138	1
0.312956309	-0.013459792	0.169	0.163	1
0.063014235	-0.013452173	0.132	0.125	1
0.678350508	-0.013450878	0.136	0.133	1
0.603526912	-0.013440182	0.299	0.292	1
0.270160888	-0.013409238	0.379	0.365	1
0.919371459	-0.013406975	0.633	0.619	1
0.184925187	-0.013394528	0.157	0.151	1
0.131406834	-0.013390424	0.158	0.152	1
0.654369952	-0.013358835	0.287	0.285	1
0.057462125	-0.013357255	0.16	0.151	1
0.410669157	-0.013327073	0.105	0.102	1
0.013464026	-0.013316509	0.144	0.134	1
0.463991956	-0.013300142	0.14	0.136	1
0.707504905	-0.013290292	0.533	0.526	1
0.963492158	-0.013282834	0.591	0.58	1
0.859590565	-0.013280827	0.168	0.167	1
0.375208585	-0.013268069	0.124	0.12	1
0.034141976	-0.013222406	0.163	0.153	1
0.291109175	-0.01321866	0.156	0.15	1
0.207273957	-0.013203208	0.124	0.119	1
0.010078359	-0.01319594	0.163	0.152	1
0.183816283	-0.013183851	0.21	0.201	1

0.998261948	-0.013170688	0.108	0.107	1
0.98178142	-0.013168416	0.622	0.608	1
0.540238204	-0.013151138	0.182	0.177	1
0.628621244	-0.01311229	0.144	0.144	1
0.165230891	-0.013042118	0.156	0.149	1
0.851216295	-0.01302863	0.26	0.257	1
0.97544689	-0.012977295	0.762	0.754	1
0.404919615	-0.012960824	0.136	0.132	1
0.486627734	-0.012896714	0.477	0.463	1
0.138753929	-0.012872247	0.164	0.157	1
0.295579966	-0.012869556	0.155	0.149	1
0.247299098	-0.012837822	0.478	0.462	1
0.854251364	-0.01282776	0.107	0.106	1
0.106507584	-0.012822211	0.177	0.169	1
0.123608749	-0.012790736	0.134	0.127	1
0.555873033	-0.012697331	0.115	0.112	1
0.023259392	-0.012689878	0.284	0.268	1
0.219876595	-0.012665304	0.272	0.262	1
0.631023113	-0.012656707	0.132	0.129	1
0.279540123	-0.012641323	0.263	0.254	1
0.056374714	-0.012631888	0.168	0.159	1
0.08836623	-0.012604699	0.309	0.296	1
0.5174302	-0.012597047	0.412	0.401	1
0.290097885	-0.012595306	0.177	0.171	1
0.227833372	-0.012567716	0.143	0.138	1
0.065854045	-0.012566793	0.162	0.154	1
0.160767974	-0.012540355	0.351	0.337	1
0.280604347	-0.0125373	0.228	0.22	1
0.105623401	-0.012525826	0.171	0.163	1
0.578479879	-0.012525724	0.155	0.152	1
0.007745114	-0.012524561	0.16	0.149	1
0.57830911	-0.012504234	0.417	0.408	1
0.264638055	-0.012497883	0.146	0.141	1
0.041346995	-0.012366809	0.192	0.181	1
0.346931908	-0.01236389	0.132	0.128	1
0.195814538	-0.012307158	0.121	0.115	1
0.375771085	-0.012305901	0.104	0.1	1
0.527205068	-0.012292314	0.266	0.259	1
0.455886126	-0.012256849	0.115	0.112	1
0.725843413	-0.012251595	0.141	0.139	1
0.659298878	-0.012226781	0.173	0.169	1
0.514412855	-0.012225649	0.169	0.166	1
0.38660575	-0.012220726	0.17	0.165	1
0.054069104	-0.012194848	0.245	0.232	1
0.34644781	-0.012183681	0.174	0.169	1
0.13596811	-0.012180704	0.215	0.206	1
0.705026609	-0.012174235	0.168	0.166	1
0.194923955	-0.012168111	0.12	0.115	1
0.381009702	-0.012157884	0.109	0.105	1
0.071409334	-0.012143002	0.38	0.362	1

0.188479219	-0.012142106	0.119	0.114	1
0.16181645	-0.012115263	0.106	0.101	1
0.142180471	-0.012111679	0.184	0.177	1
0.886499702	-0.012089829	0.127	0.126	1
0.33610417	-0.012087021	0.234	0.237	1
0.287697669	-0.012080776	0.301	0.302	1
0.368684417	-0.012063908	0.104	0.101	1
0.628125912	-0.012039752	0.197	0.193	1
0.094705769	-0.012031381	0.17	0.162	1
0.134767615	-0.012027177	0.203	0.195	1
0.001364719	-0.01201746	0.267	0.248	1
0.00547063	-0.012000835	0.209	0.195	1
0.212802013	-0.011985266	0.258	0.249	1
0.346962213	-0.011957784	0.138	0.134	1
0.206137248	-0.011866915	0.307	0.295	1
0.242601584	-0.01177106	0.144	0.139	1
0.05027827	-0.011762891	0.188	0.178	1
0.73695765	-0.011734011	0.15	0.148	1
0.701246439	-0.011733409	0.152	0.15	1
0.668266773	-0.011718668	0.13	0.127	1
0.065766975	-0.011703011	0.137	0.13	1
0.915768624	-0.011701311	0.122	0.122	1
0.43708677	-0.011676147	0.146	0.142	1
0.545135638	-0.01166744	0.457	0.443	1
0.172147755	-0.011664211	0.172	0.165	1
0.261457053	-0.011655042	0.116	0.111	1
0.382542091	-0.011641149	0.162	0.158	1
0.105970843	-0.01156223	0.348	0.333	1
0.015956148	-0.011554354	0.172	0.161	1
0.499676248	-0.011545984	0.123	0.12	1
0.58671488	-0.011542649	0.104	0.102	1
0.514445649	-0.011542042	0.138	0.134	1
0.070090176	-0.011521672	0.773	0.762	1
0.251652271	-0.0114994	0.149	0.144	1
0.378491316	-0.011488104	0.165	0.167	1
0.160179249	-0.011477374	0.156	0.15	1
0.107355299	-0.011455883	0.165	0.158	1
0.266601147	-0.011440009	0.596	0.589	1
0.514473803	-0.011414129	0.803	0.799	1
0.095960817	-0.011408881	0.234	0.223	1
0.108969845	-0.011396156	0.232	0.222	1
0.039640727	-0.011385056	0.122	0.114	1
0.147986782	-0.011383487	0.115	0.109	1
0.255246756	-0.011371317	0.185	0.178	1
0.270771505	-0.011354776	0.102	0.098	1
0.530923556	-0.011337938	0.18	0.176	1
0.421659243	-0.011334723	0.255	0.247	1
0.457842435	-0.011315353	0.129	0.125	1
0.224112319	-0.011303841	0.114	0.109	1
0.02720893	-0.011282397	0.22	0.207	1

0.258965365	-0.011254428	0.287	0.276	1
0.24296286	-0.011251558	0.145	0.14	1
0.692070603	-0.011242671	0.162	0.162	1
0.823197692	-0.011229943	0.112	0.111	1
0.193230055	-0.011222942	0.153	0.147	1
0.286282087	-0.011194512	0.4	0.386	1
0.40170175	-0.011175748	0.196	0.19	1
0.352510132	-0.011170602	0.102	0.099	1
0.255283304	-0.01108973	0.424	0.409	1
0.372231279	-0.011036059	0.151	0.146	1
0.157382325	-0.01103036	0.22	0.211	1
0.018032454	-0.0110234	0.129	0.12	1
0.665718384	-0.01101505	0.499	0.485	1
0.655613886	-0.011009467	0.109	0.109	1
0.028920155	-0.010969185	0.649	0.647	1
0.154875117	-0.010966071	0.363	0.349	1
0.05478693	-0.010942563	0.262	0.249	1
0.673653503	-0.010864173	0.157	0.154	1
0.944886032	-0.0107885	0.287	0.283	1
0.849433171	-0.010783297	0.207	0.203	1
0.722720806	-0.010781942	0.217	0.216	1
0.289577695	-0.010776759	0.104	0.1	1
0.396489072	-0.010766755	0.495	0.487	1
0.123863471	-0.010754336	0.341	0.341	1
0.029910582	-0.01074444	0.162	0.151	1
0.406819733	-0.010670364	0.186	0.182	1
0.58402705	-0.010669017	0.107	0.108	1
0.115181729	-0.010661253	0.111	0.105	1
0.391164904	-0.010654449	0.164	0.159	1
0.909213909	-0.010592566	0.16	0.158	1
0.802052868	-0.010574962	0.465	0.455	1
0.117748983	-0.010571153	0.328	0.314	1
0.917577293	-0.010569429	0.374	0.371	1
0.067761935	-0.01055723	0.203	0.192	1
0.019095457	-0.010537072	0.296	0.28	1
0.960710899	-0.010521772	0.271	0.267	1
0.138212049	-0.010515902	0.107	0.102	1
0.444289989	-0.010510029	0.177	0.171	1
0.029458905	-0.010508836	0.255	0.242	1
0.538246799	-0.010502814	0.147	0.143	1
0.004489688	-0.01050015	0.308	0.288	1
0.018235348	-0.010490717	0.153	0.143	1
0.101290712	-0.010484739	0.119	0.113	1
0.112629813	-0.010464292	0.137	0.13	1
0.540321263	-0.010458971	0.135	0.132	1
0.393852023	-0.010456399	0.376	0.375	1
0.063960706	-0.010444984	0.119	0.112	1
0.017696722	-0.010414203	0.145	0.135	1
0.130300187	-0.010351223	0.145	0.138	1
0.261387504	-0.010348589	0.32	0.31	1

0.315174368	-0.010286795	0.154	0.148	1
0.786784099	-0.010173328	0.148	0.148	1
0.074953484	-0.01016149	0.131	0.124	1
0.845857126	-0.010143718	0.168	0.166	1
0.814816724	-0.010087089	0.11	0.11	1
0.197248489	-0.010070089	0.107	0.103	1
0.172175174	-0.010055996	0.116	0.11	1
0.092414835	-0.010037772	0.166	0.158	1
0.30566259	-0.010007922	0.117	0.113	1
0.261795807	-0.010002031	0.108	0.104	1
0.791554271	-0.009993509	0.13	0.128	1
0.406123524	-0.00997987	0.518	0.503	1
0.125102769	-0.009964658	0.144	0.138	1
0.904976194	-0.009962701	0.131	0.131	1
0.921968001	-0.009946429	0.122	0.122	1
0.302843419	-0.009930513	0.918	0.911	1
0.585357289	-0.009922028	0.443	0.438	1
0.030213442	-0.009919912	0.13	0.122	1
0.625117074	-0.009913995	0.138	0.139	1
0.555953753	-0.00989238	0.422	0.417	1
0.674514734	-0.009891826	0.11	0.107	1
0.047919185	-0.009864352	0.25	0.237	1
0.682670002	-0.009861216	0.101	0.102	1
0.85003476	-0.009840595	0.233	0.232	1
0.027613844	-0.009829452	0.106	0.099	1
0.729313283	-0.00981912	0.135	0.133	1
0.055090859	-0.009815977	0.11	0.103	1
0.012835141	-0.009813925	0.236	0.223	1
0.209162441	-0.009801257	0.332	0.32	1
0.602753949	-0.009798536	0.102	0.099	1
0.530212688	-0.009795601	0.236	0.231	1
0.358714892	-0.009785051	0.121	0.117	1
0.310565414	-0.009773471	0.259	0.259	1
0.999741776	-0.009755927	0.451	0.439	1
0.301987312	-0.009718522	0.128	0.124	1
0.233880857	-0.009709095	0.133	0.128	1
0.029814069	-0.009676728	0.185	0.174	1
0.03982025	-0.009676286	0.174	0.164	1
0.156326847	-0.009632373	0.167	0.16	1
0.069968505	-0.009611627	0.254	0.242	1
0.623214335	-0.00959781	0.101	0.099	1
0.000496071	-0.00959374	0.237	0.22	1
0.019335701	-0.009566012	0.161	0.151	1
0.083391298	-0.009543741	0.137	0.13	1
0.961639728	-0.009518428	0.622	0.608	1
0.013738205	-0.009501911	0.161	0.15	1
0.540952884	-0.009490454	0.19	0.186	1
0.042532974	-0.009488654	0.209	0.198	1
0.204206438	-0.009480638	0.183	0.176	1
0.06937286	-0.009477917	0.346	0.329	1

0.09558114	-0.009456617	0.165	0.157	1
0.651791726	-0.009453438	0.323	0.317	1
0.276260191	-0.009445896	0.12	0.115	1
0.874623903	-0.009436453	0.143	0.141	1
0.107974069	-0.009427379	0.117	0.111	1
0.063171913	-0.009372081	0.124	0.117	1
0.079641281	-0.009364132	0.175	0.166	1
0.147107357	-0.009336448	0.154	0.147	1
0.229465853	-0.009293246	0.103	0.099	1
0.024638032	-0.009290325	0.154	0.144	1
0.173281803	-0.009285434	0.236	0.227	1
0.597116338	-0.00927053	0.102	0.1	1
0.080728685	-0.009231919	0.115	0.109	1
0.049686468	-0.009224645	0.114	0.108	1
0.027998366	-0.009209301	0.161	0.151	1
0.001779684	-0.009202973	0.207	0.192	1
0.083961486	-0.009189558	0.138	0.131	1
0.088609853	-0.009166076	0.184	0.175	1
0.05660181	-0.009163114	0.249	0.237	1
0.071668942	-0.009124819	0.146	0.138	1
0.3120263	-0.009119791	0.351	0.34	1
0.167415473	-0.009072274	0.356	0.341	1
0.017707328	-0.009068669	0.116	0.107	1
0.198284598	-0.009064372	0.124	0.119	1
0.855177537	-0.0090545	0.221	0.219	1
0.07391796	-0.009040359	0.207	0.198	1
0.070196719	-0.009030407	0.39	0.37	1
0.687836174	-0.009008716	0.118	0.115	1
0.054029593	-0.008993482	0.206	0.196	1
0.465920303	-0.008963697	0.595	0.591	1
0.026561449	-0.008941441	0.119	0.111	1
0.405119359	-0.008937108	0.233	0.226	1
0.135951355	-0.008907139	0.162	0.155	1
0.074407747	-0.00889891	0.133	0.126	1
0.030034952	-0.008898592	0.24	0.227	1
0.017368675	-0.008895888	0.316	0.297	1
0.897941429	-0.008894307	0.14	0.14	1
0.246635487	-0.008887987	0.128	0.123	1
0.723354347	-0.008870752	0.225	0.22	1
0.076753101	-0.008826434	0.126	0.12	1
0.689931615	-0.008826282	0.148	0.145	1
0.137058771	-0.008752447	0.113	0.107	1
0.021471764	-0.008734586	0.178	0.167	1
0.000340919	-0.008728655	0.119	0.106	1
0.174727708	-0.008708755	0.423	0.406	1
0.304515255	-0.008687177	0.181	0.175	1
0.02330101	-0.008678657	0.112	0.104	1
0.098139024	-0.008617505	0.117	0.111	1
0.022765233	-0.008608001	0.117	0.109	1
0.25033518	-0.008587936	0.115	0.11	1

0.794485192	-0.008568657	0.126	0.126	1
0.026665559	-0.008562225	0.11	0.102	1
0.065933866	-0.00854258	0.159	0.151	1
0.872447451	-0.008509477	0.207	0.205	1
0.000938087	-0.008475717	0.186	0.17	1
0.165829977	-0.008456692	0.186	0.179	1
0.062825791	-0.008456198	0.208	0.197	1
0.001700155	-0.008437357	0.177	0.163	1
0.513818783	-0.008420427	0.173	0.169	1
0.177816464	-0.008411726	0.116	0.111	1
0.212639043	-0.008397945	0.375	0.363	1
0.252615765	-0.008386549	0.259	0.251	1
0.099579012	-0.008386386	0.119	0.113	1
0.801247265	-0.008308683	0.123	0.122	1
0.024942778	-0.008306983	0.147	0.137	1
0.109804842	-0.00830174	0.125	0.119	1
0.51562644	-0.008273481	0.161	0.157	1
0.063637606	-0.008253839	0.128	0.12	1
0.049733754	-0.008228362	0.163	0.154	1
0.243560493	-0.008224149	0.256	0.246	1
0.190840867	-0.008217635	0.441	0.424	1
0.132648239	-0.008159282	0.171	0.164	1
0.287948329	-0.008153932	0.431	0.417	1
0.042867503	-0.008151115	0.197	0.186	1
0.173755865	-0.008105689	0.112	0.107	1
0.053929132	-0.0080932	0.23	0.219	1
0.012361377	-0.008091917	0.131	0.121	1
0.032226847	-0.008072449	0.126	0.118	1
0.14106153	-0.008071456	0.121	0.115	1
0.345453393	-0.008059641	0.138	0.133	1
0.007911226	-0.008001125	0.279	0.262	1
0.277674209	-0.007986674	0.198	0.191	1
0.19712498	-0.007971734	0.274	0.264	1
0.194660614	-0.00796778	0.287	0.276	1
0.122313738	-0.007964881	0.277	0.266	1
0.148374946	-0.007950931	0.116	0.12	1
0.471566015	-0.007945546	0.237	0.238	1
0.031297221	-0.007909511	0.218	0.206	1
0.03316372	-0.007876553	0.119	0.111	1
0.134635102	-0.00786899	0.105	0.1	1
0.118865355	-0.007860954	0.332	0.318	1
0.56180277	-0.007852474	0.117	0.114	1
0.271895158	-0.007839283	0.13	0.126	1
0.095991992	-0.007815283	0.209	0.199	1
0.176438705	-0.007803521	0.112	0.107	1
0.009825362	-0.007792952	0.191	0.179	1
0.11360064	-0.007762712	0.304	0.291	1
0.256352178	-0.007760567	0.15	0.144	1
0.111724705	-0.007759499	0.105	0.1	1
0.107682394	-0.00775559	0.392	0.375	1

0.026237628	-0.007753406	0.211	0.2	1
0.057903513	-0.007740555	0.181	0.172	1
0.284682052	-0.007733009	0.213	0.206	1
0.168949381	-0.007723007	0.123	0.118	1
0.01368077	-0.007690539	0.226	0.212	1
0.014399307	-0.007682288	0.233	0.219	1
0.006568647	-0.00766446	0.183	0.171	1
0.224143288	-0.00765991	0.122	0.117	1
0.074674995	-0.007658989	0.296	0.283	1
0.034591717	-0.007652328	0.239	0.226	1
0.855535713	-0.007650913	0.341	0.332	1
0.640645365	-0.007605447	0.156	0.153	1
0.208661167	-0.00757731	0.169	0.162	1
6.97349E-05	-0.007557829	0.861	0.86	1
0.669250217	-0.007549188	0.156	0.153	1
0.027465929	-0.007534786	0.179	0.169	1
0.02579565	-0.007524437	0.231	0.218	1
0.190830935	-0.007498111	0.164	0.157	1
0.109559702	-0.007496444	0.155	0.148	1
0.012516224	-0.007496317	0.289	0.272	1
0.219021417	-0.007488761	0.122	0.117	1
0.021899746	-0.007456498	0.111	0.104	1
0.011629863	-0.007410179	0.118	0.109	1
0.478371454	-0.007391636	0.346	0.337	1
0.078977599	-0.007383426	0.275	0.262	1
0.756743682	-0.007372443	0.123	0.121	1
0.013348499	-0.007367204	0.268	0.252	1
0.090703617	-0.007362662	0.105	0.099	1
0.367386088	-0.007288498	0.378	0.365	1
0.833680102	-0.007265647	0.11	0.11	1
0.003499825	-0.007244112	0.184	0.17	1
0.925053346	-0.007241911	0.339	0.332	1
0.415072373	-0.007239502	0.346	0.336	1
0.056839174	-0.007235843	0.229	0.218	1
0.591192004	-0.007229613	0.161	0.158	1
0.191821584	-0.007196688	0.239	0.23	1
0.075015056	-0.007193872	0.254	0.243	1
0.01092455	-0.007163811	0.15	0.14	1
0.212708801	-0.007158967	0.109	0.105	1
0.166852193	-0.007109274	0.137	0.131	1
0.285577979	-0.007103829	0.128	0.123	1
0.04211399	-0.006975953	0.202	0.191	1
0.564031414	-0.006970696	0.485	0.476	1
0.157814992	-0.006951239	0.112	0.107	1
0.003497299	-0.006930089	0.168	0.156	1
0.066936198	-0.006921388	0.115	0.108	1
0.033221885	-0.006906221	0.325	0.309	1
0.197840688	-0.006836028	0.2	0.192	1
0.294648709	-0.006834713	0.115	0.118	1
0.031164507	-0.006794413	0.131	0.123	1

0.361693161	-0.00677926	0.113	0.109	1
0.01657309	-0.006763789	0.255	0.241	1
0.531158284	-0.006761578	0.107	0.108	1
0.59930481	-0.00674446	0.634	0.615	1
0.449758375	-0.006692001	0.123	0.12	1
0.238623776	-0.006678869	0.107	0.103	1
0.065083971	-0.006672251	0.167	0.159	1
0.053500836	-0.006645946	0.185	0.176	1
0.270182866	-0.006609883	0.336	0.325	1
0.213860155	-0.006599602	0.157	0.151	1
0.103736794	-0.006525173	0.103	0.097	1
0.627042117	-0.006489564	0.102	0.104	1
0.196767182	-0.006481754	0.177	0.18	1
0.265080048	-0.006475915	0.459	0.442	1
0.938817623	-0.006438153	0.282	0.28	1
0.642626675	-0.006427954	0.132	0.13	1
0.035978612	-0.00642595	0.243	0.231	1
0.001650365	-0.006402121	0.174	0.161	1
0.128057361	-0.00639712	0.253	0.242	1
0.070491475	-0.006387915	0.2	0.19	1
0.112838811	-0.006378916	0.406	0.391	1
0.012256208	-0.006335452	0.128	0.136	1
0.454576554	-0.006314148	0.266	0.265	1
0.030341077	-0.006303851	0.186	0.176	1
0.00223658	-0.006241169	0.211	0.196	1
0.126107542	-0.006218969	0.114	0.108	1
0.214086684	-0.006199882	0.145	0.139	1
0.209953966	-0.006171933	0.494	0.475	1
0.071857396	-0.006131259	0.166	0.158	1
0.034664736	-0.006118134	0.111	0.104	1
0.032477905	-0.006110312	0.126	0.118	1
0.95182785	-0.006093854	0.795	0.786	1
0.302656061	-0.006090542	0.265	0.257	1
0.04447577	-0.006057957	0.114	0.107	1
0.636310056	-0.006035421	0.105	0.103	1
0.027902211	-0.006029945	0.206	0.195	1
0.001299638	-0.006005353	0.113	0.102	1
0.6507431	-0.006003889	0.338	0.334	1
0.422574847	-0.005999801	0.111	0.108	1
0.020508399	-0.005993203	0.208	0.196	1
0.007061751	-0.005993134	0.117	0.108	1
0.109677515	-0.00597609	0.215	0.205	1
0.090095885	-0.005972449	0.15	0.142	1
0.631781617	-0.005942862	0.119	0.116	1
0.075143637	-0.005880999	0.291	0.277	1
0.006218921	-0.005841808	0.149	0.138	1
0.160890575	-0.005799237	0.162	0.156	1
0.331891073	-0.005778352	0.123	0.119	1
0.028856336	-0.005751618	0.163	0.154	1
0.185043145	-0.005732439	0.125	0.12	1

0.27642394	-0.00571724	0.158	0.152	1
0.007207348	-0.005715091	0.145	0.134	1
0.004894122	-0.005714942	0.28	0.262	1
0.467872082	-0.005708233	0.299	0.29	1
0.04794035	-0.005681801	0.493	0.473	1
0.559679077	-0.005661114	0.13	0.127	1
0.754180746	-0.005651485	0.107	0.106	1
0.263812726	-0.005645179	0.16	0.154	1
0.593113276	-0.005637648	0.153	0.15	1
0.057563301	-0.005615998	0.286	0.272	1
0.109164637	-0.00561481	0.304	0.292	1
0.027117023	-0.005610793	0.142	0.133	1
0.772976981	-0.0055903	0.129	0.127	1
0.005145481	-0.005554507	0.115	0.105	1
0.257901225	-0.005522466	0.711	0.701	1
0.035768282	-0.005493129	0.122	0.115	1
0.005266138	-0.005470226	0.124	0.114	1
0.140772155	-0.005465257	0.124	0.118	1
0.24651402	-0.005447854	0.243	0.235	1
0.107323364	-0.005434118	0.167	0.16	1
0.189296703	-0.005433708	0.103	0.108	1
0.004917397	-0.005406738	0.114	0.104	1
0.426983403	-0.00539456	0.105	0.107	1
0.083342684	-0.00537869	0.22	0.209	1
0.615812748	-0.005313073	0.266	0.259	1
0.380469937	-0.005285894	0.156	0.152	1
0.035776699	-0.005266971	0.317	0.302	1
0.087757116	-0.005231484	0.136	0.13	1
0.133596969	-0.005215634	0.291	0.28	1
0.572546462	-0.005196178	0.12	0.117	1
0.710488399	-0.005173629	0.272	0.269	1
0.292917725	-0.005171915	0.576	0.559	1
0.991614392	-0.005160721	0.65	0.639	1
0.094256382	-0.005149676	0.105	0.099	1
0.054824499	-0.00514904	0.277	0.265	1
0.04580447	-0.005128354	0.251	0.238	1
0.152576852	-0.005128317	0.12	0.115	1
0.048830635	-0.005123236	0.124	0.117	1
0.001007767	-0.005101723	0.219	0.202	1
0.007192767	-0.005090405	0.238	0.223	1
0.062285437	-0.005079615	0.296	0.282	1
0.136500982	-0.005062802	0.286	0.275	1
0.199590409	-0.005055795	0.17	0.164	1
0.120640024	-0.005054755	0.111	0.105	1
0.063585429	-0.004983725	0.103	0.097	1
0.027995625	-0.004983362	0.246	0.234	1
0.223358116	-0.004920119	0.139	0.134	1
0.368307217	-0.004893979	0.16	0.155	1
0.056285115	-0.004889073	0.212	0.201	1
0.034555006	-0.004863482	0.12	0.112	1

0.003930215	-0.004861089	0.179	0.166	1
0.061236898	-0.004859438	0.118	0.111	1
0.366665975	-0.004846913	0.155	0.151	1
0.09008799	-0.00483957	0.192	0.183	1
0.11951695	-0.004833502	0.174	0.166	1
0.53331439	-0.004797702	0.308	0.3	1
0.832538201	-0.004789949	0.12	0.118	1
0.107222377	-0.004767592	0.114	0.108	1
0.0030781	-0.004766473	0.142	0.13	1
0.228179754	-0.004765941	0.115	0.111	1
0.029038012	-0.004763491	0.146	0.137	1
0.851056463	-0.004728505	0.143	0.141	1
0.051359054	-0.004727904	0.126	0.118	1
0.199761039	-0.004687396	0.369	0.356	1
0.141092457	-0.004595557	0.136	0.129	1
0.009813611	-0.004576377	0.231	0.216	1
0.284509001	-0.004559509	0.169	0.164	1
0.027603863	-0.004547771	0.266	0.252	1
0.21913262	-0.004535112	0.231	0.222	1
0.086849491	-0.004530857	0.182	0.174	1
0.172700978	-0.004506453	0.257	0.248	1
0.117088741	-0.004471305	0.118	0.112	1
0.08122154	-0.004470551	0.102	0.096	1
0.402147809	-0.004454809	0.232	0.225	1
0.009870623	-0.00442021	0.321	0.302	1
0.01049231	-0.004406906	0.119	0.11	1
0.034303908	-0.004405243	0.144	0.136	1
0.00829428	-0.004395545	0.217	0.203	1
0.100763715	-0.004373511	0.172	0.165	1
0.021015179	-0.004354569	0.102	0.094	1
0.651936213	-0.004329173	0.475	0.464	1
0.029196786	-0.004320801	0.18	0.17	1
0.045605114	-0.004277794	0.182	0.173	1
0.052790291	-0.004273188	0.198	0.188	1
0.769034492	-0.004262061	0.111	0.11	1
0.043284386	-0.004230037	0.243	0.231	1
0.027447503	-0.004194762	0.173	0.162	1
0.055335646	-0.004170297	0.238	0.227	1
0.021259085	-0.004156864	0.861	0.855	1
0.058864781	-0.004152255	0.104	0.098	1
0.000108823	-0.004121869	0.369	0.347	1
0.351888904	-0.00411096	0.149	0.145	1
0.484023685	-0.004092888	0.12	0.117	1
0.689418377	-0.00408342	0.16	0.157	1
0.101596227	-0.004063155	0.294	0.282	1
0.069392435	-0.004060073	0.15	0.142	1
0.262802978	-0.004040646	0.12	0.115	1
0.887201768	-0.004017185	0.163	0.161	1
0.399783703	-0.004014919	0.126	0.123	1
0.000221623	-0.003976969	0.161	0.146	1

0.38857932	-0.003973033	0.111	0.108	1
0.034131938	-0.003963589	0.122	0.115	1
0.006021112	-0.003925884	0.156	0.144	1
0.295744609	-0.003920635	0.314	0.305	1
0.271718002	-0.003904971	0.127	0.122	1
0.299221641	-0.003898539	0.497	0.478	1
0.479661466	-0.003896983	0.192	0.188	1
0.001228697	-0.003877824	0.104	0.094	1
0.186185568	-0.003858087	0.224	0.215	1
0.032546532	-0.003826217	0.108	0.101	1
0.019966638	-0.003810259	0.103	0.096	1
0.046698629	-0.003804841	0.149	0.141	1
0.004817768	-0.003801193	0.321	0.302	1
0.024870418	-0.003773756	0.11	0.102	1
0.007992063	-0.003763445	0.216	0.201	1
0.019866397	-0.003748297	0.111	0.103	1
0.165509898	-0.003730613	0.116	0.111	1
0.371473159	-0.003721011	0.553	0.536	1
0.065920087	-0.00369934	0.342	0.328	1
0.049400942	-0.003665038	0.166	0.157	1
0.269578977	-0.003613841	0.185	0.178	1
0.011981178	-0.00360142	0.246	0.232	1
0.072849587	-0.003591498	0.174	0.165	1
0.031194252	-0.003549975	0.104	0.097	1
0.040875864	-0.003480136	0.314	0.299	1
0.112453448	-0.003393772	0.243	0.232	1
0.125410087	-0.003393627	0.225	0.215	1
0.486476749	-0.003250248	0.136	0.133	1
0.080206676	-0.003225387	0.22	0.21	1
0.109368501	-0.003217314	0.133	0.127	1
0.013610805	-0.003166283	0.171	0.16	1
0.017942011	-0.003155325	0.258	0.243	1
0.035173952	-0.003154449	0.191	0.18	1
0.11683179	-0.003097191	0.254	0.243	1
0.010331166	-0.003091476	0.1	0.092	1
0.000616137	-0.003088461	0.129	0.117	1
0.003327828	-0.003078971	0.232	0.216	1
0.826883147	-0.003055903	0.117	0.116	1
0.078783121	-0.003053716	0.108	0.102	1
0.873420468	-0.003040796	0.136	0.135	1
0.078071641	-0.003035279	0.108	0.102	1
0.195078663	-0.003033546	0.114	0.109	1
0.052903208	-0.00302745	0.176	0.167	1
0.005049232	-0.003013972	0.123	0.113	1
0.404298216	-0.00300061	0.123	0.12	1
0.077616042	-0.002986177	0.169	0.161	1
0.055521245	-0.002964066	0.254	0.242	1
0.038325198	-0.002957893	0.182	0.172	1
0.455137133	-0.00289662	0.402	0.389	1
0.020515866	-0.002853256	0.241	0.227	1

0.314686525	-0.00284858	0.132	0.127	1
0.004465776	-0.002798166	0.21	0.196	1
0.035088723	-0.002769844	0.104	0.097	1
0.002823014	-0.002758253	0.248	0.231	1
0.141729783	-0.002753252	0.397	0.381	1
0.012015434	-0.00274646	0.247	0.232	1
0.579389894	-0.002718495	0.113	0.111	1
0.034475909	-0.002712422	0.127	0.119	1
0.006010808	-0.002703488	0.313	0.295	1
0.071454789	-0.002669551	0.327	0.312	1
0.01047517	-0.002653057	0.208	0.194	1
0.000372963	-0.002643174	0.45	0.424	1
0.729221706	-0.002636066	0.429	0.425	1
0.088397453	-0.002601282	0.131	0.125	1
0.601071506	-0.002596993	0.535	0.519	1
0.003981565	-0.00259615	0.822	0.817	1
0.390468736	-0.002536356	0.127	0.123	1
0.568279081	-0.002533369	0.183	0.18	1
0.005387867	-0.002440197	0.248	0.233	1
0.03312096	-0.002400291	0.118	0.111	1
0.018362597	-0.002370992	0.103	0.096	1
0.216807646	-0.002363516	0.502	0.487	1
0.075618156	-0.002343217	0.135	0.128	1
0.389889333	-0.002319602	0.116	0.112	1
0.012942473	-0.002291616	0.448	0.427	1
0.024322758	-0.002290798	0.149	0.14	1
0.020911498	-0.002287022	0.255	0.241	1
0.000333521	-0.002281909	0.132	0.119	1
0.080032588	-0.002279768	0.1	0.095	1
0.616189263	-0.002264107	0.422	0.412	1
0.519081335	-0.002242194	0.516	0.501	1
0.470251822	-0.002218086	0.427	0.415	1
0.037622758	-0.002196486	0.183	0.173	1
0.000166389	-0.00219637	0.161	0.146	1
0.003479757	-0.002191806	0.195	0.18	1
0.003139876	-0.002181302	0.135	0.124	1
0.075097467	-0.002176635	0.385	0.371	1
0.025807343	-0.002176211	0.101	0.094	1
0.277725994	-0.002155134	0.244	0.236	1
0.581462822	-0.002152518	0.146	0.147	1
0.271904807	-0.002139933	0.107	0.103	1
0.019201756	-0.00212451	0.109	0.101	1
0.715612131	-0.002109172	0.118	0.116	1
0.007439437	-0.002057813	0.159	0.148	1
0.009867827	-0.002049417	0.273	0.256	1
0.017510861	-0.002026771	0.263	0.248	1
0.002316985	-0.0020194	0.201	0.187	1
0.561192475	-0.002010606	0.928	0.924	1
0.050808311	-0.001997017	0.14	0.132	1
0.026345016	-0.001994917	0.142	0.133	1

0.072985579	-0.001991652	0.182	0.173	1
0.330722162	-0.001962105	0.331	0.32	1
0.080180285	-0.001890455	0.133	0.125	1
0.120991649	-0.001876245	0.131	0.124	1
0.121056828	-0.001857486	0.189	0.181	1
0.741005917	-0.001842685	0.321	0.315	1
0.028448436	-0.001826537	0.173	0.162	1
0.634030304	-0.001798421	0.386	0.378	1
0.038839242	-0.00179586	0.136	0.128	1
0.099143715	-0.001771422	0.122	0.116	1
0.010364043	-0.001769449	0.131	0.121	1
0.155624697	-0.0016652	0.138	0.132	1
0.004780012	-0.001623708	0.134	0.123	1
0.002461883	-0.001620049	0.177	0.163	1
0.02865527	-0.001615277	0.133	0.124	1
0.126390653	-0.001610904	0.104	0.099	1
0.011503217	-0.001607965	0.216	0.203	1
0.235656172	-0.001583591	0.389	0.375	1
0.039107999	-0.001564123	0.144	0.136	1
0.002402377	-0.001543688	0.375	0.35	1
0.008085928	-0.001540527	0.343	0.323	1
0.013483172	-0.001535253	0.119	0.11	1
0.965484639	-0.001508549	0.128	0.128	1
0.009935246	-0.001495641	0.102	0.094	1
0.000435422	-0.001451994	0.134	0.121	1
0.958759532	-0.001441788	0.161	0.16	1
0.023421997	-0.001428358	0.201	0.19	1
0.039515496	-0.001421751	0.139	0.131	1
0.072023133	-0.001417779	0.214	0.204	1
0.000511132	-0.001415676	0.275	0.254	1
0.047042362	-0.001403483	0.203	0.192	1
0.041596652	-0.00138756	0.118	0.111	1
0.537105464	-0.001369356	0.177	0.173	1
0.453674785	-0.001363234	0.112	0.109	1
0.002204865	-0.00136114	0.366	0.345	1
0.257797515	-0.001321319	0.236	0.228	1
0.0171226	-0.001290521	0.17	0.16	1
0.00086099	-0.00128896	0.103	0.093	1
0.000135999	-0.001269646	0.131	0.117	1
0.172734388	-0.001249569	0.314	0.302	1
0.087503913	-0.001231483	0.107	0.101	1
0.186987342	-0.001224813	0.392	0.377	1
0.008025218	-0.001168481	0.257	0.241	1
0.10565869	-0.001162705	0.361	0.346	1
0.211879484	-0.001145807	0.737	0.728	1
0.004426512	-0.001142867	0.231	0.216	1
0.02114893	-0.001093455	0.126	0.118	1
0.134571745	-0.001091796	0.1	0.095	1
0.038827979	-0.001089959	0.247	0.235	1
0.011178656	-0.001078872	0.117	0.108	1

0.170691869	-0.001074183	0.158	0.151	1
0.182707935	-0.001011202	0.113	0.108	1
0.49105405	-0.001005865	0.155	0.151	1
0.020135671	-0.000993864	0.24	0.227	1
0.040368976	-0.000975422	0.121	0.113	1
0.278299179	-0.00097122	0.109	0.105	1
0.001173802	-0.000963319	0.119	0.108	1
0.045105118	-0.000956851	0.198	0.188	1
0.019118114	-0.000941814	0.143	0.133	1
0.423681801	-0.000911126	0.101	0.103	1
0.680011321	-0.000905384	0.433	0.421	1
0.015091123	-0.000883645	0.261	0.248	1
0.09283071	-0.000829257	0.214	0.204	1
0.029327056	-0.000828881	0.15	0.141	1
0.069674955	-0.000775627	0.183	0.174	1
0.06219788	-0.000699794	0.199	0.189	1
0.028671424	-0.000690494	0.164	0.154	1
0.21575807	-0.000687912	0.101	0.097	1
0.00389632	-0.000660868	0.349	0.329	1
0.228248998	-0.000636709	0.816	0.81	1
0.000483947	-0.000608849	0.113	0.101	1
0.410924809	-0.000599432	0.143	0.139	1
0.072671332	-0.000579759	0.124	0.118	1
0.059459101	-0.000577076	0.107	0.101	1
0.007777298	-0.00052342	0.214	0.201	1
0.257691971	-0.00051683	0.317	0.307	1
0.831272318	-0.000480445	0.171	0.172	1
0.006347587	-0.000476151	0.201	0.188	1
0.112772024	-0.000465207	0.153	0.146	1
0.073795921	-0.000456899	0.322	0.307	1
0.01803233	-0.000432131	0.127	0.118	1
0.011374085	-0.000412003	0.165	0.154	1
0.946453942	-0.000396105	0.165	0.164	1
0.005548742	-0.000370236	0.22	0.206	1
0.015524388	-0.000347365	0.107	0.099	1
0.450384352	-0.000344354	0.135	0.131	1
0.252401312	-0.000334166	0.407	0.393	1
0.018755199	-0.000325904	0.111	0.103	1
0.00840335	-0.000316878	0.169	0.157	1
0.029851121	-0.000304947	0.879	0.866	1
0.006810627	-0.000243665	0.273	0.257	1
0.005487	-0.000240352	0.392	0.371	1
0.112672819	-0.000223754	0.121	0.115	1
0.005972247	-0.00020424	0.195	0.182	1
0.27372408	-0.000198803	0.281	0.272	1
0.068882122	-0.000177701	0.124	0.118	1
0.195685611	-0.000148727	0.17	0.163	1
0.054374903	-0.0001331	0.141	0.134	1
0.003281742	-0.000133086	0.301	0.282	1
0.033237639	-0.000131139	0.194	0.183	1

0.018528804	-6.76878E-05	0.224	0.211	1
0.085691138	-4.89053E-05	0.106	0.1	1
0.00991658	-2.33365E-05	0.139	0.129	1
0.043592009	-1.91376E-05	0.22	0.208	1
0.000498831	-1.52085E-05	0.18	0.165	1
0.077102433	5.31191E-06	0.182	0.174	1
0.045038821	2.12775E-05	0.396	0.377	1
0.837963442	8.18624E-05	0.381	0.374	1
0.047816631	0.000100738	0.12	0.113	1
0.323637318	0.000107589	0.225	0.218	1
0.003112844	0.000110284	0.147	0.135	1
0.0831903	0.00012191	0.137	0.13	1
0.406606901	0.000125552	0.613	0.596	1
0.001770257	0.000127804	0.268	0.249	1
0.033446042	0.000133845	0.378	0.383	1
0.167336657	0.000137889	0.157	0.151	1
0.03627326	0.000138395	0.307	0.292	1
0.009520225	0.000187059	0.111	0.102	1
0.026590716	0.000236834	0.301	0.285	1
0.016871101	0.000246967	0.254	0.24	1
0.071397902	0.0002607	0.371	0.356	1
0.380530055	0.000269331	0.104	0.101	1
0.177934286	0.000282431	0.183	0.176	1
0.39907476	0.000284928	0.556	0.542	1
0.00334546	0.000322557	0.165	0.152	1
0.251547407	0.000332989	0.109	0.105	1
0.000263995	0.000366449	0.105	0.093	1
0.163667683	0.000369699	0.405	0.394	1
0.351023744	0.000419276	0.368	0.357	1
0.24948839	0.000425987	0.117	0.113	1
0.197968258	0.000441686	0.326	0.314	1
0.085393853	0.000447902	0.215	0.206	1
0.032136066	0.000471503	0.196	0.185	1
0.001089817	0.000481975	0.139	0.127	1
0.030702897	0.000488519	0.104	0.097	1
0.000393858	0.000511056	0.164	0.149	1
0.104254953	0.000524668	0.142	0.136	1
0.132745797	0.000539518	0.379	0.363	1
0.000514051	0.000565353	0.321	0.301	1
0.00229529	0.000579605	0.136	0.124	1
0.043343127	0.000650199	0.166	0.157	1
0.493251764	0.000663564	0.13	0.127	1
0.014104583	0.000672655	0.152	0.142	1
0.87050535	0.000723194	0.74	0.741	1
0.154908008	0.000725533	0.11	0.105	1
0.010630871	0.000735717	0.123	0.114	1
0.11030327	0.000758639	0.124	0.118	1
0.677669922	0.000795943	0.107	0.105	1
0.008084182	0.000802455	0.166	0.155	1
0.080230079	0.000809362	0.128	0.121	1

0.002982362	0.00082325	0.224	0.209	1
0.628995219	0.000823544	0.346	0.339	1
0.004850728	0.000832383	0.145	0.134	1
0.000583764	0.000886418	0.24	0.222	1
0.001575096	0.000890595	0.191	0.176	1
9.29549E-05	0.000897053	0.135	0.121	1
0.004159197	0.000913423	0.115	0.105	1
0.004211383	0.000916571	0.202	0.189	1
0.018133961	0.000925768	0.223	0.211	1
0.055097993	0.000936865	0.236	0.224	1
0.071354462	0.000954773	0.122	0.116	1
0.002960846	0.000961547	0.3	0.281	1
0.065585262	0.000968153	0.144	0.136	1
0.363283113	0.000983838	0.487	0.473	1
0.288360127	0.001003913	0.105	0.101	1
0.760125099	0.001019701	0.792	0.783	1
0.111209415	0.00106109	0.295	0.283	1
0.001844193	0.001061836	0.148	0.136	1
0.082465329	0.001082379	0.122	0.115	1
0.001724566	0.001087791	0.114	0.104	1
0.016772775	0.001137321	0.134	0.125	1
0.001668553	0.001183607	0.119	0.109	1
0.016457069	0.001205925	0.127	0.118	1
0.586440067	0.001216796	0.462	0.449	1
0.064680511	0.001236682	0.106	0.1	1
0.001301099	0.001243236	0.325	0.302	1
0.000725353	0.001245713	0.108	0.097	1
0.00125626	0.001261238	0.257	0.239	1
0.223278978	0.001280521	0.202	0.196	1
0.099076213	0.001302901	0.205	0.196	1
0.000725933	0.001311692	0.149	0.136	1
8.42799E-05	0.001313191	0.285	0.263	1
0.140702819	0.00131974	0.173	0.166	1
0.477689397	0.001319764	0.124	0.121	1
0.044440211	0.001319856	0.139	0.131	1
0.058531957	0.001351422	0.127	0.12	1
0.03085107	0.001378136	0.167	0.157	1
0.019695615	0.00138369	0.148	0.139	1
0.053447326	0.001417368	0.232	0.222	1
0.016641347	0.001421841	0.102	0.094	1
0.068424778	0.001461059	0.111	0.105	1
0.289969502	0.001466771	0.543	0.53	1
0.037872854	0.001498226	0.134	0.126	1
0.70443149	0.001514658	0.156	0.153	1
0.00255792	0.001550815	0.14	0.129	1
0.003698938	0.001615251	0.17	0.158	1
0.061116149	0.001642986	0.104	0.098	1
0.286090722	0.001647884	0.401	0.388	1
0.032823267	0.001663719	0.128	0.12	1
0.029171592	0.001715105	0.15	0.141	1

0.104836454	0.001720916	0.35	0.336	1
0.119735108	0.001731584	0.201	0.193	1
0.773991877	0.001741315	0.603	0.59	1
0.027786519	0.001744473	0.117	0.109	1
0.009463328	0.00174869	0.144	0.134	1
0.000935681	0.001749087	0.16	0.147	1
0.002032525	0.001754159	0.1	0.09	1
0.013521901	0.001764223	0.122	0.113	1
0.247915933	0.001765239	0.63	0.62	1
0.109325432	0.001771324	0.117	0.111	1
0.001815764	0.001794673	0.157	0.145	1
4.4159E-05	0.001798701	0.239	0.218	1
0.541410593	0.001813403	0.111	0.109	1
0.007485963	0.001820483	0.296	0.278	1
0.028224298	0.001826162	0.117	0.11	1
0.000917393	0.00183027	0.101	0.09	1
0.034705987	0.001878233	0.103	0.096	1
0.035533914	0.00188727	0.177	0.167	1
0.006586771	0.001905758	0.179	0.167	1
0.158696228	0.001913849	0.149	0.143	1
0.033590335	0.001922276	0.173	0.164	1
0.023425037	0.001951345	0.108	0.1	1
0.01476406	0.00199588	0.107	0.1	1
0.031422489	0.001997913	0.113	0.105	1
0.008554156	0.00205233	0.129	0.12	1
0.413352742	0.002101868	0.189	0.184	1
0.008583044	0.002165532	0.118	0.109	1
0.145870636	0.002169081	0.148	0.142	1
0.004257918	0.002178972	0.108	0.099	1
0.10141365	0.002180184	0.132	0.125	1
0.06183481	0.002206692	0.189	0.18	1
0.000343677	0.002210096	0.162	0.148	1
0.03325639	0.002211732	0.133	0.124	1
0.050491695	0.002216262	0.248	0.236	1
0.014227953	0.002250292	0.191	0.179	1
0.721820364	0.002257557	0.234	0.234	1
0.016061652	0.002258975	0.201	0.189	1
0.008003095	0.002282914	0.225	0.211	1
0.00424443	0.002297623	0.17	0.158	1
0.003902582	0.002327241	0.136	0.125	1
0.389395886	0.002335132	0.219	0.213	1
0.320952948	0.002359903	0.677	0.662	1
0.001581731	0.002371912	0.122	0.111	1
0.002734131	0.002398655	0.123	0.112	1
0.003864758	0.002401937	0.199	0.185	1
0.015521517	0.002433169	0.138	0.128	1
0.535017715	0.002441875	0.136	0.133	1
0.068946937	0.002444159	0.224	0.214	1
0.026275319	0.002497592	0.265	0.251	1
0.020459806	0.002521073	0.14	0.131	1

0.124479983	0.0025551	0.108	0.102	1
0.005814969	0.002585837	0.105	0.096	1
0.253613999	0.002586227	0.429	0.416	1
0.004684052	0.002589131	0.1	0.091	1
0.378882857	0.002610163	0.231	0.225	1
0.362425907	0.002620481	0.105	0.102	1
0.002307285	0.002623775	0.186	0.172	1
0.353442517	0.002710079	0.138	0.133	1
0.006182685	0.002713994	0.229	0.215	1
0.022150698	0.002742293	0.27	0.256	1
0.001864111	0.002755853	0.104	0.094	1
0.263569048	0.002775431	0.635	0.617	1
0.000511057	0.002789488	0.12	0.108	1
0.001776306	0.002804265	0.193	0.178	1
0.002283024	0.002817171	0.214	0.198	1
0.063876551	0.002822402	0.234	0.223	1
0.117513921	0.002825136	0.256	0.246	1
0.02414887	0.002834754	0.117	0.109	1
0.110203191	0.002845761	0.122	0.116	1
0.061402822	0.00288724	0.115	0.109	1
0.007521483	0.002896886	0.152	0.141	1
0.042748534	0.002913981	0.428	0.411	1
0.008133135	0.002949742	0.152	0.142	1
7.80729E-05	0.00308942	0.138	0.123	1
0.903375996	0.003090383	0.327	0.325	1
0.007744073	0.003154618	0.113	0.104	1
0.001902471	0.003160522	0.135	0.124	1
0.060829847	0.003174751	0.112	0.105	1
0.100593917	0.003184791	0.111	0.105	1
0.172601435	0.003190758	0.138	0.143	1
0.048859323	0.003225233	0.141	0.133	1
0.005686365	0.003255161	0.176	0.164	1
0.015948575	0.003261451	0.128	0.12	1
0.245982408	0.003288865	0.907	0.902	1
0.186577672	0.003296943	0.33	0.319	1
0.156512693	0.003297828	0.797	0.797	1
0.021190807	0.003302876	0.248	0.234	1
0.001359134	0.003313886	0.105	0.095	1
0.010954447	0.003325465	0.24	0.226	1
0.032446162	0.003340016	0.171	0.162	1
0.000609824	0.003348323	0.132	0.12	1
0.000116897	0.003353155	0.208	0.19	1
0.031469081	0.003365435	0.183	0.173	1
0.027439149	0.003392396	0.148	0.139	1
0.199542848	0.00340999	0.726	0.714	1
0.030940789	0.003429392	0.404	0.384	1
0.68340265	0.003436378	0.175	0.173	1
0.983625403	0.003437478	0.199	0.197	1
0.738096043	0.003450022	0.187	0.184	1
0.015743934	0.003455304	0.143	0.133	1

0.000176959	0.003458779	0.127	0.113	1
0.000250282	0.003476699	0.185	0.168	1
0.335618617	0.003495242	0.33	0.32	1
0.06556788	0.003513863	0.312	0.299	1
0.132334746	0.003521476	0.162	0.156	1
0.013384516	0.003559635	0.124	0.115	1
0.077964825	0.003559769	0.374	0.36	1
0.001583966	0.003561412	0.188	0.173	1
0.00017547	0.003586399	0.174	0.158	1
0.001296904	0.003644384	0.109	0.098	1
0.208121402	0.003659875	0.316	0.305	1
9.58447E-05	0.003684971	0.129	0.115	1
0.005755978	0.00369543	0.291	0.274	1
0.85752982	0.003709785	0.104	0.104	1
0.139065502	0.003710748	0.164	0.157	1
0.934154563	0.003771297	0.413	0.405	1
0.212658686	0.00379413	0.189	0.184	1
0.125032912	0.003794295	0.114	0.109	1
0.002130287	0.003801217	0.145	0.133	1
0.177315428	0.003824939	0.149	0.144	1
0.001757385	0.003827238	0.199	0.184	1
0.350354522	0.003828214	0.254	0.247	1
0.012059833	0.003911049	0.185	0.174	1
0.209937483	0.003931736	0.128	0.123	1
0.004786141	0.00393968	0.196	0.183	1
0.604686685	0.003954932	0.613	0.613	1
0.000241462	0.003966624	0.419	0.392	1
0.006634501	0.003977374	0.129	0.119	1
0.015683558	0.003985584	0.146	0.136	1
0.098123936	0.004012678	0.278	0.268	1
0.001461449	0.004045824	0.448	0.421	1
0.354212078	0.004057898	0.319	0.309	1
0.032296499	0.004073357	0.353	0.335	1
0.062924086	0.004125973	0.126	0.119	1
0.003843369	0.004160144	0.108	0.098	1
0.146461837	0.004169031	0.225	0.217	1
0.97734917	0.004184476	0.896	0.886	1
0.484335514	0.004216697	0.273	0.267	1
0.071030231	0.004220566	0.103	0.097	1
0.001864133	0.004232954	0.226	0.211	1
0.002196027	0.004238751	0.261	0.244	1
0.005439866	0.004242112	0.189	0.176	1
0.0034693	0.004295822	0.191	0.177	1
0.947460827	0.00430965	0.576	0.566	1
0.002171722	0.004327033	0.177	0.164	1
0.058057497	0.004342678	0.103	0.096	1
0.615649027	0.004376811	0.108	0.105	1
0.003157908	0.004383206	0.177	0.164	1
0.004251015	0.00438887	0.23	0.215	1
0.02380733	0.004397734	0.163	0.154	1

0.31556434	0.004407808	0.162	0.158	1
0.007339712	0.004413086	0.201	0.188	1
0.015785522	0.00441489	0.111	0.103	1
0.005880461	0.004456059	0.104	0.095	1
0.001625025	0.004460209	0.114	0.103	1
0.094788625	0.004506216	0.116	0.111	1
0.001005939	0.004535805	0.167	0.154	1
0.215384239	0.004542165	0.114	0.118	1
0.0086086	0.004551069	0.194	0.182	1
0.637557324	0.004564048	0.165	0.162	1
0.031789449	0.004571646	0.349	0.333	1
0.003933263	0.004579115	0.285	0.267	1
0.004483243	0.004587621	0.118	0.108	1
0.097859155	0.004591365	0.236	0.226	1
0.088686334	0.004591826	0.516	0.497	1
0.000874237	0.004593403	0.127	0.116	1
0.045032595	0.004601433	0.1	0.094	1
0.019036442	0.00461	0.137	0.128	1
0.003778951	0.004673468	0.185	0.172	1
0.073884457	0.004736827	0.101	0.096	1
0.165281493	0.004742565	0.1	0.095	1
0.979051045	0.004766628	0.233	0.232	1
0.009888103	0.004796753	0.322	0.303	1
0.123982059	0.004807761	0.196	0.188	1
0.006758042	0.004840704	0.177	0.164	1
0.034578953	0.004849032	0.202	0.192	1
0.009044802	0.004852855	0.161	0.15	1
0.00082303	0.004854273	0.176	0.162	1
0.066075398	0.004857003	0.236	0.226	1
0.085287101	0.004893402	0.214	0.204	1
0.007603448	0.004928357	0.106	0.098	1
0.365650782	0.004931814	0.233	0.227	1
0.400966346	0.004932326	0.267	0.26	1
0.006176944	0.004946501	0.131	0.122	1
0.000457069	0.004975198	0.173	0.158	1
7.48629E-05	0.004976871	0.234	0.214	1
0.102017246	0.005066447	0.169	0.161	1
0.208588126	0.005110721	0.115	0.111	1
0.006025938	0.005130672	0.161	0.15	1
0.017393112	0.005131123	0.239	0.226	1
0.012632864	0.005157955	0.379	0.359	1
0.014459869	0.005171815	0.133	0.124	1
0.060816222	0.005191807	0.125	0.118	1
0.118945376	0.005193712	0.319	0.308	1
0.000163094	0.005224365	0.124	0.111	1
0.003598779	0.005271912	0.117	0.107	1
0.001710574	0.005290872	0.295	0.276	1
0.020129772	0.005369311	0.107	0.1	1
0.0068581	0.005392559	0.26	0.244	1
0.000199549	0.005393812	0.182	0.167	1

0.031331919	0.005399088	0.114	0.107	1
0.000798274	0.005405746	0.136	0.124	1
0.000193933	0.00544029	0.139	0.126	1
0.002260834	0.005446136	0.107	0.097	1
0.001465286	0.005503263	0.101	0.092	1
0.176804916	0.005511407	0.192	0.185	1
0.044906971	0.005524503	0.228	0.217	1
0.002323637	0.005530532	0.208	0.193	1
0.731864708	0.005624637	0.223	0.219	1
0.034327413	0.00562594	0.106	0.099	1
0.008399522	0.005634255	0.125	0.116	1
0.028169308	0.005635645	0.129	0.121	1
0.097933725	0.005657747	0.106	0.1	1
4.31861E-05	0.005665214	0.153	0.137	1
0.104314219	0.005673918	0.152	0.144	1
0.123347264	0.005681143	0.146	0.14	1
0.415634795	0.005688606	0.184	0.18	1
0.192133467	0.005765127	0.11	0.105	1
0.055759948	0.005772781	0.461	0.444	1
0.072438529	0.005807391	0.109	0.103	1
0.002352469	0.005815848	0.119	0.109	1
0.005289708	0.005868107	0.322	0.303	1
0.002452143	0.005880923	0.172	0.159	1
0.092383115	0.005953428	0.14	0.133	1
0.005283559	0.005954758	0.391	0.37	1
0.251953221	0.005957313	0.193	0.187	1
0.003739723	0.005958008	0.246	0.231	1
0.009195572	0.005961681	0.143	0.133	1
0.031119079	0.005968257	0.252	0.241	1
0.000443875	0.005992417	0.204	0.188	1
0.004577906	0.006001006	0.126	0.116	1
0.000195679	0.006096811	0.182	0.166	1
0.933707084	0.006166389	0.103	0.102	1
0.053679143	0.006174769	0.167	0.158	1
0.002960604	0.006188721	0.264	0.246	1
0.038736642	0.006192385	0.292	0.279	1
0.00443298	0.006193418	0.183	0.17	1
0.006221635	0.006218649	0.149	0.138	1
0.391329648	0.00622763	0.193	0.189	1
0.001349902	0.006260199	0.251	0.233	1
0.113413621	0.006270185	0.198	0.189	1
0.394906167	0.006285416	0.132	0.129	1
0.049379166	0.006311168	0.286	0.272	1
0.000243502	0.006333851	0.146	0.132	1
0.006731749	0.006338982	0.266	0.25	1
0.268738826	0.006349377	0.164	0.159	1
0.010682367	0.006358506	0.383	0.363	1
0.074694584	0.006359751	0.344	0.331	1
0.008593707	0.006368296	0.165	0.154	1
0.000272194	0.006382572	0.109	0.097	1

0.001125569	0.006427018	0.182	0.168	1
0.013403733	0.006438186	0.27	0.255	1
0.000674247	0.006442931	0.14	0.128	1
0.1726413	0.006496888	0.113	0.108	1
0.002441184	0.006525482	0.115	0.105	1
0.102511156	0.006575993	0.111	0.105	1
0.004169579	0.006592572	0.139	0.128	1
0.000336231	0.006599102	0.128	0.115	1
0.00268355	0.006660728	0.115	0.105	1
0.005877126	0.006729296	0.162	0.15	1
0.023157912	0.006731841	0.192	0.182	1
0.013657534	0.006736498	0.241	0.226	1
0.018644565	0.006762646	0.196	0.185	1
0.086251517	0.00677397	0.111	0.105	1
0.047759037	0.006824341	0.195	0.185	1
0.00157958	0.006830273	0.127	0.116	1
0.044293061	0.006841594	0.107	0.1	1
0.003118682	0.006883894	0.134	0.123	1
0.01412685	0.006888716	0.134	0.125	1
0.054598269	0.006904497	0.105	0.099	1
0.007817752	0.006919555	0.177	0.165	1
0.647973404	0.006928055	0.269	0.269	1
0.016139809	0.006950617	0.153	0.143	1
0.022959687	0.006963663	0.124	0.116	1
0.044506319	0.006972705	0.258	0.245	1
0.006553543	0.007003598	0.126	0.116	1
0.006839288	0.007011879	0.268	0.253	1
0.086371581	0.007012217	0.183	0.175	1
0.002605121	0.007077575	0.19	0.175	1
0.00315147	0.007115242	0.154	0.142	1
0.000774499	0.007138448	0.22	0.203	1
0.204179323	0.007168608	0.127	0.132	1
0.009260851	0.00723641	0.248	0.233	1
0.902938011	0.007249322	0.427	0.42	1
0.09236427	0.007272059	0.553	0.533	1
0.007737257	0.00727978	0.114	0.105	1
0.000189665	0.007288484	0.109	0.097	1
0.004803189	0.007310529	0.16	0.148	1
0.091875666	0.007313769	0.114	0.109	1
0.935133549	0.007317996	0.172	0.171	1
0.001745939	0.007322575	0.189	0.175	1
0.00558288	0.007341432	0.305	0.288	1
0.019506323	0.007412266	0.122	0.114	1
0.428792889	0.007427742	0.127	0.129	1
0.430203149	0.007428808	0.156	0.152	1
0.27728685	0.007474822	0.637	0.62	1
0.006708112	0.007499648	0.104	0.096	1
0.093250029	0.007539431	0.109	0.103	1
0.031895662	0.007567083	0.369	0.353	1
0.004256941	0.00758995	0.339	0.319	1

0.046042562	0.007613028	0.767	0.759	1
0.097641654	0.00761639	0.206	0.197	1
0.51087109	0.007647712	0.29	0.284	1
0.001261588	0.007651641	0.105	0.095	1
0.01619454	0.007656354	0.1	0.092	1
0.001174313	0.007689153	0.21	0.194	1
0.001490433	0.007693251	0.264	0.247	1
0.00115493	0.007693835	0.106	0.096	1
0.041395986	0.007695811	0.141	0.133	1
0.002607474	0.007721949	0.13	0.119	1
0.000409172	0.007768995	0.184	0.169	1
0.197134473	0.007864493	0.338	0.326	1
0.025036137	0.007889648	0.231	0.219	1
0.405623308	0.007900253	0.113	0.11	1
0.288919105	0.007904885	0.166	0.161	1
0.028605547	0.007932677	0.449	0.429	1
0.538382152	0.007955471	0.151	0.148	1
0.044158263	0.007968451	0.281	0.268	1
0.0006171	0.00798732	0.132	0.12	1
0.002794561	0.007991361	0.105	0.096	1
6.81059E-05	0.008032793	0.172	0.156	1
0.001168731	0.00804413	0.148	0.136	1
0.539534905	0.008087893	0.141	0.139	1
0.001034214	0.008091961	0.219	0.203	1
0.001416544	0.008096863	0.255	0.238	1
0.071757422	0.008134993	0.13	0.123	1
0.482897405	0.008139066	0.682	0.676	1
0.100355764	0.008141872	0.121	0.116	1
0.392490622	0.008169001	0.186	0.181	1
0.738582625	0.008211177	0.241	0.238	1
0.003116284	0.008257458	0.106	0.096	1
0.09202041	0.00827066	0.111	0.106	1
0.000685599	0.008325695	0.178	0.163	1
0.120365582	0.008334009	0.354	0.341	1
0.000533422	0.00833976	0.234	0.216	1
0.000496387	0.008348337	0.15	0.137	1
0.011055065	0.008360032	0.315	0.298	1
0.219492614	0.008390588	0.264	0.255	1
0.925277345	0.008411657	0.121	0.12	1
0.001315631	0.008417955	0.257	0.239	1
0.420728922	0.008470721	0.252	0.246	1
0.012062327	0.008512416	0.163	0.152	1
0.002461036	0.008513925	0.123	0.112	1
0.000757079	0.008529445	0.12	0.108	1
0.981593811	0.008554907	0.337	0.331	1
0.000278752	0.008575351	0.262	0.242	1
0.198612019	0.008579909	0.167	0.161	1
0.002730448	0.0085935	0.137	0.126	1
0.000603208	0.008596836	0.231	0.213	1
0.155401913	0.008606024	0.194	0.186	1

0.016245737	0.008610906	0.204	0.193	1
0.072446021	0.008614709	0.551	0.533	1
0.01061768	0.008628716	0.121	0.113	1
0.000403734	0.008648859	0.197	0.182	1
0.021767274	0.0086522	0.137	0.128	1
0.001246773	0.00866381	0.137	0.125	1
0.004009223	0.008677418	0.373	0.352	1
0.004512661	0.008694752	0.226	0.212	1
0.002326429	0.008701393	0.299	0.28	1
0.095032928	0.008723633	0.203	0.195	1
0.000157863	0.008771054	0.1	0.089	1
0.001877405	0.008786491	0.114	0.104	1
0.308798221	0.00880981	0.359	0.349	1
6.36821E-05	0.008816626	0.126	0.112	1
0.006546843	0.00887192	0.307	0.29	1
0.098067696	0.008879022	0.519	0.501	1
0.146825238	0.008887324	0.216	0.208	1
0.073011448	0.008914589	0.523	0.502	1
0.000469526	0.008926987	0.189	0.174	1
0.008051974	0.008950291	0.138	0.128	1
0.002005076	0.008950766	0.205	0.19	1
0.0012725	0.008998522	0.214	0.199	1
0.058919012	0.009004023	0.24	0.229	1
0.011857223	0.009006218	0.105	0.097	1
0.007256553	0.009023127	0.182	0.171	1
0.001233315	0.009040179	0.109	0.099	1
0.001051103	0.00910316	0.185	0.171	1
0.013365254	0.009109615	0.247	0.234	1
0.041107917	0.009118925	0.518	0.501	1
0.033826855	0.009155279	0.129	0.121	1
0.001150826	0.009182259	0.181	0.166	1
0.000171763	0.009187797	0.229	0.211	1
0.005547991	0.009195771	0.278	0.262	1
0.063637799	0.009238581	0.112	0.105	1
0.011338106	0.009253985	0.212	0.199	1
0.04995575	0.009257751	0.452	0.433	1
0.00872935	0.009274886	0.137	0.127	1
0.147726749	0.009329674	0.101	0.096	1
0.000364669	0.00936264	0.113	0.101	1
0.069076809	0.009390372	0.167	0.159	1
0.021854371	0.00940543	0.251	0.238	1
0.001044513	0.009412913	0.158	0.145	1
0.037250526	0.009414757	0.456	0.439	1
0.044933218	0.009458315	0.888	0.882	1
0.000282146	0.009458769	0.105	0.094	1
0.031085361	0.009464617	0.127	0.119	1
0.029104979	0.009486603	0.129	0.121	1
0.000453498	0.009530594	0.247	0.229	1
0.112311461	0.009557764	0.211	0.202	1
0.000505037	0.009597306	0.159	0.146	1

0.000275121	0.009603324	0.191	0.175	1
0.0179732	0.009615758	0.116	0.108	1
0.003050036	0.009632018	0.195	0.181	1
0.001715916	0.009642566	0.212	0.197	1
0.000386992	0.009678715	0.202	0.186	1
0.010683786	0.009721677	0.12	0.111	1
0.006431922	0.009733761	0.319	0.299	1
0.000698062	0.009753759	0.167	0.153	1
0.149430441	0.009756403	0.121	0.116	1
0.008993527	0.009768321	0.196	0.183	1
0.043561051	0.00977941	0.142	0.134	1
0.007319268	0.009811583	0.287	0.271	1
0.000767566	0.00983563	0.105	0.095	1
0.05878247	0.009875006	0.1	0.094	1
0.06619323	0.009930985	0.386	0.372	1
0.005373146	0.00995289	0.207	0.194	1
0.000957748	0.00995437	0.11	0.099	1
9.07466E-05	0.009961776	0.152	0.137	1
0.008384522	0.00997381	0.181	0.17	1
0.02390056	0.01002246	0.281	0.267	1
0.001535415	0.010033588	0.304	0.285	1
0.000129084	0.010057196	0.148	0.134	1
0.00014683	0.01008644	0.151	0.136	1
0.686274831	0.010093656	0.176	0.174	1
0.000325292	0.010202218	0.131	0.118	1
0.020633091	0.010226706	0.167	0.158	1
0.255112082	0.010239219	0.196	0.191	1
0.122138157	0.010246083	0.291	0.28	1
6.59427E-05	0.010324814	0.116	0.103	1
0.008274708	0.010342111	0.389	0.369	1
0.004414227	0.010366634	0.184	0.171	1
0.027130179	0.010366849	0.504	0.483	1
0.00053235	0.01037952	0.287	0.267	1
0.05431768	0.010391551	0.297	0.283	1
7.41894E-05	0.010463969	0.183	0.167	1
0.000324403	0.010492511	0.126	0.113	1
0.020539144	0.010526355	0.147	0.138	1
0.000181694	0.010532001	0.1	0.089	1
0.000636773	0.010561458	0.294	0.275	1
0.000304623	0.010564857	0.198	0.182	1
0.022659052	0.010617119	0.187	0.177	1
0.000664104	0.010659781	0.208	0.193	1
0.213785863	0.010666004	0.142	0.137	1
0.000281935	0.010672784	0.114	0.103	1
0.024246151	0.01067918	0.162	0.152	1
0.001824307	0.010691292	0.185	0.171	1
0.06019263	0.01069586	0.107	0.101	1
0.000129439	0.010755851	0.105	0.094	1
0.007085356	0.010765696	0.148	0.138	1
0.244663599	0.010767156	0.206	0.201	1

0.000877829	0.010778993	0.149	0.136	1
0.001614211	0.010784077	0.317	0.295	1
0.000534512	0.010841201	0.124	0.112	1
0.000897091	0.010860595	0.188	0.173	1
0.057456715	0.010868624	0.119	0.112	1
0.597036768	0.010882441	0.127	0.125	1
0.002145365	0.010917042	0.252	0.235	1
0.007152058	0.010942597	0.472	0.453	1
0.002339313	0.0109654	0.135	0.124	1
0.007017961	0.010965807	0.159	0.148	1
0.002015531	0.011007131	0.161	0.148	1
0.008581056	0.01105614	0.125	0.115	1
0.000136499	0.011059742	0.144	0.13	1
0.000279139	0.011074264	0.183	0.167	1
0.25864656	0.011077122	0.14	0.135	1
0.000168938	0.011178653	0.163	0.148	1
0.266108145	0.011189098	0.152	0.147	1
0.00204555	0.011244113	0.218	0.203	1
0.007295006	0.011265555	0.12	0.11	1
0.006902243	0.011266673	0.445	0.424	1
0.013817597	0.01132306	0.12	0.111	1
0.000583202	0.011378813	0.282	0.262	1
0.005915172	0.011414162	0.18	0.168	1
0.552520834	0.011416131	0.137	0.135	1
0.020634982	0.011446682	0.133	0.124	1
0.001033891	0.011557621	0.113	0.102	1
0.256821893	0.011565111	0.119	0.115	1
0.00039139	0.01158946	0.107	0.096	1
0.005959282	0.011595224	0.213	0.2	1
0.000286591	0.011610067	0.156	0.142	1
0.00241423	0.011647857	0.171	0.159	1
0.453673709	0.011668487	0.334	0.327	1
0.00359418	0.011673201	0.102	0.093	1
0.907752238	0.011674641	0.323	0.318	1
6.20479E-05	0.01170663	0.19	0.173	1
0.001470982	0.011807433	0.117	0.107	1
0.000628655	0.011826937	0.137	0.125	1
0.002099465	0.011832137	0.387	0.364	1
0.001183752	0.011869905	0.268	0.249	1
0.003796487	0.011902235	0.152	0.14	1
0.013616666	0.011916317	0.229	0.216	1
0.002417348	0.011924624	0.226	0.211	1
0.000612501	0.011950625	0.296	0.274	1
0.062668799	0.012026698	0.528	0.508	1
0.009054099	0.012054366	0.102	0.094	1
0.073689496	0.012065449	0.325	0.311	1
0.065992214	0.012121772	0.119	0.112	1
7.26016E-05	0.012221834	0.177	0.16	1
0.153484595	0.012230211	0.235	0.227	1
0.000289533	0.012276625	0.118	0.106	1

0.000317299	0.012309291	0.377	0.351	1
0.001142658	0.012309355	0.109	0.099	1
0.00092098	0.012334223	0.167	0.154	1
0.207441376	0.012401679	0.222	0.215	1
0.000218747	0.012414467	0.188	0.172	1
0.010746004	0.012419939	0.163	0.153	1
0.045641757	0.012422812	0.228	0.217	1
0.002490408	0.012465225	0.135	0.124	1
0.007224164	0.012488359	0.185	0.174	1
0.000216317	0.012503496	0.153	0.139	1
0.30404073	0.01254408	0.196	0.19	1
0.309869742	0.012551734	0.104	0.101	1
0.00034379	0.012616528	0.296	0.275	1
0.429640485	0.012678853	0.424	0.413	1
7.30149E-05	0.012683945	0.105	0.093	1
0.000184989	0.012695641	0.115	0.103	1
0.020203536	0.012702184	0.19	0.179	1
0.423675363	0.012724593	0.404	0.392	1
0.02157851	0.012777177	0.262	0.249	1
0.031582572	0.012789711	0.466	0.446	1
0.003390853	0.01280214	0.246	0.231	1
0.000847253	0.012820478	0.143	0.131	1
0.059847753	0.012861669	0.369	0.353	1
8.41401E-05	0.012863271	0.114	0.101	1
0.002639273	0.012909185	0.17	0.158	1
0.010773266	0.012910951	0.102	0.094	1
0.1188778	0.012951488	0.493	0.476	1
0.004640007	0.01295406	0.232	0.217	1
0.024679588	0.01297129	0.241	0.23	1
0.000185739	0.012983709	0.246	0.226	1
0.045335805	0.013013575	0.151	0.142	1
5.24537E-05	0.01301401	0.264	0.243	1
0.000133493	0.013026478	0.245	0.227	1
0.000406473	0.013092924	0.265	0.246	1
0.048158349	0.013094694	0.321	0.31	1
0.002917251	0.013117679	0.157	0.145	1
0.029071007	0.013117871	0.27	0.257	1
0.003913856	0.013185644	0.1	0.091	1
0.058229814	0.013195021	0.288	0.276	1
0.187376667	0.01321023	0.117	0.112	1
0.017915099	0.013266957	0.499	0.478	1
0.010254558	0.013303195	0.137	0.128	1
0.156408587	0.013306123	0.688	0.671	1
0.00848228	0.013322301	0.149	0.139	1
0.007978688	0.013357399	0.109	0.1	1
0.075892512	0.013358401	0.144	0.137	1
8.16508E-05	0.013396678	0.104	0.092	1
0.000406304	0.01342679	0.155	0.141	1
0.003372745	0.013453986	0.112	0.102	1
0.000249703	0.013467646	0.15	0.136	1

0.007045504	0.01347391	0.217	0.204	1
0.021997747	0.013489086	0.247	0.234	1
0.025139108	0.01349324	0.134	0.126	1
0.19210451	0.013504056	0.502	0.487	1
0.303512316	0.013508226	0.592	0.576	1
0.00295374	0.013515451	0.115	0.105	1
0.502553366	0.013584227	0.156	0.153	1
0.013550279	0.013645964	0.113	0.105	1
0.059332182	0.01368291	0.409	0.391	1
0.011117115	0.013689615	0.137	0.128	1
0.008080673	0.013698069	0.186	0.175	1
0.04834204	0.013740825	0.222	0.212	1
0.00036055	0.01375313	0.168	0.154	1
0.003830999	0.013763066	0.294	0.276	1
0.001802983	0.013786307	0.104	0.094	1
0.002295849	0.01378756	0.354	0.333	1
0.259983025	0.013791874	0.31	0.3	1
0.00341585	0.013799129	0.113	0.104	1
0.000230923	0.013845218	0.287	0.266	1
0.002109154	0.013867274	0.272	0.256	1
0.004896596	0.013900657	0.372	0.352	1
0.000436229	0.013908813	0.23	0.213	1
0.00204277	0.01398266	0.171	0.159	1
7.25332E-05	0.013988849	0.121	0.108	1
0.000993013	0.014058033	0.132	0.12	1
0.000465113	0.01405879	0.13	0.118	1
0.001946003	0.014065422	0.139	0.128	1
0.083477596	0.014092458	0.137	0.13	1
0.029392706	0.014100153	0.328	0.313	1
0.012772584	0.014126401	0.148	0.139	1
0.004999205	0.014127667	0.467	0.443	1
0.004339749	0.014174596	0.361	0.342	1
0.00115862	0.01417727	0.264	0.246	1
0.003474283	0.014195188	0.138	0.128	1
0.000293474	0.014232413	0.223	0.206	1
0.000694243	0.014241738	0.204	0.188	1
0.005029255	0.014245815	0.159	0.148	1
0.001963806	0.014259835	0.247	0.23	1
0.001400168	0.014283673	0.224	0.208	1
0.000700639	0.014309204	0.148	0.135	1
0.005788848	0.014312425	0.186	0.174	1
0.002888123	0.014334892	0.438	0.417	1
0.009442654	0.014362074	0.105	0.097	1
0.016154607	0.014370169	0.128	0.12	1
0.000165841	0.014399517	0.259	0.24	1
8.5664E-05	0.014427996	0.197	0.18	1
0.007001628	0.014460153	0.106	0.097	1
0.003113048	0.014467291	0.328	0.308	1
0.004072918	0.014472744	0.135	0.124	1
0.004186486	0.014492219	0.344	0.325	1

0.000344997	0.014541818	0.384	0.359	1
0.045925485	0.014615636	0.148	0.141	1
0.01745645	0.014616025	0.22	0.208	1
0.020286963	0.014630916	0.576	0.557	1
0.000106925	0.014637077	0.132	0.119	1
0.000170818	0.014643083	0.171	0.156	1
0.021230802	0.014652872	0.16	0.151	1
0.051531749	0.014700554	0.164	0.155	1
0.000427057	0.014745341	0.25	0.231	1
0.026965689	0.014747587	0.19	0.18	1
0.053864884	0.014770381	0.521	0.503	1
0.010010408	0.014776059	0.187	0.176	1
0.000233001	0.014814944	0.223	0.206	1
0.000119212	0.014823576	0.2	0.183	1
0.003178185	0.014843991	0.168	0.156	1
0.010798566	0.014850611	0.244	0.231	1
5.7545E-05	0.014870972	0.253	0.233	1
0.009137398	0.014914981	0.353	0.335	1
0.078838032	0.014924732	0.248	0.238	1
9.0221E-05	0.014925958	0.182	0.166	1
0.000522688	0.01499008	0.25	0.232	1
4.25996E-05	0.015028218	0.265	0.244	1
0.012239826	0.015058827	0.135	0.126	1
0.112924936	0.015092927	0.139	0.132	1
9.86204E-05	0.015142151	0.171	0.155	1
0.004032011	0.01514494	0.395	0.373	1
0.777801758	0.015145182	0.439	0.436	1
0.003939432	0.015171042	0.127	0.117	1
0.112977189	0.01517542	0.119	0.113	1
0.000350403	0.015226094	0.135	0.122	1
0.033796893	0.015231981	0.318	0.304	1
0.000534321	0.015269722	0.317	0.295	1
0.049312135	0.015285643	0.384	0.369	1
0.00742934	0.015307905	0.172	0.16	1
0.002665794	0.015314993	0.16	0.148	1
0.131369419	0.015317958	0.825	0.815	1
0.013489551	0.015350047	0.409	0.386	1
0.025722498	0.015352428	0.587	0.574	1
0.886828241	0.015392674	0.776	0.76	1
0.000481546	0.015413988	0.473	0.445	1
5.93525E-05	0.015418054	0.139	0.125	1
0.824247851	0.015425109	0.129	0.128	1
0.00255874	0.015443015	0.33	0.31	1
0.035498149	0.015514522	0.151	0.143	1
0.002986085	0.015534821	0.12	0.11	1
0.000132199	0.015537767	0.262	0.242	1
0.118350935	0.015598583	0.393	0.378	1
0.044586394	0.015600876	0.184	0.175	1
0.006167062	0.015601519	0.334	0.316	1
0.00308968	0.015611373	0.227	0.212	1

0.004498232	0.015642587	0.139	0.128	1
0.000448025	0.015665642	0.202	0.186	1
0.039842218	0.015788025	0.118	0.111	1
0.184876694	0.015788086	0.111	0.107	1
0.00069431	0.015810227	0.174	0.159	1
0.006197814	0.015868052	0.165	0.154	1
0.021481011	0.015881838	0.29	0.277	1
0.002456532	0.015936711	0.351	0.331	1
0.011056828	0.015974644	0.746	0.724	1
0.003901304	0.015976756	0.14	0.129	1
0.000862881	0.015982442	0.156	0.143	1
0.002976194	0.015983664	0.137	0.127	1
0.418026812	0.01598394	0.166	0.168	1
0.000751493	0.01598541	0.232	0.215	1
0.002201835	0.016018137	0.11	0.1	1
0.001507611	0.016058528	0.271	0.254	1
0.00468439	0.016083628	0.117	0.108	1
8.45261E-05	0.016085633	0.248	0.229	1
0.005060026	0.01609399	0.111	0.102	1
0.000980836	0.016150142	0.419	0.395	1
0.002373509	0.016202657	0.138	0.127	1
0.000626746	0.016204068	0.238	0.22	1
0.001199224	0.016207182	0.184	0.171	1
5.44264E-05	0.016216907	0.173	0.157	1
0.407352909	0.016251481	0.129	0.125	1
0.088076254	0.016258051	0.107	0.112	1
0.001675638	0.016289861	0.302	0.284	1
0.000703537	0.016336171	0.241	0.224	1
0.000121713	0.016354848	0.174	0.158	1
0.002289675	0.016370569	0.251	0.235	1
5.37051E-05	0.016381963	0.137	0.123	1
0.003265868	0.016397471	0.379	0.358	1
0.039812674	0.016408662	0.298	0.285	1
0.377782928	0.016455889	0.663	0.659	1
0.496034433	0.016479068	0.823	0.816	1
0.00072938	0.016513817	0.438	0.415	1
0.021647987	0.016518862	0.102	0.094	1
0.10895938	0.016528641	0.12	0.115	1
0.006075541	0.016534971	0.236	0.221	1
0.007769913	0.016647658	0.131	0.122	1
0.004195035	0.016678149	0.19	0.177	1
6.72814E-05	0.016685706	0.356	0.329	1
0.006404302	0.016694835	0.454	0.435	1
0.005160842	0.016699293	0.159	0.148	1
0.001249516	0.016755286	0.16	0.148	1
0.017696072	0.016766263	0.427	0.407	1
0.001593025	0.016771665	0.375	0.354	1
0.041081135	0.016814058	0.116	0.109	1
7.93187E-05	0.016826334	0.199	0.181	1
0.322791127	0.016874178	0.324	0.317	1

0.000789688	0.016912816	0.121	0.109	1
0.00043107	0.016919232	0.304	0.284	1
0.00413932	0.016965879	0.136	0.126	1
0.000911112	0.01697638	0.24	0.223	1
0.000828318	0.01698129	0.312	0.292	1
0.001203158	0.017002951	0.174	0.16	1
0.358648454	0.017027982	0.362	0.351	1
0.015391793	0.017049433	0.162	0.152	1
0.010935501	0.017093291	0.129	0.12	1
0.012886797	0.017175132	0.225	0.213	1
0.000185124	0.017180284	0.107	0.096	1
0.009312647	0.017199284	0.164	0.154	1
0.000579187	0.017209174	0.316	0.295	1
0.000145276	0.017256375	0.143	0.129	1
0.000554189	0.017290378	0.103	0.092	1
0.011604649	0.017290986	0.364	0.346	1
0.035287605	0.017370892	0.56	0.541	1
0.001508503	0.017390658	0.91	0.904	1
0.022078324	0.017429708	0.351	0.336	1
0.29025339	0.017505473	0.105	0.102	1
0.469902772	0.01751171	0.286	0.28	1
4.05683E-05	0.017524328	0.135	0.121	1
5.51837E-05	0.017564686	0.263	0.242	1
0.000696249	0.017573445	0.186	0.172	1
0.00306754	0.017582761	0.103	0.093	1
0.000180113	0.017724793	0.264	0.244	1
0.001436098	0.017789963	0.138	0.126	1
0.004039411	0.017806822	0.269	0.252	1
0.012227573	0.017811905	0.23	0.216	1
4.59098E-05	0.017819801	0.143	0.128	1
0.028723074	0.017831832	0.186	0.176	1
4.1213E-05	0.017861721	0.224	0.204	1
4.52767E-05	0.017874402	0.115	0.102	1
5.61395E-05	0.017879064	0.234	0.214	1
0.004685721	0.017889031	0.103	0.094	1
0.037515247	0.017894226	0.123	0.116	1
0.01637387	0.017896957	0.106	0.098	1
0.00085539	0.017908631	0.193	0.178	1
0.014141618	0.017941532	0.154	0.144	1
0.243649802	0.017950484	0.37	0.36	1
0.002713812	0.017955798	0.176	0.163	1
0.040713826	0.017986863	0.12	0.113	1
0.000644884	0.018030273	0.243	0.227	1
0.000136823	0.018075614	0.242	0.223	1
0.008291012	0.018079155	0.143	0.133	1
0.049570441	0.018096398	0.367	0.352	1
0.005712229	0.018191333	0.164	0.153	1
4.96016E-05	0.018194678	0.109	0.096	1
0.000260006	0.018196104	0.301	0.28	1
0.001610121	0.018220098	0.157	0.145	1

0.130394408	0.018265643	0.475	0.46	1
0.464184163	0.018364599	0.132	0.129	1
0.001741453	0.018368698	0.881	0.874	1
0.001734512	0.018375997	0.179	0.166	1
0.000244223	0.018505799	0.167	0.152	1
0.000241269	0.018531754	0.196	0.18	1
0.100769175	0.018608332	0.516	0.498	1
0.131495185	0.018622122	0.114	0.109	1
0.000191127	0.018677629	0.128	0.115	1
4.79512E-05	0.018684077	0.151	0.136	1
0.044262063	0.0186901	0.843	0.833	1
0.003694382	0.018720354	0.271	0.256	1
0.028252099	0.018798327	0.769	0.757	1
0.003852079	0.018844953	0.101	0.092	1
0.342379712	0.018876433	0.664	0.663	1
0.022661834	0.018897779	0.1	0.093	1
0.004257957	0.01890759	0.296	0.278	1
0.186694969	0.018984666	0.176	0.17	1
0.300628257	0.019036481	0.237	0.23	1
8.49509E-05	0.019036599	0.123	0.11	1
0.001947499	0.019078929	0.405	0.383	1
0.552785494	0.019120377	0.693	0.687	1
0.003640725	0.019136452	0.185	0.172	1
0.001451325	0.019185489	0.234	0.218	1
0.000331484	0.019270373	0.208	0.192	1
0.028713669	0.019281312	0.208	0.198	1
0.000348529	0.019281947	0.132	0.119	1
0.004044742	0.019350942	0.161	0.149	1
0.001250095	0.019378652	0.333	0.313	1
0.000614197	0.019394865	0.16	0.147	1
0.04483464	0.019414149	0.165	0.157	1
0.000568061	0.019458175	0.152	0.139	1
0.006250838	0.019494737	0.207	0.195	1
0.210666511	0.019522373	0.408	0.395	1
0.058834502	0.019612889	0.224	0.214	1
0.001479051	0.019616645	0.113	0.102	1
0.119391701	0.019634441	0.229	0.221	1
0.000491996	0.019663112	0.161	0.148	1
0.000406759	0.01977518	0.141	0.128	1
0.002902537	0.019823498	0.1	0.091	1
0.000103327	0.019846219	0.149	0.134	1
0.004825327	0.019855652	0.152	0.141	1
0.000856717	0.019856104	0.107	0.097	1
8.91719E-05	0.019922247	0.215	0.197	1
0.000256469	0.020018108	0.108	0.097	1
0.202796891	0.02002071	0.185	0.181	1
0.000937354	0.020038909	0.166	0.153	1
0.005093549	0.02012647	0.124	0.115	1
0.000193138	0.020245238	0.282	0.262	1
0.004531284	0.020254351	0.469	0.445	1

0.228518719	0.02029396	0.418	0.407	1
0.000201918	0.020320239	0.361	0.336	1
0.673270607	0.020326419	0.397	0.391	1
0.00339934	0.020336674	0.128	0.118	1
0.247654593	0.02039505	0.323	0.313	1
0.054643895	0.020414791	0.349	0.335	1
0.000596595	0.020424182	0.244	0.227	1
0.005201083	0.020424837	0.147	0.137	1
0.189753871	0.020442844	0.674	0.661	1
0.008866038	0.020473721	0.104	0.096	1
0.001886863	0.02051728	0.291	0.273	1
0.078390142	0.020526397	0.473	0.456	1
0.001166299	0.020544549	0.317	0.297	1
0.009698237	0.020629489	0.38	0.36	1
8.9227E-05	0.020655846	0.116	0.103	1
0.063971381	0.02067962	0.429	0.412	1
0.003364167	0.020751173	0.143	0.132	1
0.000184224	0.020751557	0.186	0.17	1
0.019560879	0.020755901	0.101	0.094	1
0.074450375	0.020771122	0.211	0.203	1
0.000216847	0.02078702	0.127	0.114	1
0.071968729	0.020797505	0.628	0.617	1
0.000509139	0.020802382	0.422	0.395	1
0.000346068	0.020894394	0.3	0.28	1
0.186710827	0.020906484	0.136	0.131	1
0.16962001	0.020935528	0.236	0.228	1
0.001608773	0.021016814	0.453	0.428	1
0.002064024	0.021048783	0.135	0.124	1
0.000197234	0.021063508	0.302	0.281	1
0.000569152	0.021094917	0.292	0.273	1
0.304324282	0.021159037	0.17	0.166	1
0.014861554	0.021253249	0.113	0.105	1
0.000983565	0.02125816	0.264	0.245	1
0.072015684	0.021278585	0.103	0.098	1
0.148814886	0.021279755	0.155	0.149	1
0.191769286	0.02129588	0.163	0.158	1
0.01897775	0.02130569	0.127	0.119	1
0.00062246	0.021322406	0.151	0.138	1
0.002079187	0.021337205	0.223	0.209	1
0.00956397	0.021389139	0.121	0.112	1
0.00013536	0.021405414	0.109	0.097	1
0.000213448	0.02143184	0.159	0.145	1
0.000171171	0.021442183	0.182	0.166	1
0.003286702	0.021528869	0.148	0.137	1
0.000484133	0.021571406	0.173	0.159	1
0.063293803	0.021636079	0.317	0.304	1
0.02389975	0.021646157	0.462	0.443	1
0.225765299	0.021662467	0.109	0.105	1
0.000246619	0.021667738	0.23	0.212	1
0.005592171	0.021670975	0.145	0.135	1

0.011834743	0.021676985	0.462	0.443	1
0.007909422	0.021677783	0.642	0.624	1
0.001308054	0.021680837	0.102	0.092	1
0.051502798	0.021683074	0.123	0.116	1
0.401163604	0.021743055	0.171	0.167	1
0.001677888	0.021807815	0.158	0.146	1
0.061821268	0.021882116	0.763	0.744	1
0.244831864	0.021919256	0.111	0.107	1
0.000251251	0.021928763	0.168	0.153	1
0.000709434	0.021960849	0.144	0.131	1
0.01554131	0.021979443	0.259	0.246	1
0.006242493	0.021985558	0.9	0.89	1
0.000204062	0.022027262	0.136	0.123	1
0.00030908	0.02203614	0.118	0.107	1
0.000166808	0.022036667	0.1	0.088	1
0.002375707	0.022048357	0.214	0.2	1
0.000519215	0.022071291	0.191	0.176	1
0.003622244	0.02208512	0.273	0.256	1
0.001259303	0.022089064	0.12	0.11	1
0.001706885	0.022134578	0.524	0.498	1
0.014532065	0.022164041	0.272	0.259	1
0.0006135	0.022276256	0.252	0.235	1
0.148905927	0.022282608	0.102	0.098	1
0.00059382	0.022350694	0.153	0.141	1
0.031203167	0.022355446	0.998	0.998	1
0.000158709	0.0224935	0.198	0.181	1
0.000368553	0.022557557	0.127	0.115	1
0.004197441	0.022577572	0.169	0.158	1
0.001139604	0.022638087	0.494	0.468	1
0.003328038	0.022674694	0.123	0.113	1
0.268905774	0.022704714	0.333	0.323	1
0.000262469	0.022782094	0.127	0.115	1
5.57918E-05	0.022797652	0.117	0.104	1
0.002104541	0.022831327	0.104	0.095	1
0.001813841	0.022874509	0.592	0.566	1
0.000543469	0.022878147	0.259	0.24	1
0.004695314	0.022911086	0.279	0.262	1
0.112307637	0.022919726	0.162	0.155	1
0.00158628	0.022920645	0.259	0.243	1
0.000227447	0.022923202	0.166	0.151	1
0.000327239	0.022969737	0.227	0.21	1
0.000211862	0.023055434	0.108	0.097	1
0.000800474	0.023089565	0.158	0.145	1
0.055102466	0.023200333	0.113	0.107	1
0.219044681	0.023217159	0.554	0.538	1
5.37756E-05	0.023253904	0.161	0.145	1
0.000135208	0.023275267	0.133	0.12	1
0.000298433	0.023278652	0.238	0.22	1
0.000310823	0.023300052	0.1	0.089	1
0.004391401	0.023311134	0.615	0.593	1

0.013517824	0.023381331	0.128	0.119	1
0.003644518	0.023452837	0.381	0.367	1
0.036126708	0.023465197	0.201	0.191	1
0.008750549	0.023466091	0.12	0.112	1
0.00431607	0.023641368	0.352	0.333	1
0.009175132	0.023660485	0.196	0.184	1
0.003658797	0.023703602	0.52	0.5	1
0.000890212	0.023740252	0.143	0.131	1
9.46911E-05	0.023783003	0.125	0.112	1
0.056486597	0.023849893	0.462	0.447	1
0.002021151	0.023913823	0.414	0.39	1
0.005249049	0.023969199	0.37	0.352	1
0.005435134	0.023977247	0.157	0.146	1
0.02742573	0.023996047	0.164	0.154	1
0.000124266	0.023999111	0.379	0.354	1
0.010746548	0.024079204	0.28	0.265	1
7.06229E-05	0.024091287	0.201	0.183	1
0.021540024	0.024097274	0.3	0.285	1
0.000273459	0.024114346	0.11	0.099	1
0.003408985	0.024119272	0.148	0.137	1
0.004046042	0.02412157	0.459	0.436	1
0.001610865	0.024163686	0.295	0.277	1
0.015252795	0.024200821	0.195	0.184	1
0.000244712	0.024338764	0.117	0.105	1
0.236114728	0.024378599	0.635	0.623	1
0.056743133	0.024410329	0.628	0.61	1
0.000121201	0.024415448	0.317	0.294	1
0.000108402	0.02442595	0.136	0.122	1
0.02287835	0.024458369	0.377	0.361	1
0.000397272	0.024479361	0.154	0.141	1
0.014277903	0.024532259	0.232	0.219	1
0.006824442	0.02453805	0.348	0.33	1
0.000118746	0.024545019	0.101	0.089	1
0.002461284	0.024585178	0.107	0.098	1
7.63815E-05	0.024628011	0.214	0.195	1
0.262431802	0.024687668	0.469	0.458	1
6.43626E-05	0.024719898	0.235	0.216	1
0.000869088	0.024767568	0.385	0.364	1
0.002173181	0.024855593	0.41	0.389	1
0.000804279	0.024861684	0.432	0.408	1
0.037372497	0.024937087	0.442	0.424	1
0.000552025	0.024976562	0.313	0.293	1
0.031813763	0.025015173	0.63	0.617	1
0.005118713	0.025030205	0.117	0.108	1
0.001791116	0.025099825	0.174	0.161	1
0.00119715	0.025143423	0.47	0.446	1
0.001557566	0.025150085	0.107	0.097	1
5.02942E-05	0.025190736	0.281	0.259	1
0.047300572	0.025206331	0.29	0.278	1
0.004138692	0.025232324	0.283	0.267	1

0.002380108	0.025269074	0.143	0.132	1
0.035325427	0.025380896	0.22	0.21	1
0.000178365	0.025412069	0.203	0.186	1
0.000514313	0.025425537	0.239	0.222	1
0.000988175	0.025433658	0.126	0.115	1
0.002386814	0.025442852	0.657	0.635	1
0.000326969	0.025475477	0.1	0.09	1
0.000808691	0.025541707	0.112	0.101	1
0.012649755	0.025543717	0.143	0.134	1
6.64556E-05	0.025574192	0.39	0.364	1
0.016932214	0.0255976	0.409	0.391	1
0.000658669	0.025637687	0.212	0.196	1
0.041839221	0.025645136	0.861	0.846	1
0.00010776	0.025696707	0.346	0.322	1
0.00033325	0.025708583	0.176	0.162	1
0.00028182	0.025716596	0.118	0.106	1
0.000139012	0.025732261	0.251	0.232	1
0.00228828	0.025752405	0.61	0.587	1
0.001070589	0.025766094	0.305	0.286	1
6.67243E-05	0.025815257	0.235	0.215	1
0.000668021	0.02584746	0.167	0.153	1
0.296665845	0.025856087	0.305	0.308	1
0.000544215	0.025891888	0.122	0.11	1
0.003331595	0.025900619	0.551	0.532	1
0.082110212	0.02595318	0.203	0.195	1
0.000157185	0.025974423	0.25	0.231	1
0.062639829	0.025997038	0.357	0.345	1
0.001265241	0.02601084	0.17	0.157	1
0.016292793	0.026031228	0.107	0.099	1
5.1138E-05	0.026043244	0.313	0.29	1
0.008602884	0.026086837	0.234	0.222	1
0.002615004	0.026142036	0.429	0.405	1
0.00252245	0.026330626	0.831	0.82	1
0.000208213	0.026345282	0.307	0.286	1
0.007301623	0.026412544	0.107	0.099	1
0.424179307	0.026432703	0.278	0.274	1
0.003232738	0.026496071	0.694	0.678	1
0.001065562	0.02650063	0.279	0.262	1
0.020311558	0.026540423	0.224	0.213	1
0.00157029	0.026543177	0.264	0.247	1
0.004079759	0.02654582	0.13	0.119	1
0.000129377	0.026548378	0.243	0.225	1
9.6312E-05	0.026575612	0.207	0.191	1
0.000157565	0.026601522	0.115	0.103	1
0.002009228	0.026613827	0.126	0.116	1
0.002327639	0.02663189	0.116	0.106	1
0.035286257	0.026644462	0.135	0.128	1
0.000216402	0.026668976	0.143	0.13	1
0.210318573	0.026792946	0.119	0.115	1
0.040685254	0.026800718	0.514	0.495	1

0.000250224	0.026832383	0.475	0.449	1
0.002243899	0.026842957	0.253	0.238	1
0.000502853	0.026911918	0.117	0.106	1
0.025272636	0.026912644	0.372	0.357	1
0.052181202	0.027005169	0.509	0.492	1
0.004651191	0.027161545	0.963	0.959	1
0.235977046	0.027162865	0.103	0.1	1
0.00316675	0.027168278	0.271	0.255	1
0.002891508	0.027170615	0.371	0.35	1
0.000177628	0.027201396	0.273	0.253	1
0.002547514	0.02722562	0.404	0.384	1
7.43404E-05	0.027229626	0.317	0.294	1
6.8824E-05	0.027233223	0.312	0.289	1
0.000256833	0.027252985	0.228	0.21	1
0.000231959	0.027283719	0.174	0.159	1
0.840497621	0.02731575	0.65	0.641	1
0.000204512	0.027361672	0.393	0.368	1
9.86647E-05	0.027366966	0.562	0.536	1
0.000180713	0.027453358	0.546	0.518	1
0.000304129	0.027521395	0.251	0.232	1
0.013553334	0.02754102	0.198	0.187	1
0.001897879	0.027558824	0.196	0.182	1
6.49E-05	0.027649233	0.351	0.328	1
0.00801205	0.027649509	0.212	0.199	1
0.00033275	0.027679882	0.122	0.11	1
4.49544E-05	0.027685031	0.187	0.17	1
0.001156064	0.027703026	0.316	0.296	1
7.09985E-05	0.027745585	0.224	0.205	1
0.031671789	0.027800555	0.152	0.144	1
0.000419285	0.027815496	0.129	0.117	1
0.001324972	0.027835383	0.433	0.408	1
0.502484802	0.027852484	0.279	0.274	1
0.000631282	0.027862561	0.235	0.218	1
0.00029213	0.027880163	0.194	0.179	1
0.119442739	0.027892182	0.302	0.292	1
0.002770509	0.027907248	0.197	0.184	1
0.000168429	0.027908058	0.116	0.104	1
0.00164404	0.027980801	0.366	0.346	1
0.000286885	0.027989599	0.135	0.123	1
5.66526E-05	0.0279924	0.214	0.195	1
0.000286134	0.028067252	0.496	0.471	1
0.641414939	0.028069954	0.545	0.543	1
0.000465545	0.028075709	0.332	0.312	1
4.65906E-05	0.028087033	0.215	0.197	1
0.000574723	0.028097441	0.286	0.267	1
0.000170055	0.028219806	0.133	0.12	1
0.022565322	0.028245886	0.156	0.148	1
0.170021664	0.028273835	0.114	0.109	1
0.002474758	0.028418503	0.226	0.211	1
0.000940086	0.028430044	0.113	0.103	1

0.060095229	0.028483864	0.576	0.559	1
0.002330063	0.028559342	0.458	0.434	1
0.001442056	0.02866349	0.476	0.453	1
0.003483811	0.028663828	0.438	0.418	1
0.083065516	0.028801164	0.167	0.16	1
0.000349207	0.028822306	0.335	0.312	1
0.000106751	0.028865985	0.122	0.109	1
0.00296694	0.02888775	0.87	0.858	1
0.00604442	0.028943739	0.19	0.178	1
0.112479968	0.028973065	0.327	0.316	1
0.001641036	0.029039464	0.623	0.602	1
0.000685238	0.029131391	0.382	0.358	1
0.000879914	0.029159255	0.263	0.246	1
0.001183168	0.02920674	0.559	0.533	1
0.000197841	0.029253058	0.21	0.193	1
0.136578247	0.029319565	0.381	0.369	1
0.010013491	0.029389066	0.725	0.708	1
0.028285899	0.029553643	0.318	0.304	1
0.012195339	0.029563328	0.548	0.525	1
0.00088657	0.029670663	0.446	0.422	1
0.008082736	0.029675538	0.644	0.625	1
0.017534041	0.029683592	0.258	0.245	1
4.54408E-05	0.029775927	0.411	0.384	1
0.00318956	0.029807788	0.192	0.179	1
0.000264724	0.029831463	0.312	0.292	1
0.00039576	0.029843405	0.178	0.164	1
0.016824665	0.029935146	0.316	0.304	1
0.000605828	0.030042557	0.479	0.455	1
5.67478E-05	0.030141309	0.437	0.411	1
0.002235214	0.030147689	0.333	0.314	1
0.010577468	0.030221832	0.291	0.275	1
0.232089007	0.03024175	0.697	0.681	1
0.000640411	0.03026306	0.922	0.913	1
0.000795017	0.030284421	0.152	0.139	1
0.000236667	0.030312269	0.235	0.218	1
0.00044804	0.030337489	0.578	0.549	1
0.000835397	0.030467444	0.489	0.464	1
5.88006E-05	0.030492787	0.27	0.249	1
0.000332041	0.030528809	0.321	0.3	1
0.860918892	0.030584285	0.388	0.387	1
9.61914E-05	0.030619943	0.245	0.225	1
0.004877414	0.0306922	0.805	0.79	1
0.004683069	0.030775484	0.468	0.455	1
0.002500314	0.030875104	0.594	0.574	1
0.002301662	0.030916321	0.102	0.092	1
0.000308117	0.030960262	0.311	0.289	1
0.013224691	0.03100108	0.129	0.12	1
0.051918632	0.031028656	0.427	0.413	1
0.030271825	0.031167894	0.169	0.16	1
0.001498296	0.031236656	0.21	0.196	1

4.20996E-05	0.031378024	0.15	0.135	1
0.002570793	0.03140177	0.414	0.394	1
0.001053623	0.031557607	0.138	0.127	1
0.359848792	0.031586723	0.107	0.111	1
0.000187052	0.031604833	0.875	0.855	1
0.000461711	0.031609529	0.392	0.368	1
0.000248056	0.031720261	0.337	0.314	1
0.000212931	0.031760016	0.478	0.455	1
0.003389007	0.031814537	0.534	0.51	1
0.01922971	0.031884938	0.101	0.094	1
7.66459E-05	0.031902179	0.312	0.289	1
0.002108424	0.032192303	0.209	0.195	1
0.002129558	0.032448941	0.716	0.698	1
4.68437E-05	0.032839102	0.196	0.178	1
0.001170458	0.032858144	0.306	0.287	1
0.059050814	0.032866953	0.38	0.366	1
0.000564229	0.032959731	0.367	0.345	1
0.000260313	0.033051025	0.102	0.091	1
0.000289812	0.03320789	0.234	0.216	1
9.27306E-05	0.033228312	0.302	0.28	1
0.117159496	0.033244112	0.191	0.185	1
0.000141552	0.033256219	0.255	0.235	1
0.000337099	0.033366144	0.189	0.174	1
0.000489218	0.033531183	0.165	0.152	1
0.002734949	0.033542527	0.508	0.483	1
0.000117677	0.033578329	0.202	0.185	1
0.001073302	0.033581694	0.142	0.13	1
0.000197719	0.033587262	0.384	0.359	1
0.002357227	0.033704287	0.36	0.342	1
0.000225287	0.034081699	0.122	0.11	1
0.00016059	0.034256591	0.538	0.511	1
0.004705103	0.034407618	0.289	0.274	1
0.001597568	0.034426861	0.236	0.222	1
0.000184012	0.034491023	0.451	0.425	1
0.004529381	0.034516058	0.501	0.479	1
0.030275228	0.034533997	0.235	0.224	1
0.000378336	0.034616133	0.232	0.215	1
0.000206617	0.034713452	0.142	0.129	1
6.35346E-05	0.034983047	0.312	0.29	1
0.000191976	0.035027685	0.165	0.151	1
0.001248833	0.035131044	0.139	0.128	1
4.41822E-05	0.035220607	0.445	0.417	1
0.037106447	0.035240845	0.106	0.1	1
0.009064768	0.035285097	0.254	0.24	1
0.000395386	0.035285922	0.191	0.175	1
0.002527746	0.035290476	0.259	0.242	1
0.001679443	0.035296291	0.18	0.167	1
0.000377879	0.035332955	0.456	0.431	1
0.00022114	0.035365915	0.765	0.756	1
0.000102606	0.035499819	0.134	0.121	1

5.92477E-05	0.035706653	0.171	0.155	1
6.01051E-05	0.035722499	0.368	0.343	1
0.01303636	0.035725241	0.554	0.533	1
8.39825E-05	0.035730478	0.404	0.38	1
0.000535386	0.035818457	0.431	0.411	1
0.001705698	0.036422839	0.37	0.35	1
0.125527484	0.036538	0.221	0.214	1
5.30987E-05	0.036677683	0.17	0.154	1
0.000591055	0.036879754	0.177	0.163	1
0.000658726	0.036944617	0.212	0.197	1
0.000733104	0.03705691	0.263	0.246	1
0.001763737	0.037126764	0.2	0.186	1
7.68608E-05	0.037128165	0.214	0.197	1
0.01663523	0.037257492	0.397	0.379	1
0.000105614	0.037283733	0.181	0.166	1
8.73159E-05	0.037512705	0.23	0.212	1
0.028309964	0.037564722	0.199	0.19	1
0.000109994	0.03756889	0.153	0.139	1
8.61424E-05	0.037893692	0.754	0.75	1
0.000142082	0.038093788	0.244	0.226	1
0.000125879	0.038228992	0.796	0.79	1
0.169877621	0.038327809	0.118	0.114	1
0.000109081	0.038468828	0.218	0.2	1
0.000253929	0.038838936	0.579	0.552	1
0.001393851	0.038865605	0.204	0.191	1
0.000196627	0.039091733	0.293	0.274	1
6.36793E-05	0.039119853	0.237	0.218	1
0.000649245	0.039148855	0.855	0.848	1
0.000134498	0.039257937	0.105	0.094	1
0.000234331	0.039351393	0.11	0.099	1
0.000186243	0.039390114	0.151	0.138	1
0.081182747	0.039575842	0.654	0.635	1
0.003058363	0.039675498	0.766	0.759	1
5.01992E-05	0.03968272	0.165	0.15	1
0.000636152	0.039757042	0.108	0.098	1
0.007451293	0.039855717	0.125	0.117	1
9.58888E-05	0.039894125	0.241	0.223	1
4.93675E-05	0.039990092	0.28	0.261	1
0.004833829	0.040016799	0.414	0.396	1
0.002876256	0.040111043	0.49	0.47	1
0.000992394	0.040265081	0.607	0.585	1
0.003040703	0.040405991	0.26	0.246	1
0.032496562	0.040478577	0.187	0.179	1
0.00678134	0.040577609	0.133	0.123	1
0.005721187	0.040695425	0.237	0.225	1
0.436222205	0.040704171	0.116	0.118	1
0.482227971	0.040898996	0.25	0.248	1
0.000118535	0.041189606	0.733	0.725	1
0.037211182	0.04119503	0.656	0.637	1
9.98418E-05	0.04131114	0.236	0.218	1

4.15766E-05	0.0414813	0.513	0.485	1
0.000399899	0.041672924	0.339	0.32	1
9.79869E-05	0.04177693	0.649	0.629	1
0.000869179	0.041823163	0.255	0.239	1
0.000298647	0.04186617	0.525	0.5	1
5.11998E-05	0.041927067	0.361	0.338	1
0.000659531	0.041967807	0.557	0.531	1
0.001972303	0.041971303	0.289	0.275	1
0.009878196	0.042049769	0.202	0.191	1
0.016119431	0.042305078	0.575	0.562	1
0.000991836	0.043108988	0.126	0.115	1
0.001680921	0.043285844	0.496	0.474	1
5.52253E-05	0.043293295	0.736	0.726	1
0.000247573	0.043301752	0.573	0.552	1
0.003592357	0.04341416	0.619	0.6	1
0.016954817	0.043580317	0.183	0.173	1
0.000252671	0.04381156	0.333	0.312	1
5.59668E-05	0.044180201	0.172	0.156	1
0.000358056	0.044243916	0.415	0.392	1
6.12645E-05	0.044270428	0.3	0.277	1
0.000139756	0.044398869	0.62	0.596	1
0.001619228	0.044411479	0.21	0.198	1
0.000170688	0.044513586	0.253	0.235	1
0.003447464	0.044664881	0.13	0.12	1
0.002158176	0.044869422	0.279	0.265	1
0.262525055	0.044928077	0.731	0.713	1
0.00691821	0.0450085	0.303	0.288	1
0.002090622	0.045435801	0.405	0.388	1
0.054135639	0.045637044	0.248	0.237	1
0.002184454	0.04602551	0.264	0.248	1
0.110000928	0.046075972	0.504	0.492	1
0.005959549	0.046129182	0.455	0.434	1
0.002996715	0.046894533	0.263	0.249	1
0.000157613	0.04690532	0.14	0.127	1
0.000314632	0.046955628	0.24	0.224	1
0.00018898	0.046955683	0.456	0.436	1
0.000235677	0.047030348	0.132	0.12	1
0.00023585	0.047076782	0.618	0.596	1
0.000165439	0.04733738	0.495	0.47	1
0.000260261	0.047462944	0.221	0.205	1
0.000459527	0.047653301	0.355	0.336	1
0.000688895	0.047731884	0.463	0.439	1
0.000737412	0.047895083	0.132	0.121	1
0.000283101	0.04828168	0.205	0.19	1
0.000530289	0.048312346	0.262	0.246	1
0.018146832	0.048378507	0.337	0.322	1
0.185403915	0.048506004	0.63	0.617	1
0.004593576	0.049131133	0.248	0.235	1
0.004272807	0.049231754	0.145	0.135	1
0.118533476	0.049494211	0.16	0.154	1

0.000298719	0.04953432	0.726	0.706	1
0.000367366	0.049663868	0.321	0.302	1
0.000140085	0.049772454	0.14	0.127	1
0.000501347	0.049810621	0.178	0.164	1
0.000275112	0.049925698	0.229	0.214	1
0.000701115	0.05013302	0.533	0.507	1
4.41014E-05	0.05095714	0.814	0.805	1
0.004828424	0.051142547	0.692	0.664	1
5.12705E-05	0.051376151	0.378	0.353	1
4.12149E-05	0.05243644	0.3	0.278	1
9.56684E-05	0.052736921	0.112	0.1	1
0.006399275	0.053326445	0.397	0.379	1
0.000200217	0.053896365	0.305	0.286	1
0.000544884	0.054240142	0.235	0.219	1
0.766071941	0.0542803	0.308	0.304	1
0.000642469	0.054729197	0.481	0.458	1
0.000332326	0.055304522	0.419	0.396	1
0.000222221	0.055525507	0.295	0.276	1
0.14474562	0.056012533	0.125	0.12	1
0.023368687	0.056314212	0.164	0.156	1
0.041576885	0.056314489	0.937	0.929	1
9.34802E-05	0.057186867	0.267	0.247	1
0.000116852	0.057663664	0.357	0.336	1
0.000288846	0.05939533	0.125	0.113	1
0.000317911	0.059632894	0.337	0.32	1
0.629135148	0.060346955	0.756	0.747	1
0.000162919	0.060561027	0.225	0.209	1
0.001522887	0.060589699	0.379	0.362	1
0.00015941	0.062483586	0.813	0.799	1
0.006379708	0.062853102	0.204	0.192	1
0.003599001	0.06356739	0.297	0.285	1
4.39881E-05	0.064605369	0.492	0.467	1
5.47099E-05	0.064687424	0.199	0.183	1
0.002153228	0.065402072	0.783	0.765	1
0.026213054	0.066380149	0.602	0.587	1
0.000145052	0.066813483	0.477	0.456	1
7.30766E-05	0.070781886	0.647	0.633	1
0.032747896	0.071100761	0.505	0.495	1
0.135124314	0.071457006	0.119	0.115	1
0.001357532	0.071990625	0.356	0.34	1
0.000255303	0.072504023	0.237	0.22	1
0.004070096	0.072766379	0.501	0.482	1
8.43942E-05	0.074475339	0.532	0.507	1
0.004196208	0.075345478	0.2	0.189	1
0.001253646	0.078316784	0.103	0.094	1
0.000140935	0.078871758	0.229	0.213	1
0.01800806	0.080986902	0.211	0.202	1
0.001616359	0.081040452	0.23	0.217	1
0.005994537	0.085438451	0.134	0.126	1
0.027371821	0.086601954	0.537	0.532	1

0.038017099	0.088643495	0.133	0.127	1
0.016662972	0.092891368	0.143	0.136	1
0.000155591	0.094548266	0.182	0.169	1
0.000718623	0.100553527	0.133	0.123	1
0.000248787	0.110497524	0.353	0.338	1
5.7638E-05	0.129945195	0.147	0.135	1
7.53847E-05	0.143711799	0.383	0.367	1
0.000155704	0.216840163	0.12	0.109	1

genes
CT010467.1
mt-Rnr2
Uba52
mt-Nd2
Rpl10
Ccl5
Mir6236
mt-Nd4
Rps25
Fau
Fam107b
Rpl23a
Cxcl2
Eif1
Rps12
Gm28438
Rps27a
Rps23
mt-Nd1
Rps17
Klf2
mt-Cytb
A130071D04Rik
Rpl30
Lyz2
Cox8a
Mir142hg
Hsp90ab1
Hspa14
Apoe
Neat1
Rplp2
mt-Co1
Rps14
Rpl31
Rpl23
Arhgef1
Trf
Snrnp70
Gm26917
Rpl27a
Ptges3
Pdia3
B2m
H3f3a
Ahnak
Nfkbia
Rhoh

Rps15
Ccrl2
Ccl6
Bcl2a1b
Fn1
Rps9
Dnaja1
Siglecg
Txn1
Cebpb
Notch2
Tyrobp
Rpl35a
Jund
Laptm5
Rpl34
mt-Rnr1
Tln1
Ripor2
Tra2a
Rps13
S100a6
Rpl26
Gm10076
Hsp90aa1
Foxp1
S100a4
Prpf18
Malat1
Gng5
Gm37474
Actb
Ogt
Grn
Zfp36l2
Cacybp
Arhgap45
Rps6
Hsp90b1
Wfdc21
Gm49980
Klf9
Iglc2
Cox6b1
Calm1
Rbpms
AC147806.2
mt-Nd5
Igkc
Irf2bp2
