# Supplementary material for: Neutrophil-enriched gene signature correlates with teplizumab therapy resistance in different stages of type 1 diabetes
Source: J Clin Invest. 2025 Sep 30;135(23):e176403. doi: 10.1172/JCI176403 (PMC12646666; doi:10.1172/JCI176403)
Supplement: Supplemental table 4 [file jci-135-176403-s292.pdf]

Supplemental Table 4: List of genes correlating with C-peptide AUC in AbATE Month 6

**Symbol**	**Pearson_corr**	**p_value**
CD160	0.509349487	0.000217879
MCOLN2	0.505830606	0.000244854
KLRD1	0.503981207	0.000260214
F2R	0.496328645	0.000333487
GAN	0.494395091	0.000354737
PDGFD	0.486066759	0.000460956
TIGIT	0.485334642	0.000471543
GNG2	0.484174742	0.000488766
EOMES	0.480654579	0.000544563
FCRL6	0.470629795	0.00073625
PPP2R2B	0.470557723	0.000737824
TOX	0.470506769	0.000738939
C8orf76	0.466150571	0.000839989
GZMA	0.461172239	0.000970504
CEP78	0.458087705	0.001060204
CCL4L2	0.456360498	0.001113607
RYR1	0.452539547	0.001240375
FLT3	0.447643943	0.001421544
BIVM	0.446934265	0.001449676
CCL5	0.441947057	0.001661761
KIF21A	0.439364752	0.001782068
SYNM	0.438704165	0.001814061
LYAR	0.434769853	0.002015392
PTGDR	0.434565787	0.002026355
SAMD3	0.428013495	0.002407705
XCL2	0.427553605	0.002436713
AUTS2	0.426961721	0.002474501
GZMK	0.425606643	0.002562972
KLRG1	0.422883779	0.002749239
SLAMF6	0.414600819	0.003391421
PPP2R5C	0.41399495	0.003443198
S1PR5	0.410009486	0.003801456

WEE1	0.407444164	0.004049014
SLC4A4	0.406624893	0.004131001
B3GAT1	0.406138025	0.004180409
PRR5L	0.402236602	0.004595413
SH2D1A	0.402234944	0.004595597
ZNF831	0.401289609	0.004701427
PTGER2	0.400402656	0.004802654
ZNF184	0.39779284	0.005111652
RUNX3	0.396414947	0.005281693
NCALD	0.395429045	0.005406371
RP11-660L16.2	0.387416639	0.006518525
C1orf21	0.385663893	0.006786753
ELOVL6	0.383951871	0.007057955
KIAA1671	0.383517282	0.007128277
CD99	0.382103522	0.007361265
STK39	0.381616186	0.007443094
RRAS2	0.380092197	0.0077041
CTSW	0.378845607	0.007923457
TRGV2	0.376529387	0.008345418
CERKL	0.376175645	0.008411542
MTMR2	0.374782862	0.008676315
DCAF13	0.373521696	0.008922248
TWF1	0.373501367	0.008926261
GPR137B	0.3720985	0.009206985
STOM	0.371887686	0.009249822
AAED1	0.371705464	0.009286988
TBX21	0.370740343	0.009485986
C5orf34	0.370448093	0.009546966
GCLM	0.370344979	0.009568562
ZBTB38	0.370212687	0.009596331
GZMH	0.369606679	0.009724423
ENPP4	0.367270927	0.010232011
HOPX	0.367165679	0.01025541
TRGV10	0.366405219	0.010425848

DENND6A	0.366282917	0.010453485
FRG1CP	0.366045766	0.010507254
CST7	0.366013559	0.010514575
RP11-4O1.2	0.365966803	0.01052521
RP5-1028K7.2	0.365955967	0.010527676
ALG10	0.36542441	0.010649264
LPCAT1	0.364919254	0.01076593
ATP6V1D	0.364700181	0.010816865
GFI1	0.364698872	0.01081717
SACM1L	0.364294878	0.010911646
LDAH	0.364285147	0.01091393
STAMBPL1	0.363730212	0.011044882
PAM	0.362260426	0.011398258
S100PBP	0.362107685	0.011435532
PIP4K2A	0.361744606	0.011524555
RAB6A	0.361542087	0.011574467
CMC1	0.361338016	0.011624951
TMEM68	0.358823735	0.012262596
HIGD1A	0.358691513	0.012296942
ACSL5	0.35849452	0.012348265
PYHIN1	0.358055698	0.012463254
GPR171	0.357868551	0.012512572
JAKMIP1	0.357364892	0.012646129
UBE2K	0.356643685	0.012839497
KRAS	0.356150191	0.012973265
SPAST	0.355337755	0.013196081
GTDC1	0.354886542	0.013321236
TRBV7-9	0.354679334	0.01337905
C1orf216	0.354299197	0.013485669
TRGC2	0.353506159	0.013710437
NAA50	0.353400104	0.013740737
CRIM1	0.353347086	0.013755906
RP11-222K16.2	0.350761339	0.014513255
C9orf64	0.350202645	0.014681481

C12orf75	0.345811001	0.016062637
GLCCI1	0.345205892	0.016261331
TGFBR3	0.344602127	0.016461661
IQGAP2	0.344257248	0.01657703
PTPN22	0.343662397	0.016777632
USP28	0.343431181	0.01685616
TRAV24	0.343229399	0.016924945
GBP3	0.342796118	0.017073452
FRMPD3	0.340114925	0.018017223
RP11-25K19.1	0.338831826	0.018484273
FGFBP2	0.33844834	0.018625834
UCHL5	0.338095178	0.018757012
XPR1	0.338009689	0.018788883
RAB27A	0.337841092	0.018851872
RP11-532F6.3	0.337491266	0.018983139
SOX13	0.337286888	0.019060185
GOLIM4	0.337034361	0.019155748
TRDV1	0.335904764	0.019588188
ARHGEF3	0.335683708	0.019673772
ZCCHC17	0.33482575	0.020008933
MSC	0.334120796	0.020287917
FEM1C	0.333921292	0.020367464
RAP2A	0.333589922	0.020500169
CD8A	0.333257176	0.020634158
VTI1B	0.332136378	0.021090922
JAKMIP2	0.331922878	0.021178889
ZNF791	0.330712797	0.021683332
C2orf69	0.330572893	0.0217423
GTF2E2	0.330471052	0.021785309
PTPN4	0.330096177	0.021944244
SDCCAG8	0.329919369	0.022019543
DLG3	0.329039494	0.022397502
RP11-539L10.2	0.328852214	0.02247865
SYT11	0.328796827	0.022502696

TIPARP	0.328775769	0.022511844
MAPKAPK2	0.328469689	0.022645165
CBLB	0.327713716	0.022977292
RASGEF1A	0.327516719	0.023064509
NRBP1	0.327503737	0.023070266
ZNF432	0.327478477	0.023081472
KLHL12	0.327348537	0.023139188
LANCL2	0.327197951	0.023206227
MED31	0.326537915	0.023501989
TLR3	0.325990441	0.023749707
REEP5	0.32535726	0.024038931
RAB9A	0.325155449	0.024131733
DNAJC1	0.324942018	0.024230204
ZUFSP	0.324280748	0.024537437
CCSER2	0.32365146	0.024832836
CASC4	0.323612549	0.024851198
7-Sep	0.323496587	0.02490599
GPR68	0.323250269	0.02502271
TMEM14A	0.323123913	0.025082763
HERPUD2	0.322879306	0.02519936
EIF4EBP2	0.322786081	0.025243916
C6orf3	0.322475136	0.025393008
TXN	0.322203586	0.025523812
KATNAL1	0.322095251	0.025576154
PPM1D	0.321920258	0.025660891
RAB29	0.321323441	0.025951654
TMED5	0.321028125	0.026096545
CDYL2	0.319822978	0.026694854
SLAMF7	0.319078161	0.027070321
ZNF181	0.318958939	0.027130828
RNF4	0.318512029	0.02735865
ALKBH1	0.318435953	0.02739759
PAQR3	0.318393388	0.027419398
SERPINB9	0.317795932	0.027727022

ANXA1	0.317549479	0.027854754
TERF1	0.317404083	0.02793034
RP11-617F23.1	0.316989819	0.028146638
SLC25A20	0.316705269	0.028296016
CCDC6	0.316386396	0.028464196
RNF19A	0.316165645	0.028581112
ARAP2	0.315750045	0.02880231
NKG7	0.314809692	0.029308066
CISD1	0.31430478	0.02958266
CCDC15	0.314172916	0.029654724
TRBV6-5	0.313145628	0.030221159
NMUR1	0.312011118	0.030857129
AK3	0.311664923	0.031053392
ATP6V0A2	0.311176324	0.031332148
SLF1	0.310813493	0.031540492
BATF	0.310722795	0.031592751
TAF13	0.310542896	0.031696619
UROS	0.310513312	0.031713727
CH507-9B2.9	0.310327726	0.031821224
PHACTR2	0.30994478	0.032043992
FBXO30	0.309881993	0.032080639
CENPBD1P1	0.309462118	0.032326606
RAB22A	0.308960405	0.03262256
PREPL	0.308795871	0.032720103
TPST1	0.308397353	0.032957365
RBMXL1	0.308260666	0.03303907
SAP30	0.30778746	0.033323227
PHLDB2	0.307318093	0.033607073
RP11-251G23.5	0.307258148	0.033643468
KLHL8	0.306509563	0.034100711
SLU7	0.306476652	0.034120931
SDHC	0.306462656	0.034129533
MEX3C	0.306273809	0.034245771
G2E3	0.305811107	0.034531955

TBK1	0.30554233	0.0346991
PPP2R5A	0.305079321	0.034988599
NCR1	0.304201456	0.035542963
KIN	0.303990058	0.035677536
APOBEC3G	0.303672723	0.035880335
ZBTB33	0.30333464	0.036097439
ZMPSTE24	0.303160998	0.036209365
ARMC10	0.303091443	0.036254278
PCBD1	0.303046658	0.036283222
PRF1	0.302118134	0.036887606
ST8SIA1	0.301926475	0.037013385
FASLG	0.301916901	0.037019677
BNIP2	0.301490542	0.037300787
HAUS2	0.301488403	0.037302201
TAX1BP3	0.301481021	0.037307084
HARBI1	0.301177616	0.037508223
IKZF3	0.300640132	0.037866732
ZNF630	0.300463471	0.037985181
UBLCP1	0.300006924	0.038292701
UBR7	0.299163525	0.038866176
KDELC2	0.29816271	0.039555821
LIPA	0.297950049	0.039703648
ITCH	0.297263709	0.040183839
BBS7	0.296976658	0.040386079
STS	0.296651718	0.04061602
ARL4C	0.296353157	0.040828238
GOLPH3	0.296203867	0.040934694
AMZ2	0.296173655	0.040956265
YARS	0.296003209	0.041078138
UBFD1	0.29599629	0.041083091
TMEM33	0.295137663	0.041701614
CD2	0.293834307	0.042655052
TMEM81	0.293824433	0.042662343
GID4	0.29348164	0.042916078

VPS33A	0.293213552	0.043115375
YARS2	0.292717465	0.043486161
GLB1L3	0.292602869	0.043572182
ACTA2	0.292091832	0.043957481
NAPEPLD	0.291938187	0.044073865
AAGAB	0.291499379	0.044407641
CENPO	0.291018503	0.044775781
ACTR1A	0.290664553	0.045048339
CKS2	0.290613422	0.045087824
CNNM4	0.290505838	0.045170995
TRIM59	0.290311883	0.045321254
C16orf87	0.29018965	0.045416159
PTBP3	0.289950472	0.04560233
CLIC3	0.28994197	0.04560896
TROVE2	0.289936826	0.045612971
VPS4B	0.289934254	0.045614977
CHST12	0.289689746	0.045805982
COA1	0.289497474	0.045956639
FASTKD1	0.289446971	0.045996278
FUT11	0.289250941	0.046150403
NFATC2	0.288997738	0.046350101
C9orf172	0.288600494	0.04666482
STK26	0.288193574	0.046989004
ELMOD2	0.288094486	0.047068222
TPRG1	0.287404073	0.047623208
RBM7	0.286987283	0.04796081
SEMA3C	0.286932297	0.048005493
SLC25A53	0.286007175	0.048762367
RNF38	0.285966878	0.048795554
EXOC6B	0.285672728	0.049038361
CKAP2	0.285592632	0.049104646
ATF2	0.285541863	0.049146699
SREK1IP1	0.28528133	0.049362962
C21orf91	0.285262846	0.049378334

MIS18BP1	0.284976161	0.049617257
PALLD	0.284959578	0.049631105
DCUN1D1	0.284957793	0.049632596
KIF5B	0.28465349	0.04988729
DIAPH2	0.284458637	0.050050933
SLC25A32	0.28434895	0.050143242
TXNDC16	0.28432924	0.050159844
TMEM87B	0.284238369	0.050236443
FAM21C	0.284214429	0.05025664
MTMR9	0.283654783	0.050730637
PWWP2A	0.283588997	0.050786592
TBC1D23	0.283493894	0.050867571
PSMC6	0.283493339	0.050868045
CCL4	0.2834229	0.050928091
AHR	0.283168071	0.0511458
PRSS23	0.283096816	0.05120681
ZDHHC9	0.282969253	0.05131618
BLOC1S3	0.282908406	0.051368416
VPS54	0.282605237	0.051629319
VCPIP1	0.282189677	0.051988686
DDX59	0.281960866	0.052187419
FYN	0.281586318	0.052514057
GPATCH2	0.281362158	0.052710332
KIAA1551	0.281325303	0.05274266
SLC30A1	0.281039982	0.052993469
CDK13	0.281037966	0.052995245
ARPC5L	0.280641095	0.053345722
TPST2	0.280309397	0.053640079
PLEKHF1	0.280120218	0.053808547
PIWIL4	0.27971946	0.054166842
MAGOH	0.279600516	0.054273554
ID2	0.279579667	0.054292276
YWHAQ	0.279544921	0.054323489
PMS1	0.279307862	0.05453683

AFTPH	0.279190747	0.054642477
ADGRG1	0.279084835	0.05473816
HSPA1L	0.278183684	0.055557755
MATK	0.277931121	0.055789225
AKAP5	0.277891991	0.055825157
LCORL	0.277787121	0.055921546
DERL1	0.27705942	0.056594099
DNAJB9	0.277031331	0.056620189
DPY19L1	0.277002861	0.056646644
LINC00662	0.276223198	0.05737497
SYNE1	0.275807866	0.057766017
AC092580-4	0.275641102	0.057923632
SMC6	0.275563765	0.057996843
PHPT1	0.275291648	0.058255034
TRAV38-2DV8	0.275143473	0.058396013
GFOD1	0.275034045	0.058500303
CRNKL1	0.275003064	0.058529858
FAAP24	0.27477754	0.058745352
ITPRIPL1	0.274407994	0.059099839
ETNK1	0.274253736	0.059248318
NPC1	0.273874675	0.05961445
RP11-278C7.1	0.272842115	0.060621002
FAM210A	0.272736777	0.060724448
GPD1L	0.272644246	0.060815433
DNAJA2	0.272562009	0.060896389
IPO11	0.272537897	0.060920141
PDCD1	0.272439312	0.061017333
SP4	0.271990785	0.061461092
CXCR3	0.271656632	0.061793369
RABIF	0.271613756	0.061836108
SLBP	0.271174696	0.06227513
S1PR2	0.271030975	0.062419379
LARP7	0.271029426	0.062420935
GNAO1	0.270574272	0.062879533

LINC01291	0.270328552	0.06312823
MBNL2	0.270010696	0.063451101
MSANTD4	0.269927648	0.063535676
YPEL1	0.26949667	0.063976028
CCDC50	0.269142949	0.064339261
ZGRF1	0.269116749	0.064366231
PRIMPOL	0.268997617	0.064488977
GS1-358P8.4	0.26863545	0.064863279
ARPP19	0.268521183	0.064981733
SMS	0.268441251	0.065064698
COPS8	0.268341291	0.065168568
MYBL1	0.268190936	0.065325054
RPS6KB1	0.267840083	0.065691378
PRKCH	0.267404497	0.066148448
ZNF720	0.267167364	0.066398341
KCTD9	0.2669943	0.06658119
SUGT1	0.266543756	0.067059091
C3orf58	0.26642804	0.067182272
SPIN4	0.266300425	0.06731833
RARRES3	0.266214107	0.067410483
SH3KBP1	0.266021154	0.067616841
PDCD4	0.265993485	0.067646474
CEP83	0.265895373	0.06775163
TMEM9B	0.265734263	0.067924592
DYNLL1	0.265581532	0.068088882
LGR6	0.265357396	0.068330552
SNW1	0.265348183	0.068340501
MAN1A2	0.264952031	0.068769362
SSPN	0.264648807	0.069099064
CNPY2	0.264496874	0.069264735
FAM177A1	0.264402166	0.069368167
C1orf27	0.264375606	0.069397196
TCTA	0.264224115	0.06956295
UBASH3B	0.264199916	0.069589458

DHFRL1	0.264184475	0.069606375
MVB12B	0.263776811	0.070054205
TTC19	0.262921445	0.07100128
SNAP29	0.262692376	0.071256624
SUPT3H	0.262436009	0.07154326
PPP1CB	0.262140277	0.071875042
GSKIP	0.261991179	0.072042777
SS18	0.26170706	0.072363267
CD84	0.261672001	0.072402893
ACTR2	0.261340665	0.072778233
LBR	0.261066345	0.073090147
ARL6IP5	0.261035774	0.073124974
LPIN2	0.260966085	0.073204412
RP11-342K6.1	0.260923694	0.073252766
C3orf33	0.260555198	0.073674167
USP38	0.260486836	0.073752554
AMMECR1	0.260306753	0.073959361
TULP4	0.260266124	0.074006083
RP11-421F16.3	0.259930746	0.074392644
GNB1	0.259869013	0.074463972
FAF1	0.259688554	0.074672789
ATF1	0.259501405	0.074889836
TAOK3	0.259439726	0.074961477
AC074117-10	0.259186066	0.075256677
HIRA	0.258969767	0.075509121
PDP2	0.258786194	0.075723896
MED28	0.258522699	0.076033017
STK38	0.258014597	0.076631913
PSMA3	0.257841968	0.076836232
TRG-AS1	0.257751737	0.076943199
CCDC117	0.257524784	0.077212764
MMGT1	0.257255859	0.077533145
COG6	0.257225243	0.077569685
HSPB11	0.257182632	0.077620564

XXbac-BPG283O16.	0.256881113	0.077981338
FEM1B	0.256638962	0.078272033
SMIM20	0.256592763	0.07832759
MAF	0.256485695	0.078456465
MFSD6	0.256427961	0.078526027
SLC31A2	0.256372286	0.078593154
RP11-644F5.11	0.256210619	0.078788332
SCP2	0.256105685	0.078915221
ARL6IP1	0.255931874	0.079125751
RNF115	0.255925921	0.07913297
IPO8	0.255824092	0.079256524
RSBN1L	0.255621013	0.079503385
NSUN4	0.255257716	0.079946513
HECA	0.255222479	0.079989597
NMRAL1	0.255172516	0.080050716
RTCA	0.255106572	0.08013144
ENDOD1	0.254948076	0.080325724
ZNF319	0.25484276	0.080455023
CPT1A	0.254429317	0.080964202
UGCG	0.254397414	0.081003597
NAA30	0.254372983	0.081033776
RBBP8	0.254289318	0.081137191
GDPGP1	0.254250854	0.081184769
MIER3	0.25419324	0.081256077
ZPR1	0.254172464	0.081281803
HAR1A	0.254127411	0.081337612
CHORDC1	0.254117933	0.081349356
STARD4	0.254090163	0.081383774
HOXB4	0.25366882	0.081907399
RBM4	0.253445934	0.082185457
NKIRAS1	0.25342289	0.082214248
ARHGAP18	0.25293293	0.082828261
CPOX	0.252651073	0.083183106
PSPC1	0.252505215	0.083367201

TOR1A	0.25244674	0.083441095
CMTR2	0.252380384	0.08352501
TBPL1	0.252224716	0.08372213
PDZD11	0.252110862	0.083866533
COMMD3	0.251476222	0.08467503
RP11-686D22.10	0.251443426	0.084716976
ERI1	0.251196064	0.08503387
TBC1D7	0.251193403	0.085037285
AKIP1	0.25117006	0.085067237
PFDN4	0.251169606	0.08506782
DENND1B	0.251124122	0.085126209
NCOA7	0.250619225	0.085776458
RAD51B	0.250022856	0.086549504
DUSP8	0.249911938	0.086693879
LINC01003	0.249685545	0.086989147
PPP1R16B	0.249501991	0.087229118
DCLRE1A	0.249116056	0.087735358
TTC27	0.24890342	0.088015256
LACE1	0.24878745	0.088168202
CPNE3	0.24849534	0.08855437
BMPR1A	0.248439322	0.088628575
ASF1A	0.24839925	0.088681687
ATP6V1C1	0.248140922	0.089024675
CYP4F22	0.247821948	0.08944961
HNRNPLL	0.247625864	0.089711613
ANKRD13C	0.247552821	0.089809365
ARF6	0.247390376	0.090027057
RMDN3	0.247270506	0.090187959
NUP37	0.24718743	0.090299602
PALB2	0.247116465	0.090395055
AGPS	0.247046686	0.09048899
AP4E1	0.24700301	0.090547824
RNASEH2A	0.24691227	0.090670151
LRRC8B	0.246754795	0.090882749

PWP1	0.246725025	0.090922982
CCT8	0.246687611	0.090973567
SLAIN2	0.246634622	0.091045247
KLHL24	0.2466246	0.091058809
PPP1R14B	0.246558054	0.091148901
RPL23AP82	0.246420186	0.091335771
WBP1L	0.246303696	0.091493896
SLC24A1	0.246199478	0.091635543
FAM53B	0.246085184	0.091791082
TMEM63C	0.24591866	0.092018064
TRIM65	0.245884812	0.092064254
DHX29	0.245843232	0.09212102
CBX1	0.24581444	0.092160344
TUBGCP3	0.245795341	0.092186437
PLCD4	0.245764922	0.092228005
ATG10	0.245704295	0.092310898
TRIM52	0.245634443	0.092406476
ATXN1	0.245411401	0.092712178
MAT2B	0.245346649	0.092801073
ASCC1	0.245326236	0.092829111
KIAA0391	0.245008214	0.093266776
CRTAM	0.244883834	0.093438384
SMYD2	0.244776069	0.093587265
IGLV3-25	0.244621616	0.093800969
MTF2	0.244527469	0.093931418
SGCB	0.244091035	0.094537975
LINC00657	0.243980514	0.094692058
TRAPPC6B	0.243943979	0.094743036
CDC27	0.243939753	0.094748934
FANCL	0.243674846	0.095119218
BRI3BP	0.243552249	0.095290962
ZBTB6	0.243250742	0.095714358
METTL18	0.242998745	0.096069347
EXOC5	0.242727407	0.096452717

NCR3	0.242701723	0.096489067
ZNF571	0.242469849	0.096817711
TM9SF3	0.242303589	0.097053891
ZNF350	0.242098969	0.097345175
C19orf12	0.24207135	0.097384543
CLIP4	0.241898835	0.097630726
RNASE6	0.241784472	0.097794189
EFR3A	0.241762428	0.09782572
FAM109B	0.241659036	0.097973721
PRKAR2A	0.241374397	0.098382062
IL32	0.241350322	0.09841666
ATG5	0.24119037	0.098646764
SACS	0.241188473	0.098649495
TFG	0.241172822	0.098672034
ENPP5	0.24104158	0.098861184
CHP1	0.2409876	0.098939062
VPS26A	0.240909749	0.099051463
SNAPC5	0.240828953	0.099168221
GYG1	0.240492978	0.099654875
FBXO45	0.240431184	0.099744583
PMAIP1	0.24039198	0.099801529
ZNF197	0.240094409	0.100234582
CAPZA1	0.240054446	0.10029285
RNF34	0.23981912	0.100636497
OXSR1	0.239738564	0.100754341
PDCD10	0.239439913	0.101192163
PDS5B	0.239343275	0.101334147
YES1	0.23900172	0.1018372
PRR7	0.238982988	0.101864845
PDIA3	0.238736563	0.102229056
RCN1	0.238669659	0.102328113
PM20D2	0.238609423	0.10241736
CCDC65	0.238460421	0.102638379
MOB1A	0.237976829	0.103358239

ACBD5	0.237860283	0.103532304
SFT2D2	0.237840762	0.103561481
USMG5	0.237758898	0.103683908
MSL2	0.237563247	0.103976955
DIEXF	0.237146152	0.104603806
CADM1	0.236884775	0.104998105
RC3H2	0.236710524	0.105261603
MTPN	0.236563046	0.105485013
PANK3	0.236511405	0.105563328
MNAT1	0.236394416	0.105740908
PI4K2B	0.236314363	0.105862556
ZNF468	0.236258985	0.105946772
STK17A	0.235586301	0.10697384
MIF4GD	0.23554592	0.107035737
SLC35E3	0.23549632	0.107111803
TNKS2	0.235487801	0.107124872
ANXA6	0.23546691	0.107156925
FZD1	0.235450394	0.107182271
USPL1	0.235435648	0.107204904
CDKN2B	0.235424703	0.107221705
SLC41A2	0.235173464	0.107607937
WAC	0.235122141	0.107686967
TGFBR1	0.235108575	0.107707864
BTN3A2	0.234996582	0.107880498
PREP	0.234707167	0.108327602
GSR	0.234483153	0.108674643
PPP3CB	0.234382458	0.108830915
ABCD3	0.234293612	0.108968942
AZIN1	0.234009441	0.109411315
PRDM4	0.233860312	0.109644015
PTPMT1	0.233808472	0.109724993
CHUK	0.233593476	0.110061327
HLA-G	0.233259321	0.11058563
FGFR1	0.233195544	0.110685916

GEMIN6	0.233189288	0.110695757
TM9SF2	0.233139415	0.110774232
PPP2R5E	0.233128583	0.110791283
CA5B	0.233075247	0.110875265
USP24	0.232946615	0.111078006
PLEK	0.232586709	0.111646769
LINC00869	0.232565062	0.111681049
PPM1L	0.232325748	0.112060557
DEK	0.232301965	0.112098326
SLC25A38	0.232293598	0.112111616
RNF6	0.232229953	0.112212745
HSPA1B	0.232018941	0.112548533
NIP7	0.231898139	0.112741112
RBM8A	0.231850233	0.112817553
ADPRH	0.231475998	0.11341605
DSTN	0.231438334	0.113476418
PSMC5	0.230947585	0.114265233
PCMT1	0.230921391	0.114307453
TRMT10A	0.230609219	0.114811538
ALG6	0.230517731	0.11495959
SNRPE	0.230479656	0.115021248
RAB1A	0.230456697	0.115058439
STAT4	0.229503482	0.116610653
LINC01560	0.229187165	0.117129242
SNF8	0.228915289	0.117576369
DERL2	0.228866771	0.117656298
EPS15	0.228858782	0.117669463
AQR	0.228698436	0.117933933
ATP2B4	0.228687642	0.117951753
FOXN2	0.22867841	0.117966995
NRG1	0.228508031	0.118248566
ZNF227	0.228371701	0.118474236
STX6	0.228358564	0.118495998
TAF1A	0.227994008	0.11910114

DPP8	0.227985795	0.1191148
ZNF808	0.227890174	0.119273927
PTPRM	0.227877613	0.119294842
TMEM5	0.227777174	0.119462181
ZNF615	0.227773533	0.119468252
DESI2	0.227707229	0.119578821
AIFM2	0.227575979	0.119797924
RPE	0.22757554	0.119798657
GNPDA2	0.227465951	0.119981834
HMGB1	0.227405471	0.120083017
GXYLT1	0.227086482	0.120617752
TCTN3	0.226830029	0.12104896
PSMC4	0.226801569	0.121096885
CLDND2	0.226715423	0.121242037
PCCA	0.226620416	0.121402275
ACAT1	0.226242688	0.122040926
SELK	0.226109549	0.122266638
IBA57	0.226053657	0.122361484
ZC3H15	0.226003619	0.122446446
RAD51D	0.225952785	0.122532804
ZSWIM3	0.225910664	0.122604396
APOBEC3H	0.225887155	0.122644367
TMEM64	0.225662461	0.123026898
KCTD20	0.225610132	0.123116113
HMGB2	0.225477085	0.123343169
ZNF683	0.225392802	0.123487168
LAG3	0.225280399	0.123679407
DTHD1	0.225147176	0.123907547
RSPRY1	0.225086292	0.124011913
VPS25	0.22508272	0.124018038
RMI2	0.224855585	0.124407994
HCG18	0.224841972	0.124431395
ZBTB11	0.224679044	0.124711724
ZNF678	0.224421856	0.125155205

RNF125	0.224380838	0.125226044
ARID5B	0.224340267	0.12529614
DCTN4	0.224311438	0.125345968
PDHB	0.224261991	0.125431465
AC013461-1	0.22426167	0.125432021
CCDC77	0.224260153	0.125434645
ACTR10	0.224088094	0.125732497
ACOX3	0.224072965	0.125758711
ZNF180	0.223905642	0.126048921
PSMA4	0.223854286	0.126138095
SERINC3	0.223803778	0.126225844
PPP2R1B	0.223642372	0.126506565
RIPK1	0.223614143	0.12655571
NOCT	0.223513355	0.126731292
VANGL1	0.223302946	0.127098436
ERLIN1	0.223229999	0.127225907
LRRFIP1	0.223092949	0.127465657
SH2D2A	0.222899252	0.127805083
METTL12	0.222867236	0.12786125
RCSD1	0.222853281	0.127885739
DLD	0.222734396	0.128094503
C3orf38	0.222654711	0.128234573
TIGD2	0.222636838	0.128266007
EOGT	0.222224635	0.128992554
CASP3	0.222149096	0.129126033
RP11-345J18.2	0.222047294	0.129306084
CNIH1	0.221990582	0.129406467
RAP1B	0.221986969	0.129412865
SENCR	0.221976516	0.129431375
ELP2	0.221938162	0.129499308
AEBP2	0.221783706	0.129773151
SEC22B	0.221742905	0.129845562
CNOT6L	0.221623507	0.130057634
LINC00426	0.221363935	0.13051958

TSFM	0.221317857	0.13060171
IL17RB	0.221279226	0.130670598
CPNE8	0.22119382	0.130822992
MICU2	0.221143892	0.130912141
MRPL36	0.220737478	0.131639518
LSM12	0.220653144	0.131790834
KLF6	0.220617731	0.131854411
PIN4	0.22054381	0.131987197
FAM63B	0.220442865	0.132168691
SLMAP	0.220295991	0.132433094
ZNF451	0.220260443	0.132497148
GPRIN3	0.220219662	0.132570659
KBTBD8	0.220197718	0.132610228
SFMBT2	0.219976359	0.133009867
ZNF283	0.219643354	0.13361277
ELK4	0.219638963	0.133620734
PRDM1	0.219633333	0.133630944
ZNF22	0.219599222	0.133692824
XRCC6BP1	0.21945708	0.133950908
HDHD2	0.219434857	0.133991292
FEZ1	0.219322459	0.134195679
MAP4K5	0.219275355	0.134281403
ATAD1	0.219013434	0.134758821
C12orf29	0.218834751	0.135085244
ADGRG5	0.218829253	0.135095298
SKAP2	0.218805193	0.135139298
ACSL6	0.218514229	0.135672262
TTPAL	0.218498557	0.135701012
SEC22C	0.21843655	0.135814812
CCR5	0.218375208	0.13592746
EIF5	0.218337354	0.135997011
MATR3	0.218193029	0.136262428
DBI	0.218168619	0.136307357
CCDC25	0.217994455	0.136628243

ABHD17C	0.217988318	0.136639559
GSK3B	0.21789013	0.136820725
GZMB	0.21782232	0.136945943
TMEM237	0.217807215	0.136973848
IL2RB	0.217751467	0.137076872
TRIAP1	0.217708029	0.137157188
AP001258-4	0.217702103	0.137168147
RAD21	0.217379392	0.137765964
ZNF33A	0.217198429	0.138102043
DBR1	0.216977887	0.138512453
TIAM2	0.216794388	0.138854622
LNX2	0.216696959	0.139036551
HERPUD1	0.216655195	0.139114593
TMEM67	0.216641697	0.139139822
DPH3	0.216422315	0.139550352
YIPF4	0.216312591	0.139756017
YWHAZ	0.21622873	0.139913357
SLC30A6	0.216093294	0.14016774
RNASE2	0.216039343	0.140269168
C15orf61	0.216015617	0.140313791
APOBEC3C	0.215999612	0.140343899
SLF2	0.215980955	0.140379002
IL15RA	0.215896277	0.140538401
PUM2	0.215868158	0.140591362
AVEN	0.215837431	0.140649252
COQ2	0.215691956	0.140923572
ZNF383	0.215575109	0.141144198
GLTP	0.2154526	0.141375787
ARL3	0.215316532	0.141633341
SLC9A3R1	0.215310329	0.14164509
KIAA1841	0.215241125	0.141776224
LANCL1	0.215172691	0.141905987
ANAPC10	0.215011514	0.142211955
NCK1	0.214960393	0.142309101

DBT	0.214923147	0.142379913
SGK3	0.214795048	0.142623649
ANAPC7	0.214634907	0.142928789
FAM172A	0.214370983	0.143432738
SENP2	0.214294072	0.143579844
GZMM	0.214233108	0.143696527
NUDT4	0.214085968	0.143978439
LRRCC1	0.214062442	0.144023551
ABCB10	0.214057311	0.144033392
TMEM181	0.214010447	0.144123292
DNAJB6	0.213862749	0.144406896
GPATCH1	0.213684375	0.144749953
ZNF322	0.213607272	0.144898428
GLTSCR1L	0.213591523	0.14492877
HEXB	0.213573932	0.144962665
CBFB	0.213504403	0.145096695
CAB39	0.213378667	0.145339309
CAPN2	0.213290102	0.145510381
STRN	0.213053363	0.145968393
MTBP	0.213046768	0.145981169
RP11-283I3.6	0.212730098	0.146595529
DDX46	0.21266723	0.146717723
RAB8B	0.212511009	0.147021692
CREB3L4	0.212390462	0.147256568
AKIRIN1	0.212261569	0.14750801
MPHOSPH9	0.212194255	0.147639451
HERC4	0.212176142	0.147674835
POLR2B	0.212151599	0.147722788
CCDC28B	0.21197506	0.148068063
GOSR2	0.211931027	0.148154276
PPP2CA	0.211739194	0.1485303
TGIF1	0.211722143	0.148563757
LLPH	0.211686809	0.148633106
MED27	0.211665484	0.148674972

RRAGC	0.211406452	0.149184199
NUDT15	0.21136882	0.149258286
PSMA1	0.211153063	0.14968358
TBC1D31	0.210997784	0.149990212
STRIP1	0.210910554	0.150162669
EMC2	0.210771345	0.150438195
CD81	0.210492744	0.150990729
ZNF559	0.210383448	0.151207897
PFDN6	0.210323682	0.151326747
WDR25	0.210323368	0.151327372
PSMD4	0.210280734	0.151412196
SLC10A7	0.210076737	0.151818551
NHLRC2	0.209987945	0.151995673
SKAP1	0.209920937	0.152129441
RPP30	0.209725112	0.152520861
NEFH	0.209689043	0.152593038
TPK1	0.209658186	0.152654805
GPR65	0.209597792	0.152775749
SPATA13	0.209536061	0.152899444
ZNF823	0.209272389	0.153428614
DCP2	0.209110515	0.153754148
NNT	0.208950388	0.154076668
MCTS1	0.20889705	0.15418421
MDM2	0.208843494	0.154292246
TMEM2	0.208757136	0.15446657
CSTF3	0.208558756	0.154867572
PPP1CA	0.208311311	0.155368822
TRDC	0.20822835	0.155537142
MYO6	0.207968374	0.156065477
FAM122A	0.207706441	0.156599121
MPST	0.207706323	0.156599362
CLSTN3	0.207641917	0.156730782
ISCA2	0.207518516	0.156982808
HIC1	0.207505316	0.157009785

HNRNPUL2	0.207439896	0.157143531
PRPF18	0.20738734	0.157251039
BCAS3	0.207370843	0.157284797
TMEM161B	0.20731568	0.157397714
SETDB2	0.207308888	0.15741162
SMIM13	0.207200433	0.157633811
PRPF40A	0.207051122	0.157940078
SEL1L	0.207030798	0.1579818
EID1	0.206849665	0.158353998
EMB	0.206550612	0.158969905
FAM114A2	0.206458695	0.159159562
TMED2	0.20627423	0.159540678
GNA13	0.206209976	0.159673587
LHFPL2	0.206192418	0.159709921
PHEX	0.205977412	0.160155322
VAMP7	0.205966689	0.16017756
DOCK11	0.205920918	0.160272505
PNP	0.205813555	0.160495373
GEMIN7	0.205665373	0.160803349
SMAP1	0.205468159	0.161213901
ACSL3	0.205394059	0.161368357
ZNF367	0.205329426	0.161503168
TAF7	0.205239511	0.161690847
ABHD15	0.205087605	0.162008284
FANCE	0.204996454	0.162198981
CMKLR1	0.20485974	0.162485304
R3HCC1	0.204827033	0.162553858
PURA	0.204785798	0.162640316
TM7SF2	0.204745913	0.162723977
TMEM99	0.204726598	0.162764501
RNF14	0.204565146	0.163103533
ZNF614	0.204545342	0.163145156
ZNF555	0.204475972	0.163291009
ASB2	0.204467098	0.163309675

VIMP	0.204444395	0.163357433
BOLA3	0.204340008	0.163577158
FBXO33	0.2042869	0.163689027
CISD3	0.204274403	0.163715362
PAIP1	0.204132796	0.164013958
FICD	0.204075495	0.164134896
LSM3	0.204013791	0.164265202
IFT46	0.20386084	0.164588525
C6orf62	0.203852046	0.164607129
THRAP3	0.203845715	0.164620522
MAN2A1	0.20372121	0.164884093
C2orf76	0.203603096	0.165134418
FAM98B	0.203559939	0.16522595
TCEB1	0.203443466	0.165473168
KPNA4	0.203377662	0.165612957
BMI1	0.203350465	0.165670758
ABCC6	0.203062791	0.166283039
C4orf3	0.203043036	0.166325144
RAP2B	0.202952343	0.16651855
PDZD4	0.202923818	0.166579414
GTF2E1	0.202885404	0.166661404
ZSCAN9	0.202523259	0.167435801
PSEN2	0.202492764	0.16750113
FXR1	0.202469044	0.167551957
HSP90AA1	0.202450989	0.167590652
MXRA7	0.202401104	0.167697602
FNTA	0.202250185	0.168021459
TSR2	0.202164442	0.168205657
EBP	0.202151066	0.168234405
SRPK2	0.202140191	0.168257781
KATNA1	0.202131119	0.168277282
S100A10	0.202056414	0.168437936
ERCC1	0.201893512	0.168788647
SUZ12	0.201868063	0.168843483

SLC30A7	0.201812156	0.168963996
EIF4G3	0.201767146	0.169061062
CRKL	0.201763211	0.169069552
TGDS	0.201705938	0.169193127
FAHD2A	0.201677546	0.169254414
NRAS	0.201292119	0.170087967
FBXO8	0.201207417	0.170271548
AGGF1	0.201194726	0.170299066
SETD8	0.201039135	0.170636702
ALDH3A2	0.20095548	0.170818438
FDPS	0.200904016	0.170930309
PARP8	0.200867428	0.171009875
ISG20L2	0.200812031	0.171130397
ARMC1	0.200554408	0.171691684
FBXL8	0.200536334	0.171731113
TOR1AIP2	0.200449175	0.171921341
RBAK	0.200412215	0.172002052
BTG3	0.20028026	0.172290436
PLEKHB2	0.20024375	0.172370291
LINC00865	0.200239356	0.172379902
TMEM30A	0.200209684	0.172444823
C14orf142	0.20010554	0.172672822
KLRF1	0.200076166	0.172737168
BZW1	0.19999223	0.172921135
GALM	0.199972607	0.172964164
NUDCD2	0.199928164	0.173061646
NFYA	0.1999072	0.173107644
TMEM184C	0.199906047	0.173110173
DIABLO	0.199804541	0.173333013
RLF	0.199602205	0.173777829
DUSP11	0.199454741	0.17410253
NADK2	0.199440365	0.17413421
SYAP1	0.199305655	0.174431252
CALM1	0.199298635	0.174446742

GDAP2	0.199186369	0.174694587
RAP1GDS1	0.199110232	0.174862818
ACYP1	0.199105811	0.174872591
PTPRA	0.199082361	0.17492443
TSNAX	0.199068477	0.174955128
NEK7	0.198922206	0.175278777
FDXACB1	0.198861791	0.17541258
FYTTD1	0.1987125	0.175743538
MKRN2	0.198596037	0.176002034
PTP4A2	0.198510609	0.176191819
AD000864-6	0.198356372	0.176534843
TDP2	0.198216968	0.176845292
TRIM69	0.198104505	0.177096031
TTBK2	0.198037087	0.177246463
ZNF721	0.197981713	0.177370091
FBXW2	0.19794748	0.177446548
WDFY1	0.197851044	0.177662065
MED4	0.197658433	0.178093079
MB21D1	0.197518685	0.178406271
GTF2F2	0.197428253	0.178609149
SOWAHC	0.197381909	0.178713184
MIR4435-2HG	0.197089171	0.17937134
NSF	0.197007249	0.179555833
GIMAP6	0.196943062	0.179700484
POLR1D	0.196900002	0.179797569
PPHLN1	0.196861476	0.179884463
GNGT2	0.196788442	0.180049274
SCN3A	0.196773126	0.18008385
TRAM1	0.196711422	0.180223194
CA4	0.196709428	0.1802277
METTL14	0.196660174	0.180338988
CLCN3	0.196647595	0.180367416
CTC-523E23.1	0.196567757	0.180547933
PSMD12	0.196558297	0.180569332

ARL8B	0.196540009	0.180610702
PPP1R12A	0.196517743	0.180661083
PACSIN1	0.196447896	0.180819187
MGST3	0.196432725	0.180853539
ZBTB44	0.196387407	0.180956187
MON1B	0.196337237	0.181069875
GPR180	0.19629615	0.181163017
UBE2A	0.196112903	0.181578849
PPP2R3A	0.196074497	0.181666088
ZNF77	0.195773897	0.18234994
YAF2	0.19572508	0.18246117
FBXO4	0.195596795	0.182753703
SMIM4	0.195554485	0.182850256
ERAP1	0.195542095	0.182878538
PSMD13	0.195510802	0.182949983
NF1	0.195509622	0.182952678
BCAS2	0.195473987	0.18303406
KLF9	0.195341405	0.183337075
CBLL1	0.195318008	0.183390587
ASCC3	0.195235655	0.183579024
PRKCQ	0.195084738	0.183924708
SMC5	0.19497887	0.184167483
HSPA4	0.194954316	0.184223824
TRGC1	0.194864918	0.184429052
TMX4	0.194705949	0.184794399
ANGEL2	0.194649677	0.18492385
TRAPPC2	0.194503368	0.185260726
MON1A	0.194432337	0.185424433
TXNDC9	0.19429188	0.185748454
KIAA0368	0.194269542	0.185800023
IWS1	0.194262633	0.185815975
MSH3	0.194236524	0.185876265
ARID4B	0.194234873	0.185880079
SS18L2	0.194082435	0.186232374

POLR3GL	0.194025637	0.18636376
KIR3DL1	0.193939267	0.18656368
TTC17	0.193862258	0.186742061
LEPR	0.193814873	0.186851884
NGDN	0.193780915	0.186930613
HCST	0.193680949	0.187162521
COMMD4	0.193608496	0.187330728
CD164	0.193471903	0.18764814
TFR2	0.193410893	0.187790039
MEOX1	0.193403816	0.187806503
NOL7	0.19339154	0.187835066
PPP2CB	0.193350523	0.187930523
MIR9-3HG	0.193333341	0.187970519
RSPH3	0.193314105	0.188015304
DIMT1	0.193289562	0.188072457
ANKIB1	0.193224321	0.188224442
ATL3	0.193221276	0.18823154
TMX1	0.193194062	0.188294964
PTGER4	0.193133292	0.188436651
RBM27	0.19306115	0.188604949
THAP2	0.193043629	0.18864584
PDP1	0.192999552	0.188748736
TRIB3	0.192962573	0.188835092
BLM	0.192919785	0.188935049
TSN	0.192871749	0.18904731
RGS9	0.192681061	0.189493426
SIKE1	0.192662857	0.189536053
ZNF599	0.192571248	0.189750672
PARK7	0.192542456	0.189818161
SNX4	0.192541312	0.189820843
FAM208A	0.192532452	0.189841614
E2F3	0.192469017	0.189990382
KAZN	0.192444763	0.190047284
TFAM	0.192402061	0.190147497

RP11-73E17.2	0.192363709	0.190237531
DDX1	0.192292273	0.190405317
TIFAB	0.192252174	0.190499546
SET	0.192200114	0.190621929
SESN2	0.192190845	0.190643725
ABO	0.192148921	0.190742331
KIFAP3	0.191970155	0.191163198
SDHAF3	0.191962039	0.191182321
ATP2C1	0.191883864	0.191366588
ZNF487	0.191870392	0.191398356
CDC73	0.191853858	0.191437349
NR3C1	0.191812461	0.191535002
RAB18	0.191807178	0.191547469
FAM160B1	0.191802532	0.191558432
ZC3H12C	0.191780178	0.191611182
MYL12A	0.191734351	0.19171936
PSMG1	0.191567522	0.192113531
PNPLA8	0.191493424	0.19228879
MREG	0.191383935	0.192547965
PBRM1	0.191348156	0.192632711
SEPSECS	0.191216659	0.192944407
USP1	0.191016164	0.193420341
JRKL	0.190926604	0.193633207
IRGQ	0.190900906	0.193694317
CTSC	0.190794742	0.193946921
TSC22D3	0.190634513	0.194328607
SERPINB1	0.190577085	0.194465538
MFAP1	0.190575081	0.194470317
RRP36	0.190432643	0.194810245
FOXD2-AS1	0.190324491	0.195068633
GOSR1	0.190280973	0.19517267
MRPS35	0.190233255	0.195286794
RP4-647J21.1	0.19023038	0.19529367
KLF3	0.190174952	0.195426296

FBXO28	0.190153434	0.195477802
CDV3	0.190150676	0.195484404
CLIC1	0.190125406	0.195544901
CNOT6	0.190004296	0.195835035
ITGA1	0.189977584	0.195899068
MMAA	0.1898919	0.196104566
QPRT	0.18986765	0.196162753
DLAT	0.189796074	0.19633457
N4BP2L1	0.189687232	0.196596046
SGPL1	0.189672054	0.196632526
EXOC4	0.189655673	0.196671907
UCK2	0.189552127	0.196920956
SLC25A26	0.189501218	0.197043485
TMEM167A	0.189472065	0.197113674
MAP4K1	0.189412252	0.197257738
GLRX3	0.189368847	0.19736233
MYL12B	0.189292909	0.197545409
NUGGC	0.189245177	0.197660547
KPNA3	0.189179641	0.197818709
ZMYND11	0.189178899	0.1978205
ZRSR2	0.189166125	0.197851338
ZBED4	0.189157743	0.197871576
AC104820-2	0.189064588	0.19809659
KPNA1	0.188792844	0.198754013
ARFIP1	0.188731471	0.198902705
KCTD12	0.18853782	0.199372389
ANXA4	0.188498506	0.199467837
TXNDC15	0.188487297	0.199495056
ST7L	0.188486978	0.199495831
CITED2	0.1884517	0.199581517
ANAPC15	0.188429439	0.199635599
SCNM1	0.188320372	0.199900724
ZFAND6	0.188310994	0.199923532
ACOT13	0.188299535	0.199951401

RP11-479G22.8	0.188298188	0.19995468
SOCS5	0.188241421	0.200092796
ZNF45	0.188238327	0.200100324
EPS8L2	0.188203131	0.200185993
LIN7C	0.188154212	0.200305109
ATP5EP2	0.188153299	0.200307331
USP46	0.187969758	0.200754692
NAA15	0.187941625	0.200823324
LARP4B	0.187928697	0.20085487
TUBGCP5	0.187765221	0.201254056
IFI27L2	0.187741319	0.201312469
TSEN15	0.187686248	0.201447098
RP11-159D12.8	0.187616787	0.201616998
CX3CR1	0.187605838	0.201643787
SPON2	0.187529124	0.201831564
PPIG	0.187481662	0.201947798
WDR12	0.187437349	0.202056365
KDM6A	0.187424741	0.202087262
PIM1	0.187405711	0.202133901
KRT10	0.187385861	0.202182561
RP11-327P2.5	0.1872219	0.202584796
ZNF670	0.187153468	0.202752845
RP11-121A8.1	0.186944294	0.203267115
NOL10	0.186836645	0.203532138
CSNK2A2	0.186828141	0.203553085
GCNT2	0.186771703	0.203692138
MACF1	0.186697819	0.203874274
UBE2G1	0.186683635	0.203909255
KLRAP1	0.186666275	0.203952072
SULT1A1	0.186645472	0.20400339
HNRNPA3	0.186636714	0.204024996
CLDND1	0.1866025	0.204109423
SCYL2	0.18648642	0.204396043
C5orf30	0.186362561	0.204702185

BLOC1S1	0.186310937	0.204829878
ZNF622	0.186288843	0.204884546
DDX20	0.186223896	0.205045304
TAX1BP1	0.186220504	0.205053702
SLFN12L	0.186163757	0.20519424
PGGT1B	0.186147901	0.205233522
MSRA	0.186094713	0.205365323
RNFT2	0.186091352	0.205373656
TBC1D22B	0.18605288	0.205469032
SUMO2	0.186020381	0.205549623
TPM3	0.185888461	0.205876993
UAP1	0.185793716	0.206112335
ECHDC1	0.185492217	0.206862502
ITGAV	0.185448355	0.206971794
METTL6	0.185429317	0.207019247
DCBLD1	0.185414848	0.207055313
ZBTB42	0.185311734	0.207312483
KIAA2013	0.185227556	0.207522587
POC5	0.185199896	0.207591661
BPNT1	0.185142881	0.207734087
ACAA2	0.185113997	0.207806266
RP11-582E3.6	0.184847738	0.208472466
IDE	0.184847662	0.208472656
GRSF1	0.184773043	0.208659628
SAR1A	0.18476555	0.208678408
C6orf203	0.184698672	0.208846094
GAS8	0.184652934	0.208960828
C11orf95	0.184638997	0.208995797
PAAF1	0.184637279	0.209000107
CNIH4	0.184585869	0.20912914
RNF217	0.184480244	0.209394421
RAC1	0.184423763	0.20953637
CHN2	0.184306046	0.209832437
HCFC1R1	0.184275924	0.209908241

API5	0.184265531	0.209934403
UBL3	0.184246608	0.209982038
KLHL28	0.18418695	0.210132269
ZNF229	0.184181741	0.21014539
RRP15	0.184075041	0.210414282
TUBD1	0.184008325	0.210582532
C1QA	0.183946959	0.210737375
CHAF1B	0.183866476	0.210940575
DPM1	0.183823993	0.211047888
VPS16	0.183820286	0.211057254
MRPL15	0.183717987	0.211315833
RPA1	0.183651718	0.211483458
COX17	0.18357548	0.211676413
SERBP1	0.183389315	0.212148103
SAP18	0.183313549	0.212340284
RAB11A	0.183278488	0.212429257
CAP1	0.183197465	0.212634967
RSRC1	0.18313324	0.212798127
ARHGAP22	0.182992097	0.213157001
TXNL1	0.182808459	0.213624556
SBNO1	0.18262953	0.214080807
THAP8	0.182455474	0.214525286
SMARCAD1	0.182429933	0.214590562
TRAV22	0.182373205	0.214735595
ETV3	0.182246687	0.215059303
ARL1	0.182223601	0.215118407
RGS3	0.182210553	0.215151817
SMAD2	0.182104116	0.215424492
ATE1	0.182095469	0.215446653
FAM161B	0.182010494	0.215664532
GALC	0.181966573	0.215777208
SRSF1	0.181912438	0.215916145
CACYBP	0.181901807	0.215943434
ZADH2	0.181846148	0.216086358

OPLAH	0.181846096	0.216086493
PAFAH2	0.18175463	0.216321505
RMND5A	0.181657878	0.216570296
RQCD1	0.181588888	0.216747819
NBR1	0.181562747	0.216815112
NCBP2	0.181523169	0.216917019
SLC25A16	0.181508397	0.216955064
TMEM104	0.18147798	0.217033416
PCNA	0.181461068	0.217076989
PDXK	0.181339424	0.217390577
RPIA	0.181223917	0.217688636
PQLC3	0.181014664	0.218229326
LIN52	0.180925875	0.218459031
ZFP36L2	0.180878726	0.218581076
MT2A	0.180797275	0.218792026
NKRF	0.180710353	0.219017301
NTMT1	0.180628167	0.219230447
PAPSS2	0.180620188	0.219251149
GGT1	0.180591368	0.219325932
PDCL3	0.180552913	0.219425744
C11orf58	0.180430357	0.219744055
PDSS2	0.180429837	0.219745408
ADCY9	0.180365249	0.219913288
HHAT	0.180332931	0.219997326
GOLGA5	0.180222155	0.220285547
DDX6	0.180103943	0.220593405
CA5BP1	0.18008546	0.220641567
RBL1	0.180041857	0.220755213
SMAGP	0.180012939	0.220830607
ZNF222	0.179986403	0.220899809
ERCC5	0.179841986	0.221276678
SLA	0.17976244	0.22148445
FIGNL1	0.179758532	0.221494662
CASP7	0.179584774	0.221948999

CCNH	0.179538542	0.222069993
AC002467-7	0.179538487	0.222070137
ZMAT3	0.179478008	0.222228486
SDE2	0.179154455	0.223076962
NQO1	0.179120411	0.223166368
PDGFC	0.178975638	0.223546846
RBBP4	0.178970358	0.223560733
UTP3	0.178956545	0.223597057
RPA2	0.178942575	0.223633803
RP11-652L8.4	0.178864981	0.223837968
SOS1	0.178714574	0.224234082
SLC25A30	0.178708324	0.224250555
ZNF780A	0.178704803	0.224259833
AFAP1L2	0.178667374	0.224358491
ARPC2	0.178583542	0.224579567
PCMTD1	0.178539897	0.224694724
MECP2	0.178515723	0.224758527
TAF5L	0.178432928	0.224977136
ZNF140	0.178407623	0.225043981
UQCC3	0.178350183	0.225195761
RHOA	0.178241693	0.225482631
VSTM1	0.178230125	0.225513235
ZSCAN2	0.178147268	0.225732515
ATF7IP	0.178124764	0.225792097
YWHAH	0.178106413	0.225840691
TRMT6	0.177983634	0.226166005
ANP32E	0.17787535	0.226453181
PSME4	0.177719863	0.226865982
PRKX	0.177619891	0.227131669
CDC42SE2	0.177618172	0.227136239
TIMM17A	0.177387582	0.227749888
SNX5	0.177309888	0.227956904
GABPA	0.177288699	0.228013385
SMDT1	0.177272502	0.228056565

MAGOHB	0.177239442	0.228144721
PPP1R7	0.17719587	0.228260942
SOCS4	0.177175238	0.228315988
HMGCR	0.177145546	0.228395224
THBS1	0.177037681	0.228683226
RAD23B	0.176911733	0.22901983
SLC25A4	0.176883791	0.229094553
INTS7	0.176734307	0.229494586
TMED9	0.176684515	0.229627942
COPS4	0.176639028	0.229749814
SUCLA2	0.176608335	0.229832075
FBXW11	0.176592265	0.229875153
RBM15	0.176589443	0.229882717
PUS10	0.176423705	0.230327328
C1orf56	0.176380723	0.230442729
PRPF4	0.176214735	0.230888755
LINC00649	0.176189473	0.230956688
HNRNPF	0.176180613	0.230980517
IQCG	0.176170622	0.23100739
SHROOM1	0.176162627	0.231028898
MAGT1	0.175969307	0.231549332
TXNL4A	0.17596703	0.231555468
ACTR3	0.17593778	0.231634283
ADO	0.175865885	0.231828084
FPGT	0.175849974	0.23187099
ZMYM4	0.175792496	0.232026028
ZRANB1	0.175512202	0.2327831
CTDNEP1	0.175352814	0.23321436
TMEM242	0.17535179	0.233217135
DNAJC21	0.175278284	0.233416208
UBE2T	0.175243098	0.233511543
CCDC18	0.175185482	0.233667707
MRPL22	0.175116349	0.233855181
ASCL2	0.175097994	0.233904974

NCEH1	0.174948982	0.234309473
RTN4IP1	0.174889182	0.234471938
NEDD1	0.174864783	0.234538249
NSMAF	0.174852379	0.234571964
PLCB1	0.174848563	0.234582336
CD244	0.174846702	0.234587396
POLA1	0.174686827	0.235022269
CD8B	0.174645377	0.235135106
RAB10	0.174572525	0.23533352
KIAA1143	0.174418359	0.235753767
MYCBP	0.174266971	0.236166941
YTHDF3	0.174253016	0.236205052
GHITM	0.174247146	0.236221085
ZNF583	0.17424086	0.236238255
OTULIN	0.174048464	0.236764171
GOLGA4	0.17388731	0.237205306
SNTB2	0.173815234	0.237402782
TCF19	0.173799541	0.237445793
WDYHV1	0.173567703	0.238081836
NRP1	0.173566695	0.238084604
TOR1AIP1	0.173516748	0.238221788
ZNF236	0.173378982	0.238600447
TRIM56	0.173344867	0.238694279
HNRNPAB	0.173323948	0.238751826
TMX3	0.173292125	0.238839393
STAG2	0.173261467	0.23892377
MITF	0.173233498	0.239000767
TSC22D2	0.173186294	0.239130753
PRKACB	0.173130218	0.239285235
HNRNPK	0.172952074	0.239776446
LINC00324	0.172719281	0.240419376
VEZF1	0.172687446	0.240507392
NFYB	0.172592631	0.240769654
MYL6	0.17258041	0.240803473

KLHL7	0.172570304	0.240831443
DDX52	0.172537217	0.240923024
RP11-284N8.3	0.172536529	0.240924928
PSMB3	0.172498741	0.241029553
YTHDF1	0.172487311	0.241061205
ZNF544	0.172395013	0.241316906
G3BP2	0.172385097	0.241344388
ANKRD39	0.172353076	0.241433146
ZNHIT6	0.172319339	0.241526686
PGRMC2	0.17229905	0.241582953
TMEM57	0.172261936	0.241685901
DENND4A	0.172248848	0.241722215
CTD-2006C1.2	0.172144179	0.24201274
RFC3	0.172016899	0.242366348
GBP4	0.171878977	0.242749915
HPS3	0.171857285	0.242810281
ICA1	0.171760033	0.243081036
SH3RF1	0.171557455	0.243645688
RP11-147L13.15	0.171515048	0.243764005
TRBV4-2	0.171330275	0.244279975
BTBD7	0.171189624	0.244673231
GNLY	0.171066121	0.245018897
SMARCE1	0.171022591	0.245140811
USP9X	0.17101027	0.245175325
IDH2	0.170993919	0.245221134
PAIP2	0.170964316	0.245304084
ERLEC1	0.170948093	0.245349549
RANGRF	0.170901421	0.245480384
LMTK2	0.170611169	0.246295098
EARS2	0.17053557	0.246507599
YWHAB	0.170480276	0.246663102
TERF2IP	0.170478769	0.246667341
ACTR6	0.170451351	0.246744476
METTL23	0.170353058	0.247021131

PDK4	0.170350699	0.247027775
GIMAP4	0.170291746	0.247193809
IFT22	0.170228477	0.24737208
GDI2	0.170218794	0.247399373
TMED10	0.170210609	0.247422444
IL2RG	0.170156719	0.247574379
RAB39B	0.170005228	0.248001826
SRP14	0.169943714	0.248175535
PRIM2	0.169849996	0.248440347
DNAJA1	0.169824848	0.248511436
CCDC152	0.169795117	0.2485955
UHMK1	0.169781439	0.248634182
TSPAN5	0.169662887	0.248969611
SGMS2	0.169648142	0.249011354
ENDOV	0.169508594	0.249406627
BROX	0.169470873	0.249513545
RABGGTB	0.169467035	0.249524426
RFTN1	0.169447195	0.249580677
WAPL	0.169348592	0.249860361
AK2	0.169339829	0.249885226
BCL7B	0.169285298	0.250040003
HMGCL	0.169201478	0.250278037
USP13	0.169200016	0.25028219
SEC61B	0.169186363	0.250320974
KLF12	0.169138862	0.250455954
C19orf68	0.169130805	0.250478851
SDHD	0.169088709	0.250598519
PPP3R1	0.169063162	0.250671161
RP11-403P17.6	0.169057005	0.250688672
CLTC	0.169035826	0.250748907
AC147651-4	0.168981865	0.250902421
UQCRC2	0.168905626	0.251119425
FGFR1OP2	0.16884581	0.251289772
PIGA	0.168776578	0.25148703

RNF8	0.168745927	0.251574394
RNF138	0.168704724	0.251691867
MRPL1	0.168647697	0.251854518
MRFAP1L1	0.168502106	0.25227009
METTL21B	0.168495985	0.252287572
MIB1	0.168458922	0.252393442
TCEA1	0.168456386	0.252400689
IGFBP3	0.168410759	0.252531064
ALG5	0.168344398	0.252720769
SRP72	0.168273925	0.252922335
SFR1	0.168089763	0.253449581
JMY	0.167972242	0.253786426
ZSCAN29	0.16792984	0.253908033
EXOSC6	0.167892253	0.254015866
PDXDC1	0.167887747	0.254028794
HSPH1	0.167883276	0.254041623
SLC7A1	0.167852637	0.254129552
SH3BP5	0.167827864	0.254200659
PUDP	0.167816647	0.254232861
PHF6	0.167704722	0.254554325
GTF3C6	0.167672711	0.254646313
PDSS1	0.167655853	0.254694769
VAV3	0.167639277	0.254742417
SCFD1	0.167525472	0.255069724
EPT1	0.167445618	0.255299555
NEB	0.167406073	0.255413421
FAM173B	0.167289121	0.255750377
CEP192	0.16719041	0.25603501
HENMT1	0.167153916	0.256140294
PEX13	0.16714866	0.256155461
C4orf48	0.167126537	0.256219301
TMEM170A	0.167108335	0.256271836
SLCO4C1	0.167059731	0.256412151
PURB	0.16705732	0.256419113

SIRT2	0.167006737	0.256565199
CTR9	0.16693853	0.256762269
RALGDS	0.166933222	0.256777611
PTPN7	0.166924005	0.256804251
C21orf59	0.166844594	0.25703385
PKP4	0.166842329	0.257040399
AGL	0.166787438	0.257199186
RAB11FIP5	0.166622581	0.257676477
LRRC37A2	0.166611462	0.257708691
WDHD1	0.166581056	0.257796792
UBQLN1	0.166530417	0.257943568
GMNN	0.166376027	0.258391403
ZNF234	0.166367318	0.258416682
PIK3C2A	0.166343518	0.258485767
SCD5	0.166333357	0.258515268
INTS6	0.166331121	0.258521759
SERPINB6	0.166282217	0.258663771
STAU2	0.16617318	0.258980589
VEZT	0.166123786	0.259124194
YOD1	0.16611398	0.259152709
OSTM1	0.166048526	0.259343103
TPRKB	0.166045827	0.259350955
APMAP	0.165915539	0.259730226
DHRS4-AS1	0.165855197	0.259906006
PHTF1	0.16582912	0.259981996
WIPF2	0.165795481	0.260080044
ZFP14	0.165625105	0.260577017
GYS1	0.165460132	0.261058834
GIMAP2	0.165441701	0.2611127
ATP5I	0.165413489	0.261195165
FCF1	0.165399778	0.261235251
PRKAR1A	0.165336859	0.261419252
DUSP10	0.165314855	0.261483619
OLA1	0.165198053	0.261825482

CUTC	0.165173662	0.26189691
PTPRS	0.16515146	0.261961937
C1orf233	0.165101419	0.26210854
MINPP1	0.165070744	0.262198437
S100A6	0.16495008	0.262552251
FGD5-AS1	0.164878953	0.262760959
SENP6	0.164875601	0.262770799
TCEANC	0.16486783	0.26279361
FAF2	0.164790381	0.263021015
ZNF684	0.16477799	0.26305741
HPGD	0.164753958	0.263128005
STRAP	0.16465558	0.26341713
WNK1	0.164595758	0.263593045
TAF1	0.164572136	0.263662531
TTC22	0.164536938	0.26376609
AP003774-4	0.164352954	0.26430785
SMARCC1	0.164321609	0.264400223
AOAH	0.164299064	0.264466676
PRDX3	0.164249234	0.264613592
ANKRD50	0.164176827	0.264827172
SLFN13	0.163981771	0.265403096
CMSS1	0.163949923	0.265497212
B4GALT5	0.163924419	0.265572595
AIMP1	0.163879874	0.265704291
NOM1	0.163855051	0.265777699
TRAPPC1	0.163733896	0.26613618
POLR2G	0.163719373	0.266179172
FAM13B	0.163637769	0.266420833
POP1	0.163598635	0.266536778
TRBJ2-2P	0.163565715	0.266634336
ARHGAP35	0.163515509	0.266783169
CXorf38	0.163486553	0.26686903
SLC29A3	0.163441885	0.267001522
ARHGAP11B	0.163288221	0.267457635

PTPN12	0.1632829	0.267473441
RP11-81H14.2	0.16327497	0.267496993
TRAF6	0.163264021	0.267529515
TRIT1	0.163204263	0.267707066
CDC16	0.163188413	0.267754171
FBXO32	0.163181925	0.267773456
ZNF394	0.163145733	0.267881041
FARSA	0.163046908	0.268174963
SSR1	0.162946684	0.268473265
ISOC1	0.162864014	0.268719484
8-Sep	0.162842674	0.268783066
SLC25A43	0.162813868	0.26886891
LRRC40	0.162800284	0.268909395
DBF4	0.162751122	0.269055958
PPP6C	0.162727911	0.269125172
PSMB1	0.162543137	0.269676588
KLHL42	0.162541867	0.269680381
NEK1	0.162421872	0.270038882
HNRNPD	0.162329576	0.270314844
MLC1	0.162261599	0.270518211
C17orf89	0.162257336	0.27053097
PON2	0.162255708	0.270535842
NFE2L2	0.162239692	0.270583773
PDLIM5	0.162230686	0.27061073
HRAS	0.162134453	0.270898871
LYSMD3	0.161985534	0.271345164
TTC38	0.16195854	0.271426114
RP11-620J15.3	0.161889269	0.27163392
RHBDD1	0.16184348	0.271771338
SUMF1	0.161826969	0.271820901
PMVK	0.161737838	0.272088561
WIPF1	0.161686193	0.27224373
EMC7	0.161660108	0.272322125
TRAV35	0.161566163	0.272604589

THUMPD1	0.16155326	0.272643402
OPA1	0.161534301	0.272700432
SH3BGRL3	0.161476233	0.272875164
AGPAT4	0.161465911	0.272906232
SEL1L3	0.161447264	0.272962362
TMCO1	0.161436247	0.272995528
ZNF254	0.161424315	0.27303145
RNF168	0.161380015	0.273164855
TIMM23	0.161359798	0.27322575
SUMO1	0.161309416	0.273377539
ZXDB	0.161231678	0.27361186
TRAV26-2	0.161195738	0.273720234
CGGBP1	0.161175832	0.273780275
METTL20	0.161061276	0.274125954
NDUFS1	0.161044143	0.274177678
ERP44	0.161043712	0.27417898
PGAM1	0.160807959	0.274891378
TRERF1	0.160789364	0.27494762
SP3	0.160787999	0.274951747
HSPA8	0.160785656	0.274958836
STRN3	0.160714672	0.275173608
DDX21	0.160621965	0.275454274
COPS2	0.160533447	0.275722435
ATP5C1	0.160491679	0.275849029
SNX14	0.160430163	0.276035547
PLB1	0.160394169	0.276144719
RB1	0.16029561	0.276443802
NFKBIB	0.160212545	0.276696033
ZNF585B	0.160170127	0.276824895
KTN1	0.160120732	0.276975006
DAXX	0.160090813	0.277065953
KBTBD6	0.160076038	0.277110873
MDFIC	0.159809644	0.277921616
ATG13	0.159777767	0.278018733

BDP1	0.159776359	0.278023023
BMP1	0.159744699	0.278119504
BCLAF1	0.15969699	0.278264934
CEP290	0.159671663	0.27834216
TFAP2E	0.159365432	0.279276995
ELMO2	0.159349219	0.279326548
PCNXL4	0.159333274	0.279375285
VTI1A	0.159272924	0.279559804
MEI1	0.159194807	0.279798761
CMTM3	0.159133022	0.279987855
SNRNP27	0.159126473	0.280007903
DGKH	0.159089278	0.280121785
ABCB7	0.158980791	0.280454119
BTBD1	0.1588765	0.280773844
SEC23A	0.158804161	0.280995753
SPRY2	0.158798983	0.281011642
FAM188A	0.158690342	0.281345143
LPAR5	0.158655096	0.281453394
FAM21A	0.158629979	0.281530552
ZNF330	0.158572896	0.281705962
RHOU	0.158506595	0.281909789
ZNF567	0.158356867	0.282370447
ZCRB1	0.158322371	0.282476646
EMG1	0.158277847	0.282613758
DUT	0.158023789	0.28339697
RHEB	0.157840958	0.283961482
SNX3	0.157825719	0.284008568
TESK1	0.157821706	0.284020966
PIK3R1	0.157517397	0.284962316
GADD45A	0.157505858	0.284998049
PSMB2	0.157463587	0.285128981
PLEKHA2	0.157456503	0.28515093
HDAC8	0.157445033	0.285186464
POLR2K	0.157378022	0.285394137

PRKCB	0.157362718	0.28544158
PLS1	0.15736228	0.285442938
GTF3A	0.157272415	0.285721622
DIS3	0.157240282	0.285821314
STARD7	0.157057748	0.286388055
CINP	0.156950734	0.286720659
TRIM24	0.156939179	0.286756587
ARF1	0.15692466	0.286801735
RMDN2	0.156912935	0.286838198
SYNE2	0.156833852	0.287084219
ACP5	0.156751122	0.287341734
PRAF2	0.156676033	0.287575593
KIAA1324	0.156632506	0.287711213
DLEU1	0.156614967	0.287765871
ING3	0.156593348	0.287833255
RP11-97C16.1	0.156554492	0.28795439
CD1C	0.156526867	0.288040529
ZNF790	0.156526477	0.288041745
RP11-747H7.3	0.156464371	0.288235471
LRIF1	0.156452493	0.288272529
C1orf50	0.156255568	0.288887399
MRPL9	0.156252583	0.288896725
PLA2G16	0.15617668	0.289133955
ARFGAP3	0.156082279	0.289429176
ATP5J	0.155999943	0.289686825
FZD3	0.155871753	0.290088258
SPAG7	0.155822951	0.290241182
CCDC53	0.155785103	0.290359813
GPKOW	0.155713816	0.290583347
HOOK3	0.155708298	0.290600653
APOBEC3D	0.155678703	0.29069349
ADK	0.155659555	0.290753566
CNOT4	0.155658982	0.290755365
PPIL4	0.155566098	0.291046897

PAFAH1B1	0.155555386	0.291080531
IQGAP1	0.155524818	0.291176523
BHLHB9	0.155474311	0.291335174
COMMD5	0.155471356	0.291344458
HELZ	0.155438359	0.29144814
MORF4L1	0.155351901	0.291719916
PLOD1	0.155266395	0.291988865
ZNF2	0.155245432	0.292054825
PPIE	0.155227136	0.292112404
HLA-K	0.155208101	0.292172312
GSS	0.15517597	0.29227346
DENR	0.155052208	0.292663269
LIMA1	0.155035065	0.29271729
RFT1	0.155009671	0.292797323
PARP11	0.155001569	0.29282286
PAPD4	0.154990577	0.292857509
NMNAT1	0.15492395	0.293067595
RP11-799B12.1	0.154685322	0.293820818
GALNT1	0.15466728	0.293877817
MFSD8	0.154639581	0.293965341
B3GNT2	0.154606644	0.294069436
NLRC3	0.154586851	0.294132003
RDX	0.154569313	0.294187451
KLRB1	0.154566093	0.29419763
SLC4A7	0.154535195	0.294295333
LPXN	0.154475139	0.294485295
FKBP5	0.154461955	0.294527008
ULK4	0.154415147	0.294675131
ZNF786	0.154352072	0.294874811
MRPL10	0.154341895	0.29490704
SQLE	0.154307583	0.295015707
ATPAF1	0.154248543	0.29520275
LRRC58	0.154135876	0.2955599
SSBP4	0.15409022	0.295704707

TRBJ2-3	0.154040723	0.295861749
ZFP92	0.154009369	0.295961256
MAD2L1BP	0.153931575	0.29620824
RFC5	0.153815383	0.296577382
LINC00243	0.15378175	0.29668429
ZNF501	0.153770875	0.296718863
GSTCD	0.153722445	0.296872858
TIMM10B	0.153627209	0.29717584
LINC01232	0.153620639	0.297196749
C5orf15	0.153615568	0.297212889
RP5-821D11.7	0.153563966	0.297377151
MGAT3	0.153541443	0.297448863
CTTNBP2NL	0.153505392	0.297563676
TBC1D15	0.153466939	0.297686168
THUMPD2	0.153428839	0.297807568
UBE2V2	0.153424788	0.297820477
KLRC1	0.153370145	0.297994653
SHFM1	0.153281015	0.298278893
CASZ1	0.153255951	0.298358856
CCDC28A	0.153103138	0.298846676
AFAP1	0.153074561	0.29893796
NEK4	0.152984832	0.299224697
MOB4	0.152950825	0.299333414
SLC25A40	0.152909995	0.299463979
SH3BGRL	0.152853524	0.299644621
VPS41	0.152830487	0.299718332
HIST1H4I	0.152581165	0.300516839
FAM199X	0.152475351	0.300856147
TRPS1	0.152466449	0.300884705
FAM69A	0.152424821	0.301018266
MFSD11	0.15241538	0.301048561
NDNL2	0.152365701	0.301208015
ZNF585A	0.15229497	0.30143513
NUS1	0.152223395	0.30166507

NFIX	0.152223279	0.301665441
COX6C	0.152206805	0.301718381
CCND3	0.152200126	0.301739847
FAM49B	0.152149681	0.301901997
ELP3	0.152137268	0.301941908
TUBB	0.152115111	0.302013154
LMBRD2	0.152109973	0.302029674
TGFBRAP1	0.152102037	0.302055196
PDGFB	0.151845328	0.302881503
CSNK1G1	0.151783777	0.303079844
USP12	0.151768717	0.303128384
RPN1	0.151626407	0.303587324
CTC-444N24.11	0.151615568	0.303622296
ST6GALNAC6	0.151540119	0.303865815
DYNC1I2	0.151455852	0.30413794
CDCA7	0.151443855	0.304176694
SRPRB	0.151413263	0.304275532
ZNF41	0.151413123	0.304275984
SPTY2D1	0.15138585	0.30436412
ZNF570	0.151371052	0.304411945
RPA3	0.151327007	0.304554327
MAN1A1	0.15128416	0.304692875
CLEC9A	0.151223532	0.304888986
NR1D2	0.151216081	0.304913094
GMFB	0.151206501	0.304944093
NUP153	0.151187566	0.305005367
LINC01420	0.151120145	0.3052236
MANEA	0.15108485	0.305337888
ASRGL1	0.15107801	0.305360038
SAMSN1	0.151038376	0.305488415
CFAP97	0.150882447	0.305993808
SHQ1	0.150377711	0.307633415
SYTL3	0.15036965	0.307659648
SPPL2A	0.150280936	0.307948425

CXCR6	0.150228538	0.308119073
ITPRIPL2	0.15022467	0.30813167
ROCK1	0.150172158	0.308302753
PPP3CA	0.150162431	0.308334451
SNRPD1	0.150038363	0.308738926
TIMM21	0.149972965	0.308952267
GPBP1	0.149839313	0.30938856
RNF167	0.149820092	0.309451338
MTMR6	0.149762112	0.309640754
BHLHE40	0.149760873	0.309644804
ZNRF2	0.149692281	0.309868986
DNASE1L3	0.149603114	0.31016057
STYX	0.14958327	0.310225484
ARL5A	0.149408891	0.310796301
PUM3	0.149357885	0.310963395
GPX7	0.149218089	0.311421645
LCP1	0.149113549	0.31176461
TOP2B	0.149073583	0.311895791
COX5A	0.149064446	0.311925786
PSMC2	0.149036265	0.312018311
SSB	0.148980461	0.312201581
DYRK1A	0.148821946	0.312722546
AIDA	0.148816874	0.312739222
ARHGAP24	0.148794126	0.312814035
FTSJ3	0.148792601	0.31281905
SNRPF	0.148728512	0.313029878
PTGES3	0.148681652	0.313184085
XPOT	0.148673794	0.313209952
DLG1	0.148672305	0.313214852
FAS	0.148667593	0.313230363
GINS4	0.148643498	0.313309683
POLE4	0.148598361	0.313458305
ATPIF1	0.14859324	0.313475168
CXCR4	0.148438634	0.313984597

BCAT1	0.1484116	0.314073727
CYTH3	0.148398395	0.314117273
RPS27L	0.148390113	0.314144583
FLI1	0.148387633	0.314152762
STAM	0.148367084	0.314220535
TNFSF14	0.148319033	0.314379045
TMEM167B	0.148313255	0.314398111
ICE2	0.14831279	0.314399645
RP11-147L13.8	0.148162634	0.314895328
APOO	0.148067381	0.31521003
LZIC	0.148059652	0.315235575
KIAA1586	0.148009555	0.315401174
CROT	0.147751987	0.316253466
HLTF	0.147748078	0.316266412
ASB6	0.147543788	0.316943462
S1PR1	0.147513239	0.317044785
EIF1AX	0.147489815	0.317122492
CDK11B	0.147460056	0.317221228
PAK1IP1	0.147456553	0.317232854
CLOCK	0.147371593	0.317514857
RAP1A	0.147310189	0.317718775
EED	0.147283192	0.317808453
C2orf44	0.14727937	0.31782115
UFL1	0.147218673	0.318022842
ABAT	0.147215346	0.318033897
SMIM15	0.147178393	0.318156733
UBA2	0.147053323	0.318572694
MTFMT	0.147003421	0.318738755
MAPK9	0.146934597	0.318967876
SNRK	0.146853454	0.319238138
CANX	0.146806765	0.319393712
RP11-156E6.1	0.146728752	0.319653768
SH2D1B	0.14669916	0.319752446
TMF1	0.146675561	0.319831155

ECT2	0.146653657	0.319904221
PPP4R1L	0.146638104	0.319956108
RP11-571F15.3	0.146536899	0.320293876
CCNDBP1	0.146533241	0.320306089
PPP3CC	0.146380911	0.320814921
OSTC	0.146332473	0.320976827
NDUFB6	0.146278707	0.3211566
SMC1A	0.146266779	0.321196494
WDR54	0.146254613	0.321237184
C11orf30	0.146111658	0.321715562
TMBIM6	0.145965894	0.322203801
RNF2	0.14595796	0.322230391
FAM222B	0.145912584	0.32238248
PSMA6	0.14576285	0.32288468
GPAM	0.145704643	0.323080037
DIAPH1	0.145587663	0.323472872
PRKAA1	0.145578786	0.323502695
TMEM106B	0.145576181	0.323511447
C2CD5	0.145568244	0.323538114
UBR1	0.145566884	0.323542684
MIOS	0.145531939	0.323660108
FLCN	0.14546397	0.32388858
SRP54	0.145447912	0.323942575
TOMM22	0.14536656	0.324216195
MEA1	0.145211507	0.324738104
PPP1R2	0.144922597	0.325711988
ABCD2	0.144863179	0.325912505
PPP4R3B	0.14480012	0.326125396
ZNF793	0.144770549	0.32622526
GGH	0.144764483	0.326245748
SNX16	0.14470584	0.326443853
PPTC7	0.144535363	0.327020185
CSNK1G3	0.144532474	0.327029957
AP1AR	0.144490925	0.327170524

SLC39A8	0.144351723	0.327641732
IKBIP	0.144344551	0.327666022
C1QTNF3	0.144299388	0.327819
DMPK	0.14411311	0.328450446
FARP2	0.144099658	0.328496076
TAGAP	0.144077625	0.328570819
ITGAL	0.144046074	0.328677872
TMPO-AS1	0.144027029	0.3287425
GLT8D1	0.143947292	0.329013178
RAD18	0.143940457	0.329036385
ADHFE1	0.14387924	0.329244296
LNPEP	0.143830275	0.329410656
C5orf24	0.143815308	0.329461517
HDDC2	0.143804399	0.329498591
SAMM50	0.143784018	0.329567865
UFSP2	0.143742147	0.329710205
TSHZ1	0.143588065	0.330234343
AP5M1	0.143562621	0.330320946
DLGAP1-AS1	0.143547377	0.330372841
INIP	0.143509925	0.330500351
PSMD14	0.14348802	0.330574945
RSU1	0.143429819	0.330773192
C19orf43	0.143350909	0.331042093
STIP1	0.143350412	0.331043788
AC016747-3	0.143317387	0.331156367
MRPL3	0.143274947	0.331301078
GGCT	0.14322307	0.331478022
PYROXD1	0.143207448	0.331531317
COA6	0.143185521	0.331606134
CMTM1	0.143176806	0.331635871
GDE1	0.143176543	0.331636768
SLC35E1	0.143055008	0.332051658
POLR2M	0.142986798	0.332284649
BLOC1S6	0.142894553	0.332599907

ERBB2IP	0.142800621	0.332921118
OSBPL3	0.142797678	0.332931187
NGLY1	0.142789057	0.332960678
HS2ST1	0.14278662	0.332969013
MAF1	0.142759176	0.333062908
THAP3	0.142698189	0.333271618
PFKP	0.142684929	0.33331701
DGCR8	0.142664529	0.333386845
PFN1	0.14258689	0.333652721
PXYLP1	0.142568962	0.333714134
HDAC2	0.14252096	0.3338786
SMAD7	0.142459406	0.334089576
RASGRP1	0.142423014	0.334214347
CCDC59	0.142347794	0.334472334
MZT1	0.142308798	0.334606131
MED13	0.142204315	0.334964777
HIVEP3	0.142183477	0.335036334
IBTK	0.142046495	0.335506963
ZNF268	0.142041917	0.3355227
ITGA4	0.141994729	0.335684922
PARP2	0.141961041	0.335800765
FCRL3	0.141835702	0.336231989
SETD3	0.141768408	0.336463653
SLC25A12	0.141673583	0.336790261
ADIPOR2	0.141630895	0.336937356
KIF3B	0.141622708	0.336965573
BRIX1	0.141581011	0.337109299
FRG1HP	0.141578071	0.337119437
USP39	0.141537908	0.337257913
BCCIP	0.141498241	0.337394716
PSMB4	0.141362514	0.33786307
MANF	0.141332616	0.337966292
COX16	0.141293889	0.338100029
HINT2	0.141190113	0.338458558

ICE1	0.141158612	0.338567434
RNF111	0.141156834	0.33857358
DHX40	0.141110368	0.338734223
ADA	0.14101457	0.339065567
RBM4B	0.140929476	0.339360055
TRAPPC11	0.140924693	0.339376613
SYTL2	0.140834082	0.339690376
PRKRIP1	0.140799909	0.339808758
TP53INP1	0.140766533	0.3399244
RCHY1	0.140760792	0.339944296
RDH14	0.140655822	0.340308176
SYNCRIP	0.140573823	0.340592594
SETBP1	0.140566641	0.340617514
STK16	0.140493241	0.340872242
RBBP5	0.140368511	0.341305379
TBL1XR1	0.140322592	0.341464923
DCP1A	0.140319557	0.341475468
EID2	0.140284018	0.341598983
PPM1G	0.140259913	0.341682774
HOTAIRM1	0.140019609	0.342518781
EFCAB11	0.13998578	0.342636575
EIF2S2	0.139956584	0.342738252
VOPP1	0.139919428	0.342867681
SEC23IP	0.139856637	0.343086475
CSNK1A1	0.139838663	0.343149121
TNPO1	0.139824891	0.343197126
TMPO	0.139789109	0.34332187
JAZF1	0.139783996	0.343339697
HECW2	0.13977639	0.343366218
NUFIP1	0.139717924	0.343570121
S100A12	0.139660601	0.343770113
PLEKHA3	0.139630973	0.343873505
RB1CC1	0.13960253	0.343972782
RINL	0.139583227	0.344040169

PPARA	0.139513843	0.344282449
RBBP7	0.139493501	0.344353502
SLC17A5	0.139444347	0.344525228
ARFGEF2	0.139443048	0.344529769
BABAM1	0.139440053	0.344540232
ZNF430	0.139372138	0.344777597
SNX30	0.139256384	0.34518239
SELT	0.13910299	0.345719263
ZW10	0.139072154	0.345827251
TOB2	0.139053683	0.345891946
NDUFB10	0.139039376	0.345942062
AUH	0.139032743	0.345965297
GIMAP8	0.138819631	0.34671236
TIGAR	0.138810488	0.346744436
TOMM40	0.138728154	0.34703334
RFX7	0.138676021	0.347216345
SEC62	0.138627263	0.347387561
RNF113A	0.138572302	0.347580618
UNG	0.138558766	0.347628174
DSN1	0.138510564	0.347797558
COQ3	0.138494811	0.347852925
S100A4	0.138358718	0.348331484
NAA38	0.138334938	0.348415147
FDX1	0.138139536	0.349103069
DLEC1	0.138133993	0.349122596
WRB	0.138125238	0.34915344
CEP128	0.138110084	0.349206827
PLAA	0.138051521	0.349413201
RHNO1	0.138050989	0.349415078
DNTTIP1	0.137999912	0.349595133
LZTFL1	0.137945566	0.349786775
QKI	0.137836347	0.350172109
ZNF649	0.137831258	0.350190069
RAB5C	0.137732368	0.350539199

HAT1	0.137710194	0.350617516
DCTN5	0.137688654	0.350693598
GTF2A1	0.13759516	0.351023964
PIGN	0.137535907	0.351233435
GLUD1	0.137417553	0.35165207
BRWD1	0.137362052	0.35184849
ABI3	0.137340882	0.351923431
ZNF354B	0.137313972	0.352018703
ZNF616	0.137273157	0.352163233
CCT2	0.137244831	0.35226356
CHID1	0.137240499	0.352278905
ZBTB2	0.137204623	0.352406005
RAB2B	0.137162168	0.352556443
ZNF148	0.137128739	0.352674928
SERTAD2	0.137095822	0.352791622
CEP76	0.137010302	0.35309491
EAPP	0.136984395	0.353186816
CH507-9B2.5	0.136967322	0.353247391
NSRP1	0.136936953	0.353355159
ARHGAP11A	0.136842609	0.353690075
SDF2	0.136839928	0.353699593
RP9	0.136672382	0.354294861
PSMD5	0.136647633	0.354382842
ZBTB43	0.136581501	0.354618
ZMAT5	0.136544553	0.354749427
ECHDC3	0.136480991	0.354975592
MAPRE2	0.136444156	0.355106695
XBP1	0.136387008	0.355310159
CNOT11	0.136336007	0.355491796
ANKRD27	0.136303669	0.355606994
C3	0.136303294	0.355608329
SLC2A1	0.136269481	0.355728808
RAB30-AS1	0.136189887	0.356012506
NOL11	0.13617173	0.356077244

FAM92A1	0.136144134	0.356175648
NBAS	0.136065905	0.356454691
CYB561D2	0.136025436	0.356599098
FANCF	0.136007012	0.356664853
CNOT2	0.135855611	0.357205477
HNRNPA2B1	0.135709178	0.357728835
PHKB	0.135680743	0.357830518
PRRC1	0.13563584	0.357991123
APOPT1	0.135560596	0.35826035
SP140	0.135557656	0.358270872
RAB20	0.13555008	0.358297986
RAB21	0.135503404	0.358465069
SLC35D2	0.135366527	0.358955307
UBR2	0.135279597	0.359266869
URI1	0.135258411	0.359342824
LRRC8C	0.135250667	0.359370591
GGPS1	0.135218627	0.359485486
DNAJB1	0.13503877	0.360130859
HUS1	0.134994089	0.360291295
MRPS14	0.134882609	0.360691774
ZCCHC4	0.134870846	0.360734046
MDM1	0.134825907	0.360895575
TRDJ1	0.134776408	0.361073541
ZNF85	0.13463495	0.361582431
IGLV1-40	0.134564312	0.36183671
LYZ	0.134548173	0.361894819
YY1	0.134483555	0.362127546
MCAT	0.134472923	0.362165845
DHX33	0.134434959	0.362302628
AVL9	0.134416094	0.362370608
GNAI3	0.134382769	0.362490715
HEBP2	0.134361534	0.362567262
MAPRE1	0.134328052	0.362687973
PPIH	0.134276041	0.362875537

TMEM216	0.134210486	0.363112025
MRPL43	0.134186375	0.363199028
ARIH2	0.1341471	0.363340778
NANP	0.134060594	0.363653109
PAK2	0.133969547	0.363982008
NEDD9	0.133950591	0.364050509
CEP350	0.133873848	0.364327912
NDUFB7	0.13385476	0.364396926
WTAP	0.13384601	0.364428568
BUD31	0.133740793	0.364809161
FAM122C	0.133715278	0.364901493
CXorf57	0.133705485	0.364936936
XPA	0.13364018	0.365173329
SPSB1	0.133584196	0.365376056
H2AFV	0.13357289	0.365417007
RAB11FIP4	0.133545048	0.365517858
RNF20	0.133443022	0.365887573
CCDC90B	0.133439632	0.365899864
CLN6	0.133312375	0.366361332
CWC27	0.133288038	0.366449628
NDC80	0.133224764	0.366679241
EIF5A2	0.133213011	0.3667219
GATC	0.133197391	0.3667786
NCAM1	0.133184546	0.366825233
RAB37	0.133181863	0.366834974
PDCD2	0.133174807	0.366860591
SPRY3	0.133157135	0.366924755
PRC1	0.133139282	0.366989582
POLR2D	0.133130059	0.367023074
RANBP2	0.133125115	0.367041031
COPB1	0.133122534	0.367050403
MRPL51	0.133098773	0.367136705
CMAS	0.133075518	0.36722118
CDC40	0.13293068	0.367747576

HIF1AN	0.132836965	0.36808841
SRI	0.132704554	0.368570308
OSTF1	0.132557677	0.369105297
TBC1D5	0.132530001	0.369206155
SUV39H1	0.13251588	0.369257623
WRNIP1	0.132513505	0.36926628
CDK17	0.132428454	0.369576367
SIL1	0.132362256	0.369817824
TAF4B	0.132288354	0.370087495
GEMIN2	0.132234997	0.370282269
ZNF17	0.132225867	0.370315605
ATP5E	0.132176271	0.370496716
TNFRSF9	0.132088354	0.370817897
SASH3	0.132070734	0.370882287
CHEK2	0.132062081	0.370913908
AASDHPPT	0.132021696	0.371061524
SLIRP	0.132012589	0.371094814
ROMO1	0.131937517	0.371369323
INAFM1	0.131930389	0.371395393
KCMF1	0.131837808	0.371734108
REEP3	0.131821385	0.371794211
SUPT16H	0.131792988	0.371898151
CHI3L1	0.131698889	0.372242702
VDAC3	0.131609408	0.372570518
ACLY	0.131607926	0.372575949
DENND2D	0.13159789	0.372612728
KAT7	0.131578288	0.372684568
FPR3	0.131516521	0.372910997
UQCRH	0.131402312	0.373329886
DNAJC27	0.131353628	0.373508531
BRD7	0.131325218	0.373612807
MTDH	0.131274145	0.373800306
IARS	0.131263514	0.373839341
RAB33B	0.131225298	0.373979684

EIF4E	0.131214338	0.374019941
IL18BP	0.131188475	0.374114941
ERLIN2	0.131150414	0.374254779
MRPS36	0.131124532	0.374349886
SCAF11	0.131123668	0.37435306
ZNF8	0.130938145	0.375035227
EIF4A3	0.130896349	0.375189013
KIF20B	0.130855114	0.375340771
PCID2	0.130848586	0.375364803
HLA-L	0.130830319	0.375432045
C7orf60	0.130821798	0.375463413
CBX3	0.130781413	0.375612108
TMEM60	0.130776383	0.375630631
XPO7	0.130636681	0.376145291
ATP2A2	0.130616261	0.376220553
DCUN1D5	0.130612811	0.37623327
PRELID3B	0.130593026	0.376306201
DAPK1	0.130548413	0.376470685
PCED1B-AS1	0.130480029	0.376722895
INSR	0.13042234	0.376935738
SMURF2	0.130392279	0.377046676
RBM18	0.130334782	0.377258918
MYADM	0.130290541	0.377422277
ELP5	0.130248794	0.377576467
MIEF1	0.130174771	0.377849953
ZFX	0.130155679	0.377920512
FAR1	0.130139433	0.377980556
TC2N	0.130116803	0.378064208
METTL21A	0.13011404	0.378074421
USP14	0.1300527	0.37830122
THEM6	0.129975854	0.378585465
FAM49A	0.12995466	0.378663884
UBE2D3	0.129951716	0.378674778
RP11-16E12.1	0.129933505	0.378742166

DNAJB14	0.1299233	0.378779932
AP1S3	0.12987504	0.378958559
CEP95	0.129834518	0.379108587
NT5DC1	0.12977975	0.379311411
SNRPD3	0.129749623	0.379423008
ACYP2	0.129747258	0.379431771
STARD3NL	0.129695765	0.379622564
SLC16A6	0.129683813	0.379666854
CARNMT1	0.129537403	0.380209683
WDR76	0.129532744	0.380226963
BCAP29	0.129399681	0.380720717
FUNDC1	0.129388332	0.380762849
PRMT9	0.129350385	0.380903737
LIPN	0.129347029	0.380916196
SPATA2	0.129346893	0.380916704
ZDHHC20	0.129307335	0.381063609
CORO1C	0.129274235	0.381186557
ACBD3	0.129216179	0.381402259
ILKAP	0.129158195	0.381617768
PGM1	0.129098724	0.381838878
CERS5	0.129018082	0.382138822
ZNF718	0.128974463	0.38230112
ZWILCH	0.128952533	0.38238273
NECAB2	0.128909015	0.382544713
HIRIP3	0.12883481	0.382821013
FARSB	0.12883428	0.382822984
MPV17L2	0.128818659	0.382881166
SLC38A1	0.12875144	0.383131578
ILF2	0.128728537	0.38321692
PDS5A	0.128717645	0.383257512
NASP	0.128672033	0.383427519
ISCA1	0.128662674	0.383462407
NME1	0.128611193	0.383654354
MRPL39	0.128539748	0.383920831

AP001372-2	0.128499965	0.384069263
TERF2	0.12847592	0.384158989
PPP3CB-AS1	0.128413922	0.384390406
SGOL2	0.128372435	0.384545307
ZNF174	0.128344283	0.384650438
LARP4	0.128282299	0.384881978
APOL6	0.128271644	0.384921785
STAU1	0.128141722	0.3854074
HNMT	0.128059419	0.385715211
XPNPEP3	0.128059067	0.385716527
FBXO34	0.127938337	0.386168323
DR1	0.127891771	0.386342662
CEP120	0.12786638	0.386437746
LRP5L	0.127833237	0.386561879
PLXDC2	0.127831065	0.386570014
DYNLL2	0.127824081	0.386596176
ATP8A1	0.127821103	0.386607332
POGLUT1	0.127801852	0.38667945
CALCOCO2	0.127779598	0.386762828
CDC42	0.127757134	0.386847004
ATP6V1E1	0.127748928	0.386877756
TBCA	0.127637905	0.387293948
SFPQ	0.127613164	0.387386733
ZEB2	0.127537983	0.387668756
TBCK	0.127430423	0.388072449
RPL36AL	0.127428746	0.388078743
MAPK1IP1L	0.127374731	0.388281571
CDS2	0.127332806	0.388439038
ZNF211	0.127322454	0.388477928
ORMDL3	0.127292305	0.388591195
ZNF436	0.127284344	0.388621108
PERP	0.12725248	0.388740846
TYW1	0.127249331	0.388752683
FYCO1	0.127235934	0.388803034

DPCD	0.127218022	0.388870359
ZNF644	0.127211251	0.388895809
GAS6	0.127209563	0.388902155
IDI1	0.12719583	0.388953783
SPECC1	0.127165264	0.389068702
WDR89	0.12714049	0.389161858
EIF4E3	0.127123098	0.389227265
RAB2A	0.126977017	0.38977689
LY75	0.126909242	0.390032051
RNASEH2B	0.126892726	0.390094242
UTP18	0.126829338	0.390332994
MRPL30	0.126825215	0.390348527
UGGT1	0.126803616	0.390429903
RNGTT	0.126803198	0.390431477
CEP97	0.126796742	0.390455802
PFDN2	0.126775257	0.390536759
TMEM50B	0.126694325	0.390841812
FKBP14	0.126665398	0.39095088
CCR2	0.126478523	0.3916559
FH	0.126431588	0.391833089
NUPL2	0.126388763	0.391994803
USP7	0.126332357	0.392207863
HNRNPU	0.126327969	0.392224438
RSBN1	0.126319323	0.392257103
MESDC2	0.126234678	0.392576975
STX18-AS1	0.126230105	0.392594264
PIK3C3	0.126212532	0.392660692
ETF1	0.126075689	0.393178209
MEAF6	0.126013919	0.393411942
FBXO21	0.125943253	0.393679439
AC093323-3	0.125911336	0.393800294
CKAP5	0.125880923	0.393915471
UFC1	0.125879564	0.393920616
CSRP1	0.125815435	0.394163548

C12orf73	0.125789631	0.39426132
BRIP1	0.125788945	0.39426392
PXMP4	0.125757787	0.394382001
ERGIC2	0.125691553	0.394633079
YRDC	0.125677055	0.394688052
LGALS2	0.125631758	0.394859828
NDFIP2	0.125603526	0.394966914
CDKN2AIPNL	0.125598947	0.394984284
SLC38A9	0.125443184	0.395575424
TSPYL1	0.125377236	0.395825859
ITGB1BP1	0.12535041	0.395927754
NUP155	0.12520512	0.396479901
METAP2	0.125183961	0.39656035
KIAA0196	0.125056531	0.397045045
DDX3X	0.125030859	0.397142737
HSPA1A	0.125007194	0.397232798
TRAPPC9	0.124969722	0.397375433
CYCS	0.124957563	0.397421721
PHF20	0.124928779	0.397531317
UQCRQ	0.124872877	0.397744209
GTPBP4	0.124869592	0.397756721
EFCAB14	0.124855965	0.397808625
PDZD8	0.124830257	0.397906563
TTI2	0.124798663	0.398026938
LINC01006	0.124683229	0.398466942
RCAN1	0.124668066	0.398524756
PARN	0.124618781	0.398712722
PRELID1	0.124497059	0.399177164
RP11-403I13.8	0.124493201	0.399191887
CD1D	0.124455465	0.399335939
C12orf49	0.124428208	0.399440008
TBC1D19	0.12441755	0.399480708
PTTG1	0.124416979	0.399482886
GBE1	0.124394687	0.399568015

TIMM10	0.124394416	0.399569052
YME1L1	0.124119363	0.400620299
ACOT7	0.124101554	0.400688423
HLA-A	0.124056673	0.400860125
DRAM2	0.123949159	0.401271622
LYPLA1	0.123937928	0.401314621
TRPM7	0.12392356	0.401369635
SRP68	0.12387575	0.401552725
CCT6A	0.123832004	0.401720295
SLAMF8	0.123802501	0.401833332
CD109	0.123781301	0.401914567
NIPA2	0.123773972	0.401942653
SPTLC2	0.123763236	0.401983796
PPP1R8	0.123762958	0.401984862
CDC37	0.1237199	0.402149902
NUTM2B-AS1	0.123693222	0.402252173
LSM8	0.123663242	0.402367125
USF3	0.123628206	0.402501487
MOSPD1	0.123625653	0.402511277
PIK3CA	0.123529656	0.402879561
SSBP3	0.123511117	0.402950707
ATAD2	0.12350529	0.402973067
CAMKK1	0.123444868	0.403205002
HNRNPA1L2	0.123428605	0.403267443
EIF4H	0.123355086	0.40354978
ARPC4	0.123332637	0.403636013
MORC3	0.123282586	0.403828315
MOAP1	0.123245101	0.403972369
TRIM33	0.123228077	0.404037802
POLD3	0.123217045	0.404080209
DNAJC3	0.123189362	0.404186632
RLIM	0.123075447	0.404624727
KIF22	0.123067769	0.404654265
GATAD2A	0.123051967	0.404715062

MFSD2A	0.123011432	0.404871039
UTP23	0.122976355	0.405006039
FOXO3	0.122969195	0.405033601
DNAJC17	0.122945104	0.40512634
HIATL1	0.122935326	0.405163983
TRAM2-AS1	0.12292869	0.405189532
RPL9	0.122922177	0.405214608
CHCHD3	0.122898588	0.40530544
NSMCE4A	0.122838565	0.405536611
GTPBP10	0.122825117	0.405588417
CETN3	0.122817816	0.405616542
MGST2	0.12269954	0.406072343
SERP1	0.122693214	0.406096732
CDK2AP2	0.122596339	0.406470292
PSMG2	0.122531433	0.406720691
LONP2	0.122438529	0.407079254
SECISBP2L	0.122294893	0.407633976
GAB3	0.122279759	0.407692447
UTP15	0.122273394	0.407717042
CHI3L2	0.122249235	0.407810398
IL12RB1	0.122242551	0.407836228
SEC24A	0.122236647	0.407859043
KRCC1	0.122059033	0.40854581
MRPS9	0.121996985	0.408785882
RALA	0.121984581	0.408833884
EDF1	0.121948911	0.408971942
RSL24D1	0.121939283	0.40900921
SNIP1	0.121936196	0.40902116
LILRA4	0.121896834	0.409173551
IMMP1L	0.121853628	0.409340859
STK4	0.121786613	0.409600446
ATP11C	0.121767454	0.409674675
PPAT	0.12172214	0.409850274
FO538757-2	0.121653313	0.410117066

CLIC2	0.12163349	0.410193926
GPATCH2L	0.121623733	0.410231759
KDM4A	0.121468021	0.410835812
CMAHP	0.1214561	0.410882079
CASD1	0.121423116	0.411010108
ZNF12	0.121390217	0.41113783
PPP4R3A	0.121372914	0.411205013
TRBV14	0.121371224	0.411211574
GOLPH3L	0.121345276	0.411312338
SNRPB2	0.121321191	0.411405877
SKIV2L2	0.12130143	0.411482637
DDX10	0.121270103	0.411604333
FGD4	0.121237686	0.411730287
SEC61G	0.121204551	0.411859057
DPY30	0.121146289	0.412085527
TM9SF1	0.121123936	0.412172432
ZNF69	0.121087044	0.41231589
ZNF582	0.121013934	0.412600269
ELF1	0.120975229	0.412750865
EMC1	0.120920775	0.412962794
PRNP	0.120879586	0.413123137
ZBTB21	0.120870187	0.413159733
ICMT	0.120858444	0.413205456
SRSF10	0.120825356	0.413334306
ZBTB7A	0.120758566	0.413594461
DERA	0.120748922	0.413632034
LYRM2	0.120746062	0.413643175
FAM179A	0.120615024	0.4141539
IER3	0.120610831	0.414170245
PNRC2	0.120608595	0.414178963
GIN1	0.120468999	0.414723455
SYF2	0.120450333	0.414796293
ATP1B3	0.120437	0.414848324
PKI55	0.120413111	0.414941558

BBS9	0.120315851	0.415321276
NOP16	0.120288492	0.415428124
SOAT1	0.120262462	0.415529798
UEVLD	0.12021557	0.415712994
LXN	0.120154039	0.41595345
RAD51C	0.120109757	0.41612655
NIPBL	0.120105549	0.416143002
NSMCE2	0.120096461	0.416178534
BPI	0.120070818	0.416278796
RP1-228H13.5	0.120024967	0.416458111
TTC8	0.120005965	0.416532435
PDE12	0.119996388	0.416569899
PPP4R2	0.119975583	0.416651289
SLC39A7	0.119959425	0.416714505
ARL4A	0.119957066	0.416723735
PCGF6	0.119944246	0.416773897
ZNF669	0.119872884	0.417053189
UBE2R2	0.119860112	0.417103186
NR1H3	0.119823307	0.417247281
POMP	0.119817125	0.417271486
RILPL2	0.119735091	0.41759277
DNASE2	0.119690036	0.417769286
HACD2	0.119660541	0.417884864
RCN2	0.119655047	0.417906393
RP11-44F14.8	0.119590129	0.418160851
BLOC1S4	0.119574438	0.418222368
MCM6	0.119565738	0.418256479
AHCYL1	0.119451285	0.418705372
ACP1	0.119193838	0.419716101
RPL7L1	0.119125874	0.419983155
EFCAB7	0.118970312	0.420594776
RP11-498C9.15	0.118958672	0.420640563
SETX	0.118952697	0.420664064
UBE2N	0.118929208	0.420756468

SPDL1	0.118922216	0.420783977
VPS29	0.118918662	0.420797959
CUEDC2	0.118805702	0.421242524
CD2AP	0.118731815	0.421533454
CSTA	0.118660725	0.421813481
SPOP	0.118543887	0.422273941
CEP57	0.118426848	0.422735475
TRIM46	0.118422518	0.422752556
CLEC4D	0.118374045	0.422943796
ZNF217	0.11835853	0.423005016
CNOT7	0.118340989	0.423074236
MRPL44	0.118303966	0.423220363
NEIL2	0.118216842	0.423564339
LARP1B	0.118206199	0.423606368
DHRS4L2	0.118198922	0.423635109
PEF1	0.118151529	0.423822305
TP53RK	0.118144648	0.423849488
STMN1	0.118098243	0.424032835
TRGV7	0.118069868	0.424144969
OBFC1	0.118025407	0.424320702
PSMA5	0.117970466	0.424537917
RBM41	0.117960855	0.424575922
PHLPP2	0.117958835	0.424583912
CD69	0.117903423	0.424803063
ZNF782	0.117887714	0.424865204
UBE2B	0.117871121	0.424930846
NDRG1	0.117855955	0.424990848
UQCR11	0.11784107	0.425049744
SLC14A1	0.117820259	0.425132096
GPN1	0.117785617	0.425269196
SHC1	0.117673719	0.425712221
EIF4G2	0.117645856	0.425822575
SPCS3	0.117612872	0.425953233
CALM2	0.117608792	0.425969399

SUDS3	0.117554074	0.426186205
MPLKIP	0.117547331	0.426212928
CRISPLD2	0.117526312	0.426296229
SMNDC1	0.117523759	0.426306348
BRAP	0.117490435	0.426438438
ICA1L	0.117320456	0.42711256
IDH1	0.117313331	0.427140832
COL6A2	0.117258123	0.427359918
UGP2	0.117233543	0.42745748
POC1B	0.117223832	0.427496031
RAD51AP1	0.117057358	0.428157167
PAQR4	0.116845668	0.428998708
NOP10	0.116833919	0.42904544
TXLNG	0.116814744	0.429121719
ATP6V1A	0.116790298	0.429218973
FLVCR1-AS1	0.116779738	0.429260992
MDH1	0.116776558	0.429273642
ITPR2	0.116701807	0.429571134
TRMT1L	0.116679437	0.429660183
ATAD2B	0.116673247	0.429684824
FAM3C	0.116644365	0.429799815
TES	0.11664433	0.429799954
COPZ1	0.116566402	0.430110304
MTO1	0.116531276	0.430250235
MBNL1	0.116337838	0.431021287
FAM168B	0.116302908	0.431160602
EDRF1	0.116301794	0.431165047
DHCR7	0.116288285	0.431218935
ATRX	0.116282937	0.431240268
GRAMD1B	0.116252587	0.43136135
C2CD3	0.116223191	0.431478641
F8A1	0.11619876	0.43157614
PHYH	0.116148994	0.431774777
ITGB3BP	0.116103472	0.431956522

EIF1B	0.11602726	0.432260887
GCH1	0.115979151	0.432453081
C1GALT1	0.115950485	0.432567625
RNF145	0.115937276	0.432620409
DPP3	0.115924315	0.432672209
ZNF764	0.115872874	0.432877823
VAMP8	0.115871554	0.4328831
ESCO1	0.115851874	0.432961781
RP1-151F17.2	0.115845925	0.432985565
SAMHD1	0.115783592	0.433234827
UQCC2	0.115765991	0.433305225
VDAC1	0.115679354	0.433651838
SPN	0.115624411	0.433871732
KLHDC8B	0.115608888	0.433933873
C16orf54	0.115581157	0.434044889
MTIF3	0.115557111	0.43414117
CTC-444N24.8	0.115548731	0.434174724
BZRAP1	0.115535433	0.434227976
SUB1	0.115528847	0.434254352
NUP58	0.115501294	0.434364704
GOLT1B	0.115497655	0.434379279
COG3	0.115493785	0.434394779
PHF11	0.11545263	0.434559641
APOBR	0.115443373	0.434596729
PHAX	0.11543449	0.434632323
MRPL47	0.115402869	0.434759028
VSIG4	0.11535669	0.434944106
ERCC6	0.115294984	0.435191482
ELOF1	0.115281919	0.435243869
WLS	0.115255044	0.435351643
FAM98A	0.115242466	0.435402088
OPTN	0.115162418	0.435723199
ANP32A	0.115114818	0.435914209
ALG8	0.114933659	0.436641595

DNAJB11	0.114914193	0.436719791
BRE	0.114866801	0.436910211
MED14	0.114853326	0.436964362
MBD2	0.114808782	0.43714339
TXLNA	0.114738363	0.437426498
NOP58	0.114723178	0.437487557
TSR3	0.114689693	0.437622227
TLE4	0.114687034	0.43763292
TMCO4	0.114629875	0.437862854
MED21	0.114580532	0.4380614
MAN2B2	0.114572498	0.43809373
ADH5	0.11454221	0.438215636
KIF1BP	0.114522263	0.438295927
ITSN2	0.114511557	0.438339025
AC000123-2	0.114486547	0.438439714
RP11-706O15.1	0.114444986	0.438607068
USP3	0.114405967	0.438764216
SLC7A5	0.114401342	0.438782844
ATXN10	0.114364235	0.438932324
SRR	0.114358463	0.438955578
NFU1	0.114356459	0.438963653
ENOPH1	0.11428855	0.439237298
OIP5-AS1	0.114284808	0.439252381
KANSL1-AS1	0.114262151	0.439343703
SLC30A5	0.114228698	0.439478558
SLC9A7	0.114216186	0.439529002
VPS37A	0.114172368	0.439705685
ABI1	0.114166343	0.439729983
ZNF252P	0.11415848	0.439761694
FBXO2	0.114108238	0.439964344
IER3IP1	0.114053082	0.440186876
TTC30B	0.114027459	0.440290274
FAM26F	0.11397835	0.440488483
PEX14	0.113969563	0.440523957

CTBP1	0.113955357	0.440581306
PTEN	0.113928048	0.440691564
RFC4	0.113885504	0.440863363
SHMT1	0.113873252	0.440912844
RP11-325F22.2	0.113779672	0.441290887
TMEM170B	0.113689513	0.441655278
NDRG3	0.113641193	0.441850641
TFIP11	0.113591553	0.442051388
FAM114A1	0.11356468	0.442160087
UBR5	0.113474797	0.442523757
LRCH1	0.113431742	0.442698021
RIPK2	0.113408521	0.442792022
SAMD12	0.113308889	0.443195467
TNFRSF21	0.113194716	0.443658045
HIAT1	0.113176527	0.443731764
MITD1	0.113170003	0.443758204
WARS2	0.113069179	0.444166969
CNOT8	0.112950293	0.44464923
CTC-429P9.3	0.112936747	0.444704201
ARL15	0.112927068	0.444743477
NDUFA2	0.112912699	0.444801793
BET1	0.112867926	0.444983521
ZNF577	0.112866822	0.444988004
KEAP1	0.112840765	0.445093788
CDK2AP1	0.1128309	0.445133839
SLC16A7	0.112769612	0.445382715
CCDC134	0.112720936	0.445580433
ARMT1	0.11269443	0.445688118
RTF1	0.112692439	0.445696207
TMEM131	0.112691563	0.445699767
ALOX5AP	0.112636304	0.445924312
CTB-131K11.1	0.112629887	0.445950394
GLS	0.11261931	0.445993383
C1orf109	0.112542676	0.446304919

PELO	0.112534254	0.446339162
IMP3	0.112505713	0.446455223
CHST11	0.112470379	0.446598932
GK5	0.112429774	0.446764112
SERINC1	0.112417661	0.44681339
CHRNB1	0.112370708	0.447004446
PDE6D	0.112363106	0.447035385
PPM1B	0.112347476	0.447098996
COG5	0.112333007	0.447157885
MAP4K3	0.112304636	0.447273371
PCNP	0.112279068	0.447377462
ATP5G1	0.112239113	0.447540154
TIMMDC1	0.112234293	0.44755978
TLDC1	0.112155084	0.447882408
CTB-25B13.12	0.112152831	0.447891589
BLVRA	0.112140354	0.44794242
ZNRD1	0.111974752	0.448617398
ATAD3A	0.111879124	0.449007426
WHAMMP3	0.111796989	0.449342566
C16orf62	0.111796699	0.44934375
ITPA	0.111766019	0.449468972
REST	0.111726643	0.449629715
RP11-582J16.4	0.111675065	0.449840316
LDOC1L	0.111617995	0.450073404
EIF2S1	0.111584735	0.450209277
H3F3A	0.11156833	0.450276304
C16orf45	0.11156335	0.450296652
SLC4A1AP	0.111555301	0.450329539
UBE2O	0.11153572	0.450409553
CHEK1	0.111393837	0.450989565
TYMS	0.11132445	0.451273365
HNRNPH2	0.111181342	0.451858999
FAM50B	0.111180598	0.451862048
ST6GALNAC1	0.111171088	0.451900978

HEXIM1	0.111129261	0.452072234
RPS2P5	0.111064453	0.452337648
NUFIP2	0.111057258	0.452367121
ZNF324	0.111056558	0.452369986
DUSP5	0.111031454	0.452472825
NDUFS6	0.11100853	0.452566746
MMP9	0.110989023	0.452646675
MEF2D	0.110902617	0.453000809
GOLGA7	0.110866436	0.45314914
ARHGAP19	0.110813161	0.453367601
VIM	0.110740723	0.453664734
AHNAK	0.110734898	0.453688634
ARID2	0.110717057	0.453761835
VRK1	0.110708043	0.453798818
CYSLTR2	0.110683051	0.453901376
ZNF595	0.110676617	0.453927778
TMCO3	0.110641851	0.454070469
SAYSD1	0.110639248	0.454081154
RAVER2	0.110620864	0.454156617
ZMAT2	0.110593873	0.454267425
RPS6KA3	0.110481676	0.45472819
ZNF267	0.110444392	0.45488136
SLC20A1	0.110437243	0.454910732
PPIP5K2	0.110404753	0.45504424
CCDC102A	0.110353485	0.455254949
TSEN54	0.110308676	0.455439155
USP48	0.110275085	0.455577271
B2M	0.110189799	0.455928043
IKZF1	0.110188095	0.455935056
RP11-147L13.12	0.110126507	0.456188454
METAP1	0.110060694	0.45645932
RWDD1	0.109931106	0.456992923
BIRC6	0.109882988	0.457191144
IMPA1	0.109862555	0.457275332

RP11-290F24.6	0.109798781	0.457538146
PSMB6	0.109779698	0.457616803
DNAJC8	0.109779622	0.457617117
CDCA4	0.109750001	0.457739226
KIAA1429	0.10965662	0.458124289
MRPS6	0.109645181	0.45817147
ABHD4	0.109603224	0.45834455
COA3	0.109525788	0.458664083
PTPN9	0.109447986	0.458985245
ANKEF1	0.10940449	0.459164847
DENND6B	0.109364247	0.459331049
RNF139	0.109326319	0.459487724
PANX1	0.109322183	0.459504807
C5orf22	0.10931568	0.459531673
ADRB2	0.109310741	0.459552079
SSBP1	0.109279226	0.459682292
RWDD2B	0.109272022	0.459712062
HSPE1	0.109270831	0.459716984
GIMAP1	0.109270047	0.459720222
AC109826-1	0.109262365	0.45975197
QSER1	0.109236172	0.459860216
TRAV12-3	0.109224304	0.459909268
CYP4F3	0.109208376	0.459975106
PRRG4	0.109200238	0.460008744
LRRC37B	0.109116565	0.460354693
WHSC1L1	0.109092331	0.460454919
NUDCD1	0.109059308	0.460591509
TRAT1	0.109051941	0.460621984
CCAR1	0.109046823	0.460643156
NACC2	0.10903139	0.460707002
RP11-47L3.1	0.109004742	0.460817254
USP42	0.108923038	0.461155381
MTFR1	0.108864374	0.461398241
ARHGAP15	0.108853175	0.461444612

NDUFS5	0.108775029	0.461768252
HN1	0.108716823	0.462009388
ADSS	0.108695594	0.462097355
CUL1	0.108639766	0.462328729
UVRAG	0.108627938	0.462377755
NFIA	0.108582494	0.462566152
TDRD3	0.108579715	0.462577671
GTF3C4	0.108554339	0.462682894
TMEM258	0.108517538	0.462835509
CUL3	0.108491504	0.462943487
NRDE2	0.10833086	0.463610087
TRAK2	0.108324852	0.463635029
DUSP2	0.108268154	0.46387043
PAFAH1B2	0.10816387	0.464303568
COQ9	0.108103789	0.464553207
HSPA5	0.108079902	0.46465248
WDR5	0.10807948	0.464654235
STRA13	0.10806737	0.464704567
RP13-104F24.2	0.107971619	0.465102643
ZNF449	0.107856608	0.46558103
EIF4E2	0.107839802	0.465650953
GLE1	0.107838046	0.465658261
ALKBH5	0.107831996	0.465683437
ZFPM1	0.107765487	0.465960232
CTD-2547L24.3	0.107696583	0.466247088
LATS2	0.107647389	0.46645195
KIF11	0.10764117	0.466477852
USP16	0.107527747	0.466950379
RP5-966M1.6	0.107465255	0.467210835
MPHOSPH10	0.107464903	0.467212299
NAA35	0.107462534	0.467222177
CLINT1	0.107401278	0.46747756
IFT80	0.10739596	0.467499734
CMPK1	0.107393172	0.467511359

SNRNP25	0.107357613	0.46765965
SLC35B4	0.107339588	0.467734826
C16orf72	0.107238583	0.468156217
PJA2	0.107214714	0.468255826
RPN2	0.107179989	0.468400762
PDE4A	0.107158691	0.468489664
COX18	0.10710904	0.46869696
CTNNBL1	0.107079166	0.468821711
EHD1	0.107076803	0.46883158
NDUFA1	0.107061034	0.468897436
GPATCH4	0.107037461	0.468995893
SNX18	0.106957609	0.469329496
PHB	0.1069256	0.469463259
TMEM38A	0.106877124	0.469665872
SLC39A9	0.106845947	0.469796205
TYW1B	0.106724477	0.470304192
RCC2	0.10671147	0.470358603
SKP1	0.106668912	0.47053666
CUL4B	0.106658654	0.470579585
CCT5	0.106652014	0.470607371
C12orf66	0.106634183	0.470681989
PLGRKT	0.106606568	0.470797565
ANXA2R	0.106574416	0.470932149
ANAPC16	0.106570751	0.470947492
CRLS1	0.106549569	0.471036168
MRPL35	0.10652312	0.471146909
SNAP47	0.106417754	0.471588207
YTHDC2	0.106413972	0.471604051
DAAM1	0.106408403	0.471627382
FAM175B	0.106381329	0.471740813
PIGS	0.106377903	0.47175517
GTF2H5	0.106374843	0.47176799
MPDU1	0.106358339	0.471837147
MCFD2	0.106306692	0.4720536

DCP1B	0.106278904	0.472170079
NCOA2	0.106223779	0.472401197
COQ5	0.106182143	0.472575799
PLK4	0.106124426	0.472817894
UBE2D1	0.106121221	0.47283134
ME1	0.106111223	0.472873283
HACD3	0.106049687	0.473131485
AP1S2	0.105919459	0.473678166
LAPTM5	0.105869109	0.473889617
FBXO10	0.105858971	0.4739322
STIL	0.105854781	0.4739498
STIM2	0.105782112	0.474255096
LPP	0.105743094	0.474419058
DCAF11	0.105668709	0.474731725
ZNF827	0.105631501	0.474888165
CTC-459F4.3	0.105504947	0.475420467
KPNA2	0.105504774	0.475421195
TAB2	0.105434246	0.475717978
C16orf86	0.105285632	0.476343676
RAB7A	0.105257402	0.476462579
PSAT1	0.105243016	0.476523181
NCBP3	0.105217449	0.476630887
NUDT3	0.105150335	0.476913686
RP11-355O1.11	0.105145154	0.47693552
CLEC11A	0.105131047	0.476994974
NFE4	0.105024498	0.47744416
CD302	0.104956914	0.477729198
EXOC1	0.104951539	0.477751871
MED1	0.104937154	0.47781255
COX7B	0.104935691	0.477818723
GATA3	0.10492844	0.477849312
ASTE1	0.104928435	0.477849332
ZKSCAN5	0.104909512	0.47792917
FKBP4	0.104878211	0.478061241

ALKBH6	0.104869693	0.478097182
APEX2	0.104848271	0.478187588
ANXA2	0.104820842	0.478303351
MTFR2	0.104806163	0.478365314
PSPN	0.104788051	0.478441771
ARG1	0.104772441	0.478507669
BAG2	0.104761486	0.47855392
ARPC3	0.104725004	0.47870796
RBMS1	0.10470968	0.478772669
SMARCA5	0.104636007	0.479083847
IGFBP4	0.104542189	0.479480267
ASXL2	0.104517554	0.479584389
WDR7	0.10451433	0.479598015
LARS	0.104441253	0.479906954
TNPO3	0.104434703	0.47993465
CYB561	0.104426733	0.479968351
CMC2	0.104404165	0.480063787
TANK	0.104366528	0.480222971
SMG1	0.10435764	0.480260565
MYL6B	0.104294182	0.480529028
PSMD6	0.104286164	0.480562954
PVRL2	0.104284148	0.480571486
CD52	0.104232834	0.480788638
PIGB	0.104191663	0.480962909
C1orf123	0.104097437	0.481361872
RP11-101E13.5	0.104091593	0.481386622
IRF2BP2	0.104055421	0.48153983
INPP1	0.103920438	0.482111781
SETD4	0.103852024	0.482401802
MCEE	0.103833275	0.482481295
PGP	0.10383314	0.48248187
RAB35	0.103817668	0.482547477
ERCC8	0.103809168	0.482583525
SEC11C	0.10379287	0.482652639

WDR55	0.103781882	0.482699241
CCNB1	0.103773539	0.482734626
PSMA2	0.103756726	0.482805941
ARSB	0.103751531	0.482827975
MED30	0.103749323	0.482837343
C14orf2	0.103681383	0.483125578
MARVELD1	0.103679353	0.483134193
OSGIN2	0.103578022	0.483564264
ISY1	0.103551456	0.483677051
TEX2	0.103442112	0.484141415
MFF	0.103414748	0.484257661
LMAN1	0.103354827	0.484512265
MAGED2	0.103354398	0.484514089
CAND1	0.103327316	0.484629186
BBS12	0.10321824	0.485092888
RAN	0.103197154	0.485182555
PDIK1L	0.103191838	0.485205164
TTF2	0.103125744	0.485486288
PTGR2	0.10308271	0.485669376
FAM120AOS	0.103046095	0.485825182
PSMD1	0.1030014	0.486015406
PDCL	0.102970095	0.486148665
ZBTB14	0.102961296	0.486186121
CPSF2	0.102940511	0.486274614
GTF2A2	0.102923326	0.486347785
TADA1	0.102919126	0.486365667
BCL7C	0.102914086	0.486387131
ASF1B	0.102879748	0.48653336
GABARAPL1	0.102875362	0.486552038
RP11-196G11.5	0.102868601	0.486580835
UBALD2	0.102795247	0.486893307
FBXO38	0.102763563	0.487028307
TOP1	0.102691491	0.487335465
BTN3A1	0.102684134	0.487366825

CAB39L	0.102671368	0.487421242
CCDC107	0.102657693	0.48747954
SEC24D	0.10264105	0.487550495
CCDC109B	0.102587926	0.487777015
EZR	0.102526135	0.488040563
SCAPER	0.102492753	0.488182974
PI4KAP2	0.102491565	0.48818804
PYM1	0.102487101	0.488207088
H2AFZ	0.10240064	0.488576043
EZH2	0.102389875	0.488621992
CTA-963H5.5	0.102347027	0.488804905
PRMT3	0.102333991	0.48886056
CD96	0.102305282	0.488983139
C10orf76	0.102294348	0.489029829
ORC5	0.102242719	0.489250322
HEG1	0.102216582	0.489361965
PDCD2L	0.102173048	0.489547954
PTPN11	0.102156725	0.489617695
DTL	0.102138049	0.489697501
DNAJC7	0.102082607	0.48993445
GCNT1	0.102068084	0.489996528
C10orf88	0.102055101	0.490052026
LINS1	0.102012562	0.490233894
TRBV11-2	0.10195443	0.490482482
AP3S1	0.10194941	0.490503951
DCUN1D4	0.101915403	0.490649411
NOLC1	0.101908863	0.490677387
OGFOD1	0.10190092	0.490711366
R3HCC1L	0.101863807	0.490870144
FAM134C	0.101822938	0.491045022
MSRB2	0.101804793	0.491122674
SUSD1	0.101735692	0.491418454
KRR1	0.101719096	0.491489506
ATG2A	0.101701598	0.491564423

RP11-568K15.1	0.101655362	0.491762417
BAZ1B	0.101600983	0.491995328
UHRF1BP1L	0.101595918	0.492017027
UBE3C	0.101467656	0.492566633
IGF2R	0.101457466	0.492610311
RP11-800A3.4	0.101451777	0.492634696
EXOSC5	0.101359773	0.493029158
IKZF5	0.101208914	0.493676315
NEDD8	0.101124427	0.494038942
HINT3	0.101085449	0.494206285
NXT2	0.101056652	0.494329937
EPHA2	0.101028167	0.494452265
ZNF48	0.10102403	0.494470034
ZNF28	0.101010488	0.494528196
NQO2	0.101010259	0.49452918
MCL1	0.101009963	0.494530449
MIEN1	0.100982895	0.494646715
LASP1	0.100938821	0.494836063
INCENP	0.10092243	0.494906486
POLR2C	0.100885714	0.495064263
RBM12B	0.100877704	0.495098682
AC096772-6	0.100812532	0.49537881
CPD	0.10079291	0.495463168
OAT	0.100709063	0.495823718
LEMD3	0.1006309	0.496159949
ANXA7	0.100598068	0.496301217
MSH2	0.100582713	0.496367291
ZNF688	0.10056738	0.496433276
CCDC88A	0.100566418	0.496437416
WRN	0.10056414	0.49644722
AGTPBP1	0.100536286	0.496567103
NUP50	0.100458077	0.496903793
CCDC186	0.10043608	0.49699851
CSDE1	0.100352796	0.497357211

MRPL21	0.100316282	0.497514518
WWP1	0.10030879	0.497546794
PBDC1	0.100275168	0.49769167
SLFN5	0.10027202	0.497705236
ADAM10	0.100207186	0.497984665
ALG9	0.100119852	0.498361197
CSGALNACT2	0.10011837	0.498367589
C6orf1	0.100092737	0.498478131
TAF15	0.100085197	0.498510647
TEFM	0.100063794	0.498602961
MSH6	0.100047482	0.498673325
CCDC127	0.100038972	0.498710033
MTRR	0.100000944	0.498874092
IDS	0.099875823	0.499414076
TCP11L1	0.099833727	0.499595817
APAF1	0.099700268	0.500172226
CELF2	0.099698353	0.500180501
NCKAP1L	0.099620008	0.500519034
F8	0.099603445	0.500590615
SLC39A4	0.099602947	0.500592767
ZFP1	0.09954804	0.500830113
HAVCR2	0.099469916	0.501167918
BUB3	0.099429253	0.501343784
ATP5F1	0.099409589	0.501428844
NIN	0.099362924	0.501630732
PINK1-AS	0.099358693	0.501649038
SPOPL	0.099349913	0.501687029
PCM1	0.099338866	0.501734827
ZNF277	0.099309288	0.501862827
MAP3K5	0.099305347	0.501879881
BIN2	0.099294592	0.501926428
TAF9	0.099247184	0.502131635
M6PR	0.099245071	0.502140779
TMEM168	0.099167611	0.50247616

TMSB10P1	0.099100909	0.502765054
UBA6	0.0990509	0.502981701
GARS	0.099017413	0.5031268
SRBD1	0.098987633	0.503255856
LRBA	0.098977823	0.503298371
PDE7B	0.098968082	0.50334059
SLC9B2	0.098930668	0.503502766
CDK1	0.098925178	0.503526565
PEX7	0.09886767	0.503775896
XRN1	0.098830787	0.503935841
MCM2	0.098788576	0.504118921
MTHFD2	0.098763171	0.504229125
LCLAT1	0.098760776	0.504239516
SIAH2	0.098736087	0.504346628
ADAT1	0.098685891	0.504564436
RP11-1094M14.8	0.098646572	0.504735083
ZXDA	0.0985997	0.504938545
RACGAP1	0.098592398	0.504970243
ZFP82	0.09858914	0.504984389
FCGR2B	0.098588457	0.504987353
CCDC167	0.098565862	0.505085454
SESTD1	0.098536289	0.505213866
RAB12	0.098392814	0.505837097
HSD17B12	0.098359215	0.505983104
RPRD2	0.098349561	0.506025058
C14orf169	0.098277955	0.506336308
GPR18	0.09826129	0.506408759
CCNA2	0.098246407	0.506473472
FBXO5	0.098193455	0.506703733
WASL	0.098175313	0.506782635
PRR5	0.098119933	0.507023527
CRIPT	0.098093108	0.507140233
TMEM256	0.097950595	0.507760486
RECQL	0.097924033	0.507876134

IGHG3	0.097914891	0.507915936
NMD3	0.097815121	0.508350457
RTFDC1	0.097814163	0.508354632
IGLV6-57	0.097756185	0.508607225
EID2B	0.097724775	0.508744097
TM2D3	0.097694503	0.508876026
GSTP1	0.097684931	0.508917745
TAGLN2P1	0.097676139	0.508956067
RAD50	0.097634473	0.509137696
LRIG1	0.097592922	0.509318856
DNAAF2	0.097591985	0.509322945
GPBP1L1	0.097574223	0.509400397
POLB	0.097568688	0.509424532
PDIA6	0.097555568	0.509481749
SDF2L1	0.097541867	0.5095415
TRAV8-2	0.097532779	0.509581139
ZYG11B	0.097532362	0.509582957
C20orf197	0.097490979	0.509763466
ATMIN	0.097474587	0.509834973
ITM2A	0.097442469	0.509975099
ORC3	0.097404283	0.510141727
ARMCX3	0.097387731	0.510213961
GPD2	0.097373734	0.510275048
YIPF6	0.097329693	0.510467285
VMA21	0.097315364	0.51052984
IRF2BPL	0.097251229	0.510809864
ATP5G3	0.097246875	0.510828876
JUND	0.09722823	0.5109103
FLNA	0.097072664	0.511589927
MAGEH1	0.097027934	0.51178542
MSANTD2	0.096992996	0.511938147
ADAM22	0.09698896	0.511955795
IFNLR1	0.096858326	0.512527053
UTP11L	0.096838155	0.512615291

ETFA	0.09683801	0.512615925
SLC39A6	0.096808898	0.512743284
TXNIP	0.096786525	0.512841176
LAS1L	0.096781835	0.512861694
IGLV2-14	0.096771745	0.512905848
STT3B	0.096728573	0.513094781
ZNF37A	0.096706544	0.513191198
NUP54	0.096648249	0.513446392
DIRC2	0.096578904	0.51375004
LSM2	0.096561446	0.5138265
ING2	0.096545435	0.513896628
EXT2	0.096483786	0.514166689
CHAF1A	0.096477423	0.514194569
MAPKAP1	0.096475599	0.514202562
PLEKHF2	0.096468725	0.514232677
EPRS	0.096466545	0.514242232
POLE3	0.096442	0.514349781
NCL	0.096429406	0.514404969
C22orf39	0.096414218	0.51447153
COQ10B	0.096299201	0.514975724
HNRNPR	0.096287432	0.515027329
SPAG1	0.096273415	0.515088795
SMYD5	0.096222036	0.515314124
AGAP1	0.096205101	0.515388407
MED18	0.096188575	0.515460902
SLC25A24	0.096171468	0.515535951
ALS2	0.096169275	0.51554557
LAMTOR2	0.096141768	0.515666257
PSMA7	0.096089027	0.515897694
EPC2	0.096032229	0.516146991
SCAMP1	0.096026855	0.516170578
FAM58A	0.096024177	0.516182335
UBE2I	0.09598199	0.516367548
RAP1GAP2	0.095951129	0.516503057

FAM107B	0.095932146	0.51658642
EXTL2	0.09591439	0.516664401
USP4	0.095882361	0.51680508
ZNF343	0.095844298	0.516972285
GMFG	0.095833831	0.517018268
DNAL4	0.095811514	0.517116322
11-Sep	0.095809966	0.517123125
CDC7	0.095752181	0.517377055
NAT1	0.095724704	0.517497823
6-Mar	0.095700124	0.51760587
ARHGDIB	0.095695488	0.517626247
RTTN	0.095673951	0.517720928
MSN	0.095650867	0.517822423
NOC3L	0.09564329	0.517855736
GAPDH	0.095589506	0.518092251
SIK2	0.095574515	0.518158186
ZKSCAN1	0.095563612	0.518206141
CHIC2	0.095538295	0.518317503
PGAP1	0.095455037	0.518683814
USP33	0.095449402	0.518708614
ZNF654	0.095438768	0.518755408
LACTB	0.09539972	0.518927268
RMND1	0.095381903	0.519005693
NDUFA6	0.095368211	0.519065962
EIF2B2	0.095333765	0.51921761
NAA25	0.095224662	0.519698076
STIM1	0.095181208	0.519889499
PSME3	0.095174057	0.519921005
SF3B6	0.095145681	0.520046031
RBM42	0.095138948	0.520075698
ZNF484	0.09513058	0.520112572
AFF4	0.095095054	0.520269131
DLG5	0.095065262	0.520400436
UTRN	0.094978418	0.520783295

ZNF213	0.094961247	0.520859014
DYRK1B	0.094956887	0.520878238
FUBP3	0.094898766	0.52113457
XPO1	0.094817789	0.521491809
TARS	0.094816293	0.521498413
SUV39H2	0.094809823	0.521526961
ZNF852	0.094802724	0.521558285
COX15	0.094768778	0.521708089
GOT1	0.09476541	0.52172295
MRPS30	0.094748177	0.521799011
EBNA1BP2	0.094695809	0.522030168
BRD3	0.094654393	0.522213023
KIF15	0.09464593	0.522250388
TAF8	0.094639997	0.522276586
E2F2	0.094626496	0.522336206
WIPI1	0.094589374	0.522500147
HIST1H1C	0.094516204	0.522823361
TCF4	0.094428274	0.5232119
RHOBTB3	0.094425465	0.523224318
ZNF880	0.094399316	0.523339893
HLA-DPB1	0.094380495	0.523423088
GNPAT	0.094348575	0.5235642
ALDH18A1	0.094262569	0.523944507
MBP	0.094258588	0.523962113
RNASEL	0.094238396	0.524051417
HMGB3	0.094206156	0.524194032
JAK2	0.094198512	0.524227844
ZNF776	0.094192867	0.524252818
CD53	0.094137909	0.524495978
YIPF1	0.094103168	0.524649717
MORF4L2	0.094048887	0.524889971
PLBD1	0.094038725	0.524934953
CTU1	0.094014782	0.525040951
TFRC	0.093966556	0.525254479

SUPT4H1	0.093885203	0.525614786
MLEC	0.093863197	0.525712265
DNAJC16	0.093856669	0.525741186
SOD1	0.09382553	0.525879145
LSM5	0.093823319	0.525888943
SLC1A5	0.093789899	0.526037032
BMP8B	0.093786006	0.52605428
CCDC58	0.093640587	0.526698896
SUCLG2	0.093579392	0.526970279
IFT52	0.093567217	0.527024278
RIT1	0.093489599	0.527368611
VPS26B	0.093474987	0.527433444
NRF1	0.093452176	0.527534667
MALSU1	0.093443085	0.527575009
ACSL4	0.093439478	0.527591018
GNPNAT1	0.093365558	0.527919112
PIGU	0.09335883	0.527948982
MEGF9	0.093356505	0.527959301
DYNC2H1	0.093354003	0.527970409
MRPS12	0.093338851	0.528037677
DNM1L	0.093336179	0.528049539
ECI2	0.093286725	0.528269128
RPRD1A	0.093269733	0.528344587
ENOX2	0.093237838	0.52848624
ACAP2	0.093113769	0.529037439
CSPP1	0.093072671	0.529220089
CSE1L	0.093061582	0.529269379
IL10RA	0.093058239	0.529284236
LSM14A	0.093015965	0.529472153
NUDT21	0.09300831	0.529506187
VWA5A	0.092964611	0.529700478
TFCP2	0.092961579	0.529713963
AGK	0.092694823	0.53090078
DYRK2	0.092680701	0.530963646

TET1	0.092662052	0.531046674
CTSO	0.092641594	0.531137758
DPYSL2	0.092603875	0.531305717
AOC3	0.092587444	0.531378886
RSF1	0.092489921	0.531813292
APPBP2	0.092486851	0.531826971
OSBPL9	0.092480007	0.531857465
ZNF225	0.092478132	0.531865818
IK	0.092461966	0.531937848
RAB5A	0.092431207	0.53207491
PORCN	0.092407514	0.5321805
CCT6P1	0.092407339	0.532181282
SPATA5	0.092401743	0.532206221
TRBJ1-6	0.09238998	0.532258647
REPS2	0.092330726	0.532522781
FAM96A	0.092314567	0.532594825
CENPE	0.092138178	0.533381537
GSAP	0.092125349	0.533438773
OFD1	0.092113577	0.533491304
CLIP1	0.092102777	0.533539493
NAP1L4	0.092089365	0.533599347
CIR1	0.092068413	0.533692851
PROSC	0.091999838	0.53399894
EMP3	0.091970878	0.53412823
NDUFA5	0.091940703	0.534262963
AMOT	0.091913671	0.534383678
ARL13B	0.091834599	0.534736854
TRIM23	0.091832463	0.534746394
NME7	0.091801373	0.534885291
FAM195B	0.091786687	0.534950909
STXBP3	0.091769083	0.535029567
MTHFD1	0.091744816	0.535138008
NFS1	0.091513908	0.536170389
ASB8	0.091463769	0.536394687

BTBD10	0.091436426	0.536517028
TMEM109	0.09138673	0.536739413
FAM120A	0.091381329	0.536763584
NAPG	0.091363333	0.536844128
MSANTD3	0.091356928	0.536872797
FAM174A	0.091322184	0.537028317
RIOK3	0.09130565	0.537102335
CASP8AP2	0.091299982	0.53712771
SLC39A1	0.091295008	0.537149979
MAPK6	0.091292681	0.537160397
TAF3	0.091248943	0.537356235
DDX49	0.091236853	0.53741037
H2AFX	0.091222434	0.537474941
PRKDC	0.091206463	0.537546469
SYNRG	0.091194293	0.537600974
YEATS4	0.091142079	0.537834858
ZNF160	0.091107965	0.537987696
FKBP2	0.091053995	0.53822953
EIF1	0.090969301	0.538609141
CDC5L	0.09096161	0.538643622
FCF1P2	0.090939861	0.538741127
PPIAP22	0.090874257	0.539035299
RPL17	0.090861001	0.539094749
MCM9	0.090829626	0.539235471
HSD17B11	0.090798307	0.539375963
CCNB2	0.090765626	0.539522577
SDHAF1	0.090726414	0.539698522
C14orf119	0.090721768	0.539719369
SPCS2	0.090666613	0.5399669
TMEM91	0.090622784	0.540163639
FIG4	0.090567746	0.540410745
MAP3K9	0.090545285	0.540511604
SERAC1	0.090533034	0.54056662
UBA5	0.090477825	0.54081458

UBTD2	0.090473277	0.540835008
RBX1	0.090454286	0.540920319
RPP14	0.090357122	0.541356886
OCIAD1	0.09034444	0.54141388
FEZ2	0.090313245	0.541554089
RP11-77H9.2	0.09030773	0.541578875
MOCS3	0.090298871	0.541618698
GSPT1	0.090296004	0.541631584
ABHD17B	0.090268926	0.541753309
MPP6	0.09024057	0.541880794
EIF3J	0.09023673	0.541898059
C19orf53	0.090149119	0.542292039
CISD2	0.090140055	0.54233281
PLEKHM3	0.089977994	0.543061978
SNHG8	0.089901241	0.543407482
AC062029-1	0.089882068	0.543493805
RALY	0.089864733	0.54357186
GNPTAB	0.089758509	0.544050269
CREBRF	0.089751492	0.544081881
SSR3	0.089734333	0.544159181
MRPS34	0.089734093	0.544160264
SPTSSA	0.08972221	0.544213801
OXCT1	0.089702674	0.54430182
AASDH	0.089702617	0.544302079
MTCH2	0.089636452	0.54460024
TMEM59	0.089600491	0.544762328
PIGF	0.089595486	0.544784886
CFL1	0.089570034	0.54489962
BRCC3	0.089553823	0.544972705
TTLL5	0.089490778	0.545256974
FOXK2	0.089411298	0.54561545
PPP1CC	0.089402935	0.545653173
ZNF749	0.089353883	0.545874476
NUSAP1	0.089347384	0.545903801

DHX15	0.089318412	0.546034532
CIPC	0.089290401	0.546160942
SKA2	0.08927504	0.546230269
KHDRBS1	0.089188176	0.546622391
RP11-104N10.2	0.089170354	0.54670286
TRMT13	0.089103673	0.547003981
KIF27	0.089070142	0.547155433
NPTN	0.089048962	0.547251108
SAP30L	0.089028424	0.547343892
HNRNPC	0.089025112	0.547358853
PITPNB	0.088915445	0.547854421
SFT2D1	0.088912444	0.547867985
CSRP2BP	0.088865235	0.548081385
TLK1	0.088860741	0.5481017
ASUN	0.088830602	0.548237962
ZNF770	0.088799836	0.548377075
INO80C	0.08877081	0.548508336
IDH3A	0.088710513	0.548781051
CALHM2	0.088699021	0.54883304
DNAJA4	0.088672581	0.548952651
PTER	0.088621936	0.549181797
APOL3	0.088606505	0.549251627
NFATC1	0.088540637	0.54954974
PPIB	0.088354813	0.550391176
WDR36	0.088347272	0.550425335
ETFB	0.088287585	0.550695748
CHST10	0.088220386	0.551000265
TNFRSF12A	0.088211838	0.551039005
GIMAP7	0.088199827	0.551093445
BCL10	0.088197405	0.551104421
KIAA0753	0.088164037	0.551255676
P2RY1	0.088108509	0.551507422
FNDC3A	0.088022089	0.551899331
RBMX2	0.087973115	0.552121481

GOPC	0.087972448	0.552124509
WDR1	0.087928421	0.552324258
ANKRD54	0.087920923	0.55235828
MRPL14	0.0878361	0.552743223
C1D	0.087786014	0.552970587
CTC-479C5.12	0.087758175	0.553096977
DARS	0.087751006	0.553129529
FBXW7	0.087744513	0.55315901
GMPS	0.087736208	0.553196721
ZNF304	0.087726805	0.553239417
CLTA	0.087684082	0.553433434
CEPT1	0.087682893	0.553438836
HGF	0.087626159	0.553696531
CD247	0.087608891	0.553774977
TWF2	0.087581252	0.553900549
CCNI	0.087560699	0.553993934
WAC-AS1	0.08754703	0.554056048
ZNF655	0.087542357	0.554077281
FGL2	0.087511667	0.554216751
TTC37	0.087494391	0.554295266
CHMP4B	0.087470925	0.554401925
IGLV3-10	0.087456583	0.55446712
TMCC3	0.087433077	0.554573973
TRAV29DV5	0.087381969	0.554806337
YEATS2	0.087370348	0.554859182
MPP7	0.087349177	0.554955453
KBTBD3	0.087285359	0.555245704
UBE2E1	0.087203897	0.555616309
BAG4	0.087178941	0.555729868
ACAT2	0.087168912	0.555775509
HDAC1	0.087093322	0.556119551
DYNLRB1	0.08706712	0.556238827
MAD2L1	0.087045635	0.556336645
PSME1	0.08699091	0.556585827

RP1-47M23.3	0.086984273	0.556616052
RRBP1	0.086901662	0.556992322
DYM	0.086884955	0.557068434
CIT	0.086884039	0.557072607
ABHD12	0.086843041	0.557259399
ZNF271P	0.086820167	0.557363628
UBXN8	0.086792017	0.557491911
RASSF1	0.086775183	0.557568628
SLC19A2	0.086767624	0.557603083
ALDH9A1	0.086727325	0.557786771
ZNF529	0.086642231	0.558174734
SASS6	0.086640886	0.558180868
C12orf45	0.086590301	0.558411561
TAF2	0.086537552	0.558652166
DAD1	0.08653304	0.558672749
TMEM135	0.086466309	0.558977209
CREBL2	0.086400491	0.559277579
MPC1	0.08637905	0.559375446
C6orf106	0.086350209	0.559507098
HSPBAP1	0.086328254	0.559607329
NUDC	0.086321665	0.559637413
ARCN1	0.08627606	0.559845644
UBL5	0.08626965	0.559874916
CHM	0.086253964	0.55994655
TMEM41A	0.086204777	0.560171197
ISOC2	0.086175995	0.560302667
RP11-488L18.10	0.086063892	0.560814882
HMOX2	0.086033634	0.560953172
GRAMD4	0.086002494	0.561095508
DHX36	0.085980294	0.561196991
TRIP12	0.085927734	0.561437297
MRPL40	0.085911845	0.561509953
PSENEN	0.08590392	0.561546194
BLMH	0.085897326	0.561576344

STAG3L4	0.085888941	0.56161469
TNIK	0.085876141	0.561673228
SLTM	0.085827354	0.561896371
SLC25A46	0.08578151	0.562106091
SLC25A11	0.085750362	0.562248602
ZNF799	0.085743277	0.562281021
LONRF1	0.085725169	0.562363879
RPS24	0.08569576	0.56249846
CHMP2A	0.085614575	0.562870064
PARP4	0.08561045	0.562888946
DENND4C	0.08558368	0.563011506
NPHP4	0.085560559	0.563117369
TAF12	0.085556099	0.563137791
C20orf27	0.085539265	0.563214874
HERC2P2	0.085512652	0.563336746
ANP32B	0.085471634	0.563524613
METTL5	0.085428974	0.563720027
FUT8	0.085413004	0.563793192
AP1G1	0.085371537	0.563983185
STT3A	0.085363589	0.5640196
PAN3	0.085287366	0.564368928
LINC00963	0.085280217	0.564401694
AKAP11	0.08526441	0.564474152
AC093627-10	0.085252256	0.564529868
NUCKS1	0.085146538	0.565014598
UST	0.085053117	0.565443103
NUDT16	0.085004999	0.565663871
COPS5	0.084983318	0.565763359
AC006978-6	0.084911871	0.566091262
PPIA	0.084911393	0.566093456
RORA	0.084896147	0.566163442
KIF3C	0.084886518	0.566207644
PRICKLE3	0.084846361	0.566391996
LINC00152	0.08484272	0.566408713
