# Supplementary material for: Neutrophil-enriched gene signature correlates with teplizumab therapy resistance in different stages of type 1 diabetes
Source: J Clin Invest. 2025 Sep 30;135(23):e176403. doi: 10.1172/JCI176403 (PMC12646666; doi:10.1172/JCI176403)
Supplement: Supplemental table 5 [file jci-135-176403-s293.pdf]

Supplemental Table 5: Marker genes of each cell type in human whole blood single cell RNA-sequencing d

p_val	avg_log2FC	pct.1	pct.2	p_val_adj	cluster	gene
0	3.89458344	0.936	0.102	0	T cells	TCF7
0	3.80571473	0.672	0.042	0	T cells	CCR7
0	3.77007927	0.548	0.019	0	T cells	MAL
0	3.76765608	0.942	0.082	0	T cells	TRAC
0	3.76028914	0.902	0.122	0	T cells	LDHB
0	3.66600525	0.353	0.011	0	T cells	LRRN3
0	3.59913537	0.793	0.072	0	T cells	RCAN3
0	3.58465541	0.894	0.13	0	T cells	TRBC2
0	3.56473824	0.936	0.132	0	T cells	IL7R
0	3.52302977	0.641	0.078	0	T cells	TRBC1
0	3.41496283	0.461	0.023	0	T cells	AQP3
0	3.40264762	0.93	0.092	0	T cells	CD3E
0	3.28388566	0.505	0.027	0	T cells	RGCC
0	3.25753299	0.786	0.061	0	T cells	CD3G
0	3.1111618	0.999	0.308	0	T cells	RPS12
0	3.0340265	0.619	0.049	0	T cells	CD3D
0	3.007374	0.882	0.12	0	T cells	SPOCK2
0	2.9932724	0.998	0.3	0	T cells	RPL13
0	2.97462547	0.805	0.071	0	T cells	LEF1
0	2.96962652	1	0.478	0	T cells	RPS6
0	2.96469878	0.944	0.189	0	T cells	GIMAP7
0	2.96313847	0.997	0.295	0	T cells	RPL3
0	2.96068127	0.962	0.233	0	T cells	NPM1
0	2.95131805	0.998	0.34	0	T cells	RPS18
0	2.94588384	0.983	0.255	0	T cells	RPS5
0	2.94564913	0.637	0.075	0	T cells	PIK3IP1
0	2.93165103	0.992	0.26	0	T cells	RPL4
0	2.92558207	0.503	0.038	0	T cells	FAM102A
0	2.9069984	0.761	0.101	0	T cells	LBH
0	2.90566871	0.308	0.015	0	T cells	CD40LG
0	2.90340526	0.992	0.261	0	T cells	RPL10A
0	2.90282294	0.427	0.036	0	T cells	TBC1D4
0	2.90240895	0.998	0.349	0	T cells	RPLP2
0	2.90134396	1	0.43	0	T cells	RPL32
0	2.90101446	0.999	0.379	0	T cells	RPS20
0	2.90043607	0.48	0.039	0	T cells	TRABD2A
0	2.89464098	0.943	0.21	0	T cells	RPSA
0	2.87656819	0.998	0.296	0	T cells	RPL5
0	2.87360024	0.996	0.273	0	T cells	RPL22
0	2.85971607	0.419	0.031	0	T cells	LDLRAP1
0	2.85871165	0.994	0.297	0	T cells	RPL36
0	2.85462363	0.995	0.275	0	T cells	RPS4X
0	2.8423428	0.999	0.432	0	T cells	RPS27
0	2.83939157	0.661	0.097	0	T cells	GIMAP5
0	2.82104771	1	0.532	0	T cells	RPS29
0	2.81527179	0.963	0.249	0	T cells	EEF1B2
0	2.80851348	0.947	0.189	0	T cells	ETS1
0	2.80698699	1	0.378	0	T cells	RPS8

0	2.80112153	0.999	0.439	0	T cells	RPL9
0	2.78335738	0.991	0.304	0	T cells	RPS21
0	2.77848268	0.832	0.125	0	T cells	IL32
0	2.77609664	0.998	0.388	0	T cells	RPS3A
0	2.77528486	0.497	0.047	0	T cells	PRKCQ-AS1
0	2.76216521	0.999	0.417	0	T cells	RPL31
0	2.7563919	0.995	0.305	0	T cells	RPS23
0	2.75609616	0.359	0.023	0	T cells	CD5
0	2.75522343	0.999	0.4	0	T cells	RPL13A
0	2.73605067	0.923	0.225	0	T cells	EEF1G
0	2.72874586	0.825	0.168	0	T cells	SNHG29
0	2.72544545	0.828	0.146	0	T cells	SLFN5
0	2.71988822	0.344	0.026	0	T cells	LINC01550
0	2.71638004	0.997	0.355	0	T cells	RPL14
0	2.71583346	0.994	0.295	0	T cells	RPL36A
0	2.7153408	0.996	0.321	0	T cells	RPS17
0	2.71450305	0.701	0.128	0	T cells	GAS5
0	2.7059238	0.997	0.396	0	T cells	RPS25
0	2.70151393	0.995	0.331	0	T cells	RPL12
0	2.70141668	1	0.511	0	T cells	RPL21
0	2.7002284	0.335	0.033	0	T cells	MYC
0	2.69837981	0.997	0.413	0	T cells	RPS15A
0	2.69774675	0.958	0.235	0	T cells	RPL18A
0	2.69772919	1	0.468	0	T cells	RPS27A
0	2.69622515	0.992	0.288	0	T cells	RPL23A
0	2.68956234	0.551	0.056	0	T cells	TESPA1
0	2.68646315	0.999	0.398	0	T cells	RPS13
0	2.6797786	0.669	0.129	0	T cells	SNHG32
0	2.67798345	0.997	0.382	0	T cells	RPS16
0	2.67295088	1	0.561	0	T cells	EEF1A1
0	2.66527207	0.901	0.2	0	T cells	RPLP0
0	2.66223453	0.411	0.042	0	T cells	ITM2A
0	2.66133366	0.699	0.134	0	T cells	NOP53
0	2.65857878	0.992	0.324	0	T cells	RPL19
0	2.65775029	0.426	0.057	0	T cells	GPR183
0	2.65434481	0.767	0.089	0	T cells	CD2
0	2.65221612	0.992	0.349	0	T cells	RPS14
0	2.64508107	0.995	0.324	0	T cells	RPL10
0	2.64398502	0.998	0.388	0	T cells	RPL11
0	2.64273425	0.999	0.387	0	T cells	RPS28
0	2.64173605	0.982	0.316	0	T cells	RPS3
0	2.63546693	1	0.618	0	T cells	RPL34
0	2.62190071	0.576	0.072	0	T cells	OXNAD1
0	2.62044588	0.334	0.022	0	T cells	SIRPG
0	2.61788754	0.993	0.332	0	T cells	RPL35A
0	2.61690446	0.457	0.06	0	T cells	TRIB2
0	2.61198225	0.915	0.217	0	T cells	RPL35
0	2.61053484	0.687	0.093	0	T cells	ABLIM1
0	2.60267001	0.985	0.293	0	T cells	RPL7
0	2.59364602	0.358	0.036	0	T cells	C12orf57

0	2.59192876	0.992	0.321	0	T cells	RPS2
0	2.58627244	0.977	0.271	0	T cells	RPS19
0	2.57426322	0.996	0.421	0	T cells	RPL38
0	2.57411538	0.999	0.367	0	T cells	RPL37A
0	2.57145358	0.874	0.208	0	T cells	RPL18
0	2.57112978	0.393	0.034	0	T cells	TRAT1
0	2.55304696	0.322	0.023	0	T cells	CHRM3-AS2
0	2.53705775	0.458	0.059	0	T cells	AL365361.1
0	2.53130324	0.518	0.061	0	T cells	LAT
0	2.51968251	0.781	0.106	0	T cells	BCL11B
0	2.50730469	0.714	0.109	0	T cells	LINC00861
0	2.50426575	0.363	0.027	0	T cells	CD28
0	2.49970667	0.65	0.108	0	T cells	PCED1B-AS1
0	2.4953167	0.999	0.517	0	T cells	RPL39
0	2.49496381	0.991	0.336	0	T cells	RPL27
0	2.47946905	0.998	0.381	0	T cells	RPL6
0	2.47785171	0.999	0.454	0	T cells	RPL17
0	2.47338263	0.993	0.373	0	T cells	RPLP1
0	2.47214402	0.57	0.104	0	T cells	PEBP1
0	2.45864581	0.393	0.063	0	T cells	SNHG8
0	2.44405971	0.587	0.119	0	T cells	IL6ST
0	2.44102648	1	0.596	0	T cells	RPL30
0	2.43829119	0.925	0.252	0	T cells	RPL24
0	2.4306711	0.988	0.321	0	T cells	RPL29
0	2.42995771	0.639	0.114	0	T cells	BCL9L
0	2.42460065	0.999	0.474	0	T cells	RPL27A
0	2.401463	0.879	0.203	0	T cells	TLE5
0	2.40067026	0.982	0.328	0	T cells	RPS7
0	2.39951518	0.295	0.028	0	T cells	AC243960.1
0	2.39872732	0.998	0.379	0	T cells	RPL26
0	2.39279365	0.797	0.182	0	T cells	EIF3E
0	2.38440945	0.42	0.069	0	T cells	SNHG25
0	2.38046889	0.998	0.441	0	T cells	RPL23
0	2.37784844	0.271	0.021	0	T cells	CD27
0	2.37780138	0.934	0.278	0	T cells	TOMM7
0	2.36958844	0.999	0.574	0	T cells	RPL37
0	2.36503545	0.417	0.057	0	T cells	LIME1
0	2.35226476	0.284	0.031	0	T cells	HSPB1
0	2.34893281	0.494	0.059	0	T cells	ITGA6
0	2.34555965	0.92	0.258	0	T cells	RPS15
0	2.34239452	0.366	0.041	0	T cells	THEM4
0	2.33342745	0.495	0.104	0	T cells	GIMAP1
0	2.32047884	0.79	0.197	0	T cells	HINT1
0	2.31992893	0.923	0.256	0	T cells	RPL7A
0	2.31300059	0.951	0.267	0	T cells	RACK1
0	2.31233884	0.876	0.215	0	T cells	HSP90AB1
0	2.31087532	0.634	0.093	0	T cells	RASGRP1
0	2.30147669	0.878	0.225	0	T cells	HNRNPA1
0	2.3004155	0.762	0.143	0	T cells	FCMR
0	2.29221168	0.506	0.089	0	T cells	DYRK2

0	2.28812127	0.331	0.035	0	T cells	FLT3LG
0	2.28671897	0.533	0.08	0	T cells	S1PR1
0	2.27488776	0.937	0.259	0	T cells	RPL8
0	2.26556321	0.602	0.134	0	T cells	SNHG6
0	2.24656436	0.671	0.164	0	T cells	SATB1
0	2.23799964	0.76	0.191	0	T cells	SNHG5
0	2.2324682	0.987	0.304	0	T cells	NAP1L1
0	2.23174694	0.978	0.297	0	T cells	RPL15
0	2.22669456	0.587	0.129	0	T cells	SOD1
0	2.21528599	0.644	0.088	0	T cells	ITK
0	2.20898987	0.786	0.147	0	T cells	SLC38A1
0	2.20802585	0.687	0.141	0	T cells	DGKA
0	2.20773819	0.411	0.082	0	T cells	FBL
0	2.19291722	0.577	0.081	0	T cells	LCK
0	2.19093828	0.999	0.587	0	T cells	RPL41
0	2.19050986	0.638	0.153	0	T cells	RSL1D1
0	2.18888269	0.686	0.172	0	T cells	NSA2
0	2.17906453	0.296	0.038	0	T cells	GATA3
0	2.17000867	0.618	0.147	0	T cells	SNRPD2
0	2.13527855	0.779	0.203	0	T cells	COX7C
0	2.13525051	0.96	0.363	0	T cells	RPL28
0	2.13164941	0.382	0.053	0	T cells	CD7
0	2.13159337	0.634	0.157	0	T cells	RPS4Y1
0	2.12818074	0.35	0.069	0	T cells	PPA1
0	2.12400627	0.587	0.066	0	T cells	CAMK4
0	2.1167116	0.39	0.063	0	T cells	SNHG14
0	2.11601131	0.874	0.236	0	T cells	EEF2
0	2.11320544	0.376	0.051	0	T cells	LY9
0	2.11197437	0.746	0.322	0	T cells	RPS26
0	2.11070487	0.34	0.062	0	T cells	SRSF8
0	2.10332109	0.326	0.039	0	T cells	ATP8B2
0	2.07618005	0.689	0.173	0	T cells	COX6C
0	2.06363518	1	0.876	0	T cells	TPT1
0	2.05327238	0.322	0.064	0	T cells	CBX5
0	2.05190483	0.288	0.043	0	T cells	CDC25B
0	2.05181558	0.632	0.106	0	T cells	CD96
0	2.0438181	0.999	0.468	0	T cells	RPS24
0	2.03048787	0.371	0.075	0	T cells	RPS10
0	2.02990279	0.319	0.063	0	T cells	CFAP97
0	2.02600095	0.437	0.088	0	T cells	ZNF101
0	2.02315017	0.439	0.095	0	T cells	ZNF91
0	2.01115921	0.607	0.12	0	T cells	PRKACB
0	2.00629034	0.491	0.093	0	T cells	BIRC3
0	1.99470982	0.495	0.121	0	T cells	PABPC4
0	1.99458361	0.925	0.321	0	T cells	EIF4B
0	1.99033223	0.678	0.188	0	T cells	ST13
0	1.98938899	0.897	0.293	0	T cells	HSPA8
0	1.97850918	0.943	0.241	0	T cells	CD52
0	1.97473952	0.256	0.049	0	T cells	PLSCR3
0	1.96657397	0.332	0.034	0	T cells	NELL2

0	1.96262205	0.443	0.056	0	T cells	INPP4B
0	1.96063473	0.371	0.079	0	T cells	OOEP
0	1.95814231	0.36	0.058	0	T cells	SVIP
0	1.94346453	0.295	0.081	0	T cells	LPAR6
0	1.93570129	0.492	0.102	0	T cells	CCND2
0	1.9333181	0.441	0.104	0	T cells	NOP58
0	1.92743655	0.315	0.037	0	T cells	CD6
0	1.92659566	0.444	0.091	0	T cells	LRRC8C
0	1.92024119	0.466	0.095	0	T cells	DENND2D
0	1.91933343	0.297	0.061	0	T cells	PHF10
0	1.91777295	0.496	0.097	0	T cells	RGS10
0	1.90992674	0.835	0.252	0	T cells	BTF3
0	1.90704856	0.589	0.155	0	T cells	TTC3
0	1.9011505	0.989	0.461	0	T cells	RPS11
0	1.89058713	0.346	0.082	0	T cells	GIMAP6
0	1.88139197	0.392	0.102	0	T cells	APEX1
0	1.87979683	0.352	0.087	0	T cells	HSPH1
0	1.87924892	0.65	0.175	0	T cells	NUCKS1
0	1.86878326	0.373	0.069	0	T cells	SEPTIN1
0	1.8643953	0.321	0.077	0	T cells	ABCE1
0	1.85824802	0.96	0.383	0	T cells	NACA
0	1.8549445	0.254	0.042	0	T cells	SNRPN
0	1.85435563	0.542	0.15	0	T cells	PA2G4
0	1.84866727	0.397	0.106	0	T cells	SMDT1
0	1.83870778	0.878	0.3	0	T cells	ANP32B
0	1.83638812	0.604	0.171	0	T cells	EIF3F
0	1.83419085	0.403	0.109	0	T cells	EPRS
0	1.82375768	0.805	0.243	0	T cells	NCL
0	1.81895579	0.565	0.151	0	T cells	PPDPF
0	1.80699288	0.605	0.171	0	T cells	DDX24
0	1.80646621	0.46	0.122	0	T cells	ATP1A1
0	1.80603459	0.298	0.058	0	T cells	OPTN
0	1.80372662	0.267	0.057	0	T cells	ARHGAP5
0	1.80292409	0.299	0.074	0	T cells	GNL3
0	1.80112196	0.802	0.269	0	T cells	COMMD6
0	1.80021824	0.513	0.145	0	T cells	SSB
0	1.79869656	0.339	0.082	0	T cells	HSPE1
0	1.79220092	0.474	0.118	0	T cells	URI1
0	1.79150908	0.627	0.184	0	T cells	RAN
0	1.79106895	0.457	0.081	0	T cells	PCED1B
0	1.78446129	0.991	0.506	0	T cells	MT-ATP8
0	1.77717031	0.503	0.136	0	T cells	CMPK1
0	1.77378508	0.901	0.271	0	T cells	PPIA
0	1.7671278	0.472	0.082	0	T cells	BCL2
0	1.76333567	0.421	0.107	0	T cells	MDFIC
0	1.75677093	0.691	0.201	0	T cells	KMT2A
0	1.751803	0.25	0.044	0	T cells	CHMP7
0	1.74036633	0.395	0.103	0	T cells	ELK4
0	1.73795547	0.318	0.084	0	T cells	NOLC1
0	1.73724938	0.998	0.482	0	T cells	PTMA

0	1.73497521	0.289	0.07	0	T cells	NOL11
0	1.73197743	0.758	0.135	0	T cells	ARL4C
0	1.73014967	0.626	0.142	0	T cells	EVL
0	1.72704392	0.285	0.049	0	T cells	ANKH
0	1.72590527	0.3	0.082	0	T cells	SSRP1
0	1.72374916	0.364	0.088	0	T cells	TMEM106B
0	1.71488155	0.833	0.275	0	T cells	CD48
0	1.7136167	0.71	0.183	0	T cells	CLEC2D
0	1.71354091	0.33	0.077	0	T cells	GPRIN3
0	1.71021066	0.437	0.114	0	T cells	NUCB2
0	1.70029743	0.299	0.076	0	T cells	CUTA
0	1.69864384	0.788	0.253	0	T cells	EIF3H
0	1.6966015	0.447	0.1	0	T cells	SPTBN1
0	1.69386192	0.422	0.057	0	T cells	THEMIS
0	1.69351042	0.263	0.042	0	T cells	SATB1-AS1
0	1.68736585	0.524	0.139	0	T cells	SPTAN1
0	1.68501115	0.321	0.068	0	T cells	ORMDL3
0	1.68441955	0.414	0.118	0	T cells	NDUFS5
0	1.68127581	1	0.924	0	T cells	MT-ND3
0	1.68072346	0.335	0.095	0	T cells	SDAD1
0	1.68005498	0.439	0.127	0	T cells	DDX18
0	1.67949766	0.985	0.611	0	T cells	MT-ND4L
0	1.66553316	0.258	0.059	0	T cells	SNHG1
0	1.66252197	0.31	0.072	0	T cells	MDN1
0	1.66144184	0.427	0.128	0	T cells	NME2
0	1.66092702	1	0.897	0	T cells	MT-ATP6
0	1.65944555	0.469	0.13	0	T cells	ERAP2
0	1.65904568	0.375	0.089	0	T cells	TMEM245
0	1.65818986	0.269	0.07	0	T cells	LMAN1
0	1.65507915	0.922	0.324	0	T cells	TMSB10
0	1.65288363	0.326	0.052	0	T cells	MLLT3
0	1.64767811	0.331	0.094	0	T cells	CCT2
0	1.64310026	0.403	0.106	0	T cells	NPAT
0	1.64275023	0.308	0.08	0	T cells	WDR43
0	1.64090188	0.306	0.078	0	T cells	HMGA1
0	1.63852624	0.294	0.085	0	T cells	MPHOSPH10
0	1.63593399	0.361	0.088	0	T cells	PPM1K
0	1.63313275	0.453	0.135	0	T cells	EIF5B
0	1.63312864	0.4	0.1	0	T cells	MCUB
0	1.63243478	0.51	0.154	0	T cells	GTF3A
0	1.63056821	0.343	0.096	0	T cells	LARS
0	1.62879843	0.995	0.728	0	T cells	MT-ND5
0	1.62349066	0.476	0.145	0	T cells	ATXN7L3B
0	1.62131306	0.651	0.18	0	T cells	SLC25A6
0	1.62004695	0.435	0.128	0	T cells	CCT4
0	1.61620221	0.776	0.275	0	T cells	HNRNPDL
0	1.61172826	0.346	0.097	0	T cells	PURA
0	1.6101687	0.354	0.099	0	T cells	NOP56
0	1.60974774	0.542	0.139	0	T cells	CD4
0	1.60604872	0.762	0.272	0	T cells	UQCRB

0	1.60268026	0.274	0.069	0	T cells	CCNG1
0	1.60190631	0.268	0.052	0	T cells	GOLGA8B
0	1.6014888	0.283	0.071	0	T cells	SNHG3
0	1.5941202	0.335	0.072	0	T cells	SLC7A6
0	1.58913033	0.415	0.122	0	T cells	GIMAP2
0	1.58793352	0.307	0.089	0	T cells	TMEM173
0	1.58704122	0.348	0.081	0	T cells	ESYT1
0	1.58661441	0.462	0.142	0	T cells	ILF3
0	1.58586221	0.362	0.098	0	T cells	PARP1
0	1.58561853	0.333	0.099	0	T cells	IMP3
0	1.58454112	0.747	0.242	0	T cells	TRAF3IP3
0	1.58396445	0.299	0.068	0	T cells	PLEKHA1
0	1.58344467	0.317	0.069	0	T cells	ITGB7
0	1.57600818	0.326	0.085	0	T cells	ZBTB4
0	1.57421934	0.536	0.167	0	T cells	G3BP1
0	1.57209262	0.27	0.048	0	T cells	SFXN1
0	1.57063972	0.526	0.166	0	T cells	RBMX
0	1.57037175	0.406	0.182	0	T cells	ITGB1
0	1.55660561	0.327	0.082	0	T cells	PLAAT4
0	1.55627212	0.999	0.923	0	T cells	MT-ND2
0	1.55512567	0.424	0.127	0	T cells	BUB3
0	1.55462477	1	0.914	0	T cells	MT-CYB
0	1.55182467	0.316	0.091	0	T cells	HTATSF1
0	1.5478567	0.295	0.086	0	T cells	USP14
0	1.54502385	0.295	0.064	0	T cells	AL591895.1
0	1.5425587	0.354	0.111	0	T cells	EIF2A
0	1.54202944	0.355	0.108	0	T cells	METAP2
0	1.53779466	0.585	0.181	0	T cells	HMGN1
0	1.53243085	0.49	0.114	0	T cells	SLFN12L
0	1.53231181	0.81	0.316	0	T cells	EIF4A2
0	1.5314103	0.292	0.077	0	T cells	SBDS
0	1.53098474	0.996	0.712	0	T cells	PABPC1
0	1.52393331	0.401	0.122	0	T cells	GSPT1
0	1.52018037	0.261	0.058	0	T cells	KPNA5
0	1.51739501	0.607	0.206	0	T cells	FXYD5
0	1.50805612	0.488	0.158	0	T cells	HSPD1
0	1.50758589	0.442	0.143	0	T cells	EIF1AX
0	1.50499438	0.412	0.126	0	T cells	DNMT1
0	1.50276691	0.274	0.08	0	T cells	ADH5
0	1.5014922	0.383	0.12	0	T cells	IPO7
0	1.5007905	0.469	0.086	0	T cells	RHOH
0	1.50072142	0.276	0.086	0	T cells	AIMP1
0	1.5005648	0.288	0.089	0	T cells	IL27RA
0	1.49802589	0.432	0.141	0	T cells	TOMM20
0	1.49720561	1	0.89	0	T cells	MT-CO1
0	1.49650479	0.421	0.106	0	T cells	WWP1
0	1.49049824	0.259	0.069	0	T cells	ICE2
0	1.48751356	0.354	0.095	0	T cells	NBEAL1
0	1.48667002	0.544	0.184	0	T cells	SYNCRIP
0	1.48573353	0.299	0.087	0	T cells	LRPPRC

0	1.48534794	0.687	0.203	0	T cells	KIF2A
0	1.48261739	0.328	0.105	0	T cells	RWDD1
0	1.47913367	1	0.945	0	T cells	MT-CO3
0	1.47691193	0.826	0.334	0	T cells	TMEM123
0	1.47139809	0.281	0.068	0	T cells	TNRC6C-AS1
0	1.46526896	0.428	0.142	0	T cells	SSBP1
0	1.45880423	0.313	0.098	0	T cells	CEBPZ
0	1.45627031	1	0.958	0	T cells	MT-ND4
0	1.45556788	0.324	0.107	0	T cells	MDH1
0	1.45551446	0.3	0.089	0	T cells	ZNF146
0	1.44810868	0.599	0.201	0	T cells	SRSF7
0	1.44742251	0.311	0.091	0	T cells	TTC37
0	1.4459706	0.668	0.217	0	T cells	PIK3R1
0	1.44501451	0.546	0.128	0	T cells	IKZF3
0	1.44440221	0.555	0.155	0	T cells	SPN
0	1.44349043	0.893	0.407	0	T cells	EEF1D
0	1.44201646	0.252	0.069	0	T cells	PTCD3
0	1.43886738	0.357	0.116	0	T cells	TMPO
0	1.43879175	0.309	0.093	0	T cells	AKAP11
0	1.43765254	0.273	0.075	0	T cells	SLC16A7
0	1.43241171	0.28	0.093	0	T cells	RAD50
0	1.43197908	0.344	0.112	0	T cells	EIF3M
0	1.43140763	0.46	0.09	0	T cells	TC2N
0	1.41922964	0.254	0.079	0	T cells	ACP1
0	1.41894008	0.698	0.23	0	T cells	SEPTIN6
0	1.41416061	0.653	0.228	0	T cells	HNRNPH1
0	1.41216851	0.397	0.124	0	T cells	TRIM14
0	1.4112727	0.403	0.14	0	T cells	BANF1
0	1.4078488	0.353	0.115	0	T cells	ABRACL
0	1.4053942	0.503	0.174	0	T cells	OIP5-AS1
0	1.39547995	0.349	0.12	0	T cells	RAD23A
0	1.3944825	0.289	0.086	0	T cells	CAND1
0	1.39351587	0.725	0.24	0	T cells	UCP2
0	1.39244557	0.593	0.184	0	T cells	MLLT6
0	1.39074863	0.367	0.124	0	T cells	RPL7L1
0	1.3840772	0.349	0.117	0	T cells	RFC1
0	1.38217325	0.344	0.113	0	T cells	EIF3G
0	1.38012497	0.35	0.12	0	T cells	PYURF
0	1.37854251	0.79	0.289	0	T cells	ATP5MC2
0	1.36680773	0.384	0.135	0	T cells	EIF3I
0	1.36383269	0.273	0.088	0	T cells	APOL3
0	1.36321647	0.353	0.118	0	T cells	UBE2G2
0	1.36149973	0.261	0.059	0	T cells	NLRC3
0	1.36060278	0.307	0.105	0	T cells	NDUFA12
0	1.35730784	0.383	0.126	0	T cells	DUT
0	1.35383742	0.298	0.086	0	T cells	MT-ND6
0	1.35167657	0.513	0.178	0	T cells	NDUFA4
0	1.35000302	0.531	0.178	0	T cells	NDFIP1
0	1.34793214	0.304	0.061	0	T cells	PRKCQ
0	1.34735876	0.416	0.127	0	T cells	ANXA6

0	1.34601162	0.556	0.17	0	T cells	RASA3
0	1.34579674	0.418	0.149	0	T cells	ATP5F1A
0	1.33988925	0.312	0.097	0	T cells	STYX
0	1.33785418	0.325	0.089	0	T cells	ZMYND11
0	1.33645378	0.362	0.131	0	T cells	TOMM6
0	1.33147356	0.698	0.266	0	T cells	LEPROTL1
0	1.33123463	0.314	0.106	0	T cells	TCERG1
0	1.33070718	1	0.941	0	T cells	MT-CO2
0	1.32749225	0.352	0.074	0	T cells	CDR2
0	1.32519583	0.615	0.207	0	T cells	TMC8
0	1.32516228	0.431	0.16	0	T cells	EID1
0	1.32371404	0.302	0.084	0	T cells	CCDC66
0	1.31792167	0.443	0.127	0	T cells	ESYT2
0	1.31513276	0.318	0.109	0	T cells	HSPA9
0	1.31476956	0.425	0.104	0	T cells	RORA
0	1.31263409	0.313	0.102	0	T cells	THUMPD1
0	1.31136236	0.768	0.315	0	T cells	ATM
0	1.31066459	0.297	0.09	0	T cells	UXT
0	1.30600059	0.308	0.107	0	T cells	ARL2BP
0	1.30566355	0.274	0.095	0	T cells	MRPL34
0	1.3039162	0.277	0.095	0	T cells	ELK3
0	1.30291138	0.264	0.077	0	T cells	TECR
0	1.30283098	0.4	0.135	0	T cells	TPP2
0	1.30185222	0.361	0.129	0	T cells	RSL24D1
0	1.29930752	0.462	0.153	0	T cells	CHD3
0	1.29922879	0.377	0.119	0	T cells	MALT1
0	1.29868873	0.253	0.079	0	T cells	BLOC1S4
0	1.29361357	0.323	0.116	0	T cells	ATP5PO
0	1.29335271	0.347	0.086	0	T cells	TTC39C
0	1.29291192	0.364	0.131	0	T cells	SESN3
0	1.29286098	0.297	0.11	0	T cells	LRRC58
0	1.29082453	0.44	0.153	0	T cells	FUBP1
0	1.29008436	0.291	0.099	0	T cells	CLNS1A
0	1.2885467	0.308	0.105	0	T cells	CYP20A1
0	1.28820023	0.748	0.305	0	T cells	MATR3
0	1.2878292	0.966	0.555	0	T cells	UBA52
0	1.28778944	0.873	0.326	0	T cells	YBX1
0	1.28733075	0.643	0.243	0	T cells	SERBP1
0	1.2858444	0.705	0.275	0	T cells	PSIP1
0	1.28580459	0.407	0.142	0	T cells	LARP1
0	1.28235068	0.75	0.298	0	T cells	SEPTIN9
0	1.28119815	0.465	0.111	0	T cells	KLF12
0	1.27906052	0.266	0.071	0	T cells	UXS1
0	1.27705919	0.394	0.122	0	T cells	NAP1L4
0	1.27657559	0.275	0.098	0	T cells	POLE3
0	1.27515491	0.385	0.14	0	T cells	NDUFB2
0	1.27514292	0.39	0.14	0	T cells	ERP29
0	1.27278273	0.302	0.092	0	T cells	NAA16
0	1.26978149	0.553	0.202	0	T cells	KTN1
0	1.26921564	0.309	0.11	0	T cells	SSR4

0	1.26885223	0.296	0.107	0	T cells	MESD
0	1.26222504	0.352	0.116	0	T cells	CASP8AP2
0	1.2618517	0.31	0.101	0	T cells	DARS
0	1.26008323	0.501	0.183	0	T cells	TCEA1
0	1.25844317	0.26	0.088	0	T cells	NOL8
0	1.2570111	0.288	0.073	0	T cells	USP9Y
0	1.25686702	0.343	0.104	0	T cells	SUCLG2
0	1.25655408	0.281	0.096	0	T cells	C19orf53
0	1.25330989	0.328	0.12	0	T cells	SNRPE
0	1.25295631	0.351	0.128	0	T cells	PHB2
0	1.2484105	0.418	0.155	0	T cells	SRP72
0	1.24601855	0.361	0.129	0	T cells	SRSF6
0	1.24492107	0.266	0.093	0	T cells	NDUFB9
0	1.2406054	0.365	0.126	0	T cells	AIDA
0	1.2372143	0.265	0.086	0	T cells	SF3B3
0	1.23690562	0.496	0.158	0	T cells	MGAT4A
0	1.23668284	0.258	0.093	0	T cells	DNAJC21
0	1.23595914	0.393	0.148	0	T cells	UFM1
0	1.23374064	0.409	0.154	0	T cells	NDUFB8
0	1.2327174	0.336	0.122	0	T cells	TRIM44
0	1.23150061	0.865	0.419	0	T cells	FAU
0	1.22415064	0.269	0.085	0	T cells	UBA2
0	1.22366729	0.328	0.118	0	T cells	NOL7
0	1.22247901	0.409	0.15	0	T cells	UQCRH
0	1.22053317	0.573	0.225	0	T cells	COX4I1
0	1.21792311	0.539	0.2	0	T cells	C9orf78
0	1.21734646	0.262	0.091	0	T cells	CHORDC1
0	1.21271806	0.278	0.106	0	T cells	CCT8
0	1.21131306	0.257	0.089	0	T cells	PSMC5
0	1.20974711	0.273	0.095	0	T cells	ICE1
0	1.20966896	0.286	0.11	0	T cells	CCT6A
0	1.20918113	0.306	0.106	0	T cells	RDH11
0	1.20841921	0.275	0.093	0	T cells	RBBP7
0	1.20802	0.26	0.086	0	T cells	DKC1
0	1.2076945	0.511	0.195	0	T cells	ZRANB2
0	1.20701514	0.353	0.079	0	T cells	SCML4
0	1.19870109	0.927	0.533	0	T cells	SRSF5
0	1.19796117	0.252	0.091	0	T cells	NDUFB11
0	1.19772077	0.549	0.23	0	T cells	GSTK1
0	1.19587702	0.329	0.099	0	T cells	CRTC3
0	1.19572836	0.595	0.23	0	T cells	TRAM1
0	1.19345172	0.58	0.224	0	T cells	UHMK1
0	1.19247257	0.262	0.099	0	T cells	GRSF1
0	1.18658169	0.558	0.22	0	T cells	HNRNPR
0	1.18575229	0.694	0.288	0	T cells	HNRNPA3
0	1.18492532	0.251	0.076	0	T cells	AC092821.3
0	1.18459297	0.695	0.247	0	T cells	HSP90B1
0	1.18441004	0.261	0.1	0	T cells	SEM1
0	1.18043526	0.496	0.173	0	T cells	GLS
0	1.1802819	0.678	0.223	0	T cells	FYN

0	1.17577454	0.278	0.102	0	T cells	PTDSS1
0	1.17564606	0.302	0.116	0	T cells	ZNF706
0	1.17487831	0.343	0.079	0	T cells	TXK
0	1.17345697	0.266	0.08	0	T cells	CD2AP
0	1.17143723	0.352	0.131	0	T cells	BDP1
0	1.16663408	0.253	0.057	0	T cells	CD226
0	1.16379477	0.497	0.189	0	T cells	EIF3K
0	1.16309702	0.372	0.138	0	T cells	UBE3A
0	1.16224805	0.437	0.148	0	T cells	CCDC88C
0	1.16113899	0.259	0.089	0	T cells	COX20
0	1.1596148	0.304	0.112	0	T cells	MTA2
0	1.1559604	0.272	0.098	0	T cells	NDUFA5
0	1.1550885	0.787	0.342	0	T cells	PFDN5
0	1.15505834	0.289	0.103	0	T cells	UPF3A
0	1.15462573	0.595	0.228	0	T cells	IRF2BP2
0	1.15359481	0.338	0.125	0	T cells	API5
0	1.15246527	0.441	0.166	0	T cells	BAZ1B
0	1.15214994	0.428	0.165	0	T cells	KRTCAP2
0	1.1514759	0.366	0.143	0	T cells	ATP5MC3
0	1.14590016	0.282	0.061	0	T cells	FHIT
0	1.14160856	0.546	0.178	0	T cells	AAK1
0	1.13484431	0.45	0.171	0	T cells	LPXN
0	1.12836769	0.278	0.075	0	T cells	HELB
0	1.1282088	0.272	0.089	0	T cells	WDR6
0	1.12291543	0.322	0.119	0	T cells	UBP1
0	1.12044846	0.276	0.078	0	T cells	SLAMF6
0	1.1190912	0.86	0.438	0	T cells	RPS9
0	1.11459125	0.475	0.144	0	T cells	TNRC6C
0	1.11404744	0.603	0.252	0	T cells	NONO
0	1.11210615	0.259	0.073	0	T cells	CCDC7
0	1.10688156	0.269	0.109	0	T cells	HIGD2A
0	1.1041571	0.37	0.142	0	T cells	ERAP1
0	1.10297136	0.254	0.081	0	T cells	MPRIP
0	1.10196063	0.29	0.108	0	T cells	KARS
0	1.09720118	0.255	0.084	0	T cells	PHF14
0	1.09505953	0.25	0.073	0	T cells	ODF2L
0	1.09473194	0.26	0.094	0	T cells	TNFSF8
0	1.09265364	0.491	0.188	0	T cells	FNBP4
0	1.09256234	1	0.934	0	T cells	MT-ND1
0	1.08857097	0.528	0.215	0	T cells	TUBB
0	1.08846665	0.447	0.132	0	T cells	DOCK10
0	1.08585141	0.442	0.179	0	T cells	EIF3D
0	1.08470653	0.544	0.223	0	T cells	TOP2B
0	1.08366364	0.526	0.203	0	T cells	ANAPC16
0	1.08356971	0.311	0.113	0	T cells	ZBTB38
0	1.08351316	0.538	0.227	0	T cells	SRSF3
0	1.08106011	0.314	0.105	0	T cells	OFD1
0	1.08026772	0.351	0.136	0	T cells	FAM78A
0	1.079783	0.327	0.127	0	T cells	ABCF1
0	1.07891986	0.373	0.137	0	T cells	CRBN

0	1.07289971	0.405	0.163	0	T cells	SMARCE1
0	1.07231088	0.875	0.434	0	T cells	HSP90AA1
0	1.06832161	0.295	0.113	0	T cells	NASP
0	1.06354354	0.394	0.161	0	T cells	TASOR
0	1.06147667	0.398	0.165	0	T cells	RBM17
0	1.06110046	0.28	0.112	0	T cells	DDB1
0	1.06011997	0.441	0.181	0	T cells	POLR1D
0	1.05578871	0.74	0.34	0	T cells	SUN2
0	1.05543749	0.442	0.181	0	T cells	WBP11
0	1.05411085	0.256	0.096	0	T cells	KLRK1
0	1.05138184	0.272	0.115	0	T cells	SUMO3
0	1.05000367	0.395	0.159	0	T cells	ERCC5
0	1.04967426	0.462	0.17	0	T cells	CD99
0	1.04677013	0.254	0.101	0	T cells	FYTTD1
0	1.04583243	0.275	0.106	0	T cells	NXPE3
0	1.04351882	0.367	0.144	0	T cells	ANP32E
0	1.04051709	0.575	0.238	0	T cells	C11orf58
0	1.03887655	0.332	0.113	0	T cells	RNF125
0	1.03546711	0.316	0.123	0	T cells	SNU13
0	1.0324701	0.32	0.101	0	T cells	HIST1H4C
0	1.03210028	0.331	0.131	0	T cells	UBE2N
0	1.03029127	0.275	0.111	0	T cells	ZNF770
0	1.02750983	0.493	0.209	0	T cells	U2SURP
0	1.02649303	0.318	0.063	0	T cells	GRAP2
0	1.02618734	0.351	0.141	0	T cells	CACYBP
0	1.02606519	0.254	0.098	0	T cells	NUDT21
0	1.02508148	0.455	0.171	0	T cells	VAMP8
0	1.02360553	0.253	0.092	0	T cells	CENPC
0	1.02157902	0.276	0.101	0	T cells	TRIM52
0	1.01809177	0.287	0.122	0	T cells	SNRPB2
0	1.01736232	0.271	0.106	0	T cells	TMEM50B
0	1.01700486	0.664	0.267	0	T cells	MTDH
0	1.01585277	0.296	0.117	0	T cells	LSM8
0	1.01428817	0.962	0.599	0	T cells	CALM1
0	1.01343848	0.434	0.176	0	T cells	TRA2B
0	1.01188955	0.363	0.152	0	T cells	COX7A2L
0	1.01004518	0.373	0.12	0	T cells	AMBRA1
0	1.00833684	0.257	0.097	0	T cells	GABPB1-IT1
0	1.00675277	0.521	0.212	0	T cells	M6PR
0	1.00663732	0.323	0.122	0	T cells	CEP57
0	1.00581282	0.25	0.084	0	T cells	LIX1L
0	1.00542741	0.332	0.131	0	T cells	ZNF800
0	1.00277755	0.258	0.1	0	T cells	CD84
0	1.00137884	0.825	0.44	0	T cells	RBL2
0	1.000833	0.251	0.084	0	T cells	CHD6
0	0.99913918	0.799	0.41	0	T cells	LTB
0	0.99535815	0.524	0.227	0	T cells	SRSF2
0	0.99501857	0.652	0.275	0	T cells	OST4
0	0.994736	0.382	0.141	0	T cells	SSBP3
0	0.99398922	0.278	0.108	0	T cells	TRIM28

0	0.99347343	0.298	0.122	0	T cells	NECAP2
0	0.99077199	0.365	0.147	0	T cells	ZBTB1
0	0.99046789	0.33	0.148	0	T cells	IL10RA
0	0.98835031	0.328	0.123	0	T cells	MRFAP1L1
0	0.98462687	0.565	0.225	0	T cells	CALR
0	0.98419966	0.349	0.142	0	T cells	ZBED5
0	0.98370579	0.79	0.388	0	T cells	SRSF11
0	0.98301376	0.403	0.16	0	T cells	UBTF
0	0.98262709	0.389	0.166	0	T cells	TARDBP
0	0.98244569	0.318	0.124	0	T cells	DHX36
0	0.98115386	0.283	0.115	0	T cells	MEAF6
0	0.98068172	0.47	0.194	0	T cells	TES
0	0.97871857	0.579	0.253	0	T cells	GCC2
0	0.97852926	0.297	0.118	0	T cells	THAP12
0	0.97718727	0.636	0.273	0	T cells	RBM3
0	0.97668691	0.316	0.138	0	T cells	CAPN2
0	0.9750227	0.277	0.121	0	T cells	DYNC1I2
0	0.9721845	0.296	0.119	0	T cells	TRIM4
0	0.97150404	0.257	0.092	0	T cells	PPWD1
0	0.96945794	0.253	0.093	0	T cells	MGA
0	0.9685479	0.476	0.21	0	T cells	ATP5PD
0	0.96605845	0.404	0.177	0	T cells	EIF5A
0	0.96529786	0.454	0.186	0	T cells	PRRC2B
0	0.96458744	0.269	0.112	0	T cells	RNF187
0	0.96318911	0.415	0.168	0	T cells	EZR
0	0.95946224	0.852	0.431	0	T cells	SET
0	0.95939546	0.295	0.126	0	T cells	GIMAP8
0	0.95835907	0.388	0.162	0	T cells	SUPT16H
0	0.958189	0.706	0.329	0	T cells	ATP5MG
0	0.95747547	0.268	0.116	0	T cells	SEC61G
0	0.95734674	0.273	0.114	0	T cells	NUTF2
0	0.95613307	0.4	0.141	0	T cells	ARHGEF3
0	0.9543024	0.713	0.359	0	T cells	CYLD
0	0.95424274	0.594	0.265	0	T cells	PRMT2
0	0.95101636	0.57	0.262	0	T cells	RPL36AL
0	0.94695397	0.404	0.169	0	T cells	SEC31A
0	0.94609079	0.279	0.116	0	T cells	NDUFC1
0	0.94480944	0.26	0.096	0	T cells	DDX50
0	0.94386889	0.406	0.172	0	T cells	RPN2
0	0.94130329	0.55	0.159	0	T cells	PRKCH
0	0.9364464	0.354	0.155	0	T cells	RBBP4
0	0.9363653	0.646	0.301	0	T cells	SFPQ
0	0.9333136	0.574	0.247	0	T cells	LNPEP
0	0.93263755	0.41	0.178	0	T cells	SRSF10
0	0.92746295	0.32	0.141	0	T cells	NDUFB4
0	0.92717727	0.299	0.076	0	T cells	PRKCA
0	0.92630746	0.346	0.147	0	T cells	TIA1
0	0.92558679	0.399	0.178	0	T cells	SRP9
0	0.92545914	0.365	0.162	0	T cells	SNRPG
0	0.92452476	0.413	0.179	0	T cells	THOC2

0	0.92447693	0.535	0.17	0	T cells	ST6GAL1
0	0.92028739	0.263	0.101	0	T cells	SP140L
0	0.92018393	0.254	0.09	0	T cells	RALA
0	0.91826439	0.494	0.212	0	T cells	DDX21
0	0.91656067	0.44	0.195	0	T cells	SMC3
0	0.91234599	0.323	0.124	0	T cells	GBP4
0	0.91000432	0.441	0.161	0	T cells	S100A10
0	0.90798048	0.442	0.16	0	T cells	PLAC8
0	0.90791546	0.54	0.233	0	T cells	PCM1
0	0.90753317	0.33	0.133	0	T cells	SNHG16
0	0.90562868	0.35	0.147	0	T cells	XRCC6
0	0.90547471	0.919	0.592	0	T cells	SARAF
0	0.90516888	0.469	0.209	0	T cells	UBE2V1
0	0.90484777	0.379	0.164	0	T cells	RANBP2
0	0.9044479	0.349	0.148	0	T cells	SMC5
0	0.90307181	0.382	0.124	0	T cells	PTPN4
0	0.90155689	0.27	0.116	0	T cells	ORMDL1
0	0.90145192	0.439	0.193	0	T cells	DHX9
0	0.90091155	0.362	0.145	0	T cells	TNRC6A
0	0.90060909	0.366	0.086	0	T cells	BACH2
0	0.89855318	0.308	0.128	0	T cells	TTC14
0	0.89850863	0.252	0.115	0	T cells	NDUFC2
0	0.89572717	0.862	0.471	0	T cells	ADD3
0	0.89477755	0.391	0.146	0	T cells	DAPK1
0	0.89394305	0.436	0.195	0	T cells	HNRNPA0
0	0.89386615	0.263	0.107	0	T cells	WHAMM
0	0.88684867	0.357	0.157	0	T cells	YWHAQ
0	0.88591307	0.473	0.197	0	T cells	HCST
0	0.88506236	0.378	0.165	0	T cells	CNOT7
0	0.88312051	0.346	0.146	0	T cells	NFATC2IP
0	0.88037341	0.269	0.108	0	T cells	ZNF451
0	0.87700211	0.309	0.134	0	T cells	DNTTIP2
0	0.87523071	0.666	0.308	0	T cells	EIF3A
0	0.87115316	0.261	0.082	0	T cells	CNST
0	0.87036068	0.533	0.244	0	T cells	AHNAK
0	0.86676489	0.471	0.119	0	T cells	SKAP1
0	0.8652928	0.268	0.117	0	T cells	DDX27
0	0.86447858	0.419	0.106	0	T cells	CD247
0	0.86348242	0.364	0.158	0	T cells	PCNP
0	0.86333513	0.254	0.077	0	T cells	NFATC2
0	0.86120279	0.928	0.548	0	T cells	HNRNPA2B1
0	0.85888437	0.563	0.256	0	T cells	PTGES3
0	0.85864018	0.441	0.17	0	T cells	TUT4
0	0.85826828	0.771	0.374	0	T cells	FAM107B
0	0.85817425	0.386	0.177	0	T cells	OXA1L
0	0.85732381	0.379	0.14	0	T cells	CCSER2
0	0.85610693	0.498	0.232	0	T cells	ATXN2L
0	0.85517805	0.364	0.148	0	T cells	HUWE1
0	0.85203935	0.258	0.121	0	T cells	APPL1
0	0.84716481	0.444	0.193	0	T cells	STT3B

0	0.84537879	0.384	0.157	0	T cells	DYNLL2
0	0.84361172	0.293	0.137	0	T cells	TUFM
0	0.84265039	0.254	0.101	0	T cells	CPSF6
0	0.84004248	0.417	0.168	0	T cells	PDS5A
0	0.83808853	0.394	0.182	0	T cells	ZC3H15
0	0.83445545	0.363	0.15	0	T cells	ZNF131
0	0.83182729	0.534	0.254	0	T cells	ITGA4
0	0.83047315	0.259	0.117	0	T cells	KIAA1143
0	0.82975053	0.583	0.281	0	T cells	PNRC2
0	0.82954984	0.82	0.338	0	T cells	SAMHD1
0	0.82387885	0.558	0.262	0	T cells	HNRNPM
0	0.82316615	0.286	0.108	0	T cells	KAT6B
0	0.82258781	0.256	0.096	0	T cells	CAMK2D
0	0.82211897	0.314	0.14	0	T cells	TRIP11
0	0.81984135	0.646	0.318	0	T cells	EIF2S3
0	0.81978232	0.329	0.155	0	T cells	SLC25A5
0	0.81793613	0.63	0.302	0	T cells	LUC7L3
0	0.81696497	0.655	0.286	0	T cells	CYFIP2
0	0.8151774	0.291	0.138	0	T cells	ERH
0	0.81464726	0.305	0.122	0	T cells	RSBN1
0	0.81449027	0.668	0.334	0	T cells	SRRM1
0	0.81177668	0.498	0.238	0	T cells	EFCAB14
0	0.81019112	0.291	0.129	0	T cells	USP16
0	0.80336095	0.298	0.117	0	T cells	PCSK7
0	0.80158597	0.505	0.229	0	T cells	VPS13C
0	0.80120602	0.362	0.164	0	T cells	PCMTD2
0	0.79918693	0.474	0.227	0	T cells	PRPF4B
0	0.79679229	0.251	0.085	0	T cells	PPP3CC
0	0.795384	0.398	0.177	0	T cells	CCAR1
0	0.79388525	0.447	0.207	0	T cells	EBLN3P
0	0.79345017	0.406	0.187	0	T cells	OCIAD1
0	0.79341576	0.281	0.125	0	T cells	EDEM1
0	0.79319174	0.258	0.092	0	T cells	CCDC18-AS1
0	0.79251016	0.259	0.116	0	T cells	PRKX
0	0.79215973	0.281	0.119	0	T cells	ZNF644
0	0.79106232	0.259	0.085	0	T cells	AKT3
0	0.78907003	0.311	0.129	0	T cells	DAP3
0	0.78765532	0.386	0.155	0	T cells	USP47
0	0.78543742	0.348	0.158	0	T cells	RNPS1
0	0.78419328	0.431	0.205	0	T cells	LDHA
0	0.7839911	0.252	0.108	0	T cells	PRPF38A
0	0.7839507	0.326	0.151	0	T cells	MED1
0	0.78366126	0.446	0.202	0	T cells	XPO1
0	0.78337907	0.253	0.1	0	T cells	TAF3
0	0.78281485	0.324	0.147	0	T cells	TFAM
0	0.78133493	0.25	0.114	0	T cells	MRPL20
0	0.77985597	0.394	0.187	0	T cells	SPCS1
0	0.77809441	0.578	0.231	0	T cells	PDE7A
0	0.77797929	0.254	0.114	0	T cells	FBXO7
0	0.77530554	0.487	0.231	0	T cells	TMED10

0	0.77520081	0.455	0.219	0	T cells	PTBP1
0	0.76891224	0.345	0.099	0	T cells	TNIK
0	0.76599879	0.272	0.127	0	T cells	TCP1
0	0.76597736	0.436	0.192	0	T cells	CNTRL
0	0.76242873	0.62	0.275	0	T cells	PAG1
0	0.76234106	0.262	0.106	0	T cells	ZNF506
0	0.76222862	0.282	0.138	0	T cells	SCP2
0	0.76031966	0.274	0.102	0	T cells	PRKY
0	0.75864874	0.302	0.137	0	T cells	EIF3J
0	0.75694511	0.278	0.134	0	T cells	GLUD1
0	0.75576748	0.271	0.111	0	T cells	TRPM7
0	0.75558191	0.552	0.259	0	T cells	NORAD
0	0.75543044	0.285	0.126	0	T cells	RAB29
0	0.75541757	0.508	0.206	0	T cells	RAPGEF6
0	0.75404604	0.362	0.17	0	T cells	DHX15
0	0.75302389	0.448	0.215	0	T cells	TRMT112
0	0.75263843	0.346	0.162	0	T cells	RIF1
0	0.75152817	0.428	0.195	0	T cells	RBM26
0	0.75035113	0.327	0.149	0	T cells	DYNC1H1
0	0.74837212	0.412	0.198	0	T cells	FXR1
0	0.74759176	0.689	0.301	0	T cells	TNFAIP8
0	0.74642974	0.273	0.135	0	T cells	EIF4G1
0	0.74584236	0.322	0.151	0	T cells	BRD7
0	0.74361946	0.463	0.226	0	T cells	EIF2S2
0	0.74206169	0.412	0.155	0	T cells	SERINC5
0	0.74034974	0.564	0.264	0	T cells	CNOT6L
0	0.73999997	0.277	0.134	0	T cells	VPS36
0	0.73864112	0.296	0.149	0	T cells	SSR3
0	0.73060691	0.505	0.251	0	T cells	SMARCA5
0	0.73012361	0.275	0.102	0	T cells	CCDC91
0	0.73001076	0.375	0.183	0	T cells	ZKSCAN1
0	0.72881437	0.699	0.365	0	T cells	HP1BP3
0	0.72834704	0.286	0.121	0	T cells	DNAJB1
0	0.72814694	0.278	0.118	0	T cells	SF3A3
0	0.72722444	0.396	0.185	0	T cells	REST
0	0.72614023	0.43	0.212	0	T cells	TIAL1
0	0.72514626	0.338	0.149	0	T cells	PPHLN1
0	0.72067154	0.26	0.108	0	T cells	AP1G2
0	0.71615823	0.606	0.289	0	T cells	MYCBP2
0	0.7158265	0.784	0.436	0	T cells	GIMAP4
0	0.71463584	0.253	0.109	0	T cells	TMEM14B
0	0.71178597	0.321	0.118	0	T cells	CDC14A
0	0.70476374	0.547	0.284	0	T cells	PNN
0	0.70397649	0.455	0.229	0	T cells	TMEM258
0	0.70328317	0.475	0.211	0	T cells	SYNRG
0	0.7028257	0.457	0.215	0	T cells	LUC7L2
0	0.69975951	0.374	0.187	0	T cells	KHSRP
0	0.69744971	0.433	0.147	0	T cells	RUNX3
0	0.69744587	0.267	0.134	0	T cells	CLDND1
0	0.69367315	0.346	0.157	0	T cells	RPA2

0	0.6935749	0.605	0.305	0	T cells	BTN3A2
0	0.69266247	0.355	0.162	0	T cells	PRPF8
0	0.69229062	0.447	0.218	0	T cells	TAF1D
0	0.69138928	0.552	0.288	0	T cells	SSR2
0	0.689806	0.549	0.277	0	T cells	SLC25A3
0	0.68939037	0.262	0.088	0	T cells	TSPOAP1-AS1
0	0.68507042	0.314	0.148	0	T cells	FNTA
0	0.68437852	0.385	0.161	0	T cells	PHC3
0	0.68361212	0.416	0.206	0	T cells	MAZ
0	0.68239882	0.351	0.131	0	T cells	MSI2
0	0.67962949	0.283	0.148	0	T cells	COPZ1
0	0.67929886	0.317	0.143	0	T cells	DIDO1
0	0.67895726	0.484	0.249	0	T cells	CGGBP1
0	0.67872004	0.74	0.397	0	T cells	ZC3HAV1
0	0.6762334	0.595	0.313	0	T cells	MAN1A2
0	0.67598882	0.715	0.379	0	T cells	HNRNPD
0	0.67565762	0.386	0.143	0	T cells	HIVEP2
0	0.6730962	0.348	0.165	0	T cells	G3BP2
0	0.67175406	0.318	0.152	0	T cells	ETNK1
0	0.66998685	0.949	0.678	0	T cells	DDX5
0	0.66980257	0.302	0.149	0	T cells	TMED4
0	0.66813963	0.582	0.272	0	T cells	MACF1
0	0.66800532	0.258	0.12	0	T cells	CBLL1
0	0.66766401	0.269	0.123	0	T cells	FAM133B
0	0.66661753	0.637	0.335	0	T cells	CD47
0	0.66562097	0.254	0.128	0	T cells	AGGF1
0	0.6655047	0.304	0.135	0	T cells	IRF3
0	0.66226003	0.644	0.345	0	T cells	CBX3
0	0.66139345	0.284	0.139	0	T cells	PPM1G
0	0.66055132	0.486	0.228	0	T cells	PDIA3
0	0.65761662	0.427	0.189	0	T cells	SPATA13
0	0.65583308	0.454	0.233	0	T cells	ATP5PB
0	0.64974737	0.265	0.13	0	T cells	GANAB
0	0.64920599	0.521	0.273	0	T cells	HNRNPF
0	0.64814952	0.306	0.148	0	T cells	USP1
0	0.64808272	0.315	0.16	0	T cells	MAN2A1
0	0.64778315	0.513	0.251	0	T cells	ENO1
0	0.64351185	0.324	0.155	0	T cells	SNRNP200
0	0.64207054	0.32	0.149	0	T cells	ANAPC5
0	0.64194253	0.3	0.144	0	T cells	HMGN4
0	0.64169355	0.277	0.111	0	T cells	PHF1
0	0.64145535	0.39	0.187	0	T cells	EIF2AK1
0	0.6401969	0.512	0.269	0	T cells	SKP1
0	0.63889777	0.511	0.262	0	T cells	UBB
0	0.63838455	0.368	0.183	0	T cells	TBCA
0	0.6341862	0.715	0.351	0	T cells	CD44
0	0.63394127	0.25	0.109	0	T cells	LONP2
0	0.63320755	0.29	0.125	0	T cells	ZNF721
0	0.63087019	0.33	0.148	0	T cells	FAM117A
0	0.62788187	0.26	0.105	0	T cells	ASXL1

0	0.62772186	0.254	0.135	0	T cells	ATP5F1C
0	0.62229941	0.302	0.151	0	T cells	TMEM248
0	0.62106419	0.467	0.211	0	T cells	ST3GAL1
0	0.62098682	0.255	0.122	0	T cells	KPNA3
0	0.62082433	0.337	0.175	0	T cells	DNAJC8
0	0.62025063	0.377	0.193	0	T cells	HADHA
0	0.61585614	0.365	0.175	0	T cells	HDAC1
0	0.61548705	0.295	0.15	0	T cells	RBX1
0	0.61496061	0.421	0.191	0	T cells	PPP1R2
0	0.61481056	0.81	0.49	0	T cells	CSDE1
0	0.61303202	0.295	0.144	0	T cells	MFNG
0	0.61248664	0.474	0.194	0	T cells	FOXO1
0	0.60949041	0.41	0.21	0	T cells	DDX3Y
0	0.60919563	0.296	0.137	0	T cells	ZNF276
0	0.60742738	0.645	0.351	0	T cells	KHDRBS1
0	0.60307962	0.255	0.118	0	T cells	TECPR1
0	0.60165737	0.385	0.191	0	T cells	CCNL2
0	0.59838212	0.297	0.16	0	T cells	PARK7
0	0.598222	0.281	0.134	0	T cells	YLPM1
0	0.59687224	0.299	0.149	0	T cells	DDX42
0	0.59499681	0.374	0.181	0	T cells	SERPINB9
0	0.59350616	0.595	0.318	0	T cells	WDR82
0	0.59009494	0.418	0.203	0	T cells	CEP85L
0	0.58978209	0.257	0.114	0	T cells	TRRAP
0	0.58924716	0.314	0.157	0	T cells	PRDX6
0	0.58594255	0.336	0.171	0	T cells	CS
0	0.58294419	0.606	0.3	0	T cells	PIM2
0	0.58268514	0.315	0.167	0	T cells	SRSF1
0	0.5825829	0.361	0.181	0	T cells	DENR
0	0.58103827	0.265	0.122	0	T cells	ZNF430
0	0.57750508	0.584	0.32	0	T cells	ARGLU1
0	0.57441171	0.265	0.13	0	T cells	C21orf91
0	0.57271867	0.416	0.22	0	T cells	ATP5ME
0	0.57110408	0.282	0.155	0	T cells	NARS
0	0.57068567	0.423	0.212	0	T cells	OGT
0	0.56962517	0.534	0.278	0	T cells	NKTR
0	0.56816813	0.359	0.188	0	T cells	ATP5IF1
0	0.56197747	0.527	0.26	0	T cells	GLG1
0	0.56094976	0.295	0.155	0	T cells	SREK1
0	0.56078118	0.272	0.14	0	T cells	PTPN11
0	0.55985123	0.304	0.152	0	T cells	UBQLN2
0	0.55891841	0.388	0.195	0	T cells	TOB1
0	0.55690306	0.333	0.176	0	T cells	CTR9
0	0.55626693	0.772	0.441	0	T cells	ARL6IP5
0	0.55496921	0.302	0.163	0	T cells	ATP5MD
0	0.55170804	0.308	0.164	0	T cells	LGALS8
0	0.55045413	0.338	0.157	0	T cells	LINC00623
0	0.54884869	0.442	0.242	0	T cells	RBBP6
0	0.54879373	0.303	0.158	0	T cells	CCDC59
0	0.54618415	0.263	0.15	0	T cells	PDIA6

0	0.54613258	0.286	0.158	0	T cells	UQCRQ
0	0.54507472	0.317	0.152	0	T cells	MAPRE2
0	0.54397172	0.462	0.252	0	T cells	VOPP1
0	0.53555715	0.63	0.33	0	T cells	EMB
0	0.53479758	0.321	0.176	0	T cells	UQCR10
0	0.53316966	0.419	0.226	0	T cells	PPP1CC
0	0.53109292	0.256	0.134	0	T cells	CHMP4A
0	0.53093078	0.3	0.164	0	T cells	TMCO1
0	0.52576145	0.776	0.413	0	T cells	FOXP1
0	0.52209215	0.28	0.134	0	T cells	ANKIB1
0	0.52121111	0.487	0.223	0	T cells	ACAP1
0	0.52028893	0.267	0.134	0	T cells	CAMLG
0	0.5197207	0.448	0.253	0	T cells	RBM8A
0	0.51842983	0.376	0.19	0	T cells	SHFL
0	0.51801179	0.434	0.209	0	T cells	TLK1
0	0.51573002	0.277	0.133	0	T cells	QRICH1
0	0.51547698	0.466	0.212	0	T cells	ITPKB
0	0.51531636	0.397	0.207	0	T cells	CERK
0	0.51444567	0.264	0.12	0	T cells	RAB3GAP1
0	0.50672693	0.456	0.253	0	T cells	HNRNPL
0	0.50447992	0.517	0.292	0	T cells	USP7
0	0.50431169	0.494	0.257	0	T cells	PUM1
0	0.50405042	0.59	0.297	0	T cells	CANX
0	0.50383561	0.474	0.238	0	T cells	APOL6
0	0.50335331	0.344	0.177	0	T cells	CNOT2
0	0.50278454	0.293	0.158	0	T cells	SYPL1
0	0.50235611	0.51	0.248	0	T cells	EIF4A1
0	0.50101802	0.357	0.197	0	T cells	ATP5PF
0	0.49721364	0.581	0.327	0	T cells	ZC3H6
0	0.49662729	0.638	0.364	0	T cells	SUMO2
0	0.49496663	0.343	0.188	0	T cells	SMARCC2
0	0.49427296	0.362	0.197	0	T cells	CWC15
0	0.49198072	0.28	0.156	0	T cells	SMC1A
0	0.49172322	0.363	0.19	0	T cells	ZBTB44
0	0.48824755	0.317	0.165	0	T cells	DENND1C
0	0.48693384	0.679	0.398	0	T cells	DDX39B
0	0.484849	0.279	0.145	0	T cells	MLXIP
0	0.47841576	0.782	0.466	0	T cells	IKZF1
0	0.47680683	0.291	0.166	0	T cells	RAP2B
0	0.47624623	0.928	0.712	0	T cells	BTG1
0	0.47543328	0.417	0.217	0	T cells	BBX
0	0.47396023	0.308	0.166	0	T cells	POLR2B
0	0.47337378	0.485	0.226	0	T cells	PKM
0	0.47318065	0.273	0.155	0	T cells	BLOC1S6
0	0.47307618	0.345	0.185	0	T cells	RBM27
0	0.4729576	0.388	0.22	0	T cells	ATP5F1B
0	0.4724292	0.273	0.107	0	T cells	LRBA
0	0.46986275	0.258	0.136	0	T cells	DAXX
0	0.46889109	0.424	0.239	0	T cells	ARL6IP1
0	0.46729339	0.284	0.155	0	T cells	CSNK2A1

0	0.46560955	0.447	0.25	0	T cells	DDX46
0	0.46392316	0.447	0.253	0	T cells	AKAP9
0	0.46291682	0.265	0.141	0	T cells	SRI
0	0.46256482	0.381	0.201	0	T cells	PCGF5
0	0.45341025	0.295	0.161	0	T cells	ALKBH5
0	0.44935011	0.829	0.552	0	T cells	SON
0	0.44906493	0.456	0.257	0	T cells	FGFR1OP2
0	0.4488349	0.354	0.194	0	T cells	DAD1
0	0.44801913	0.61	0.348	0	T cells	MPHOSPH8
0	0.44722803	0.266	0.134	0	T cells	PHACTR2
0	0.44482562	0.475	0.277	0	T cells	H2AFV
0	0.44466942	0.281	0.159	0	T cells	PABPN1
0	0.44419546	0.549	0.307	0	T cells	CIRBP
0	0.44372455	0.516	0.289	0	T cells	PPP4R3B
0	0.43551271	0.359	0.198	0	T cells	PPP2R5E
0	0.43488522	0.525	0.306	0	T cells	TSPAN14
0	0.43386134	0.28	0.156	0	T cells	TBRG1
0	0.43235486	0.762	0.485	0	T cells	SF1
0	0.43208571	0.26	0.128	0	T cells	USP24
0	0.43135004	0.284	0.129	0	T cells	DNMT3A
0	0.42913472	0.281	0.159	0	T cells	DNAJA2
0	0.42882936	0.251	0.132	0	T cells	LEMD3
0	0.42855067	0.656	0.399	0	T cells	ARHGEF1
0	0.42455394	0.803	0.532	0	T cells	HNRNPU
0	0.42434841	0.483	0.261	0	T cells	RASGRP2
0	0.42282927	0.325	0.187	0	T cells	CIAO1
0	0.42275183	0.288	0.145	0	T cells	SIN3A
0	0.42265085	0.283	0.157	0	T cells	ZC3H7A
0	0.4224584	0.259	0.142	0	T cells	UBE2Z
0	0.42147	0.291	0.154	0	T cells	HECTD1
0	0.42115048	0.32	0.177	0	T cells	SEC63
0	0.41939162	0.579	0.335	0	T cells	SMARCA2
0	0.41841693	0.308	0.162	0	T cells	DCAF5
0	0.4160688	0.269	0.139	0	T cells	RNF138
0	0.4159107	0.39	0.187	0	T cells	P2RY8
0	0.41562158	0.282	0.132	0	T cells	RPRD2
0	0.41252606	0.264	0.146	0	T cells	UBQLN1
0	0.41035537	0.259	0.132	0	T cells	SUPT5H
0	0.4069311	0.729	0.476	0	T cells	BCLAF1
0	0.40678574	0.617	0.368	0	T cells	CLK1
0	0.40650271	0.583	0.349	0	T cells	ARF6
0	0.40006354	0.263	0.12	0	T cells	TCF12
0	0.39998931	0.56	0.337	0	T cells	FUS
0	0.39891374	0.285	0.132	0	T cells	GPI
0	0.39797381	0.304	0.156	0	T cells	STIM2
0	0.39717728	0.354	0.194	0	T cells	BRWD1
0	0.39204772	0.607	0.36	0	T cells	PDCD4
0	0.39172835	0.25	0.133	0	T cells	TMEM87A
0	0.38992858	0.258	0.146	0	T cells	CAPRIN1
0	0.38846594	0.277	0.162	0	T cells	HSPA4

0	0.38547407	0.357	0.199	0	T cells	ZFR
0	0.38467106	0.419	0.246	0	T cells	HNRNPUL2
0	0.38351322	0.289	0.167	0	T cells	FBXO9
0	0.38259756	0.259	0.133	0	T cells	WDR33
0	0.38154997	0.338	0.187	0	T cells	PBRM1
0	0.38094115	0.448	0.267	0	T cells	CHCHD2
0	0.38046469	0.29	0.156	0	T cells	RSU1
0	0.37859973	0.467	0.28	0	T cells	CDV3
0	0.37853422	0.361	0.209	0	T cells	SBNO1
0	0.37691951	0.555	0.338	0	T cells	SRP14
0	0.37325409	0.437	0.255	0	T cells	GOLGA4
0	0.3719194	0.267	0.161	0	T cells	PSMD7
0	0.37190575	0.399	0.23	0	T cells	ZNF655
0	0.37089166	0.954	0.781	0	T cells	ACTG1
0	0.37036099	0.292	0.163	0	T cells	ARIH2
0	0.36955397	0.351	0.193	0	T cells	LIMS1
0	0.36922638	0.824	0.576	0	T cells	PRRC2C
0	0.36804105	0.27	0.165	0	T cells	FAM204A
0	0.36803881	0.589	0.357	0	T cells	SUB1
0	0.3662443	0.265	0.145	0	T cells	FAM168B
0	0.36582307	0.334	0.202	0	T cells	SPCS2
0	0.36472135	0.365	0.174	0	T cells	NLRC5
0	0.36426581	0.535	0.313	0	T cells	GGNBP2
0	0.36375389	0.372	0.2	0	T cells	TAF15
0	0.36278899	0.277	0.148	0	T cells	MAPKAP1
0	0.36259379	0.307	0.157	0	T cells	TBC1D10C
0	0.36213098	0.559	0.306	0	T cells	FKBP5
0	0.3612243	0.265	0.141	0	T cells	SNRNP70
0	0.35974513	0.501	0.295	0	T cells	SMG1
0	0.35758422	0.389	0.226	0	T cells	UBE2I
0	0.3560345	0.286	0.171	0	T cells	MAPK1IP1L
0	0.35597491	0.32	0.18	0	T cells	CHTOP
0	0.35566677	0.449	0.268	0	T cells	ZC3H13
0	0.35292457	0.631	0.37	0	T cells	UBE2D2
0	0.35129995	0.322	0.148	0	T cells	NCK2
0	0.34591817	0.325	0.135	0	T cells	P2RY10
0	0.34181293	0.487	0.292	0	T cells	YME1L1
0	0.34056111	0.303	0.177	0	T cells	UBAP2L
0	0.33174361	0.383	0.171	0	T cells	ZNF609
0	0.32912774	0.512	0.289	0	T cells	PIM1
0	0.32790839	0.345	0.184	0	T cells	PTPRA
0	0.32735043	0.497	0.278	0	T cells	RASA2
0	0.32695254	0.599	0.366	0	T cells	PPIG
0	0.32671544	0.299	0.176	0	T cells	SECISBP2L
0	0.32123914	0.392	0.235	0	T cells	ATP5MF
0	0.32086498	0.402	0.241	0	T cells	TM9SF3
0	0.31322816	0.36	0.206	0	T cells	PTPN1
0	0.31305017	0.379	0.232	0	T cells	ARHGDIA
0	0.30833935	0.282	0.16	0	T cells	IGBP1
0	0.30699714	0.725	0.483	0	T cells	PNISR

0	0.30676787	0.327	0.199	0	T cells	MTIF3
0	0.30651088	0.425	0.249	0	T cells	DCAF7
0	0.3061268	0.295	0.176	0	T cells	PTPN2
0	0.30581636	0.574	0.356	0	T cells	SEC62
0	0.30558702	0.748	0.499	0	T cells	VAMP2
0	0.30438695	0.457	0.276	0	T cells	RBM4
0	0.30371266	0.291	0.174	0	T cells	EFR3A
0	0.30072772	0.419	0.253	0	T cells	ATF4
0	0.30024138	0.504	0.315	0	T cells	EIF5
0	0.29959578	0.252	0.144	0	T cells	KPNA6
0	0.29773266	0.36	0.216	0	T cells	SRP19
0	0.29745	0.552	0.345	0	T cells	STX16
0	0.29735166	0.425	0.221	0	T cells	EML4
0	0.29381055	0.301	0.187	0	T cells	VHL
0	0.29154386	0.451	0.281	0	T cells	SSR1
0	0.29116279	0.294	0.176	0	T cells	PTAR1
0	0.28978142	0.475	0.294	0	T cells	KLF13
0	0.28738755	0.359	0.214	0	T cells	CRYBG1
0	0.2873823	0.269	0.156	0	T cells	ENTPD4
0	0.28496542	0.635	0.412	0	T cells	PRPF38B
0	0.2844948	0.889	0.647	0	T cells	HMGB1
0	0.28033804	0.671	0.448	0	T cells	HNRNPUL1
0	0.27976165	0.492	0.295	0	T cells	SEPTIN2
0	0.27904005	0.812	0.557	0	T cells	MYL12A
0	0.27557109	0.343	0.191	0	T cells	AP2B1
0	0.27276479	0.316	0.196	0	T cells	RO60
0	0.26814323	0.539	0.339	0	T cells	EIF4H
0	0.26788579	0.485	0.302	0	T cells	ISCU
0	0.26786573	0.837	0.552	0	T cells	IL2RG
0	0.26576861	0.36	0.215	0	T cells	SUZ12
0	0.26374653	0.455	0.286	0	T cells	MAT2B
0	0.26231572	0.271	0.157	0	T cells	STK26
0	0.26143538	0.254	0.146	0	T cells	ESCO1
0	0.26006206	0.454	0.287	0	T cells	U2AF2
0	0.2592206	0.532	0.321	0	T cells	EMP3
0	0.25846216	0.396	0.215	0	T cells	ARHGEF6
0	0.25838507	0.473	0.298	0	T cells	SAP18
0	0.25779087	0.657	0.435	0	T cells	DDX6
0	0.25628767	0.689	0.444	0	T cells	BPTF
0	0.25547137	0.501	0.312	0	T cells	ZBTB7A
0	0.25499503	0.43	0.266	0	T cells	CNOT1
0	0.25135901	0.485	0.307	0	T cells	ATP6V1G1
0	0.25120713	0.362	0.204	0	T cells	CLINT1
0	0.25076377	0.419	0.258	0	T cells	PHF11
0	0.25037233	0.676	0.446	0	T cells	RBM25
6.498E-304	0.25853052	0.31	0.193	2.18E-299	T cells	MIA3
2.978E-301	0.28327731	0.258	0.155	9.988E-297	T cells	USP22
4.588E-301	0.46359255	0.25	0.153	1.539E-296	T cells	PSMB2
2.723E-295	0.25214835	0.282	0.173	9.134E-291	T cells	CWC22
3.761E-280	0.30014271	0.272	0.169	1.262E-275	T cells	CPSF2

1.948E-188	0.35238336	0.985	0.964	6.533E-184	T cells	MTRNR2L12
0	6.15030125	0.64	0.036	0	NK/CD8 T cells	GNLY
0	6.05666259	0.927	0.028	0	NK/CD8 T cells	NKG7
0	5.73571725	0.879	0.024	0	NK/CD8 T cells	PRF1
0	5.49958061	0.907	0.024	0	NK/CD8 T cells	GZMA
0	5.20457458	0.558	0.007	0	NK/CD8 T cells	FGFBP2
0	4.91202	0.758	0.038	0	NK/CD8 T cells	IL2RB
0	4.64574716	0.516	0.007	0	NK/CD8 T cells	GZMB
0	4.5758787	0.467	0.004	0	NK/CD8 T cells	GZMH
0	4.56834016	0.72	0.036	0	NK/CD8 T cells	KLRD1
0	4.55999462	0.856	0.047	0	NK/CD8 T cells	CCL5
0	4.55588679	0.505	0.013	0	NK/CD8 T cells	KLRF1
0	4.45808371	0.744	0.032	0	NK/CD8 T cells	CTSW
0	4.18364567	0.707	0.025	0	NK/CD8 T cells	SLAMF7
0	4.17023642	0.432	0.014	0	NK/CD8 T cells	SPON2
0	4.09584305	0.529	0.009	0	NK/CD8 T cells	EOMES
0	4.05748821	0.403	0.006	0	NK/CD8 T cells	TRDC
0	4.04263776	0.682	0.099	0	NK/CD8 T cells	CX3CR1
0	3.99537419	0.849	0.065	0	NK/CD8 T cells	KLRK1
0	3.97692679	0.417	0.01	0	NK/CD8 T cells	TRGC1
0	3.97686752	0.582	0.024	0	NK/CD8 T cells	TRGC2
0	3.93704472	0.374	0.011	0	NK/CD8 T cells	GZMK
0	3.85969339	0.464	0.01	0	NK/CD8 T cells	HOPX
0	3.859424	0.575	0.034	0	NK/CD8 T cells	KLRB1
0	3.8289127	0.5	0.01	0	NK/CD8 T cells	TBX21
0	3.81461059	0.445	0.003	0	NK/CD8 T cells	S1PR5
0	3.66087421	0.967	0.167	0	NK/CD8 T cells	ARL4C
0	3.65646239	0.376	0.004	0	NK/CD8 T cells	ADGRG1
0	3.59785239	0.293	0.003	0	NK/CD8 T cells	CD160
0	3.58956684	0.559	0.031	0	NK/CD8 T cells	KLRG1
0	3.56061336	0.714	0.054	0	NK/CD8 T cells	PYHIN1
0	3.50380835	0.625	0.035	0	NK/CD8 T cells	MYBL1
0	3.4749156	0.624	0.033	0	NK/CD8 T cells	TGFBR3
0	3.46930453	0.466	0.043	0	NK/CD8 T cells	CD8A
0	3.44657414	0.305	0.008	0	NK/CD8 T cells	SH2D1B
0	3.42408951	0.391	0.009	0	NK/CD8 T cells	PTGDR
0	3.40976183	0.889	0.136	0	NK/CD8 T cells	RUNX3
0	3.39326176	0.369	0.016	0	NK/CD8 T cells	NCR1
0	3.34547271	0.442	0.01	0	NK/CD8 T cells	F2R
0	3.30713622	0.3	0.004	0	NK/CD8 T cells	PRSS23
0	3.24639045	0.398	0.011	0	NK/CD8 T cells	C1orf21
0	3.2313007	0.343	0.014	0	NK/CD8 T cells	DUSP2
0	3.23104103	0.46	0.041	0	NK/CD8 T cells	CEP78
0	3.15963771	0.63	0.047	0	NK/CD8 T cells	SAMD3
0	3.11617532	0.275	0.004	0	NK/CD8 T cells	FCRL6
0	3.10270246	0.517	0.042	0	NK/CD8 T cells	SH2D1A
0	3.08035813	0.4	0.019	0	NK/CD8 T cells	SH2D2A
0	2.94902739	0.405	0.023	0	NK/CD8 T cells	SYTL2
0	2.91147415	0.316	0.019	0	NK/CD8 T cells	TIGIT
0	2.90405172	0.832	0.165	0	NK/CD8 T cells	SPN

0	2.87907902	0.523	0.063	0	NK/CD8 T cells	TRG-AS1
0	2.80100622	0.524	0.068	0	NK/CD8 T cells	CD7
0	2.79321795	0.539	0.03	0	NK/CD8 T cells	CCL4
0	2.74526731	0.744	0.142	0	NK/CD8 T cells	CD2
0	2.73595023	0.775	0.122	0	NK/CD8 T cells	SLFN12L
0	2.70731903	0.581	0.084	0	NK/CD8 T cells	APOBEC3G
0	2.70418293	0.754	0.185	0	NK/CD8 T cells	IL32
0	2.69930677	0.311	0.016	0	NK/CD8 T cells	GZMM
0	2.67776457	0.808	0.141	0	NK/CD8 T cells	IKZF3
0	2.66608913	0.296	0.017	0	NK/CD8 T cells	LINC01871
0	2.64802206	0.744	0.166	0	NK/CD8 T cells	ID2
0	2.64204795	0.385	0.027	0	NK/CD8 T cells	C12orf75
0	2.62795573	0.335	0.024	0	NK/CD8 T cells	FCRL3
0	2.62665039	0.359	0.043	0	NK/CD8 T cells	GPR171
0	2.62615783	0.606	0.104	0	NK/CD8 T cells	XBP1
0	2.61975457	0.311	0.023	0	NK/CD8 T cells	ENPP4
0	2.60771621	0.527	0.074	0	NK/CD8 T cells	LYAR
0	2.60506013	0.487	0.067	0	NK/CD8 T cells	FUT11
0	2.59499638	0.752	0.117	0	NK/CD8 T cells	PTPN4
0	2.53715089	0.558	0.073	0	NK/CD8 T cells	SLAMF6
0	2.52647273	0.537	0.065	0	NK/CD8 T cells	STOM
0	2.49659643	0.402	0.043	0	NK/CD8 T cells	ADRB2
0	2.47830291	0.79	0.168	0	NK/CD8 T cells	CD99
0	2.47352639	0.456	0.064	0	NK/CD8 T cells	CD81
0	2.46625423	0.797	0.195	0	NK/CD8 T cells	HCST
0	2.46166427	0.33	0.031	0	NK/CD8 T cells	SBK1
0	2.44704019	0.876	0.194	0	NK/CD8 T cells	SLFN5
0	2.44475502	0.622	0.123	0	NK/CD8 T cells	APOBEC3C
0	2.43797515	0.326	0.045	0	NK/CD8 T cells	CXXC5
0	2.43586636	0.333	0.04	0	NK/CD8 T cells	PTGER2
0	2.43313005	0.537	0.081	0	NK/CD8 T cells	GPRIN3
0	2.43116541	0.545	0.07	0	NK/CD8 T cells	NFATC2
0	2.41319641	0.387	0.062	0	NK/CD8 T cells	ZBTB16
0	2.38589439	0.469	0.064	0	NK/CD8 T cells	OPTN
0	2.37422399	0.855	0.157	0	NK/CD8 T cells	CST7
0	2.33182647	0.679	0.165	0	NK/CD8 T cells	PLAC8
0	2.331444	0.358	0.052	0	NK/CD8 T cells	CMC1
0	2.33009975	0.489	0.098	0	NK/CD8 T cells	GNPTAB
0	2.30557741	0.625	0.108	0	NK/CD8 T cells	S1PR1
0	2.29725518	0.506	0.088	0	NK/CD8 T cells	PLAAT4
0	2.27403173	0.326	0.036	0	NK/CD8 T cells	CD69
0	2.25629445	0.333	0.043	0	NK/CD8 T cells	GK5
0	2.25402028	0.264	0.028	0	NK/CD8 T cells	ZNF600
0	2.25023797	0.429	0.05	0	NK/CD8 T cells	PRR5L
0	2.22613432	0.305	0.033	0	NK/CD8 T cells	TSEN54
0	2.22401013	0.585	0.114	0	NK/CD8 T cells	DENND2D
0	2.21990714	0.592	0.116	0	NK/CD8 T cells	RAB29
0	2.19774611	0.663	0.111	0	NK/CD8 T cells	RORA
0	2.19696408	0.331	0.035	0	NK/CD8 T cells	CHST12
0	2.14309566	0.262	0.049	0	NK/CD8 T cells	CHST2

0	2.13255185	0.427	0.079	0	NK/CD8 T cells	ARPC5L
0	2.11864073	0.585	0.126	0	NK/CD8 T cells	CCND2
0	2.11848901	0.943	0.236	0	NK/CD8 T cells	AHNAK
0	2.11015464	0.742	0.17	0	NK/CD8 T cells	CD3E
0	2.09559172	0.326	0.05	0	NK/CD8 T cells	MEX3C
0	2.09548139	0.528	0.135	0	NK/CD8 T cells	CD3G
0	2.0892343	0.358	0.063	0	NK/CD8 T cells	DDIT4
0	2.07754606	0.598	0.136	0	NK/CD8 T cells	ANXA6
0	2.07475402	0.726	0.139	0	NK/CD8 T cells	CD96
0	2.07010747	0.769	0.236	0	NK/CD8 T cells	CALR
0	2.06939981	0.667	0.153	0	NK/CD8 T cells	BCL9L
0	2.0543711	0.334	0.05	0	NK/CD8 T cells	USP28
0	2.04244472	0.694	0.151	0	NK/CD8 T cells	PRKACB
0	2.03119051	0.408	0.061	0	NK/CD8 T cells	CD226
0	2.02864441	0.899	0.254	0	NK/CD8 T cells	TLE5
0	2.02832503	0.443	0.095	0	NK/CD8 T cells	GIMAP6
0	2.0273831	0.411	0.063	0	NK/CD8 T cells	NLRC3
0	2.02330363	0.623	0.153	0	NK/CD8 T cells	YWHAQ
0	2.01986072	0.414	0.077	0	NK/CD8 T cells	PLEKHA1
0	2.00640042	0.353	0.054	0	NK/CD8 T cells	GATA3
0	1.99651066	0.594	0.144	0	NK/CD8 T cells	SSBP3
0	1.99062107	0.289	0.036	0	NK/CD8 T cells	SLA2
0	1.96890592	0.868	0.244	0	NK/CD8 T cells	FYN
0	1.96700827	0.945	0.247	0	NK/CD8 T cells	ETS1
0	1.96678012	0.338	0.041	0	NK/CD8 T cells	IKZF2
0	1.95948988	0.439	0.06	0	NK/CD8 T cells	MAF
0	1.94835475	0.527	0.115	0	NK/CD8 T cells	RNF125
0	1.94716774	0.643	0.133	0	NK/CD8 T cells	RASGRP1
0	1.93684377	0.361	0.07	0	NK/CD8 T cells	TSHZ1
0	1.9332563	0.442	0.106	0	NK/CD8 T cells	CD3D
0	1.93056067	0.534	0.119	0	NK/CD8 T cells	DYRK2
0	1.92069125	0.264	0.029	0	NK/CD8 T cells	SLFN13
0	1.91702219	0.397	0.075	0	NK/CD8 T cells	STK39
0	1.91671587	0.634	0.115	0	NK/CD8 T cells	CD247
0	1.91426685	0.81	0.194	0	NK/CD8 T cells	SLC38A1
0	1.91170502	0.595	0.143	0	NK/CD8 T cells	ARAP2
0	1.90572257	0.742	0.204	0	NK/CD8 T cells	MLLT6
0	1.90332035	0.701	0.157	0	NK/CD8 T cells	LINC00861
0	1.89854123	0.566	0.22	0	NK/CD8 T cells	IL7R
0	1.89686325	0.389	0.067	0	NK/CD8 T cells	PARP15
0	1.89476646	0.286	0.031	0	NK/CD8 T cells	NCALD
0	1.89308146	0.852	0.191	0	NK/CD8 T cells	TRBC2
0	1.88157888	0.469	0.066	0	NK/CD8 T cells	STAT4
0	1.87072409	0.72	0.206	0	NK/CD8 T cells	NUCKS1
0	1.8696053	0.406	0.082	0	NK/CD8 T cells	ITGB7
0	1.8625497	0.526	0.136	0	NK/CD8 T cells	CAPN2
0	1.86191336	0.252	0.035	0	NK/CD8 T cells	MIAT
0	1.85979558	0.328	0.042	0	NK/CD8 T cells	AUTS2
0	1.84893642	0.725	0.19	0	NK/CD8 T cells	SPOCK2
0	1.84868544	0.354	0.08	0	NK/CD8 T cells	CFAP97

0	1.82728277	0.613	0.123	0	NK/CD8 T cells	TRBC1
0	1.82719898	0.476	0.117	0	NK/CD8 T cells	ZBTB38
0	1.82532212	0.922	0.249	0	NK/CD8 T cells	GIMAP7
0	1.82155197	0.548	0.146	0	NK/CD8 T cells	IL10RA
0	1.81552597	0.941	0.316	0	NK/CD8 T cells	PPIA
0	1.81166375	0.314	0.06	0	NK/CD8 T cells	CDC25B
0	1.79720319	0.739	0.246	0	NK/CD8 T cells	PIK3R1
0	1.79523359	0.725	0.23	0	NK/CD8 T cells	PDIA3
0	1.79436031	0.299	0.066	0	NK/CD8 T cells	ZNF22
0	1.7907206	0.828	0.278	0	NK/CD8 T cells	MACF1
0	1.78969369	0.579	0.118	0	NK/CD8 T cells	LCK
0	1.78594459	0.362	0.074	0	NK/CD8 T cells	ITM2A
0	1.78371803	0.261	0.051	0	NK/CD8 T cells	SSBP4
0	1.77999161	0.346	0.079	0	NK/CD8 T cells	LMAN1
0	1.77836276	0.681	0.158	0	NK/CD8 T cells	LBH
0	1.7643209	0.309	0.053	0	NK/CD8 T cells	FKBP11
0	1.7586866	0.742	0.24	0	NK/CD8 T cells	UHMK1
0	1.75271188	0.448	0.091	0	NK/CD8 T cells	TMEM181
0	1.74739563	0.33	0.06	0	NK/CD8 T cells	NPC1
0	1.74457741	0.291	0.058	0	NK/CD8 T cells	C19orf12
0	1.73913908	0.506	0.095	0	NK/CD8 T cells	CBLB
0	1.7381522	0.425	0.105	0	NK/CD8 T cells	CYB5B
0	1.72659905	0.59	0.174	0	NK/CD8 T cells	TRAC
0	1.72557915	0.474	0.135	0	NK/CD8 T cells	GIMAP1
0	1.7240182	0.438	0.099	0	NK/CD8 T cells	ZMYND11
0	1.72305021	0.575	0.146	0	NK/CD8 T cells	GIMAP5
0	1.72240903	0.503	0.15	0	NK/CD8 T cells	NDUFB2
0	1.72127364	0.657	0.202	0	NK/CD8 T cells	TES
0	1.71613499	0.255	0.023	0	NK/CD8 T cells	ADGRG5
0	1.71422175	0.626	0.151	0	NK/CD8 T cells	PCED1B-AS1
0	1.71225413	1	0.899	0	NK/CD8 T cells	MT-CO1
0	1.70054862	0.35	0.083	0	NK/CD8 T cells	AC092821.3
0	1.69313389	0.268	0.048	0	NK/CD8 T cells	OSBPL3
0	1.69250823	0.392	0.095	0	NK/CD8 T cells	AL365361.1
0	1.68835139	0.25	0.048	0	NK/CD8 T cells	BIN1
0	1.67426059	0.903	0.339	0	NK/CD8 T cells	HSPA8
0	1.67280427	0.998	0.748	0	NK/CD8 T cells	MT-ND5
0	1.65935919	0.609	0.173	0	NK/CD8 T cells	TPST2
0	1.65880144	0.45	0.121	0	NK/CD8 T cells	ZNF91
0	1.65724509	0.732	0.176	0	NK/CD8 T cells	PRKCH
0	1.65626545	0.333	0.077	0	NK/CD8 T cells	LAIR1
0	1.65315222	0.53	0.158	0	NK/CD8 T cells	RAP2B
0	1.65009319	0.58	0.178	0	NK/CD8 T cells	PA2G4
0	1.64088887	0.392	0.098	0	NK/CD8 T cells	ZBTB4
0	1.64002619	0.581	0.176	0	NK/CD8 T cells	GTF3A
0	1.6281217	0.425	0.133	0	NK/CD8 T cells	CLDND1
0	1.62725434	0.367	0.088	0	NK/CD8 T cells	LIME1
0	1.62177735	0.633	0.183	0	NK/CD8 T cells	ITGB1
0	1.60925042	0.444	0.102	0	NK/CD8 T cells	LAT
0	1.60814184	0.268	0.046	0	NK/CD8 T cells	PAFAH2

0	1.60769391	0.826	0.284	0	NK/CD8 T cells	NCL
0	1.60600052	0.796	0.272	0	NK/CD8 T cells	UCP2
0	1.60567625	0.408	0.094	0	NK/CD8 T cells	TRIB2
0	1.60267166	0.313	0.054	0	NK/CD8 T cells	ZNF831
0	1.59509159	0.818	0.273	0	NK/CD8 T cells	HSP90B1
0	1.5931685	0.296	0.077	0	NK/CD8 T cells	MBNL3
0	1.59106336	0.288	0.08	0	NK/CD8 T cells	ARL14EP
0	1.58579648	0.823	0.269	0	NK/CD8 T cells	HSP90AB1
0	1.57237644	0.324	0.084	0	NK/CD8 T cells	SRSF8
0	1.57011712	0.993	0.543	0	NK/CD8 T cells	MT-ATP8
0	1.57002923	0.33	0.091	0	NK/CD8 T cells	ADH5
0	1.56960154	0.565	0.16	0	NK/CD8 T cells	CCDC88C
0	1.56663998	0.992	0.624	0	NK/CD8 T cells	CALM1
0	1.5640031	0.666	0.204	0	NK/CD8 T cells	FLNA
0	1.56365824	0.568	0.131	0	NK/CD8 T cells	KLF12
0	1.5525681	0.454	0.137	0	NK/CD8 T cells	SCP2
0	1.55035343	0.848	0.277	0	NK/CD8 T cells	HNRNPA1
0	1.54529694	0.999	0.475	0	NK/CD8 T cells	RPS27
0	1.54513333	0.522	0.114	0	NK/CD8 T cells	TC2N
0	1.54125952	0.488	0.145	0	NK/CD8 T cells	BUB3
0	1.53842804	0.559	0.149	0	NK/CD8 T cells	ARHGEF3
0	1.5299635	0.282	0.081	0	NK/CD8 T cells	CYCS
0	1.52620933	0.589	0.191	0	NK/CD8 T cells	G3BP1
0	1.52441557	0.415	0.131	0	NK/CD8 T cells	EPRS
0	1.52415775	0.478	0.155	0	NK/CD8 T cells	BANF1
0	1.52182287	0.29	0.064	0	NK/CD8 T cells	SIGIRR
0	1.52163174	0.486	0.145	0	NK/CD8 T cells	URI1
0	1.52154499	0.307	0.07	0	NK/CD8 T cells	RFTN1
0	1.5201214	0.463	0.144	0	NK/CD8 T cells	PDIA6
0	1.51738071	0.976	0.343	0	NK/CD8 T cells	RPL23A
0	1.51694529	0.462	0.145	0	NK/CD8 T cells	DNMT1
0	1.51494293	0.274	0.07	0	NK/CD8 T cells	SEC11C
0	1.51421287	0.471	0.108	0	NK/CD8 T cells	PTPN22
0	1.51392062	0.26	0.059	0	NK/CD8 T cells	YARS
0	1.51257732	0.379	0.091	0	NK/CD8 T cells	SEPTIN1
0	1.51040998	0.36	0.112	0	NK/CD8 T cells	SDAD1
0	1.50945682	0.418	0.124	0	NK/CD8 T cells	ELK4
0	1.50912714	0.936	0.328	0	NK/CD8 T cells	RPS19
0	1.50856723	0.287	0.084	0	NK/CD8 T cells	PNP
0	1.50663481	0.392	0.128	0	NK/CD8 T cells	EIF3G
0	1.50644301	0.374	0.115	0	NK/CD8 T cells	MESD
0	1.50547658	0.322	0.062	0	NK/CD8 T cells	TFDP2
0	1.50309621	0.396	0.119	0	NK/CD8 T cells	SSR4
0	1.50282455	0.498	0.154	0	NK/CD8 T cells	ERAP2
0	1.5017128	0.668	0.195	0	NK/CD8 T cells	ATP2B4
0	1.49925814	0.658	0.215	0	NK/CD8 T cells	COX6C
0	1.49813608	0.57	0.186	0	NK/CD8 T cells	SNRPD2
0	1.49097301	0.339	0.086	0	NK/CD8 T cells	ORMDL3
0	1.49008505	0.287	0.065	0	NK/CD8 T cells	CDK6
0	1.48879945	0.278	0.083	0	NK/CD8 T cells	IMPAD1

0	1.48578262	0.396	0.095	0	NK/CD8 T cells	TXK
0	1.48524006	0.987	0.35	0	NK/CD8 T cells	RPL3
0	1.48351209	0.321	0.073	0	NK/CD8 T cells	RHOF
0	1.48056984	0.327	0.101	0	NK/CD8 T cells	NOLC1
0	1.47931733	0.337	0.099	0	NK/CD8 T cells	USP14
0	1.47831511	0.85	0.362	0	NK/CD8 T cells	SUN2
0	1.47416589	0.618	0.215	0	NK/CD8 T cells	GPR65
0	1.4721885	0.355	0.111	0	NK/CD8 T cells	CCT2
0	1.47175096	0.275	0.067	0	NK/CD8 T cells	HCG18
0	1.46547315	0.361	0.111	0	NK/CD8 T cells	FBL
0	1.46217999	0.273	0.067	0	NK/CD8 T cells	USP11
0	1.46195565	0.367	0.115	0	NK/CD8 T cells	IMP3
0	1.46089507	0.529	0.173	0	NK/CD8 T cells	EID1
0	1.45910673	0.85	0.273	0	NK/CD8 T cells	RPSA
0	1.45670189	0.315	0.097	0	NK/CD8 T cells	SSRP1
0	1.45578783	0.391	0.116	0	NK/CD8 T cells	PARP1
0	1.45376923	0.251	0.05	0	NK/CD8 T cells	SIDT1
0	1.45092454	0.327	0.081	0	NK/CD8 T cells	ODF2L
0	1.45065428	0.26	0.076	0	NK/CD8 T cells	TOMM5
0	1.44855547	1	0.905	0	NK/CD8 T cells	MT-ATP6
0	1.44851385	0.598	0.208	0	NK/CD8 T cells	SYNCRIP
0	1.44757057	0.698	0.24	0	NK/CD8 T cells	APOL6
0	1.44645926	0.355	0.079	0	NK/CD8 T cells	IL18RAP
0	1.4437216	0.503	0.152	0	NK/CD8 T cells	MAPRE2
0	1.44228694	0.348	0.095	0	NK/CD8 T cells	MT2A
0	1.44175595	0.274	0.083	0	NK/CD8 T cells	GLO1
0	1.44109861	0.419	0.125	0	NK/CD8 T cells	CDK2AP2
0	1.43586108	0.696	0.238	0	NK/CD8 T cells	KMT2A
0	1.43447229	0.274	0.078	0	NK/CD8 T cells	NR1D2
0	1.43426359	0.694	0.224	0	NK/CD8 T cells	CLEC2D
0	1.43410273	0.294	0.089	0	NK/CD8 T cells	ACP1
0	1.43390305	0.503	0.168	0	NK/CD8 T cells	ATXN7L3B
0	1.43154316	0.352	0.101	0	NK/CD8 T cells	RBBP7
0	1.43146448	0.4	0.117	0	NK/CD8 T cells	PRKX
0	1.43140753	0.708	0.267	0	NK/CD8 T cells	UBB
0	1.42959452	0.83	0.276	0	NK/CD8 T cells	RPL35
0	1.42958125	0.421	0.139	0	NK/CD8 T cells	RPL7L1
0	1.42897937	0.324	0.102	0	NK/CD8 T cells	IL27RA
0	1.42830289	0.258	0.058	0	NK/CD8 T cells	TRAF5
0	1.42194946	0.348	0.105	0	NK/CD8 T cells	GSAP
0	1.42004443	0.26	0.058	0	NK/CD8 T cells	LPIN1
0	1.41804255	0.646	0.239	0	NK/CD8 T cells	TMED10
0	1.4138187	0.856	0.344	0	NK/CD8 T cells	PIP4K2A
0	1.41232696	0.871	0.311	0	NK/CD8 T cells	RPL7A
0	1.41118515	0.287	0.085	0	NK/CD8 T cells	PRNP
0	1.4101089	0.576	0.172	0	NK/CD8 T cells	PRDM1
0	1.40940855	0.369	0.117	0	NK/CD8 T cells	NOP56
0	1.40764603	0.623	0.218	0	NK/CD8 T cells	RAN
0	1.40606416	0.386	0.107	0	NK/CD8 T cells	PPM1K
0	1.40280408	0.823	0.304	0	NK/CD8 T cells	CD52

0	1.4015667	0.796	0.351	0	NK/CD8 T cells	RPS26
0	1.40143716	0.318	0.104	0	NK/CD8 T cells	RAD50
0	1.39712943	0.453	0.148	0	NK/CD8 T cells	PEBP1
0	1.39578917	0.355	0.112	0	NK/CD8 T cells	RECQL
0	1.39577093	0.499	0.174	0	NK/CD8 T cells	SSB
0	1.39520426	0.263	0.085	0	NK/CD8 T cells	HMGN3
0	1.39359897	0.258	0.07	0	NK/CD8 T cells	TCOF1
0	1.39318123	0.496	0.137	0	NK/CD8 T cells	RASSF1
0	1.39132886	0.343	0.106	0	NK/CD8 T cells	FYTTD1
0	1.3892067	0.373	0.121	0	NK/CD8 T cells	HSPA9
0	1.38570988	0.943	0.37	0	NK/CD8 T cells	RPS3
0	1.38417691	0.337	0.096	0	NK/CD8 T cells	DSTN
0	1.38177472	0.421	0.133	0	NK/CD8 T cells	PHACTR2
0	1.37978559	0.394	0.134	0	NK/CD8 T cells	RAD23A
0	1.37878117	0.297	0.097	0	NK/CD8 T cells	ABCE1
0	1.37777221	0.31	0.099	0	NK/CD8 T cells	APOL3
0	1.37740395	0.994	0.429	0	NK/CD8 T cells	RPL6
0	1.37703962	0.297	0.093	0	NK/CD8 T cells	CUTA
0	1.37559619	0.761	0.259	0	NK/CD8 T cells	ITGA4
0	1.37389411	0.369	0.114	0	NK/CD8 T cells	PURA
0	1.3731266	1	0.594	0	NK/CD8 T cells	EEF1A1
0	1.37227022	0.356	0.121	0	NK/CD8 T cells	MDH1
0	1.3714779	0.542	0.184	0	NK/CD8 T cells	PPDPF
0	1.36964416	0.39	0.135	0	NK/CD8 T cells	PYURF
0	1.3683693	0.458	0.159	0	NK/CD8 T cells	EIF5B
0	1.36800361	0.612	0.18	0	NK/CD8 T cells	EVL
0	1.36760545	0.997	0.522	0	NK/CD8 T cells	PTMA
0	1.36739489	0.986	0.64	0	NK/CD8 T cells	MT-ND4L
0	1.36656183	0.314	0.088	0	NK/CD8 T cells	SLC16A7
0	1.36390883	0.352	0.076	0	NK/CD8 T cells	PRKCQ
0	1.35419258	0.77	0.314	0	NK/CD8 T cells	HNRNPA3
0	1.35197301	0.998	0.509	0	NK/CD8 T cells	RPS27A
0	1.34607706	0.992	0.426	0	NK/CD8 T cells	RPL26
0	1.34555759	0.685	0.264	0	NK/CD8 T cells	LNPEP
0	1.34488368	0.34	0.097	0	NK/CD8 T cells	NCOA7
0	1.34250652	0.443	0.142	0	NK/CD8 T cells	TRIM14
0	1.33800117	0.68	0.252	0	NK/CD8 T cells	TRAM1
0	1.33732145	0.926	0.464	0	NK/CD8 T cells	HSP90AA1
0	1.33645839	0.327	0.11	0	NK/CD8 T cells	HSPH1
0	1.33615233	0.996	0.514	0	NK/CD8 T cells	RPL27A
0	1.33258685	0.377	0.122	0	NK/CD8 T cells	MTA2
0	1.33199164	0.308	0.081	0	NK/CD8 T cells	LY9
0	1.33105389	0.641	0.24	0	NK/CD8 T cells	HNRNPR
0	1.33078243	0.993	0.496	0	NK/CD8 T cells	RPL17
0	1.33002546	1	0.946	0	NK/CD8 T cells	MT-CO2
0	1.32949558	0.963	0.332	0	NK/CD8 T cells	RPS4X
0	1.32817197	0.274	0.085	0	NK/CD8 T cells	ATL3
0	1.32713325	0.351	0.113	0	NK/CD8 T cells	UPF3A
0	1.32578299	0.271	0.078	0	NK/CD8 T cells	UCHL5
0	1.32352732	0.33	0.107	0	NK/CD8 T cells	HTATSF1

0	1.32325011	0.346	0.118	0	NK/CD8 T cells	STARD7
0	1.32290494	0.491	0.171	0	NK/CD8 T cells	SOD1
0	1.32181044	0.963	0.319	0	NK/CD8 T cells	RPL10A
0	1.3217024	0.712	0.276	0	NK/CD8 T cells	CNOT6L
0	1.32106255	0.293	0.101	0	NK/CD8 T cells	MPHOSPH10
0	1.32064215	0.353	0.12	0	NK/CD8 T cells	RWDD1
0	1.31962154	0.495	0.175	0	NK/CD8 T cells	SMARCE1
0	1.31959943	0.703	0.27	0	NK/CD8 T cells	SERBP1
0	1.31845576	0.527	0.179	0	NK/CD8 T cells	EZR
0	1.3176857	0.263	0.084	0	NK/CD8 T cells	PANK3
0	1.3166002	0.333	0.081	0	NK/CD8 T cells	DNAJC1
0	1.3147015	0.265	0.077	0	NK/CD8 T cells	KRR1
0	1.31162587	0.577	0.206	0	NK/CD8 T cells	DDX24
0	1.31029278	0.762	0.264	0	NK/CD8 T cells	RPLP0
0	1.30998064	0.993	0.416	0	NK/CD8 T cells	RPL37A
0	1.30630831	0.472	0.167	0	NK/CD8 T cells	CMPK1
0	1.30256298	0.991	0.458	0	NK/CD8 T cells	RPS15A
0	1.30254289	0.294	0.1	0	NK/CD8 T cells	NUS1
0	1.30041181	0.983	0.405	0	NK/CD8 T cells	RPL14
0	1.29933198	0.353	0.101	0	NK/CD8 T cells	ESYT1
0	1.29903172	0.378	0.132	0	NK/CD8 T cells	RFC1
0	1.2970838	0.95	0.381	0	NK/CD8 T cells	RPS7
0	1.29302949	0.335	0.114	0	NK/CD8 T cells	SNX5
0	1.29102919	0.394	0.133	0	NK/CD8 T cells	UBE2G2
0	1.29018437	0.327	0.114	0	NK/CD8 T cells	CEBPZ
0	1.28653929	0.272	0.068	0	NK/CD8 T cells	GOLGA8B
0	1.28588577	0.53	0.187	0	NK/CD8 T cells	LPXN
0	1.28527287	0.515	0.182	0	NK/CD8 T cells	RPN2
0	1.28424009	0.472	0.149	0	NK/CD8 T cells	ESYT2
0	1.28377645	0.353	0.118	0	NK/CD8 T cells	RDH11
0	1.28360104	0.979	0.376	0	NK/CD8 T cells	RPL10
0	1.28345745	0.989	0.391	0	NK/CD8 T cells	RPS18
0	1.2806707	0.303	0.105	0	NK/CD8 T cells	HSPE1
0	1.28031582	0.77	0.313	0	NK/CD8 T cells	HNRNPDL
0	1.28015651	0.437	0.131	0	NK/CD8 T cells	GBP4
0	1.27972797	0.389	0.133	0	NK/CD8 T cells	NOP58
0	1.27906039	0.294	0.097	0	NK/CD8 T cells	ZMAT3
0	1.2775125	0.434	0.156	0	NK/CD8 T cells	ATP5MC3
0	1.27672594	0.407	0.123	0	NK/CD8 T cells	PCSK7
0	1.27390898	0.324	0.107	0	NK/CD8 T cells	TTC37
0	1.27259986	0.997	0.508	0	NK/CD8 T cells	RPS24
0	1.27073972	0.299	0.103	0	NK/CD8 T cells	DNAJC21
0	1.2683088	0.467	0.166	0	NK/CD8 T cells	ILF3
0	1.2672476	0.712	0.239	0	NK/CD8 T cells	KIF2A
0	1.26594728	0.485	0.178	0	NK/CD8 T cells	SNHG6
0	1.26584876	0.417	0.153	0	NK/CD8 T cells	DDX18
0	1.26478632	0.413	0.152	0	NK/CD8 T cells	EIF3I
0	1.26265906	0.397	0.137	0	NK/CD8 T cells	ABCF1
0	1.26020655	0.416	0.143	0	NK/CD8 T cells	BDP1
0	1.25873691	0.609	0.136	0	NK/CD8 T cells	SKAP1

0	1.25818874	0.947	0.351	0	NK/CD8 T cells	RPL15
0	1.25509655	0.294	0.091	0	NK/CD8 T cells	MDN1
0	1.25478563	0.312	0.108	0	NK/CD8 T cells	SEM1
0	1.25377058	0.443	0.163	0	NK/CD8 T cells	SSBP1
0	1.25338589	0.581	0.22	0	NK/CD8 T cells	UBE2V1
0	1.25035456	0.371	0.108	0	NK/CD8 T cells	LPCAT1
0	1.24901672	0.541	0.186	0	NK/CD8 T cells	VAMP8
0	1.24467645	1	0.92	0	NK/CD8 T cells	MT-CYB
0	1.2432278	0.955	0.359	0	NK/CD8 T cells	RPS21
0	1.24183517	0.29	0.098	0	NK/CD8 T cells	MLEC
0	1.24112071	0.988	0.447	0	NK/CD8 T cells	RPL13A
0	1.23953002	0.748	0.317	0	NK/CD8 T cells	BTN3A2
0	1.23886718	0.365	0.128	0	NK/CD8 T cells	SFT2D2
0	1.23779396	0.268	0.088	0	NK/CD8 T cells	NOL11
0	1.23754805	0.307	0.109	0	NK/CD8 T cells	POLE3
0	1.23754501	0.775	0.306	0	NK/CD8 T cells	CANX
0	1.23719695	0.506	0.17	0	NK/CD8 T cells	SPTAN1
0	1.23571438	0.412	0.152	0	NK/CD8 T cells	NME2
0	1.23558555	0.461	0.172	0	NK/CD8 T cells	SRP72
0	1.233918	0.975	0.375	0	NK/CD8 T cells	RPS17
0	1.23363231	0.528	0.19	0	NK/CD8 T cells	NOP53
0	1.23361414	0.346	0.128	0	NK/CD8 T cells	METAP2
0	1.23276107	0.999	0.548	0	NK/CD8 T cells	RPL21
0	1.23230602	0.607	0.231	0	NK/CD8 T cells	SRSF7
0	1.23198521	0.348	0.122	0	NK/CD8 T cells	SEC61G
0	1.23113889	0.978	0.362	0	NK/CD8 T cells	RPS12
0	1.23098685	0.277	0.077	0	NK/CD8 T cells	JADE2
0	1.2303882	0.588	0.235	0	NK/CD8 T cells	TUBB
0	1.22998544	0.384	0.116	0	NK/CD8 T cells	OFD1
0	1.22909311	0.838	0.309	0	NK/CD8 T cells	RPL24
0	1.22882751	0.953	0.351	0	NK/CD8 T cells	RPL36A
0	1.22843919	0.555	0.194	0	NK/CD8 T cells	GLS
0	1.22701944	0.486	0.181	0	NK/CD8 T cells	KRTCAP2
0	1.22645812	0.286	0.079	0	NK/CD8 T cells	VPS13A
0	1.22318308	0.999	0.568	0	NK/CD8 T cells	RPS29
0	1.22186048	0.303	0.105	0	NK/CD8 T cells	ATP1B3
0	1.22159231	0.477	0.151	0	NK/CD8 T cells	CCSER2
0	1.21946807	0.288	0.107	0	NK/CD8 T cells	TMEM173
0	1.21835777	0.809	0.347	0	NK/CD8 T cells	ATM
0	1.21778157	0.303	0.105	0	NK/CD8 T cells	ZNF146
0	1.21729923	0.883	0.37	0	NK/CD8 T cells	EIF4B
0	1.21678247	0.981	0.351	0	NK/CD8 T cells	RPL5
0	1.2153204	0.252	0.089	0	NK/CD8 T cells	HCFC1
0	1.21372294	0.33	0.12	0	NK/CD8 T cells	CCT6A
0	1.21299874	0.43	0.153	0	NK/CD8 T cells	TPP2
0	1.2121988	0.289	0.085	0	NK/CD8 T cells	UBE2Q2
0	1.2120886	0.429	0.152	0	NK/CD8 T cells	UBE3A
0	1.21132223	0.271	0.082	0	NK/CD8 T cells	UBE2F
0	1.20963311	0.257	0.089	0	NK/CD8 T cells	ILF2
0	1.20955849	0.353	0.123	0	NK/CD8 T cells	MEAF6

0	1.20726828	0.338	0.116	0	NK/CD8 T cells	THUMPD1
0	1.20723379	1	0.949	0	NK/CD8 T cells	MT-CO3
0	1.20634671	0.282	0.094	0	NK/CD8 T cells	CDC16
0	1.20599313	0.467	0.173	0	NK/CD8 T cells	TASOR
0	1.20588968	0.257	0.094	0	NK/CD8 T cells	GNL3
0	1.20467366	0.45	0.17	0	NK/CD8 T cells	NDUFB8
0	1.20402792	0.996	0.426	0	NK/CD8 T cells	RPS20
0	1.20336143	0.296	0.102	0	NK/CD8 T cells	CHORDC1
0	1.2033103	0.527	0.192	0	NK/CD8 T cells	ZBTB44
0	1.20294054	0.286	0.072	0	NK/CD8 T cells	SP4
0	1.20258508	0.953	0.375	0	NK/CD8 T cells	RPS2
0	1.20208961	0.272	0.082	0	NK/CD8 T cells	SLC4A7
0	1.20188411	0.391	0.133	0	NK/CD8 T cells	MDFIC
0	1.20163813	0.262	0.09	0	NK/CD8 T cells	UFC1
0	1.19945681	0.268	0.092	0	NK/CD8 T cells	SLC25A36
0	1.19282978	0.863	0.296	0	NK/CD8 T cells	NPM1
0	1.19193855	0.584	0.198	0	NK/CD8 T cells	RASA3
0	1.19092526	0.409	0.153	0	NK/CD8 T cells	CCT4
0	1.19047561	0.962	0.4	0	NK/CD8 T cells	RPS14
0	1.18971121	0.259	0.095	0	NK/CD8 T cells	RCC2
0	1.18959443	0.308	0.072	0	NK/CD8 T cells	IL18R1
0	1.18721254	0.314	0.108	0	NK/CD8 T cells	NDUFA5
0	1.18713414	0.301	0.107	0	NK/CD8 T cells	HNRNPAB
0	1.18701307	1	0.962	0	NK/CD8 T cells	MT-ND4
0	1.18570066	0.281	0.111	0	NK/CD8 T cells	CD84
0	1.18523129	0.996	0.462	0	NK/CD8 T cells	RPL31
0	1.18497186	0.961	0.388	0	NK/CD8 T cells	RPL27
0	1.18459337	0.428	0.164	0	NK/CD8 T cells	UFM1
0	1.17763598	0.28	0.094	0	NK/CD8 T cells	DAZAP1
0	1.1768864	0.263	0.09	0	NK/CD8 T cells	GORASP2
0	1.17586825	0.972	0.355	0	NK/CD8 T cells	RPL13
0	1.17565798	0.271	0.091	0	NK/CD8 T cells	BLOC1S4
0	1.17547982	0.788	0.358	0	NK/CD8 T cells	GNG2
0	1.17501351	0.602	0.23	0	NK/CD8 T cells	M6PR
0	1.17402298	0.274	0.096	0	NK/CD8 T cells	GPR183
0	1.1737399	0.58	0.227	0	NK/CD8 T cells	KTN1
0	1.17290872	0.602	0.246	0	NK/CD8 T cells	SRSF3
0	1.17248853	0.876	0.31	0	NK/CD8 T cells	EEF1B2
0	1.17043865	0.328	0.121	0	NK/CD8 T cells	DDB1
0	1.17041403	0.459	0.174	0	NK/CD8 T cells	FUBP1
0	1.16826234	0.303	0.105	0	NK/CD8 T cells	CPT1A
0	1.16821498	0.999	0.928	0	NK/CD8 T cells	MT-ND2
0	1.16805425	0.258	0.089	0	NK/CD8 T cells	CBX5
0	1.16771179	0.416	0.137	0	NK/CD8 T cells	SLC20A1
0	1.1674155	0.332	0.114	0	NK/CD8 T cells	WHAMM
0	1.16686659	0.713	0.295	0	NK/CD8 T cells	RBM3
0	1.1663679	0.291	0.108	0	NK/CD8 T cells	MRPL34
0	1.16612256	0.292	0.098	0	NK/CD8 T cells	SF3B3
0	1.16570375	0.435	0.168	0	NK/CD8 T cells	ATP5F1A
0	1.16390053	0.672	0.254	0	NK/CD8 T cells	COX7C

0	1.16382282	0.382	0.138	0	NK/CD8 T cells	API5
0	1.16327021	0.29	0.099	0	NK/CD8 T cells	COX20
0	1.16324692	0.328	0.12	0	NK/CD8 T cells	RNF187
0	1.16307956	0.304	0.092	0	NK/CD8 T cells	GSE1
0	1.16307529	0.424	0.156	0	NK/CD8 T cells	ERAP1
0	1.1624349	0.683	0.209	0	NK/CD8 T cells	ANXA1
0	1.16234242	0.863	0.444	0	NK/CD8 T cells	ITGAL
0	1.16104061	0.974	0.36	0	NK/CD8 T cells	RPS23
0	1.16086607	0.655	0.202	0	NK/CD8 T cells	GBP5
0	1.15972998	0.999	0.518	0	NK/CD8 T cells	RPS6
0	1.15880917	0.347	0.122	0	NK/CD8 T cells	CTBP1
0	1.15861553	0.661	0.198	0	NK/CD8 T cells	FCMR
0	1.15806537	0.327	0.121	0	NK/CD8 T cells	TCERG1
0	1.15761156	0.268	0.101	0	NK/CD8 T cells	AIMP1
0	1.15561469	0.774	0.353	0	NK/CD8 T cells	ARF6
0	1.15544207	0.329	0.129	0	NK/CD8 T cells	APEX1
0	1.15171334	0.483	0.184	0	NK/CD8 T cells	BAZ1B
0	1.14983358	0.399	0.13	0	NK/CD8 T cells	SPTBN1
0	1.14897559	0.429	0.156	0	NK/CD8 T cells	ANP32E
0	1.14816376	0.525	0.205	0	NK/CD8 T cells	TCEA1
0	1.14689438	0.409	0.148	0	NK/CD8 T cells	FAM78A
0	1.14659805	0.563	0.22	0	NK/CD8 T cells	NSA2
0	1.14504815	0.615	0.242	0	NK/CD8 T cells	VPS13C
0	1.14503861	0.333	0.119	0	NK/CD8 T cells	FBXO7
0	1.14339536	0.657	0.252	0	NK/CD8 T cells	HINT1
0	1.14320805	0.433	0.164	0	NK/CD8 T cells	RBBP4
0	1.14302352	0.378	0.135	0	NK/CD8 T cells	DHX36
0	1.14218855	0.932	0.35	0	NK/CD8 T cells	RPL7
0	1.14164315	0.277	0.074	0	NK/CD8 T cells	RASAL3
0	1.14073726	0.286	0.109	0	NK/CD8 T cells	GRSF1
0	1.14018152	0.269	0.082	0	NK/CD8 T cells	CLSTN1
0	1.13797337	0.267	0.083	0	NK/CD8 T cells	DERL1
0	1.13474868	0.899	0.46	0	NK/CD8 T cells	SET
0	1.1331577	0.887	0.434	0	NK/CD8 T cells	SYNE2
0	1.13243431	0.27	0.09	0	NK/CD8 T cells	CREBZF
0	1.1313858	0.382	0.145	0	NK/CD8 T cells	GSPT1
0	1.12834185	0.305	0.089	0	NK/CD8 T cells	USP9Y
0	1.12799604	0.275	0.1	0	NK/CD8 T cells	NOL8
0	1.12775158	0.28	0.101	0	NK/CD8 T cells	TMX3
0	1.12763636	0.973	0.383	0	NK/CD8 T cells	RPL12
0	1.12705768	0.3	0.106	0	NK/CD8 T cells	ICE1
0	1.12667701	0.322	0.12	0	NK/CD8 T cells	ATP2A2
0	1.12667693	0.996	0.619	0	NK/CD8 T cells	RPL41
0	1.12445615	0.593	0.241	0	NK/CD8 T cells	ARL6IP1
0	1.12352279	0.957	0.377	0	NK/CD8 T cells	RPL19
0	1.12331194	0.987	0.436	0	NK/CD8 T cells	RPS3A
0	1.1229854	0.289	0.102	0	NK/CD8 T cells	CAND1
0	1.12226598	0.426	0.159	0	NK/CD8 T cells	ZBTB1
0	1.12168887	0.337	0.092	0	NK/CD8 T cells	PPP3CC
0	1.11817151	0.523	0.139	0	NK/CD8 T cells	ITK

0	1.11668252	0.608	0.25	0	NK/CD8 T cells	EFCAB14
0	1.11663332	0.759	0.338	0	NK/CD8 T cells	MATR3
0	1.1161609	0.311	0.12	0	NK/CD8 T cells	NDUFA12
0	1.11430275	0.271	0.1	0	NK/CD8 T cells	WDR43
0	1.11372859	0.289	0.093	0	NK/CD8 T cells	CD2AP
0	1.11257784	0.335	0.104	0	NK/CD8 T cells	ITGA6
0	1.10980292	0.27	0.083	0	NK/CD8 T cells	RAP1GDS1
0	1.10970717	0.327	0.112	0	NK/CD8 T cells	CUL4A
0	1.10867822	0.785	0.355	0	NK/CD8 T cells	EIF4A2
0	1.10847276	0.252	0.09	0	NK/CD8 T cells	AASDHPPT
0	1.1080922	0.27	0.092	0	NK/CD8 T cells	TERF1
0	1.1063144	0.355	0.142	0	NK/CD8 T cells	IPO7
0	1.10630669	0.522	0.21	0	NK/CD8 T cells	EIF3F
0	1.10552094	0.985	0.435	0	NK/CD8 T cells	RPS28
0	1.10516711	0.308	0.118	0	NK/CD8 T cells	LARS
0	1.10487458	0.729	0.325	0	NK/CD8 T cells	MAN1A2
0	1.10466483	0.32	0.12	0	NK/CD8 T cells	CYP20A1
0	1.10349815	0.494	0.196	0	NK/CD8 T cells	RBMX
0	1.10335565	0.273	0.101	0	NK/CD8 T cells	PSMC5
0	1.10293277	0.36	0.137	0	NK/CD8 T cells	TRIM44
0	1.10175299	0.27	0.095	0	NK/CD8 T cells	SBDS
0	1.1013107	0.499	0.2	0	NK/CD8 T cells	OIP5-AS1
0	1.09953148	0.279	0.099	0	NK/CD8 T cells	UBA2
0	1.09876918	0.97	0.444	0	NK/CD8 T cells	RPS25
0	1.09839477	0.539	0.213	0	NK/CD8 T cells	SATB1
0	1.09817643	0.337	0.131	0	NK/CD8 T cells	SNRPB2
0	1.09742187	0.506	0.203	0	NK/CD8 T cells	RPS4Y1
0	1.09727956	0.272	0.08	0	NK/CD8 T cells	ARID5B
0	1.09633218	0.564	0.224	0	NK/CD8 T cells	C9orf78
0	1.09597931	0.872	0.325	0	NK/CD8 T cells	RACK1
0	1.09507892	0.448	0.179	0	NK/CD8 T cells	ADCY7
0	1.0943894	0.363	0.118	0	NK/CD8 T cells	LY6E
0	1.09293759	0.255	0.088	0	NK/CD8 T cells	NAA15
0	1.09186797	0.424	0.167	0	NK/CD8 T cells	EIF1AX
0	1.09128058	0.365	0.145	0	NK/CD8 T cells	NDUFS5
0	1.08865085	0.264	0.096	0	NK/CD8 T cells	KRT10
0	1.08720766	0.943	0.375	0	NK/CD8 T cells	RPL29
0	1.0863197	0.891	0.366	0	NK/CD8 T cells	YBX1
0	1.08617155	0.273	0.098	0	NK/CD8 T cells	DKC1
0	1.08576061	0.367	0.148	0	NK/CD8 T cells	TOMM6
0	1.08446739	0.827	0.336	0	NK/CD8 T cells	TOMM7
0	1.08368424	0.633	0.244	0	NK/CD8 T cells	SNHG5
0	1.08284489	0.677	0.267	0	NK/CD8 T cells	SEPTIN6
0	1.08280985	0.618	0.238	0	NK/CD8 T cells	TMC8
0	1.0825689	0.498	0.195	0	NK/CD8 T cells	TTC3
0	1.08192752	0.488	0.192	0	NK/CD8 T cells	TRA2B
0	1.08120277	0.98	0.431	0	NK/CD8 T cells	RPS16
0	1.08081076	0.51	0.2	0	NK/CD8 T cells	RSL1D1
0	1.07955156	0.804	0.38	0	NK/CD8 T cells	RAP1B
0	1.07952067	0.29	0.088	0	NK/CD8 T cells	NUP210

0	1.07863031	0.297	0.102	0	NK/CD8 T cells	OAS2
0	1.07815277	0.259	0.074	0	NK/CD8 T cells	SESN1
0	1.0779954	0.272	0.106	0	NK/CD8 T cells	NDUFB9
0	1.07787477	1	0.93	0	NK/CD8 T cells	MT-ND3
0	1.07700885	0.377	0.147	0	NK/CD8 T cells	GIMAP2
0	1.07699455	0.819	0.301	0	NK/CD8 T cells	RPL18A
0	1.07698288	0.375	0.135	0	NK/CD8 T cells	MRFAP1L1
0	1.07677393	0.941	0.32	0	NK/CD8 T cells	RPL4
0	1.07591322	0.382	0.143	0	NK/CD8 T cells	ZNF800
0	1.07483321	0.251	0.078	0	NK/CD8 T cells	DDHD1
0	1.07310612	0.63	0.262	0	NK/CD8 T cells	HNRNPH1
0	1.07278678	0.283	0.092	0	NK/CD8 T cells	MPRIP
0	1.0694523	0.794	0.318	0	NK/CD8 T cells	RPS15
0	1.06869821	0.467	0.183	0	NK/CD8 T cells	SEC31A
0	1.06847978	0.602	0.171	0	NK/CD8 T cells	BCL11B
0	1.06488376	0.966	0.422	0	NK/CD8 T cells	RPLP1
0	1.06411306	0.54	0.215	0	NK/CD8 T cells	HMGN1
0	1.06358031	0.997	0.606	0	NK/CD8 T cells	RPL37
0	1.06330484	0.751	0.35	0	NK/CD8 T cells	CD47
0	1.06237449	0.98	0.4	0	NK/CD8 T cells	RPLP2
0	1.0618665	0.999	0.647	0	NK/CD8 T cells	RPL34
0	1.0612781	0.385	0.154	0	NK/CD8 T cells	SSR3
0	1.0604416	0.293	0.101	0	NK/CD8 T cells	CENPC
0	1.05886442	0.342	0.128	0	NK/CD8 T cells	THAP12
0	1.05817254	0.95	0.386	0	NK/CD8 T cells	RPL35A
0	1.05446018	0.336	0.135	0	NK/CD8 T cells	SNRPE
0	1.05391241	0.704	0.271	0	NK/CD8 T cells	RPL18
0	1.05346467	0.517	0.183	0	NK/CD8 T cells	MGAT4A
0	1.05312208	0.639	0.278	0	NK/CD8 T cells	KCNAB2
0	1.05269158	0.261	0.103	0	NK/CD8 T cells	NDUFAB1
0	1.05197359	0.514	0.203	0	NK/CD8 T cells	NDUFA4
0	1.05129653	0.995	0.426	0	NK/CD8 T cells	RPS8
0	1.05012926	0.44	0.174	0	NK/CD8 T cells	ERCC5
0	1.04942079	0.771	0.292	0	NK/CD8 T cells	EEF2
0	1.04925845	0.889	0.509	0	NK/CD8 T cells	PPP2R5C
0	1.04819757	0.936	0.354	0	NK/CD8 T cells	RPL36
0	1.04768189	0.253	0.1	0	NK/CD8 T cells	LPAR6
0	1.04688203	0.349	0.126	0	NK/CD8 T cells	KPNA3
0	1.04482875	0.295	0.124	0	NK/CD8 T cells	LRRC58
0	1.04476405	0.44	0.154	0	NK/CD8 T cells	FAM117A
0	1.04454667	0.995	0.482	0	NK/CD8 T cells	RPL9
0	1.0441169	0.826	0.319	0	NK/CD8 T cells	RPL8
0	1.04369012	0.71	0.294	0	NK/CD8 T cells	MTDH
0	1.04347779	0.377	0.152	0	NK/CD8 T cells	COPZ1
0	1.04324059	0.296	0.107	0	NK/CD8 T cells	ZNF83
0	1.04315743	0.312	0.113	0	NK/CD8 T cells	TMEM106B
0	1.04260425	0.257	0.092	0	NK/CD8 T cells	CNOT9
0	1.04231933	0.309	0.123	0	NK/CD8 T cells	DYNLL1
0	1.04145197	0.308	0.122	0	NK/CD8 T cells	ARL2BP
0	1.04075251	0.739	0.303	0	NK/CD8 T cells	BTF3

0	1.04055642	0.302	0.122	0	NK/CD8 T cells	NDUFC2
0	1.04002056	0.266	0.095	0	NK/CD8 T cells	ASCC3
0	1.03765203	0.896	0.317	0	NK/CD8 T cells	RPS5
0	1.03759725	0.421	0.167	0	NK/CD8 T cells	RNPS1
0	1.03647705	0.282	0.119	0	NK/CD8 T cells	CCT8
0	1.03619409	0.578	0.245	0	NK/CD8 T cells	TOP2B
0	1.03605603	0.462	0.189	0	NK/CD8 T cells	DBI
0	1.03423313	0.36	0.132	0	NK/CD8 T cells	NPAT
0	1.03403166	0.255	0.092	0	NK/CD8 T cells	TECR
0	1.03204845	0.317	0.128	0	NK/CD8 T cells	APPL1
0	1.03100859	0.343	0.122	0	NK/CD8 T cells	ZNF101
0	1.03097556	0.435	0.112	0	NK/CD8 T cells	TNIK
0	1.03008417	0.442	0.176	0	NK/CD8 T cells	SUPT16H
0	1.03006588	0.411	0.157	0	NK/CD8 T cells	NFATC2IP
0	1.0281749	0.572	0.246	0	NK/CD8 T cells	SRSF2
0	1.02687575	0.318	0.12	0	NK/CD8 T cells	ARL5A
0	1.02610846	0.527	0.218	0	NK/CD8 T cells	HELZ
0	1.02390183	0.363	0.163	0	NK/CD8 T cells	HIPK2
0	1.02063326	0.356	0.114	0	NK/CD8 T cells	DIP2A
0	1.01909108	0.477	0.198	0	NK/CD8 T cells	WBP11
0	1.01870995	0.291	0.091	0	NK/CD8 T cells	RNF115
0	1.0166027	0.407	0.159	0	NK/CD8 T cells	SMC5
0	1.01647863	0.959	0.331	0	NK/CD8 T cells	RPL22
0	1.01622322	0.378	0.155	0	NK/CD8 T cells	TNFAIP3
0	1.01606578	0.449	0.191	0	NK/CD8 T cells	EIF5A
0	1.01327685	0.295	0.125	0	NK/CD8 T cells	SUMO3
0	1.01252662	0.995	0.474	0	NK/CD8 T cells	RPL32
0	1.01175577	0.788	0.333	0	NK/CD8 T cells	CAST
0	1.00941576	0.766	0.343	0	NK/CD8 T cells	EMB
0	1.00913142	0.261	0.097	0	NK/CD8 T cells	PSMA5
0	1.00891276	0.378	0.151	0	NK/CD8 T cells	PSMB2
0	1.00808198	0.522	0.205	0	NK/CD8 T cells	SH3BP5
0	1.00389227	0.349	0.104	0	NK/CD8 T cells	BHLHE40
0	1.00308203	0.653	0.299	0	NK/CD8 T cells	PNRC2
0	1.00244821	0.367	0.144	0	NK/CD8 T cells	AIDA
0	1.00221202	0.27	0.106	0	NK/CD8 T cells	LRPPRC
0	1.00197041	0.345	0.118	0	NK/CD8 T cells	KAT6B
0	1.00165189	0.356	0.134	0	NK/CD8 T cells	SACM1L
0	1.00145063	0.257	0.097	0	NK/CD8 T cells	OTUD4
0	0.99965969	0.331	0.13	0	NK/CD8 T cells	PPIB
0	0.99959904	0.762	0.323	0	NK/CD8 T cells	CD48
0	0.99902599	0.385	0.143	0	NK/CD8 T cells	NAP1L4
0	0.99852065	0.418	0.173	0	NK/CD8 T cells	SNRPG
0	0.99663333	0.356	0.144	0	NK/CD8 T cells	DNTTIP2
0	0.99553024	0.302	0.121	0	NK/CD8 T cells	KARS
0	0.99479729	0.61	0.261	0	NK/CD8 T cells	C11orf58
0	0.99186761	0.682	0.285	0	NK/CD8 T cells	TRAF3IP3
0	0.99107525	0.391	0.149	0	NK/CD8 T cells	HMGN4
0	0.99106826	0.31	0.13	0	NK/CD8 T cells	DYNC1I2
0	0.99103399	0.419	0.161	0	NK/CD8 T cells	HUWE1

0	0.99023343	0.301	0.117	0	NK/CD8 T cells	TMEM50B
0	0.99005045	0.448	0.163	0	NK/CD8 T cells	LINC00623
0	0.98998473	0.306	0.133	0	NK/CD8 T cells	EIF2A
0	0.98878944	0.309	0.123	0	NK/CD8 T cells	NUTF2
0	0.98776277	0.464	0.193	0	NK/CD8 T cells	THOC2
0	0.98710662	0.977	0.467	0	NK/CD8 T cells	RPL38
0	0.98501971	0.251	0.098	0	NK/CD8 T cells	RPF1
0	0.98374983	0.283	0.098	0	NK/CD8 T cells	POLR3GL
0	0.98273767	0.338	0.111	0	NK/CD8 T cells	PRKY
0	0.98260188	0.255	0.09	0	NK/CD8 T cells	GOPC
0	0.98220538	0.345	0.14	0	NK/CD8 T cells	VPS36
0	0.98108385	0.779	0.218	0	NK/CD8 T cells	SYNE1
0	0.98098515	0.389	0.142	0	NK/CD8 T cells	ZNF276
0	0.97989677	0.291	0.103	0	NK/CD8 T cells	MGA
0	0.97982512	0.459	0.168	0	NK/CD8 T cells	USP47
0	0.97883548	0.255	0.107	0	NK/CD8 T cells	CNDP2
0	0.97863581	0.313	0.095	0	NK/CD8 T cells	AKT3
0	0.97862169	0.62	0.278	0	NK/CD8 T cells	NONO
0	0.97665677	0.419	0.164	0	NK/CD8 T cells	ARIH2
0	0.97607183	0.972	0.502	0	NK/CD8 T cells	RPS11
0	0.9759579	0.281	0.112	0	NK/CD8 T cells	AKAP11
0	0.97319527	0.293	0.11	0	NK/CD8 T cells	SUGP2
0	0.97275146	0.483	0.201	0	NK/CD8 T cells	SPCS2
0	0.97144043	0.286	0.11	0	NK/CD8 T cells	CBX6
0	0.97132207	0.305	0.112	0	NK/CD8 T cells	TRIM52
0	0.97036173	0.257	0.076	0	NK/CD8 T cells	DENND4C
0	0.96768396	0.39	0.154	0	NK/CD8 T cells	CRBN
0	0.96543108	0.333	0.107	0	NK/CD8 T cells	TTC39C
0	0.96412137	0.255	0.104	0	NK/CD8 T cells	FEM1B
0	0.963642	0.708	0.317	0	NK/CD8 T cells	COMMD6
0	0.96309833	0.428	0.166	0	NK/CD8 T cells	RPA2
0	0.96213904	0.259	0.095	0	NK/CD8 T cells	CA5B
0	0.96086026	0.953	0.575	0	NK/CD8 T cells	HNRNPA2B1
0	0.95766819	0.628	0.285	0	NK/CD8 T cells	HNRNPF
0	0.95763854	0.299	0.096	0	NK/CD8 T cells	PRKCQ-AS1
0	0.9566048	0.527	0.2	0	NK/CD8 T cells	SPATA13
0	0.95641868	0.367	0.131	0	NK/CD8 T cells	GSTP1
0	0.95447747	0.307	0.126	0	NK/CD8 T cells	NASP
0	0.95366634	0.482	0.187	0	NK/CD8 T cells	PTPRA
0	0.95357606	0.466	0.189	0	NK/CD8 T cells	SERPINB9
0	0.95235498	0.986	0.485	0	NK/CD8 T cells	RPL23
0	0.95197581	0.319	0.135	0	NK/CD8 T cells	ABRACL
0	0.9508736	0.334	0.133	0	NK/CD8 T cells	UBP1
0	0.95027047	0.399	0.17	0	NK/CD8 T cells	UQCRH
0	0.94866608	0.507	0.22	0	NK/CD8 T cells	ZRANB2
0	0.94729829	0.257	0.11	0	NK/CD8 T cells	ELK3
0	0.94712274	0.482	0.195	0	NK/CD8 T cells	EIF2AK1
0	0.94564202	0.732	0.33	0	NK/CD8 T cells	EIF3A
0	0.94374745	0.869	0.415	0	NK/CD8 T cells	RPL28
0	0.94284807	0.386	0.164	0	NK/CD8 T cells	LARP1

0	0.94257644	0.394	0.158	0	NK/CD8 T cells	DYNC1H1
0	0.94153336	0.512	0.177	0	NK/CD8 T cells	S100A10
0	0.94130703	0.424	0.181	0	NK/CD8 T cells	DNAJC8
0	0.93907763	0.269	0.096	0	NK/CD8 T cells	CHD6
0	0.93733001	0.512	0.216	0	NK/CD8 T cells	LDHA
0	0.93712741	0.326	0.137	0	NK/CD8 T cells	SNU13
0	0.93670056	0.733	0.292	0	NK/CD8 T cells	EEF1G
0	0.93565582	0.995	0.628	0	NK/CD8 T cells	RPL30
0	0.93399565	0.389	0.159	0	NK/CD8 T cells	ETNK1
0	0.93378165	0.555	0.226	0	NK/CD8 T cells	SYNRG
0	0.93215383	0.975	0.436	0	NK/CD8 T cells	RPL11
0	0.93093816	0.389	0.159	0	NK/CD8 T cells	BRD7
0	0.92985101	0.317	0.129	0	NK/CD8 T cells	LSM8
0	0.92828306	0.76	0.33	0	NK/CD8 T cells	ATP5MC2
0	0.92689504	0.564	0.223	0	NK/CD8 T cells	SLC25A6
0	0.92615682	0.342	0.149	0	NK/CD8 T cells	DUT
0	0.92407649	0.268	0.096	0	NK/CD8 T cells	LIX1L
0	0.92397302	0.416	0.181	0	NK/CD8 T cells	TARDBP
0	0.923551	0.806	0.383	0	NK/CD8 T cells	UTRN
0	0.9227038	0.332	0.146	0	NK/CD8 T cells	TUFM
0	0.92130113	0.686	0.324	0	NK/CD8 T cells	SFPQ
0	0.91823029	0.487	0.204	0	NK/CD8 T cells	PRRC2B
0	0.91771956	0.381	0.164	0	NK/CD8 T cells	PARK7
0	0.91699894	0.432	0.17	0	NK/CD8 T cells	DYNLL2
0	0.91549624	0.488	0.201	0	NK/CD8 T cells	PPP2R5E
0	0.91441191	0.518	0.229	0	NK/CD8 T cells	U2SURP
0	0.91383778	0.346	0.146	0	NK/CD8 T cells	TMED9
0	0.91368764	0.278	0.119	0	NK/CD8 T cells	COX5A
0	0.91336624	0.321	0.125	0	NK/CD8 T cells	VPS26B
0	0.91260651	0.254	0.11	0	NK/CD8 T cells	PSMG2
0	0.91158922	0.265	0.109	0	NK/CD8 T cells	NUDT21
0	0.91119474	0.252	0.104	0	NK/CD8 T cells	RPS27L
0	0.91001021	0.297	0.094	0	NK/CD8 T cells	THEMIS
0	0.90958519	0.326	0.142	0	NK/CD8 T cells	EIF4G1
0	0.9089506	0.292	0.117	0	NK/CD8 T cells	TMEM245
0	0.90788864	0.312	0.134	0	NK/CD8 T cells	TMEM9B
0	0.90727117	0.992	0.554	0	NK/CD8 T cells	RPL39
0	0.90679259	0.478	0.211	0	NK/CD8 T cells	HNRNPA0
0	0.90607129	0.542	0.244	0	NK/CD8 T cells	ATP5PB
0	0.90482439	0.574	0.267	0	NK/CD8 T cells	BZW1
0	0.90472627	0.346	0.145	0	NK/CD8 T cells	UBE2N
0	0.90405013	0.289	0.104	0	NK/CD8 T cells	AEBP2
0	0.90312381	0.32	0.137	0	NK/CD8 T cells	TMPO
0	0.90268269	0.478	0.196	0	NK/CD8 T cells	SHFL
0	0.90106355	0.331	0.136	0	NK/CD8 T cells	GANAB
0	0.90091893	0.297	0.135	0	NK/CD8 T cells	SMDT1
0	0.90057206	0.371	0.141	0	NK/CD8 T cells	CHD9
0	0.89991661	0.378	0.165	0	NK/CD8 T cells	SLC25A5
0	0.89982943	0.504	0.21	0	NK/CD8 T cells	FNBP4
0	0.89952063	0.265	0.116	0	NK/CD8 T cells	PTDSS1

0	0.897648	0.72	0.335	0	NK/CD8 T cells	SEPTIN9
0	0.8969801	0.296	0.123	0	NK/CD8 T cells	BTBD1
0	0.89681264	0.537	0.241	0	NK/CD8 T cells	PRPF4B
0	0.89653468	0.817	0.417	0	NK/CD8 T cells	SRSF11
0	0.89494474	0.812	0.402	0	NK/CD8 T cells	FAM107B
0	0.89432348	0.301	0.105	0	NK/CD8 T cells	CAMK2D
0	0.89428855	0.328	0.117	0	NK/CD8 T cells	HIST1H4C
0	0.89409676	0.367	0.168	0	NK/CD8 T cells	TOMM20
0	0.89345861	0.357	0.135	0	NK/CD8 T cells	WWP1
0	0.89328237	0.408	0.163	0	NK/CD8 T cells	ZNF131
0	0.89162435	0.261	0.09	0	NK/CD8 T cells	MAP4
0	0.89053062	0.253	0.098	0	NK/CD8 T cells	PHF14
0	0.88559496	0.439	0.195	0	NK/CD8 T cells	ZC3H15
0	0.8849459	0.322	0.127	0	NK/CD8 T cells	LRRC8C
0	0.88418272	0.306	0.125	0	NK/CD8 T cells	P4HB
0	0.88398691	0.573	0.252	0	NK/CD8 T cells	DCAF7
0	0.88272268	0.706	0.31	0	NK/CD8 T cells	CYFIP2
0	0.87917506	0.273	0.12	0	NK/CD8 T cells	NBEAL1
0	0.87903554	0.311	0.124	0	NK/CD8 T cells	SUCLG2
0	0.87895298	0.255	0.092	0	NK/CD8 T cells	PCNX4
0	0.87825683	0.288	0.121	0	NK/CD8 T cells	PSME2
0	0.87757311	0.25	0.094	0	NK/CD8 T cells	SREBF2
0	0.87513132	0.607	0.281	0	NK/CD8 T cells	HNRNPM
0	0.87441101	0.354	0.156	0	NK/CD8 T cells	ATP1A1
0	0.87101845	0.372	0.161	0	NK/CD8 T cells	MED1
0	0.8668609	0.322	0.133	0	NK/CD8 T cells	SURF4
0	0.86622031	0.293	0.099	0	NK/CD8 T cells	TSPOAP1-AS1
0	0.86541877	0.505	0.206	0	NK/CD8 T cells	CLINT1
0	0.86444846	0.594	0.284	0	NK/CD8 T cells	RPL36AL
0	0.86400993	0.265	0.097	0	NK/CD8 T cells	SNHG14
0	0.86385932	0.325	0.142	0	NK/CD8 T cells	GLUD1
0	0.86335492	0.599	0.284	0	NK/CD8 T cells	SPCS3
0	0.86148177	0.356	0.144	0	NK/CD8 T cells	NUCB2
0	0.86130289	0.294	0.126	0	NK/CD8 T cells	KIAA1143
0	0.86117613	0.255	0.105	0	NK/CD8 T cells	MT-ND6
0	0.85954883	0.861	0.374	0	NK/CD8 T cells	TMSB10
0	0.8593042	0.54	0.207	0	NK/CD8 T cells	AAK1
0	0.85837764	0.33	0.149	0	NK/CD8 T cells	RSL24D1
0	0.85835611	0.858	0.372	0	NK/CD8 T cells	SAMHD1
0	0.85592493	0.282	0.123	0	NK/CD8 T cells	EMC4
0	0.85533592	0.317	0.129	0	NK/CD8 T cells	MAGT1
0	0.85495846	0.407	0.182	0	NK/CD8 T cells	RBM17
0	0.85445612	0.53	0.198	0	NK/CD8 T cells	ST6GAL1
0	0.85308109	0.341	0.151	0	NK/CD8 T cells	PSMA6
0	0.85305954	0.41	0.166	0	NK/CD8 T cells	DCAF5
0	0.85270358	0.335	0.135	0	NK/CD8 T cells	C21orf91
0	0.85231408	0.94	0.567	0	NK/CD8 T cells	IL2RG
0	0.85152885	0.269	0.108	0	NK/CD8 T cells	POLR2G
0	0.84994893	0.448	0.156	0	NK/CD8 T cells	DOCK10
0	0.84631738	0.621	0.281	0	NK/CD8 T cells	BTN3A1

0	0.84325012	0.446	0.191	0	NK/CD8 T cells	CCAR1
0	0.84320838	0.423	0.193	0	NK/CD8 T cells	SRP9
0	0.84185532	0.352	0.114	0	NK/CD8 T cells	LRBA
0	0.83878716	0.407	0.177	0	NK/CD8 T cells	PTPN2
0	0.8384022	0.492	0.217	0	NK/CD8 T cells	XPO1
0	0.83779755	0.389	0.157	0	NK/CD8 T cells	STK26
0	0.83758268	0.282	0.121	0	NK/CD8 T cells	SNRPB
0	0.83694579	0.341	0.146	0	NK/CD8 T cells	PPM1G
0	0.83677439	0.607	0.272	0	NK/CD8 T cells	CAPNS1
0	0.83375738	0.276	0.119	0	NK/CD8 T cells	DARS
0	0.8335803	0.513	0.194	0	NK/CD8 T cells	P2RY8
0	0.83294362	0.251	0.097	0	NK/CD8 T cells	ATXN10
0	0.83224771	0.305	0.119	0	NK/CD8 T cells	TENT4B
0	0.83221902	0.289	0.093	0	NK/CD8 T cells	GALNT10
0	0.8314064	0.337	0.14	0	NK/CD8 T cells	DAXX
0	0.83032349	0.66	0.317	0	NK/CD8 T cells	UQCRB
0	0.8293383	0.621	0.289	0	NK/CD8 T cells	PRMT2
0	0.82900243	0.414	0.181	0	NK/CD8 T cells	DHX15
0	0.82659194	0.336	0.145	0	NK/CD8 T cells	PTPN11
0	0.82658092	0.373	0.169	0	NK/CD8 T cells	TMCO1
0	0.82650702	0.319	0.147	0	NK/CD8 T cells	PHB2
0	0.82399034	0.252	0.11	0	NK/CD8 T cells	EDF1
0	0.82381205	0.892	0.432	0	NK/CD8 T cells	NACA
0	0.82341415	0.456	0.17	0	NK/CD8 T cells	TNRC6C
0	0.82297142	0.285	0.134	0	NK/CD8 T cells	EIF3M
0	0.82222655	0.295	0.127	0	NK/CD8 T cells	DDX27
0	0.82186993	0.273	0.125	0	NK/CD8 T cells	TMX1
0	0.82093765	0.505	0.233	0	NK/CD8 T cells	PTBP1
0	0.8190041	0.607	0.261	0	NK/CD8 T cells	EIF4A1
0	0.81898712	0.875	0.52	0	NK/CD8 T cells	SEPTIN7
0	0.81807083	0.426	0.192	0	NK/CD8 T cells	GAS5
0	0.8174805	0.441	0.196	0	NK/CD8 T cells	RO60
0	0.81698489	0.988	0.445	0	NK/CD8 T cells	RPS13
0	0.81611886	0.343	0.157	0	NK/CD8 T cells	CACYBP
0	0.81590367	0.28	0.112	0	NK/CD8 T cells	SP140L
0	0.81572383	0.355	0.157	0	NK/CD8 T cells	TMED4
0	0.81546547	0.519	0.237	0	NK/CD8 T cells	ST13
0	0.81376118	0.462	0.212	0	NK/CD8 T cells	SMC3
0	0.81225188	0.508	0.234	0	NK/CD8 T cells	ARHGDIA
0	0.81212105	0.358	0.161	0	NK/CD8 T cells	TIA1
0	0.81187258	0.326	0.136	0	NK/CD8 T cells	CASP8AP2
0	0.80987665	0.46	0.21	0	NK/CD8 T cells	DHX9
0	0.80939626	0.272	0.112	0	NK/CD8 T cells	MRPS6
0	0.80918209	0.531	0.25	0	NK/CD8 T cells	ATXN2L
0	0.80864299	0.32	0.138	0	NK/CD8 T cells	CEP57
0	0.80510317	0.273	0.116	0	NK/CD8 T cells	STYX
0	0.80494674	0.282	0.127	0	NK/CD8 T cells	ORMDL1
0	0.80481342	0.293	0.137	0	NK/CD8 T cells	NOL7
0	0.80451822	0.404	0.175	0	NK/CD8 T cells	G3BP2
0	0.8040693	0.375	0.158	0	NK/CD8 T cells	ANAPC5

0	0.80344599	0.486	0.225	0	NK/CD8 T cells	TIAL1
0	0.80291653	0.261	0.111	0	NK/CD8 T cells	EXOC5
0	0.8013928	0.614	0.298	0	NK/CD8 T cells	KLF13
0	0.80133801	0.293	0.106	0	NK/CD8 T cells	ATP8A1
0	0.80084188	0.251	0.101	0	NK/CD8 T cells	NUMA1
0	0.80076341	0.382	0.164	0	NK/CD8 T cells	SNRNP200
0	0.79994107	0.443	0.185	0	NK/CD8 T cells	PDS5A
0	0.79970944	0.301	0.136	0	NK/CD8 T cells	TCP1
0	0.79892111	0.425	0.194	0	NK/CD8 T cells	SRSF10
0	0.79818372	0.311	0.129	0	NK/CD8 T cells	ZNF644
0	0.79756557	0.388	0.171	0	NK/CD8 T cells	POLR2B
0	0.79705176	0.358	0.168	0	NK/CD8 T cells	COX7A2L
0	0.7966482	0.32	0.147	0	NK/CD8 T cells	ERH
0	0.79580041	0.474	0.216	0	NK/CD8 T cells	CERK
0	0.79453584	0.463	0.197	0	NK/CD8 T cells	PTPN18
0	0.79400644	0.27	0.099	0	NK/CD8 T cells	TMC6
0	0.79279581	0.554	0.256	0	NK/CD8 T cells	PCM1
0	0.79205343	0.26	0.123	0	NK/CD8 T cells	SNX17
0	0.78955953	0.288	0.119	0	NK/CD8 T cells	ZNF451
0	0.78937699	0.5	0.234	0	NK/CD8 T cells	DDX21
0	0.78920466	0.322	0.149	0	NK/CD8 T cells	SRSF6
0	0.78590467	0.302	0.14	0	NK/CD8 T cells	ATP5F1C
0	0.7852652	0.325	0.155	0	NK/CD8 T cells	NDUFB4
0	0.78485105	0.263	0.108	0	NK/CD8 T cells	DDX50
0	0.78458757	0.412	0.191	0	NK/CD8 T cells	CIAO1
0	0.78434346	0.564	0.26	0	NK/CD8 T cells	GOLGA4
0	0.78301885	0.378	0.173	0	NK/CD8 T cells	PCNP
0	0.78264456	0.337	0.133	0	NK/CD8 T cells	USP24
0	0.78246792	0.255	0.109	0	NK/CD8 T cells	GABPB1-IT1
0	0.78243314	0.371	0.157	0	NK/CD8 T cells	SAR1A
0	0.78211242	0.412	0.182	0	NK/CD8 T cells	CTR9
0	0.78111336	0.312	0.14	0	NK/CD8 T cells	USP16
0	0.78070814	0.358	0.162	0	NK/CD8 T cells	XRCC6
0	0.77870275	0.463	0.218	0	NK/CD8 T cells	MAZ
0	0.77595164	0.661	0.339	0	NK/CD8 T cells	CD164
0	0.77365076	0.563	0.238	0	NK/CD8 T cells	SNHG29
0	0.77289083	0.398	0.179	0	NK/CD8 T cells	UBTF
0	0.77217348	1	0.886	0	NK/CD8 T cells	TPT1
0	0.77128365	0.559	0.267	0	NK/CD8 T cells	SMARCA5
0	0.77119003	0.3	0.121	0	NK/CD8 T cells	TRPM7
0	0.770704	0.318	0.141	0	NK/CD8 T cells	TTC14
0	0.77032842	0.252	0.108	0	NK/CD8 T cells	ACLY
0	0.77019775	0.517	0.229	0	NK/CD8 T cells	LUC7L2
0	0.76968328	0.563	0.246	0	NK/CD8 T cells	EIF3E
0	0.7672401	0.264	0.114	0	NK/CD8 T cells	CUL5
0	0.76640645	0.477	0.226	0	NK/CD8 T cells	ATP5F1B
0	0.7657705	0.412	0.195	0	NK/CD8 T cells	ZKSCAN1
0	0.76299556	0.505	0.243	0	NK/CD8 T cells	TMEM258
0	0.76265028	0.274	0.122	0	NK/CD8 T cells	FRG1
0	0.76009336	0.273	0.112	0	NK/CD8 T cells	POM121

0	0.75947025	0.64	0.305	0	NK/CD8 T cells	OST4
0	0.75798603	0.514	0.244	0	NK/CD8 T cells	FXYD5
0	0.75797068	0.276	0.136	0	NK/CD8 T cells	ATP5PO
0	0.75767964	0.339	0.145	0	NK/CD8 T cells	SRI
0	0.75665345	0.486	0.223	0	NK/CD8 T cells	OGT
0	0.75491482	0.388	0.161	0	NK/CD8 T cells	STIM2
0	0.75464248	0.298	0.136	0	NK/CD8 T cells	AK2
0	0.75287683	0.35	0.157	0	NK/CD8 T cells	USP1
0	0.75143857	0.454	0.215	0	NK/CD8 T cells	EIF3K
0	0.75137403	0.536	0.263	0	NK/CD8 T cells	HNRNPL
0	0.75102919	0.35	0.159	0	NK/CD8 T cells	FNTA
0	0.75028819	0.312	0.139	0	NK/CD8 T cells	CHMP4A
0	0.75001685	0.765	0.4	0	NK/CD8 T cells	DIAPH1
0	0.74993666	0.298	0.134	0	NK/CD8 T cells	TMEM230
0	0.7497442	0.292	0.133	0	NK/CD8 T cells	TRIM4
0	0.74942083	0.584	0.279	0	NK/CD8 T cells	NORAD
0	0.74813893	0.414	0.203	0	NK/CD8 T cells	POLR1D
0	0.74576302	0.265	0.098	0	NK/CD8 T cells	GAB3
0	0.74537762	0.405	0.181	0	NK/CD8 T cells	CHD3
0	0.74306077	0.333	0.152	0	NK/CD8 T cells	TRIP11
0	0.7429358	0.425	0.199	0	NK/CD8 T cells	REST
0	0.74262561	0.389	0.171	0	NK/CD8 T cells	DENND1C
0	0.73976981	0.281	0.128	0	NK/CD8 T cells	PSMA3
0	0.7390106	0.819	0.47	0	NK/CD8 T cells	RBL2
0	0.738584	0.391	0.158	0	NK/CD8 T cells	TGFBR1
0	0.73821431	0.333	0.147	0	NK/CD8 T cells	EIF3J
0	0.7369654	0.566	0.267	0	NK/CD8 T cells	ENO1
0	0.73678085	1	0.939	0	NK/CD8 T cells	MT-ND1
0	0.73324723	0.27	0.123	0	NK/CD8 T cells	MRPL20
0	0.73176967	0.356	0.16	0	NK/CD8 T cells	CSNK2A1
0	0.73132781	0.254	0.116	0	NK/CD8 T cells	DIS3
0	0.73038492	0.254	0.11	0	NK/CD8 T cells	DDX23
0	0.73012114	0.268	0.118	0	NK/CD8 T cells	SARS
0	0.72885936	0.259	0.122	0	NK/CD8 T cells	BST2
0	0.72815711	0.334	0.141	0	NK/CD8 T cells	ANKIB1
0	0.72814573	0.259	0.107	0	NK/CD8 T cells	PHACTR4
0	0.72151268	0.419	0.198	0	NK/CD8 T cells	TMED5
0	0.71886905	0.648	0.304	0	NK/CD8 T cells	EIF3H
0	0.71760117	0.931	0.614	0	NK/CD8 T cells	PTP4A2
0	0.71380589	0.445	0.195	0	NK/CD8 T cells	AP2B1
0	0.71145579	0.285	0.085	0	NK/CD8 T cells	GRAP2
0	0.71129194	0.584	0.276	0	NK/CD8 T cells	SLC9A3R1
0	0.70944677	0.633	0.317	0	NK/CD8 T cells	ZBTB7A
0	0.70796427	0.589	0.294	0	NK/CD8 T cells	SLC25A3
0	0.70789071	0.264	0.119	0	NK/CD8 T cells	PRPF38A
0	0.70738868	0.52	0.264	0	NK/CD8 T cells	CGGBP1
0	0.70656093	0.414	0.198	0	NK/CD8 T cells	ATP5IF1
0	0.70653046	0.312	0.131	0	NK/CD8 T cells	ORAI1
0	0.70612956	0.336	0.161	0	NK/CD8 T cells	SMC1A
0	0.70592483	0.373	0.16	0	NK/CD8 T cells	RSU1

0	0.70455169	0.262	0.11	0	NK/CD8 T cells	GALC
0	0.70379256	0.351	0.17	0	NK/CD8 T cells	ATP5MD
0	0.70082388	0.266	0.115	0	NK/CD8 T cells	PPA2
0	0.69999299	0.509	0.217	0	NK/CD8 T cells	INPP4A
0	0.69771095	0.338	0.163	0	NK/CD8 T cells	C17orf49
0	0.69731957	0.49	0.157	0	NK/CD8 T cells	ANXA2
0	0.69722853	0.259	0.125	0	NK/CD8 T cells	ZNF770
0	0.69509145	0.563	0.28	0	NK/CD8 T cells	PTGES3
0	0.69388298	0.393	0.189	0	NK/CD8 T cells	VHL
0	0.69339023	0.468	0.231	0	NK/CD8 T cells	ATP5ME
0	0.69329587	0.417	0.203	0	NK/CD8 T cells	OCIAD1
0	0.69240036	0.418	0.158	0	NK/CD8 T cells	ABLIM1
0	0.69213961	0.809	0.45	0	NK/CD8 T cells	EEF1D
0	0.69139964	0.25	0.115	0	NK/CD8 T cells	SF3A2
0	0.69134082	0.373	0.181	0	NK/CD8 T cells	RANBP2
0	0.69070573	0.409	0.186	0	NK/CD8 T cells	HDAC1
0	0.69017808	0.261	0.114	0	NK/CD8 T cells	BRD1
0	0.68926432	0.362	0.129	0	NK/CD8 T cells	GLCCI1
0	0.68839568	0.281	0.127	0	NK/CD8 T cells	VDAC3
0	0.68754235	0.381	0.191	0	NK/CD8 T cells	HSPD1
0	0.68735778	0.285	0.129	0	NK/CD8 T cells	ADSS
0	0.68389257	0.314	0.146	0	NK/CD8 T cells	GLTP
0	0.68277804	0.31	0.143	0	NK/CD8 T cells	MALT1
0	0.68114589	0.327	0.164	0	NK/CD8 T cells	UQCRQ
0	0.68018391	0.747	0.402	0	NK/CD8 T cells	HNRNPD
0	0.67843754	0.293	0.114	0	NK/CD8 T cells	CCDC91
0	0.67619775	0.44	0.191	0	NK/CD8 T cells	TUT4
0	0.67559815	0.54	0.26	0	NK/CD8 T cells	IRF2BP2
0	0.67535992	0.697	0.379	0	NK/CD8 T cells	BRD2
0	0.67268278	0.519	0.263	0	NK/CD8 T cells	RBM8A
0	0.67008816	0.259	0.11	0	NK/CD8 T cells	DMTF1
0	0.6694863	0.962	0.697	0	NK/CD8 T cells	DDX5
0	0.66909304	0.557	0.194	0	NK/CD8 T cells	TCF7
0	0.66802711	0.289	0.13	0	NK/CD8 T cells	SND1
0	0.66776367	0.281	0.135	0	NK/CD8 T cells	AGGF1
0	0.66770607	0.404	0.199	0	NK/CD8 T cells	KHSRP
0	0.66597666	0.334	0.145	0	NK/CD8 T cells	IRF3
0	0.66597133	0.732	0.355	0	NK/CD8 T cells	ANP32B
0	0.6653504	0.329	0.159	0	NK/CD8 T cells	RBX1
0	0.66533958	0.668	0.345	0	NK/CD8 T cells	HSPA5
0	0.66486729	0.368	0.174	0	NK/CD8 T cells	RIF1
0	0.66464615	0.719	0.389	0	NK/CD8 T cells	HP1BP3
0	0.66273849	0.833	0.487	0	NK/CD8 T cells	RNF213
0	0.66101422	0.818	0.463	0	NK/CD8 T cells	ARL6IP5
0	0.66044268	0.441	0.216	0	NK/CD8 T cells	COPB1
0	0.65854215	0.288	0.133	0	NK/CD8 T cells	FAM133B
0	0.6578049	0.398	0.202	0	NK/CD8 T cells	EIF3D
0	0.6568506	0.483	0.237	0	NK/CD8 T cells	ZNF655
0	0.65594668	0.261	0.12	0	NK/CD8 T cells	SS18L2
0	0.65564133	0.331	0.161	0	NK/CD8 T cells	TMEM248

0	0.6550921	0.482	0.243	0	NK/CD8 T cells	EIF2S2
0	0.6534867	0.754	0.416	0	NK/CD8 T cells	TERF2IP
0	0.65210869	0.528	0.209	0	NK/CD8 T cells	LDHB
0	0.65202135	0.317	0.164	0	NK/CD8 T cells	ERP29
0	0.65191074	0.315	0.163	0	NK/CD8 T cells	PABPC4
0	0.65071025	0.376	0.177	0	NK/CD8 T cells	EFR3A
0	0.65012893	0.328	0.131	0	NK/CD8 T cells	RETREG3
0	0.64963747	0.335	0.122	0	NK/CD8 T cells	BCL2
0	0.64845405	0.399	0.196	0	NK/CD8 T cells	SMARCC2
0	0.64806711	0.304	0.136	0	NK/CD8 T cells	RSBN1
0	0.64559397	0.26	0.126	0	NK/CD8 T cells	ACO2
0	0.64389306	0.343	0.139	0	NK/CD8 T cells	GPI
0	0.64277764	0.302	0.127	0	NK/CD8 T cells	CBFB
0	0.64273256	0.323	0.155	0	NK/CD8 T cells	SETD3
0	0.64251062	0.393	0.196	0	NK/CD8 T cells	TBCA
0	0.64247107	0.252	0.114	0	NK/CD8 T cells	ECPAS
0	0.6424015	0.36	0.174	0	NK/CD8 T cells	MAPK1IP1L
0	0.64210344	0.321	0.161	0	NK/CD8 T cells	BLOC1S6
0	0.63920427	0.643	0.329	0	NK/CD8 T cells	EMP3
0	0.63816712	0.433	0.183	0	NK/CD8 T cells	NLRC5
0	0.6375704	0.291	0.129	0	NK/CD8 T cells	SF3A3
0	0.63492162	0.253	0.111	0	NK/CD8 T cells	TAF3
0	0.63485546	0.367	0.147	0	NK/CD8 T cells	MSI2
0	0.6339713	0.261	0.119	0	NK/CD8 T cells	MTF2
0	0.63238826	0.529	0.275	0	NK/CD8 T cells	CHCHD2
0	0.63233546	0.553	0.287	0	NK/CD8 T cells	H2AFV
0	0.63175204	0.343	0.151	0	NK/CD8 T cells	DGKZ
0	0.62939693	0.27	0.121	0	NK/CD8 T cells	CRTC3
0	0.6279249	0.352	0.163	0	NK/CD8 T cells	PPHLN1
0	0.62756735	0.349	0.162	0	NK/CD8 T cells	TNRC6A
0	0.62621091	0.552	0.284	0	NK/CD8 T cells	UBE2L3
0	0.62609264	0.665	0.36	0	NK/CD8 T cells	SRRM1
0	0.62570959	0.494	0.212	0	NK/CD8 T cells	ERN1
0	0.62388716	0.257	0.13	0	NK/CD8 T cells	NDUFC1
0	0.62263724	0.514	0.263	0	NK/CD8 T cells	AKAP9
0	0.62250907	0.443	0.208	0	NK/CD8 T cells	TAF15
0	0.62222029	0.568	0.303	0	NK/CD8 T cells	PNN
0	0.61931421	0.276	0.14	0	NK/CD8 T cells	GIMAP8
0	0.61848536	0.673	0.36	0	NK/CD8 T cells	ATP5MG
0	0.61761381	0.67	0.374	0	NK/CD8 T cells	PDCD4
0	0.61745814	0.332	0.163	0	NK/CD8 T cells	SREK1
0	0.61738271	0.333	0.155	0	NK/CD8 T cells	DIDO1
0	0.6168117	0.307	0.148	0	NK/CD8 T cells	UBE2Z
0	0.61537335	0.279	0.131	0	NK/CD8 T cells	ZBTB11
0	0.61534672	0.629	0.327	0	NK/CD8 T cells	LUC7L3
0	0.6144974	0.358	0.176	0	NK/CD8 T cells	TMED2
0	0.61362945	0.571	0.307	0	NK/CD8 T cells	SSR2
0	0.61123394	0.268	0.127	0	NK/CD8 T cells	BAG5
0	0.60945971	0.539	0.275	0	NK/CD8 T cells	ZC3H13
0	0.60633004	0.701	0.382	0	NK/CD8 T cells	DEK

0	0.60260903	0.918	0.639	0	NK/CD8 T cells	ZFP36L2
0	0.6023275	0.299	0.139	0	NK/CD8 T cells	TMEM87A
0	0.60208378	0.331	0.16	0	NK/CD8 T cells	USO1
0	0.60110555	0.587	0.304	0	NK/CD8 T cells	SEPTIN2
0	0.60108957	0.373	0.192	0	NK/CD8 T cells	SNHG32
0	0.60103224	0.289	0.145	0	NK/CD8 T cells	SEC61B
0	0.60086091	0.314	0.162	0	NK/CD8 T cells	NARS
0	0.5996929	0.392	0.203	0	NK/CD8 T cells	SPCS1
0	0.59965762	0.844	0.502	0	NK/CD8 T cells	ADD3
0	0.59812489	0.484	0.247	0	NK/CD8 T cells	TM9SF3
0	0.59742595	0.326	0.162	0	NK/CD8 T cells	TBRG1
0	0.59302144	0.289	0.141	0	NK/CD8 T cells	CDC37
0	0.5921789	0.258	0.12	0	NK/CD8 T cells	AP1G2
0	0.59161824	0.489	0.259	0	NK/CD8 T cells	GSTK1
0	0.5907149	0.379	0.173	0	NK/CD8 T cells	ZDHHC20
0	0.59026054	0.998	0.912	0	NK/CD8 T cells	PFN1
0	0.58925704	0.876	0.572	0	NK/CD8 T cells	MYL12A
0	0.58873398	0.439	0.221	0	NK/CD8 T cells	SRP19
0	0.58854189	0.316	0.161	0	NK/CD8 T cells	TFAM
0	0.58561487	0.304	0.142	0	NK/CD8 T cells	CCNT1
0	0.58452894	0.58	0.305	0	NK/CD8 T cells	SMG1
0	0.58199538	0.455	0.232	0	NK/CD8 T cells	ANAPC16
0	0.58158467	0.307	0.148	0	NK/CD8 T cells	SMC4
0	0.58133939	0.343	0.176	0	NK/CD8 T cells	SRSF1
0	0.580405	0.34	0.161	0	NK/CD8 T cells	HECTD1
0	0.57685887	0.969	0.793	0	NK/CD8 T cells	ACTG1
0	0.57664679	0.337	0.125	0	NK/CD8 T cells	RHOH
0	0.57527937	0.371	0.184	0	NK/CD8 T cells	SEC63
0	0.57475789	0.389	0.193	0	NK/CD8 T cells	DENR
0	0.57412769	0.552	0.292	0	NK/CD8 T cells	MAT2B
0	0.57357086	0.596	0.3	0	NK/CD8 T cells	PIM1
0	0.57352321	0.499	0.261	0	NK/CD8 T cells	DDX46
0	0.57091302	0.303	0.15	0	NK/CD8 T cells	FAM111A
0	0.57023133	0.322	0.165	0	NK/CD8 T cells	PABPN1
0	0.5700032	0.39	0.21	0	NK/CD8 T cells	TOB1
0	0.56981488	0.395	0.194	0	NK/CD8 T cells	PBRM1
0	0.56883293	0.706	0.349	0	NK/CD8 T cells	APMAP
0	0.56656492	0.893	0.363	0	NK/CD8 T cells	NAP1L1
0	0.5662632	0.487	0.258	0	NK/CD8 T cells	COX4I1
0	0.56484035	0.311	0.121	0	NK/CD8 T cells	PCED1B
0	0.56334192	0.306	0.142	0	NK/CD8 T cells	QRICH1
0	0.56077308	0.322	0.165	0	NK/CD8 T cells	PSMD7
0	0.55886205	0.253	0.119	0	NK/CD8 T cells	CCDC82
0	0.55828098	0.465	0.229	0	NK/CD8 T cells	BBX
0	0.5578781	0.339	0.146	0	NK/CD8 T cells	C5orf56
0	0.55732903	0.436	0.218	0	NK/CD8 T cells	DNAJB14
0	0.55706669	0.418	0.188	0	NK/CD8 T cells	GPATCH8
0	0.55589657	0.566	0.297	0	NK/CD8 T cells	ZNF207
0	0.55586777	0.48	0.255	0	NK/CD8 T cells	RBBP6
0	0.55572926	0.568	0.305	0	NK/CD8 T cells	USP7

0	0.55350856	0.719	0.423	0	NK/CD8 T cells	PRPF38B
0	0.55298431	0.281	0.137	0	NK/CD8 T cells	SZRD1
0	0.5521768	0.869	0.57	0	NK/CD8 T cells	SON
0	0.55175855	0.552	0.239	0	NK/CD8 T cells	NFATC3
0	0.55081531	0.716	0.417	0	NK/CD8 T cells	DDX39B
0	0.54906687	0.312	0.148	0	NK/CD8 T cells	KPNA6
0	0.54844392	0.664	0.372	0	NK/CD8 T cells	KHDRBS1
0	0.54578897	0.475	0.255	0	NK/CD8 T cells	HNRNPUL2
0	0.54448362	0.922	0.59	0	NK/CD8 T cells	UBA52
0	0.54390628	0.268	0.109	0	NK/CD8 T cells	TRAPPC10
0	0.54348197	0.306	0.138	0	NK/CD8 T cells	PTGER4
0	0.5428888	0.296	0.137	0	NK/CD8 T cells	ZNF721
0	0.53809178	0.509	0.236	0	NK/CD8 T cells	XAF1
0	0.53781769	0.282	0.113	0	NK/CD8 T cells	TESPA1
0	0.53780189	0.407	0.203	0	NK/CD8 T cells	BRWD1
0	0.5373568	0.374	0.188	0	NK/CD8 T cells	CNOT2
0	0.53591738	0.549	0.301	0	NK/CD8 T cells	SRPRA
0	0.53386686	0.383	0.178	0	NK/CD8 T cells	PHC3
0	0.53279645	0.273	0.133	0	NK/CD8 T cells	KAT7
0	0.53222424	0.412	0.207	0	NK/CD8 T cells	ZFR
0	0.53187354	0.675	0.382	0	NK/CD8 T cells	SUMO2
0	0.53125299	0.33	0.169	0	NK/CD8 T cells	ALKBH5
0	0.52849175	0.607	0.339	0	NK/CD8 T cells	ARGLU1
0	0.5283168	0.347	0.185	0	NK/CD8 T cells	UQCR10
0	0.52811636	0.43	0.226	0	NK/CD8 T cells	EBLN3P
0	0.52810214	0.596	0.314	0	NK/CD8 T cells	MYCBP2
0	0.52707441	0.786	0.462	0	NK/CD8 T cells	GIMAP4
0	0.52620132	0.308	0.128	0	NK/CD8 T cells	TCF12
0	0.52500092	0.343	0.168	0	NK/CD8 T cells	ARPP19
0	0.52497788	0.433	0.234	0	NK/CD8 T cells	TRMT112
0	0.5245232	0.871	0.568	0	NK/CD8 T cells	SRSF5
0	0.52372188	0.588	0.323	0	NK/CD8 T cells	CIRBP
0	0.52368526	0.352	0.182	0	NK/CD8 T cells	CS
0	0.52331218	0.319	0.168	0	NK/CD8 T cells	HSPA4
0	0.52322471	0.518	0.287	0	NK/CD8 T cells	SKP1
0	0.5208761	0.342	0.18	0	NK/CD8 T cells	PCMTD2
0	0.52052849	0.305	0.151	0	NK/CD8 T cells	FAM168B
0	0.51829729	0.263	0.125	0	NK/CD8 T cells	PPP3CB
0	0.51731338	0.315	0.17	0	NK/CD8 T cells	FAM204A
0	0.51624466	0.385	0.207	0	NK/CD8 T cells	ATP5PF
0	0.51607336	0.278	0.13	0	NK/CD8 T cells	FAM53B
0	0.51560417	0.702	0.415	0	NK/CD8 T cells	ARHGEF1
0	0.51320293	0.651	0.37	0	NK/CD8 T cells	SUB1
0	0.51256441	0.263	0.131	0	NK/CD8 T cells	VIRMA
0	0.51179345	0.347	0.177	0	NK/CD8 T cells	PRPF8
0	0.51053678	0.57	0.32	0	NK/CD8 T cells	TSPAN14
0	0.51017048	0.482	0.255	0	NK/CD8 T cells	NCKAP1L
0	0.50965764	0.254	0.111	0	NK/CD8 T cells	NPIPB5
0	0.5095402	0.427	0.213	0	NK/CD8 T cells	PTPN1
0	0.50850898	0.339	0.184	0	NK/CD8 T cells	CNOT7

0	0.50765721	0.522	0.263	0	NK/CD8 T cells	VOPP1
0	0.50712677	0.447	0.234	0	NK/CD8 T cells	UBE2I
0	0.50648803	0.483	0.248	0	NK/CD8 T cells	HCP5
0	0.50496813	0.506	0.243	0	NK/CD8 T cells	STK17A
0	0.50046827	0.546	0.297	0	NK/CD8 T cells	NKTR
0	0.49958771	0.607	0.338	0	NK/CD8 T cells	WDR82
0	0.49914099	0.387	0.208	0	NK/CD8 T cells	CWC15
0	0.49776714	0.265	0.131	0	NK/CD8 T cells	FAM217B
0	0.49756471	0.397	0.216	0	NK/CD8 T cells	FXR1
0	0.49709745	0.972	0.777	0	NK/CD8 T cells	YWHAZ
0	0.49660887	0.449	0.243	0	NK/CD8 T cells	ATP5MF
0	0.49530546	0.633	0.349	0	NK/CD8 T cells	SMARCA2
0	0.49483673	0.418	0.234	0	NK/CD8 T cells	ATP5PD
0	0.49444335	0.751	0.459	0	NK/CD8 T cells	HNRNPUL1
0	0.49117421	0.489	0.255	0	NK/CD8 T cells	KIAA2026
0	0.49090702	0.541	0.28	0	NK/CD8 T cells	GLG1
0	0.48987027	0.307	0.142	0	NK/CD8 T cells	PIK3IP1
0	0.48909181	0.334	0.167	0	NK/CD8 T cells	RAB22A
0	0.48728676	0.499	0.265	0	NK/CD8 T cells	CD300A
0	0.48666128	0.773	0.46	0	NK/CD8 T cells	FAU
0	0.48591836	0.273	0.136	0	NK/CD8 T cells	MYSM1
0	0.48545449	0.305	0.16	0	NK/CD8 T cells	DDX42
0	0.48450379	0.356	0.184	0	NK/CD8 T cells	CMTM3
0	0.48173252	0.387	0.206	0	NK/CD8 T cells	CCNL2
0	0.48043875	0.446	0.222	0	NK/CD8 T cells	KAT2B
0	0.47866044	0.411	0.181	0	NK/CD8 T cells	SYTL3
0	0.4783015	0.363	0.193	0	NK/CD8 T cells	CPNE3
0	0.4771408	0.299	0.151	0	NK/CD8 T cells	VPS26A
0	0.47658586	0.47	0.242	0	NK/CD8 T cells	SOS1
0	0.47569427	0.323	0.174	0	NK/CD8 T cells	LGALS8
0	0.47533211	0.284	0.14	0	NK/CD8 T cells	PRKAA1
0	0.47351646	0.93	0.663	0	NK/CD8 T cells	HMGB1
0	0.47151524	0.424	0.233	0	NK/CD8 T cells	PSMA7
0	0.46871376	0.348	0.182	0	NK/CD8 T cells	SECISBP2L
0	0.46834912	0.461	0.25	0	NK/CD8 T cells	DR1
0	0.46818213	0.294	0.144	0	NK/CD8 T cells	KLHL28
0	0.46651977	0.426	0.23	0	NK/CD8 T cells	TNKS2
0	0.46541271	0.422	0.237	0	NK/CD8 T cells	SLC38A2
0	0.46527256	0.41	0.213	0	NK/CD8 T cells	CNTRL
0	0.46316361	0.488	0.27	0	NK/CD8 T cells	FGFR1OP2
0	0.4630195	0.278	0.139	0	NK/CD8 T cells	LEMD3
0	0.46277134	0.606	0.349	0	NK/CD8 T cells	EIF4H
0	0.46025732	0.272	0.125	0	NK/CD8 T cells	KDM4C
0	0.45853289	0.371	0.208	0	NK/CD8 T cells	HADHA
0	0.45834822	0.405	0.215	0	NK/CD8 T cells	STT3B
0	0.4558995	0.256	0.125	0	NK/CD8 T cells	POM121C
0	0.45558175	0.346	0.165	0	NK/CD8 T cells	TBC1D10C
0	0.45477024	0.369	0.196	0	NK/CD8 T cells	RBM27
0	0.45317498	0.512	0.289	0	NK/CD8 T cells	SSR1
0	0.45137228	0.986	0.734	0	NK/CD8 T cells	PABPC1

0	0.45112473	0.381	0.193	0	NK/CD8 T cells	RNF19A
0	0.44998355	0.271	0.133	0	NK/CD8 T cells	PITPNB
0	0.44929312	0.257	0.103	0	NK/CD8 T cells	PLCB1
0	0.44719162	0.274	0.137	0	NK/CD8 T cells	CNOT4
0	0.44700591	0.31	0.155	0	NK/CD8 T cells	MAPKAP1
0	0.4455977	0.68	0.384	0	NK/CD8 T cells	PFDN5
0	0.44303288	0.264	0.124	0	NK/CD8 T cells	DENND6A
0	0.44264697	0.318	0.164	0	NK/CD8 T cells	PRKAR2A
0	0.44207847	0.403	0.214	0	NK/CD8 T cells	RBM26
0	0.44147183	0.257	0.125	0	NK/CD8 T cells	TRRAP
0	0.44097893	0.752	0.458	0	NK/CD8 T cells	BPTF
0	0.44042897	0.501	0.292	0	NK/CD8 T cells	CDV3
0	0.4366902	0.782	0.494	0	NK/CD8 T cells	STK38
0	0.43654161	0.344	0.185	0	NK/CD8 T cells	SLAIN2
0	0.43541903	0.626	0.356	0	NK/CD8 T cells	CTSC
0	0.4331247	0.509	0.296	0	NK/CD8 T cells	U2AF2
0	0.43278157	0.442	0.25	0	NK/CD8 T cells	SRSF9
0	0.42966462	0.804	0.489	0	NK/CD8 T cells	IKZF1
0	0.42852077	0.295	0.145	0	NK/CD8 T cells	PKN1
0	0.42753624	0.51	0.283	0	NK/CD8 T cells	GCC2
0	0.42670942	0.253	0.107	0	NK/CD8 T cells	SCML4
0	0.42478448	0.4	0.226	0	NK/CD8 T cells	DDX3Y
0	0.42466497	0.305	0.141	0	NK/CD8 T cells	RPRD2
0	0.42317024	0.525	0.304	0	NK/CD8 T cells	YME1L1
0	0.42112955	0.302	0.139	0	NK/CD8 T cells	JAZF1
0	0.4177891	0.269	0.128	0	NK/CD8 T cells	NOL4L
0	0.41770626	0.313	0.165	0	NK/CD8 T cells	DRAP1
0	0.4170879	0.292	0.147	0	NK/CD8 T cells	ADIPOR2
0	0.41691923	0.343	0.185	0	NK/CD8 T cells	PURB
0	0.41682149	0.775	0.473	0	NK/CD8 T cells	SERF2
0	0.41281454	0.395	0.222	0	NK/CD8 T cells	SCAMP2
0	0.41224644	0.501	0.275	0	NK/CD8 T cells	PUM1
0	0.40896529	0.657	0.39	0	NK/CD8 T cells	ADD1
0	0.40865699	0.578	0.313	0	NK/CD8 T cells	TRIM22
0	0.40774929	0.723	0.447	0	NK/CD8 T cells	DDX6
0	0.4044932	0.281	0.138	0	NK/CD8 T cells	SASH3
0	0.40287702	0.553	0.247	0	NK/CD8 T cells	PITPNC1
0	0.40216802	0.591	0.352	0	NK/CD8 T cells	SRP14
0	0.4012507	0.554	0.319	0	NK/CD8 T cells	PSIP1
0	0.40088437	0.994	0.897	0	NK/CD8 T cells	CFL1
0	0.40064269	0.493	0.243	0	NK/CD8 T cells	ACAP1
0	0.40038817	0.444	0.256	0	NK/CD8 T cells	PSMB1
0	0.40010201	0.336	0.158	0	NK/CD8 T cells	SP140
0	0.39859711	0.529	0.306	0	NK/CD8 T cells	PPP4R3B
0	0.39830065	0.484	0.274	0	NK/CD8 T cells	CNOT1
0	0.39805221	0.564	0.301	0	NK/CD8 T cells	BIRC6
0	0.39571058	0.262	0.131	0	NK/CD8 T cells	SNX14
0	0.39542678	0.342	0.183	0	NK/CD8 T cells	MED4
0	0.3939152	0.558	0.324	0	NK/CD8 T cells	CEP350
0	0.38875409	0.472	0.267	0	NK/CD8 T cells	PHF11

0	0.3880432	0.575	0.331	0	NK/CD8 T cells	B4GALT1
0	0.38304842	0.708	0.443	0	NK/CD8 T cells	ATRX
0	0.3794314	0.539	0.323	0	NK/CD8 T cells	EIF4EBP2
0	0.37926717	0.816	0.552	0	NK/CD8 T cells	HNRNPU
0	0.36962007	0.288	0.131	0	NK/CD8 T cells	OXNAD1
0	0.36939177	0.615	0.37	0	NK/CD8 T cells	CBX3
0	0.36780949	0.39	0.218	0	NK/CD8 T cells	SBNO1
0	0.36640163	0.519	0.308	0	NK/CD8 T cells	SAP18
0	0.36245851	0.53	0.292	0	NK/CD8 T cells	RASA2
0	0.36159263	0.518	0.301	0	NK/CD8 T cells	UBE2Q1
0	0.36017807	0.453	0.253	0	NK/CD8 T cells	CPNE1
0	0.35955637	0.782	0.516	0	NK/CD8 T cells	VAMP2
0	0.3551809	0.533	0.309	0	NK/CD8 T cells	NUB1
0	0.35503984	0.709	0.425	0	NK/CD8 T cells	ZC3HAV1
0	0.35469923	0.43	0.225	0	NK/CD8 T cells	FRYL
0	0.35074447	0.386	0.214	0	NK/CD8 T cells	PCGF5
0	0.34767375	0.544	0.326	0	NK/CD8 T cells	EIF5
0	0.34687449	0.753	0.494	0	NK/CD8 T cells	BCLAF1
0	0.34437549	0.443	0.233	0	NK/CD8 T cells	ST3GAL1
0	0.34359919	0.66	0.383	0	NK/CD8 T cells	CD44
0	0.3427058	0.85	0.593	0	NK/CD8 T cells	PRRC2C
0	0.34034298	0.34	0.172	0	NK/CD8 T cells	RABEP1
0	0.32831917	0.328	0.166	0	NK/CD8 T cells	RAB6A
0	0.3278294	0.592	0.363	0	NK/CD8 T cells	EWSR1
0	0.32689906	0.762	0.494	0	NK/CD8 T cells	HLA-F
0	0.30864189	0.629	0.379	0	NK/CD8 T cells	LSM14A
0	0.30640399	0.298	0.151	0	NK/CD8 T cells	ILRUN
0	0.30486939	0.722	0.462	0	NK/CD8 T cells	AKNA
0	0.29988666	0.611	0.368	0	NK/CD8 T cells	ATF7IP
0	0.29886183	0.336	0.176	0	NK/CD8 T cells	PSME4
0	0.29676987	0.947	0.699	0	NK/CD8 T cells	MBNL1
0	0.2966873	0.43	0.244	0	NK/CD8 T cells	GALNT1
0	0.29365283	0.316	0.147	0	NK/CD8 T cells	DENND4A
0	0.29185811	0.611	0.336	0	NK/CD8 T cells	TNFAIP8
0	0.28423319	0.697	0.443	0	NK/CD8 T cells	TRIR
0	0.28012112	0.51	0.296	0	NK/CD8 T cells	HERPUD2
0	0.27295454	0.455	0.26	0	NK/CD8 T cells	SMARCC1
0	0.26773081	0.369	0.18	0	NK/CD8 T cells	ADA2
0	0.25473147	0.785	0.513	0	NK/CD8 T cells	FNBP1
2.301E-307	0.25827549	0.696	0.463	7.719E-303	NK/CD8 T cells	RBM25
6.87E-306	0.38794582	0.311	0.164	2.304E-301	NK/CD8 T cells	TAF1
1.071E-305	0.36036081	0.413	0.238	3.592E-301	NK/CD8 T cells	TAF1D
4.946E-304	0.55487147	0.269	0.137	1.659E-299	NK/CD8 T cells	RRAGA
1.994E-303	0.43177941	0.281	0.145	6.689E-299	NK/CD8 T cells	YLPM1
2.789E-303	0.39958643	0.399	0.23	9.354E-299	NK/CD8 T cells	ZFP91
3.942E-303	0.39726235	0.366	0.206	1.322E-298	NK/CD8 T cells	MTIF3
3.975E-302	0.25883083	0.614	0.395	1.333E-297	NK/CD8 T cells	SLTM
4.805E-302	0.31972436	0.47	0.275	1.612E-297	NK/CD8 T cells	HDGF
1.254E-301	0.33837457	0.282	0.146	4.207E-297	NK/CD8 T cells	AMBRA1
1.695E-301	0.56253664	0.273	0.141	5.684E-297	NK/CD8 T cells	COX7A2

2.977E-301	0.3890814	0.398	0.228	9.987E-297	NK/CD8 T cells	MAPRE1
8.838E-301	0.68797935	0.259	0.133	2.964E-296	NK/CD8 T cells	ZNF706
2.315E-300	0.30309803	0.358	0.198	7.766E-296	NK/CD8 T cells	ADNP
7.178E-300	0.35997747	0.321	0.173	2.408E-295	NK/CD8 T cells	DCAF8
6.11E-298	0.38103781	0.38	0.215	2.05E-293	NK/CD8 T cells	PSMA4
1.93E-297	0.46255262	0.253	0.126	6.473E-293	NK/CD8 T cells	CSNK1G3
4.077E-297	0.4601939	0.296	0.156	1.367E-292	NK/CD8 T cells	ARCN1
1.409E-296	0.43026469	0.279	0.145	4.726E-292	NK/CD8 T cells	DAP3
1.429E-296	0.57240483	0.251	0.126	4.794E-292	NK/CD8 T cells	THOC7
7.363E-296	0.43672429	0.286	0.149	2.47E-291	NK/CD8 T cells	TINF2
1.419E-295	0.27030593	0.271	0.137	4.76E-291	NK/CD8 T cells	CDC14A
1.647E-295	0.41737577	0.28	0.145	5.524E-291	NK/CD8 T cells	FIP1L1
3.039E-295	0.44751015	0.345	0.193	1.019E-290	NK/CD8 T cells	LARP7
3.901E-293	0.26597613	0.914	0.738	1.308E-288	NK/CD8 T cells	JAK1
2.956E-292	0.35313513	0.357	0.2	9.915E-288	NK/CD8 T cells	CDK12
6.505E-291	0.29585197	0.481	0.287	2.182E-286	NK/CD8 T cells	COPA
2.43E-290	0.35642213	0.263	0.133	8.151E-286	NK/CD8 T cells	CDC27
3.254E-290	0.35204821	0.433	0.255	1.092E-285	NK/CD8 T cells	BRK1
1.146E-289	0.52596846	0.298	0.159	3.842E-285	NK/CD8 T cells	ATP6V1F
1.841E-289	0.31865535	0.388	0.22	6.175E-285	NK/CD8 T cells	UBLCP1
1.664E-286	0.27585934	0.826	0.567	5.581E-282	NK/CD8 T cells	EIF4G2
3.159E-284	0.46404091	0.266	0.135	1.06E-279	NK/CD8 T cells	DNAJB1
4.794E-284	0.46423665	0.257	0.131	1.608E-279	NK/CD8 T cells	TIPRL
2.337E-282	0.27422873	0.947	0.726	7.84E-278	NK/CD8 T cells	YWHAB
6.289E-280	0.2964446	0.465	0.281	2.11E-275	NK/CD8 T cells	SELENOF
1.492E-279	0.32694737	0.259	0.132	5.006E-275	NK/CD8 T cells	MED15
1.949E-278	0.25235414	0.545	0.339	6.538E-274	NK/CD8 T cells	CCDC69
9.55E-278	0.3350184	0.472	0.285	3.203E-273	NK/CD8 T cells	CSNK2B
7.083E-277	0.47882614	0.273	0.143	2.376E-272	NK/CD8 T cells	PDAP1
1.125E-276	0.25372278	0.374	0.214	3.775E-272	NK/CD8 T cells	TNPO1
1.619E-276	0.35368346	0.271	0.141	5.432E-272	NK/CD8 T cells	SUPT5H
2.049E-276	0.29584255	0.443	0.264	6.872E-272	NK/CD8 T cells	ATF4
2.941E-275	0.34696876	0.298	0.161	9.866E-271	NK/CD8 T cells	GOLPH3
2.275E-274	0.31959824	0.369	0.211	7.63E-270	NK/CD8 T cells	GPS2
5.234E-274	0.36624646	0.256	0.131	1.756E-269	NK/CD8 T cells	INO80D
8.97E-274	0.33352263	0.334	0.185	3.009E-269	NK/CD8 T cells	SPTY2D1
1.496E-273	0.41438392	0.278	0.148	5.017E-269	NK/CD8 T cells	TOPBP1
6.919E-272	0.31042985	0.346	0.195	2.321E-267	NK/CD8 T cells	C1GALT1
3.766E-271	0.26787558	0.485	0.297	1.263E-266	NK/CD8 T cells	HIPK1
3.732E-270	0.52865436	0.252	0.133	1.252E-265	NK/CD8 T cells	MCUB
9.271E-270	0.34106367	0.376	0.217	3.11E-265	NK/CD8 T cells	POMP
9.823E-270	0.49979172	0.282	0.152	3.295E-265	NK/CD8 T cells	SNHG16
1.568E-269	0.26578699	0.474	0.289	5.259E-265	NK/CD8 T cells	RBM4
7.028E-266	0.35009236	0.286	0.152	2.358E-261	NK/CD8 T cells	ESCO1
8.405E-266	0.3654476	0.295	0.16	2.819E-261	NK/CD8 T cells	ZC3H18
1.224E-265	0.42849527	0.288	0.156	4.107E-261	NK/CD8 T cells	MFNG
2.431E-265	0.31540777	0.381	0.223	8.155E-261	NK/CD8 T cells	CRYBG1
2.46E-265	0.3844911	0.284	0.153	8.252E-261	NK/CD8 T cells	UBQLN1
9.982E-265	0.25402454	0.747	0.511	3.348E-260	NK/CD8 T cells	C6orf62
1.434E-263	0.51271133	0.252	0.132	4.809E-259	NK/CD8 T cells	GABPA

1.767E-263	0.31087116	0.348	0.197	5.926E-259	NK/CD8 T cells	PPP1CA
5.633E-263	0.3745362	0.304	0.168	1.89E-258	NK/CD8 T cells	AP3D1
1.013E-261	0.38485102	0.308	0.173	3.398E-257	NK/CD8 T cells	MAN2A1
1.601E-259	0.27016444	0.262	0.136	5.37E-255	NK/CD8 T cells	RNF168
2.688E-259	0.30943413	0.287	0.155	9.018E-255	NK/CD8 T cells	RALGAPB
9.96E-259	0.4293997	0.28	0.153	3.341E-254	NK/CD8 T cells	CAPRIN1
2.554E-258	0.27505583	0.475	0.294	8.567E-254	NK/CD8 T cells	RSRC2
9.357E-258	0.33502174	0.256	0.134	3.139E-253	NK/CD8 T cells	DCP1A
4.272E-257	0.56517697	0.263	0.142	1.433E-252	NK/CD8 T cells	KDELR1
1.699E-256	0.30364901	0.44	0.267	5.698E-252	NK/CD8 T cells	PSMB4
1.757E-256	0.37611733	0.254	0.133	5.894E-252	NK/CD8 T cells	PRPF3
9.809E-256	0.41422193	0.263	0.141	3.29E-251	NK/CD8 T cells	NDUFS1
5.612E-254	0.32963116	0.432	0.262	1.882E-249	NK/CD8 T cells	COX6A1
4.317E-253	0.44640333	0.251	0.131	1.448E-248	NK/CD8 T cells	CBLL1
1.521E-248	0.29977449	0.287	0.156	5.1E-244	NK/CD8 T cells	SIN3A
2.817E-247	0.33479178	0.276	0.151	9.45E-243	NK/CD8 T cells	WDFY1
8.54E-247	0.33347841	0.305	0.171	2.865E-242	NK/CD8 T cells	AKT2
1.415E-246	0.29378101	0.31	0.175	4.746E-242	NK/CD8 T cells	SFSWAP
3.677E-246	0.33420404	0.321	0.183	1.233E-241	NK/CD8 T cells	PTAR1
8.739E-246	0.47009875	0.277	0.153	2.931E-241	NK/CD8 T cells	CCT5
9.245E-246	0.2975294	0.256	0.134	3.101E-241	NK/CD8 T cells	IDI1
8.062E-243	0.29256732	0.324	0.184	2.704E-238	NK/CD8 T cells	IRAK4
8.886E-243	0.4219514	0.306	0.175	2.981E-238	NK/CD8 T cells	CPSF2
9.823E-242	0.31605881	0.331	0.19	3.295E-237	NK/CD8 T cells	CHTOP
2.324E-241	0.28674484	0.286	0.159	7.795E-237	NK/CD8 T cells	SMAP1
2.861E-240	0.44445555	0.255	0.138	9.596E-236	NK/CD8 T cells	UQCRC2
2.168E-239	0.39590709	0.269	0.148	7.273E-235	NK/CD8 T cells	MAVS
2.261E-239	0.29468683	0.262	0.141	7.584E-235	NK/CD8 T cells	DCAF10
5.608E-238	0.285556	0.368	0.219	1.881E-233	NK/CD8 T cells	SNW1
6.246E-238	0.37267139	0.267	0.147	2.095E-233	NK/CD8 T cells	DNAJC7
3.524E-237	0.267181	0.375	0.225	1.182E-232	NK/CD8 T cells	PSMF1
5.414E-236	0.33218494	0.357	0.211	1.816E-231	NK/CD8 T cells	PRKCSH
4.523E-235	0.3168493	0.351	0.207	1.517E-230	NK/CD8 T cells	DAD1
3.156E-231	0.36477029	0.337	0.199	1.058E-226	NK/CD8 T cells	BAX
6.401E-230	0.27900959	0.285	0.16	2.147E-225	NK/CD8 T cells	IREB2
3.805E-229	0.42851827	0.286	0.162	1.276E-224	NK/CD8 T cells	ZBED5
9.86E-229	0.27215065	0.289	0.162	3.307E-224	NK/CD8 T cells	SRP54
1.203E-228	0.26077907	0.261	0.142	4.035E-224	NK/CD8 T cells	WDR33
5.273E-226	0.42997476	0.253	0.138	1.769E-221	NK/CD8 T cells	LTN1
1.673E-222	0.31848567	0.303	0.176	5.61E-218	NK/CD8 T cells	FBXO9
1.945E-220	0.27000119	0.324	0.19	6.524E-216	NK/CD8 T cells	LMO4
1.539E-219	0.37267135	0.294	0.17	5.162E-215	NK/CD8 T cells	CCDC59
1.667E-219	0.32144235	0.279	0.157	5.593E-215	NK/CD8 T cells	ATF6B
3.593E-219	0.32404715	0.282	0.159	1.205E-214	NK/CD8 T cells	GNL3L
7.154E-219	0.28118572	0.275	0.157	2.4E-214	NK/CD8 T cells	MAP2K1
1.811E-218	0.37744648	0.268	0.151	6.074E-214	NK/CD8 T cells	GPATCH2L
2.455E-217	0.29832129	0.333	0.198	8.236E-213	NK/CD8 T cells	ARL8B
3.577E-217	0.30887742	0.278	0.157	1.2E-212	NK/CD8 T cells	CCNT2
3.212E-211	0.33020491	0.286	0.164	1.077E-206	NK/CD8 T cells	MON1B
1.177E-210	0.26610222	0.319	0.188	3.95E-206	NK/CD8 T cells	USF2

7.001E-210	0.32213427	0.258	0.144	2.348E-205	NK/CD8 T cells	FDFT1
2.154E-208	0.31469406	0.384	0.236	7.224E-204	NK/CD8 T cells	COX8A
1.223E-206	0.27739631	0.259	0.145	4.103E-202	NK/CD8 T cells	DENND4B
3.956E-203	0.34877774	0.327	0.197	1.327E-198	NK/CD8 T cells	OXA1L
4.773E-203	0.30683505	0.293	0.17	1.601E-198	NK/CD8 T cells	SEPHS2
1.627E-202	0.31826657	0.288	0.168	5.458E-198	NK/CD8 T cells	DNAJA2
4.495E-202	0.40380693	0.263	0.151	1.508E-197	NK/CD8 T cells	MRPL33
3.121E-201	0.40259307	0.32	0.191	1.047E-196	NK/CD8 T cells	C1orf162
2.776E-200	0.28683903	0.332	0.2	9.312E-196	NK/CD8 T cells	MIA3
1.214E-199	0.26892816	0.25	0.141	4.071E-195	NK/CD8 T cells	MIB1
3.173E-198	0.26433894	0.29	0.171	1.064E-193	NK/CD8 T cells	PRDX6
6.873E-198	0.28143682	0.254	0.144	2.305E-193	NK/CD8 T cells	TSNAX
1.338E-193	0.28980267	0.251	0.14	4.489E-189	NK/CD8 T cells	TBCC
2.611E-191	0.27221649	0.338	0.207	8.757E-187	NK/CD8 T cells	CTDNEP1
8.993E-191	0.25458682	0.336	0.205	3.016E-186	NK/CD8 T cells	SEC22B
7.318E-189	0.27004279	0.313	0.19	2.455E-184	NK/CD8 T cells	CLTA
1.405E-186	0.29848288	0.263	0.153	4.714E-182	NK/CD8 T cells	HPS1
1.994E-185	0.30159127	0.252	0.145	6.69E-181	NK/CD8 T cells	CAMLG
8.84E-179	0.27219557	0.271	0.161	2.965E-174	NK/CD8 T cells	SNAP29
1.408E-166	0.2816536	0.279	0.17	4.723E-162	NK/CD8 T cells	CDC40
0	6.87458716	0.993	0.024	0	B cells	MS4A1
0	6.81662071	0.885	0.019	0	B cells	IGHM
0	6.47737187	0.989	0.016	0	B cells	CD79A
0	6.38413392	0.798	0.008	0	B cells	IGHD
0	5.19067042	0.926	0.033	0	B cells	HLA-DQA1
0	5.13545092	0.811	0.01	0	B cells	CD22
0	4.90328702	0.805	0.01	0	B cells	PAX5
0	4.8650428	0.95	0.033	0	B cells	BANK1
0	4.80405529	0.826	0.02	0	B cells	NIBAN3
0	4.65849314	0.755	0.022	0	B cells	FCRL1
0	4.60036218	0.726	0.04	0	B cells	IGKC
0	4.46144835	0.596	0.002	0	B cells	FCRLA
0	4.42118534	0.665	0.011	0	B cells	CD79B
0	4.32829804	0.608	0.007	0	B cells	LINC00926
0	4.25010051	0.485	0.006	0	B cells	FCER2
0	4.24169265	0.574	0.004	0	B cells	TNFRSF13C
0	4.23373062	0.648	0.01	0	B cells	HLA-DOB
0	4.1426286	0.647	0.02	0	B cells	P2RX5
0	4.05363509	0.375	0.002	0	B cells	TCL1A
0	4.05026436	0.491	0.004	0	B cells	LINC02397
0	4.03173501	0.602	0.017	0	B cells	HLA-DOA
0	4.01718595	0.503	0.004	0	B cells	SPIB
0	4.01458199	0.777	0.05	0	B cells	HLA-DQB1
0	3.93002059	0.967	0.12	0	B cells	HLA-DPB1
0	3.87800801	1	0.493	0	B cells	CD74
0	3.85428964	0.429	0.001	0	B cells	VPREB3
0	3.83781312	0.457	0.009	0	B cells	COBLL1
0	3.83475789	0.992	0.139	0	B cells	HLA-DPA1
0	3.82072388	0.506	0.006	0	B cells	POU2AF1
0	3.79876925	0.998	0.163	0	B cells	HLA-DRA

0	3.78643747	0.34	0.005	0	B cells	FCRL5
0	3.7735316	0.541	0.036	0	B cells	PLPP5
0	3.71680008	0.888	0.129	0	B cells	BIRC3
0	3.69047087	0.481	0.015	0	B cells	CD180
0	3.67236833	0.623	0.037	0	B cells	TCF4
0	3.64874711	0.407	0.003	0	B cells	SNX22
0	3.64147631	0.814	0.066	0	B cells	SWAP70
0	3.63411833	0.366	0.008	0	B cells	COL19A1
0	3.62272748	0.672	0.04	0	B cells	BCL11A
0	3.62131381	0.463	0.009	0	B cells	BLNK
0	3.61027334	0.588	0.031	0	B cells	PALM2-AKAP2
0	3.58738483	0.413	0.006	0	B cells	FCRL2
0	3.54352402	0.498	0.01	0	B cells	BLK
0	3.5418404	0.989	0.143	0	B cells	HLA-DRB1
0	3.53468021	0.512	0.026	0	B cells	BTLA
0	3.50511737	0.88	0.099	0	B cells	RALGPS2
0	3.48353472	0.459	0.012	0	B cells	STAP1
0	3.47178247	0.561	0.032	0	B cells	CCR6
0	3.4468333	0.498	0.021	0	B cells	HLA-DQA2
0	3.44646605	0.554	0.015	0	B cells	CD24
0	3.44185871	0.384	0.003	0	B cells	CD19
0	3.42298464	0.5	0.025	0	B cells	RAB30
0	3.40796558	0.686	0.064	0	B cells	IRF8
0	3.37026844	0.832	0.094	0	B cells	HLA-DMB
0	3.35195883	0.728	0.066	0	B cells	SEL1L3
0	3.35037291	0.261	0.012	0	B cells	PCDH9
0	3.34474618	0.439	0.034	0	B cells	FCRL3
0	3.33934732	0.586	0.042	0	B cells	ADAM28
0	3.30250367	0.395	0.012	0	B cells	CD40
0	3.29286007	0.601	0.05	0	B cells	CXXC5
0	3.29278386	0.368	0.013	0	B cells	BCL7A
0	3.29135132	0.372	0.013	0	B cells	RHEX
0	3.24936017	0.354	0.007	0	B cells	CPNE5
0	3.24527151	0.362	0.007	0	B cells	CXCR5
0	3.24118444	0.458	0.035	0	B cells	CCDC50
0	3.23751593	0.392	0.011	0	B cells	EBF1
0	3.22307747	0.351	0.007	0	B cells	TSPAN13
0	3.19305539	0.69	0.049	0	B cells	AFF3
0	3.0798405	0.579	0.048	0	B cells	STRBP
0	3.06495201	0.328	0.009	0	B cells	CD72
0	3.05590271	0.293	0.006	0	B cells	IGLC2
0	3.03474926	0.322	0.019	0	B cells	KMO
0	3.02376906	0.337	0.01	0	B cells	RASGRP3
0	3.00924535	0.377	0.014	0	B cells	OSBPL10
0	2.99004327	0.497	0.052	0	B cells	HLA-DRB5
0	2.9873458	0.347	0.014	0	B cells	PLEKHG1
0	2.96775926	0.452	0.039	0	B cells	IFT57
0	2.95499323	0.508	0.038	0	B cells	TPD52
0	2.94510487	0.433	0.037	0	B cells	TSPAN3
0	2.92388599	0.36	0.023	0	B cells	SERPINB9P1

0	2.92065331	0.569	0.055	0	B cells	CIITA
0	2.91574664	0.281	0.005	0	B cells	PNOC
0	2.87699912	0.332	0.014	0	B cells	DENND5B
0	2.85032372	0.934	0.21	0	B cells	FCMR
0	2.84231738	0.35	0.019	0	B cells	CDCA7L
0	2.83312966	0.274	0.008	0	B cells	PAWR
0	2.82707052	0.419	0.035	0	B cells	QRSL1
0	2.80207374	0.406	0.013	0	B cells	PDLIM1
0	2.78513027	0.288	0.007	0	B cells	PKIG
0	2.70249444	0.363	0.03	0	B cells	SAV1
0	2.65473017	0.652	0.111	0	B cells	PPM1K
0	2.65204068	0.346	0.038	0	B cells	FAM3C
0	2.62688627	0.543	0.069	0	B cells	GGA2
0	2.62685757	0.507	0.051	0	B cells	TLR10
0	2.6121536	0.373	0.049	0	B cells	TBC1D9
0	2.55613097	0.525	0.077	0	B cells	PARP15
0	2.54138566	0.367	0.049	0	B cells	AGPAT5
0	2.53517329	0.857	0.168	0	B cells	IKZF3
0	2.52412957	0.418	0.054	0	B cells	TCF3
0	2.51496355	0.289	0.025	0	B cells	NT5E
0	2.51257592	0.646	0.111	0	B cells	BACH2
0	2.4947979	0.747	0.125	0	B cells	MARCH1
0	2.42970333	0.456	0.061	0	B cells	TRAF5
0	2.42895414	0.427	0.07	0	B cells	LY86
0	2.42260679	0.622	0.121	0	B cells	PARP1
0	2.40899229	0.635	0.104	0	B cells	HLA-DMA
0	2.39631522	0.266	0.021	0	B cells	SLC9A7
0	2.3960486	0.285	0.024	0	B cells	SETBP1
0	2.39231999	0.758	0.162	0	B cells	MEF2C
0	2.39064729	0.364	0.041	0	B cells	CYB561A3
0	2.37802039	0.518	0.105	0	B cells	DNAJC10
0	2.37513121	0.405	0.072	0	B cells	ZNF318
0	2.35117355	0.501	0.08	0	B cells	FGD2
0	2.33989507	0.533	0.097	0	B cells	SMC6
0	2.32901864	0.313	0.034	0	B cells	LINC01215
0	2.30475825	0.986	0.321	0	B cells	CD52
0	2.30340475	0.398	0.069	0	B cells	ZNF92
0	2.29889486	0.525	0.086	0	B cells	METTL7A
0	2.27643309	0.281	0.034	0	B cells	METTL8
0	2.26128148	0.293	0.012	0	B cells	JCHAIN
0	2.23761681	0.752	0.188	0	B cells	EZR
0	2.20762713	0.337	0.041	0	B cells	USP6NL
0	2.19035703	0.323	0.043	0	B cells	CLIC4
0	2.16579551	0.276	0.043	0	B cells	OPN3
0	2.16330914	0.553	0.129	0	B cells	APPL1
0	2.16303913	0.353	0.073	0	B cells	SCIMP
0	2.14709452	0.458	0.071	0	B cells	MTSS1
0	2.13107873	0.604	0.147	0	B cells	AIDA
0	2.12917567	0.547	0.109	0	B cells	ARHGAP17
0	2.12487389	0.26	0.045	0	B cells	TTN

0	2.10570438	0.281	0.043	0	B cells	TMEM19
0	2.10283518	0.632	0.17	0	B cells	SMIM14
0	2.10059617	0.829	0.255	0	B cells	HVCN1
0	2.08025541	0.299	0.039	0	B cells	EAF2
0	2.07111441	0.42	0.078	0	B cells	NUP88
0	2.07045656	0.389	0.046	0	B cells	TMEM156
0	2.06152078	0.505	0.108	0	B cells	CAMK2D
0	2.01131436	0.491	0.098	0	B cells	PLEKHF2
0	2.00353866	0.344	0.071	0	B cells	SNHG7
0	2.00337939	0.406	0.082	0	B cells	CD81
0	2.00288039	0.713	0.188	0	B cells	SNX2
0	1.98766935	0.277	0.047	0	B cells	GNA12
0	1.97943378	0.26	0.031	0	B cells	BCAS4
0	1.96934245	0.262	0.045	0	B cells	SHMT2
0	1.96858793	0.279	0.042	0	B cells	VEGFB
0	1.94958248	0.43	0.084	0	B cells	ARID5B
0	1.94243278	0.958	0.319	0	B cells	RPL18A
0	1.92752208	0.98	0.54	0	B cells	CD37
0	1.92064277	0.486	0.095	0	B cells	SLAMF6
0	1.91841243	0.253	0.045	0	B cells	NOC3L
0	1.91641401	0.448	0.103	0	B cells	DRAM2
0	1.90874177	0.465	0.096	0	B cells	ITPR1
0	1.90686702	0.467	0.068	0	B cells	GNG7
0	1.90460262	0.586	0.146	0	B cells	APOBEC3C
0	1.90447679	0.383	0.084	0	B cells	MBNL3
0	1.89635013	0.697	0.186	0	B cells	PLAC8
0	1.8943297	0.403	0.074	0	B cells	HDAC9
0	1.88887652	0.974	0.34	0	B cells	RPS5
0	1.88789444	0.313	0.053	0	B cells	TRIO
0	1.88132623	0.689	0.147	0	B cells	P2RY10
0	1.87312238	0.996	0.385	0	B cells	RPS23
0	1.86201854	0.995	0.522	0	B cells	RPS11
0	1.8602412	0.796	0.236	0	B cells	POU2F2
0	1.85054997	0.734	0.178	0	B cells	LBH
0	1.8479346	0.503	0.106	0	B cells	BTK
0	1.84382271	0.999	0.45	0	B cells	RPS8
0	1.84257791	0.355	0.089	0	B cells	GLO1
0	1.83643366	0.949	0.337	0	B cells	RPL8
0	1.83565804	0.351	0.029	0	B cells	CNR2
0	1.82737019	0.267	0.043	0	B cells	AIM2
0	1.82635549	0.276	0.049	0	B cells	ZNF395
0	1.82294036	0.625	0.157	0	B cells	SP140
0	1.80902159	0.995	0.408	0	B cells	RPL12
0	1.80566229	0.431	0.104	0	B cells	DMXL1
0	1.80036335	0.306	0.056	0	B cells	SYVN1
0	1.77069403	0.789	0.221	0	B cells	SLC38A1
0	1.76672454	0.705	0.227	0	B cells	EBLN3P
0	1.75963763	0.859	0.292	0	B cells	UCP2
0	1.75534508	0.875	0.291	0	B cells	HSP90AB1
0	1.745186	0.27	0.066	0	B cells	CCDC6

0	1.7296762	0.735	0.207	0	B cells	ST6GAL1
0	1.72654949	0.994	0.417	0	B cells	RPS18
0	1.72305132	0.917	0.296	0	B cells	RPSA
0	1.72293109	0.998	0.47	0	B cells	RPL13A
0	1.72093458	0.993	0.562	0	B cells	MT-ATP8
0	1.71517755	0.987	0.37	0	B cells	RPL23A
0	1.70499554	0.971	0.353	0	B cells	RPS19
0	1.69669243	0.289	0.072	0	B cells	CHCHD10
0	1.68502541	0.998	0.45	0	B cells	RPS20
0	1.68459074	0.89	0.298	0	B cells	RPL35
0	1.68066549	0.257	0.038	0	B cells	ODC1
0	1.67817771	0.998	0.498	0	B cells	RPS27
0	1.6672346	0.983	0.346	0	B cells	RPL10A
0	1.65497997	0.434	0.078	0	B cells	FCGR2B
0	1.64904637	0.995	0.425	0	B cells	RPLP2
0	1.64818516	0.99	0.655	0	B cells	MT-ND4L
0	1.64506127	0.994	0.381	0	B cells	RPL13
0	1.64375721	0.285	0.065	0	B cells	WDR11
0	1.64132556	0.984	0.383	0	B cells	RPS21
0	1.63727077	0.99	0.4	0	B cells	RPS17
0	1.63668715	0.312	0.074	0	B cells	SLC39A10
0	1.63389588	0.392	0.119	0	B cells	YBX3
0	1.63164198	0.925	0.333	0	B cells	EEF1B2
0	1.62981974	0.423	0.081	0	B cells	SIPA1L3
0	1.62913157	0.302	0.065	0	B cells	MAP4K1
0	1.62851884	0.301	0.073	0	B cells	DENND11
0	1.62829584	0.867	0.282	0	B cells	RPLP0
0	1.62827883	1	0.903	0	B cells	MT-CO1
0	1.62474149	0.841	0.286	0	B cells	RPL18
0	1.62272616	0.338	0.087	0	B cells	DCK
0	1.61778682	0.275	0.058	0	B cells	FAM43A
0	1.61680438	0.874	0.31	0	B cells	EEF2
0	1.6155585	0.437	0.113	0	B cells	GSAP
0	1.61379866	0.39	0.1	0	B cells	RNASEH2B
0	1.61358158	0.34	0.1	0	B cells	TAPT1
0	1.61065368	0.411	0.121	0	B cells	SNX5
0	1.607367	0.997	0.44	0	B cells	RPL37A
0	1.59968354	0.976	0.375	0	B cells	RPL15
0	1.59428876	0.479	0.201	0	B cells	IL4R
0	1.58752736	0.98	0.399	0	B cells	RPL29
0	1.58440148	0.265	0.057	0	B cells	RHBDD1
0	1.58133265	0.64	0.201	0	B cells	NOP53
0	1.57511262	0.993	0.459	0	B cells	RPL11
0	1.57260262	0.979	0.378	0	B cells	RPL36
0	1.57249826	0.909	0.334	0	B cells	RPL7A
0	1.56987182	0.29	0.065	0	B cells	CCDC32
0	1.56871352	0.983	0.399	0	B cells	RPS2
0	1.56257152	0.379	0.083	0	B cells	ARHGAP24
0	1.56243799	0.542	0.167	0	B cells	SYPL1
0	1.55640905	0.933	0.347	0	B cells	RACK1

0	1.55267385	0.412	0.115	0	B cells	AKAP11
0	1.55082519	0.992	0.377	0	B cells	RPL3
0	1.54912422	0.995	0.45	0	B cells	RPL26
0	1.5481521	1	0.909	0	B cells	MT-ATP6
0	1.54505407	0.302	0.074	0	B cells	PRKD3
0	1.54261715	0.501	0.159	0	B cells	PEBP1
0	1.54057537	0.292	0.057	0	B cells	PHACTR1
0	1.53896038	0.301	0.08	0	B cells	ARHGAP5
0	1.53719617	0.433	0.136	0	B cells	ELK4
0	1.53557295	0.984	0.401	0	B cells	RPL19
0	1.53539584	0.79	0.31	0	B cells	PLEKHA2
0	1.53219983	0.412	0.135	0	B cells	APEX1
0	1.53095022	0.998	0.496	0	B cells	RPL32
0	1.52927697	0.412	0.116	0	B cells	SP140L
0	1.5266468	0.997	0.573	0	B cells	RPL39
0	1.52370857	0.872	0.307	0	B cells	EEF1G
0	1.52037419	0.987	0.357	0	B cells	RPL22
0	1.50902625	0.981	0.358	0	B cells	RPS4X
0	1.50507154	0.254	0.06	0	B cells	AC243960.1
0	1.50359364	0.424	0.105	0	B cells	ZNF107
0	1.50219967	1	0.933	0	B cells	MT-ND3
0	1.49696351	0.967	0.404	0	B cells	RPS7
0	1.49489266	0.366	0.097	0	B cells	CTSH
0	1.49437049	0.991	0.377	0	B cells	RPL5
0	1.49330047	0.996	0.567	0	B cells	RPL21
0	1.49100819	0.999	0.539	0	B cells	RPS6
0	1.48979823	0.819	0.283	0	B cells	TLE5
0	1.48500927	0.889	0.336	0	B cells	RPS15
0	1.47861005	0.906	0.343	0	B cells	PPIA
0	1.4757768	0.372	0.118	0	B cells	HSPH1
0	1.47399914	0.998	0.504	0	B cells	RPL9
0	1.47211701	0.984	0.402	0	B cells	RPL10
0	1.46808319	0.997	0.759	0	B cells	MT-ND5
0	1.46802547	0.992	0.454	0	B cells	RPS16
0	1.46430575	0.98	0.383	0	B cells	NAP1L1
0	1.46176119	0.997	0.468	0	B cells	RPS13
0	1.46093295	0.995	0.506	0	B cells	RPL23
0	1.45770554	1	0.948	0	B cells	MT-CO2
0	1.45521471	0.32	0.099	0	B cells	GNL3
0	1.45356577	0.608	0.217	0	B cells	TCEA1
0	1.45337669	0.983	0.412	0	B cells	RPL27
0	1.4528383	0.316	0.094	0	B cells	CBX5
0	1.45217471	0.417	0.139	0	B cells	SMDT1
0	1.44977988	0.994	0.458	0	B cells	RPS28
0	1.44963856	0.31	0.086	0	B cells	CEPT1
0	1.44714983	0.496	0.159	0	B cells	ANXA6
0	1.44410175	0.277	0.079	0	B cells	USP12
0	1.44330779	0.999	0.586	0	B cells	RPS29
0	1.4418894	1	0.952	0	B cells	MT-CO3
0	1.43905464	0.297	0.065	0	B cells	PRKCE

0	1.43291923	0.987	0.445	0	B cells	RPLP1
0	1.43252949	0.997	0.53	0	B cells	RPS27A
0	1.43017414	0.999	0.623	0	B cells	RPL37
0	1.42988532	0.304	0.085	0	B cells	GABPB1-AS1
0	1.42636725	0.278	0.071	0	B cells	AMFR
0	1.42554498	0.992	0.388	0	B cells	RPS12
0	1.42408305	1	0.963	0	B cells	MT-ND4
0	1.42398715	0.997	0.635	0	B cells	RPL41
0	1.42344285	0.996	0.517	0	B cells	RPL17
0	1.42318529	0.316	0.093	0	B cells	CFAP97
0	1.42294432	0.683	0.256	0	B cells	VPS13C
0	1.41090913	0.356	0.107	0	B cells	SYNGR2
0	1.40934519	0.363	0.109	0	B cells	MT-ND6
0	1.40814615	0.456	0.147	0	B cells	MALT1
0	1.40436381	0.996	0.535	0	B cells	RPL27A
0	1.40379388	0.263	0.064	0	B cells	USP28
0	1.40337386	0.999	0.662	0	B cells	RPL34
0	1.40296102	0.254	0.077	0	B cells	ZNF22
0	1.40269933	0.992	0.459	0	B cells	RPS3A
0	1.3974245	0.25	0.077	0	B cells	ZNF480
0	1.39685174	0.336	0.078	0	B cells	TMED8
0	1.39496983	0.381	0.127	0	B cells	STARD7
0	1.39173421	0.98	0.376	0	B cells	RPL36A
0	1.3907352	0.473	0.14	0	B cells	SPTBN1
0	1.38805024	0.452	0.162	0	B cells	NME2
0	1.38773385	0.295	0.069	0	B cells	TIFA
0	1.38519213	0.995	0.484	0	B cells	RPL31
0	1.3808086	0.997	0.542	0	B cells	PTMA
0	1.37405318	0.287	0.053	0	B cells	PPP1R16B
0	1.37133702	0.906	0.319	0	B cells	NPM1
0	1.36788194	0.985	0.466	0	B cells	RPS25
0	1.36618704	0.558	0.194	0	B cells	SNHG32
0	1.36536179	0.98	0.409	0	B cells	RPL35A
0	1.36475031	0.484	0.166	0	B cells	ERP29
0	1.3617968	0.497	0.13	0	B cells	RHOH
0	1.36178319	0.35	0.121	0	B cells	CCT2
0	1.36025504	0.314	0.09	0	B cells	LILRB1
0	1.35889131	0.25	0.075	0	B cells	BMI1
0	1.358698	0.994	0.453	0	B cells	RPL6
0	1.3530661	0.304	0.095	0	B cells	SRSF8
0	1.34897155	0.466	0.148	0	B cells	CHD9
0	1.3489038	0.326	0.113	0	B cells	RAD50
0	1.34869038	0.404	0.142	0	B cells	TMPO
0	1.347444	0.582	0.197	0	B cells	GAS5
0	1.34519311	0.969	0.345	0	B cells	RPL4
0	1.34082775	0.631	0.226	0	B cells	HMGN1
0	1.33886648	0.99	0.488	0	B cells	RPL38
0	1.33756956	0.386	0.1	0	B cells	GPR183
0	1.33582022	0.513	0.126	0	B cells	BCL2
0	1.32951689	0.342	0.111	0	B cells	CNDP2

0	1.32726517	0.993	0.481	0	B cells	RPS15A
0	1.32705926	0.348	0.109	0	B cells	TRIB2
0	1.32409956	0.643	0.246	0	B cells	SRSF7
0	1.32059486	0.272	0.089	0	B cells	ARL14EP
0	1.31734939	0.436	0.115	0	B cells	GRK3
0	1.31656832	0.363	0.128	0	B cells	TCERG1
0	1.31576604	0.999	0.611	0	B cells	EEF1A1
0	1.31511372	0.325	0.103	0	B cells	SNHG8
0	1.31287782	0.27	0.078	0	B cells	PWP1
0	1.31275594	0.273	0.082	0	B cells	SNHG1
0	1.31229799	0.998	0.643	0	B cells	RPL30
0	1.30969516	0.339	0.099	0	B cells	SREBF2
0	1.3091187	0.458	0.134	0	B cells	S1PR1
0	1.30865492	0.271	0.091	0	B cells	SELENOH
0	1.30862368	0.864	0.331	0	B cells	RPL24
0	1.30787722	0.655	0.235	0	B cells	SLC25A6
0	1.30414429	0.955	0.374	0	B cells	RPL7
0	1.30386721	0.372	0.135	0	B cells	ZNF706
0	1.29995818	0.626	0.23	0	B cells	NUCKS1
0	1.29898383	1	0.924	0	B cells	MT-CYB
0	1.29540036	0.302	0.098	0	B cells	SLC25A36
0	1.29389137	0.361	0.124	0	B cells	LARS
0	1.29364846	0.254	0.06	0	B cells	GUCD1
0	1.29225647	0.416	0.145	0	B cells	TRIM44
0	1.2827026	0.715	0.268	0	B cells	HINT1
0	1.28003436	0.794	0.308	0	B cells	NCL
0	1.27956351	0.556	0.206	0	B cells	TTC3
0	1.27814136	0.792	0.332	0	B cells	HNRNPDL
0	1.27446059	0.407	0.144	0	B cells	NOP58
0	1.27340565	0.619	0.235	0	B cells	RAN
0	1.27123308	0.389	0.101	0	B cells	PPP3CC
0	1.27053134	0.939	0.394	0	B cells	RPS3
0	1.2704412	1	0.931	0	B cells	MT-ND2
0	1.26998215	0.318	0.101	0	B cells	CDC16
0	1.26969353	0.408	0.121	0	B cells	TRAK1
0	1.26876962	0.321	0.111	0	B cells	LRPPRC
0	1.26727189	0.984	0.429	0	B cells	RPL14
0	1.26518144	0.364	0.123	0	B cells	DARS
0	1.25659598	0.289	0.101	0	B cells	RCC2
0	1.25482675	0.261	0.076	0	B cells	USP11
0	1.25411821	0.551	0.199	0	B cells	PPDPF
0	1.25195197	0.397	0.127	0	B cells	OFD1
0	1.2509484	0.689	0.258	0	B cells	KMT2A
0	1.25021449	0.335	0.114	0	B cells	OOEP
0	1.24900121	0.804	0.303	0	B cells	HNRNPA1
0	1.24629797	0.377	0.132	0	B cells	NASP
0	1.24410004	0.32	0.1	0	B cells	CD2AP
0	1.2430355	0.297	0.107	0	B cells	SSRP1
0	1.24120688	0.455	0.163	0	B cells	ZBED5
0	1.24065185	0.457	0.169	0	B cells	BANF1

0	1.23775482	0.312	0.106	0	B cells	WDR43
0	1.23738907	0.355	0.102	0	B cells	SNX29
0	1.23708254	0.604	0.233	0	B cells	NSA2
0	1.23544715	0.267	0.089	0	B cells	PHF10
0	1.23530463	0.928	0.433	0	B cells	RPL28
0	1.23355244	0.253	0.082	0	B cells	PEA15
0	1.23015735	0.3	0.106	0	B cells	ABCE1
0	1.22922852	0.274	0.082	0	B cells	SINHCAF
0	1.22857812	0.416	0.154	0	B cells	BDP1
0	1.2269587	0.337	0.122	0	B cells	FBL
0	1.22414969	0.311	0.096	0	B cells	RNGTT
0	1.21904481	0.263	0.085	0	B cells	KRR1
0	1.21852557	0.325	0.116	0	B cells	TTC37
0	1.21737483	0.359	0.112	0	B cells	ACBD3
0	1.21641357	0.733	0.371	0	B cells	RPS26
0	1.21599196	0.378	0.144	0	B cells	EPRS
0	1.21388875	0.532	0.173	0	B cells	HHEX
0	1.21248158	0.995	0.529	0	B cells	RPS24
0	1.21121338	0.323	0.112	0	B cells	RBBP7
0	1.2098383	0.261	0.087	0	B cells	NR1D2
0	1.20911479	0.305	0.113	0	B cells	HSPE1
0	1.20839929	0.484	0.168	0	B cells	ADK
0	1.20707571	0.684	0.256	0	B cells	EIF3E
0	1.20644298	0.628	0.213	0	B cells	ZCCHC7
0	1.20582199	0.43	0.117	0	B cells	WDFY4
0	1.20539708	0.356	0.107	0	B cells	FAM117B
0	1.20416952	0.531	0.207	0	B cells	RBMX
0	1.19975163	0.3	0.111	0	B cells	NOLC1
0	1.1992422	0.78	0.375	0	B cells	SEC62
0	1.19734488	0.606	0.238	0	B cells	MBD4
0	1.1961128	0.678	0.248	0	B cells	SNHG29
0	1.19180095	0.697	0.272	0	B cells	COX7C
0	1.19172331	0.968	0.424	0	B cells	RPS14
0	1.18936055	0.286	0.108	0	B cells	AIMP1
0	1.1889105	0.844	0.357	0	B cells	TOMM7
0	1.18843817	0.656	0.245	0	B cells	CLEC2D
0	1.18783639	0.875	0.453	0	B cells	LTB
0	1.1821023	0.423	0.121	0	B cells	CCR7
0	1.1816502	0.278	0.077	0	B cells	SMAD3
0	1.18003256	0.47	0.184	0	B cells	SRP72
0	1.17635717	0.269	0.079	0	B cells	KYNU
0	1.17098247	0.259	0.09	0	B cells	LAIR1
0	1.16989041	0.344	0.126	0	B cells	IMP3
0	1.1696639	0.551	0.223	0	B cells	EIF3F
0	1.16678937	0.314	0.113	0	B cells	ZNF146
0	1.16388007	0.523	0.204	0	B cells	SNRPD2
0	1.16243542	0.386	0.152	0	B cells	RPL7L1
0	1.1610187	0.4	0.157	0	B cells	TOMM6
0	1.15864915	0.553	0.212	0	B cells	RSL1D1
0	1.15601291	0.351	0.128	0	B cells	NOP56

0	1.15490085	0.468	0.189	0	B cells	SSB
0	1.15051947	0.521	0.212	0	B cells	OIP5-AS1
0	1.14931472	0.297	0.107	0	B cells	NOL8
0	1.1489921	0.734	0.303	0	B cells	NCOA3
0	1.14667639	0.273	0.096	0	B cells	SNHG3
0	1.14636368	0.321	0.123	0	B cells	CEBPZ
0	1.14590294	0.573	0.217	0	B cells	SH3BP5
0	1.14516157	0.896	0.388	0	B cells	YBX1
0	1.14514116	0.251	0.082	0	B cells	TP53
0	1.13798717	0.331	0.124	0	B cells	UPF3A
0	1.13788661	0.678	0.309	0	B cells	SCARNA9
0	1.13676484	0.365	0.146	0	B cells	RAD23A
0	1.13615004	0.331	0.108	0	B cells	ANKRD10
0	1.13611408	0.701	0.284	0	B cells	SEPTIN6
0	1.13597797	0.564	0.221	0	B cells	FNBP4
0	1.13568069	0.416	0.164	0	B cells	DDX18
0	1.13540872	0.364	0.132	0	B cells	DDX27
0	1.13186426	0.303	0.103	0	B cells	SNHG14
0	1.1293097	0.802	0.373	0	B cells	EIF4A2
0	1.12832359	0.315	0.123	0	B cells	SDAD1
0	1.12698098	0.531	0.215	0	B cells	RPS4Y1
0	1.12362464	0.353	0.137	0	B cells	METAP2
0	1.12063453	0.332	0.109	0	B cells	NFX1
0	1.12059356	0.412	0.126	0	B cells	MGAT5
0	1.11437359	0.551	0.222	0	B cells	DDX24
0	1.11268313	0.272	0.087	0	B cells	USPL1
0	1.11010963	0.553	0.217	0	B cells	CNTRL
0	1.10930324	0.257	0.077	0	B cells	GOLGA8B
0	1.10904903	0.459	0.183	0	B cells	ATXN7L3B
0	1.10788405	0.317	0.093	0	B cells	PHTF2
0	1.1071311	0.57	0.231	0	B cells	REL
0	1.10501376	0.31	0.121	0	B cells	CLNS1A
0	1.09960654	0.623	0.264	0	B cells	UHMK1
0	1.09954964	0.375	0.153	0	B cells	PHB2
0	1.0975384	0.472	0.191	0	B cells	SNHG6
0	1.09554079	0.722	0.322	0	B cells	MYCBP2
0	1.09546796	0.441	0.179	0	B cells	UQCRH
0	1.09485049	0.302	0.098	0	B cells	USP9Y
0	1.08854092	0.306	0.111	0	B cells	RPS10
0	1.08845617	0.47	0.189	0	B cells	SMARCE1
0	1.08714295	0.39	0.155	0	B cells	SRSF6
0	1.08679487	0.444	0.179	0	B cells	ILF3
0	1.08431777	0.444	0.178	0	B cells	EIF1AX
0	1.08368828	0.301	0.097	0	B cells	UHRF2
0	1.08314702	0.405	0.164	0	B cells	CCT4
0	1.08275554	0.285	0.091	0	B cells	LY9
0	1.08137453	0.283	0.101	0	B cells	HSD17B4
0	1.08107474	0.867	0.393	0	B cells	EIF4B
0	1.0807949	0.47	0.186	0	B cells	FUBP1
0	1.08036256	0.422	0.172	0	B cells	HSPA4

0	1.07709982	0.308	0.113	0	B cells	SNHG25
0	1.07422003	0.366	0.121	0	B cells	CALHM6
0	1.07317295	0.25	0.085	0	B cells	ITFG2
0	1.07283107	0.578	0.242	0	B cells	KTN1
0	1.06926546	0.551	0.231	0	B cells	ZRANB2
0	1.06264058	0.655	0.289	0	B cells	SERBP1
0	1.06240423	0.48	0.196	0	B cells	GTF3A
0	1.06230056	0.41	0.164	0	B cells	UBE3A
0	1.05932896	0.303	0.107	0	B cells	DCLRE1C
0	1.05765071	0.291	0.107	0	B cells	COX20
0	1.05758471	0.512	0.193	0	B cells	UVRAG
0	1.05733569	0.452	0.17	0	B cells	POLD4
0	1.05565394	0.274	0.077	0	B cells	TTC9
0	1.0534348	0.578	0.247	0	B cells	ST13
0	1.05095098	0.503	0.211	0	B cells	G3BP1
0	1.04977327	0.406	0.165	0	B cells	BLOC1S6
0	1.0486619	0.429	0.17	0	B cells	ERAP2
0	1.04266569	1	0.942	0	B cells	MT-ND1
0	1.03939534	0.338	0.125	0	B cells	NBEAL1
0	1.03847697	0.488	0.179	0	B cells	PRKACB
0	1.03776884	0.414	0.176	0	B cells	UFM1
0	1.03593624	0.576	0.239	0	B cells	ANAPC16
0	1.03320034	0.735	0.333	0	B cells	COMMD6
0	1.03145981	0.44	0.186	0	B cells	TASOR
0	1.02798185	0.31	0.094	0	B cells	UGCG
0	1.02670201	0.287	0.102	0	B cells	CEP120
0	1.02640521	0.756	0.356	0	B cells	MATR3
0	1.02162201	0.356	0.141	0	B cells	EDEM1
0	1.01944632	0.35	0.144	0	B cells	RFC1
0	1.01877577	0.3	0.111	0	B cells	MGA
0	1.01769512	0.522	0.21	0	B cells	GLS
0	1.01567764	0.368	0.153	0	B cells	UBE2N
0	1.01379201	0.876	0.471	0	B cells	FAU
0	1.0122111	0.284	0.1	0	B cells	MPRIP
0	1.00920002	0.314	0.12	0	B cells	CIAO2A
0	1.00828284	0.482	0.205	0	B cells	TRA2B
0	1.00765511	0.404	0.168	0	B cells	TIA1
0	1.00629282	0.444	0.171	0	B cells	PIKFYVE
0	1.00511813	0.356	0.135	0	B cells	ZNF644
0	1.0029089	0.436	0.186	0	B cells	SOD1
0	1.00064344	0.434	0.181	0	B cells	CMPK1
0	0.99858349	0.272	0.095	0	B cells	RFX7
0	0.98990916	0.257	0.085	0	B cells	RHBDF2
0	0.98939858	0.924	0.451	0	B cells	NACA
0	0.98815215	0.41	0.169	0	B cells	ETNK1
0	0.97816164	0.741	0.322	0	B cells	BTF3
0	0.97400148	0.354	0.137	0	B cells	ZNF91
0	0.97174282	0.396	0.173	0	B cells	LARP1
0	0.97057811	0.628	0.281	0	B cells	SNX3
0	0.96894642	0.63	0.278	0	B cells	HNRNPH1

0	0.96597754	0.458	0.176	0	B cells	RABEP1
0	0.9635721	0.884	0.394	0	B cells	TMSB10
0	0.95980346	0.659	0.314	0	B cells	PNRC2
0	0.95898909	0.507	0.167	0	B cells	ABLIM1
0	0.95798746	0.514	0.214	0	B cells	RB1
0	0.95564147	0.443	0.186	0	B cells	SECISBP2L
0	0.95545765	0.409	0.171	0	B cells	ZBTB1
0	0.95131524	0.52	0.226	0	B cells	SYNCRIP
0	0.94615989	0.454	0.197	0	B cells	BAZ1B
0	0.94367425	0.514	0.22	0	B cells	PTPN1
0	0.93905673	0.335	0.129	0	B cells	CSNK1G3
0	0.93868423	0.38	0.14	0	B cells	USP24
0	0.93154332	0.32	0.11	0	B cells	CCDC18-AS1
0	0.92964875	0.538	0.186	0	B cells	RUBCNL
0	0.92636072	0.698	0.335	0	B cells	HNRNPA3
0	0.92598806	0.363	0.147	0	B cells	TTC14
0	0.92550546	0.712	0.317	0	B cells	EIF3H
0	0.92320608	0.879	0.479	0	B cells	SET
0	0.91614519	0.284	0.098	0	B cells	DOP1B
0	0.91106224	0.765	0.348	0	B cells	ATP5MC2
0	0.90986553	0.564	0.259	0	B cells	HNRNPR
0	0.90792772	0.417	0.178	0	B cells	RNPS1
0	0.90579177	0.687	0.312	0	B cells	MTDH
0	0.90422689	0.412	0.178	0	B cells	YWHAQ
0	0.90201473	0.507	0.219	0	B cells	RBM26
0	0.90100102	0.336	0.129	0	B cells	KDM4C
0	0.89955276	0.347	0.125	0	B cells	HIST1H4C
0	0.89933052	0.836	0.28	0	B cells	ETS1
0	0.8902236	0.61	0.261	0	B cells	SNHG5
0	0.88881349	0.462	0.209	0	B cells	EIF3D
0	0.88291332	0.407	0.17	0	B cells	STIM2
0	0.8826234	0.381	0.162	0	B cells	URI1
0	0.87601429	0.459	0.205	0	B cells	THOC2
0	0.87527151	0.477	0.203	0	B cells	TMEM131
0	0.87514761	0.456	0.203	0	B cells	SRSF10
0	0.87315185	0.431	0.196	0	B cells	SEC31A
0	0.86951215	0.589	0.264	0	B cells	CALR
0	0.863566	0.631	0.312	0	B cells	PNN
0	0.85514443	0.336	0.134	0	B cells	PHKB
0	0.85382743	0.529	0.237	0	B cells	COX6C
0	0.85195789	0.488	0.2	0	B cells	TUT4
0	0.84975459	0.448	0.185	0	B cells	PHC3
0	0.84746658	0.673	0.3	0	B cells	HSP90B1
0	0.84429491	0.841	0.464	0	B cells	EEF1D
0	0.84380273	0.585	0.277	0	B cells	C11orf58
0	0.84296346	0.553	0.26	0	B cells	TOP2B
0	0.84137077	0.579	0.286	0	B cells	SELENOF
0	0.83744548	0.409	0.152	0	B cells	DENND4A
0	0.83508961	0.488	0.229	0	B cells	XPO1
0	0.8347667	0.743	0.344	0	B cells	TNFAIP8

0	0.82939066	0.604	0.28	0	B cells	STX7
0	0.82910809	0.514	0.242	0	B cells	U2SURP
0	0.82838239	0.317	0.122	0	B cells	ASXL1
0	0.82637565	0.681	0.339	0	B cells	LUC7L3
0	0.826251	0.401	0.151	0	B cells	DGKD
0	0.82050123	0.457	0.196	0	B cells	PDS5A
0	0.81924899	0.538	0.261	0	B cells	SRSF2
0	0.81753801	0.486	0.216	0	B cells	PRRC2B
0	0.81460602	0.491	0.22	0	B cells	MIS18BP1
0	0.80442479	0.676	0.273	0	B cells	AHNAK
0	0.80146876	0.682	0.282	0	B cells	ITGA4
0	0.80086973	0.33	0.125	0	B cells	LRBA
0	0.79323627	0.637	0.278	0	B cells	GAPT
0	0.7835946	0.771	0.394	0	B cells	PFDN5
0	0.7819361	0.667	0.34	0	B cells	SFPQ
0	0.76707934	0.945	0.591	0	B cells	HNRNPA2B1
0	0.76571585	0.513	0.237	0	B cells	BBX
0	0.7644758	0.512	0.244	0	B cells	DDX21
0	0.76202332	0.674	0.35	0	B cells	TRIM38
0	0.76198381	0.581	0.289	0	B cells	UBB
0	0.76016565	0.756	0.367	0	B cells	HSPA8
0	0.75314302	0.792	0.434	0	B cells	SRSF11
0	0.74980439	0.34	0.135	0	B cells	TCF12
0	0.74940705	0.766	0.37	0	B cells	ANP32B
0	0.74608486	0.911	0.579	0	B cells	SRSF5
0	0.74314395	0.69	0.373	0	B cells	ARF6
0	0.74107078	0.663	0.349	0	B cells	ARGLU1
0	0.74082023	0.467	0.2	0	B cells	PXK
0	0.74001357	0.56	0.268	0	B cells	PCM1
0	0.73623303	0.383	0.157	0	B cells	SESN3
0	0.73185301	0.944	0.603	0	B cells	UBA52
0	0.72404083	0.657	0.322	0	B cells	TRIM22
0	0.71988577	0.435	0.166	0	B cells	CDK14
0	0.71668763	0.58	0.266	0	B cells	SEMA4B
0	0.71365002	0.507	0.233	0	B cells	TLK1
0	0.71350944	0.76	0.417	0	B cells	HNRNPD
0	0.70160352	0.681	0.355	0	B cells	EIF2S3
0	0.70032544	0.622	0.316	0	B cells	RBM3
0	0.70005746	0.546	0.245	0	B cells	ARID1B
0	0.68677568	0.832	0.488	0	B cells	RPS9
0	0.68335254	0.993	0.745	0	B cells	PABPC1
0	0.66832691	0.73	0.199	0	B cells	CYBB
0	0.6662593	0.679	0.369	0	B cells	CD47
0	0.64579587	0.579	0.271	0	B cells	PDE7A
0	0.64569277	0.468	0.203	0	B cells	EVL
0	0.62406307	0.69	0.344	0	B cells	CD48
0	0.59407263	1	0.89	0	B cells	TPT1
0	0.58718685	0.711	0.389	0	B cells	MAP3K1
0	0.58572336	0.519	0.222	0	B cells	ADAM19
0	0.57320873	0.812	0.49	0	B cells	RCSD1

0	0.5580794	0.702	0.369	0	B cells	ATM
0	0.55786903	0.8	0.454	0	B cells	FOXP1
7.584E-308	0.77685876	0.518	0.254	2.544E-303	B cells	PRPF4B
2.409E-307	1.06470338	0.272	0.098	8.082E-303	B cells	ARPC5L
1.257E-306	0.96061665	0.341	0.139	4.218E-302	B cells	SFT2D2
3.683E-306	0.63460602	0.594	0.304	1.235E-301	B cells	TRAF3IP3
4.64E-305	0.91585968	0.358	0.149	1.556E-300	B cells	API5
5.459E-305	0.86214638	0.422	0.191	1.831E-300	B cells	TARDBP
4.264E-304	0.57279563	0.493	0.235	1.43E-299	B cells	RBM6
3.618E-303	1.1399087	0.266	0.096	1.213E-298	B cells	NOL11
4.253E-303	0.97161023	0.393	0.173	1.427E-298	B cells	EIF5B
2.364E-302	0.72116562	0.509	0.246	7.93E-298	B cells	PPP1CC
6.192E-302	0.74062404	0.53	0.262	2.077E-297	B cells	ATXN2L
1.757E-300	1.07313053	0.25	0.088	5.892E-296	B cells	AP1B1
3.06E-300	0.94789335	0.302	0.115	1.026E-295	B cells	DMTF1
7.328E-300	0.74734906	0.537	0.266	2.458E-295	B cells	COX4I1
7.472E-300	0.75437852	0.392	0.169	2.506E-295	B cells	MAPRE2
8.57E-300	0.96782414	0.251	0.086	2.875E-295	B cells	JADE2
7.052E-299	0.93789299	0.394	0.176	2.365E-294	B cells	SSBP1
1.032E-298	0.81285599	0.388	0.169	3.461E-294	B cells	TNRC6A
4.671E-298	1.11121557	0.254	0.09	1.567E-293	B cells	PTCD3
6.135E-298	0.56092039	0.517	0.255	2.058E-293	B cells	TBC1D5
2.432E-297	0.67763631	0.498	0.244	8.158E-293	B cells	MEF2A
7.517E-297	0.97502376	0.315	0.125	2.521E-292	B cells	ZNF451
9.268E-297	1.2284602	0.256	0.092	3.109E-292	B cells	TRAK2
1.293E-296	0.8964013	0.294	0.112	4.337E-292	B cells	BTAF1
2.623E-296	0.96210827	0.333	0.136	8.8E-292	B cells	LSM8
5.309E-296	0.62848193	0.476	0.226	1.781E-291	B cells	FOXO1
3.24E-295	0.87919209	0.528	0.274	1.087E-290	B cells	VOPP1
3.881E-295	0.69553649	0.558	0.285	1.302E-290	B cells	LNPEP
6.32E-295	0.58019232	0.59	0.307	2.12E-290	B cells	MACF1
1.76E-293	1.03850312	0.268	0.098	5.905E-289	B cells	CREBZF
3.752E-293	0.5503744	0.834	0.486	1.259E-288	B cells	HSP90AA1
4.262E-293	0.93609337	0.259	0.093	1.429E-288	B cells	SCAF4
5.217E-293	1.03623705	0.275	0.102	1.75E-288	B cells	ZCCHC10
5.41E-293	0.53311154	0.633	0.329	1.815E-288	B cells	CYFIP2
3.483E-292	0.70520255	0.574	0.294	1.168E-287	B cells	NONO
4.165E-292	0.91897144	0.273	0.099	1.397E-287	B cells	ITGB7
2.24E-291	0.55397548	0.389	0.17	7.514E-287	B cells	DOCK10
6.826E-291	0.38942472	0.559	0.277	2.29E-286	B cells	FCHSD2
9.921E-291	0.72854081	0.51	0.252	3.328E-286	B cells	TUBB
3.583E-290	1.06057446	0.322	0.132	1.202E-285	B cells	MDH1
9.74E-290	1.01677424	0.353	0.151	3.267E-285	B cells	IPO7
1.051E-288	0.90421827	0.374	0.163	3.525E-284	B cells	BUB3
1.082E-288	0.86081038	0.428	0.198	3.628E-284	B cells	HSPD1
3.386E-287	0.9939348	0.372	0.164	1.136E-282	B cells	EIF3I
4.561E-287	1.12460054	0.284	0.109	1.53E-282	B cells	MPHOSPH10
1.888E-286	0.58357019	0.697	0.4	6.333E-282	B cells	ADD1
4.449E-286	0.81703503	0.388	0.172	1.492E-281	B cells	CDC40
2.847E-285	0.98148116	0.252	0.09	9.55E-281	B cells	ARHGEF7

3.027E-285	0.89767613	0.424	0.199	1.015E-280	B cells	PA2G4
1.797E-283	0.7226481	0.425	0.195	6.028E-279	B cells	HDAC1
3.214E-283	0.9979049	0.317	0.129	1.078E-278	B cells	ZNF770
1.285E-282	0.85991959	0.311	0.122	4.31E-278	B cells	ZNF506
3.741E-282	0.59098722	0.366	0.155	1.255E-277	B cells	KLF12
5.929E-282	0.43183776	0.415	0.181	1.989E-277	B cells	RUNX3
8.467E-282	0.9255685	0.298	0.117	2.84E-277	B cells	ZMYND11
1.43E-281	1.03617922	0.275	0.105	4.795E-277	B cells	HMGA1
3.775E-281	0.83036805	0.413	0.192	1.266E-276	B cells	RBM17
8.045E-281	0.7400979	0.413	0.187	2.699E-276	B cells	SPTAN1
2.308E-280	0.67800419	0.325	0.13	7.743E-276	B cells	CYSLTR1
2.999E-280	0.50602043	0.9	0.637	1.006E-275	B cells	SP100
2.595E-279	0.94947698	0.375	0.168	8.705E-275	B cells	PABPC4
3.226E-279	0.83868534	0.342	0.145	1.082E-274	B cells	MDFIC
1.727E-278	0.92830149	0.337	0.143	5.794E-274	B cells	NOL7
1.666E-277	0.86094216	0.36	0.156	5.587E-273	B cells	DUT
3.355E-277	0.79034775	0.411	0.188	1.125E-272	B cells	SUPT16H
1.652E-276	0.66652384	0.541	0.274	5.543E-272	B cells	TRAM1
2.047E-275	1.00558703	0.276	0.106	6.866E-271	B cells	UBA2
8.002E-275	0.70354029	0.514	0.255	2.684E-270	B cells	FXYD5
1.261E-274	0.3592221	0.586	0.297	4.23E-270	B cells	ATP2A3
6.302E-274	0.46357186	0.599	0.308	2.114E-269	B cells	ORAI2
9.213E-274	0.88262263	0.338	0.143	3.09E-269	B cells	RAB29
2.487E-273	0.70508902	0.524	0.267	8.341E-269	B cells	EFCAB14
7.325E-273	0.91555253	0.34	0.146	2.457E-268	B cells	UBE2G2
8.046E-273	0.59219437	0.442	0.209	2.699E-268	B cells	P2RY8
9.739E-273	0.76720364	0.271	0.102	3.267E-268	B cells	HEATR5B
4.671E-272	0.75965951	0.411	0.188	1.567E-267	B cells	UBTF
1.001E-271	0.72978539	0.519	0.263	3.358E-267	B cells	SRSF3
1.374E-271	0.60782019	0.592	0.318	4.607E-267	B cells	PRDM2
8.541E-271	0.91517861	0.32	0.134	2.865E-266	B cells	MEAF6
2.582E-270	0.99639082	0.266	0.1	8.662E-266	B cells	MDN1
4.259E-269	0.87702816	0.393	0.181	1.429E-264	B cells	ATP5F1A
1.131E-268	0.90994155	0.383	0.174	3.793E-264	B cells	SLC25A5
1.991E-268	0.80705775	0.378	0.169	6.678E-264	B cells	ANP32E
3.797E-268	0.54910048	0.804	0.509	1.274E-263	B cells	PNISR
5.506E-268	0.70311092	0.285	0.11	1.847E-263	B cells	HECTD4
9.47E-268	0.95245698	0.281	0.109	3.176E-263	B cells	CENPC
6.109E-267	0.72338362	0.455	0.221	2.049E-262	B cells	DHX9
7.332E-266	0.59877651	0.665	0.373	2.459E-261	B cells	SRRM1
1.053E-265	0.83389576	0.31	0.128	3.532E-261	B cells	TRPM7
2.847E-265	0.71747601	0.446	0.214	9.551E-261	B cells	SPCS2
3.744E-265	0.82134716	0.41	0.192	1.256E-260	B cells	EID1
1.028E-264	0.86437257	0.35	0.154	3.449E-260	B cells	ZNF800
6.184E-263	0.63358935	0.485	0.242	2.074E-258	B cells	UBE2I
6.52E-263	0.90334985	0.334	0.145	2.187E-258	B cells	SNU13
1.154E-262	1.0435922	0.29	0.118	3.869E-258	B cells	POLE3
4.546E-262	0.75679222	0.432	0.207	1.525E-257	B cells	KHSRP
3.214E-261	0.51645605	0.414	0.186	1.078E-256	B cells	CHD7
1.51E-260	0.9673137	0.267	0.104	5.066E-256	B cells	PHF14

4.345E-260	0.60897196	0.307	0.125	1.457E-255	B cells	TRAF3
1.074E-259	0.99885869	0.251	0.095	3.601E-255	B cells	NAA15
1.65E-259	0.8168151	0.383	0.177	5.536E-255	B cells	RBBP4
1.75E-259	1.01420373	0.27	0.106	5.869E-255	B cells	DKC1
4.467E-259	0.77150705	0.27	0.103	1.499E-254	B cells	IER5
2.756E-256	0.47680304	0.315	0.126	9.243E-252	B cells	ZHX2
5.367E-256	0.85030409	0.35	0.156	1.8E-251	B cells	GSPT1
8.607E-256	0.89497649	0.277	0.11	2.887E-251	B cells	WDR6
5.45E-255	0.88382694	0.255	0.097	1.828E-250	B cells	BRI3BP
2.881E-254	0.86226692	0.273	0.107	9.664E-250	B cells	NUMA1
3.828E-254	0.73733009	0.291	0.116	1.284E-249	B cells	NPIPB5
5.452E-254	0.64613568	0.432	0.209	1.829E-249	B cells	EIF2AK1
2.753E-253	1.03207423	0.274	0.11	9.235E-249	B cells	SNRPD3
3.1E-253	1.04619467	0.255	0.099	1.04E-248	B cells	ACP1
5.771E-253	0.7532045	0.255	0.097	1.936E-248	B cells	MAP4
7.719E-253	0.52821039	0.712	0.422	2.589E-248	B cells	FAM107B
1.284E-252	0.59493328	0.617	0.332	4.307E-248	B cells	UQCRB
6.3E-252	0.75295205	0.413	0.197	2.113E-247	B cells	VHL
8.371E-252	0.54394203	0.609	0.333	2.808E-247	B cells	CIRBP
2.654E-251	0.58487751	0.642	0.356	8.902E-247	B cells	GDI2
1.672E-250	0.65453681	0.403	0.188	5.607E-246	B cells	SERTAD2
3.704E-250	0.84550174	0.334	0.146	1.243E-245	B cells	DHX36
3.148E-249	0.73014365	0.338	0.146	1.056E-244	B cells	FDFT1
3.564E-249	0.8504848	0.288	0.117	1.196E-244	B cells	CPSF6
5.997E-249	0.89089468	0.311	0.133	2.011E-244	B cells	HSPA9
1.659E-248	0.93058122	0.268	0.107	5.565E-244	B cells	SF3B3
2.551E-247	0.42290055	0.623	0.346	8.557E-243	B cells	PARP14
1.217E-245	0.57574811	0.469	0.234	4.082E-241	B cells	MDM4
2.155E-245	0.68955088	0.437	0.213	7.229E-241	B cells	BLOC1S2
5.526E-245	0.78876807	0.264	0.103	1.853E-240	B cells	HMBOX1
8.536E-245	0.90812492	0.269	0.107	2.863E-240	B cells	TERF2
4.222E-244	0.70777977	0.331	0.143	1.416E-239	B cells	ZNF721
1.533E-243	0.69836075	0.37	0.17	5.141E-239	B cells	PPHLN1
2.602E-243	0.76082982	0.366	0.168	8.729E-239	B cells	ARAP2
7.265E-243	0.74408377	0.365	0.168	2.437E-238	B cells	CSNK2A1
2.111E-241	0.56829449	0.554	0.296	7.082E-237	B cells	HNRNPM
2.394E-241	0.85268348	0.333	0.149	8.032E-237	B cells	VPS36
3.002E-241	0.84174719	0.373	0.176	1.007E-236	B cells	TOMM20
3.084E-241	0.79358983	0.327	0.142	1.035E-236	B cells	DYRK2
9.754E-241	0.91048112	0.294	0.124	3.272E-236	B cells	RECQL
5.371E-240	0.98025606	0.287	0.122	1.802E-235	B cells	PTDSS1
2.548E-238	0.62792797	0.643	0.378	8.546E-234	B cells	ATF7IP
2.677E-238	0.82064644	0.261	0.102	8.98E-234	B cells	CAPG
1.251E-237	0.96975319	0.269	0.11	4.197E-233	B cells	CAND1
2.122E-237	0.95503004	0.271	0.111	7.117E-233	B cells	CHORDC1
2.916E-237	0.78198938	0.305	0.132	9.782E-233	B cells	SUCLG2
1.333E-236	0.58889155	0.388	0.181	4.471E-232	B cells	PSME4
1.598E-236	0.62602835	0.518	0.275	5.361E-232	B cells	HNRNPL
2.553E-236	0.73510132	0.418	0.206	8.564E-232	B cells	ZC3H15
3.355E-236	0.41556491	0.51	0.252	1.125E-231	B cells	LTA4H

9.099E-236	0.78605481	0.269	0.106	3.052E-231	B cells	SEPTIN1
9.367E-236	0.74014104	0.412	0.2	3.142E-231	B cells	OXA1L
2.975E-234	1.00572307	0.271	0.112	9.978E-230	B cells	DNAJC21
4.291E-234	0.7379541	0.31	0.133	1.439E-229	B cells	UTP6
5.401E-234	0.70371785	0.469	0.241	1.812E-229	B cells	TRMT112
1.538E-231	0.55483941	0.413	0.197	5.159E-227	B cells	FAM214A
2.028E-231	0.72978337	0.392	0.189	6.801E-227	B cells	CNOT7
4.554E-231	0.59874352	0.475	0.244	1.527E-226	B cells	TAF1D
2.242E-230	0.70152929	0.288	0.121	7.519E-226	B cells	CCDC91
3.806E-230	0.72355197	0.395	0.192	1.277E-225	B cells	DHX15
3.936E-230	0.66886216	0.409	0.2	1.32E-225	B cells	C1GALT1
6.238E-230	0.50044209	0.665	0.387	2.092E-225	B cells	PDCD4
9.252E-230	1.02942507	0.25	0.101	3.103E-225	B cells	NRIP1
1.344E-229	0.98644919	0.255	0.103	4.507E-225	B cells	KRT10
4.017E-229	0.86300282	0.312	0.138	1.347E-224	B cells	DYNC1I2
2.39E-228	0.83172935	0.301	0.131	8.016E-224	B cells	ACO2
1.805E-226	0.73063617	0.359	0.169	6.054E-222	B cells	NFATC2IP
2.137E-225	0.58089956	0.437	0.22	7.17E-221	B cells	PCGF5
2.852E-225	0.60773666	0.411	0.203	9.567E-221	B cells	CCAR1
2.06E-224	0.59682037	0.427	0.215	6.91E-220	B cells	ZFR
2.522E-224	0.71712908	0.357	0.167	8.458E-220	B cells	TFAM
9.838E-224	0.47728993	0.596	0.331	3.3E-219	B cells	WASHC4
2.043E-223	0.58199118	0.566	0.316	6.852E-219	B cells	USP7
9.538E-223	0.36318379	0.441	0.22	3.199E-218	B cells	ZBTB20
4.972E-222	0.83231783	0.251	0.099	1.668E-217	B cells	ORMDL3
5.39E-222	0.87408757	0.297	0.131	1.808E-217	B cells	DDB1
3.148E-221	0.90242242	0.251	0.102	1.056E-216	B cells	ASCC3
6.089E-221	0.77438139	0.345	0.161	2.042E-216	B cells	PDIA6
1.806E-220	0.59153313	0.522	0.28	6.057E-216	B cells	SMARCA5
1.389E-219	0.64150159	0.321	0.145	4.658E-215	B cells	JAZF1
2.377E-218	0.5594312	0.465	0.243	7.974E-214	B cells	LUC7L2
2.568E-218	0.97058064	0.264	0.111	8.612E-214	B cells	USP14
9.941E-218	0.60801354	0.345	0.161	3.335E-213	B cells	MAPKAP1
1.901E-217	0.49501064	0.511	0.273	6.376E-213	B cells	UBE2J1
3.604E-217	0.65066577	0.275	0.115	1.209E-212	B cells	ATP8A1
6.639E-217	0.45320118	0.556	0.298	2.227E-212	B cells	KIAA0040
6.943E-217	0.72345595	0.256	0.104	2.329E-212	B cells	LUC7L
7.092E-216	0.55171014	0.496	0.265	2.379E-211	B cells	NCKAP1L
1.318E-215	0.63373137	0.362	0.173	4.421E-211	B cells	HUWE1
1.348E-215	0.67967313	0.419	0.212	4.523E-211	B cells	WBP11
1.825E-215	0.89715075	0.275	0.118	6.12E-211	B cells	NDUFA5
8.378E-215	0.71042002	0.393	0.196	2.81E-210	B cells	KRTCAP2
2.008E-214	0.6745987	0.32	0.144	6.735E-210	B cells	LEMD3
8.043E-214	0.64061786	0.353	0.168	2.698E-209	B cells	HECTD1
1.658E-213	0.92625618	0.25	0.103	5.562E-209	B cells	CUTA
1.047E-212	0.62505263	0.506	0.275	3.51E-208	B cells	CGGBP1
1.917E-212	0.55752979	0.487	0.26	6.432E-208	B cells	PDCD6IP
3.244E-211	0.63931581	0.434	0.223	1.088E-206	B cells	HNRNPA0
4.473E-211	0.86638398	0.27	0.115	1.5E-206	B cells	ICE1
3.715E-210	0.68229938	0.386	0.192	1.246E-205	B cells	SEC63

4.038E-210	0.63984961	0.308	0.137	1.354E-205	B cells	PCSK7
4.386E-210	0.57610902	0.993	0.967	1.471E-205	B cells	MTRNR2L12
5.209E-210	0.41760004	0.797	0.518	1.747E-205	B cells	ADD3
5.943E-210	0.57028862	0.5	0.274	1.994E-205	B cells	AKAP9
1.075E-209	0.60087654	0.487	0.264	3.607E-205	B cells	RBBP6
1.318E-209	0.68540381	0.287	0.125	4.419E-205	B cells	AP1G2
1.846E-209	0.48393962	0.656	0.385	6.191E-205	B cells	KHDRBS1
6.357E-209	0.42336079	0.34	0.157	2.132E-204	B cells	MSI2
6.821E-209	0.95413476	0.254	0.107	2.288E-204	B cells	MAT2A
6.99E-209	0.70620735	0.355	0.171	2.345E-204	B cells	SMC5
7.997E-209	0.41824102	0.682	0.413	2.682E-204	B cells	NSD3
8.65E-209	0.68213917	0.372	0.182	2.902E-204	B cells	PCNP
6.514E-208	0.49458635	0.528	0.286	2.185E-203	B cells	RASGRP2
1.291E-207	0.71011554	0.356	0.171	4.33E-203	B cells	PABPN1
1.799E-207	0.5901739	0.252	0.102	6.035E-203	B cells	MTMR1
4.815E-205	0.90455269	0.282	0.126	1.615E-200	B cells	CCT8
1.256E-204	0.44707474	0.939	0.709	4.213E-200	B cells	DDX5
2.093E-204	0.72048931	0.4	0.203	7.022E-200	B cells	EIF5A
3.121E-204	0.6598231	0.258	0.108	1.047E-199	B cells	RUBCN
4.424E-204	0.80142204	0.288	0.129	1.484E-199	B cells	FBXO7
9.045E-204	0.60152851	0.435	0.226	3.034E-199	B cells	EIF3K
2.203E-203	0.43014234	0.549	0.307	7.389E-199	B cells	NKTR
7.692E-203	0.62778174	0.301	0.136	2.58E-198	B cells	RAB3GAP1
1.35E-202	0.63101989	0.299	0.136	4.529E-198	B cells	SND1
2.044E-201	0.66460292	0.412	0.212	6.856E-197	B cells	OCIAD1
2.19E-201	0.49004752	0.318	0.144	7.346E-197	B cells	NGLY1
2.966E-199	0.48583581	0.432	0.222	9.95E-195	B cells	BMP2K
9.206E-199	0.2877714	0.653	0.356	3.088E-194	B cells	CAST
1.332E-198	0.7850692	0.261	0.111	4.466E-194	B cells	AL365361.1
4.12E-198	0.91215779	0.285	0.13	1.382E-193	B cells	CCT6A
4.719E-197	0.73454217	0.312	0.146	1.583E-192	B cells	CEP57
1.379E-196	0.66587458	0.349	0.17	4.625E-192	B cells	SREK1
1.483E-196	0.78227886	0.259	0.111	4.973E-192	B cells	OAS2
1.689E-196	0.45274933	0.578	0.329	5.666E-192	B cells	PSIP1
2.744E-196	0.66441689	0.349	0.17	9.203E-192	B cells	BRD7
6.874E-196	0.89664029	0.282	0.127	2.306E-191	B cells	PURA
1.087E-195	0.64421406	0.349	0.171	3.646E-191	B cells	XRCC6
2.181E-195	0.84305891	0.312	0.148	7.315E-191	B cells	PYURF
5.673E-195	0.81301471	0.285	0.129	1.903E-190	B cells	CYP20A1
8.256E-195	0.60964762	0.426	0.224	2.769E-190	B cells	SMC3
1.115E-194	0.72276912	0.289	0.132	3.742E-190	B cells	PRKX
2.555E-193	0.4409694	0.832	0.562	8.569E-189	B cells	HNRNPU
1.456E-192	0.56036501	0.432	0.229	4.885E-188	B cells	CRYBG1
1.561E-192	0.2806294	0.61	0.353	5.237E-188	B cells	LENG8
1.733E-192	0.57244367	0.394	0.201	5.813E-188	B cells	DENR
4.362E-192	0.8334164	0.305	0.145	1.463E-187	B cells	SNRPE
4.674E-192	0.60247805	0.422	0.219	1.568E-187	B cells	NDUFA4
8.911E-191	0.64658282	0.408	0.212	2.989E-186	B cells	POLR1D
9.975E-191	0.51218513	0.417	0.219	3.346E-186	B cells	TAF15
6.54E-190	0.61409615	0.37	0.188	2.194E-185	B cells	PTPN2

3.452E-188	0.49194672	0.371	0.185	1.158E-183	B cells	KLHL5
3.712E-188	0.49100071	0.566	0.325	1.245E-183	B cells	ATP6V1G1
5.322E-188	0.78364342	0.271	0.121	1.785E-183	B cells	DIS3
6.107E-188	0.88022104	0.285	0.132	2.048E-183	B cells	LRRC58
7.628E-188	0.54620555	0.381	0.193	2.559E-183	B cells	CTR9
8.66E-188	0.38561508	0.655	0.4	2.905E-183	B cells	UBE2D2
6.425E-186	0.50152033	0.557	0.321	2.155E-181	B cells	ISCU
8.634E-186	0.63819559	0.373	0.189	2.896E-181	B cells	RANBP2
1.031E-184	0.747021	0.263	0.116	3.459E-180	B cells	ZNF83
1.738E-184	0.48990369	0.315	0.151	5.829E-180	B cells	AMBRA1
6.963E-184	0.3958226	0.618	0.35	2.336E-179	B cells	EIF3A
2.827E-183	0.37586174	0.734	0.471	9.483E-179	B cells	BPTF
3.465E-183	0.5427942	0.426	0.227	1.162E-178	B cells	TES
3.684E-183	0.80785897	0.296	0.141	1.236E-178	B cells	SNRPB2
4.835E-183	0.33740287	0.52	0.294	1.622E-178	B cells	TMEM131L
1.205E-182	0.49269867	0.52	0.293	4.042E-178	B cells	PTGES3
1.213E-182	0.45244828	0.395	0.198	4.07E-178	B cells	RNASE6
1.307E-182	0.72466936	0.362	0.185	4.384E-178	B cells	NDUFB8
5.588E-182	0.48827463	0.336	0.162	1.874E-177	B cells	ELMSAN1
4.089E-181	0.49602272	0.402	0.21	1.372E-176	B cells	PTPN18
5.063E-181	0.5718319	0.333	0.161	1.698E-176	B cells	HMGN4
1.035E-180	0.68266364	0.259	0.115	3.471E-176	B cells	CWF19L2
1.623E-180	0.53815413	0.453	0.246	5.443E-176	B cells	PTBP1
4.295E-180	0.73059307	0.259	0.115	1.441E-175	B cells	UXT
1.452E-179	0.29064987	0.66	0.405	4.872E-175	B cells	UTRN
1.961E-179	0.71519387	0.256	0.114	6.579E-175	B cells	C2CD5
2.457E-179	0.40802039	0.486	0.268	8.242E-175	B cells	DCAF7
6.633E-179	0.78219494	0.296	0.141	2.225E-174	B cells	ATP5PO
1.365E-177	0.57028429	0.337	0.168	4.578E-173	B cells	ANAPC5
1.07E-176	0.59931618	0.293	0.136	3.588E-172	B cells	FAM53B
3.15E-176	0.81612621	0.294	0.141	1.057E-171	B cells	EIF2A
3.951E-176	0.67666117	0.294	0.139	1.325E-171	B cells	FAM133B
4.56E-176	0.38206552	0.481	0.267	1.53E-171	B cells	SMARCC1
9.581E-176	0.64800353	0.302	0.143	3.214E-171	B cells	NPAT
1.118E-175	0.72405731	0.289	0.136	3.752E-171	B cells	GABPA
1.519E-175	0.73408564	0.308	0.15	5.094E-171	B cells	ABCF1
3.496E-175	0.65013455	0.289	0.137	1.173E-170	B cells	MCUB
6.829E-175	0.41205097	0.444	0.235	2.291E-170	B cells	MLLT6
7.308E-175	0.35859032	0.559	0.321	2.451E-170	B cells	OST4
1.301E-174	0.739519	0.318	0.157	4.365E-170	B cells	RSL24D1
1.663E-174	0.78157433	0.262	0.121	5.579E-170	B cells	CUL5
7.054E-173	0.58883329	0.292	0.136	2.366E-168	B cells	ZNF397
2.106E-170	0.33341336	0.45	0.244	7.064E-166	B cells	EML4
4.843E-170	0.50554127	0.358	0.183	1.625E-165	B cells	CCDC88C
8.787E-170	0.70736493	0.276	0.13	2.948E-165	B cells	ARL5A
1.145E-169	0.31400456	0.369	0.187	3.841E-165	B cells	FAM13B
1.514E-169	0.55383691	0.332	0.166	5.078E-165	B cells	PNPLA8
7.689E-167	0.70434015	0.253	0.115	2.579E-162	B cells	DDX50
1.154E-166	0.42956144	0.388	0.204	3.869E-162	B cells	ADNP
1.393E-166	0.56203765	0.268	0.123	4.673E-162	B cells	MFN1

3.4E-166	0.58564867	0.332	0.168	1.14E-161	B cells	TMEM248
6.453E-166	0.3876696	0.776	0.517	2.165E-161	B cells	SF1
7.13E-166	0.58485979	0.384	0.205	2.391E-161	B cells	ZKSCAN1
8.929E-166	0.5815794	0.329	0.166	2.995E-161	B cells	DDX42
2.134E-165	0.69333171	0.334	0.17	7.159E-161	B cells	ATP5MC3
2.293E-165	0.38217279	0.649	0.396	7.691E-161	B cells	CLK1
9.114E-165	0.59115109	0.277	0.13	3.057E-160	B cells	NIPA2
2.169E-164	0.35378012	0.41	0.221	7.276E-160	B cells	CLINT1
6.828E-164	0.44901929	0.304	0.148	2.29E-159	B cells	RPRD2
8.606E-164	0.37660708	0.843	0.578	2.887E-159	B cells	EIF4G2
9.435E-164	0.60602533	0.329	0.166	3.165E-159	B cells	USP1
1.494E-163	0.58021441	0.282	0.133	5.013E-159	B cells	DUSP22
2.112E-163	0.6463287	0.318	0.16	7.085E-159	B cells	TRIP11
2.642E-163	0.49266663	0.393	0.205	8.861E-159	B cells	VAMP8
1.067E-162	0.36415651	0.527	0.31	3.581E-158	B cells	CDK13
7.108E-162	0.73806733	0.275	0.132	2.384E-157	B cells	NUTF2
1.606E-161	0.69025954	0.33	0.169	5.388E-157	B cells	NDUFB2
1.914E-161	0.65690353	0.267	0.127	6.419E-157	B cells	CRTC3
3.028E-161	0.57955226	0.365	0.193	1.016E-156	B cells	DNAJC8
1.887E-160	0.34078511	0.638	0.392	6.328E-156	B cells	PPIG
1.097E-159	0.54572816	0.358	0.189	3.681E-155	B cells	CS
2.658E-159	0.71488321	0.277	0.134	8.917E-155	B cells	ORMDL1
4.3E-159	0.48754286	0.307	0.151	1.442E-154	B cells	RNF41
1.122E-158	0.57669268	0.252	0.114	3.764E-154	B cells	APOBEC3G
1.407E-158	0.69992229	0.26	0.123	4.72E-154	B cells	CYB5B
1.83E-158	0.39668057	0.466	0.258	6.137E-154	B cells	PDIA3
2.315E-158	0.30980816	0.288	0.137	7.764E-154	B cells	EPSTI1
3.28E-158	0.42386889	0.273	0.128	1.1E-153	B cells	PTPN22
3.377E-158	0.72841421	0.269	0.13	1.133E-153	B cells	RNF187
6.694E-158	0.41818857	0.26	0.12	2.245E-153	B cells	LRCH3
7.102E-158	0.41099597	0.416	0.227	2.382E-153	B cells	AFF4
1.716E-156	0.44326489	0.279	0.13	5.755E-152	B cells	PHF1
5.624E-156	0.73476497	0.284	0.141	1.887E-151	B cells	EIF3M
6.097E-156	0.64169	0.268	0.128	2.045E-151	B cells	TRIM28
6.473E-156	0.45125845	0.39	0.21	2.171E-151	B cells	ZBTB44
1.879E-155	0.37118609	0.258	0.119	6.302E-151	B cells	CBLB
1.979E-155	0.44124919	0.52	0.302	6.639E-151	B cells	HNRNPF
5.746E-155	0.30275136	0.946	0.739	1.928E-150	B cells	GNAS
9.264E-155	0.64216725	0.306	0.154	3.107E-150	B cells	DNTTIP2
2.846E-153	0.66822187	0.283	0.14	9.547E-149	B cells	TRIM4
3.661E-153	0.5305967	0.336	0.174	1.228E-148	B cells	SNRNP200
2.586E-152	0.56440871	0.263	0.125	8.675E-148	B cells	MTF2
4.929E-152	0.65372004	0.305	0.155	1.653E-147	B cells	ERH
4.975E-152	0.588872	0.329	0.171	1.669E-147	B cells	MED1
5.774E-152	0.69212278	0.251	0.119	1.937E-147	B cells	CD84
5.964E-152	0.54179509	0.263	0.125	2E-147	B cells	LONP2
1.493E-151	0.34551203	0.371	0.199	5.008E-147	B cells	GPATCH8
3.742E-151	0.53471452	0.301	0.149	1.255E-146	B cells	DAXX
1.234E-150	0.53430801	0.271	0.13	4.139E-146	B cells	TRRAP
1.83E-150	0.32828119	0.734	0.495	6.138E-146	B cells	ITSN2

4.248E-150	0.40180237	0.396	0.22	1.425E-145	B cells	TTC17
4.18E-149	0.48288686	0.343	0.179	1.402E-144	B cells	RPA2
7.192E-149	0.48421141	0.318	0.16	2.413E-144	B cells	MARCKSL1
1.788E-148	0.39251182	0.332	0.169	5.998E-144	B cells	FAM117A
5.079E-148	0.62272148	0.281	0.139	1.704E-143	B cells	THAP12
1.063E-147	0.53563706	0.297	0.15	3.567E-143	B cells	YLPM1
2.604E-147	0.34596412	0.646	0.405	8.734E-143	B cells	HP1BP3
5.874E-147	0.31594371	0.442	0.252	1.97E-142	B cells	SOS1
7.367E-147	0.31056891	0.699	0.455	2.471E-142	B cells	ATRX
1.907E-146	0.39309344	0.307	0.154	6.396E-142	B cells	SNRNP70
2.186E-146	0.38271999	0.517	0.304	7.333E-142	B cells	U2AF2
2.269E-146	0.34338904	0.847	0.583	7.61E-142	B cells	SON
2.778E-146	0.44878429	0.294	0.146	9.317E-142	B cells	WDR33
3.293E-146	0.3334408	0.397	0.217	1.105E-141	B cells	VAV3
3.349E-146	0.34079049	0.533	0.318	1.123E-141	B cells	SMG1
3.569E-146	0.46534305	0.335	0.175	1.197E-141	B cells	ZNF131
5.435E-146	0.62122656	0.318	0.166	1.823E-141	B cells	CACYBP
5.553E-146	0.33334374	0.433	0.243	1.863E-141	B cells	SYNRG
6.946E-146	0.54988042	0.373	0.204	2.33E-141	B cells	SRP9
1.103E-145	0.57566444	0.254	0.122	3.699E-141	B cells	SMIM7
1.267E-145	0.52138688	0.277	0.137	4.251E-141	B cells	SF3A3
1.677E-145	0.64711376	0.267	0.131	5.624E-141	B cells	BTBD1
1.88E-145	0.60408631	0.251	0.119	6.307E-141	B cells	SUGP2
4.245E-145	0.6185131	0.255	0.122	1.424E-140	B cells	TRIM52
5.384E-145	0.3818683	0.454	0.256	1.806E-140	B cells	STK17A
5.709E-145	0.76297011	0.265	0.131	1.915E-140	B cells	ARL2BP
6.24E-145	0.67181312	0.256	0.123	2.093E-140	B cells	SKIL
6.413E-145	0.29508318	0.43	0.243	2.151E-140	B cells	EPS15
4.11E-144	0.25048437	0.443	0.249	1.379E-139	B cells	XAF1
4.809E-144	0.39855032	0.292	0.145	1.613E-139	B cells	POGZ
6.872E-143	0.52004161	0.305	0.156	2.305E-138	B cells	PSMC6
2.229E-142	0.34221702	0.483	0.285	7.476E-138	B cells	PUM1
2.858E-142	0.33607883	0.787	0.537	9.586E-138	B cells	SEPTIN7
3.277E-142	0.36711595	0.4	0.222	1.099E-137	B cells	RIOK3
3.465E-142	0.43689132	0.341	0.183	1.162E-137	B cells	USP47
3.637E-142	0.6427277	0.29	0.148	1.22E-137	B cells	USP16
3.725E-142	0.41152585	0.389	0.216	1.249E-137	B cells	PPP2R5E
1.355E-141	0.49985998	0.37	0.205	4.545E-137	B cells	TBCA
1.751E-140	0.54927349	0.344	0.186	5.874E-136	B cells	SNRPG
9.284E-140	0.43822698	0.37	0.203	3.114E-135	B cells	RBM27
6.557E-139	0.37694983	0.552	0.342	2.199E-134	B cells	B4GALT1
1.123E-138	0.45338966	0.274	0.135	3.767E-134	B cells	INO80D
3.334E-138	0.4323026	0.368	0.203	1.118E-133	B cells	PBRM1
3.647E-138	0.31778688	0.542	0.327	1.223E-133	B cells	CHD4
3.915E-138	0.70798797	0.26	0.13	1.313E-133	B cells	MESD
4.607E-138	0.40812045	0.302	0.154	1.545E-133	B cells	IRF3
1.566E-137	0.55710595	0.253	0.124	5.254E-133	B cells	OXR1
2.82E-137	0.33417474	0.371	0.201	9.458E-133	B cells	RNF19A
5.578E-137	0.29120607	0.305	0.155	1.871E-132	B cells	POU2F1
7.56E-137	0.41197641	0.396	0.223	2.536E-132	B cells	NUDT3

4.877E-136	0.33822991	0.417	0.236	1.636E-131	B cells	OGT
6.232E-136	0.53092135	0.337	0.183	2.091E-131	B cells	MAPK1IP1L
3.502E-135	0.38926113	0.395	0.22	1.175E-130	B cells	VPS35
6.399E-135	0.67467515	0.257	0.128	2.146E-130	B cells	THUMPD1
7.125E-135	0.56700889	0.296	0.154	2.39E-130	B cells	SCP2
9.4E-135	0.37103289	0.305	0.157	3.153E-130	B cells	ILRUN
1.083E-134	0.2714391	0.531	0.32	3.634E-130	B cells	AFTPH
2.315E-134	0.45434569	0.276	0.138	7.765E-130	B cells	PITPNB
5.476E-134	0.32295309	0.48	0.283	1.837E-129	B cells	CNOT1
9.605E-134	0.46975116	0.369	0.206	3.222E-129	B cells	SMARCC2
1.416E-133	0.51538532	0.343	0.188	4.75E-129	B cells	ERCC5
2.305E-133	0.43317399	0.395	0.223	7.732E-129	B cells	FXR1
1.204E-132	0.43653529	0.378	0.212	4.037E-128	B cells	MTIF3
1.353E-132	0.42413404	0.28	0.142	4.538E-128	B cells	CNOT4
2.919E-132	0.7129382	0.262	0.133	9.791E-128	B cells	SUMO3
3.704E-132	0.48761959	0.376	0.212	1.242E-127	B cells	SPCS1
5.743E-132	0.31137516	0.493	0.294	1.926E-127	B cells	NORAD
6.152E-132	0.60126522	0.251	0.125	2.064E-127	B cells	TMEM245
3.627E-131	0.29357899	0.528	0.322	1.217E-126	B cells	PPP4R3A
1.233E-130	0.36169709	0.46	0.27	4.136E-126	B cells	ZNF24
2.458E-130	0.52976989	0.324	0.175	8.244E-126	B cells	FAM204A
3.006E-130	0.5507964	0.259	0.129	1.008E-125	B cells	MFF
3.282E-130	0.32462609	0.508	0.31	1.101E-125	B cells	ZNF207
3.475E-130	0.28357887	0.448	0.259	1.166E-125	B cells	TMC8
5.789E-130	0.45065332	0.35	0.193	1.942E-125	B cells	USF2
9.75E-130	0.30659664	0.749	0.505	3.271E-125	B cells	BCLAF1
1.183E-129	0.3295585	0.622	0.395	3.969E-125	B cells	BRD2
1.352E-129	0.42916751	0.461	0.276	4.537E-125	B cells	RBM8A
1.697E-129	0.5323275	0.279	0.143	5.691E-125	B cells	TCP1
1.862E-129	0.29203417	0.435	0.254	6.247E-125	B cells	NEMF
2.59E-129	0.61189947	0.286	0.15	8.687E-125	B cells	GLUD1
3.113E-129	0.33444367	0.442	0.25	1.044E-124	B cells	M6PR
3.118E-129	0.42679486	0.251	0.122	1.046E-124	B cells	DSE
5.487E-129	0.37560322	0.449	0.262	1.84E-124	B cells	TMED10
5.539E-129	0.4616351	0.26	0.131	1.858E-124	B cells	PPP3CB
6.148E-129	0.44081041	0.371	0.209	2.062E-124	B cells	TMED5
5.254E-127	0.39365627	0.297	0.157	1.762E-122	B cells	SCFD1
1.089E-126	0.42468291	0.441	0.259	3.652E-122	B cells	ATP5PB
2.108E-126	0.5791273	0.251	0.125	7.071E-122	B cells	WHAMM
2.184E-126	0.38162494	0.351	0.194	7.327E-122	B cells	USF3
6.335E-126	0.53970813	0.254	0.128	2.125E-121	B cells	CLK2
6.575E-126	0.5042806	0.295	0.156	2.206E-121	B cells	EIF3J
2.312E-125	0.61975676	0.257	0.132	7.757E-121	B cells	DYNLL1
4.081E-125	0.47175639	0.308	0.166	1.369E-120	B cells	RBX1
6.085E-125	0.47817296	0.333	0.184	2.041E-120	B cells	SRSF1
1.303E-124	0.42134512	0.408	0.238	4.37E-120	B cells	TIAL1
2.315E-124	0.25151518	0.724	0.488	7.767E-120	B cells	SRRM2
2.459E-124	0.63084139	0.255	0.13	8.247E-120	B cells	KARS
3.037E-124	0.54074346	0.275	0.144	1.019E-119	B cells	AK2
4.253E-124	0.34288859	0.33	0.179	1.427E-119	B cells	DCAF8

8.797E-124	0.46905676	0.256	0.129	2.951E-119	B cells	RAB3GAP2
1.012E-123	0.41636615	0.285	0.149	3.396E-119	B cells	QRICH1
1.101E-123	0.47627065	0.31	0.168	3.693E-119	B cells	FNTA
1.235E-123	0.5675756	0.274	0.144	4.144E-119	B cells	UBP1
1.822E-123	0.5319035	0.278	0.145	6.11E-119	B cells	CASP8AP2
2.006E-122	0.40695017	0.335	0.185	6.73E-118	B cells	PRPF8
2.676E-122	0.6147279	0.289	0.155	8.975E-118	B cells	TUFM
2.738E-121	0.28024339	0.631	0.408	9.184E-117	B cells	THRAP3
3.275E-121	0.28207021	0.665	0.431	1.099E-116	B cells	DDX39B
5.829E-121	0.4320301	0.321	0.176	1.955E-116	B cells	ALKBH5
9.296E-121	0.4436469	0.286	0.151	3.118E-116	B cells	FIP1L1
2.19E-120	0.54203748	0.301	0.163	7.346E-116	B cells	COPZ1
1.126E-119	0.30154234	0.6	0.38	3.777E-115	B cells	CBX3
1.719E-119	0.51190846	0.289	0.155	5.765E-115	B cells	UBE2Z
3.488E-119	0.3502998	0.376	0.214	1.17E-114	B cells	CCNL2
1.878E-118	0.42062271	0.407	0.24	6.299E-114	B cells	UBE2V1
1.976E-118	0.34470033	0.401	0.233	6.628E-114	B cells	DDX3Y
3.285E-118	0.46117595	0.268	0.139	1.102E-113	B cells	ZNF430
7.807E-118	0.26706385	0.321	0.176	2.619E-113	B cells	DAPK1
8.204E-118	0.28751078	0.322	0.174	2.752E-113	B cells	ZMYND8
9.236E-118	0.5807213	0.251	0.129	3.098E-113	B cells	SNRPB
3.519E-116	0.29787192	0.541	0.336	1.18E-111	B cells	EIF5
1.745E-115	0.27260495	0.905	0.675	5.853E-111	B cells	HMGB1
9.603E-115	0.30097969	0.505	0.316	3.221E-110	B cells	PPP4R3B
3.467E-114	0.39482067	0.302	0.163	1.163E-109	B cells	DIDO1
3.851E-114	0.29158373	0.601	0.375	1.292E-109	B cells	ATP5MG
1.188E-113	0.56477086	0.258	0.136	3.986E-109	B cells	MTA2
1.821E-113	0.30691002	0.62	0.396	6.109E-109	B cells	SUMO2
1.998E-113	0.32986725	0.443	0.265	6.701E-109	B cells	HNRNPUL2
2.133E-113	0.37601037	0.426	0.255	7.156E-109	B cells	EIF2S2
1.33E-112	0.45001797	0.258	0.136	4.462E-108	B cells	CBFB
1.857E-112	0.28523583	0.566	0.362	6.23E-108	B cells	FUS
6.359E-112	0.2655065	0.941	0.787	2.133E-107	B cells	DDX17
1.12E-111	0.37919675	0.348	0.199	3.756E-107	B cells	LARP7
4.506E-111	0.28439018	0.523	0.334	1.511E-106	B cells	CEP350
4.633E-111	0.60273621	0.254	0.134	1.554E-106	B cells	SSR4
5.5E-111	0.31308812	0.508	0.309	1.845E-106	B cells	SLC25A3
9.605E-111	0.59639452	0.265	0.142	3.222E-106	B cells	EIF3G
1.218E-110	0.35281448	0.372	0.216	4.086E-106	B cells	CWC15
3.931E-110	0.33790778	0.4	0.239	1.318E-105	B cells	TNKS2
7.921E-110	0.27129968	0.368	0.212	2.657E-105	B cells	BRWD1
1.187E-109	0.52441544	0.255	0.134	3.982E-105	B cells	SMU1
4.728E-109	0.28442376	0.452	0.276	1.586E-104	B cells	PHF11
7.468E-109	0.36220074	0.341	0.197	2.505E-104	B cells	CNOT2
9.364E-109	0.26444902	0.393	0.231	3.141E-104	B cells	SUZ12
9.759E-109	0.53603482	0.253	0.134	3.274E-104	B cells	CTBP1
1.814E-108	0.25237588	0.273	0.145	6.084E-104	B cells	EHMT1
3.276E-108	0.41459156	0.282	0.153	1.099E-103	B cells	TOPBP1
2.277E-107	0.27202203	0.486	0.301	7.639E-103	B cells	RSRC2
4.189E-107	0.25115164	0.683	0.46	1.405E-102	B cells	DDX6

6.565E-107	0.34765066	0.343	0.196	2.202E-102	B cells	CHTOP
1.092E-106	0.45055893	0.259	0.137	3.663E-102	B cells	FAM76B
1.415E-106	0.27412675	0.409	0.242	4.746E-102	B cells	HERPUD1
3.632E-106	0.30250062	0.317	0.176	1.218E-101	B cells	ARPP19
3.871E-106	0.45234074	0.348	0.203	1.298E-101	B cells	DBI
4.996E-106	0.43627897	0.345	0.201	1.676E-101	B cells	RPN2
1.188E-105	0.36591432	0.367	0.215	3.986E-101	B cells	HADHA
7.099E-105	0.40074143	0.275	0.151	2.381E-100	B cells	ANKIB1
1.946E-103	0.48501057	0.262	0.142	6.526E-99	B cells	AGGF1
3.338E-103	0.26661479	0.34	0.197	1.1197E-98	B cells	STAT2
1.294E-102	0.26296625	0.443	0.273	4.3407E-98	B cells	DDX46
2.744E-102	0.45424984	0.299	0.17	9.2037E-98	B cells	TBRG1
7.16E-102	0.29921228	0.345	0.196	2.4016E-97	B cells	C1orf162
1.967E-101	0.41137521	0.299	0.17	6.5982E-97	B cells	DYNC1H1
4.784E-101	0.25013074	0.325	0.182	1.6048E-96	B cells	CBFA2T3
8.782E-101	0.51902156	0.288	0.163	2.9458E-96	B cells	NDUFB4
2.338E-100	0.4010671	0.349	0.206	7.8431E-96	B cells	MIA3
4.923E-100	0.41651945	0.264	0.144	1.6512E-95	B cells	RSBN1
1.152E-99	0.39053023	0.319	0.184	3.8653E-95	B cells	RIF1
2.268E-99	0.39322347	0.269	0.148	7.6085E-95	B cells	CHMP4A
4.207E-99	0.41618841	0.251	0.133	1.411E-94	B cells	ZNF101
5.147E-99	0.30268028	0.377	0.227	1.7265E-94	B cells	COPB1
5.55E-99	0.33114487	0.322	0.187	1.8615E-94	B cells	EFR3A
7.229E-99	0.37420942	0.325	0.188	2.425E-94	B cells	PCMTD2
7.479E-99	0.36358491	0.316	0.182	2.5085E-94	B cells	POLR2B
1.7109E-97	0.26902314	0.442	0.276	5.739E-93	B cells	SNX1
3.4198E-97	0.28657785	0.392	0.237	1.1471E-92	B cells	ZFP91
2.274E-96	0.2941892	0.291	0.164	7.6278E-92	B cells	RABGAP1
3.2248E-96	0.27541728	0.407	0.25	1.0817E-91	B cells	ZNF655
3.1435E-95	0.30875006	0.258	0.142	1.0544E-90	B cells	RETREG3
3.4351E-95	0.34395986	0.293	0.166	1.1522E-90	B cells	USP22
2.4249E-94	0.30113028	0.269	0.149	8.1337E-90	B cells	SSBP2
3.0682E-94	0.3382421	0.393	0.239	1.0292E-89	B cells	ATP5F1B
4.7107E-94	0.27890031	0.289	0.162	1.5801E-89	B cells	SIN3A
2.8355E-93	0.46750187	0.25	0.138	9.511E-89	B cells	ZBTB38
1.4196E-92	0.31381417	0.266	0.147	4.7616E-88	B cells	WDR48
1.9293E-92	0.35750138	0.284	0.162	6.4715E-88	B cells	CCNT2
9.1219E-92	0.30424867	0.361	0.217	3.0598E-87	B cells	PRKCSH
1.7251E-91	0.37375398	0.251	0.138	5.7866E-87	B cells	PRPF3
2.6168E-91	0.4153458	0.292	0.17	8.7774E-87	B cells	SMC1A
1.171E-89	0.28740723	0.308	0.18	3.9279E-85	B cells	SFSWAP
1.2053E-89	0.34525991	0.277	0.157	4.043E-85	B cells	HPS1
1.6424E-89	0.33764722	0.337	0.203	5.509E-85	B cells	CIAO1
3.0164E-89	0.29813237	0.341	0.204	1.0118E-84	B cells	SERPINB9
5.0092E-89	0.40375376	0.271	0.155	1.6802E-84	B cells	PTPN11
3.1502E-88	0.28489	0.576	0.384	1.0567E-83	B cells	SUB1
4.2237E-88	0.26269274	0.303	0.175	1.4168E-83	B cells	SEPHS2
2.5957E-87	0.36690555	0.272	0.155	8.7069E-83	B cells	SMC4
2.9322E-86	0.41928	0.3	0.178	9.8355E-82	B cells	COX7A2L
3.4287E-86	0.25170859	0.333	0.199	1.1501E-81	B cells	ADPGK

4.3383E-86	0.29802811	0.411	0.26	1.4552E-81	B cells	ARL6IP1
2.9232E-84	0.25167808	0.451	0.288	9.8053E-80	B cells	CHCHD2
5.7499E-84	0.33045857	0.263	0.151	1.9287E-79	B cells	DAP3
5.8576E-84	0.26324881	0.293	0.171	1.9648E-79	B cells	TAF1
2.5756E-83	0.33521872	0.261	0.149	8.6392E-79	B cells	NSRP1
4.3689E-82	0.35610991	0.285	0.167	1.4655E-77	B cells	TMED4
1.5024E-81	0.27899372	0.272	0.158	5.0394E-77	B cells	FAM168B
1.6653E-80	0.34793595	0.267	0.156	5.5859E-76	B cells	PPM1G
3.2369E-79	0.36841585	0.274	0.161	1.0858E-74	B cells	YWHAG
4.1753E-79	0.29374877	0.254	0.147	1.4005E-74	B cells	TMEM87A
1.5481E-78	0.3788177	0.292	0.176	5.1927E-74	B cells	PARK7
5.0397E-78	0.26441512	0.267	0.157	1.6905E-73	B cells	KPNA6
1.883E-77	0.27188108	0.384	0.244	6.316E-73	B cells	ATP5ME
7.8039E-77	0.29965079	0.266	0.155	2.6177E-72	B cells	TINF2
1.3923E-76	0.41868123	0.253	0.149	4.6703E-72	B cells	ATP5F1C
3.1829E-76	0.29668252	0.281	0.168	1.0676E-71	B cells	USO1
1.3751E-75	0.26914741	0.338	0.211	4.6124E-71	B cells	REST
1.2337E-74	0.31204718	0.33	0.207	4.1383E-70	B cells	LPXN
2.008E-71	0.25442538	0.334	0.209	6.7354E-67	B cells	ATP5IF1
2.3342E-70	0.42106312	0.268	0.163	7.8295E-66	B cells	PSMB2
9.5668E-70	0.29859822	0.286	0.176	3.209E-65	B cells	CCDC59
1.768E-69	0.27915377	0.265	0.159	5.9304E-65	B cells	UBQLN1
2.1012E-68	0.28533397	0.275	0.169	7.0482E-64	B cells	TPP2
2.0029E-63	0.30711829	0.255	0.156	6.7185E-59	B cells	DNAJC13
3.8902E-63	0.29302642	0.274	0.17	1.3049E-58	B cells	NARS
2.5029E-61	0.32892629	0.261	0.163	8.3956E-57	B cells	FAM78A
9.8461E-59	0.3182841	0.267	0.167	3.3027E-54	B cells	SSR3
1.9674E-57	0.29903867	0.255	0.159	6.5991E-53	B cells	CCT5
7.6463E-56	0.55085267	0.311	0.211	2.5648E-51	B cells	ITGB1
0	8.97239114	0.946	0.018	0	Plasma cells	JCHAIN
0	8.47568101	0.381	0.008	0	Plasma cells	IGHA1
0	8.30005164	0.305	0.013	0	Plasma cells	IGLC2
0	8.17762465	0.826	0.056	0	Plasma cells	IGKC
0	7.76406924	0.398	0.01	0	Plasma cells	IGLC1
0	6.90307115	0.91	0.038	0	Plasma cells	TXNDC5
0	6.24899381	0.935	0.031	0	Plasma cells	ITM2C
0	5.84085357	0.259	0.002	0	Plasma cells	IGHA2
0	5.76586261	0.88	0.02	0	Plasma cells	RHEX
0	5.20631716	0.847	0.005	0	Plasma cells	MZB1
0	5.20060726	0.777	0.044	0	Plasma cells	CCDC50
0	5.15052552	0.932	0.05	0	Plasma cells	TCF4
0	4.94734929	0.828	0.003	0	Plasma cells	DERL3
0	4.87624449	0.534	0.002	0	Plasma cells	AC009570.2
0	4.78372373	0.504	0.005	0	Plasma cells	SERPINF1
0	4.75312413	0.458	0.011	0	Plasma cells	LILRA4
0	4.75190268	0.542	0.04	0	Plasma cells	IGHM
0	4.63472665	0.842	0.082	0	Plasma cells	SEC11C
0	4.56842114	0.471	0.01	0	Plasma cells	PLD4
0	4.51428435	0.496	0.016	0	Plasma cells	SPIB
0	4.37832296	0.469	0.041	0	Plasma cells	GZMB

0	4.34217408	0.632	0.02	0	Plasma cells	IRF4
0	4.2982676	0.401	0.003	0	Plasma cells	AC023590.1
0	4.27589758	0.485	0.002	0	Plasma cells	TNFRSF17
0	4.20992006	0.992	0.308	0	Plasma cells	HSP90B1
0	4.13714765	0.504	0.031	0	Plasma cells	TENT5C
0	4.05944379	0.403	0.004	0	Plasma cells	LINC00996
0	4.01269169	0.417	0.003	0	Plasma cells	MAP1A
0	3.98807454	0.899	0.069	0	Plasma cells	SLAMF7
0	3.9382846	0.812	0.069	0	Plasma cells	PDIA4
0	3.90856361	0.774	0.019	0	Plasma cells	BLNK
0	3.85027067	0.398	0.002	0	Plasma cells	DNASE1L3
0	3.82610476	0.88	0.136	0	Plasma cells	XBP1
0	3.78442133	0.52	0.04	0	Plasma cells	NIBAN3
0	3.73296021	0.58	0.056	0	Plasma cells	BCL11A
0	3.71447967	0.493	0.018	0	Plasma cells	POU2AF1
0	3.71011428	0.512	0.015	0	Plasma cells	TSPAN13
0	3.64001876	0.629	0.041	0	Plasma cells	PPP1R14B
0	3.63801078	0.905	0.136	0	Plasma cells	SSR4
0	3.60493584	0.785	0.05	0	Plasma cells	C12orf75
0	3.59818324	0.433	0.007	0	Plasma cells	CLEC4C
0	3.5181333	0.373	0.007	0	Plasma cells	LINC01374
0	3.51498725	0.559	0.033	0	Plasma cells	TLR7
0	3.51359439	0.837	0.072	0	Plasma cells	SELENOS
0	3.51130751	0.932	0.081	0	Plasma cells	SEL1L3
0	3.48699888	0.845	0.096	0	Plasma cells	LMAN1
0	3.47296574	0.428	0.008	0	Plasma cells	PTPRS
0	3.46591108	0.371	0.002	0	Plasma cells	TNFRSF21
0	3.42711779	0.907	0.142	0	Plasma cells	PPIB
0	3.41059682	0.744	0.019	0	Plasma cells	COBLL1
0	3.37890196	0.357	0.006	0	Plasma cells	PHEX
0	3.35690445	0.45	0.003	0	Plasma cells	SCAMP5
0	3.34983891	0.883	0.062	0	Plasma cells	CXXC5
0	3.3342475	0.924	0.168	0	Plasma cells	SSR3
0	3.29461532	0.401	0.006	0	Plasma cells	NT5DC2
0	3.29111701	0.58	0.04	0	Plasma cells	STMN1
0	3.24811808	0.575	0.011	0	Plasma cells	SLC7A5
0	3.24253032	0.332	0.005	0	Plasma cells	NRP1
0	3.23326548	0.36	0.008	0	Plasma cells	CUX2
0	3.22556163	0.458	0.015	0	Plasma cells	CPNE5
0	3.214105	0.91	0.167	0	Plasma cells	PEBP1
0	3.2088529	0.684	0.047	0	Plasma cells	SLC1A5
0	3.20160074	0.341	0.004	0	Plasma cells	AC007381.1
0	3.19627235	0.616	0.054	0	Plasma cells	IDH3A
0	3.16592407	0.488	0.026	0	Plasma cells	ABHD15
0	3.13997983	0.766	0.074	0	Plasma cells	MANF
0	3.13815695	0.36	0.005	0	Plasma cells	PACSIN1
0	3.13067784	0.733	0.044	0	Plasma cells	PALM2-AKAP2
0	3.12077431	0.256	0.006	0	Plasma cells	LINC01478
0	3.11423706	0.94	0.153	0	Plasma cells	SEC61B
0	3.10290193	0.346	0.001	0	Plasma cells	LAMP5

0	3.10123696	0.54	0.048	0	Plasma cells	OPN3
0	3.09290355	0.621	0.041	0	Plasma cells	CD79A
0	3.0928544	0.624	0.014	0	Plasma cells	ST6GALNAC4
0	3.04462699	0.351	0.01	0	Plasma cells	SCN9A
0	3.04265865	0.499	0.039	0	Plasma cells	LILRB4
0	3.03139248	0.894	0.136	0	Plasma cells	SEC61G
0	3.03062767	0.635	0.072	0	Plasma cells	TXNDC11
0	3.01637858	0.335	0.013	0	Plasma cells	PLXNA4
0	3.00988294	0.736	0.05	0	Plasma cells	HYOU1
0	3.0066371	0.967	0.214	0	Plasma cells	SPCS1
0	3.00552188	0.872	0.164	0	Plasma cells	PDIA6
0	3.00544721	0.488	0.037	0	Plasma cells	MANEA
0	2.99654454	0.49	0.019	0	Plasma cells	DUSP5
0	2.99358763	0.812	0.096	0	Plasma cells	METTL7A
0	2.99236119	0.831	0.085	0	Plasma cells	OSTC
0	2.9546681	0.332	0.015	0	Plasma cells	PRXL2A
0	2.94715825	0.523	0.043	0	Plasma cells	CD38
0	2.92959929	0.627	0.049	0	Plasma cells	MYDGF
0	2.90590858	0.569	0.047	0	Plasma cells	VEGFB
0	2.90391208	0.678	0.021	0	Plasma cells	DENND5B
0	2.89849464	0.311	0	0	Plasma cells	AC097375.1
0	2.88775749	0.48	0.048	0	Plasma cells	CYB561A3
0	2.86805745	0.504	0.032	0	Plasma cells	ZFAT
0	2.8336749	0.362	0.015	0	Plasma cells	TUBB6
0	2.82573583	0.678	0.053	0	Plasma cells	CNP
0	2.8235871	0.441	0.011	0	Plasma cells	SLC1A4
0	2.81128416	0.401	0.019	0	Plasma cells	AHI1
0	2.79861114	0.322	0.007	0	Plasma cells	TRAF4
0	2.78759701	0.289	0.003	0	Plasma cells	CIB2
0	2.78537066	0.433	0.036	0	Plasma cells	CLN8
0	2.77387322	0.409	0.015	0	Plasma cells	TLR9
0	2.75957568	0.384	0.016	0	Plasma cells	DAB2
0	2.74185492	0.371	0.006	0	Plasma cells	LGMN
0	2.72813291	0.668	0.079	0	Plasma cells	ERLEC1
0	2.72239253	0.477	0.026	0	Plasma cells	C12orf45
0	2.72011803	0.297	0.001	0	Plasma cells	CAV1
0	2.71897179	0.534	0.028	0	Plasma cells	SPATS2
0	2.716573	0.349	0.025	0	Plasma cells	CMKLR1
0	2.7157003	0.681	0.064	0	Plasma cells	DNAJB9
0	2.70763057	0.292	0.001	0	Plasma cells	PPM1J
0	2.69681411	0.684	0.048	0	Plasma cells	TMEM19
0	2.69346182	0.431	0.027	0	Plasma cells	PRDX4
0	2.67564205	0.289	0.008	0	Plasma cells	PERP
0	2.6465391	0.926	0.203	0	Plasma cells	RPN2
0	2.64648112	0.305	0.009	0	Plasma cells	TPM2
0	2.63024584	0.807	0.103	0	Plasma cells	GNL3
0	2.60270673	0.357	0.017	0	Plasma cells	FLT3
0	2.5834038	0.567	0.045	0	Plasma cells	EAF2
0	2.57554027	0.368	0.023	0	Plasma cells	CXCR3
0	2.56533265	0.343	0.009	0	Plasma cells	PMEPA1

0	2.55075759	0.27	0.008	0	Plasma cells	SLC35F3
0	2.51524642	0.251	0.003	0	Plasma cells	PTCRA
0	2.50816901	0.684	0.086	0	Plasma cells	SCARB2
0	2.50268432	0.422	0.031	0	Plasma cells	AC007952.4
0	2.49990156	0.512	0.038	0	Plasma cells	ADA
0	2.49839095	0.501	0.038	0	Plasma cells	TEX14
0	2.48174287	0.82	0.11	0	Plasma cells	MLEC
0	2.46937575	0.275	0.004	0	Plasma cells	LINC02812
0	2.46372779	0.916	0.16	0	Plasma cells	HLA-DPA1
0	2.45598585	0.455	0.021	0	Plasma cells	BCL7A
0	2.45056676	0.76	0.121	0	Plasma cells	RRBP1
0	2.44538453	0.594	0.046	0	Plasma cells	HSPA13
0	2.44235749	0.362	0.025	0	Plasma cells	WAKMAR2
0	2.44182783	0.646	0.063	0	Plasma cells	DNAJB11
0	2.43477816	0.439	0.02	0	Plasma cells	IL3RA
0	2.42624712	0.319	0.017	0	Plasma cells	MCOLN2
0	2.39948565	0.504	0.046	0	Plasma cells	TSPAN3
0	2.39870486	0.768	0.106	0	Plasma cells	LIME1
0	2.37862302	0.251	0.001	0	Plasma cells	AC004846.1
0	2.37386286	0.278	0.004	0	Plasma cells	SH3BP4
0	2.37169686	0.665	0.064	0	Plasma cells	ALG2
0	2.37059888	0.649	0.062	0	Plasma cells	TCF3
0	2.36158505	0.387	0.018	0	Plasma cells	RASGRP3
0	2.35918519	0.33	0.008	0	Plasma cells	NUGGC
0	2.3388782	0.406	0.024	0	Plasma cells	FABP5
0	2.3317885	0.534	0.048	0	Plasma cells	PLPP5
0	2.32392994	0.373	0.011	0	Plasma cells	PNOC
0	2.32169311	0.752	0.094	0	Plasma cells	GLO1
0	2.32153745	0.869	0.158	0	Plasma cells	TMED9
0	2.31526361	0.518	0.048	0	Plasma cells	DAAM1
0	2.31510801	0.275	0.006	0	Plasma cells	IFNLR1
0	2.31093592	0.414	0.026	0	Plasma cells	SDF2L1
0	2.30959219	0.433	0.033	0	Plasma cells	PFKFB2
0	2.29592464	0.272	0.01	0	Plasma cells	SSPN
0	2.28447349	0.439	0.032	0	Plasma cells	TRAM2
0	2.26755984	0.346	0.02	0	Plasma cells	MYO1E
0	2.26742013	0.425	0.032	0	Plasma cells	NME1
0	2.25843841	0.482	0.048	0	Plasma cells	CRELD2
0	2.25006996	0.839	0.14	0	Plasma cells	ZNF706
0	2.24763354	0.561	0.054	0	Plasma cells	EIF2AK4
0	2.244027	0.281	0.01	0	Plasma cells	FZD3
0	2.22472423	0.431	0.03	0	Plasma cells	DANCR
0	2.22078219	0.264	0.002	0	Plasma cells	GAS6
0	2.21367519	0.275	0.01	0	Plasma cells	CLIC3
0	2.21051467	0.313	0.009	0	Plasma cells	AC008074.2
0	2.20844799	0.847	0.143	0	Plasma cells	CDK2AP2
0	2.20626374	0.58	0.066	0	Plasma cells	N4BP2
0	2.20426485	0.564	0.047	0	Plasma cells	TFRC
0	2.19745398	0.48	0.029	0	Plasma cells	SETBP1
0	2.18177047	0.52	0.049	0	Plasma cells	SRPRB

0	2.18166808	0.602	0.056	0	Plasma cells	TBC1D9
0	2.17870957	0.278	0.005	0	Plasma cells	SLC41A2
0	2.16787217	0.251	0	0	Plasma cells	MIXL1
0	2.16698167	0.362	0.018	0	Plasma cells	FCER1A
0	2.16191735	0.455	0.042	0	Plasma cells	SRM
0	2.14095773	0.262	0.003	0	Plasma cells	BMP8B
0	2.13150321	0.548	0.043	0	Plasma cells	STT3A
0	2.13055468	0.749	0.112	0	Plasma cells	NUS1
0	2.12276962	0.441	0.026	0	Plasma cells	CDCA7L
0	2.11092777	0.689	0.09	0	Plasma cells	FGD2
0	2.10598158	0.436	0.026	0	Plasma cells	CD180
0	2.09220148	0.499	0.036	0	Plasma cells	CEP128
0	2.08624969	0.567	0.049	0	Plasma cells	TPD52
0	2.08371033	0.428	0.037	0	Plasma cells	RAB30
0	2.07396979	0.452	0.04	0	Plasma cells	AARS
0	2.06638508	0.275	0.003	0	Plasma cells	MYBL2
0	2.06604128	0.379	0.027	0	Plasma cells	CKS2
0	2.06367794	0.643	0.067	0	Plasma cells	COMMD3
0	2.03674636	0.455	0.04	0	Plasma cells	ALG5
0	2.02754021	0.575	0.042	0	Plasma cells	ODC1
0	2.02615421	0.526	0.046	0	Plasma cells	EIF2AK3
0	2.02433158	0.768	0.077	0	Plasma cells	GNG7
0	2.02326453	0.493	0.037	0	Plasma cells	SEC61A1
0	1.99622865	0.54	0.047	0	Plasma cells	LIMD1
0	1.99168029	0.564	0.057	0	Plasma cells	SEPTIN11
0	1.98601592	0.343	0.019	0	Plasma cells	PLAAT3
0	1.98259951	0.313	0.018	0	Plasma cells	PDXP
0	1.98094213	0.526	0.038	0	Plasma cells	BTLA
0	1.97753035	0.569	0.063	0	Plasma cells	CALU
0	1.96799965	0.591	0.065	0	Plasma cells	CREB3L2
0	1.96708284	0.537	0.058	0	Plasma cells	PREB
0	1.95205411	0.458	0.027	0	Plasma cells	DCPS
0	1.9415724	0.327	0.016	0	Plasma cells	CLECL1
0	1.93948833	0.387	0.023	0	Plasma cells	GPX7
0	1.93176822	0.837	0.139	0	Plasma cells	SND1
0	1.9256607	0.272	0.003	0	Plasma cells	CHPF
0	1.92339541	0.346	0.015	0	Plasma cells	PDIA5
0	1.89935671	0.36	0.022	0	Plasma cells	PDLIM1
0	1.89777614	0.436	0.037	0	Plasma cells	B4GALT3
0	1.89704929	0.395	0.031	0	Plasma cells	IFNG-AS1
0	1.89126147	0.264	0.013	0	Plasma cells	FCRL5
0	1.88854575	0.575	0.061	0	Plasma cells	STRBP
0	1.88195546	0.251	0.004	0	Plasma cells	TNFRSF13B
0	1.87777976	0.561	0.064	0	Plasma cells	SLC33A1
0	1.87133718	0.545	0.061	0	Plasma cells	MGAT2
0	1.86842284	0.589	0.069	0	Plasma cells	CCDC32
0	1.83614883	0.267	0.014	0	Plasma cells	SYCP2L
0	1.82055782	0.46	0.04	0	Plasma cells	SMARCB1
0	1.81223564	0.341	0.017	0	Plasma cells	C11orf24
0	1.74414391	0.335	0.024	0	Plasma cells	PGM3

0	1.71534265	0.267	0.014	0	Plasma cells	UCK2
0	1.69007286	0.455	0.032	0	Plasma cells	MCUR1
0	1.65356456	0.523	0.054	0	Plasma cells	CHST12
0	1.64551514	0.545	0.055	0	Plasma cells	HLA-DQA1
0	1.6161671	0.264	0.01	0	Plasma cells	GMPPB
0	1.59884537	0.297	0.013	0	Plasma cells	E2F5
0	1.58124133	0.311	0.016	0	Plasma cells	MGLL
0	1.57959874	0.504	0.045	0	Plasma cells	MAGED1
0	1.51815463	0.392	0.03	0	Plasma cells	SMPD3
0	1.51764425	0.52	0.054	0	Plasma cells	TMEM156
0	1.45141816	0.302	0.018	0	Plasma cells	C11orf80
0	1.43684379	0.292	0.017	0	Plasma cells	MYO1D
5.291E-308	2.03188112	0.646	0.085	1.775E-303	Plasma cells	IER3IP1
6.915E-308	1.67093285	0.281	0.018	2.32E-303	Plasma cells	ENTPD7
2.162E-306	2.50527347	0.774	0.124	7.253E-302	Plasma cells	PPM1K
2.378E-306	1.79568812	0.463	0.045	7.977E-302	Plasma cells	PAICS
3.437E-305	2.09592992	0.365	0.029	1.153E-300	Plasma cells	TEX2
4.674E-305	2.15569707	0.319	0.023	1.568E-300	Plasma cells	NEK8
4.97E-303	3.31634604	0.553	0.069	1.667E-298	Plasma cells	FKBP11
1.806E-302	1.68871588	0.42	0.038	6.058E-298	Plasma cells	ADGRG5
1.077E-298	1.93596358	0.52	0.057	3.613E-294	Plasma cells	CSNK1E
1.3E-298	1.59130733	0.289	0.019	4.362E-294	Plasma cells	ZNF593
1.431E-297	1.97119334	0.401	0.035	4.801E-293	Plasma cells	ZNF275
9.852E-297	2.17966844	0.785	0.131	3.305E-292	Plasma cells	MESD
2.217E-296	1.68152584	0.259	0.016	7.435E-292	Plasma cells	SNRNP25
5.485E-295	1.57007286	0.548	0.062	1.84E-290	Plasma cells	SIDT1
1.198E-294	1.87964493	0.918	0.176	4.017E-290	Plasma cells	MEF2C
1.607E-294	1.82696346	0.327	0.024	5.392E-290	Plasma cells	ALDH5A1
1.183E-293	2.14222789	0.45	0.045	3.969E-289	Plasma cells	PTMS
2.236E-293	2.43599678	0.717	0.112	7.502E-289	Plasma cells	CLPTM1L
1.057E-292	1.88850026	0.441	0.043	3.545E-288	Plasma cells	UBA5
4.338E-291	1.86645009	0.428	0.041	1.455E-286	Plasma cells	PARVB
5.566E-290	2.94605211	0.948	0.245	1.867E-285	Plasma cells	HERPUD1
1.977E-289	2.2731804	0.54	0.065	6.632E-285	Plasma cells	GPATCH11
2.3E-288	2.0487122	0.886	0.164	7.715E-284	Plasma cells	HLA-DRB1
8.523E-288	2.06109209	0.798	0.136	2.859E-283	Plasma cells	P4HB
5.491E-285	1.84300319	0.684	0.099	1.842E-280	Plasma cells	ILF2
1.356E-283	2.31292387	0.864	0.168	4.549E-279	Plasma cells	NME2
1.44E-283	1.72106906	0.545	0.065	4.83E-279	Plasma cells	RAP2A
6.651E-283	1.76495238	0.466	0.049	2.231E-278	Plasma cells	CLIC4
1.151E-282	1.57013256	0.286	0.02	3.859E-278	Plasma cells	LINC02245
1.38E-282	2.01352884	0.401	0.037	4.63E-278	Plasma cells	SCRN1
3.639E-282	1.65342246	0.433	0.042	1.221E-277	Plasma cells	REXO2
1.771E-281	2.58651556	0.926	0.219	5.941E-277	Plasma cells	SPCS2
3.671E-281	1.93893462	0.678	0.1	1.231E-276	Plasma cells	GORASP2
2.126E-280	1.98598763	0.548	0.067	7.132E-276	Plasma cells	PRMT1
4.032E-279	2.14776023	0.61	0.084	1.352E-274	Plasma cells	MRPS24
6.073E-279	1.74415795	0.349	0.029	2.037E-274	Plasma cells	ZDHHC4
4.766E-278	2.21703085	0.319	0.025	1.599E-273	Plasma cells	CLCN5
5.448E-277	2.02413547	0.556	0.07	1.827E-272	Plasma cells	DDOST

6.494E-277	2.41323882	0.796	0.145	2.178E-272	Plasma cells	EDEM1
9.47E-277	1.78702485	0.411	0.04	3.176E-272	Plasma cells	RPL22L1
5.065E-275	1.78223109	0.474	0.052	1.699E-270	Plasma cells	ARL1
5.339E-275	1.68883229	0.365	0.032	1.791E-270	Plasma cells	GMPPA
7.511E-275	1.94054429	0.782	0.134	2.519E-270	Plasma cells	DYNLL1
1.776E-274	2.60310095	0.967	0.279	5.956E-270	Plasma cells	TRAM1
5.189E-273	2.12868674	0.689	0.107	1.741E-268	Plasma cells	SMC6
1.22E-272	1.85487032	0.51	0.06	4.093E-268	Plasma cells	SLC35B1
2.91E-272	2.22177582	0.638	0.093	9.761E-268	Plasma cells	CYCS
9.116E-272	1.83283433	0.362	0.032	3.058E-267	Plasma cells	SLC2A1
1.959E-271	2.86344324	0.967	0.271	6.572E-267	Plasma cells	CALR
9.012E-271	2.11792492	0.815	0.149	3.023E-266	Plasma cells	MDFIC
1.358E-270	2.0758156	0.918	0.191	4.555E-266	Plasma cells	ADA2
5.815E-270	2.00447212	0.36	0.032	1.951E-265	Plasma cells	HLA-DOA
6.114E-270	2.46811811	0.428	0.045	2.051E-265	Plasma cells	FMNL3
6.978E-270	1.95858467	0.313	0.024	2.341E-265	Plasma cells	ZDHHC14
7.88E-270	4.01236764	0.616	0.098	2.643E-265	Plasma cells	UGCG
8.364E-270	2.02068508	0.55	0.07	2.806E-265	Plasma cells	ATP5MC1
1.63E-268	1.27888155	0.452	0.047	5.468E-264	Plasma cells	DIPK1A
1.666E-268	1.74520416	0.425	0.043	5.587E-264	Plasma cells	MIF4GD
1.991E-268	5.55638872	0.542	0.08	6.68E-264	Plasma cells	IRF8
2.062E-268	2.20072483	0.368	0.034	6.916E-264	Plasma cells	GRAMD1B
8.807E-266	1.71023628	0.616	0.086	2.954E-261	Plasma cells	OAS1
2.082E-265	1.71042291	0.332	0.028	6.985E-261	Plasma cells	AHCY
3.04E-265	1.7864447	0.409	0.04	1.02E-260	Plasma cells	DSN1
8.449E-264	1.62472417	0.488	0.056	2.834E-259	Plasma cells	ARMCX3
2.326E-260	1.86701633	0.594	0.082	7.801E-256	Plasma cells	AKR1A1
2.372E-260	1.69012399	0.362	0.033	7.958E-256	Plasma cells	HLA-DQA2
4.69E-260	2.36149706	0.956	0.265	1.573E-255	Plasma cells	TMED10
2.016E-259	1.89232736	0.45	0.049	6.762E-255	Plasma cells	TMA16
9.828E-259	1.44506374	0.431	0.045	3.296E-254	Plasma cells	TMEM263
1.786E-257	2.04490041	0.858	0.173	5.992E-253	Plasma cells	ERP29
2.037E-255	1.43510666	0.294	0.022	6.832E-251	Plasma cells	BLK
6.921E-255	2.68645673	0.349	0.033	2.322E-250	Plasma cells	NREP
7.015E-255	1.82216741	0.428	0.046	2.353E-250	Plasma cells	HIGD1A
8.387E-255	2.02662252	0.444	0.05	2.813E-250	Plasma cells	SHMT2
1.178E-254	1.88525763	0.58	0.081	3.95E-250	Plasma cells	GGA2
5.315E-254	1.85067119	0.76	0.132	1.783E-249	Plasma cells	STARD7
1.236E-253	2.05041898	0.466	0.054	4.146E-249	Plasma cells	PHB
2.838E-253	2.18404343	0.866	0.19	9.521E-249	Plasma cells	SRP72
1.039E-252	1.68658909	0.42	0.044	3.484E-248	Plasma cells	BET1
1.953E-252	1.90456337	0.578	0.081	6.552E-248	Plasma cells	LRRC59
3.366E-252	2.06061326	0.447	0.051	1.129E-247	Plasma cells	MTHFD2
6.9E-251	3.07783357	0.719	0.135	2.315E-246	Plasma cells	IRF7
3.667E-250	2.7575747	0.948	0.277	1.23E-245	Plasma cells	UBE2J1
1.203E-248	2.13298715	0.779	0.149	4.034E-244	Plasma cells	KDELR1
4.596E-246	2.07227037	0.736	0.133	1.542E-241	Plasma cells	PARP1
1.869E-245	1.61116426	0.42	0.045	6.269E-241	Plasma cells	FAM3C
1.041E-244	2.45249241	0.507	0.068	3.49E-240	Plasma cells	CCR2
2.208E-244	1.77988374	0.33	0.029	7.408E-240	Plasma cells	ATG101

6.594E-244	2.17385581	0.831	0.173	2.212E-239	Plasma cells	ATP5MC3
4.811E-243	1.97018198	0.722	0.128	1.614E-238	Plasma cells	COX5A
1.03E-242	2.0287928	0.858	0.185	3.456E-238	Plasma cells	ATP5F1A
1.45E-242	2.01480672	0.608	0.094	4.862E-238	Plasma cells	KDELR2
2.661E-242	2.465897	0.635	0.105	8.926E-238	Plasma cells	CD2AP
3.157E-241	1.73723758	0.49	0.061	1.059E-236	Plasma cells	SYVN1
6.589E-240	2.15696292	0.785	0.157	2.21E-235	Plasma cells	NUCB2
1.951E-239	2.03286398	0.523	0.071	6.544E-235	Plasma cells	KCTD5
1.362E-238	1.76654203	0.621	0.095	4.57E-234	Plasma cells	HMGN3
1.815E-238	1.42722976	0.3	0.025	6.089E-234	Plasma cells	LINC02384
5.184E-238	1.55074689	0.349	0.033	1.739E-233	Plasma cells	DPAGT1
7.361E-238	1.37961331	0.444	0.051	2.469E-233	Plasma cells	HIBCH
1.373E-237	1.75019035	0.49	0.062	4.604E-233	Plasma cells	TMEM214
3.007E-237	1.73916186	0.493	0.063	1.009E-232	Plasma cells	TMEM256
3.452E-237	1.85012488	0.823	0.166	1.158E-232	Plasma cells	COPZ1
4.228E-237	2.20584129	0.984	0.296	1.418E-232	Plasma cells	RPLP0
2.53E-236	1.89248591	0.499	0.065	8.485E-232	Plasma cells	SLFN11
8.982E-236	1.9037344	0.482	0.062	3.013E-231	Plasma cells	FDX1
2.28E-235	1.88397794	0.616	0.095	7.649E-231	Plasma cells	LILRB1
1.256E-234	1.52240628	0.433	0.05	4.215E-230	Plasma cells	TMEM39A
1.557E-234	1.91051377	0.883	0.2	5.223E-230	Plasma cells	KRTCAP2
4.146E-233	1.74054242	0.515	0.069	1.391E-228	Plasma cells	KDM5D
5.464E-233	1.68803876	0.444	0.053	1.833E-228	Plasma cells	XPNPEP1
5.681E-232	1.56063689	0.365	0.037	1.906E-227	Plasma cells	ACAT1
6.578E-232	2.36755342	0.935	0.259	2.207E-227	Plasma cells	TMEM258
3.408E-231	1.68388239	0.52	0.07	1.143E-226	Plasma cells	PAIP1
3.334E-230	2.06876517	0.85	0.184	1.118E-225	Plasma cells	HLA-DRA
4.232E-230	1.818772	0.659	0.11	1.42E-225	Plasma cells	ABCE1
2.508E-229	1.42063654	0.262	0.02	8.411E-225	Plasma cells	YIF1A
2.889E-229	1.78676693	0.436	0.052	9.689E-225	Plasma cells	TMEM268
1.599E-228	1.74120155	0.624	0.099	5.364E-224	Plasma cells	REPIN1
2.479E-228	1.98943912	0.422	0.05	8.314E-224	Plasma cells	TMEM109
1.089E-227	1.56747292	0.406	0.045	3.652E-223	Plasma cells	BUD23
1.448E-226	2.05869058	0.3	0.026	4.856E-222	Plasma cells	KMO
1.217E-225	1.67143269	0.635	0.103	4.081E-221	Plasma cells	ROMO1
1.599E-225	1.80207811	0.485	0.064	5.365E-221	Plasma cells	NHP2
1.216E-224	1.98806377	0.82	0.178	4.078E-220	Plasma cells	MAN1A1
3.844E-224	2.0305094	0.283	0.024	1.289E-219	Plasma cells	LHFPL2
4.517E-224	1.99526166	0.458	0.058	1.515E-219	Plasma cells	TARBP1
7.269E-224	1.87352273	0.567	0.086	2.438E-219	Plasma cells	SINHCAF
1.808E-223	1.49526424	0.608	0.094	6.064E-219	Plasma cells	NDUFV2
8.999E-223	1.63277433	0.379	0.041	3.019E-218	Plasma cells	RIOX2
1.328E-222	3.27507747	0.807	0.198	4.454E-218	Plasma cells	PLAC8
1.4E-222	1.87221215	0.469	0.061	4.696E-218	Plasma cells	P2RY14
3.871E-221	1.3259656	0.485	0.063	1.299E-216	Plasma cells	GFPT1
4.05E-221	1.68014321	0.605	0.096	1.359E-216	Plasma cells	MRPL51
5.126E-221	2.1314017	0.722	0.141	1.719E-216	Plasma cells	HLA-DPB1
1.146E-220	1.82902924	0.67	0.117	3.844E-216	Plasma cells	HSPE1
2.709E-220	1.64394164	0.602	0.095	9.086E-216	Plasma cells	IMPAD1
3.339E-220	1.74061274	0.662	0.114	1.12E-215	Plasma cells	DNAJC10

7.844E-220	1.44333338	0.436	0.053	2.631E-215	Plasma cells	CISD2
1.031E-219	1.76149692	0.493	0.067	3.459E-215	Plasma cells	IVD
2.67E-219	1.37825238	0.406	0.046	8.955E-215	Plasma cells	TMED3
3.781E-219	1.53093145	0.499	0.067	1.268E-214	Plasma cells	LARP1B
7.028E-219	1.671583	0.608	0.098	2.358E-214	Plasma cells	IBTK
7.14E-219	1.73865407	0.896	0.209	2.395E-214	Plasma cells	CD99
8.43E-219	2.00852011	0.485	0.066	2.828E-214	Plasma cells	ZBTB33
9.13E-219	2.01220469	0.292	0.026	3.062E-214	Plasma cells	SOGA1
1.484E-217	1.76706802	0.414	0.049	4.978E-213	Plasma cells	G6PC3
2.875E-217	2.3693207	0.875	0.209	9.645E-213	Plasma cells	VAMP8
6.854E-217	2.17612777	0.967	0.336	2.299E-212	Plasma cells	CANX
8.529E-217	1.30867695	0.256	0.02	2.861E-212	Plasma cells	TOR3A
1.107E-215	2.304164	0.967	0.306	3.714E-211	Plasma cells	UCP2
1.801E-215	1.59019299	0.49	0.067	6.042E-211	Plasma cells	MRPS14
5.763E-215	2.26084803	0.965	0.32	1.933E-210	Plasma cells	MTDH
1.425E-213	1.68825915	0.436	0.055	4.779E-209	Plasma cells	CHAMP1
2.772E-213	1.81939551	0.82	0.178	9.299E-209	Plasma cells	SLC25A5
1.965E-212	1.34903567	0.668	0.115	6.592E-208	Plasma cells	BTK
3.648E-211	1.75663008	0.866	0.2	1.224E-206	Plasma cells	SEC31A
3.669E-211	1.7555307	0.698	0.132	1.231E-206	Plasma cells	ATP2A2
2.436E-209	1.84009388	0.649	0.116	8.17E-205	Plasma cells	CNDP2
3.405E-209	1.25644521	0.564	0.086	1.142E-204	Plasma cells	ALCAM
9.262E-209	1.37278422	0.46	0.061	3.107E-204	Plasma cells	IQCB1
1.104E-208	2.11497783	0.992	0.357	3.704E-204	Plasma cells	PPIA
1.497E-208	1.94823419	0.932	0.262	5.02E-204	Plasma cells	PDIA3
4.146E-208	1.68072596	0.52	0.077	1.391E-203	Plasma cells	CHCHD10
1.813E-207	1.73430018	0.638	0.113	6.081E-203	Plasma cells	NDUFAB1
1.824E-207	1.7631741	0.678	0.126	6.119E-203	Plasma cells	CCT2
1.889E-207	1.66687579	0.692	0.129	6.338E-203	Plasma cells	CCT8
2.166E-207	1.49415047	0.501	0.071	7.266E-203	Plasma cells	MAP4K1
1.025E-206	1.40132184	0.531	0.079	3.438E-202	Plasma cells	CDK6
1.107E-206	1.52182973	0.627	0.106	3.713E-202	Plasma cells	CDC16
3.232E-205	1.57509457	0.512	0.075	1.084E-200	Plasma cells	MRPS33
3.834E-205	1.57307773	0.828	0.181	1.286E-200	Plasma cells	RBBP4
4.654E-205	1.80227293	0.809	0.177	1.561E-200	Plasma cells	PARK7
7.419E-205	1.81771339	0.929	0.243	2.488E-200	Plasma cells	COX6C
7.462E-205	1.39859451	0.466	0.063	2.503E-200	Plasma cells	RAD17
8.623E-205	1.53664697	0.406	0.05	2.892E-200	Plasma cells	CCNC
1.276E-204	1.68554293	0.681	0.127	4.281E-200	Plasma cells	SNX5
1.375E-204	2.54989866	0.548	0.093	4.611E-200	Plasma cells	LAIR1
1.703E-204	1.77785844	0.621	0.108	5.712E-200	Plasma cells	HMGA1
7.687E-204	1.9652804	0.343	0.037	2.578E-199	Plasma cells	HS3ST3B1
9.276E-204	1.46393132	0.61	0.102	3.111E-199	Plasma cells	ERGIC3
2.774E-203	2.1437071	0.311	0.031	9.304E-199	Plasma cells	ATP13A2
6.951E-203	2.76177488	1	0.506	2.332E-198	Plasma cells	CD74
1.459E-202	1.66978891	0.504	0.074	4.892E-198	Plasma cells	MIF
1.897E-202	1.43319574	0.61	0.103	6.364E-198	Plasma cells	GALNT2
2.571E-202	1.63811286	0.54	0.083	8.624E-198	Plasma cells	NDUFA7
3.074E-202	1.60732332	0.556	0.088	1.031E-197	Plasma cells	TOMM5
3.779E-202	1.87639704	0.951	0.278	1.268E-197	Plasma cells	HINT1

4.751E-202	1.75859173	0.493	0.07	1.594E-197	Plasma cells	SNRPN
6.895E-202	1.42323332	0.362	0.04	2.313E-197	Plasma cells	MARCH9
8.381E-202	1.78861237	0.777	0.168	2.811E-197	Plasma cells	EIF3I
8.726E-202	1.3585655	0.327	0.034	2.927E-197	Plasma cells	IFI27L2
3.13E-201	1.58929684	0.708	0.137	1.05E-196	Plasma cells	PSMA3
3.43E-201	1.5045611	0.346	0.037	1.151E-196	Plasma cells	SEPHS1
5.157E-201	2.63786717	0.507	0.081	1.73E-196	Plasma cells	ELL2
6.062E-201	1.88064792	0.763	0.162	2.033E-196	Plasma cells	YWHAG
1.199E-199	1.46689338	0.343	0.037	4.023E-195	Plasma cells	ACSS1
1.745E-199	1.6918717	0.82	0.187	5.853E-195	Plasma cells	TMED2
2.022E-199	1.40292787	0.264	0.023	6.781E-195	Plasma cells	HINT2
2.118E-199	1.40408692	0.441	0.059	7.105E-195	Plasma cells	FUT8
3.784E-199	1.96423675	0.913	0.244	1.269E-194	Plasma cells	RAN
4.355E-199	2.92576213	0.984	0.388	1.461E-194	Plasma cells	SUB1
4.465E-199	1.22144143	0.319	0.032	1.498E-194	Plasma cells	MCEE
5.012E-199	1.54388133	0.499	0.073	1.681E-194	Plasma cells	LARP4
5.16E-199	1.64385974	0.515	0.078	1.731E-194	Plasma cells	SLC25A39
1.79E-198	1.51917743	0.523	0.079	6.005E-194	Plasma cells	MRPS18B
3.219E-198	2.14349975	0.499	0.078	1.08E-193	Plasma cells	SLC44A1
3.611E-198	1.87560582	0.708	0.141	1.211E-193	Plasma cells	APEX1
3.495E-197	1.59052562	0.632	0.112	1.172E-192	Plasma cells	NDUFB11
3.821E-196	1.52016463	0.335	0.036	1.282E-191	Plasma cells	GOLIM4
5.536E-196	1.68981347	0.425	0.056	1.857E-191	Plasma cells	IDH2
5.632E-196	1.52882877	0.526	0.081	1.889E-191	Plasma cells	LSM5
1.402E-195	1.48554631	0.335	0.036	4.703E-191	Plasma cells	P2RX5
2.19E-195	2.82442276	0.608	0.12	7.346E-191	Plasma cells	CCDC88A
7.125E-195	1.60997662	0.815	0.178	2.39E-190	Plasma cells	ANXA2
4.695E-194	1.13640999	0.283	0.026	1.575E-189	Plasma cells	SNX25
8.15E-194	1.45113087	0.512	0.078	2.734E-189	Plasma cells	GCN1
1.34E-193	1.3834057	0.281	0.026	4.495E-189	Plasma cells	LZTFL1
1.638E-193	1.56459477	0.349	0.039	5.495E-189	Plasma cells	UFSP2
1.956E-193	1.65527633	0.823	0.188	6.56E-189	Plasma cells	NDUFB8
1.533E-192	1.46434607	0.305	0.031	5.142E-188	Plasma cells	TIMM13
1.559E-192	1.72468865	0.627	0.114	5.228E-188	Plasma cells	COMT
2.035E-192	1.63509464	0.719	0.145	6.825E-188	Plasma cells	SMDT1
2.125E-192	1.55872683	0.327	0.035	7.128E-188	Plasma cells	DTWD1
7.104E-192	1.57055101	0.572	0.097	2.383E-187	Plasma cells	UFL1
7.54E-192	1.47374172	0.649	0.118	2.529E-187	Plasma cells	EDF1
1.703E-191	1.56635642	0.771	0.165	5.714E-187	Plasma cells	NDUFB4
4.288E-191	1.07752675	0.515	0.078	1.438E-186	Plasma cells	KLHL6
6.738E-191	2.04553279	0.335	0.038	2.26E-186	Plasma cells	RPS6KA4
7.675E-191	2.75903375	0.46	0.072	2.574E-186	Plasma cells	TGFBI
8.65E-191	2.00773151	0.774	0.185	2.902E-186	Plasma cells	UQCRH
1.651E-190	2.13040626	0.264	0.025	5.537E-186	Plasma cells	MYCL
4.456E-190	1.38013401	0.52	0.08	1.495E-185	Plasma cells	USP11
7.845E-190	1.78863025	0.564	0.098	2.632E-185	Plasma cells	MRPL52
1.689E-189	1.4411049	0.49	0.073	5.667E-185	Plasma cells	PRRC1
2.363E-189	1.49748608	0.447	0.062	7.927E-185	Plasma cells	ACADM
2.417E-189	1.51988461	0.305	0.031	8.106E-185	Plasma cells	MRPL36
3.772E-189	1.4794427	0.569	0.095	1.265E-184	Plasma cells	MRPL57

6.713E-189	2.63665824	0.504	0.083	2.252E-184	Plasma cells	AQP3
8.022E-189	1.44655472	0.444	0.062	2.691E-184	Plasma cells	RHBDD1
9.058E-189	1.06875896	0.414	0.054	3.038E-184	Plasma cells	NTAN1
1.239E-188	1.62428977	0.643	0.12	4.157E-184	Plasma cells	GRSF1
2.16E-188	1.4351866	0.646	0.119	7.246E-184	Plasma cells	MRPL34
6.38E-188	1.63299393	0.33	0.037	2.14E-183	Plasma cells	ARHGAP31
3.795E-187	1.43692885	0.698	0.14	1.273E-182	Plasma cells	MAGT1
3.93E-187	1.45904973	0.42	0.056	1.318E-182	Plasma cells	SEC24A
4.165E-187	1.31058468	0.706	0.138	1.397E-182	Plasma cells	MORF4L2
8.63E-187	1.87919713	0.946	0.304	2.895E-182	Plasma cells	SPCS3
1.474E-186	1.46695908	0.646	0.121	4.944E-182	Plasma cells	SEM1
2.022E-186	1.41070025	0.48	0.072	6.782E-182	Plasma cells	HERC2
4.44E-186	1.62816429	0.793	0.18	1.489E-181	Plasma cells	UFM1
8.884E-186	1.50738245	0.474	0.07	2.98E-181	Plasma cells	EI24
3.239E-185	1.56061528	0.283	0.028	1.086E-180	Plasma cells	FLNB
6.492E-185	1.32930374	0.45	0.063	2.178E-180	Plasma cells	RNASEH1
1.963E-184	1.70355829	0.409	0.055	6.584E-180	Plasma cells	KIF20B
1.047E-183	1.34707143	0.411	0.055	3.512E-179	Plasma cells	NDUFA9
1.18E-183	1.44616444	0.45	0.064	3.958E-179	Plasma cells	IARS
1.588E-183	1.46097542	0.431	0.06	5.328E-179	Plasma cells	SRP68
8.292E-183	1.52310847	0.575	0.101	2.781E-178	Plasma cells	ADI1
1.586E-182	1.41335225	0.545	0.091	5.32E-178	Plasma cells	TXNL4A
5.975E-182	2.29418515	0.722	0.172	2.004E-177	Plasma cells	PABPC4
7.277E-182	1.44442613	0.613	0.111	2.441E-177	Plasma cells	ATRAID
7.337E-182	1.86402072	0.817	0.202	2.461E-177	Plasma cells	RNASE6
1.446E-181	1.4867753	0.534	0.088	4.851E-177	Plasma cells	SNRPC
1.759E-181	1.65318158	0.85	0.211	5.901E-177	Plasma cells	SNRPD2
2.452E-181	1.49669037	0.256	0.024	8.224E-177	Plasma cells	HADH
2.655E-181	1.33785777	0.501	0.078	8.905E-177	Plasma cells	MAP2K6
2.783E-181	1.55291557	0.632	0.119	9.336E-177	Plasma cells	HNRNPAB
3.334E-181	1.54450137	0.67	0.133	1.118E-176	Plasma cells	NDUFC2
4.13E-181	1.60230263	0.583	0.105	1.385E-176	Plasma cells	RCC2
4.515E-181	1.40575022	0.482	0.073	1.515E-176	Plasma cells	NRAS
5.732E-181	1.54436509	0.638	0.121	1.923E-176	Plasma cells	CBX6
8.861E-181	1.42491355	0.608	0.111	2.972E-176	Plasma cells	DSTN
9.847E-181	2.01504418	0.698	0.156	3.303E-176	Plasma cells	APOBEC3C
1.516E-180	1.57583183	0.51	0.081	5.085E-176	Plasma cells	ZNF22
1.718E-180	1.82948494	0.619	0.116	5.762E-176	Plasma cells	LGALS1
2.286E-180	1.32719587	0.54	0.089	7.666E-176	Plasma cells	SLC39A7
3.359E-180	1.73711674	0.698	0.149	1.127E-175	Plasma cells	EPRS
4.471E-180	1.39864875	0.463	0.069	1.5E-175	Plasma cells	GPAA1
4.581E-180	1.64824229	0.275	0.027	1.536E-175	Plasma cells	SLC7A1
5.121E-180	1.65962553	0.411	0.057	1.718E-175	Plasma cells	AGPAT5
6.393E-180	1.66403288	0.864	0.223	2.145E-175	Plasma cells	NDUFA4
6.99E-180	1.47845815	0.608	0.111	2.345E-175	Plasma cells	SSRP1
1.197E-179	1.66762172	0.583	0.106	4.016E-175	Plasma cells	CUTA
1.766E-179	1.11378991	0.561	0.097	5.924E-175	Plasma cells	DNAJC1
1.919E-179	1.61678275	0.526	0.087	6.436E-175	Plasma cells	UTP3
3.321E-179	1.67377743	0.272	0.027	1.114E-174	Plasma cells	SLC9A7
1.8E-178	1.33300117	0.431	0.061	6.037E-174	Plasma cells	MCCC2

2.275E-178	1.55405541	0.877	0.226	7.632E-174	Plasma cells	TCEA1
6.592E-178	1.61334823	0.529	0.089	2.211E-173	Plasma cells	COX16
7.063E-178	1.38730614	0.49	0.077	2.369E-173	Plasma cells	FBH1
7.338E-178	1.69911863	0.76	0.176	2.461E-173	Plasma cells	BANF1
8.451E-178	2.21609347	0.657	0.143	2.835E-173	Plasma cells	SFT2D2
1.522E-177	1.42785759	0.578	0.101	5.104E-173	Plasma cells	ARPC5L
3.414E-177	1.53447848	0.692	0.144	1.145E-172	Plasma cells	EIF2A
4.29E-177	1.57054626	0.548	0.095	1.439E-172	Plasma cells	SELENOH
5.355E-177	1.93612436	0.433	0.064	1.796E-172	Plasma cells	SNX9
1.266E-176	1.49738487	0.575	0.103	4.248E-172	Plasma cells	RBIS
2.366E-176	1.41116009	0.785	0.178	7.935E-172	Plasma cells	EIF5B
3.076E-176	1.4171187	0.474	0.073	1.032E-171	Plasma cells	AK3
1.232E-175	1.69079856	0.959	0.326	4.131E-171	Plasma cells	OST4
3.143E-175	1.51263829	0.725	0.157	1.054E-170	Plasma cells	RPL7L1
3.553E-175	3.69804142	0.542	0.114	1.192E-170	Plasma cells	APP
3.743E-175	1.29790536	0.722	0.15	1.256E-170	Plasma cells	ATP5F1C
9.469E-175	1.45681709	0.73	0.158	3.176E-170	Plasma cells	ERH
1.323E-174	1.51609101	0.82	0.196	4.438E-170	Plasma cells	EID1
1.18E-173	1.48270834	0.392	0.052	3.959E-169	Plasma cells	MRPL9
1.761E-173	1.47643005	0.58	0.106	5.908E-169	Plasma cells	PDHB
2.413E-173	1.81144994	0.425	0.062	8.095E-169	Plasma cells	PHACTR1
4.909E-173	1.91021798	0.526	0.093	1.647E-168	Plasma cells	PRDX1
6.22E-173	1.21771228	0.589	0.106	2.086E-168	Plasma cells	EMC7
9.728E-173	1.65265865	0.569	0.104	3.263E-168	Plasma cells	RNF5
1.395E-172	1.45910272	0.695	0.146	4.678E-168	Plasma cells	NOL7
1.67E-172	1.1498171	0.256	0.024	5.601E-168	Plasma cells	MEI1
3.578E-172	1.51254718	0.466	0.072	1.2E-167	Plasma cells	SLC39A6
5.065E-172	1.51445066	0.738	0.165	1.699E-167	Plasma cells	PSMB2
1.313E-171	1.51064701	0.381	0.051	4.404E-167	Plasma cells	CKAP2
1.942E-171	1.38083398	0.706	0.149	6.515E-167	Plasma cells	NOP58
2.735E-171	1.42543066	0.264	0.026	9.173E-167	Plasma cells	MCM6
3.464E-171	1.58497196	0.308	0.035	1.162E-166	Plasma cells	QDPR
4.202E-171	1.35589452	0.665	0.134	1.409E-166	Plasma cells	DDB1
7.699E-171	1.46383267	0.264	0.026	2.582E-166	Plasma cells	SLC25A23
1.499E-170	1.4765642	0.398	0.055	5.029E-166	Plasma cells	TCEAL8
1.687E-170	1.56647655	0.578	0.107	5.659E-166	Plasma cells	GPR183
2.11E-170	1.34014491	0.493	0.08	7.077E-166	Plasma cells	DNAJC15
2.82E-170	1.41208363	0.545	0.095	9.461E-166	Plasma cells	DDX1
4.013E-170	1.62841037	0.782	0.189	1.346E-165	Plasma cells	SNRPG
1.064E-169	1.29436931	0.283	0.03	3.57E-165	Plasma cells	FAM98A
1.296E-169	1.3032682	0.507	0.083	4.348E-165	Plasma cells	PGRMC2
3.614E-169	1.4610229	0.826	0.202	1.212E-164	Plasma cells	GTF3A
3.95E-169	1.48994806	0.692	0.149	1.325E-164	Plasma cells	SNU13
4.786E-169	1.2569647	0.425	0.061	1.605E-164	Plasma cells	CCDC117
5.873E-169	1.5720765	0.902	0.244	1.97E-164	Plasma cells	SLC25A6
6.32E-169	1.47970663	0.332	0.041	2.12E-164	Plasma cells	TMEM14C
8.22E-169	1.38513586	0.54	0.094	2.757E-164	Plasma cells	SF3B5
4.016E-168	1.40783972	0.559	0.101	1.347E-163	Plasma cells	UFC1
8.532E-168	1.44465582	0.899	0.234	2.862E-163	Plasma cells	SLC38A1
1.371E-167	1.55671621	0.474	0.076	4.599E-163	Plasma cells	RANBP1

2.414E-167	1.83070436	0.259	0.026	8.098E-163	Plasma cells	CPED1
3.484E-167	1.0712834	0.436	0.065	1.169E-162	Plasma cells	VKORC1L1
4.75E-167	1.28207759	0.597	0.114	1.593E-162	Plasma cells	NFX1
5.604E-167	1.36997849	0.733	0.162	1.88E-162	Plasma cells	PSMA6
7.196E-167	1.45252	0.316	0.037	2.414E-162	Plasma cells	PDCL3
7.516E-167	1.38128132	0.676	0.142	2.521E-162	Plasma cells	METAP2
2.488E-166	1.30889866	0.55	0.098	8.346E-162	Plasma cells	PSMC2
2.598E-166	1.28264774	0.373	0.049	8.716E-162	Plasma cells	NOC3L
4.022E-166	1.25432749	0.567	0.102	1.349E-161	Plasma cells	ACP1
8.826E-166	1.33517732	0.708	0.154	2.96E-161	Plasma cells	DNAJC7
1.88E-165	1.49041478	0.689	0.148	6.306E-161	Plasma cells	SNRPE
1.889E-165	1.35383902	0.496	0.083	6.336E-161	Plasma cells	SUMF2
1.913E-165	1.25832189	0.488	0.081	6.415E-161	Plasma cells	HDAC9
2.749E-165	1.33689551	0.327	0.04	9.222E-161	Plasma cells	RBBP8
3.661E-165	1.39980137	0.262	0.026	1.228E-160	Plasma cells	GEN1
5.982E-165	1.39834641	0.493	0.082	2.007E-160	Plasma cells	DCTD
6.023E-165	1.58236302	0.267	0.028	2.02E-160	Plasma cells	ZNF589
6.896E-165	1.26003263	0.575	0.107	2.313E-160	Plasma cells	RNASEH2B
9.284E-165	1.43472965	0.351	0.045	3.114E-160	Plasma cells	NAXE
1.209E-164	1.61071815	0.594	0.117	4.054E-160	Plasma cells	APOBEC3G
1.511E-164	1.4486007	0.52	0.091	5.068E-160	Plasma cells	LAP3
1.893E-164	1.49726455	0.916	0.258	6.351E-160	Plasma cells	SNHG29
8.987E-164	1.94229257	0.387	0.055	3.014E-159	Plasma cells	CD27
1.415E-163	1.07902505	0.381	0.052	4.745E-159	Plasma cells	PPARA
1.415E-163	1.30119039	0.575	0.106	4.746E-159	Plasma cells	KRT10
1.729E-163	1.40900826	0.433	0.066	5.799E-159	Plasma cells	C16orf58
3.842E-163	1.98360274	0.46	0.077	1.289E-158	Plasma cells	NPC1
5.054E-163	1.28452726	0.561	0.103	1.695E-158	Plasma cells	ZCRB1
1.308E-162	1.41477637	0.654	0.136	4.388E-158	Plasma cells	MDH1
1.485E-162	1.544573	0.992	0.347	4.982E-158	Plasma cells	EEF1B2
1.804E-162	1.21476928	0.79	0.186	6.051E-158	Plasma cells	MAPK1IP1L
4.227E-162	1.58473745	0.913	0.268	1.418E-157	Plasma cells	SNHG5
4.676E-162	1.15072443	0.452	0.072	1.569E-157	Plasma cells	TTC7A
5.113E-162	1.48425983	0.422	0.064	1.715E-157	Plasma cells	LTV1
5.773E-162	1.16402117	0.406	0.058	1.936E-157	Plasma cells	CEP97
6.798E-162	1.17264814	0.605	0.116	2.28E-157	Plasma cells	SEC13
1.311E-161	1.36807385	0.33	0.041	4.398E-157	Plasma cells	ZFYVE26
1.937E-161	1.49021938	0.253	0.025	6.498E-157	Plasma cells	NPIPA1
2.581E-161	1.48241619	0.975	0.312	8.659E-157	Plasma cells	RPL35
7.325E-161	1.61504601	0.94	0.316	2.457E-156	Plasma cells	SRPRA
9.93E-161	1.64108985	0.965	0.323	3.331E-156	Plasma cells	EEF2
1.216E-160	1.58655444	0.373	0.051	4.08E-156	Plasma cells	SIT1
1.679E-160	1.36358041	0.548	0.1	5.632E-156	Plasma cells	QARS
2.248E-160	1.39745364	0.706	0.157	7.541E-156	Plasma cells	TUFM
2.683E-160	1.54212003	0.477	0.081	9E-156	Plasma cells	TXNDC15
6.665E-160	1.33308295	0.567	0.106	2.236E-155	Plasma cells	ADH5
6.779E-160	1.70031048	1	0.463	2.274E-155	Plasma cells	RPS8
7.305E-160	1.23228169	0.428	0.065	2.45E-155	Plasma cells	METTL5
8.099E-160	1.67472439	0.921	0.303	2.717E-155	Plasma cells	SSR1
2.144E-159	1.23236856	0.545	0.098	7.191E-155	Plasma cells	CFAP97

2.34E-159	1.48393006	0.64	0.133	7.848E-155	Plasma cells	NOP56
2.554E-159	1.33019271	0.422	0.065	8.568E-155	Plasma cells	AFF3
2.747E-159	1.45462344	0.515	0.091	9.214E-155	Plasma cells	VDAC1
2.755E-159	1.41509438	0.668	0.144	9.242E-155	Plasma cells	SNRPB2
6.453E-159	1.5181492	0.973	0.305	2.165E-154	Plasma cells	HSP90AB1
6.92E-159	1.25828503	0.406	0.06	2.321E-154	Plasma cells	POLR2K
1.44E-158	1.56061732	0.575	0.113	4.831E-154	Plasma cells	RPS27L
1.573E-158	1.37463843	0.602	0.12	5.276E-154	Plasma cells	TTC37
1.854E-158	1.47827706	0.883	0.242	6.22E-154	Plasma cells	ATP5F1B
1.925E-158	2.07857611	0.926	0.366	6.457E-154	Plasma cells	HSPA5
2.666E-158	1.32603889	0.294	0.034	8.943E-154	Plasma cells	CYP51A1
4.579E-158	1.57402578	0.616	0.129	1.536E-153	Plasma cells	NXPE3
6.452E-158	1.33624095	0.597	0.117	2.164E-153	Plasma cells	RBBP7
2.75E-157	1.53582023	0.995	0.348	9.223E-153	Plasma cells	RPL7A
3.196E-157	1.42653119	0.58	0.114	1.072E-152	Plasma cells	USP14
3.373E-157	1.32363568	0.52	0.094	1.131E-152	Plasma cells	RAP1GDS1
4.203E-157	1.13677667	0.332	0.042	1.41E-152	Plasma cells	UCHL3
1.046E-156	1.45581941	0.575	0.112	3.509E-152	Plasma cells	TMX3
1.315E-156	1.39387437	0.463	0.077	4.411E-152	Plasma cells	MRPL3
1.822E-156	1.08545006	0.534	0.096	6.111E-152	Plasma cells	SEC23B
1.859E-156	1.38322748	0.455	0.075	6.235E-152	Plasma cells	CNPY2
1.886E-156	1.40753028	0.978	0.311	6.327E-152	Plasma cells	RPSA
2.135E-156	1.39827108	0.755	0.18	7.163E-152	Plasma cells	COX7A2L
8.718E-156	1.24886958	0.439	0.07	2.924E-151	Plasma cells	TIMM17A
2.152E-155	1.22300537	0.297	0.035	7.219E-151	Plasma cells	FARP2
2.304E-155	1.01936183	0.384	0.054	7.728E-151	Plasma cells	GEMIN7
2.409E-155	1.29277474	0.349	0.047	8.08E-151	Plasma cells	CARM1
3.172E-155	1.35325119	0.425	0.067	1.064E-150	Plasma cells	RUFY3
6.338E-155	1.62472228	0.951	0.322	2.126E-150	Plasma cells	RBM3
6.537E-155	0.43270653	0.392	0.056	2.193E-150	Plasma cells	BANK1
1.038E-154	1.94962742	0.499	0.092	3.482E-150	Plasma cells	DCK
1.42E-154	1.35636839	0.471	0.08	4.764E-150	Plasma cells	PSMB6
2.24E-154	1.31610277	0.294	0.035	7.515E-150	Plasma cells	SLC12A2
2.956E-154	1.75548905	0.313	0.039	9.916E-150	Plasma cells	LAX1
3.244E-154	1.2856537	0.629	0.132	1.088E-149	Plasma cells	MFF
5.375E-154	1.16351545	0.406	0.061	1.803E-149	Plasma cells	GOSR2
7.354E-154	1.27267204	0.45	0.073	2.467E-149	Plasma cells	EIF2S1
1.724E-153	1.55338199	0.428	0.068	5.782E-149	Plasma cells	HLA-DQB1
1.939E-153	1.49078918	0.986	0.355	6.505E-149	Plasma cells	RPS5
2.082E-153	1.28358112	0.52	0.094	6.984E-149	Plasma cells	DERL1
2.187E-153	1.07655901	0.373	0.053	7.336E-149	Plasma cells	ATG4C
3.47E-153	1.62673609	0.542	0.105	1.164E-148	Plasma cells	CAPG
5.194E-153	1.31920412	0.526	0.096	1.742E-148	Plasma cells	LY9
6.433E-153	1.58752088	0.256	0.027	2.158E-148	Plasma cells	ZBTB10
7.367E-153	1.48535648	0.632	0.136	2.471E-148	Plasma cells	SUMO3
1.055E-152	1.51515338	0.997	0.373	3.538E-148	Plasma cells	RPS4X
1.063E-152	1.27030977	0.379	0.054	3.564E-148	Plasma cells	MTRR
1.41E-152	1.40386933	0.403	0.062	4.73E-148	Plasma cells	NUP42
1.565E-152	1.58970827	0.717	0.174	5.25E-148	Plasma cells	UQCRQ
1.924E-152	1.13995154	0.354	0.049	6.453E-148	Plasma cells	WDR41

2.375E-152	1.33820922	0.537	0.101	7.967E-148	Plasma cells	CASP3
6.853E-152	1.25365787	0.757	0.181	2.299E-147	Plasma cells	TMCO1
9.524E-152	1.40250224	0.995	0.334	3.194E-147	Plasma cells	RPL18A
1.184E-151	1.42349214	0.809	0.214	3.971E-147	Plasma cells	TTC3
1.289E-151	1.38027406	0.817	0.225	4.324E-147	Plasma cells	CLINT1
1.685E-151	1.56938378	0.54	0.104	5.652E-147	Plasma cells	MDN1
2.25E-151	1.37280167	0.39	0.058	7.548E-147	Plasma cells	AGA
3.91E-151	1.2212673	0.548	0.103	1.312E-146	Plasma cells	FKBP3
5.723E-151	1.40061479	0.621	0.133	1.92E-146	Plasma cells	SEC24D
6.694E-151	1.38079311	0.477	0.083	2.245E-146	Plasma cells	GARS
1.176E-150	1.52247016	1	0.533	3.944E-146	Plasma cells	RPS11
1.268E-150	1.25445996	0.809	0.209	4.254E-146	Plasma cells	SPN
1.402E-150	0.87373057	0.305	0.037	4.703E-146	Plasma cells	EEF2K
1.738E-150	1.57981857	0.556	0.113	5.831E-146	Plasma cells	CERS6
2.565E-150	1.16370121	0.406	0.062	8.604E-146	Plasma cells	FAM126A
1.035E-149	1.40173577	0.798	0.21	3.473E-145	Plasma cells	ARF4
1.272E-149	1.37773534	0.253	0.027	4.265E-145	Plasma cells	NUDT1
1.445E-149	1.50268894	0.561	0.113	4.847E-145	Plasma cells	SPINT2
1.847E-149	1.57754794	0.256	0.028	6.196E-145	Plasma cells	ADPRH
2.835E-149	1.44347579	0.975	0.333	9.509E-145	Plasma cells	NPM1
2.871E-149	1.48408242	0.995	0.361	9.629E-145	Plasma cells	RACK1
2.967E-149	1.73257511	0.379	0.057	9.954E-145	Plasma cells	SLC2A6
3.244E-149	1.02518277	0.332	0.044	1.088E-144	Plasma cells	NANS
3.656E-149	1.33756505	0.45	0.075	1.226E-144	Plasma cells	EBAG9
5.789E-149	1.28723417	0.785	0.195	1.942E-144	Plasma cells	SSB
7.686E-149	1.32306635	0.768	0.196	2.578E-144	Plasma cells	SEC63
9.305E-149	1.26659941	0.346	0.048	3.121E-144	Plasma cells	IDE
1.537E-148	1.46948756	0.632	0.139	5.155E-144	Plasma cells	APPL1
1.651E-148	1.08258921	0.39	0.059	5.538E-144	Plasma cells	TRIO
1.82E-148	1.49938491	0.872	0.258	6.103E-144	Plasma cells	EIF2S2
2.042E-148	1.2459677	0.629	0.134	6.849E-144	Plasma cells	ACO2
3.774E-148	1.10496873	0.485	0.085	1.266E-143	Plasma cells	RFTN1
3.779E-148	1.34391023	0.281	0.033	1.267E-143	Plasma cells	STAMBPL1
4.729E-148	1.40289098	0.259	0.028	1.586E-143	Plasma cells	MAGEH1
5.75E-148	1.34429237	0.297	0.037	1.929E-143	Plasma cells	COX18
8.584E-148	1.08322272	0.668	0.144	2.879E-143	Plasma cells	TMEM230
9.889E-148	1.23837444	0.474	0.082	3.317E-143	Plasma cells	PWP1
1.237E-147	1.31934938	0.779	0.198	4.148E-143	Plasma cells	LMO4
1.691E-147	1.2424487	0.281	0.033	5.673E-143	Plasma cells	HMGXB3
2.911E-147	1.49261154	0.959	0.319	9.763E-143	Plasma cells	NCL
3.715E-147	1.42451737	0.319	0.042	1.246E-142	Plasma cells	EBNA1BP2
4.227E-147	1.31167156	0.289	0.035	1.418E-142	Plasma cells	AC073111.4
9.793E-147	1.13645494	0.605	0.124	3.285E-142	Plasma cells	CIAO2A
1.01E-146	1.3216711	0.387	0.059	3.387E-142	Plasma cells	PSMC3
1.176E-146	1.44851348	0.992	0.368	3.945E-142	Plasma cells	RPS19
5.251E-146	1.11263849	0.319	0.041	1.761E-141	Plasma cells	MRPL2
8.625E-146	1.21617073	0.33	0.044	2.893E-141	Plasma cells	GLT8D1
8.774E-146	1.32153645	0.744	0.183	2.943E-141	Plasma cells	EIF1AX
9.221E-146	1.24922283	0.864	0.238	3.093E-141	Plasma cells	ZRANB2
2.623E-145	1.22846402	0.534	0.103	8.797E-141	Plasma cells	PSMD8

3.841E-145	1.36293782	0.929	0.281	1.288E-140	Plasma cells	COX7C
3.873E-145	1.46595369	0.997	0.392	1.299E-140	Plasma cells	RPL3
6.29E-145	1.14258845	0.523	0.097	2.11E-140	Plasma cells	RTRAF
8.029E-145	1.24036618	0.488	0.09	2.693E-140	Plasma cells	MBNL2
1.635E-144	1.44964663	0.425	0.071	5.484E-140	Plasma cells	SLC3A2
2.959E-144	1.1604926	0.474	0.084	9.924E-140	Plasma cells	COX17
5.021E-144	1.15814259	0.278	0.033	1.684E-139	Plasma cells	FAM136A
5.429E-144	1.28991868	0.706	0.169	1.821E-139	Plasma cells	RBX1
6.019E-144	1.25972813	0.49	0.089	2.019E-139	Plasma cells	EIF4A3
8.975E-144	1.03521757	0.499	0.091	3.01E-139	Plasma cells	PDXDC1
1.021E-143	0.92851629	0.875	0.229	3.425E-139	Plasma cells	LDHB
1.601E-143	1.25973715	0.649	0.143	5.372E-139	Plasma cells	EIF3M
1.773E-143	1.26831969	0.567	0.115	5.948E-139	Plasma cells	DNAJC21
2.436E-143	1.39506481	0.992	0.361	8.173E-139	Plasma cells	RPL10A
2.45E-143	1.36886169	0.362	0.054	8.217E-139	Plasma cells	MRPL37
3.494E-143	1.05570157	0.662	0.145	1.172E-138	Plasma cells	SURF4
4.999E-143	1.22408767	0.474	0.085	1.677E-138	Plasma cells	SAR1B
6.248E-143	1.53157966	0.523	0.103	2.096E-138	Plasma cells	ITGB7
9.831E-143	1.29009938	0.654	0.148	3.298E-138	Plasma cells	RFC1
1.458E-142	1.43580335	0.97	0.344	4.892E-138	Plasma cells	RPL24
1.528E-142	1.0943653	0.597	0.126	5.125E-138	Plasma cells	OXR1
1.985E-142	1.10828669	0.515	0.096	6.659E-138	Plasma cells	YIPF5
3.656E-142	1.34268229	0.654	0.146	1.226E-137	Plasma cells	GSTP1
6.455E-142	1.22334792	0.559	0.112	2.165E-137	Plasma cells	SYNGR2
8.727E-142	1.32946674	0.58	0.121	2.927E-137	Plasma cells	POLE3
1.137E-141	1.62708923	0.496	0.094	3.815E-137	Plasma cells	IGFLR1
1.353E-141	1.92533511	0.294	0.038	4.539E-137	Plasma cells	CRYBG3
1.668E-141	1.56103932	0.864	0.263	5.594E-137	Plasma cells	ARL6IP1
1.842E-141	1.14071718	0.259	0.029	6.18E-137	Plasma cells	ASPHD2
2.065E-141	0.96737673	0.289	0.035	6.926E-137	Plasma cells	APOBEC3F
2.089E-141	1.28203943	0.654	0.148	7.008E-137	Plasma cells	COX7A2
2.273E-141	1.24581805	0.45	0.077	7.624E-137	Plasma cells	CHCHD7
2.331E-141	1.31874232	0.403	0.065	7.82E-137	Plasma cells	CYC1
3.175E-141	1.4473103	0.501	0.095	1.065E-136	Plasma cells	SNRPD1
3.628E-141	1.6343388	0.657	0.16	1.217E-136	Plasma cells	GSPT1
3.892E-141	1.2739787	0.327	0.045	1.306E-136	Plasma cells	ABI2
4.773E-141	1.24821482	0.67	0.153	1.601E-136	Plasma cells	EIF4G1
1.111E-140	1.34685907	0.297	0.038	3.727E-136	Plasma cells	PAK1IP1
1.159E-140	1.33676033	0.292	0.037	3.889E-136	Plasma cells	PTRHD1
1.257E-140	1.49059772	0.997	0.458	4.215E-136	Plasma cells	RPLP1
1.569E-140	1.23519309	0.439	0.075	5.262E-136	Plasma cells	CDYL
1.588E-140	1.30067109	0.452	0.079	5.325E-136	Plasma cells	DNAJC19
1.913E-140	1.24422033	0.659	0.15	6.418E-136	Plasma cells	RAD23A
2.786E-140	1.33660168	0.809	0.226	9.344E-136	Plasma cells	STT3B
3.12E-140	1.3918178	0.403	0.065	1.047E-135	Plasma cells	PDCD5
3.336E-140	1.19124828	0.278	0.034	1.119E-135	Plasma cells	ARMCX6
7.87E-140	1.29961264	0.441	0.076	2.64E-135	Plasma cells	DDI2
8.103E-140	1.3099791	0.673	0.158	2.718E-135	Plasma cells	NDUFS5
9.622E-140	1.26025049	0.493	0.092	3.228E-135	Plasma cells	FUNDC2
1.298E-139	1.2157673	0.46	0.081	4.352E-135	Plasma cells	RABAC1

1.75E-139	1.21048321	0.678	0.158	5.869E-135	Plasma cells	PPM1G
2.366E-139	1.45443062	0.989	0.418	7.936E-135	Plasma cells	RPS7
2.553E-139	1.5384723	0.763	0.207	8.565E-135	Plasma cells	EIF5A
2.663E-139	1.30204881	0.322	0.044	8.933E-135	Plasma cells	FAM174C
2.941E-139	1.2352484	0.33	0.046	9.864E-135	Plasma cells	ATP13A1
2.947E-139	1.06433299	0.425	0.071	9.886E-135	Plasma cells	MCTS1
4.357E-139	1.29960532	0.807	0.219	1.461E-134	Plasma cells	OIP5-AS1
4.379E-139	1.3792231	0.804	0.218	1.469E-134	Plasma cells	ATP5PF
4.395E-139	1.35233209	0.684	0.162	1.474E-134	Plasma cells	TOMM6
4.433E-139	1.28085157	0.698	0.17	1.487E-134	Plasma cells	USO1
6.326E-139	1.64982777	0.39	0.063	2.122E-134	Plasma cells	HLA-DRB5
6.354E-139	1.23953122	0.812	0.215	2.131E-134	Plasma cells	RBMX
1.44E-138	1.1433207	0.654	0.148	4.831E-134	Plasma cells	GANAB
1.864E-138	1.11290614	0.256	0.029	6.251E-134	Plasma cells	EZH2
1.896E-138	1.27394515	0.728	0.18	6.358E-134	Plasma cells	SSBP1
2.349E-138	1.14264023	0.54	0.108	7.878E-134	Plasma cells	NDUFS6
3.482E-138	1.47589245	0.556	0.117	1.168E-133	Plasma cells	HLA-DMA
5.254E-138	1.26681504	0.499	0.095	1.762E-133	Plasma cells	GOLGA3
1.227E-137	1.19179465	0.597	0.128	4.114E-133	Plasma cells	UPF3A
1.286E-137	1.37504494	0.485	0.091	4.315E-133	Plasma cells	TOMM22
1.457E-137	1.10484861	0.425	0.072	4.887E-133	Plasma cells	YARS
1.601E-137	1.39228573	0.662	0.158	5.37E-133	Plasma cells	PHB2
2.975E-137	1.46328206	0.997	0.453	9.979E-133	Plasma cells	RPL37A
3.133E-137	1.37426111	0.755	0.201	1.051E-132	Plasma cells	EZR
3.718E-137	1.41087577	0.997	0.4	1.247E-132	Plasma cells	RPS23
7.609E-137	1.11301799	0.801	0.212	2.552E-132	Plasma cells	CYBB
8.646E-137	1.32737461	0.455	0.081	2.9E-132	Plasma cells	IPO5
1.483E-136	1.26554038	0.657	0.153	4.974E-132	Plasma cells	VPS36
1.49E-136	1.19037735	0.589	0.125	4.996E-132	Plasma cells	AAMP
1.495E-136	1.18206688	0.433	0.075	5.015E-132	Plasma cells	POLR2L
1.603E-136	1.33345519	0.362	0.055	5.376E-132	Plasma cells	TARS
2.212E-136	1.33199529	0.379	0.06	7.421E-132	Plasma cells	MRPL16
2.234E-136	1.300156	0.831	0.235	7.492E-132	Plasma cells	HMGN1
2.252E-136	1.41852103	0.771	0.215	7.553E-132	Plasma cells	DAD1
2.55E-136	1.09160407	0.349	0.052	8.553E-132	Plasma cells	ARHGAP18
4.148E-136	1.18922016	0.433	0.074	1.391E-131	Plasma cells	TIFA
4.397E-136	1.1173859	0.7	0.17	1.475E-131	Plasma cells	UBE3A
5.143E-136	1.29434584	0.406	0.068	1.725E-131	Plasma cells	TBC1D9B
7.073E-136	1.03446058	0.559	0.114	2.372E-131	Plasma cells	NMT1
8.525E-136	2.54050188	0.736	0.212	2.86E-131	Plasma cells	ITGB1
1.056E-135	1.06780804	0.589	0.125	3.542E-131	Plasma cells	ACADVL
1.262E-135	1.30995107	0.444	0.079	4.233E-131	Plasma cells	TOMM70
1.425E-135	1.07106974	0.341	0.049	4.78E-131	Plasma cells	TSEN15
1.535E-135	1.3093907	0.444	0.079	5.15E-131	Plasma cells	ECH1
1.778E-135	1.09159972	0.482	0.09	5.963E-131	Plasma cells	NNT
2.863E-135	1.10063149	0.796	0.213	9.603E-131	Plasma cells	EIF2AK1
3.189E-135	0.92853712	0.403	0.066	1.07E-130	Plasma cells	SLC30A5
3.865E-135	1.06739865	0.444	0.078	1.297E-130	Plasma cells	SNX4
3.895E-135	1.82034576	0.526	0.112	1.306E-130	Plasma cells	HLA-DMB
5.82E-135	1.43778754	0.997	0.431	1.952E-130	Plasma cells	RPS18

6.282E-135	1.12833704	0.801	0.211	2.107E-130	Plasma cells	TRA2B
6.408E-135	1.31578451	0.85	0.249	2.15E-130	Plasma cells	KTN1
7.347E-135	1.23152844	0.725	0.18	2.464E-130	Plasma cells	TOMM20
7.748E-135	1.23121243	0.33	0.047	2.599E-130	Plasma cells	TSPAN31
7.834E-135	1.14735912	0.281	0.035	2.628E-130	Plasma cells	RUVBL1
1.377E-134	1.42602376	0.861	0.279	4.62E-130	Plasma cells	VOPP1
1.438E-134	1.07824233	0.259	0.031	4.823E-130	Plasma cells	COQ5
1.584E-134	1.12148731	0.308	0.042	5.315E-130	Plasma cells	PRKRA
1.937E-134	1.21460729	0.537	0.108	6.498E-130	Plasma cells	SNHG8
1.982E-134	1.14534158	0.458	0.084	6.648E-130	Plasma cells	NDUFS4
2.292E-134	1.29268902	0.629	0.144	7.688E-130	Plasma cells	ATP5PO
3.039E-134	1.12435355	0.79	0.214	1.019E-129	Plasma cells	HM13
5.122E-134	1.35865714	0.926	0.306	1.718E-129	Plasma cells	YWHAE
6.049E-134	1.29936546	0.289	0.037	2.029E-129	Plasma cells	NUDCD2
9.711E-134	1.36557045	0.992	0.384	3.257E-129	Plasma cells	RPL23A
1.134E-133	1.19848665	0.608	0.133	3.805E-129	Plasma cells	CCT6A
1.517E-133	1.38244479	0.997	0.39	5.09E-129	Plasma cells	RPL15
1.578E-133	1.37130554	1	0.414	5.293E-129	Plasma cells	RPS17
1.585E-133	1.042197	0.488	0.092	5.316E-129	Plasma cells	DYNLRB1
1.612E-133	1.30406166	0.409	0.069	5.408E-129	Plasma cells	BCCIP
2.012E-133	1.2437899	0.401	0.067	6.75E-129	Plasma cells	MRPS34
3.047E-133	1.28673001	0.619	0.143	1.022E-128	Plasma cells	GLCCI1
3.222E-133	1.24409475	0.286	0.037	1.081E-128	Plasma cells	CLCC1
3.515E-133	1.24185601	0.346	0.052	1.179E-128	Plasma cells	SMIM15
3.614E-133	1.17523743	0.48	0.09	1.212E-128	Plasma cells	UCHL5
4.164E-133	1.23118601	0.55	0.116	1.397E-128	Plasma cells	BTAF1
9.782E-133	1.26327725	0.556	0.117	3.281E-128	Plasma cells	ATP1B3
9.833E-133	0.89810064	0.485	0.091	3.298E-128	Plasma cells	LRRK1
1.208E-132	1.15076861	0.441	0.078	4.051E-128	Plasma cells	ATP8B2
2.039E-132	1.42716059	0.569	0.123	6.84E-128	Plasma cells	HSPH1
2.694E-132	0.93578754	0.646	0.147	9.035E-128	Plasma cells	NGLY1
2.924E-132	1.5340404	0.311	0.043	9.806E-128	Plasma cells	AC008124.1
3.945E-132	1.31795933	0.995	0.372	1.323E-127	Plasma cells	RPL22
4.119E-132	1.25616214	0.501	0.099	1.381E-127	Plasma cells	HCFC1
4.915E-132	1.23284476	0.488	0.094	1.648E-127	Plasma cells	SLC25A46
5.862E-132	1.28986637	0.918	0.295	1.966E-127	Plasma cells	UBB
5.958E-132	1.19523994	0.856	0.246	1.998E-127	Plasma cells	TRMT112
1.039E-131	1.15883709	0.695	0.169	3.486E-127	Plasma cells	DDX18
1.288E-131	1.06294371	0.253	0.03	4.322E-127	Plasma cells	NABP2
1.346E-131	1.13452207	0.744	0.192	4.514E-127	Plasma cells	PTPN2
1.66E-131	1.27545869	1	0.36	5.569E-127	Plasma cells	RPL4
1.978E-131	1.06816288	0.523	0.104	6.635E-127	Plasma cells	NDUFB5
3.381E-131	1.45190104	0.54	0.114	1.134E-126	Plasma cells	AL365361.1
4.024E-131	1.23434006	0.381	0.062	1.35E-126	Plasma cells	NDUFS3
4.549E-131	1.05037525	0.722	0.18	1.526E-126	Plasma cells	COPG1
6.708E-131	1.34720234	0.995	0.392	2.25E-126	Plasma cells	RPL5
7.21E-131	1.25300302	0.714	0.181	2.418E-126	Plasma cells	ATP5MD
8.247E-131	1.15169235	0.55	0.115	2.766E-126	Plasma cells	KIAA0930
1.272E-130	1.21780746	0.937	0.299	4.266E-126	Plasma cells	RPL18
1.505E-130	1.05825188	0.482	0.092	5.048E-126	Plasma cells	ARFGEF2

1.525E-130	1.16256255	0.335	0.05	5.114E-126	Plasma cells	NDUFAF3
2.374E-130	1.50014889	0.708	0.185	7.963E-126	Plasma cells	CBFA2T3
2.619E-130	1.14986041	0.332	0.049	8.784E-126	Plasma cells	MRPS7
2.721E-130	1.15720108	0.981	0.321	9.128E-126	Plasma cells	EEF1G
2.74E-130	1.18934072	0.332	0.049	9.191E-126	Plasma cells	DCAF13
3.045E-130	1.22635213	0.926	0.296	1.022E-125	Plasma cells	TLE5
3.439E-130	1.30398977	0.948	0.314	1.153E-125	Plasma cells	HNRNPA1
3.659E-130	1.19322171	0.815	0.226	1.227E-125	Plasma cells	POMP
5.704E-130	1.36281826	0.997	0.416	1.913E-125	Plasma cells	RPL10
7.203E-130	1.33223308	0.433	0.078	2.416E-125	Plasma cells	SNRPF
8.39E-130	1.51276323	0.368	0.06	2.814E-125	Plasma cells	PAFAH2
1.087E-129	1.2294664	0.341	0.051	3.645E-125	Plasma cells	PFDN4
1.386E-129	1.23075999	0.815	0.23	4.648E-125	Plasma cells	EIF3F
2.16E-129	1.09091713	0.553	0.117	7.244E-125	Plasma cells	ACBD3
2.519E-129	1.44463288	0.888	0.291	8.45E-125	Plasma cells	CHCHD2
2.531E-129	1.20379763	0.357	0.056	8.489E-125	Plasma cells	NSMCE4A
3.073E-129	1.19890198	0.482	0.094	1.031E-124	Plasma cells	UQCRFS1
7.39E-129	1.19539932	0.46	0.087	2.479E-124	Plasma cells	MRPS21
8.467E-129	1.24196696	0.286	0.038	2.84E-124	Plasma cells	CETN3
1.105E-128	1.41084766	1	0.48	3.706E-124	Plasma cells	RPS13
1.208E-128	1.08228379	0.401	0.068	4.052E-124	Plasma cells	TMEM243
1.572E-128	1.28184436	0.875	0.267	5.275E-124	Plasma cells	SRSF2
1.601E-128	1.03125171	0.589	0.128	5.372E-124	Plasma cells	TMEM50B
1.94E-128	1.0400741	0.586	0.129	6.508E-124	Plasma cells	MAN2B1
2.307E-128	1.23270282	0.381	0.063	7.738E-124	Plasma cells	BLMH
5.039E-128	1.06015112	0.578	0.126	1.69E-123	Plasma cells	PSMA2
1.512E-127	1.4003002	0.989	0.413	5.073E-123	Plasma cells	RPS2
1.542E-127	1.20871319	0.409	0.071	5.171E-123	Plasma cells	CFAP298
2.168E-127	1.32873451	0.992	0.391	7.273E-123	Plasma cells	RPL36A
2.679E-127	2.03790306	0.556	0.133	8.988E-123	Plasma cells	OFD1
3.077E-127	1.11413943	0.387	0.065	1.032E-122	Plasma cells	MRPS15
3.208E-127	1.40031035	0.992	0.4	1.076E-122	Plasma cells	YBX1
3.745E-127	1.07286808	0.403	0.069	1.256E-122	Plasma cells	UBXN2A
6.721E-127	0.87462505	0.548	0.115	2.254E-122	Plasma cells	CAMTA1
8.347E-127	1.43236964	0.602	0.145	2.8E-122	Plasma cells	RUNX2
1.203E-126	1.26663034	0.346	0.054	4.035E-122	Plasma cells	UTP14A
1.895E-126	1.06043144	0.422	0.075	6.358E-122	Plasma cells	WBP1
2.02E-126	1.17206004	0.262	0.033	6.777E-122	Plasma cells	PMVK
2.32E-126	1.40775538	0.995	0.466	7.782E-122	Plasma cells	RPL6
2.676E-126	1.17865867	0.504	0.102	8.977E-122	Plasma cells	TECR
4.324E-126	1.16263687	0.771	0.21	1.45E-121	Plasma cells	ZC3H15
4.522E-126	1.23913996	0.428	0.078	1.517E-121	Plasma cells	GUSB
4.524E-126	1.26284784	0.256	0.032	1.518E-121	Plasma cells	UBFD1
8.196E-126	0.95180835	0.64	0.153	2.749E-121	Plasma cells	ANKIB1
9.292E-126	1.31958672	0.741	0.203	3.117E-121	Plasma cells	HSPD1
1.356E-125	1.47703402	0.995	0.493	4.549E-121	Plasma cells	RPS15A
1.552E-125	1.2483021	0.466	0.091	5.207E-121	Plasma cells	MRPS31
3.019E-125	0.84677296	0.665	0.16	1.013E-120	Plasma cells	SCFD1
3.204E-125	1.22152719	0.856	0.255	1.075E-120	Plasma cells	ATP5MF
3.371E-125	1.14132635	0.586	0.131	1.131E-120	Plasma cells	IMP3

4.462E-125	1.21840358	0.368	0.06	1.497E-120	Plasma cells	GTF3C6
4.475E-125	1.14720989	0.695	0.178	1.501E-120	Plasma cells	LARP1
5.006E-125	1.23770546	0.635	0.156	1.679E-120	Plasma cells	CHD9
7.245E-125	0.92631109	0.354	0.057	2.43E-120	Plasma cells	UBE2E2
8.399E-125	1.25531484	0.755	0.203	2.817E-120	Plasma cells	PA2G4
9.224E-125	1.46092489	0.578	0.134	3.094E-120	Plasma cells	TMX1
1.056E-124	1.16447337	0.995	0.744	3.541E-120	Plasma cells	GNAS
1.209E-124	1.06889321	0.286	0.039	4.057E-120	Plasma cells	AAAS
1.279E-124	1.34908957	0.997	0.471	4.292E-120	Plasma cells	RPS28
1.641E-124	1.13957941	0.888	0.266	5.504E-120	Plasma cells	EIF3E
1.758E-124	1.22303131	0.807	0.233	5.896E-120	Plasma cells	SYNCRIP
2.174E-124	0.90736914	0.597	0.133	7.291E-120	Plasma cells	NIPA2
2.656E-124	0.89862579	0.711	0.181	8.911E-120	Plasma cells	MAN2A1
2.902E-124	1.40536503	1	0.54	9.733E-120	Plasma cells	RPS24
1.036E-123	1.29081227	0.308	0.045	3.476E-119	Plasma cells	SLC50A1
1.091E-123	1.26007132	0.807	0.24	3.66E-119	Plasma cells	OGT
1.159E-123	1.88739105	0.845	0.284	3.888E-119	Plasma cells	NPC2
1.445E-123	1.09638023	0.545	0.117	4.848E-119	Plasma cells	GTF2A2
1.46E-123	1.20373551	0.272	0.036	4.898E-119	Plasma cells	RMDN3
1.735E-123	1.16030549	0.439	0.082	5.819E-119	Plasma cells	MTCH2
2.601E-123	1.04249892	0.507	0.105	8.726E-119	Plasma cells	HSD17B4
3.274E-123	1.10393406	0.619	0.146	1.098E-118	Plasma cells	AK2
3.521E-123	0.95815832	0.292	0.04	1.181E-118	Plasma cells	PMM2
3.694E-123	1.21699508	0.529	0.115	1.239E-118	Plasma cells	FEZ2
1.442E-122	1.1401979	0.324	0.049	4.838E-118	Plasma cells	PSMD14
3.05E-122	1.8412757	0.463	0.093	1.023E-117	Plasma cells	CRIP1
3.095E-122	1.71851521	0.477	0.1	1.038E-117	Plasma cells	DPYSL2
3.65E-122	0.93267291	0.493	0.101	1.224E-117	Plasma cells	R3HDM1
3.689E-122	1.1112698	0.58	0.133	1.238E-117	Plasma cells	RDH11
5.647E-122	0.98442288	0.417	0.075	1.894E-117	Plasma cells	RMC1
7.408E-122	1.03926534	0.537	0.114	2.485E-117	Plasma cells	OAS2
1.09E-121	1.20829385	0.559	0.126	3.656E-117	Plasma cells	CYB5B
1.243E-121	1.33189457	0.959	0.352	4.169E-117	Plasma cells	RPL8
1.44E-121	1.18816878	0.302	0.044	4.829E-117	Plasma cells	TP53RK
1.492E-121	1.17695982	0.278	0.038	5.003E-117	Plasma cells	HLTF
1.689E-121	1.09715025	0.286	0.04	5.665E-117	Plasma cells	APOOL
2.389E-121	1.16096803	0.76	0.212	8.014E-117	Plasma cells	TMED5
4.005E-121	1.27433431	1	0.467	1.343E-116	Plasma cells	RPS16
5.054E-121	1.02818668	0.507	0.105	1.695E-116	Plasma cells	MTMR1
7.13E-121	1.08202352	0.51	0.106	2.392E-116	Plasma cells	IRAK1
9.091E-121	1.09274061	0.351	0.057	3.049E-116	Plasma cells	RAB2B
9.597E-121	1.20978841	0.253	0.032	3.219E-116	Plasma cells	MRPL15
1.295E-120	1.34876857	0.482	0.099	4.345E-116	Plasma cells	CBX5
1.301E-120	1.1497183	0.583	0.135	4.364E-116	Plasma cells	LRRC58
1.76E-120	1.47404581	0.937	0.38	5.904E-116	Plasma cells	ATP5MG
2.22E-120	1.28801238	1	0.422	7.446E-116	Plasma cells	RPL12
3.431E-120	1.41548181	0.319	0.049	1.151E-115	Plasma cells	SLAMF1
3.545E-120	1.18967468	0.45	0.088	1.189E-115	Plasma cells	SUGT1
3.742E-120	1.19215011	0.678	0.172	1.255E-115	Plasma cells	NDUFB2
4.278E-120	1.08701317	0.338	0.053	1.435E-115	Plasma cells	RPA3

4.719E-120	1.35170401	1	0.518	1.583E-115	Plasma cells	RPL23
5.189E-120	1.06767866	0.796	0.219	1.741E-115	Plasma cells	RSL1D1
6.465E-120	1.12405581	0.365	0.061	2.169E-115	Plasma cells	PCNT
7.036E-120	1.16256753	0.338	0.054	2.36E-115	Plasma cells	CPSF3
7.199E-120	1.27877614	0.997	0.413	2.415E-115	Plasma cells	RPL29
7.744E-120	1.20303898	0.793	0.235	2.598E-115	Plasma cells	SRP19
7.869E-120	1.09723098	0.578	0.133	2.64E-115	Plasma cells	ARL5A
1.024E-119	0.83487268	0.589	0.134	3.435E-115	Plasma cells	CYSLTR1
1.651E-119	0.89648027	0.559	0.124	5.54E-115	Plasma cells	PPA2
2.08E-119	1.21824465	0.817	0.246	6.979E-115	Plasma cells	ATP5ME
3.345E-119	0.97280623	0.809	0.23	1.122E-114	Plasma cells	COPB1
8.866E-119	1.32018016	0.749	0.219	2.974E-114	Plasma cells	HDLBP
9.825E-119	1.46507941	1	0.553	3.295E-114	Plasma cells	PTMA
1.027E-118	0.92593016	0.357	0.059	3.446E-114	Plasma cells	PPP1R16B
1.081E-118	1.24438966	0.425	0.081	3.625E-114	Plasma cells	TATDN3
1.093E-118	1.86448715	0.815	0.278	3.665E-114	Plasma cells	IRF2BP2
1.228E-118	1.19754352	0.665	0.169	4.12E-114	Plasma cells	CCT4
1.238E-118	0.96313909	0.409	0.074	4.154E-114	Plasma cells	NDUFA11
1.9E-118	1.12701896	0.341	0.055	6.372E-114	Plasma cells	MRPS23
2.229E-118	1.06786806	0.281	0.039	7.476E-114	Plasma cells	RIOK2
2.332E-118	1.08527086	0.586	0.137	7.823E-114	Plasma cells	DDX27
2.683E-118	0.72712894	0.801	0.22	9E-114	Plasma cells	ARL4C
3.941E-118	0.99672665	0.297	0.043	1.322E-113	Plasma cells	HAUS1
4.21E-118	1.19018395	0.989	0.398	1.412E-113	Plasma cells	RPS21
4.615E-118	1.23756606	0.951	0.331	1.548E-113	Plasma cells	BTF3
4.831E-118	1.02404361	0.281	0.039	1.62E-113	Plasma cells	STX18
5.256E-118	1.04629629	0.711	0.186	1.763E-113	Plasma cells	CMPK1
5.775E-118	1.29986036	1	0.546	1.937E-113	Plasma cells	RPL27A
6.611E-118	0.83638901	0.64	0.153	2.218E-113	Plasma cells	MYCBP
8.35E-118	0.99787757	0.379	0.065	2.801E-113	Plasma cells	MRPS16
8.687E-118	1.07727767	0.39	0.069	2.914E-113	Plasma cells	SETDB2
1.177E-117	1.23294061	0.341	0.055	3.948E-113	Plasma cells	NHLRC2
1.631E-117	1.65804502	0.728	0.218	5.472E-113	Plasma cells	MAPKAPK2
1.654E-117	0.97166699	0.411	0.075	5.549E-113	Plasma cells	HAX1
1.97E-117	1.15206639	0.744	0.206	6.608E-113	Plasma cells	DBI
2.067E-117	0.87392617	0.676	0.171	6.933E-113	Plasma cells	ANAPC5
2.303E-117	1.32024505	0.992	0.462	7.725E-113	Plasma cells	NACA
2.48E-117	1.05256183	0.665	0.167	8.32E-113	Plasma cells	BUB3
2.548E-117	1.21558507	0.635	0.158	8.545E-113	Plasma cells	SMC4
3.043E-117	0.88708949	0.36	0.06	1.021E-112	Plasma cells	MZT2B
3.604E-117	1.09741566	0.259	0.034	1.209E-112	Plasma cells	FTSJ1
4.405E-117	0.92615842	0.439	0.084	1.477E-112	Plasma cells	CCDC90B
6.071E-117	0.87150848	0.52	0.11	2.036E-112	Plasma cells	SNRNP27
6.514E-117	1.61678272	0.431	0.086	2.185E-112	Plasma cells	PEA15
6.972E-117	1.0588751	0.27	0.037	2.339E-112	Plasma cells	DLAT
7.152E-117	1.27962887	0.907	0.313	2.399E-112	Plasma cells	SLC25A3
9.841E-117	1.14608717	0.518	0.114	3.301E-112	Plasma cells	ZNRF2
1.109E-116	1.36309039	0.943	0.363	3.719E-112	Plasma cells	SERP1
1.375E-116	1.25858538	0.341	0.056	4.613E-112	Plasma cells	FAM98B
1.429E-116	1.17644581	0.575	0.135	4.794E-112	Plasma cells	RWDD1

1.455E-116	1.21754252	0.477	0.1	4.879E-112	Plasma cells	BRI3BP
2.634E-116	1.04839427	0.414	0.077	8.837E-112	Plasma cells	MRPS10
3.896E-116	1.03167987	0.381	0.067	1.307E-111	Plasma cells	PFDN2
4.52E-116	1.20405697	0.523	0.115	1.516E-111	Plasma cells	NOLC1
4.642E-116	0.91390259	0.499	0.104	1.557E-111	Plasma cells	TM9SF1
6.326E-116	0.90433218	0.507	0.107	2.122E-111	Plasma cells	PSMA5
6.876E-116	1.17235945	0.305	0.046	2.306E-111	Plasma cells	DNAJC4
9.234E-116	1.04832945	0.251	0.033	3.097E-111	Plasma cells	LSM7
9.418E-116	1.0757957	0.845	0.262	3.159E-111	Plasma cells	TM9SF3
1.196E-115	1.18864289	0.297	0.044	4.01E-111	Plasma cells	HSD17B10
1.37E-115	1.16654345	0.575	0.137	4.596E-111	Plasma cells	HSPA9
1.623E-115	1.11435453	0.575	0.135	5.444E-111	Plasma cells	NUTF2
2.479E-115	1.02585875	0.493	0.102	8.314E-111	Plasma cells	ORMDL3
2.612E-115	1.07770467	0.823	0.243	8.761E-111	Plasma cells	UBE2V1
3.131E-115	1.68283872	0.959	0.406	1.05E-110	Plasma cells	TMSB10
3.181E-115	1.04079928	0.289	0.042	1.067E-110	Plasma cells	TXLNA
3.7E-115	1.15381759	0.58	0.137	1.241E-110	Plasma cells	NDUFC1
5.137E-115	1.01196468	0.297	0.044	1.723E-110	Plasma cells	PI4K2B
5.736E-115	0.99324526	0.706	0.185	1.924E-110	Plasma cells	ILF3
6.013E-115	1.56252293	0.316	0.05	2.017E-110	Plasma cells	BEX4
8.569E-115	1.00431915	0.635	0.158	2.874E-110	Plasma cells	UBE2N
9.919E-115	1.07619555	0.251	0.033	3.327E-110	Plasma cells	AK6
1.412E-114	1.2987438	0.553	0.13	4.737E-110	Plasma cells	LARS
1.823E-114	1.1031477	0.275	0.039	6.116E-110	Plasma cells	GOT2
2.119E-114	1.05815699	0.272	0.038	7.107E-110	Plasma cells	TSFM
2.301E-114	1.61985825	0.924	0.36	7.719E-110	Plasma cells	CD164
2.414E-114	1.00148962	0.36	0.061	8.096E-110	Plasma cells	DUS1L
2.554E-114	0.97927257	0.676	0.171	8.567E-110	Plasma cells	NARS
2.626E-114	1.0064506	0.531	0.117	8.807E-110	Plasma cells	NDUFA3
2.838E-114	1.01734478	0.444	0.087	9.519E-110	Plasma cells	PHAX
3.216E-114	1.26940894	0.932	0.343	1.079E-109	Plasma cells	HNRNPA3
3.318E-114	1.1139662	0.556	0.127	1.113E-109	Plasma cells	FBL
3.66E-114	1.01087356	0.916	0.283	1.228E-109	Plasma cells	AHNAK
4.381E-114	1.03740326	0.368	0.064	1.47E-109	Plasma cells	NAT10
4.557E-114	1.22387464	0.602	0.151	1.528E-109	Plasma cells	TRIM44
4.811E-114	1.40853809	0.967	0.383	1.614E-109	Plasma cells	PLP2
4.897E-114	1.21842824	1	0.472	1.643E-109	Plasma cells	RPS3A
7.188E-114	1.17518161	0.433	0.085	2.411E-109	Plasma cells	ARHGAP5
1.087E-113	1.15354404	0.357	0.061	3.646E-109	Plasma cells	ARMC1
1.213E-113	0.9114819	0.537	0.119	4.07E-109	Plasma cells	YTHDF2
1.252E-113	1.13961833	0.51	0.112	4.198E-109	Plasma cells	AIMP1
1.607E-113	1.03306028	0.766	0.217	5.391E-109	Plasma cells	G3BP1
2.354E-113	1.04983817	0.403	0.075	7.894E-109	Plasma cells	PDHA1
2.828E-113	1.19848603	0.997	0.425	9.486E-109	Plasma cells	RPL27
3.551E-113	1.0020577	0.411	0.077	1.191E-108	Plasma cells	PDCD2
3.916E-113	1.07488216	0.567	0.134	1.314E-108	Plasma cells	TCERG1
5.851E-113	1.01524269	0.796	0.23	1.962E-108	Plasma cells	DDX24
1.347E-112	1.05585308	0.493	0.106	4.517E-108	Plasma cells	DAZAP1
1.647E-112	0.88481456	0.698	0.183	5.524E-108	Plasma cells	YWHAQ
1.715E-112	1.19889987	0.703	0.196	5.752E-108	Plasma cells	SMARCE1

2.579E-112	1.15069418	0.883	0.284	8.65E-108	Plasma cells	C11orf58
2.831E-112	1.13906743	0.992	0.388	9.496E-108	Plasma cells	RPL7
2.898E-112	1.38677319	0.305	0.047	9.722E-108	Plasma cells	TSPYL2
3.219E-112	1.06380183	0.706	0.191	1.08E-107	Plasma cells	SOD1
3.412E-112	1.25084238	0.27	0.038	1.144E-107	Plasma cells	TMEM147
3.768E-112	1.26057818	0.545	0.128	1.264E-107	Plasma cells	SDAD1
4.417E-112	0.96319557	0.76	0.217	1.482E-107	Plasma cells	GLS
4.46E-112	1.09773995	0.638	0.16	1.496E-107	Plasma cells	RSL24D1
4.735E-112	0.96947839	0.556	0.129	1.588E-107	Plasma cells	DARS
5.047E-112	1.17038107	0.455	0.093	1.693E-107	Plasma cells	ARL14EP
7.126E-112	1.2241582	0.883	0.298	2.39E-107	Plasma cells	SERBP1
8.771E-112	1.55981762	0.48	0.107	2.942E-107	Plasma cells	PPA1
9.598E-112	1.19580687	1	0.464	3.22E-107	Plasma cells	RPS20
1.114E-111	1.09439356	0.444	0.09	3.737E-107	Plasma cells	EIF3B
1.129E-111	1.09744952	0.992	0.403	3.786E-107	Plasma cells	RPS12
1.142E-111	0.73394534	0.48	0.1	3.831E-107	Plasma cells	MAP4
1.174E-111	1.00117295	0.267	0.037	3.939E-107	Plasma cells	BPNT1
1.332E-111	1.09105081	0.27	0.038	4.468E-107	Plasma cells	AIMP2
1.362E-111	1.07593551	0.272	0.039	4.569E-107	Plasma cells	SLC35B2
1.512E-111	1.22224777	0.455	0.095	5.073E-107	Plasma cells	N4BP2L1
1.532E-111	0.99379838	0.482	0.102	5.139E-107	Plasma cells	CNOT9
1.811E-111	0.94919322	0.48	0.101	6.073E-107	Plasma cells	NUP210
1.848E-111	0.906912	0.452	0.09	6.197E-107	Plasma cells	OTUD6B-AS1
3.558E-111	1.12259585	0.52	0.118	1.193E-106	Plasma cells	RAD50
3.872E-111	1.08668183	0.335	0.056	1.299E-106	Plasma cells	TRAPPC2L
5.61E-111	0.60386175	0.501	0.107	1.882E-106	Plasma cells	PPP3CC
6.018E-111	1.24149785	0.997	0.528	2.019E-106	Plasma cells	RPL17
6.5E-111	1.0204743	0.649	0.17	2.18E-106	Plasma cells	SRP54
8.142E-111	1.19090871	0.319	0.052	2.731E-106	Plasma cells	UTP4
8.149E-111	0.9334711	0.376	0.067	2.734E-106	Plasma cells	ZMIZ2
1.273E-110	1.19974797	0.967	0.357	4.272E-106	Plasma cells	ATP5MC2
1.893E-110	1.37581111	0.477	0.103	6.349E-106	Plasma cells	CTSH
2.2E-110	1.41752188	0.812	0.275	7.38E-106	Plasma cells	ATF4
2.385E-110	1.12770734	0.826	0.259	7.998E-106	Plasma cells	RPN1
2.962E-110	0.81560101	0.319	0.051	9.934E-106	Plasma cells	NSD2
3.81E-110	0.98865505	0.341	0.057	1.278E-105	Plasma cells	C12orf49
3.964E-110	1.08110506	0.556	0.132	1.33E-105	Plasma cells	CYP20A1
4.338E-110	0.63645684	0.362	0.063	1.455E-105	Plasma cells	ANKRD28
5.974E-110	0.98835801	0.564	0.132	2.004E-105	Plasma cells	EMC4
7.761E-110	1.04030223	0.335	0.056	2.603E-105	Plasma cells	CAPN15
1.004E-109	1.01096215	0.496	0.108	3.369E-105	Plasma cells	SNHG14
1.44E-109	1.00130548	0.46	0.096	4.831E-105	Plasma cells	ELAVL1
1.598E-109	0.81629392	0.466	0.097	5.361E-105	Plasma cells	RABL6
1.74E-109	1.09647096	0.324	0.053	5.836E-105	Plasma cells	C1QBP
1.862E-109	0.86616893	0.447	0.09	6.246E-105	Plasma cells	EPM2AIP1
2.273E-109	1.23909285	0.332	0.056	7.625E-105	Plasma cells	MRPS30
2.289E-109	1.07729691	1	0.392	7.679E-105	Plasma cells	RPL36
2.889E-109	0.88323819	0.433	0.085	9.691E-105	Plasma cells	PAGR1
2.999E-109	0.84588153	0.401	0.075	1.006E-104	Plasma cells	SCYL3
3.021E-109	1.07173155	0.477	0.102	1.013E-104	Plasma cells	NSUN2

3.146E-109	1.07064954	0.616	0.156	1.055E-104	Plasma cells	IPO7
3.221E-109	1.1790239	0.259	0.036	1.081E-104	Plasma cells	ZDHHC21
3.464E-109	1.0974809	0.294	0.045	1.162E-104	Plasma cells	CCT7
4.109E-109	1.06675837	0.286	0.043	1.378E-104	Plasma cells	MRPL35
4.194E-109	1.07474245	0.292	0.044	1.407E-104	Plasma cells	ERG28
4.663E-109	1.04371218	0.529	0.121	1.564E-104	Plasma cells	NDUFA5
7.715E-109	0.92885832	0.599	0.146	2.588E-104	Plasma cells	SELENOK
7.81E-109	1.01406059	0.422	0.083	2.62E-104	Plasma cells	MRPL18
1.388E-108	1.01527457	0.61	0.154	4.657E-104	Plasma cells	API5
1.463E-108	1.09858878	0.959	0.379	4.907E-104	Plasma cells	RPS26
1.499E-108	1.18759954	0.869	0.283	5.03E-104	Plasma cells	EIF4A1
1.679E-108	1.05403251	0.373	0.068	5.633E-104	Plasma cells	ARFIP2
1.871E-108	1.15980465	1	0.483	6.276E-104	Plasma cells	RPL13A
2.067E-108	1.32085801	0.856	0.313	6.934E-104	Plasma cells	NCOA3
2.502E-108	1.12149725	0.46	0.097	8.394E-104	Plasma cells	CCT3
2.701E-108	1.03833096	0.507	0.114	9.059E-104	Plasma cells	CAND1
4.316E-108	1.1829459	0.771	0.24	1.448E-103	Plasma cells	NUCKS1
4.344E-108	1.08670666	0.379	0.07	1.457E-103	Plasma cells	MTIF2
6.013E-108	0.97009696	0.354	0.063	2.017E-103	Plasma cells	OSBPL3
6.73E-108	1.07002803	0.771	0.23	2.257E-103	Plasma cells	COPB2
7.051E-108	0.79538215	0.662	0.172	2.365E-103	Plasma cells	CSNK2A1
7.209E-108	1.07769173	0.384	0.072	2.418E-103	Plasma cells	TULP4
7.468E-108	0.85464449	0.496	0.108	2.505E-103	Plasma cells	CLCN3
9.179E-108	1.34518311	0.398	0.077	3.079E-103	Plasma cells	SNHG7
1.146E-107	1.13430357	0.55	0.132	3.845E-103	Plasma cells	PSME2
1.321E-107	1.10024232	0.837	0.265	4.432E-103	Plasma cells	HNRNPR
1.594E-107	0.96295536	0.883	0.294	5.348E-103	Plasma cells	SEPTIN6
1.915E-107	1.05211436	0.327	0.055	6.424E-103	Plasma cells	PFDN6
2.402E-107	1.21097386	0.259	0.037	8.058E-103	Plasma cells	SMIM30
2.607E-107	1.07486064	0.501	0.112	8.746E-103	Plasma cells	SLIRP
2.674E-107	0.93276205	0.292	0.045	8.97E-103	Plasma cells	SKA2
2.95E-107	0.85451864	0.259	0.036	9.894E-103	Plasma cells	BSCL2
4.918E-107	0.96179601	0.403	0.077	1.65E-102	Plasma cells	ZNF92
5.697E-107	1.0674458	0.594	0.147	1.911E-102	Plasma cells	ABRACL
5.993E-107	1.06805709	0.567	0.137	2.01E-102	Plasma cells	NASP
6.744E-107	0.9796198	0.39	0.074	2.262E-102	Plasma cells	C5orf15
7.041E-107	1.28387767	0.351	0.063	2.362E-102	Plasma cells	PRDX2
8.763E-107	1.11600879	0.995	0.423	2.94E-102	Plasma cells	RPL35A
9.078E-107	1.00759136	0.409	0.081	3.045E-102	Plasma cells	RAB4A
9.088E-107	1.14692715	0.42	0.084	3.048E-102	Plasma cells	MIEF1
1.015E-106	0.99543593	0.466	0.1	3.404E-102	Plasma cells	KIAA0100
1.332E-106	0.69986749	0.447	0.091	4.468E-102	Plasma cells	MACO1
1.361E-106	0.83256041	0.272	0.04	4.565E-102	Plasma cells	BICD1
1.408E-106	1.0227149	0.283	0.043	4.722E-102	Plasma cells	TSR1
1.453E-106	0.94235677	0.466	0.099	4.873E-102	Plasma cells	TMED7
1.495E-106	1.09524198	0.302	0.048	5.014E-102	Plasma cells	FARSB
1.593E-106	1.01339572	0.308	0.05	5.342E-102	Plasma cells	MXD4
1.814E-106	1.03727273	0.267	0.039	6.084E-102	Plasma cells	NDUFAF4
2.073E-106	1.0140174	0.553	0.132	6.952E-102	Plasma cells	MRPL20
2.113E-106	1.42732567	1	0.905	7.088E-102	Plasma cells	MT-CO1

2.226E-106	0.8555888	0.436	0.088	7.467E-102	Plasma cells	SLC35C2
2.567E-106	1.00931557	0.564	0.136	8.61E-102	Plasma cells	VDAC3
2.681E-106	1.15336616	0.817	0.254	8.992E-102	Plasma cells	M6PR
2.8E-106	0.98237777	0.621	0.158	9.392E-102	Plasma cells	AIDA
2.978E-106	0.96512071	0.398	0.076	9.988E-102	Plasma cells	CIAO2B
3.973E-106	1.07946032	0.501	0.113	1.333E-101	Plasma cells	MPHOSPH10
4.333E-106	1.1562633	0.313	0.052	1.453E-101	Plasma cells	GNA12
4.458E-106	0.9955037	0.349	0.062	1.495E-101	Plasma cells	HPS5
5.282E-106	0.9778497	0.469	0.101	1.772E-101	Plasma cells	AASDHPPT
5.5E-106	1.12067664	0.362	0.066	1.845E-101	Plasma cells	GNL2
5.798E-106	1.27676862	0.308	0.05	1.945E-101	Plasma cells	PAXX
6.735E-106	1.14259327	0.431	0.088	2.259E-101	Plasma cells	NIFK
9.708E-106	0.99668537	0.997	0.395	3.256E-101	Plasma cells	RPL13
1.1E-105	1.14288591	0.504	0.116	3.69E-101	Plasma cells	UNC93B1
1.177E-105	1.15079879	0.469	0.103	3.948E-101	Plasma cells	SLC25A36
1.304E-105	0.94179486	0.327	0.055	4.374E-101	Plasma cells	TIMM8B
1.365E-105	1.03183262	0.45	0.095	4.58E-101	Plasma cells	CGAS
1.508E-105	0.49337883	0.373	0.067	5.057E-101	Plasma cells	GOLPH3L
1.618E-105	1.10623014	0.989	0.437	5.426E-101	Plasma cells	RPS14
1.716E-105	0.75136948	0.73	0.202	5.754E-101	Plasma cells	FAM214A
1.861E-105	0.96931775	0.858	0.263	6.244E-101	Plasma cells	ATP5PB
2.691E-105	1.21018097	0.379	0.072	9.025E-101	Plasma cells	BZW2
2.797E-105	1.18001589	0.594	0.151	9.383E-101	Plasma cells	PYURF
2.936E-105	1.15977916	0.316	0.053	9.847E-101	Plasma cells	GUF1
3.616E-105	0.82402797	0.526	0.121	1.213E-100	Plasma cells	ZMYND11
4.977E-105	1.17258128	0.272	0.041	1.67E-100	Plasma cells	NDUFAF8
5.516E-105	0.94096305	0.64	0.171	1.85E-100	Plasma cells	TPP2
6.561E-105	0.99986603	0.523	0.121	2.201E-100	Plasma cells	C19orf53
7.539E-105	0.97087116	0.33	0.056	2.529E-100	Plasma cells	PIGT
7.841E-105	0.94326697	0.392	0.076	2.63E-100	Plasma cells	SMAD5
9.566E-105	1.13051978	0.33	0.057	3.209E-100	Plasma cells	SMARCAD1
1.12E-104	1.06854317	0.7	0.198	3.757E-100	Plasma cells	SNHG6
1.692E-104	1.13723443	0.322	0.054	5.674E-100	Plasma cells	GTF2H3
1.929E-104	1.06125622	0.316	0.053	6.47E-100	Plasma cells	PSMB5
2.189E-104	1.21777082	0.995	0.463	7.344E-100	Plasma cells	RPL26
2.364E-104	1.20448315	0.864	0.304	7.931E-100	Plasma cells	RPL36AL
2.397E-104	1.18631713	1	0.632	8.041E-100	Plasma cells	RPL37
3.083E-104	1.90935103	0.526	0.14	1.034E-99	Plasma cells	ZBTB38
3.469E-104	1.16238987	0.267	0.04	1.164E-99	Plasma cells	ALG3
3.629E-104	1.08829155	0.719	0.209	1.217E-99	Plasma cells	LPXN
5.533E-104	0.80845829	0.559	0.134	1.856E-99	Plasma cells	BTBD1
8.648E-104	1.04988273	0.441	0.093	2.901E-99	Plasma cells	CASP10
1.037E-103	0.90451888	0.425	0.087	3.479E-99	Plasma cells	NUP88
1.1E-103	1.12876525	0.997	0.516	3.691E-99	Plasma cells	RPL9
1.414E-103	0.92191783	0.341	0.06	4.742E-99	Plasma cells	CRLS1
1.437E-103	1.07497705	0.343	0.061	4.821E-99	Plasma cells	GLRX5
1.706E-103	1.05814915	0.997	0.415	5.723E-99	Plasma cells	RPL19
1.91E-103	1.06543046	0.36	0.066	6.406E-99	Plasma cells	PLEKHA7
2.081E-103	0.67075753	0.42	0.085	6.98E-99	Plasma cells	DENND1B
2.083E-103	1.02898153	0.616	0.16	6.988E-99	Plasma cells	SRSF6
