# Supplementary material for: Neutrophil-enriched gene signature correlates with teplizumab therapy resistance in different stages of type 1 diabetes
Source: J Clin Invest. 2025 Sep 30;135(23):e176403. doi: 10.1172/JCI176403 (PMC12646666; doi:10.1172/JCI176403)
Supplement: Supplemental table 6 [file jci-135-176403-s294.pdf]

Symbol	Pearson_corr	p_value
VPS39	-0.713026415	9.79E-06
TMEM65	-0.66529652	6.04E-05
RP11-458F8.4	-0.625818529	0.00021674
MLXIP	-0.62265988	0.000238295
GNAQ	-0.619141433	0.000264522
LOXL2	-0.603285444	0.000417131
FAM83H	-0.601331596	0.000440486
RP11-274B21.2	-0.60123703	0.000441645
SLC35E2B	-0.599865154	0.000458763
CUL4B	-0.591690728	0.000573419
RYBP	-0.589254884	0.000612133
DLEU2	-0.588800178	0.000619608
MAML1	-0.586214484	0.000663663
CRISPLD2	-0.582381292	0.00073403
RANBP10	-0.577881224	0.000824919
RP11-384K6.6	-0.577819513	0.000826231
CAMK1D	-0.577315237	0.00083702
SEMA4D	-0.576988489	0.000844077
RNF175	-0.576388367	0.000857174
APPL2	-0.576360708	0.000857781
SETD1B	-0.575765157	0.000870962
IL13RA1	-0.574472483	0.000900183
USP19	-0.570447531	0.000996734
CELF2	-0.56834812	0.001050597
KIAA0430	-0.568316537	0.001051427
PTEN	-0.566818488	0.001091427
MAP2K7	-0.566214906	0.001107915
CCDC142	-0.565256807	0.001134535
IL1RAP	-0.565007924	0.001141541
LINC01127	-0.562474243	0.001215036
CHPT1	-0.561898896	0.001232289
HARS2	-0.559195822	0.001316236
JARID2	-0.55658434	0.001402028

LL22NC03-86G7.1	-0.555474917	0.001439923
NAMPTP1	-0.554002149	0.001491604
KMT2B	-0.553839399	0.001497413
RERE	-0.553127525	0.001523054
ZNF687	-0.553042815	0.00152613
TLR2	-0.551137637	0.001596761
GLTSCR1	-0.550035932	0.001638888
SZT2	-0.548297258	0.001707337
RP11-1334A24.6	-0.548282692	0.001707921
PTPRN2	-0.547325511	0.001746664
KDM7A	-0.547140057	0.001754258
TNNT3	-0.546579873	0.001777371
CASP2	-0.544168725	0.001879899
RNF44	-0.542922937	0.001934855
TPCN2	-0.542860272	0.001937656
KDM5B	-0.542392467	0.001958675
HEBP2	-0.542189062	0.001967876
SMAP2	-0.541878507	0.001981996
MYH11	-0.540783974	0.002032464
TRIM66	-0.54045351	0.002047918
SCAND2P	-0.540305848	0.002054857
ZFAND3	-0.540185325	0.002060535
MMP25-AS1	-0.539562635	0.00209009
BRD4	-0.539215526	0.002106723
SLC25A45	-0.53878835	0.002127351
ELF2	-0.538025872	0.002164604
MLLT1	-0.537444595	0.002193383
GSN	-0.53651188	0.002240253
CCNJL	-0.535706051	0.002281442
RNF144B	-0.53426512	0.002356728
NSUN7	-0.533263477	0.00241032
FOXO3	-0.533159361	0.00241595
MICALCL	-0.53120098	0.002523997
HIST1H4H	-0.530365836	0.002571327

SLC23A2	-0.528622363	0.002672611
RP11-54A4.2	-0.527639374	0.00273122
IRS2	-0.527631729	0.00273168
MAU2	-0.527542022	0.002737084
PDK1	-0.526841047	0.002779632
ELL	-0.526709784	0.002787662
SLC9A1	-0.526692428	0.002788725
GIT2	-0.526617303	0.002793332
ZNRD1-AS1	-0.525616553	0.002855325
MYH10	-0.523274784	0.003005042
USP4	-0.523189437	0.003010624
WWP2	-0.52285892	0.003032325
APH1B	-0.521563206	0.003118703
MTMR10	-0.520927879	0.003161825
CNTNAP3	-0.520911484	0.003162944
FAM63A	-0.519918317	0.003231403
COA1	-0.519466383	0.003262975
CCDC170	-0.519218027	0.003280437
USF3	-0.519142365	0.003285773
NDOR1	-0.51890152	0.003302808
RP11-81A1.6	-0.518584288	0.003325362
ANKRD33B	-0.518013895	0.003366248
SLC31A2	-0.517988313	0.003368091
N4BP1	-0.51762633	0.003394274
PPP6R3	-0.516686253	0.00346309
TENM1	-0.515787151	0.003530025
WDTC1	-0.515711529	0.003535706
DHX33	-0.515475158	0.003553511
DHX40	-0.515054435	0.003585393
ANGPT1	-0.514106216	0.003658151
RP11-434B12.1	-0.513550817	0.003701354
UBE4B	-0.513397047	0.003713392
RPGRIP1	-0.51325584	0.003724476
LINC00174	-0.51320006	0.003728863

OSBPL2	-0.512978823	0.003746304
YY1AP1	-0.512939717	0.003749394
CREB1	-0.512938139	0.003749519
TMPPE	-0.511960397	0.003827498
ERGIC1	-0.511952152	0.003828162
TBC1D30	-0.511384906	0.003874044
PCYT1A	-0.51108455	0.003898529
RAB3D	-0.510455668	0.003950225
LINC00854	-0.510454758	0.0039503
SP3	-0.510078277	0.00398153
MAP7	-0.509599927	0.004021514
ITGA5	-0.509411842	0.00403733
SSH2	-0.50931227	0.004045724
PPP1R15B	-0.509216983	0.004053771
DNHD1	-0.508690616	0.004098471
RP11-295D4.3	-0.508554651	0.004110086
BROX	-0.508451271	0.004118936
LRRN2	-0.508384755	0.004124639
ENKUR	-0.508258116	0.004135516
UVSSA	-0.507529086	0.004198608
C7orf49	-0.507332584	0.004215754
HERC4	-0.506981405	0.004246547
ZBTB34	-0.506595521	0.004280604
CIC	-0.506326052	0.004304525
ZCCHC6	-0.505710577	0.004359592
MAP3K14-AS1	-0.505628082	0.004367019
MED13L	-0.505144802	0.004410743
PKP4	-0.504792492	0.004442854
DHDDS	-0.504634682	0.004457302
ZNF655	-0.504576785	0.004462613
VPS52	-0.504006272	0.004515235
ACOX1	-0.503851094	0.004529639
MXI1	-0.503752658	0.004538797
FAM157C	-0.503614622	0.004551666

GPSM2	-0.503517477	0.004560741
KLHL21	-0.503474054	0.004564802
PARP8	-0.503006997	0.004608684
ZNF611	-0.502940405	0.00461497
CLMN	-0.502345781	0.004671424
ATF6	-0.501946939	0.004709621
JPX	-0.501913739	0.004712812
ACTN1	-0.501624642	0.004740682
PELI1	-0.501472946	0.004755362
KBTBD7	-0.501435932	0.004758949
SNTB2	-0.501299932	0.004772152
AC006547-13	-0.5009811	0.004803226
KDM3B	-0.500864688	0.004814615
LUCAT1	-0.500850271	0.004816027
TNRC6B	-0.500697878	0.004830975
TRAPPC6B	-0.500479381	0.004852475
UPF1	-0.500332458	0.004866978
TLE3	-0.500269933	0.004873162
GORASP1	-0.500144767	0.00488556
RP11-332M2.1	-0.499735789	0.00492626
MAPKBP1	-0.499589801	0.004940858
APC	-0.499250873	0.004974891
P2RY13	-0.499124559	0.004987626
DNASE1	-0.498749687	0.005025584
TGFBR2	-0.498172295	0.005084532
ZFAND4	-0.498066939	0.005095352
SIRPA	-0.497701931	0.005132988
EXOC8	-0.49758248	0.005145357
ADAMTSL4-AS1	-0.497298426	0.00517487
SUN1	-0.496848444	0.00522192
BAZ2B	-0.496727868	0.00523459
ZNF641	-0.495592169	0.005355212
PHC2	-0.494826754	0.005437838
AGO4	-0.494662753	0.005455682

FAM214A	-0.494032176	0.005524759
TM6SF1	-0.493995614	0.005528787
GATAD2A	-0.493880497	0.005541485
AVL9	-0.493877343	0.005541834
INPP5A	-0.493836996	0.00554629
KDM6B	-0.493829029	0.005547171
ERVK3-1	-0.493803047	0.005550043
RICTOR	-0.493073323	0.005631225
MZF1	-0.492826424	0.00565892
BTBD19	-0.49277314	0.005664913
CTD-2587H24.14	-0.492670568	0.005676463
PSTPIP2	-0.492272282	0.005721503
INO80D	-0.492108016	0.005740168
PACSIN2	-0.491822229	0.005772764
TRIB1	-0.491637173	0.005793955
ERMN	-0.491176313	0.005847016
BAIAP3	-0.491132337	0.005852101
PLD1	-0.490656843	0.005907321
MCL1	-0.490261724	0.005953544
TACC3	-0.489867275	0.005999996
NPRL3	-0.489766884	0.006011867
SCYL1	-0.48951434	0.006041819
TBL1X	-0.489269761	0.006070948
TMCC1	-0.489007192	0.006102352
POLM	-0.488464444	0.006167704
DBF4B	-0.488327892	0.00618424
ATP6V1A	-0.48828547	0.006189384
RP11-274B21.3	-0.487742131	0.0062556
DENND3	-0.487519503	0.006282905
XPO6	-0.487367995	0.006301545
FBXL20	-0.48722361	0.006319352
PRR14L	-0.487040337	0.006342017
BOD1L1	-0.486739065	0.006379425
CEP85L	-0.48671343	0.006382617

KIAA0556	-0.485974495	0.006475203
UBR2	-0.485897978	0.006484855
SLC12A6	-0.485665359	0.006514273
C5AR2	-0.485529812	0.006531468
GSK3B	-0.485282921	0.006562885
RASSF2	-0.485024309	0.006595932
SLCO3A1	-0.484975831	0.006602142
HGD	-0.484863399	0.006616565
TANGO2	-0.484769919	0.006628577
MICAL2	-0.484565634	0.006654891
WDFY2	-0.484418868	0.006673851
PPP2R2D	-0.484369842	0.006680195
MIRLET7BHG	-0.484331831	0.006685117
H6PD	-0.484272094	0.006692858
KEL	-0.484058943	0.006720543
RP11-44M6.7	-0.483627507	0.006776876
ZNF486	-0.483534565	0.006789064
SOCS4	-0.483470018	0.006797539
NCOA1	-0.483395087	0.006807389
ZMIZ1	-0.483365498	0.006811282
KIF1B	-0.483061296	0.006851415
NEK6	-0.48306071	0.006851492
TUBGCP4	-0.482666502	0.006903798
ATXN1	-0.482532225	0.006921692
RAF1	-0.482408459	0.00693822
EPOR	-0.482278262	0.006955643
DOCK5	-0.482090541	0.006980829
SLC6A12	-0.481990096	0.006994337
LINC00641	-0.481976881	0.006996116
RRP12	-0.481518656	0.007058035
C3orf62	-0.481402957	0.007073742
RASGRP4	-0.480861292	0.007147674
TRIM38	-0.480561695	0.007188847
ZER1	-0.480463136	0.007202436

ZNF700	-0.480457893	0.007203159
CD93	-0.480151941	0.007245484
KLRK1	-0.479665663	0.007313188
MOB3A	-0.479590942	0.007323639
TET3	-0.479579987	0.007325172
AREL1	-0.47956682	0.007327015
SLC16A5	-0.479509769	0.007335006
ARHGEF11	-0.479350338	0.007357376
CFLAR	-0.479237336	0.007373267
ULK1	-0.47918765	0.007380263
FNIP1	-0.479161777	0.007383909
NRD1	-0.479054231	0.007399077
PTAFR	-0.478943926	0.007414663
PATL1	-0.478818156	0.007432468
AC024560-3	-0.478285444	0.007508283
TOM1L2	-0.478189219	0.007522047
STAM2	-0.478174383	0.007524171
RBM33	-0.478133315	0.007530053
RELL1	-0.478085914	0.007536848
PREX1	-0.478066376	0.00753965
IL17RA	-0.478060765	0.007540455
ASPH	-0.478011815	0.007547479
BIN3	-0.477861951	0.00756902
STK35	-0.477797729	0.007578267
MICALL2	-0.477690819	0.007593682
BRWD3	-0.4771793	0.007667802
SPAG9	-0.477098606	0.007679551
PHOSPHO1	-0.476521805	0.007763974
FAM131A	-0.476450747	0.007774428
RALGAPA2	-0.476412946	0.007779995
RP11-403I13.8	-0.476325616	0.007792867
DNM2	-0.476229403	0.00780707
NLRP6	-0.47561989	0.007897556
IGF2BP2	-0.475499989	0.00791546

MFSD8	-0.475499203	0.007915577
RP11-802E16.3	-0.475439202	0.00792455
GNAI3	-0.475159195	0.007966536
C16orf54	-0.475076082	0.007979035
RP11-59C5.3	-0.474791291	0.008021988
SP1	-0.474612751	0.008049015
PRPF3	-0.474486074	0.008068239
FAM129A	-0.4743667	0.008086389
VWCE	-0.474255961	0.008103257
AP1G1	-0.474191505	0.008113089
ENTPD1	-0.474147667	0.008119782
MDM2	-0.473793714	0.008173991
DCAF12	-0.473682555	0.008191079
PLPPR2	-0.473673442	0.008192481
BORCS8	-0.473546184	0.008212083
NUAK2	-0.473464631	0.008224666
METTL22	-0.473401356	0.00823444
MT-TT	-0.473325105	0.008246231
ITPRIP	-0.47331355	0.008248019
STRADB	-0.473003679	0.008296093
AHCTF1	-0.472783596	0.00833038
BRCA1	-0.472094771	0.008438469
SUSD6	-0.472072335	0.008442009
CTD-3252C9.4	-0.472066064	0.008442999
BBIP1	-0.472031497	0.008448457
C18orf25	-0.471462359	0.008538747
ANKRD9	-0.471391718	0.00855001
NOL4L	-0.471064997	0.008602267
RP11-455F5.5	-0.470656025	0.00866806
LGALSL	-0.470630254	0.00867222
MYSM1	-0.470529166	0.008688554
RILP	-0.470517534	0.008690435
CDS2	-0.470450688	0.008701253
WDR55	-0.470224456	0.008737948

ARHGAP19	-0.469841451	0.00880037
MFN2	-0.469839901	0.008800623
BTNL8	-0.469800804	0.008807016
ASAP1	-0.469797147	0.008807615
ALOX12-AS1	-0.4696915	0.008824911
MBOAT7	-0.469590372	0.008841494
FAM210B	-0.469292329	0.008890521
NCOA4	-0.469160117	0.008912343
RP11-495P10.1	-0.469083238	0.008925052
IL6R	-0.468768191	0.008977296
TIMP2	-0.468739606	0.008982049
CACNB4	-0.468696704	0.008989187
PANX2	-0.468651411	0.008996727
SLC22A23	-0.468632103	0.008999943
FOXO4	-0.468319246	0.00905219
ZNF319	-0.468130028	0.009083912
PPFIA1	-0.467909523	0.009120999
PLEKHM1	-0.467593207	0.009174423
ARHGEF40	-0.46742863	0.009202323
ANKRD44	-0.467265598	0.009230032
IRF2	-0.467070985	0.0092632
PSEN1	-0.466981691	0.009278453
HCG27	-0.466623163	0.009339906
RN7SK	-0.466231973	0.009407348
ZFC3H1	-0.466175065	0.009417193
SLA	-0.466135228	0.00942409
PCBP1-AS1	-0.465921013	0.009461251
WDR37	-0.465645684	0.009509194
PIGB	-0.465582198	0.009520278
RP11-159D12.2	-0.465558247	0.009524462
ZNF746	-0.465497748	0.009535039
HDAC7	-0.465352644	0.009560446
AC005154-6	-0.465316392	0.009566803
ELOVL5	-0.465264643	0.009575882

KDM2A	-0.465195823	0.009587969
NUMB	-0.465086664	0.009607167
AC116366-5	-0.465006737	0.009621244
KMT2C	-0.464966037	0.009628418
CAMK2G	-0.464823053	0.009653661
SEC14L1	-0.464619588	0.009689676
RP11-430C7.5	-0.464299245	0.009746609
CHMP3	-0.464276406	0.009750679
FRS3	-0.463992484	0.009801392
FBXO41	-0.463897198	0.009818461
PI4KAP1	-0.463876517	0.009822169
CEP63	-0.463852666	0.009826447
LPGAT1	-0.463811326	0.009833865
TPTEP1	-0.463781499	0.009839221
UBN1	-0.463576755	0.009876048
C1RL	-0.463484628	0.009892657
TTC17	-0.463479851	0.009893519
PFKFB4	-0.463250779	0.009934921
AP001062-7	-0.4631151	0.009959512
RBM47	-0.46299332	0.009981628
