# Supplementary material for: Neutrophil-enriched gene signature correlates with teplizumab therapy resistance in different stages of type 1 diabetes
Source: J Clin Invest. 2025 Sep 30;135(23):e176403. doi: 10.1172/JCI176403 (PMC12646666; doi:10.1172/JCI176403)
Supplement: Supplemental table 7 [file jci-135-176403-s295.pdf]

Supplemental Table 7: List of genes correlating with time to clinical diagnosis in TN10 Baseline.

Symbol	Pearson_corr	p_value
NOP10	0.580199852	5.64E-05
PSMB1	0.554871142	0.000137015
ANXA2	0.54556119	0.000186558
TTC1	0.535584051	0.000257138
PSMB3	0.530903329	0.000297892
ATP6V0E1	0.52920566	0.000314054
TSPO	0.521557759	0.000397085
TAF10	0.521141281	0.000402126
CLIC1	0.520044499	0.000415679
CD63	0.519554654	0.000421863
ARID1A	0.518191387	0.000439511
PSMB8	0.516952714	0.000456118
MYL12B	0.516668429	0.000460008
RAC2	0.516612539	0.000460776
CAPZB	0.514936776	0.000484352
NDUFA1	0.512838714	0.00051539
PA2G4	0.512366453	0.000522618
JTB	0.51173682	0.000532395
TIMP1	0.507256461	0.000606833
MT2A	0.504956476	0.000648551
PDXK	0.504754042	0.000652343
POLR2J	0.503744194	0.000671557
JPT1	0.502165383	0.00070261
CDK11B	0.501753883	0.000710911
MAPKAPK5-AS1	0.500905892	0.000728296
NFKBIA	0.498736253	0.000774515
TXN	0.498389935	0.00078213
FBRS	0.497195665	0.000808905
UBE2M	0.496860581	0.000816563
ALDOA	0.495848903	0.000840077
COX6A1	0.495305028	0.000852966
MZT2B	0.494777392	0.000865639

RHOG	0.494534186	0.000871537
THRAP3	0.493008935	0.000909346
NDRG1	0.49194471	0.000936586
NR1H3	0.491040824	0.000960291
COX7B	0.489978229	0.000988839
CCL5	0.488844745	0.001020122
GNAI2	0.488484456	0.001030249
UBE2K	0.487944161	0.001045602
SMIM26	0.486820948	0.001078173
S100A10	0.486593457	0.001084879
EMP3	0.486089921	0.001099854
SSR2	0.485552635	0.001116034
TERF2IP	0.484623991	0.001144501
CORO1A	0.483906028	0.001166949
PYCARD	0.483051023	0.001194192
COX5B	0.482679825	0.001206194
GRN	0.482586427	0.001209231
NEDD8	0.48026256	0.001287008
GSTP1	0.479912511	0.001299101
ALOX5AP	0.479816054	0.001302451
DYNLRB1	0.479253422	0.001322144
COPE	0.47853397	0.00134771
GAPDH	0.477856845	0.001372171
HLA-A	0.477411835	0.001388461
DPY30	0.476956199	0.001405317
FAM89B	0.475005949	0.001479535
PSME1	0.474688161	0.001491952
CNOT3	0.474648694	0.0014935
ACTG1	0.474208085	0.001510885
BAG6	0.47258726	0.001576389
RPLP0P6	0.471173261	0.001635574
EBP	0.470898012	0.001647321
CAPNS1	0.470776026	0.001652551
DAD1	0.467720077	0.001788453

CST3	0.46674754	0.001833727
LSP1	0.466648261	0.001838406
YWHAB	0.465807244	0.001878461
LMO4	0.465312487	0.001902383
ARL6IP5	0.465088964	0.001913278
NDUFA3	0.46466046	0.00193432
OR7E38P	0.464607895	0.001936915
NDUFA4	0.464059951	0.001964151
ZNF322	0.464006809	0.00196681
KRT18	0.46386883	0.00197373
DYNLL1	0.461734274	0.00208355
NDUFB10	0.460163838	0.002167753
AGTRAP	0.459819611	0.002186606
COX8A	0.459384281	0.002210654
PFN1	0.459203049	0.002220735
NECAB3	0.459176614	0.002222208
MAF	0.45844082	0.002263575
ISG20	0.458174784	0.002278698
COTL1	0.457608187	0.002311203
CHMP2A	0.457310685	0.002328433
RFLNB	0.457099245	0.002340747
CALM1	0.45692636	0.002350859
RNF167	0.456755798	0.002360872
MGST3	0.456599172	0.002370101
TMEM14A	0.454161893	0.002517851
NDUFAF2	0.453922313	0.002532803
TRBV10-2	0.453248435	0.002575278
TMEM258	0.452867613	0.002599558
COX7A2	0.452706752	0.002609874
UBL5	0.452689874	0.002610958
TMEM219	0.452347818	0.002633022
TRAPPC2L	0.45115992	0.002710924
PHF20	0.451010934	0.002720836
S100A6	0.449619041	0.002814987

C4orf3	0.449522703	0.002821608
MRPL34	0.449405629	0.002829673
BCAP31	0.449259754	0.002839749
ARHGDIB	0.449016158	0.002856647
POLE4	0.448890268	0.002865414
GOLGA2	0.44879633	0.002871972
IFITM1	0.448322843	0.002905225
COX6B1	0.44815374	0.002917182
SMIM29	0.44720734	0.002984905
UBALD2	0.446186099	0.003059527
WASF2	0.445901699	0.003080597
DBI	0.445536728	0.003107822
HLA-F	0.445099584	0.003140709
SF3B2	0.444765563	0.003166043
SMAP1	0.444029126	0.003222532
TPT1	0.443796904	0.003240527
ARF3	0.443635466	0.003253089
NDUFB8	0.443397141	0.003271711
HLA-E	0.443152591	0.003290917
H2AZ1	0.442911392	0.003309957
S100A11	0.442779331	0.003320422
CXCR4	0.442433135	0.003347994
CDYL	0.442389404	0.003351491
COX14	0.442245901	0.003362989
SMIM27	0.442150032	0.003370689
IL2RG	0.442065479	0.003377494
SPNS3	0.442038602	0.003379659
UXT	0.441968069	0.003385348
EIF2S2	0.441927731	0.003388605
MAP2K2	0.441756176	0.003402488
PPDPF	0.441218968	0.003446284
TMSB4XP8	0.440985135	0.003465501
TRMT112	0.440911952	0.003471534
VIM	0.440727551	0.003486778

TPI1	0.440516336	0.00350431
TBCB	0.440380432	0.003515631
ELOB	0.440111452	0.003538133
SSR4	0.439934324	0.00355302
FCGRT	0.439929031	0.003553466
HSP90AA1	0.439644424	0.003577503
UQCRH	0.439295933	0.003607129
FCER1G	0.43904104	0.003628934
WDR83OS	0.43813407	0.003707458
CSTA	0.43788111	0.003729622
CALM2	0.437155164	0.003793873
CDC42	0.437008171	0.003807
STX2	0.436281041	0.00387252
NKAPD1	0.435250275	0.003967087
LSM14B	0.434701365	0.004018265
MRPL54	0.43462115	0.004025792
GMFG	0.434587751	0.004028929
RRP36	0.434234057	0.004062287
RPL36AL	0.433614103	0.004121339
NPC2	0.433046915	0.00417602
PPP3CB	0.432203825	0.004258468
EIF3K	0.432060339	0.004272641
MTPN	0.43181548	0.004296921
SLIRP	0.431694151	0.004308996
GINS3	0.431660069	0.004312394
TIMM17B	0.430613454	0.004417867
AIF1	0.429978668	0.004482927
EBPL	0.429689019	0.004512889
ATP5ME	0.42883687	0.004602054
CIR1	0.428182024	0.004671612
PIAS4	0.428000844	0.004691018
FIS1	0.427801388	0.004712463
IFITM2	0.42774763	0.004718257
LAMP1	0.427674114	0.004726191

CSNK2B	0.42765955	0.004727764
HIGD2A	0.427523887	0.00474244
SSBP3	0.427264644	0.004770595
S100A12	0.427225925	0.004774813
COX7C	0.427123401	0.004785996
TXNDC17	0.42694766	0.004805219
LSM8	0.426825528	0.004818618
PSME2	0.426814901	0.004819785
LRRFIP1	0.426795547	0.004821912
TMA7	0.4264521	0.004859786
CD2	0.425694549	0.004944244
CFL1	0.425303571	0.00498833
DUSP5	0.425160086	0.005004595
ZNF16	0.424832435	0.005041909
PPP4C	0.424590242	0.005069646
FCN1	0.424198342	0.005114809
HCST	0.423906326	0.005148688
PSMB4	0.423823494	0.005158334
PSMA5	0.423785921	0.005162714
HIKESHI	0.422685177	0.005292486
SH3BGRL3	0.422683891	0.005292639
RPL35A	0.422224748	0.005347602
SLC66A2	0.422113731	0.005360966
SURF1	0.421985913	0.005376388
HSPB1	0.421932659	0.005382825
RPS12	0.421732228	0.005407112
ATP6V1F	0.420854739	0.005514561
EIF1	0.420266863	0.005587579
NDUFB9	0.420075256	0.005611558
LTC4S	0.419863344	0.005638183
SNF8	0.419587382	0.005673018
TRBC2	0.419532799	0.005679931
LAMTOR5	0.418575917	0.005802301
LGALS1	0.418185635	0.005852864

CARHSP1	0.417845354	0.005897261
RPL5	0.417662096	0.005921291
C1QA	0.417659635	0.005921615
RPS18	0.416713931	0.006046983
TM7SF2	0.416430622	0.006084985
PGP	0.41638739	0.006090803
ST6GALNAC1	0.415943918	0.006150753
RPL3	0.41537592	0.006228284
TRIR	0.415363061	0.006230048
RPS3	0.415333295	0.006234136
GPX4	0.415136254	0.00626125
ENO1	0.415125271	0.006262765
PPP1CA	0.414812986	0.006305956
BRK1	0.414786685	0.006309606
TYROBP	0.414638091	0.006330258
RPL30	0.414373373	0.006367195
TFF3	0.414317767	0.006374977
BTG1	0.414199402	0.006391571
S100A4	0.413974818	0.006423158
HBM	0.413697748	0.006462313
ATP6V1G1	0.413574165	0.006479844
SKP1	0.413435352	0.006499585
CNPY3	0.413403041	0.006504187
SOD1	0.41339126	0.006505866
CHCHD2	0.413045142	0.006555355
SLC25A3	0.41283532	0.006585515
UBA52	0.412552351	0.006626378
BRD4	0.412403635	0.006647942
DCXR	0.4123819	0.006651098
PARK7	0.412360886	0.006654152
CYBA	0.412342917	0.006656763
RACK1	0.411523159	0.006776858
PRELID1	0.411327808	0.006805751
TRBV19	0.411304351	0.006809228

RPS4X	0.411246466	0.006817813
SWI5	0.410837157	0.006878791
SRP14	0.41051541	0.006927054
HLA-DRB6	0.41040174	0.006944175
AP2S1	0.41031173	0.006957758
UBE2R2	0.410260317	0.006965527
RPL19	0.410224074	0.006971008
SEC61B	0.410196874	0.006975123
ATP5MJ	0.410170889	0.006979057
RPS13	0.41007021	0.006994318
NDUFAF8	0.409778992	0.007038621
TLNRD1	0.409507941	0.007080075
CARD16	0.408645246	0.007213417
TMEM208	0.408126133	0.007294693
ARF5	0.408055728	0.007305777
LMAN2	0.407653876	0.007369318
NDUFS5	0.407630241	0.00737307
EEF1A1	0.407472086	0.00739822
PHPT1	0.407435632	0.007404027
CBR3	0.407304318	0.007424979
RPL10	0.407238092	0.007435564
S1PR4	0.407017589	0.007470905
OST4	0.406865867	0.007495305
FXYD5	0.406672173	0.007526556
POMP	0.406413007	0.007568545
NIBAN2	0.406153492	0.007610793
TMEM59	0.405902671	0.007651819
AURKAIP1	0.405537503	0.007711889
RBM8A	0.405526463	0.007713712
NDUFS6	0.405044219	0.007793679
PRDX5	0.404952095	0.007809037
GPX1	0.404914387	0.00781533
SELPLG	0.40478143	0.007837556
HLA-C	0.404676337	0.007855162

VPS4B	0.404633136	0.00786241
ATP5PF	0.404562341	0.007874299
PTGES3	0.404170871	0.00794032
UQCR10	0.404135321	0.007946339
H3-3B	0.403999999	0.007969287
RPL13A	0.40386513	0.007992215
MCTS1	0.403245872	0.008098219
EDF1	0.4031465	0.008115341
PLAAT4	0.402999179	0.008140783
SNHG9	0.402976411	0.008144721
PFDN5	0.402976193	0.008144759
IL32	0.402795164	0.008176129
ENSG00000235859	0.402627302	0.008205311
RPL37	0.402507991	0.008226107
NDUFA2	0.402429948	0.008239734
GSTO1	0.402369148	0.008250364
CTSD	0.40233246	0.008256784
PTP4A2	0.402196785	0.008280563
LST1	0.402178087	0.008283845
FTLP3	0.401941163	0.008325525
DOK2	0.401905116	0.008331882
SEC61G	0.401728028	0.008363174
CIB1	0.401704257	0.008367382
ATP5PD	0.40142702	0.008416595
BRWD3	0.401264805	0.008445505
TXNIP	0.400895681	0.008511609
TMBIM6	0.400684524	0.008549624
RPL31	0.400678192	0.008550766
GADD45G	0.399254377	0.008810956
COX4I1	0.397879432	0.009068642
RPL35	0.397802999	0.009083155
DEK	0.397403607	0.009159314
TRAPPC1	0.397188653	0.009200529
PPIB	0.396815787	0.0092724

FKBP2	0.396552357	0.009323467
CD52	0.396332074	0.009366354
ATP5PO	0.396319857	0.009368738
ATOX1	0.395965062	0.009438185
C19orf53	0.395507536	0.009528392
PSMA7	0.395495138	0.009530847
SDHAF2	0.395375735	0.009554515
RPA3	0.395135036	0.00960238
NAA38	0.394779438	0.00967347
CCNI	0.39468889	0.009691644
KLF2	0.39408228	0.009814154
CST7	0.39313656	0.010007795
PBDC1	0.392841432	0.010068891
ATP5MC3	0.392470092	0.010146216
MTDH	0.392282284	0.010185518
COX17	0.392260482	0.010190088
KLF6	0.392244826	0.010193372
RNF181	0.392197414	0.010203321
DIAPH1	0.392144694	0.010214393
CITED2	0.391973312	0.010250458
GNB2	0.391854019	0.010275625
OAZ1	0.391655164	0.010317696
ATP5F1E	0.391586517	0.010332254
SPCS1	0.39149765	0.010351125
ID2	0.39136699	0.010378925
JOSD2	0.391241183	0.010405753
IMP3	0.391222183	0.01040981
UQCR11	0.391116902	0.010432314
ATP6V0B	0.389071538	0.010877845
ATP5MK	0.389018278	0.01088966
HINT1	0.388657692	0.010969942
SLC25A6	0.388587829	0.010985555
SF3B5	0.38829012	0.011052299
MKNK2	0.388214938	0.011069209

ZNF667	0.388146772	0.01108456
COX6C	0.3881406	0.011085951
RAN	0.387898699	0.01114058
EHD1	0.38761376	0.011205223
VKORC1	0.387383306	0.011257739
RPS6	0.387347715	0.011265868
RPL27	0.386504003	0.011460052
FAU	0.38622367	0.0115252
C19orf38	0.385794174	0.011625627
RPL29	0.385490973	0.011696972
ARPC3	0.385433415	0.011710558
RBX1	0.385387007	0.011721521
RPL11	0.385283466	0.011746014
MFSD10	0.385169224	0.011773089
ENSG00000241990	0.385133316	0.01178161
RPS8	0.384527149	0.01192625
RPS9	0.384294383	0.011982192
PABPC1	0.384289811	0.011983294
USP47	0.384271244	0.011987766
NELFE	0.384243545	0.01199444
SNRPD2	0.384214502	0.012001442
CLC	0.383505857	0.01217337
RPS23	0.383034569	0.012288869
RPL8	0.38294501	0.012310923
NUDT2	0.382868228	0.012329857
CBX5	0.382794005	0.012348184
ENSG00000260563	0.38202781	0.012538728
ARPC4	0.382018141	0.012541149
TMEM141	0.381926492	0.012564111
LAMTOR4	0.381840021	0.012585809
RTF1	0.381807701	0.012593927
RPS25	0.381630882	0.01263842
RPL36	0.381501833	0.012670977
IQGAP2	0.381468036	0.012679515

NDUFB7	0.381413813	0.012693224
ATP6AP1-DT	0.381388579	0.012699608
IGSF6	0.381059912	0.012783008
ABHD14A	0.380778956	0.01285467
MYL12A	0.380692935	0.012876679
TALDO1	0.380662596	0.012884449
ANP32A	0.380386377	0.012955375
TOMM5	0.380249607	0.012990618
RPL34	0.380114841	0.013025423
HSPA8	0.379990907	0.0130575
MACROH2A1	0.379887116	0.013084416
NDUFB1	0.379782126	0.013111691
GPSM3	0.379734855	0.013123987
RPL26	0.379689625	0.013135761
TUBB4B	0.379604714	0.01315789
RPL32	0.379553709	0.013171197
RPL39	0.379487905	0.013188383
ZNRF1	0.379464725	0.013194441
NDUFS3	0.379452862	0.013197542
RPL18	0.379443146	0.013200083
VAMP8	0.379335544	0.013228247
CEBPE	0.379323358	0.01323144
RPS24	0.378459323	0.013459498
ATP5F1EP2	0.378242677	0.013517201
TMEM205	0.37805576	0.013567155
NSMCE1	0.37804335	0.013570478
CHCHD1	0.3778769	0.013615103
KCNK6	0.377711029	0.013659697
UQCRB	0.377625644	0.013682701
PPP2R2A	0.376970216	0.013860381
RPS11	0.376725834	0.01392713
RPS15	0.376692049	0.013936379
HAX1	0.376564835	0.013971253
GIMAP7	0.376257412	0.014055835

CTDNEP1	0.376179127	0.014077443
RPL4	0.3761781	0.014077727
ATP5MC2	0.37605661	0.014111316
CD3D	0.375644631	0.014225728
RPL12	0.375140126	0.014366905
CD99	0.373545533	0.014820967
LAPTM5	0.373413969	0.014858967
H4C3	0.373104545	0.014948664
RABAC1	0.372974922	0.014986376
RPS14	0.372858575	0.015020294
DUSP23	0.372517694	0.015120043
LTB	0.372173409	0.015221358
ATP5MG	0.372009803	0.015269704
PSMB6	0.371792744	0.015334047
SCAND1	0.371487503	0.015424917
POLR2L	0.371202251	0.015510249
EIF2S3	0.37112079	0.015534691
ARRDC1	0.371063878	0.015551786
SNRPF	0.370808183	0.015628789
PSMD4	0.370736673	0.015650382
BPTF	0.370314311	0.015778432
PLXNA3	0.369954328	0.015888269
PSENEN	0.369552659	0.016011588
RPLP0	0.369495021	0.01602935
CDKN1B	0.369362254	0.016070328
RPS20	0.369341334	0.016076793
CFD	0.369291257	0.016092277
ERP29	0.369115683	0.016146665
CD151	0.368714563	0.016271505
TXNL4A	0.368615534	0.01630245
TAGLN2	0.36853046	0.016329075
RPL41	0.368522423	0.016331592
TRADD	0.367619919	0.016616345
KDM6B	0.367504697	0.016653

FPR1	0.367406438	0.016684312
TRBC1	0.367189333	0.016753672
CSTB	0.367183683	0.01675548
SYAP1	0.366784416	0.016883685
RPS19	0.366745683	0.016896166
RPL38	0.366693329	0.016913049
IER2	0.366668673	0.016921004
H2AZ2	0.366586164	0.016947651
FRG1BP	0.366325966	0.017031913
CLTA	0.366141983	0.017091708
PGLS	0.365941429	0.01715709
NDUFB11	0.365815267	0.017198327
SAP30	0.3655436	0.017287409
TMEM147	0.365414373	0.017329919
TMOD3	0.365350694	0.017350899
PLEKHO1	0.365336193	0.01735568
RPLP1	0.365060907	0.017446646
CALM3	0.364711276	0.017562759
RPL14	0.364695307	0.017568078
TRIM41	0.364356536	0.017681233
BORCS6	0.364252	0.017716274
TRAC	0.364110724	0.017763723
MIEN1	0.364069728	0.017777512
MRPL55	0.363949449	0.01781802
IL4I1	0.363849394	0.017851775
SDHAF1	0.363816824	0.017862775
BLOC1S1	0.363763511	0.017880793
COA8	0.363309348	0.018034904
RPL10A	0.363279441	0.018045092
MRPL41	0.362949746	0.01815772
CYTOR	0.362463976	0.018324745
RPL23	0.362425295	0.0183381
YIF1B	0.362107497	0.018448135
MTATP6P1	0.361970516	0.018495735

PAQR7	0.36191958	0.018513462
RPS29	0.36147352	0.018669307
MIER1	0.36144259	0.018680154
TNIP1	0.361386312	0.018699904
UBXN1	0.36123057	0.018754652
TMSB10	0.361087617	0.018805023
MACO1	0.361020695	0.018828643
S100A9	0.360927929	0.018861425
BRI3	0.360874544	0.018880313
FUT11	0.360388084	0.019053154
ANKRD44-AS1	0.360369553	0.019059765
ENSG00000271109	0.360226982	0.019110687
ATP5MC1	0.360195008	0.019122123
BBC3	0.360137563	0.019142683
UQCRFS1	0.360058201	0.019171119
ANP32B	0.360047343	0.019175012
IRF2BPL	0.359879258	0.019235364
VSIR	0.359838878	0.019249887
DYNLT1	0.359579812	0.019343279
NCBP2AS2	0.359577038	0.019344281
COX5A	0.359503298	0.019370934
IGBP1	0.359439041	0.019394185
CLUAP1	0.359397475	0.019409238
RPLP2	0.359248336	0.019463329
THEM5	0.35921686	0.019474761
ACTB	0.359150542	0.019498866
MT-RNR1	0.359105258	0.01951534
CTDSP1	0.359041032	0.019538725
RPS19BP1	0.358741814	0.019647983
APOBR	0.358660182	0.01967788
POLR1H	0.358586102	0.019705044
CD74	0.358560217	0.019714543
DNAJC5	0.358517806	0.019730115
RPS15P4	0.358511786	0.019732326

COPS9	0.358409219	0.019770031
KIF21B	0.358272114	0.019820528
RPS2	0.358258258	0.019825637
RPL24	0.357752932	0.020012727
CCDC124	0.357469515	0.020118306
MRPL52	0.357461043	0.020121469
CAMP	0.357422417	0.020135896
PSMB10	0.357379468	0.020151948
HLA-DRB1	0.357055032	0.020273551
SQLE	0.357032905	0.020281866
RPL6	0.356624287	0.02043595
SRI	0.355898922	0.020711893
AKR1A1	0.35550453	0.020863234
SEM1	0.355502117	0.020864163
PSMB2	0.355394801	0.020905505
RPL13	0.355137716	0.021004823
ENSG00000233461	0.354940067	0.021081448
GLRX	0.354783078	0.021142477
PSMC5	0.354713177	0.021169698
RABL6	0.354700933	0.021174469
APOBEC3C	0.354665164	0.021188413
NDUFA13	0.354625028	0.021204067
CMTM2	0.354508016	0.021249763
MYL6	0.354283963	0.021337489
FKBP8	0.35417411	0.021380612
RPL27A	0.354152972	0.021388919
MARCKS	0.35411706	0.021403036
ZFP36L2	0.354009033	0.021445551
DMAC1	0.353935047	0.02147471
CPNE3	0.353931591	0.021476073
RPL28	0.353356417	0.021703899
MSRB1	0.353004986	0.021844095
PGAP1	0.352607542	0.022003558
METRN	0.352178671	0.022176722

PCBP2	0.352033359	0.022235652
ENSG00000268205	0.352006286	0.022246646
TTF1	0.351936746	0.022274905
RPSA	0.351772638	0.022341713
MT-ND3	0.351737125	0.022356192
RPL32P32	0.351431387	0.022481173
DNAJC8	0.351243478	0.022558276
RRBP1	0.351041054	0.022641583
DTX2	0.350866356	0.022713686
NENF	0.350322995	0.022939173
RETN	0.350295924	0.022950456
NDUFB2	0.350246113	0.022971228
MOSMO	0.349954519	0.023093147
CRIP1	0.349731036	0.023186953
TMEM86B	0.349696446	0.023201501
ZCCHC17	0.349645128	0.023223097
RPL26L1	0.349601555	0.023241448
C1orf56	0.349550472	0.023262976
NKG7	0.349341896	0.023351052
TUSC2	0.34930206	0.023367905
EEF1B2	0.349202226	0.023410187
SERF2	0.349200644	0.023410857
ANAPC11	0.348989279	0.023500586
ZNHIT1	0.348652412	0.023644186
BAX	0.348369796	0.023765224
RPL37A	0.348365573	0.023767037
RPS2P5	0.347967415	0.023938447
ASB16-AS1	0.347909286	0.023963558
MT-ND6	0.347644657	0.024078152
SAC3D1	0.347622292	0.024087858
RPL7A	0.347350103	0.024206242
ECH1	0.347259965	0.024245553
CUTA	0.34716842	0.024285532
ETHE1	0.347042617	0.024340561

TBCA	0.346991465	0.024362966
MRTO4	0.346486538	0.024585051
SIGIRR	0.346478595	0.024588558
ADCK5	0.346408567	0.024619495
ORMDL2	0.346174727	0.024723036
EID1	0.345978797	0.024810071
MRPL51	0.345815876	0.024882637
POLR3K	0.345807773	0.024886251
SNRPE	0.345634374	0.024963687
BST2	0.345495156	0.025026004
GNG5	0.345418387	0.025060422
NDUFS8	0.345143888	0.025183815
YY1	0.345033886	0.025233404
ENSG00000266469	0.344711782	0.025379078
TWF2	0.344698146	0.025385261
MT-TL1	0.344629916	0.025416214
CLTB	0.344375895	0.025531731
LSM1	0.344273319	0.025578501
ABRACL	0.343942375	0.025729883
CMIP	0.343939784	0.025731071
PPP2R5A	0.343628507	0.025874142
FTL	0.343588274	0.025892683
RPS5	0.343530186	0.025919471
DPM3	0.343348006	0.026003634
PPP4R2	0.343287053	0.026031844
PSAP	0.342603792	0.026349809
LAMTOR1	0.34255491	0.026372681
NDUFA11	0.3424785	0.026408465
METTL26	0.34178864	0.026733368
RPS27A	0.341683383	0.026783231
UQCRQ	0.341633639	0.026806824
CDC25B	0.341615579	0.026815393
SLC15A3	0.34145959	0.026889506
LRRC45	0.341280998	0.026974567

MRPL20	0.341205791	0.027010454
ADIPOR2	0.341088485	0.027066508
MEPCE	0.340924314	0.027145118
LINC02693	0.340681058	0.027261946
RPL23A	0.340653864	0.027275032
BMF	0.340551615	0.027324283
UPP1	0.340140579	0.027523014
TIMM44	0.340099888	0.027542752
MRPL24	0.340006538	0.027588079
C8orf76	0.339401951	0.027883144
PMM1	0.339293673	0.027936264
RPS16	0.339213652	0.027975575
C8orf58	0.33913018	0.02801663
ECE2	0.339022967	0.028069435
S100A8	0.33884243	0.028158541
PTMA	0.338578323	0.028289316
TNFSF12	0.338565952	0.028295454
NUDT14	0.338558018	0.028299391
NOSIP	0.338469076	0.028343558
ANAPC15	0.338365041	0.028395292
PPIG	0.338256612	0.028449294
IFFO2	0.33820322	0.028475917
TMEM160	0.338190364	0.02848233
IRF2BP1	0.3379216	0.028616682
KDELR1	0.337486441	0.028835324
RALBP1	0.337191434	0.028984334
TMED9	0.336931952	0.029115926
CDIP1	0.336826618	0.029169485
NICOL1	0.336573367	0.02929859
TSR2	0.336530963	0.029320254
NME3	0.336450262	0.029361519
CD3G	0.336349006	0.029413362
HEIH	0.336303267	0.029436805
FBL	0.336268435	0.029454669

NDUFS7	0.336155553	0.029512621
ENC1	0.336068523	0.029557366
CDKN2A	0.336058207	0.029562673
SET	0.335936214	0.029625497
COPZ1	0.33537033	0.029918361
ISG15	0.335250779	0.029980538
RPL39L	0.335174981	0.030020015
PPM1N	0.335112681	0.030052494
H2AC25	0.335000519	0.030111041
LRRC37A16P	0.33499761	0.030112561
NUDT16	0.334737418	0.030248746
CD3E	0.334705308	0.030265588
HMGB2	0.334573883	0.030334601
SIVA1	0.334435585	0.030407364
SSNA1	0.334322034	0.030467214
NAP1L1	0.33431436	0.030471262
RPL15	0.334235702	0.030512783
TUBG1	0.334212329	0.030525129
BTF3	0.333887182	0.030697316
MRPS28	0.333451307	0.030929397
SPECC1L	0.333121064	0.031106198
POLR2I	0.332985498	0.031179016
NCF1B	0.332930812	0.03120843
TBL1XR1	0.332693615	0.031336277
CFDP1	0.332678789	0.031344282
SPECC1	0.332675946	0.031345818
HLA-DPB1	0.332510212	0.031435425
GADD45A	0.332446727	0.031469805
YWHAG	0.332362472	0.031515482
CRIP2	0.331841454	0.031799153
BAD	0.331835148	0.031802599
MAP7D1	0.331815419	0.031813382
ARPC2	0.331810541	0.03181605
FOXN2	0.33167803	0.031888562

PWWP2B	0.331540174	0.031964144
C1orf122	0.331524635	0.031972673
BRICD5	0.331447689	0.032014934
MAP3K11	0.331402279	0.032039896
SSRP1	0.331027152	0.032246718
ERCC1	0.330979695	0.032272961
TXN2	0.330910325	0.032311353
ENSG00000261596	0.330798774	0.032373169
MAPKAPK2	0.330790245	0.032377899
ANXA11	0.330679925	0.032439136
IL7R	0.330517953	0.032529217
SOX13	0.330383806	0.03260398
RPL13P12	0.330247256	0.032680226
TGFB1	0.329917059	0.032865208
PCBD2	0.329843922	0.032906297
TIMP2	0.329802977	0.032929319
IGFLR1	0.329712512	0.032980231
REX1BD	0.329572222	0.033059311
RAC1	0.329544631	0.033074882
MCEMP1	0.329526426	0.03308516
RHOA	0.32914412	0.033301595
ZNF524	0.328642699	0.033587229
HSD17B10	0.328611357	0.03360515
ATP5F1D	0.32855598	0.033636833
DEFA3	0.328520429	0.033657185
DUSP16	0.328343637	0.033758547
SLC27A5	0.328224664	0.033826901
CACNA1I	0.328018998	0.03394533
DNAJB2	0.327752998	0.034099007
CCDC167	0.32757155	0.034204163
SNHG29	0.327378843	0.034316136
COMMD6	0.32734886	0.034333585
JUND	0.327343352	0.034336791
KLF13	0.327277124	0.034375362

ENSG00000260997	0.32722017	0.03440856
TFDP2	0.327174237	0.034435353
SMDT1	0.327158609	0.034444473
RPL22L1	0.326452092	0.034858843
RPS7	0.326198611	0.035008504
PRR13	0.32615068	0.035036862
NDUFAF1	0.326052137	0.035095226
STXBP2	0.326007713	0.035121562
PEBP1	0.325896519	0.035187554
DCAF7	0.325636906	0.035342026
ENSG00000215447	0.325530785	0.035405329
TSTD1	0.325387821	0.035490757
SUMO2	0.325353278	0.035511424
RPS21	0.324973677	0.035739183
CFP	0.324822743	0.035830075
TRAIP	0.324775575	0.035858519
TIMM13	0.324603983	0.035962149
NOL7	0.324486898	0.036033001
CCS	0.324408956	0.03608023
ABCF1	0.324207982	0.036202245
LSM3	0.323707771	0.036507399
TMEM107	0.323625442	0.036557826
HBQ1	0.323517353	0.036624117
PRPF6	0.323390804	0.036701855
CCR5	0.323051169	0.036911158
PPARA	0.322996084	0.036945196
SOCS1	0.322555637	0.037218285
HNRNPA1L3	0.322449795	0.037284156
ALKBH7	0.322423529	0.037300518
CEBPD	0.321919931	0.037615353
NHERF1	0.321896164	0.037630265
UBL4A	0.321853662	0.037656944
ATP5MF	0.321549307	0.037848444
CCDC43	0.321375004	0.037958473

CD14	0.321271591	0.038023876
ENSG00000238142	0.321228518	0.038051145
HRAS	0.321121133	0.038119197
UCP2	0.321094219	0.038136269
ENSG00000256618	0.321036745	0.038172746
SNUPN	0.3208468	0.038293502
TRIM56	0.320658211	0.038413704
LIMD2	0.320447001	0.038548691
CIAO2B	0.320412751	0.038570617
TUBA1A	0.32040068	0.038578347
SPRYD3	0.320093155	0.038775706
NABP2	0.320011779	0.038828068
MRPL33	0.319801246	0.038963806
PSMB9	0.319738027	0.039004641
DNAJB1	0.31950251	0.039157076
METRNL	0.319377239	0.039238354
LINC01963	0.319114296	0.039409404
INAFM1	0.31891232	0.039541206
NAGK	0.318882586	0.03956064
SURF2	0.318823197	0.039599479
YBX1	0.318408001	0.039871879
HNRNPUL1	0.31825687	0.039971412
THOC7	0.3181579	0.040036701
SLC27A3	0.318135025	0.040051804
IFITM3	0.318050023	0.040107966
IFI27L2	0.317806773	0.040269038
CYB561	0.317806752	0.040269052
ENSG00000256020	0.317672187	0.040358384
NDUFA12	0.317634123	0.040383682
COA3	0.317612283	0.040398203
FTH1	0.317285686	0.040615863
CALHM6	0.317084611	0.040750344
CISD1	0.31707271	0.040758314
MT-RNR2	0.316918174	0.040861932

ZRSR2	0.316749336	0.040975386
ROMO1	0.316699034	0.041009236
BATF3	0.316689482	0.041015667
SNHG8	0.31663412	0.041052954
IQCE	0.31661511	0.041065763
LIPT2	0.316390718	0.041217216
SELENOW	0.316308626	0.041272736
PDAP1	0.316231938	0.041324658
SCAMP3	0.316101541	0.041413064
RTN4	0.316098454	0.04141516
TMEM104	0.315786081	0.041627577
APRT	0.315775226	0.041634975
NCF1	0.315705286	0.041682664
LINC01871	0.31570459	0.041683139
DNAL1	0.31524423	0.041998148
CDK2AP2	0.315201392	0.042027559
LINC03078	0.315175533	0.042045321
EIF4E2	0.314997546	0.042167742
NDFIP1	0.314979286	0.042180317
MT-TV	0.314869047	0.042256304
HMGA1	0.314826982	0.042285329
UNC119B	0.314766342	0.042327199
ZNF580	0.314722507	0.042357485
CSK	0.314544968	0.042480335
BBX	0.31417689	0.042735952
OSCAR	0.314146806	0.042756899
PTPRN2-AS1	0.314082622	0.042801619
REEP5	0.314056083	0.04282012
DGCR6L	0.314018924	0.042846036
MT-CO2	0.313939847	0.042901231
AHDC1	0.313915338	0.04291835
RPL9	0.313799936	0.042999028
ZYX	0.313565654	0.043163196
NSA2	0.31351241	0.043200576

RPS17	0.313267859	0.043372604
KMT2B	0.313247015	0.043387292
LDHB	0.313141635	0.043461612
ATP1B2	0.313139403	0.043463188
TRBV3-1	0.312956662	0.043592317
FAM174C	0.312586488	0.043854846
RRAGC	0.312530927	0.043894361
DGUOK	0.312372109	0.04400747
PLP2	0.3123439	0.044027586
MT1F	0.312274901	0.044076819
ENSG00000259623	0.312127254	0.04418232
HLA-DRA	0.312109318	0.04419515
OLIG1	0.311798443	0.044418008
EIF3F	0.311769504	0.0444388
NPM3	0.31148438	0.044644076
EIF3H	0.311382833	0.044717371
SNRPB2	0.311369704	0.044726855
TES	0.311114247	0.044911703
SMARCC1	0.310873197	0.045086694
CCDC12	0.310397324	0.045433778
MAP3K6	0.310324117	0.045487364
RPS27L	0.310178375	0.045594197
FYB1	0.309894108	0.045803158
RINL	0.309845879	0.045838687
TMEM216	0.309770001	0.04589463
C1orf35	0.309731578	0.04592298
SERGEF	0.309634538	0.045994641
DUT	0.309486663	0.046104018
TJAP1	0.309364996	0.046194167
ZDHHC12	0.30926191	0.046270661
DNPH1	0.309148306	0.046355077
TRBV11-2	0.308871019	0.046561647
CDV3	0.30883503	0.046588512
POLR2G	0.308672565	0.046709946

INCENP	0.308603112	0.046761937
FRG1HP	0.308478091	0.046855641
ZNF784	0.30841797	0.046900757
BBLN	0.308410421	0.046906425
RHBDD2	0.308382398	0.046927468
STAC3	0.308216867	0.047051923
RPL18AP3	0.308160601	0.047094288
ITPKB-IT1	0.308142464	0.047107951
HSP90AB1	0.308063795	0.047167249
C12orf57	0.307902048	0.047289359
LAMTOR2	0.307731144	0.04741866
RXRA	0.307472009	0.047615258
TOMM7	0.307462486	0.047622496
ILF3-DT	0.307404634	0.047666481
SLC6A8	0.307325543	0.047726669
IGKC	0.307098897	0.047899485
PCED1B-AS1	0.306943016	0.048018637
PER1	0.306923294	0.048033729
SDF2L1	0.306901279	0.04805058
TMEM42	0.306699103	0.048205558
ZNF764	0.306629569	0.048258953
STUB1	0.306496893	0.048360966
PDCD7	0.306477523	0.048375874
PAK2	0.306437595	0.048406616
GSTM4	0.306330973	0.048488786
SERPINA1	0.306303613	0.048509889
ZC3H15	0.306039466	0.048714014
ARFRP1	0.305980751	0.048759481
ELOF1	0.305888131	0.048831274
ENSG00000274922	0.30583391	0.048873341
CCDC28B	0.305610952	0.049046632
SERBP1	0.305597814	0.049056859
NDUFB6	0.305467537	0.04915836
BCORL1	0.305221808	0.049350276

UBXN6	0.305143769	0.049411351
CDC37	0.305070547	0.049468711
SEC62	0.305068579	0.049470254
DHRS1	0.305024709	0.049504648
GSDMD	0.304783919	0.049693767
SLC66A1	0.304614968	0.049826811
IRF1	0.30454189	0.049884447
MLEC	0.304391369	0.05000333
GZMM	0.304143669	0.050199463
TRMT10C	0.30398048	0.050329019
ARMC5	0.303659856	0.050584345
CHAF1A	0.303593398	0.050637399
NDUFA6	0.303476787	0.050730598
MAPKBP1	0.303398816	0.050792992
PRKAR2A	0.303335531	0.050843679
PRDX1	0.303095756	0.051036094
RPL18A	0.30300359	0.051110212
CEBPZ	0.302835019	0.051245996
CMTM7	0.302502225	0.051514916
HSPA1A	0.302453376	0.051554484
PSPH	0.302232574	0.051733643
EIF3G	0.302197158	0.051762426
VASP	0.302090485	0.051849199
FUT7	0.301926435	0.051982873
AKT1S1	0.301808443	0.052079189
CLEC10A	0.301731104	0.052142398
ARHGAP10	0.301675518	0.052187866
ATP6V0D1	0.301436686	0.05238359
NACA	0.301368605	0.05243949
DTYMK	0.301312084	0.052485935
C1QB	0.301176492	0.05259749
DDX46	0.301035136	0.052713991
ARFGAP1	0.301026736	0.05272092
LNPK	0.300979801	0.052759652

ORAI1	0.300974179	0.052764293
CASZ1	0.300902609	0.052823403
AGER	0.300900218	0.052825379
OLIG2	0.300718939	0.05297534
TPRKB	0.300600616	0.053073405
CDK12	0.300527941	0.05313371
SELENOM	0.30037788	0.053258404
PCNT	0.300123908	0.053469978
SERTAD1	0.300022346	0.053554774
ZBTB7A	0.299885501	0.053669199
AP2A2	0.299845258	0.053702887
PPP1R18	0.299648585	0.053867765
LSM7	0.299530612	0.053966861
MAL	0.299430934	0.054050704
ANKRD22	0.299402923	0.054074283
FUNDC2	0.298932092	0.054471868
TUFM	0.29892839	0.054475003
SLC2A4RG	0.29887745	0.05451816
SELENOK	0.298858868	0.05453391
RBP7	0.298847504	0.054543544
VPS28	0.298714651	0.054656272
PEA15	0.298622605	0.054734483
C1orf131	0.298561957	0.054786065
MCRIP1	0.298282785	0.055024007
C15orf39	0.298231683	0.055067651
USP48	0.298137901	0.05514782
ENSG00000280347	0.298005685	0.055261002
GYPC	0.297923759	0.055331227
TSHZ2	0.297769562	0.055463595
MT1E	0.297240188	0.05591996
S1PR2	0.297211123	0.055945104
MT-ND4	0.297088248	0.0560515
HES4	0.297047471	0.056086844
PF4	0.296649009	0.056433158

ZFP64	0.296471172	0.056588272
RNU4-62P	0.296198408	0.056826847
SARS1	0.296180177	0.056842821
UBC	0.295925589	0.057066275
RHOB	0.295883437	0.057103339
LMAN1	0.295632971	0.057323974
GTF2IRD2B	0.295549382	0.057397759
ENSG00000280194	0.295505727	0.057436325
BARD1	0.295381795	0.057545919
HLA-DQA2	0.295239353	0.057672089
ARIH2OS	0.295138702	0.057761375
IRF3	0.295092801	0.05780213
ZNF532	0.295083865	0.057810067
RAE1	0.295079653	0.057813808
VAMP5	0.294985604	0.057897398
NSRP1	0.294904472	0.057969586
ENDOG	0.294637991	0.058207193
KLHDC10	0.294587778	0.058252053
KANSL1-AS1	0.294566763	0.058270835
EFHD2	0.29422175	0.058579891
CBX3	0.294094833	0.05869391
DDRGK1	0.293971485	0.058804892
COMTD1	0.293855557	0.05890935
MTRFR	0.293699946	0.059049799
GPR157	0.2936849	0.059063393
OLA1	0.293631732	0.05911145
SREK1	0.293627228	0.059115523
TIMM22	0.293586409	0.059152441
UQCRC1	0.293542485	0.059192188
KHSRP	0.293395812	0.05932507
NUCKS1	0.293379303	0.059340041
MTLN	0.29273568	0.059926078
CHCHD10	0.292716396	0.059943708
TRBV5-1	0.292658299	0.059996845

CCNQ	0.292612418	0.060038837
BATF	0.292575013	0.060073088
ENSG00000225616	0.292127329	0.060484234
TMEM238	0.291923293	0.06067236
AAK1	0.291894436	0.060699005
KIAA0930	0.29182352	0.060764523
SNRPD3	0.291702523	0.06087644
SYNE1	0.291636913	0.060937196
NAXE	0.29147827	0.0610843
MAN1A2	0.291424288	0.06113442
NOP53	0.291154185	0.061385692
TAOK1	0.290852662	0.061667164
RNPEPL1	0.290826616	0.061691527
ICAM2	0.290723372	0.061788171
NDUFAF3	0.290620818	0.061884289
MESD	0.29054982	0.061950901
RAD21	0.29053849	0.061961537
TRAPPC3	0.290536186	0.061963699
MT-ATP8	0.290343549	0.062144754
GUK1	0.290280965	0.062203665
FAM110A	0.290058766	0.062413184
RNPS1	0.289834118	0.062625583
ENSG00000278600	0.28973844	0.062716218
ATP5IF1	0.289670128	0.062780993
TMEM9	0.289559744	0.062885775
RNASEH2C	0.289153639	0.063272467
PET100	0.28851694	0.063882531
CD81	0.288336419	0.064056348
PER2	0.28824213	0.064147284
TPGS1	0.288049555	0.064333331
GSK3A	0.287817154	0.064558423
GAMT	0.287813544	0.064561924
GSK3B	0.287701301	0.064670866
MPHOSPH8	0.287596767	0.064772457

VPS9D1	0.287480125	0.064885963
USP16	0.287344099	0.065018532
ENTREP3	0.2872659	0.065094841
DNAJC1	0.287177434	0.065181254
FKBP1A	0.287168265	0.065190216
ENSG00000236935	0.287162975	0.065195387
GADD45GIP1	0.287138179	0.065219628
TADA2A	0.287063199	0.065292973
EIF3D	0.286858172	0.065493866
S100P	0.286794312	0.065556538
NKAPP1	0.2866997	0.065649478
CD37	0.286691676	0.065657365
PI16	0.286633243	0.065714823
PFDN2	0.286577916	0.065769264
ATP2B4	0.2865664	0.065780599
FAM200B	0.286506667	0.065839423
SEPTIN11	0.286394567	0.065949929
PTRHD1	0.286366098	0.065978017
DRAP1	0.286330036	0.06601361
RPP25L	0.286294732	0.066048468
EIF3A	0.286166509	0.066175199
PIK3R3	0.286163031	0.066178639
TLR5	0.286105106	0.066235956
DYNLL2	0.285958122	0.066381571
KMT5A	0.285845906	0.066492913
FRG1	0.28565023	0.066687419
PLEKHA5	0.2855588	0.066778456
HINT2	0.285532941	0.066804223
REXO1	0.285448364	0.06688855
PPP1R12C	0.285281869	0.067054799
MZT2A	0.284996722	0.067340285
LY96	0.284823269	0.067514415
USF2	0.284333689	0.068007828
CELA1	0.284293646	0.06804831

BABAM1	0.284289675	0.068052326
MTIF3	0.28399593	0.068349891
TSC22D4	0.283941653	0.068404986
ENSG00000227615	0.283744523	0.068605383
KDM2A	0.283540679	0.068813093
RASSF3	0.283431609	0.068924436
TRBJ2-3	0.28343	0.068926079
FTSJ3	0.283264667	0.069095131
RAB5IF	0.283112059	0.069251463
C1orf162	0.282978681	0.069388325
CHD4	0.282956474	0.069411132
ZNF385A	0.282792388	0.06957984
SP110	0.28277003	0.069602853
ANXA2R	0.282692903	0.069682285
SLC25A39	0.282653938	0.069722442
ANTKMT	0.282610823	0.069766897
ARPC5	0.282258804	0.070130693
MPDU1	0.282160219	0.070232845
ARL14EP	0.281975222	0.070424851
TRIP6	0.28178026	0.070627648
ENSG00000187951	0.281491683	0.070928666
CENPU	0.28132488	0.071103122
UCHL3	0.281204773	0.071228949
MLLT1	0.281182367	0.071252441
RAB32	0.28111413	0.071324024
STX10	0.281089438	0.071349942
CCDC102B	0.281050304	0.071391031
CBX6	0.281043175	0.071398519
ADAP1	0.280802043	0.071652139
LYPD2	0.280634514	0.071828762
GLB1L3	0.280498283	0.07197264
KPTN	0.280464728	0.072008113
ZNF768	0.280252929	0.072232339
ATP13A3	0.280149568	0.072341964

CHMP4B	0.280144332	0.072347522
PRPS1	0.280110848	0.072383065
MT1X	0.279973951	0.072528525
POLR3G	0.279954073	0.072549666
PTTG1	0.27986634	0.072643028
SHISA5	0.279761821	0.072754379
MT-CO3	0.279730568	0.0727877
MRPL14	0.279616979	0.072908909
PDIA5	0.279407237	0.073133138
SDHAF4	0.27934722	0.0731974
AOC1	0.279249307	0.073302334
IFTAP	0.279187719	0.0733684
HTATSF1	0.279164046	0.073393806
SLC25A1	0.27913905	0.073420639
MRPL28	0.279120232	0.073440845
PHF1	0.279040398	0.073526619
NEXN	0.278804715	0.073780296
B3GNT8	0.278789742	0.073796436
NUDT1	0.278787357	0.073799007
TUBA4A	0.278743725	0.073846055
PFN2	0.27871703	0.073874852
SPN	0.278582666	0.074019928
TMEM150A	0.278486586	0.074123805
CAMLG	0.277883553	0.074778396
SMIM7	0.277767704	0.074904668
TNFRSF18	0.277661246	0.075020852
UBL7	0.277408422	0.075297341
NUDC	0.277308343	0.075407009
AIP	0.276859693	0.075900185
MRPL11	0.276600164	0.076186626
SEMA4D	0.27655819	0.076233031
ZNF835	0.276517401	0.076278148
MRPS15	0.276472135	0.076328242
EIF6	0.276450789	0.076351873

SLC7A6OS	0.276389987	0.076419217
CHMP6	0.276374393	0.076436496
UQCC3	0.276152225	0.076683006
ECI1	0.276076889	0.076766737
SUB1	0.276010495	0.076840589
NCLN	0.275774944	0.077103051
MT-ATP6	0.275719178	0.077165291
ARRB2	0.275426169	0.077492962
TRBV18	0.275400949	0.077521216
HPF1	0.275390288	0.077533162
BSG	0.275324441	0.077606978
MT-ND4L	0.275055479	0.077909064
BANF1	0.275017356	0.077951956
ZNF706	0.274958881	0.078017783
MEOX1	0.274905122	0.078078339
U2AF2	0.274801388	0.078195292
QTRT1	0.274783444	0.078215536
C19orf25	0.274739593	0.078265026
SFMBT2	0.274726258	0.078280082
MALSU1	0.274723062	0.07828369
TNK2	0.2745703	0.078456322
EIF5A	0.274559408	0.078468641
ATP1A1	0.274479029	0.078559606
UFC1	0.274302651	0.078759501
HEBP2	0.274191463	0.07888572
PLXNA4	0.274168027	0.078912343
CITED4	0.273995377	0.079108697
MANBAL	0.27399337	0.079110982
MAF1	0.273922962	0.079191168
ARHGAP30	0.273870539	0.079250912
GIPC1	0.273635674	0.079519011
AP3S2	0.273612334	0.079545693
SRGN	0.273555494	0.079610699
PRKDC	0.273547591	0.07961974

PQBP1	0.273486347	0.079689837
NANS	0.273467429	0.079711499
EIF5	0.273268985	0.079939005
MGST2	0.27319727	0.080021348
PRRC2C	0.273170612	0.080051973
RAP1A	0.273104329	0.08012816
TRMT12	0.272921085	0.08033908
BLVRB	0.272909168	0.080352812
MDH2	0.272678982	0.080618414
HSPE1	0.272467999	0.08086246
MNAT1	0.272382124	0.080961957
RALA	0.272304345	0.081052157
SKI	0.27204292	0.081355904
BCL9L	0.271993031	0.08141397
TEDC1	0.271983784	0.081424737
SNHG6	0.271849288	0.081581455
EMG1	0.271845592	0.081585765
CDIPT	0.271794924	0.081644869
NLK	0.271787334	0.081653725
SND1-IT1	0.271764584	0.081680276
SMIM24	0.271756157	0.081690113
RANBP2	0.271470861	0.082023674
ORAI3	0.27145883	0.082037764
ZNF525	0.271431535	0.082069736
CENPX	0.271322548	0.082197497
CCDC85B	0.271121698	0.082433351
MFSD12	0.271118249	0.082437406
POLR3GL	0.270771754	0.082845543
PARVG	0.270677008	0.082957419
PHF5A	0.270659632	0.08297795
ISOC2	0.27065706	0.082980988
NECAP2	0.27065126	0.082987842
CERS4	0.270624779	0.083019141
B9D2	0.270615595	0.083029998

ZSCAN16-AS1	0.270570196	0.083083682
TMEM109	0.270566966	0.083087504
ZNF57	0.270552378	0.08310476
ARL2	0.270155379	0.083575465
ZNF702P	0.269876189	0.08390773
LSM10	0.269716747	0.084097944
SMPD3	0.269615107	0.084219375
ARL13B	0.269409223	0.084465767
MMP9	0.26938001	0.084500773
ZC3H4	0.269270531	0.084632063
MEI1	0.269265772	0.084637774
RNF169	0.269009952	0.084945193
BLVRA	0.268952067	0.085014874
ABCA2	0.268835055	0.085155866
SLC22A18	0.268832166	0.08515935
SCO1	0.268805264	0.085191792
ENSG00000269958	0.26875005	0.085258407
TATDN2	0.2683367	0.085758393
CD48	0.268285492	0.085820493
EIF5B	0.268165483	0.085966163
ENSG00000273855	0.2681503	0.085984606
GSTK1	0.268025127	0.086136775
SF1	0.267997852	0.086169959
KIF1C	0.267802392	0.086408064
ISCA2	0.267469336	0.086814959
HS2ST1	0.267386961	0.086915827
ARHGDIA	0.267351046	0.086959833
MRPL10	0.267284236	0.08704174
ZDHHC20	0.26718395	0.0871648
IL2RA	0.267101112	0.08726655
EMC3	0.267013014	0.087374865
SRPK2	0.266898485	0.087515829
SMKR1	0.266895539	0.087519458
TKT	0.26679355	0.087645141

DHRS4L2	0.266784345	0.087656491
TSPAN16	0.266781886	0.087659524
IGFBP2	0.26670097	0.08775935
LAIR2	0.26664767	0.087825156
IGHGP	0.266639729	0.087834963
NIPBL	0.266480308	0.088032033
SNRPD1	0.266328282	0.088220279
PLEKHM2	0.266289631	0.088268188
LAGE3	0.26625114	0.08831592
ENSG00000269246	0.266220869	0.088353471
PTPA	0.266109607	0.088491602
ZFAS1	0.266105139	0.088497152
CHCHD5	0.266058612	0.088554967
EGLN2	0.265928156	0.088717228
KCNMB1	0.265890187	0.088764498
TNFSF10	0.265889192	0.088765738
DCTN2	0.265791471	0.088887485
ENSG00000260592	0.265743334	0.088947506
ANAPC16	0.265527553	0.089216942
SPIN2B	0.265523314	0.089222241
MISP3	0.265405093	0.089370134
MYOF	0.265404205	0.089371246
SNHG32	0.265252925	0.089560771
RNF145	0.264980496	0.089902861
CCDC22	0.26496526	0.089922023
SLA	0.264950184	0.089940986
RNF187	0.26490256	0.090000911
ENSG00000258843	0.264854533	0.090061375
SIRT4	0.264852163	0.090064359
ABHD17A	0.264822346	0.090101914
ERGIC3	0.264701795	0.090253872
MAP3K4	0.264545149	0.090451626
MFHAS1	0.264536552	0.090462489
FOXN3-AS1	0.264469612	0.090547106

LINC02361	0.264427562	0.090600291
SQOR	0.264366896	0.090677065
MAD1L1	0.264350162	0.09069825
MBD3	0.264284606	0.090781283
GPI	0.26415971	0.09093964
GPKOW	0.264073778	0.091048719
ZCRB1	0.264033873	0.091099407
NDUFAB1	0.263902787	0.091266068
ARID3A	0.263889519	0.09128295
MCRIP2	0.263604822	0.091645773
FBXW4	0.263205752	0.092156231
CMSS1	0.263151936	0.092225235
ANKRD16	0.263116749	0.092270375
GTSF1	0.263103384	0.092287525
UBXN4	0.263070146	0.092330186
SLC4A8	0.263037699	0.092371846
RAP2A	0.263032779	0.092378165
MSRB2	0.262846992	0.092616999
MRPS21	0.262750957	0.092740642
MRPS7	0.262682059	0.092829425
SPATA2L	0.262680772	0.092831083
MAD2L2	0.262648325	0.092872919
SMUG1	0.262560328	0.09298645
MRPS16	0.262465661	0.093108706
FNBP1	0.262396642	0.093197916
HIRIP3	0.262362073	0.093242623
TBC1D10B	0.262180699	0.093477462
HLA-B	0.262177668	0.093481391
H1-10	0.262129318	0.093544072
NDUFB4	0.262100869	0.093580968
ADD1	0.261799426	0.093972612
RBM42	0.261607734	0.094222321
ENSA	0.261414509	0.094474544
HNRNPA1	0.261386967	0.094510538

SPRY1	0.261309142	0.094612303
ERH	0.261184449	0.094775528
TOX2	0.261128131	0.09484932
DNAJC9	0.261000617	0.095016563
UBE2L3	0.26036933	0.095847885
PRDX4	0.260041182	0.09628222
CSNK1G1	0.259991753	0.096347774
KCNAB2	0.259930835	0.096428614
ZFP36L1	0.259913736	0.096451314
LINC00467	0.259892693	0.096479255
MXD4	0.259864251	0.096517032
FBXL8	0.259746224	0.096673915
COPRS	0.259709532	0.096722726
TIAL1	0.259683538	0.096757319
MIB1	0.259655903	0.096794104
MICOS13	0.259478181	0.09703093
CANT1	0.259474495	0.097035847
ENSG00000279361	0.259406668	0.097126352
FEM1B	0.259373322	0.097170871
NCL	0.259314246	0.097249779
UBE2L6	0.259170985	0.097441341
CCDC15	0.259149328	0.097470324
CDT1	0.258940497	0.097750146
AGFG2	0.258788421	0.097954306
DSTN	0.258733322	0.098028357
PTPN23	0.258729442	0.098033573
NME2	0.258608496	0.098196277
MRPL21	0.258567757	0.098251128
FBXW9	0.258483879	0.098364136
HAAO	0.258249898	0.098679904
POLR1E	0.257937759	0.099102362
PGLYRP1	0.257915135	0.099133035
PRR14	0.257796585	0.099293884
GPR137	0.25773371	0.099379276

BRI3BP	0.257687942	0.099441468
RUNX1	0.257679681	0.099452698
PCNX2	0.257602527	0.099557617
SSU72	0.257524662	0.09966359
ENSG00000274275	0.257362599	0.099884431
ELF1	0.257137752	0.100191449
DHRSX	0.257136699	0.100192889
PDPR	0.257016804	0.100356898
MANF	0.256957546	0.100438034
MORF4L1	0.256943075	0.100457856
TAX1BP3	0.256870542	0.100557252
ENSG00000255320	0.256748814	0.100724234
NACA3P	0.25655593	0.100989259
NMRAL1	0.256439145	0.101149984
MYDGF	0.256334874	0.10129365
MT-ND1	0.256286387	0.10136051
HBA2	0.256184648	0.101500911
BAIAP2-DT	0.256183413	0.101502616
ARMC7	0.256180951	0.101506015
MRPS5	0.256063682	0.101668038
ANP32E	0.255705243	0.102164496
BRMS1	0.255668823	0.102215044
MOB2	0.255571489	0.102350227
EEF1DP7	0.255535319	0.102400498
RANBP9	0.255505228	0.102442333
GGNBP2	0.255495884	0.102455326
CDC26	0.255287003	0.102746125
MARCKSL1	0.255262363	0.102780469
HMOX2	0.25516997	0.102909331
GINS2	0.255165045	0.102916203
SMPD1	0.255144261	0.102945209
PNMA3	0.254279458	0.104157677
GEMIN7	0.254214324	0.104249435
PLEKHA2	0.254004088	0.104546031

TUT4	0.253962687	0.104604514
RHOC	0.253855746	0.104755696
AGAP2	0.253812815	0.104816434
PRRC2A	0.253783464	0.104857974
ERICH6-AS1	0.253678223	0.105007025
HCFC1	0.253647976	0.105049894
RUNX3	0.253583112	0.105141869
CEP135	0.25339997	0.105401892
AHSP	0.253390565	0.105415258
CCDC59	0.253028332	0.105931046
SNX17	0.252904005	0.10610852
CENPH	0.252860908	0.106170092
PPP1R16A	0.252843855	0.106194464
SNRPB	0.252818824	0.106230244
RNF5	0.25275373	0.106323336
LDAF1	0.252732115	0.106354262
PYM1	0.252510001	0.106672449
KATNB1	0.252508134	0.106675127
POGZ	0.252369877	0.106873556
ILRUN-AS1	0.25224916	0.10704704
WAS	0.252181651	0.107144153
KMT2A	0.251965349	0.107455756
MRPS34	0.25195956	0.107464106
H1-4	0.251765999	0.10774355
FEZ2	0.251739247	0.107782216
DMTN	0.25173397	0.107789844
ENSG00000213058	0.251594866	0.107991076
L3MBTL2	0.251550213	0.108055733
USE1	0.251489641	0.108143488
ARF1	0.25148694	0.108147402
CEP126	0.251426266	0.108235361
RAB31	0.251328794	0.108376781
SEPTIN6	0.251242126	0.108502643
BHLHE41	0.251107336	0.10869861

PRDM10	0.251050803	0.108780883
LRPAP1	0.251030406	0.108810577
HLA-G	0.250784035	0.109169747
ENSG00000237550	0.250732535	0.10924494
PRR5L	0.250721137	0.109261586
NUCB1	0.250676199	0.109327238
CD300C	0.250602671	0.109434722
CKAP2	0.250559608	0.109497709
CCDC25	0.250532308	0.109537655
ILRUN	0.250478548	0.109616348
ZNF284	0.250367099	0.109779626
PHF19	0.250306343	0.109868713
UBE2B	0.250178614	0.110056183
ZNF688	0.250170485	0.110068122
LYZ	0.250094287	0.110180084
HLA-DRB5	0.249847723	0.110542968
MRPS33	0.249783709	0.11063733
TMEM191A	0.249678183	0.110793017
WDR43	0.249605409	0.110900481
ENSG00000258682	0.249445073	0.111137524
PDCD6	0.249422879	0.111170367
TOB2	0.249366245	0.111254206
PHB2	0.249296503	0.111357516
HPS1	0.249123522	0.111614071
ZBTB8OS	0.248995833	0.111803738
IGFBP3	0.248976764	0.111832084
LSR	0.2489561	0.111862807
TMEM179B	0.248928876	0.111903294
MAN1B1-DT	0.248717391	0.112218181
SPATS2	0.248549221	0.112469054
HEBP1	0.248501568	0.112540218
FBXO48	0.248497659	0.112546058
NDUFV3	0.248423069	0.112657524
RNASE2	0.248355745	0.112758205

NR2F6	0.24826698	0.112891052
UTP3	0.248240406	0.112930847
BABAM2	0.248200671	0.112990369
AGAP7P	0.248195664	0.112997872
MGAT1	0.248090506	0.11315552
UROD	0.247964154	0.113345163
SPATA6	0.247931608	0.113394049
C11orf68	0.24784372	0.113526147
CENPB	0.247704491	0.113735648
PANX2	0.2476893	0.113758523
TAF1	0.247612617	0.113874053
TMEM183A	0.247394227	0.114203562
MCF2L	0.247337165	0.114289777
PTMS	0.247301425	0.114343801
HDGF	0.24702258	0.114765966
MICALL1	0.246988621	0.114817461
TRAPPC6A	0.2469818	0.114827805
ZNF106	0.246909286	0.114937829
HTT	0.246699291	0.115256895
ASCL2	0.246634058	0.115356146
BORCS7	0.246433873	0.11566113
MPST	0.246431824	0.115664255
CCL23	0.246386363	0.1157336
MTHFD2	0.246350333	0.115788582
HNRNPUL2	0.246271923	0.115908308
ZC3H13	0.24612116	0.116138769
MAP4	0.246079686	0.116202228
PURB	0.246075376	0.116208825
TRAPPC4	0.246026023	0.116284376
SLC39A8	0.245915512	0.116453689
SEPTIN9	0.245857899	0.116542031
ENSG00000227775	0.245743367	0.116717799
CNTLN	0.245709299	0.11677012
TESC	0.245683753	0.116809366

PRSS33	0.245649178	0.116862498
RPL22	0.245579943	0.116968948
FBXL15	0.245519018	0.117062681
RETREG2	0.245503164	0.117087082
SNRPG	0.245399655	0.117246487
LTBP3	0.245384959	0.117269131
IRS2	0.245266628	0.117451589
CHD3	0.245266432	0.117451892
ARL4C	0.245072184	0.117751873
GLCE	0.245033893	0.117811074
NUP214	0.244898697	0.118020281
CD320	0.244886139	0.118039727
GOLGA3	0.244875161	0.11805673
HLA-H	0.244757794	0.118238616
SETD1B	0.244740498	0.118265439
TCF7L2	0.244540209	0.118576372
CISD3	0.244402734	0.118790149
FBXL20	0.244351221	0.118870328
PPBP	0.244336972	0.118892513
SLC35F6	0.244294061	0.118959344
PWAR5	0.244126202	0.119221042
TXNDC9	0.243962685	0.119476389
SLC25A35	0.243944222	0.119505247
TCF25	0.243865642	0.119628125
GRIPAP1	0.243727143	0.119844933
POLD2	0.2436255	0.120004234
SKAP1	0.243328573	0.120470511
PSMC3	0.243275378	0.120554188
SREK1IP1	0.243243353	0.120604587
AJM1	0.243176326	0.120710119
CXCR3	0.242821377	0.121270135
SPAST	0.242092877	0.122425634
EEF1D	0.242059357	0.122479001
ATP6V1E1	0.241950101	0.122653063

CAPG	0.241835984	0.122835069
YPEL3	0.241709576	0.123036914
GDPD5	0.241696234	0.123058233
IMPA2	0.241585592	0.123235131
FMNL2	0.241475155	0.123411893
SLC25A29	0.24142689	0.123489202
ANG	0.241395939	0.123538799
CENPF	0.241304645	0.123685177
ENSG00000260822	0.241269109	0.12374219
CD59	0.241237671	0.123792645
SYTL3	0.241130024	0.123965522
GRK6	0.241087056	0.124034578
RPS27AP16	0.241012254	0.124154865
RPS18P9	0.240976263	0.124212772
ENSG00000240652	0.240962581	0.12423479
ITPA	0.240960329	0.124238415
UBR5	0.240945623	0.124262084
ASGR1	0.240917817	0.12430685
TRPM4	0.240899068	0.124337041
CCDC186	0.240808315	0.124483255
TMUB1	0.240803933	0.124490319
RFC1	0.24044269	0.125073634
SOWAHD	0.240377687	0.125178813
ZMAT5	0.2403655	0.12519854
CSF1	0.240307196	0.125292948
ADD2	0.240243498	0.125396153
ACSF2	0.240194893	0.125474944
FEM1C	0.240179294	0.125500239
MRPL17	0.239870742	0.126001369
RNF44	0.23985467	0.126027513
CROCCP2	0.239836555	0.126056985
PMVK	0.239724551	0.126239325
SLC3A2	0.239622905	0.126404973
SETD1A	0.239449325	0.126688226

COQ3	0.239442428	0.12669949
PNRC1	0.239427939	0.126723157
NDUFC1	0.239384495	0.126794139
UBE2Q1	0.239318664	0.126901755
ENSG00000279620	0.23894195	0.127518898
NME1	0.238842614	0.127682006
RPS27	0.238840475	0.12768552
SRGAP3	0.238724687	0.127875844
NDUFC2	0.238554846	0.128155399
PRR14L	0.238356688	0.128482141
SHISAL2A	0.238223956	0.128701349
DDX24	0.238222919	0.128703064
TCEAL4	0.238209756	0.128724818
IGHG1	0.23818921	0.12875878
KCNE1	0.238182567	0.128769762
CHEK1	0.23810606	0.128896292
CSRP1	0.238084576	0.128931839
SDF4	0.238082115	0.128935911
CHIC2	0.238060387	0.128971871
SHARPIN	0.237980801	0.129103649
SUPT5H	0.237937599	0.129175225
SERTAD2	0.237908412	0.129223598
ENSG00000267904	0.237860489	0.129303053
USP30-AS1	0.237721702	0.129533361
SART1	0.237705268	0.129560653
ATRX	0.237694711	0.129578188
ATN1	0.237661725	0.129632985
CTSW	0.237532725	0.129847451
KGD4	0.237410915	0.130050206
YIF1A	0.237370689	0.130117214
UTP14C	0.237366952	0.13012344
RPS15A	0.237325897	0.130191859
HECA	0.237321715	0.130198831
SYF2	0.237171816	0.130448878

ZSCAN29	0.237165265	0.130459813
OCIAD2	0.237133808	0.130512336
PHF23	0.237080673	0.130601089
S1PR1-DT	0.236890735	0.130918716
NAA10	0.236826323	0.13102656
ZNF582-DT	0.236718146	0.13120783
LCN2	0.236706731	0.131226969
PURA	0.236665098	0.13129679
CTSS	0.236640941	0.131337315
PARL	0.236530594	0.131522551
SLC39A7	0.236509962	0.131557206
SPTAN1	0.236432553	0.13168729
NCR3	0.236260065	0.131977499
RAB3GAP2	0.236110239	0.132229965
SGTA	0.23603018	0.132365018
TCN2	0.235915552	0.132558563
ENSG00000255310	0.235710224	0.132905779
SNAI3	0.235671282	0.132971709
SMPD2	0.235498661	0.133264248
CRELD2	0.235372847	0.133477765
PNPLA2	0.235258621	0.133671838
MT-ND2	0.235248906	0.133688354
TMEM256	0.235047968	0.134030291
CILK1	0.235008602	0.134097358
BUD31	0.234870603	0.134332656
POLR1D	0.234704996	0.134615433
ENSG00000261505	0.234642589	0.134722109
C19orf47	0.234638499	0.134729103
KIFC2	0.234435906	0.135075853
EIF3J	0.234391467	0.135152004
PXMP2	0.234323615	0.135268332
OGFR	0.234194992	0.135489057
ITGA3	0.234183669	0.1355085
ETFB	0.234159987	0.135549173

POP7	0.234022432	0.135785599
SPATA1	0.233889315	0.136014688
TCHP	0.23388362	0.136024495
MFSD13A	0.233855446	0.136073021
ARSK	0.23373227	0.136285324
PARP11-AS1	0.233576757	0.136553716
GRB10	0.233560805	0.136581268
AAMDC	0.233545899	0.136607019
MAPK3	0.233495547	0.136694027
SS18L2	0.233406656	0.136847732
MED10	0.233342308	0.136959079
DDX51	0.233270139	0.137084039
PRR5	0.233254934	0.137110377
NCAPH2	0.233019171	0.137519246
PAXX	0.232918101	0.137694802
ZNF341	0.232834747	0.137839711
TMCO1	0.232756276	0.137976235
GTF3A	0.232722945	0.138034255
PPP1R14B	0.232680272	0.138108563
EPC1	0.232546258	0.138342116
TTC3	0.232484681	0.138449529
JAKMIP2	0.232464878	0.138484084
EVI2B	0.232415289	0.138570646
ADGRE5	0.23238854	0.138617355
MYADM	0.232135661	0.139059509
STK16	0.232021105	0.139260153
HMG20B	0.231838006	0.139581292
ARHGAP11A	0.231803612	0.139641677
ACTR1B	0.231560555	0.140068961
HCFC1R1	0.231533385	0.140116784
BEX3	0.231472999	0.140223118
MRFAP1	0.231469882	0.140228608
PRUNE2	0.231358482	0.140424932
TRAV12-3	0.231293414	0.140539699

LGALS9	0.231289743	0.140546175
CTTNBP2NL	0.231288906	0.140547653
DHX29	0.231242646	0.14062929
MRPL36	0.231220374	0.140668608
MRPS12	0.231097732	0.140885258
BAZ1B	0.230988937	0.141077653
GET4	0.230959976	0.1411289
RNH1	0.230873527	0.141281958
TRBV6-1	0.230868358	0.141291114
ISYNA1	0.230769448	0.141466392
RIC8B	0.230716952	0.141559485
RAB1B	0.23065363	0.141671837
PRPF31	0.230599409	0.141768093
CDC5L	0.230569165	0.141821805
PCBP4	0.230565073	0.141829074
CCND2	0.230563747	0.141831429
EVA1B	0.230518288	0.141912194
LINC00570	0.230491708	0.141959435
TMEM53	0.230480182	0.141979923
NUTF2	0.230276858	0.142341704
CRISP3	0.230264216	0.142364221
TOMM22	0.230215039	0.142451836
PARP1	0.230210316	0.142460252
ARAP3	0.229959281	0.142908143
PSTPIP1	0.229577781	0.143590794
URI1	0.229367869	0.143967432
TOP1	0.229344355	0.144009668
PPP2R5E	0.229326643	0.144041488
ETS1	0.229321229	0.144051216
VPS29	0.229266852	0.144148943
SELENOS	0.22918542	0.144295385
EXT1	0.228760204	0.145061852
SERTAD3	0.228660687	0.145241667
ECHS1	0.22863726	0.145284021

C3orf86P	0.22862285	0.145310077
NUP50-DT	0.228611591	0.145330439
FAM177A1	0.228166185	0.146137607
AKT3	0.228087938	0.146279749
CA5B	0.227948252	0.146533749
CENPM	0.227835516	0.146738982
ENSG00000205890	0.227759979	0.146876613
SVIP	0.227737788	0.146917062
RAD23A	0.227627416	0.147118375
MED11	0.227542022	0.147274268
ENSG00000278133	0.227520171	0.147314179
PRDX2	0.227498976	0.147352899
IL18	0.227458154	0.147427494
CTCF	0.227290913	0.147733391
IL18BP	0.227179868	0.147936759
CD6	0.22712292	0.148041133
NUDT16L1	0.227028788	0.148213776
TMEM91	0.226868387	0.148508303
MMGT1	0.226828761	0.148581129
ARL3	0.226828088	0.148582367
CPSF3	0.226732104	0.148758882
HCLS1	0.226695699	0.14882587
ENSG00000258199	0.226431497	0.149312692
RPUSD3	0.226395926	0.149378325
FBXW4P1	0.226340233	0.149481129
PAFAH1B3	0.226273422	0.149604523
ENSG00000271976	0.226266333	0.149617621
LPAR2	0.226077772	0.149966301
WDTC1	0.226043461	0.150029812
RHOQ	0.225976953	0.150152977
RAB11FIP4	0.225976143	0.150154477
FAAP20	0.225935142	0.150230445
FBXL17	0.225909791	0.150277429
VSTM1	0.225733518	0.150604422

LPCAT4	0.225698603	0.150669253
BOP1	0.225441684	0.151146933
SETD9	0.225440593	0.151148964
CCT7	0.225331257	0.151352588
EAPP	0.225180283	0.151634084
ENSG00000273837	0.22499674	0.151976826
C9orf78	0.224981968	0.152004435
GBP3	0.224949531	0.152065074
STEAP3	0.224775118	0.152391428
PRSS36	0.224368811	0.153153684
BDP1	0.224367169	0.153156771
B2M	0.224275047	0.153329987
PFDN4	0.224271927	0.153335855
ZMAT2	0.224268057	0.153343137
LEPROTL1	0.224255383	0.15336698
RNASET2	0.224254111	0.153369372
LY6E	0.22424526	0.153386026
PALS2	0.224228167	0.153418189
ZNF418	0.224194876	0.153480849
SEC61A1	0.224084529	0.15368867
CD27	0.223867054	0.154098854
NASP	0.223524426	0.154746715
ATP6V1D	0.223412778	0.154958256
LLPH	0.223411591	0.154960506
MRPS10	0.223313466	0.155146601
EID2	0.223284132	0.155202263
ZDHHC12-DT	0.223187135	0.155386428
NMB	0.223153392	0.155450531
ENSG00000228427	0.222957141	0.155823744
HDAC7	0.222946583	0.15584384
ZNF578	0.222879422	0.155971722
SMG9	0.222868525	0.155992478
ESF1	0.222829267	0.156067273
CWF19L2	0.222795836	0.156130988

BAP1	0.222719443	0.15627665
CFAP36	0.222716717	0.15628185
TEX264	0.222571279	0.156559447
SDSL	0.222278917	0.157118563
IGHV1-69	0.222237604	0.157197687
TRBV10-3	0.221625158	0.158374084
ADAM8	0.221579691	0.158461672
RAB29	0.221562642	0.158494526
TMEM39B	0.221448113	0.158715346
FLNA	0.221392785	0.158822103
ENSG00000244459	0.22130891	0.158984043
DDX39A	0.221298577	0.159004001
RPS28	0.221194259	0.159205596
TYMP	0.221132896	0.159324266
UBB	0.221027251	0.159528726
NDUFV1	0.22099342	0.15959424
ENSG00000279166	0.220857067	0.159858493
NDUFB3	0.220844904	0.159882079
INSL3	0.220825276	0.159920149
IDO1	0.220787072	0.159994265
SNHG7	0.220687856	0.160186864
IPCEF1	0.220442165	0.160664526
MNT	0.220436548	0.160675459
NELFB	0.220181587	0.161172261
HAR1A	0.220071748	0.161386631
FHOD1	0.219808782	0.161900696
TIMM10	0.219765187	0.161986033
TNFRSF1B	0.219717574	0.162079272
MACROH2A2	0.219581301	0.16234635
H1-2	0.219546796	0.162414026
PDCD1	0.219545251	0.162417055
ABTB1	0.219493948	0.162517718
PITHD1	0.219489441	0.162526562
QSOX1	0.219459137	0.162586045

NHSL1-AS1	0.219206862	0.163081845
EIF4EBP1	0.21917791	0.163138815
FRG1CP	0.219162257	0.163169621
TSC22D3	0.219074246	0.163342916
RAB44	0.219058684	0.163373572
USF3	0.219041979	0.163406482
NPRL3	0.218965522	0.163557177
TBKBP1	0.218957939	0.163572129
SMIM12	0.218860609	0.163764123
GBGT1	0.218856033	0.163773153
CHMP1A	0.218815539	0.163853083
SF3B6	0.218797473	0.163888752
IRF7	0.218661563	0.16415727
ALYREF	0.218658296	0.164163729
VOPP1	0.21859075	0.164297302
SIL1	0.2184427	0.164590349
DOT1L	0.21844263	0.164590487
F2RL2	0.218293843	0.164885375
SAP30L	0.218063681	0.165342296
CD7	0.217979073	0.16551049
ELANE	0.217682363	0.16610131
PBX1	0.217627202	0.166211316
SNRPN	0.217614241	0.16623717
MAP4K3-DT	0.217606924	0.166251768
ZNF24	0.217595739	0.166274086
AGPAT1	0.217525934	0.166413409
FZR1	0.217499425	0.166466342
MRPL23	0.217482659	0.166499825
KLHL34	0.217096134	0.167273109
CSNK1G2	0.216985297	0.16749533
PAFAH1B2	0.216969428	0.167527163
FAHD1	0.216888276	0.167690023
DDX54	0.216740492	0.167986899
MED16	0.21670082	0.16806666

HKDC1	0.216669381	0.168129887
MALAT1	0.216614155	0.168240994
TAOK2	0.216586711	0.168296225
PLIN2	0.216485309	0.168500417
TRAPPC2B	0.216392653	0.168687155
LYL1	0.216270219	0.168934133
GPAA1	0.216243251	0.168988569
CCDC120	0.216191397	0.169093276
POLE2	0.216147408	0.169182135
ENSG00000275764	0.216109777	0.16925818
RRM1	0.216065785	0.169347108
HMGB1	0.21596704	0.169546844
COA4	0.215760913	0.16996433
PEX16	0.21573613	0.170014574
SPSB2	0.215652934	0.170183325
LINC00623	0.215633063	0.170223646
PERP	0.215590903	0.170309223
ADAMTSL4	0.21545866	0.170577848
FKBP3	0.215440812	0.170614125
NKX3-1	0.215349654	0.170799502
GZMH	0.215346186	0.170806557
NFKBIB	0.215092794	0.171322622
ENY2	0.215010733	0.17148999
ARPC1B	0.214940312	0.171633712
SLC30A1	0.214819266	0.171880955
STOML2	0.214795083	0.171930381
TIFAB	0.214709843	0.172104679
ZFTRAF1	0.214678329	0.172169151
ATRAID	0.214665129	0.172196161
KCTD12	0.214568175	0.172394642
TIMM8B	0.214471222	0.172593287
NPDC1	0.214435351	0.172666822
LINC02976	0.214389531	0.172760787
VPS37B	0.214316182	0.172911284

KIF21A	0.214285764	0.172973723
SPOUT1	0.214259192	0.173028282
TMSB4X	0.21418708	0.173176402
KPNB1	0.214127662	0.173298519
HTRA2	0.214042292	0.173474082
PPP1R9B	0.21392598	0.173713481
MARF1	0.213893249	0.173780893
AHNAK	0.213793005	0.173987467
HSPBP1	0.213759768	0.174056
RPS15AP10	0.213746143	0.174084097
ATAD3A	0.213598071	0.174389677
COPS6	0.213524136	0.174542403
SERINC2	0.213418276	0.174761243
DIABLO	0.213328569	0.174946845
SLC7A8	0.213303744	0.174998232
FAM50A	0.213287941	0.17503095
RSF1	0.213220877	0.175169842
NCF1C	0.213127647	0.17536306
ACAD9	0.21310332	0.175413502
THRA	0.212728803	0.17619138
SHKBP1	0.212692854	0.176266177
MCRS1	0.212606259	0.176446442
CWC15	0.212578213	0.176504854
FBP1	0.212534004	0.176596959
ADRM1	0.212493735	0.176680883
CMC2	0.21241985	0.176834941
ENSG00000279369	0.212254124	0.177180848
KANK1	0.212246057	0.177197698
TTC22	0.212144092	0.177410776
MRPS23	0.212037634	0.177633439
SSBP4	0.212022386	0.177665349
AKAP11	0.21199761	0.177717203
GSTM3	0.21173021	0.178277561
GSN	0.211593208	0.178565146

FGD4	0.211574509	0.178604425
DDTL	0.211365733	0.179043382
ENSG00000255557	0.211112238	0.179577401
ZNF621	0.210993716	0.179827472
COMMD9	0.210871836	0.180084886
RILP	0.210863887	0.180101684
ABI3	0.210850691	0.180129572
NT5C	0.210825553	0.180182708
B3GAT3	0.210807071	0.18022178
NACC2	0.210666101	0.180520006
ENSG00000272449	0.210594913	0.180670738
B4GALT1	0.210462358	0.180951651
WBP4	0.210452592	0.180972359
EP400	0.210422452	0.181036282
CYB561D2	0.210418939	0.181043732
MRPL27	0.210376883	0.181132956
SCNM1	0.21029162	0.18131394
CAPS	0.210265147	0.181370159
ENSG00000274421	0.21026459	0.181371341
IGLV4-69	0.210251537	0.181399067
GCAT	0.210193661	0.181522032
TARBP2	0.210169072	0.181574293
JUN	0.210114903	0.181689459
MTHFD1L	0.210087343	0.181748075
CCL3	0.210012898	0.181906472
HMGN5	0.209935663	0.182070908
UQCC4	0.209883679	0.182181645
ACAT1	0.209870005	0.182210781
STMP1	0.209862741	0.182226261
IDH3G	0.2097767	0.182409682
RANBP3	0.2096393	0.182702864
CDC42EP3-AS1	0.209483345	0.183036045
IGHE	0.209435756	0.183137802
EMD	0.209426727	0.183157111

PPP1R14BP3	0.209278232	0.183474908
MORN3	0.209225328	0.183588222
ZNF710-AS1	0.209173717	0.183698817
SSR1	0.209054716	0.183953998
LCK	0.209037408	0.183991132
RWDD1	0.208885158	0.184318025
FHL3	0.208858503	0.184375298
RSBN1L	0.208792269	0.184517669
GAS5	0.208729519	0.184652623
TTLL12	0.208557049	0.185023909
DDX6	0.208549487	0.185040198
LSM4	0.208512077	0.185120809
ENSG00000272369	0.208506541	0.18513274
TBL1X	0.208093071	0.186025375
SNU13	0.208022426	0.186178195
SUGP1	0.207987328	0.186254152
DCTN3	0.207984873	0.186259466
IK	0.207956532	0.186320817
TOX4	0.207939857	0.186356922
BNIP1	0.207720844	0.186831584
LFNG	0.207513462	0.18728183
SWINGN	0.207423837	0.187476651
TRPV1	0.207406778	0.18751375
MAP2K7	0.20737198	0.187589442
CDA	0.207310646	0.187722906
RNPEP	0.207297026	0.187752554
POLR3C	0.207250696	0.187853424
C6orf226	0.20715593	0.188059873
TAB1	0.206917498	0.188580011
ERF	0.206887633	0.188645233
POLR1F	0.206821098	0.188790595
TM9SF1	0.206800796	0.188834966
TAX1BP1	0.206773342	0.188894981
GAREM2	0.206761793	0.18892023

FCGBP	0.206714447	0.189023769
ZNF467	0.206678495	0.189102418
CLSPN	0.206486478	0.189522865
TTC28-AS1	0.206407263	0.189696511
KIF3C	0.206293014	0.189947152
TRBV2	0.206232108	0.190080864
INPP1	0.206204581	0.19014132
TNNT1	0.206040083	0.190502871
SNRNP27	0.206020686	0.190545535
RAB6B	0.205975531	0.190644883
ACIN1	0.205931853	0.190741018
GZMA	0.205875453	0.190865201
SLC52A2	0.205624754	0.191417893
FLYWCH2	0.205490852	0.191713559
GOLGB1	0.205467076	0.19176609
ENSG00000279996	0.205465346	0.191769913
RANBP1	0.20542614	0.191856561
ZMYND19	0.205410519	0.191891093
HRH2	0.205383836	0.191950088
ENSG00000262999	0.205337789	0.192051927
SNHG25	0.205256902	0.19223091
HGF	0.205189597	0.192379931
ACTR1A	0.205057392	0.192672883
ENSG00000275964	0.204919197	0.192979445
ADPRS	0.204857289	0.19311689
BCL2L11	0.204820927	0.193197651
PPP1R8	0.204756653	0.193340463
SMARCD3	0.204556687	0.19378525
CAMTA1	0.204497608	0.193916798
FOXK2	0.204479224	0.193957747
RIN1	0.204421781	0.194085733
ENSG00000278730	0.204354899	0.194234822
GLOD4	0.204254461	0.194458867
XIAP	0.204250706	0.194467248

NOC4L	0.204214134	0.194548876
CDK1	0.204067853	0.194875615
WHAMM	0.204024992	0.194971422
NORAD	0.20395283	0.195132806
MRPL58	0.203950619	0.195137751
FMC1	0.203926653	0.195191369
NINJ1	0.203854165	0.19535361
PAGR1	0.203793	0.195490582
COMMD4	0.203759885	0.195564767
ENSG00000224114	0.203758763	0.195567281
WWC2	0.203728748	0.19563454
PLAAT3	0.203685332	0.195731854
AFDN	0.203598043	0.195927615
CTTN-DT	0.203247719	0.196714665
ENSG00000255026	0.203193649	0.196836338
NUDCD2	0.203183808	0.196858489
FDX1	0.203166688	0.196897028
ITM2B	0.203033385	0.197197292
CENPW	0.203011356	0.197246942
PLEC	0.202981632	0.19731395
ACSS3	0.202868513	0.197569108
ATF5	0.202841782	0.197629437
RCN1	0.202832632	0.197650092
TRAPPC12	0.202686762	0.197979562
SCN9A	0.202632709	0.198101747
GTF2H5	0.202630247	0.198107313
APLP2	0.20256308	0.198259218
RNF125	0.202547935	0.198293482
RNF168	0.202495541	0.198412048
DSC2	0.202378813	0.198676381
YKT6	0.202220153	0.199036064
LDLR	0.202083923	0.199345266
THOC6	0.20203543	0.199455411
SRRM1	0.2019777	0.199586594

SRF	0.201910963	0.199738319
GOPC	0.201845842	0.199886446
GTF2F1	0.201581637	0.200488217
ANKRD11	0.201562556	0.200531727
DGKH	0.201555468	0.200547891
H2AJ	0.20150111	0.200671884
TSPAN17	0.201475888	0.200729434
VGLL4	0.201435454	0.200821719
SEC11A	0.201351587	0.201013229
LITAF	0.201286465	0.201162024
ENSG00000226471	0.201150164	0.201473702
SP1	0.201147378	0.201480074
TMEM158	0.201034725	0.20173794
PSMG4	0.200864496	0.202128034
RGS2	0.200768303	0.202348702
TMEM19	0.200765203	0.202355816
PDZD4	0.20049733	0.202971226
AGAP1	0.200311884	0.203398035
GTF3C3	0.200310504	0.203401214
EHBP1L1	0.200246503	0.203548662
EXOSC9	0.200208653	0.203635896
IFI6	0.200159939	0.20374821
CR1L	0.200046474	0.204009978
ENSG00000266088	0.200001397	0.204114036
IMPDH2	0.199872412	0.204412003
TMEM11	0.199858304	0.20444461
ELP6	0.199775561	0.204635935
NAF1	0.199624891	0.204984646
OSGEP	0.199582874	0.205081966
RPS3A	0.199483175	0.205313014
ARRB1	0.199479634	0.205321223
SEC11C	0.199425219	0.20544741
SMAD3	0.199356861	0.205606004
KDM5A	0.199057445	0.206301674

NBDY	0.199041904	0.206337829
PML	0.199016843	0.206396138
FUT4	0.198899629	0.20666901
LYPLA2	0.19871783	0.207092734
NPM1	0.198692494	0.207151835
CCSAP	0.198611638	0.207340521
F2RL1	0.198539976	0.207507852
EXPH5	0.19851828	0.207558529
GARS1	0.198480103	0.207647728
KNL1	0.198408436	0.207815243
MRPL57	0.198313205	0.208037983
KLHL25	0.198177622	0.20835539
APEX2	0.198090111	0.20856044
ATP5F1A	0.198060902	0.208628912
HLA-L	0.1975473	0.209835449
TSPYL4	0.19747819	0.20999817
PSIP1	0.197461097	0.210038429
BCL2A1	0.197348412	0.210303974
DNAJC17	0.197327027	0.210354393
TRAPPC10	0.197250392	0.210535149
PPOX	0.197068091	0.210965567
PMF1	0.197002188	0.211121316
CLPP	0.196883311	0.211402462
PUSL1	0.196688478	0.211863807
MED19	0.196660726	0.21192958
CCDC34	0.1965382	0.212220129
MTCO1P11	0.196440087	0.212452988
SLC25A11	0.19641197	0.212519753
CCR5AS	0.196355154	0.212654708
SAMD4A	0.196335785	0.212700729
ZSWIM4	0.196317928	0.212743165
MPND	0.196272752	0.212850545
NOM1	0.196225017	0.212964049
KLRB1	0.196048267	0.213384692

RPL21	0.19604641	0.213389114
NSFL1C	0.196043333	0.213396443
RRP7A	0.196038416	0.213408152
CBX1	0.195984487	0.213536622
EIF4G1	0.195891307	0.21375872
TSEN34	0.195888195	0.213766141
PABPC1P3	0.195815089	0.213940508
SEC63	0.19574844	0.214099559
ZBTB38	0.195740904	0.21411755
GPATCH4	0.195704059	0.214205518
TBC1D24	0.195702333	0.214209639
ARPC5L	0.195507533	0.21467515
CPTP	0.195442737	0.214830146
SYNGR2	0.195366385	0.215012888
SOS1	0.195361064	0.215025625
SRSF3	0.195329814	0.215100454
POP1	0.195304704	0.215160592
SPINT2	0.195289174	0.215197792
ADISSP	0.19518279	0.215452743
MSRA	0.195094739	0.215663915
ENSG00000251023	0.195092696	0.215668816
GLIPR2	0.195016008	0.215852858
PI3	0.194902122	0.216126369
AEBP2	0.194861652	0.21622362
PAK4	0.19480372	0.216362886
TAF5L	0.194779142	0.216421989
PFKL	0.194740547	0.216514822
PGD	0.194623556	0.216796391
DHX36	0.194548017	0.216978329
KNOP1	0.194544653	0.216986433
RUSC1-AS1	0.194525601	0.217032339
TRBV12-3	0.194456929	0.217197857
ANKRD39	0.194316841	0.217535778
ZNF575	0.194213641	0.217784952

PPP1R7	0.194180123	0.217865923
FAM13A	0.194156787	0.217922308
RER1	0.194074146	0.218122071
SMCO4	0.193885677	0.218578118
CCDC159	0.1937158	0.218989743
GALNT10	0.193691823	0.219047884
METTL1	0.193531314	0.219437371
C10orf88	0.193229311	0.220171503
CLN8-AS1	0.193186574	0.220275529
IKZF4	0.193181645	0.220287529
UBP1	0.193172166	0.220310605
CISD2	0.193116442	0.220446308
SINHCAF	0.19305063	0.220606651
TBC1D9	0.192918257	0.220929405
RN7SL382P	0.192916752	0.220933078
NDUFA8	0.192836879	0.221127985
SSPN	0.192826029	0.221154469
SH3KBP1	0.192821335	0.221165928
SGF29	0.192816111	0.221178684
PYCR3	0.192810971	0.221191231
ZNHIT2	0.192650605	0.221582995
KXD1	0.192625013	0.221645559
ENSG00000258365	0.192450057	0.222073592
ZNF609	0.19244552	0.2220847
VTI1B	0.19241417	0.222161462
PIP4K2B	0.192384108	0.222235087
GTF2A2	0.192297578	0.222447099
TRIP11	0.192166801	0.222767791
CCM2	0.192163916	0.22277487
B4GALT5	0.192146846	0.222816754
NCF4	0.191976436	0.223235174
MTX3	0.191954516	0.223289037
VCAN	0.191926055	0.223358982
NECAB2	0.191686748	0.223947706

UGGT2	0.191676268	0.223973514
LINC02035	0.191563777	0.224250646
RELA	0.191550497	0.224283378
CTNS	0.191488341	0.224436623
RRN3P3	0.191472848	0.22447483
GATAD2A	0.191410375	0.224628947
LDHA	0.191297728	0.224907022
SUMO3	0.191283167	0.224942982
RASGEF1A	0.191193434	0.225164687
MIR142HG	0.191180981	0.225195467
MARCHF2	0.191083653	0.225436127
CDC34	0.19093373	0.225807186
AHR	0.190909806	0.225866436
MIRLET7BHG	0.19087326	0.225956967
ENSG00000279884	0.190717667	0.226342676
TMEM41B	0.190651419	0.226507039
ERO1B	0.190623893	0.226575355
ICAM3	0.190476823	0.226940611
SEC16A	0.190450918	0.227004987
LTB4R	0.190400693	0.227129841
UBN1	0.190390463	0.227155276
RSRC2	0.190389161	0.227158514
SELENOF	0.190348941	0.227258537
MAGOH	0.190174621	0.227692407
RFNG	0.190020913	0.228075445
RPL21P44	0.190005131	0.228114799
POP5	0.189968944	0.22820505
PLCB3	0.189830452	0.228550682
BCL7C	0.189822299	0.228571042
DAXX	0.189784709	0.228664921
PGPEP1	0.189694329	0.228890753
TNFRSF4	0.189650326	0.229000756
ZBTB4	0.189611689	0.229097376
KRT10	0.189549719	0.229252403

HBG2	0.189526591	0.22931028
CD82	0.189447139	0.22950918
GATA3	0.189251375	0.229999759
SUCLG1	0.189203544	0.230119733
TFCP2	0.18913272	0.230297456
C6orf62	0.18899419	0.230645348
RIN3	0.188991471	0.230652181
RARA	0.188883126	0.230924525
ENSG00000230733	0.188827938	0.231063334
B4GALT7	0.188814114	0.231098115
ISY1	0.188772454	0.231202948
JDP2	0.188615662	0.231597786
H4C8	0.188479277	0.23194161
GRINA	0.18825779	0.232500717
ENSG00000272501	0.188225799	0.232581547
NEIL3	0.188020142	0.23310164
CUEDC2	0.187904416	0.233394652
PRKACA	0.18789042	0.233430107
HBB	0.187843107	0.233549985
BRD3	0.187808828	0.233636864
TRBV7-2	0.187775479	0.23372141
SLTM	0.187729854	0.233837109
PRDM5	0.187644399	0.234053916
NHP2	0.187634617	0.234078744
YAF2	0.187600283	0.234165897
PPARD	0.187499677	0.234421404
NUGGC	0.18746732	0.234503622
ATXN1	0.18746606	0.234506823
ACAP2	0.18743807	0.234577959
TLE5	0.187414164	0.234638729
CACNB4	0.187401477	0.234670983
MIR4435-2HG	0.187293747	0.234944992
MED18	0.187194402	0.235197866
KIF3B	0.186929816	0.235872253

R3HCC1L	0.186914896	0.23591032
N4BP1	0.186827972	0.236132185
BEX2	0.186650666	0.236585185
ZNF581	0.186455874	0.237083539
ASPM	0.186430865	0.237147573
CHP1	0.186413144	0.237192952
ZBTB42	0.186399789	0.237227156
SUCO	0.18632836	0.237410156
CDCA3	0.186177288	0.23779751
ZNF75D	0.186144344	0.237882035
NUDT5	0.186108882	0.237973045
EBAG9	0.186081235	0.238044016
MEA1	0.186016405	0.238210491
MLST8	0.185943004	0.238399071
IKZF3	0.185911217	0.238480767
SLC35C2	0.185775702	0.238829277
TCEA3	0.185706972	0.239006165
ANKRD37	0.185660631	0.239125481
SERP1	0.185623501	0.23922111
METTL16	0.185441162	0.239691105
LRRFIP2	0.185403684	0.239787787
SCARNA21	0.185282647	0.240100201
SNX10	0.185248402	0.240188641
ZNRD2	0.185126884	0.240502655
MAN1B1	0.185100304	0.240571374
APOL3	0.185023879	0.240769043
DDX18	0.185009694	0.240805745
FIBP	0.185004626	0.240818858
FAHD2A	0.184889263	0.24111749
TRBV4-1	0.184885357	0.241127605
ENSG00000268575	0.184876668	0.241150108
TK1	0.184730743	0.241528244
TRBV9	0.184629736	0.241790219
TAF1D	0.184584462	0.241907706

POC1B-AS1	0.184545826	0.242007997
WIPI2	0.184513632	0.24209159
FAHD2B	0.184480144	0.242178559
EXOC3L1	0.184449398	0.242258429
LARP1	0.18437352	0.242455612
EPRS1	0.18437059	0.242463231
SCAF1	0.184246286	0.242786501
ENSG00000279059	0.184222585	0.242848171
GABARAPL2	0.183949176	0.243560358
FAM120A	0.183905531	0.243674177
C7orf50	0.183862245	0.243787095
LINC02772	0.183598661	0.244475457
MBOAT7	0.183560741	0.244574595
ENSG00000259834	0.183555683	0.24458782
TOM1	0.183442422	0.244884103
AMMECR1L	0.183374331	0.24506234
TMEM51	0.183372778	0.245066406
APPL1	0.183318321	0.245209018
ZNF501	0.183281568	0.245305298
LINC02555	0.183266382	0.245345087
LRRC57	0.183260814	0.245359678
BAHD1	0.183191727	0.245540761
PET117	0.183179366	0.245573168
TRBV30	0.182825499	0.246502172
ZFYVE21	0.182783051	0.24661377
GAB3	0.182729813	0.246753782
BRMS1L	0.182658656	0.246941005
TRMU	0.182591346	0.247118193
CTSZ	0.1825865	0.247130955
RRP9	0.182445915	0.247501321
ATP8B1-AS1	0.182414843	0.247583231
SLC30A4	0.182410557	0.24759453
BCAT2	0.182409838	0.247596426
ZNF529-AS1	0.182369963	0.247701571

ARAP1	0.182353655	0.247744581
NR6A1	0.182353209	0.24774576
MRPL12	0.18230012	0.247885809
SFT2D2	0.182206985	0.248131635
C11orf58	0.182120926	0.24835893
SMARCD1	0.182097593	0.24842058
TMSB4XP4	0.182085619	0.24845222
KATNAL1	0.182060719	0.248518026
LINC01881	0.182006242	0.248662043
LHPP	0.181934468	0.248851872
ENSG00000279511	0.181856833	0.249057311
C9orf85	0.181853682	0.24906565
RPS27AP12	0.181831659	0.24912395
FCSK	0.181824912	0.249141812
RIPOR3	0.181752386	0.24933388
KRT1	0.181745811	0.249351296
NAA80	0.18174528	0.249352703
SNRPC	0.181575168	0.249803612
ERAL1	0.18152466	0.249937599
PTBP3	0.181520162	0.249949532
SMAGP	0.18147249	0.250076044
ATG101	0.181469624	0.250083649
BET1L	0.181411316	0.250238449
PTAR1	0.181317955	0.25048644
RPUSD4	0.181295786	0.250545349
TNRC6A	0.181249497	0.250668387
ZNF667-AS1	0.181209542	0.250774619
DBTP1	0.181188467	0.250830665
HMGN2	0.181166569	0.25088891
CINP	0.181011007	0.251302935
EPHB3	0.180971435	0.25140833
RTRAF	0.180724553	0.252066528
SIT1	0.180693409	0.252149642
SPNS1	0.180667001	0.25222013

BEX5	0.180611325	0.252368784
ENSG00000267194	0.1805174	0.252619698
FAM20B	0.180505866	0.252650521
GGT1	0.180453517	0.252790451
POR	0.180336374	0.253103767
BCL11B	0.180317777	0.253153529
PCBD1	0.180280145	0.253254251
ENSG00000241666	0.180268442	0.253285579
CCDC137	0.180191045	0.25349283
LGALS2	0.180132203	0.25365047
CORO1B	0.179995647	0.254016561
PCNX1	0.179813832	0.254504541
CDC42EP4	0.179754663	0.25466348
ZNF846	0.179740134	0.254702517
PSMG3	0.179545164	0.25522677
IGHV3-20	0.179493948	0.255364605
ZBTB44	0.179472901	0.255421262
CAMK1	0.179452921	0.255475054
CDK5	0.179425952	0.255547673
OXLD1	0.17939678	0.255626243
TAF13	0.179256486	0.25600432
MAP3K13	0.179255933	0.256005809
SPI1	0.17925093	0.256019299
FAM13A-AS1	0.179250292	0.256021018
PIP4K2A	0.179132345	0.256339177
MSL1	0.179085214	0.256466383
RTL8A	0.178850037	0.257101763
PLCXD1	0.178798707	0.25724058
ACBD3	0.178724706	0.257440796
CDCA5	0.178659571	0.257617112
ING2	0.178544546	0.257928673
TRBV6-5	0.178541344	0.25793735
CCDC170	0.178491998	0.258071093
ABHD14B	0.178460682	0.25815599

CORO7	0.178446213	0.258195222
DLGAP1-AS1	0.178309317	0.258566608
DOHH	0.178285389	0.258631559
SNRNP48	0.178267395	0.258680409
PLEKHJ1	0.17825735	0.258707681
ENSG00000280388	0.177860268	0.259787332
HSPA1L	0.177767292	0.260040563
YTHDC1	0.177642212	0.260381491
IGLV2-11	0.177638709	0.260391044
DDAH2	0.177608193	0.260474267
CTSH	0.177561749	0.260600964
INPPL1	0.177496145	0.260779999
SLC1A5	0.177443314	0.260924237
ENSG00000236540	0.177382421	0.261090549
URM1	0.177347797	0.261185149
RYBP	0.177331669	0.26122922
OTUB1	0.177235107	0.26149319
BRD9	0.177233714	0.261496999
BRD2	0.177162382	0.261692115
EPHB2	0.177161393	0.261694823
ZNF629	0.176745198	0.262835186
RASSF7	0.176735434	0.262861978
ECHDC2	0.176723696	0.262894189
SMS	0.176689846	0.262987096
VSIG2	0.176688807	0.262989949
ACRBP	0.176549969	0.263371243
STX5	0.176507339	0.263488394
C18orf54	0.176264047	0.264157633
AIMP1	0.176222583	0.264271804
CHKB-DT	0.176179894	0.264389383
ENSG00000165121	0.176146893	0.264480299
PRPH2	0.176064289	0.264707965
PPP1R3F	0.176060912	0.264717276
SLC25A37	0.176038045	0.264780323

NDUFS4	0.175942057	0.265045087
LARP7	0.175893427	0.26517929
GHRL	0.175740437	0.265601787
SNHG30	0.175708173	0.265690945
D2HGDH	0.175600378	0.265988965
TST	0.175580303	0.266044491
SAT2	0.175557995	0.266106202
TRIM28	0.175323794	0.266754649
NET1	0.175219901	0.267042637
ZCCHC3	0.175199587	0.267098972
FAM162A	0.175165777	0.267192748
PRKX	0.175029322	0.267571451
NUBP2	0.174922595	0.267867898
POMGNT2	0.174554066	0.2688932
KAT6A	0.174548766	0.268907964
SETX	0.174414169	0.269283091
DCAF8	0.174384991	0.269364457
ALG9	0.174304908	0.269587858
KCMF1	0.174057665	0.270278349
AP5S1	0.174011761	0.270406676
SLC29A1	0.173997721	0.270445933
ENSG00000269982	0.1739437	0.27059702
MED8	0.17388345	0.270765589
HLA-DQA1	0.173815222	0.270956566
NDUFS2	0.173805787	0.270982983
MYPOP	0.173757346	0.271118637
ZRANB1	0.173755801	0.271122962
ESD	0.173698962	0.271282193
TSR3	0.173637524	0.271454375
LILRA5	0.173452765	0.271972604
SLFN12	0.173400508	0.272119296
GDI1	0.173336084	0.272300217
FBXO6	0.17315797	0.272800821
OCEL1	0.173157966	0.272800831

MED26	0.173122637	0.272900197
PTOV1	0.1729191	0.273473131
NRM	0.172891379	0.273551224
ENSG00000281100	0.172754666	0.273936572
NSD3	0.172751817	0.273944607
CNFN	0.172747449	0.273956926
TMEM248	0.172607032	0.274353106
EIF3M	0.172453478	0.27478678
GTPBP6	0.172446304	0.274807052
MRPS2	0.172403908	0.274926874
POLL	0.172379092	0.274997025
ZNF703	0.172342503	0.27510048
ATXN3	0.1721954	0.275516672
PIH1D1	0.172101966	0.275781231
ARHGEF4	0.172032621	0.275977694
GABPB2	0.171971024	0.27615228
TSGA10	0.171919842	0.276297402
TAB2	0.171903066	0.276344981
TNFRSF13B	0.171898523	0.276357866
RNF207	0.17187583	0.276422235
ENSG00000283041	0.171651118	0.277060165
ACTA2	0.171636302	0.277102263
THAP12	0.171622991	0.277140083
PSMB7	0.171585617	0.277246298
EBNA1BP2	0.171542723	0.277368231
FERMT3	0.171479204	0.277548863
NECTIN1	0.171469168	0.27757741
EIF1AX	0.171452082	0.277626011
ENSG00000269044	0.17144705	0.277640329
DARS1	0.171321425	0.277997874
ENSG00000283103	0.171283996	0.278104459
PBXIP1	0.171253998	0.278189902
COX7A2L	0.170993642	0.278932205
ZNF777	0.170969142	0.279002124

ENSG00000278231	0.170958655	0.279032056
ENSG00000282980	0.170942145	0.279079181
ST20	0.170805566	0.279469236
ZNF561-AS1	0.170774516	0.279557959
KIF14	0.170767309	0.279578555
MAD2L1	0.170714589	0.279729253
BCL2L12	0.170683628	0.279817777
ECI2	0.170633865	0.279960099
ZNF277	0.170631464	0.279966966
OIP5-AS1	0.170610194	0.280027813
POPDC2	0.170444991	0.280500706
POLR3B	0.170420476	0.280570924
ARAF	0.17041899	0.280575182
LINC00892	0.170397188	0.280637637
BSPRY	0.170351034	0.280769891
CNIH1	0.170182457	0.281253279
HOOK2	0.170090461	0.281517306
KMT2D	0.170043408	0.281652408
PRPF38B	0.170012937	0.281739921
NXT1	0.170011171	0.281744993
C15orf61	0.169978621	0.281838499
CDC42BPB	0.169916406	0.28201728
SIRPA	0.16991536	0.282020285
TMEM164	0.169836129	0.282248072
ABL2	0.169808734	0.28232686
ENSG00000182584	0.16980453	0.282338951
FADS2	0.169647015	0.282792248
EIF4B	0.169630967	0.282838459
UQCC5	0.169614039	0.282887208
DGAT1	0.169594356	0.282943897
TLK2	0.169512332	0.283180216
RPL13AP5	0.169463122	0.283322057
GAB2	0.169060435	0.284484488
HNRNPD	0.169011431	0.28462616

TRAF1	0.1689599	0.284775184
ZNF419	0.168728986	0.285443602
SLCO4A1-AS2	0.168710046	0.285498472
ZC3H6	0.168650819	0.285670099
DIXDC1	0.168618783	0.28576296
ELOVL1	0.168532101	0.286014323
MMP24OS	0.168516332	0.286060064
TUBB	0.168468029	0.286200212
CNOT6	0.168374984	0.286470297
SVBP	0.168359693	0.286514699
GATA1	0.168336823	0.286581115
NEFL	0.168275978	0.286757869
ZBTB32	0.168236472	0.286872668
TFPT	0.168206198	0.286960663
PPP2R3B	0.168110388	0.287239255
ZNF644	0.16802497	0.287487781
ARSA	0.16796588	0.287659786
LRRC75B	0.167941285	0.2877314
PPIE	0.167808128	0.288119312
CBX4	0.16758857	0.288759669
PRADC1	0.167565281	0.288827647
ZNF837	0.167531231	0.288927054
ST8SIA1	0.167509511	0.288990476
LMTK2	0.167481022	0.289073677
SLC7A1	0.16746152	0.289130641
COA6-AS1	0.167321378	0.289540196
LRRC37BP1	0.16729554	0.289615748
MLLT6	0.167236217	0.28978926
SGO1	0.167146903	0.290050615
IFI35	0.167065058	0.290290249
MRPS9	0.16704959	0.290335554
FOXK1	0.16702338	0.290412328
WDR46	0.166993334	0.290500356
ENSG00000271147	0.166988642	0.290514106

TRA2A	0.166896394	0.290784487
SLC16A3	0.166887838	0.290809572
UBE2F	0.166839574	0.290951107
STX4	0.166835418	0.290963298
ZBTB34	0.16678356	0.291115426
ALDH4A1	0.166746062	0.291225461
C3orf62	0.166572165	0.291736104
RUVBL2	0.166524215	0.291877006
PLPBP	0.166476885	0.292016131
ARVCF	0.166331203	0.292444631
FLCN	0.166258733	0.29265794
POLR2H	0.166121478	0.293062215
LINC01506	0.166093453	0.293144804
BRD7	0.166085867	0.293167165
HLA-DMA	0.166053158	0.29326358
ASB2	0.165983148	0.293470018
ABCG2	0.165946432	0.293578321
PPP3R1	0.165931065	0.293623658
AZU1	0.165825535	0.293935118
SPINT1	0.165815173	0.293965712
COMMD1	0.165803977	0.293998771
H2AC20	0.165780258	0.294068813
SLC22A23	0.165778554	0.294073845
FLOT2	0.165741117	0.294184421
ENSG00000284691	0.165711862	0.294270849
PVALB	0.165407016	0.295172427
DGCR8	0.165371446	0.295277741
GSPT1	0.165352891	0.295332687
TMED3	0.165332869	0.295391985
CHST12	0.165319705	0.295430975
PASK	0.165267506	0.29558562
SLF1	0.165203343	0.295775778
SH2B2	0.165200009	0.295785661
BICRA	0.165172862	0.295866143

CCL4	0.165161153	0.295900859
BIN3	0.165102579	0.296074571
FXR1	0.164985136	0.296423063
VANGL1	0.16496709	0.296476637
ACP5	0.164783745	0.297021277
EEF1AKMT1	0.16477831	0.297037433
CLDN15	0.16476672	0.297071883
WWP1	0.16475858	0.297096081
EIF4EBP3	0.164676738	0.297339446
OTULIN	0.164663531	0.29737873
APBB1IP	0.164634449	0.297465246
RTF2	0.164602276	0.297560977
CGAS	0.1645371	0.297754967
CCDC146	0.164396999	0.298172243
CWC25	0.164345002	0.298327206
LMO7	0.164182517	0.29881178
MAML3	0.164174646	0.298835265
PPM1G	0.164085214	0.299102208
COIL	0.164022664	0.299289
IRF4	0.163953662	0.299495146
ZDHHC1	0.163855242	0.299789336
WNT10A	0.163854466	0.299791659
IGHV4-61	0.163826526	0.299875208
CMKLR1	0.163789311	0.299986518
STIMATE	0.163755328	0.300088186
TPRG1	0.163724603	0.300180124
MED13	0.163710307	0.300222908
TPPP3	0.163620667	0.300491264
CDKN1C	0.163593595	0.30057234
SMARCA4	0.163553115	0.300693599
GPBAR1	0.163496981	0.300861798
CRACR2B	0.163364321	0.301259541
TOR1AIP2	0.163296517	0.301462963
NDC80	0.163269764	0.301543249

MYL4	0.163170322	0.301841797
PAXIP1	0.163113162	0.302013491
TNFRSF14	0.163028176	0.302268882
TNPO2	0.162985368	0.302397577
CLTCL1	0.162757675	0.303082685
TPST2	0.162700271	0.303255564
PSMD8	0.162684801	0.303302165
EIF3I	0.162641596	0.303432337
SNHG15	0.16250722	0.303837426
CMTM8	0.16243911	0.304042885
ZG16B	0.162405028	0.304145726
HBA1	0.162323689	0.304391258
SPAG7	0.162267241	0.304561725
TMPRSS9	0.162078492	0.305132178
MICA	0.162021233	0.305305364
GNGT2	0.161958274	0.305495865
TNRC18	0.161949615	0.305522069
SKIL	0.161897833	0.305678816
NAPRT	0.161745924	0.306138948
SORBS3	0.161740307	0.30615597
CHM	0.161624173	0.306508049
DNAJC2	0.161572116	0.306665952
LYRM7	0.161536912	0.306772764
TBC1D10C	0.161117861	0.308046027
MIDN	0.161101371	0.3080962
RPL13P5	0.161096539	0.308110903
FCGR1A	0.16106639	0.30820265
LINC00937	0.161020662	0.308341841
AMN	0.161015758	0.308356769
TRIM52-AS1	0.16098281	0.308457086
ZNF273	0.160887311	0.308747974
R3HDM2	0.160885224	0.308754331
TOB1	0.160851694	0.308856504
CAST	0.160810877	0.308980911

IL6R	0.160786634	0.309054819
LINC01637	0.160773858	0.30909377
PLD3	0.160749878	0.309166893
URB1-AS1	0.160716837	0.309267662
PSMA4	0.160707955	0.309294752
DSTNP2	0.160703573	0.309308118
NDST1	0.160593002	0.30964552
CTSB	0.160513486	0.309888302
RGS19	0.160328446	0.310453744
RAB11B	0.160192993	0.310868071
COQ5	0.160170029	0.310938351
BRCC3	0.159817569	0.31201827
LRP10	0.159673973	0.312458919
THG1L	0.159600988	0.31268304
CDK11A	0.159554441	0.312826028
ZNF416	0.159538942	0.312873646
ENSG00000274213	0.159343676	0.313473994
FLACC1	0.159270953	0.313697768
LINC02482	0.159231048	0.3138206
BAG1	0.159206091	0.313897437
FCF1P2	0.159130472	0.314130325
SPRING1	0.159108865	0.314196885
ADPRH	0.159030682	0.314437817
PSMC4	0.15901137	0.314497346
TRAV23DV6	0.159001445	0.314527944
CDC20	0.158985315	0.314577673
SYNJ1	0.158974608	0.314610687
RNMT	0.158973285	0.314614767
DAZAP2	0.158916012	0.314791396
ERO1A	0.158895632	0.314854261
DAZAP1	0.158831154	0.315053213
SEC14L3	0.158712206	0.315420441
OPA3	0.158627056	0.315683492
MYG1	0.158589739	0.315798818

CAMKK2	0.158528907	0.315986871
ZNF428	0.158399251	0.316387921
ATP23	0.158306683	0.316674446
KPNA5	0.158296208	0.31670688
SAMM50	0.158241551	0.316876146
NETO2	0.158225276	0.316926561
PDZD8	0.158219235	0.316945273
GTF3C6	0.158178043	0.317072899
TNFRSF9	0.158131591	0.317216856
PKNOX1	0.157984324	0.317673523
DEF6	0.157825301	0.318167108
KIN	0.157603571	0.318856136
JOSD1	0.157587083	0.318907409
NTMT1	0.15754988	0.31902312
NR3C2	0.157333142	0.319697763
USP5	0.157211277	0.32007749
ATG13	0.157203603	0.320101411
FOXP4	0.157096906	0.320434121
SOCS5	0.156951045	0.320889308
PPP2R1A	0.156856229	0.321185415
PMAIP1	0.156757156	0.321495003
ANKIB1	0.156706053	0.321654765
DAAM1	0.156695657	0.32168727
TAL1	0.156464043	0.322412029
TNRC6C	0.15638614	0.322656032
SHLD2	0.156350052	0.322769103
PDRG1	0.15628278	0.322979945
LINC00324	0.156050299	0.323709248
SLC7A7	0.155998082	0.323873198
GADD45B	0.155996808	0.323877199
THNSL1	0.155985015	0.323914235
GCDH	0.155975012	0.32394565
GPAM	0.155970839	0.323958757
C6orf89	0.155857936	0.324313482

ACVR2B	0.155830557	0.324399539
ADGRG5	0.15580962	0.324465358
RTL8C	0.155693658	0.324830057
TRAV26-1	0.155663234	0.324925779
KIAA2013	0.155655777	0.324949244
EFNA4	0.155541822	0.325307961
RAPGEF1	0.155531136	0.325341614
JAZF1	0.155527711	0.325352398
PAF1	0.155523703	0.325365022
ENSG00000272669	0.155503279	0.325429347
MT-CYB	0.155427828	0.32566705
COMMD3	0.155322886	0.325997841
GGH	0.1552762	0.32614507
SPEN-AS1	0.155267505	0.326172497
PPFIBP1	0.155240874	0.326256503
POLR2E	0.15503686	0.326900504
GIMAP4	0.155022765	0.326945025
CD8B	0.155007441	0.326993436
TRIM8-DT	0.154983228	0.327069935
BTBD7	0.154974753	0.327096712
RSRC1	0.154949147	0.327177626
ADGRL1	0.15489084	0.327361925
EEF2	0.154808967	0.327620821
CENPV	0.154803124	0.327639302
DESI1	0.154754968	0.327791644
FAM98B	0.154749349	0.327809425
ATP8B3	0.154663294	0.328081779
FAM114A1	0.154540351	0.328471125
BIN1	0.15451789	0.32854229
BCL2L1	0.154507841	0.328574129
GOLIM4	0.154457689	0.32873307
ARHGAP9	0.154280794	0.329294061
SOX4	0.154220493	0.329485433
ZNF555	0.154194532	0.329567844

CHASERR	0.15417826	0.329619504
SSBP1	0.154153252	0.32969891
PDCL3	0.154135271	0.329756011
PNPLA4	0.154031134	0.33008683
CACNA2D2	0.154026809	0.330100575
IER5L	0.153979794	0.330250003
RNASE6	0.15379216	0.330846778
RPS6KA4	0.15376592	0.330930289
MED12L	0.15376164	0.330943912
KCNIP2	0.153718972	0.331079737
CCNB1IP1	0.153641327	0.331326992
CACNA1H	0.153631576	0.331358053
ANKRD12	0.153624939	0.331379195
TOMM20	0.153513465	0.331734415
HMGB3	0.153359292	0.332226087
LILRA2	0.153282119	0.33247237
PGAM1P8	0.153280542	0.332477404
CD93	0.153271308	0.332506878
REXO4	0.153249363	0.332576939
KMT2E-AS1	0.153248475	0.332579773
CDKN2D	0.153219671	0.332671741
HSPA6	0.15320641	0.332714088
DDX1	0.153105462	0.333036562
GCN1	0.153000962	0.333370585
UBAP2	0.152975611	0.333451648
NAGS	0.152959123	0.333504377
GSDMB	0.152904242	0.333679923
RPP14	0.152890212	0.333724812
C1orf159	0.152779417	0.334079416
BPI	0.152760287	0.334140667
SNX11	0.152720058	0.334269495
NDN	0.152453963	0.335122398
ZNF18	0.152373849	0.335379448
SHTN1	0.152297434	0.335624745

ENDOV	0.152203598	0.335926115
IFT22	0.152190134	0.33596937
CDCA8	0.15216686	0.336044151
NFE2L1	0.152107554	0.336234748
SPATA13	0.152046631	0.336430614
ITSN1	0.152004921	0.336564752
C22orf46P	0.151961123	0.33670564
CIMAP1B	0.151859336	0.337033204
CHN1	0.15183349	0.337116413
B3GNT9	0.151589962	0.337901036
ACBD4	0.15157487	0.337949699
ZNF787	0.151458563	0.338324863
FAAP24	0.151458075	0.338326436
YDJC	0.151413917	0.338468941
RBMS1	0.151398349	0.33851919
ABALON	0.151354295	0.338661409
RCN3	0.151280508	0.338899698
ZNF542P	0.151274135	0.338920285
SPOCK2	0.151157125	0.339298384
ZNF672	0.151112605	0.33944231
NGLY1	0.151095082	0.339498972
IFT25	0.151043445	0.339665971
PPP1R9A	0.151020239	0.339741038
DRC12	0.15090753	0.34010578
NCKIPSD	0.150890214	0.340161836
RGCC	0.150876076	0.340207611
NME4	0.15082852	0.34036161
CEBPB	0.150756874	0.340593699
EXTL3	0.150698297	0.340783524
POLD4	0.150697487	0.340786147
ITPRIPL1	0.150640453	0.340971036
NCAPH	0.150611668	0.341064374
EIF5A2	0.150384514	0.341801481
SORT1	0.150330658	0.341976385

SNX12	0.1502095	0.342370064
PIN1	0.150150407	0.342562177
PTPRC	0.150080169	0.342790609
CLIC3	0.150021847	0.342980355
ERCC6L	0.150007073	0.343028432
PRR12	0.149916245	0.343324093
XK	0.1498853	0.34342486
CLCN5	0.149853054	0.343529882
TMEM115	0.149810972	0.343666967
SELENON	0.149691406	0.34405665
PKM	0.149689678	0.344062285
TYW5	0.149542905	0.344541015
SFSWAP	0.149511693	0.344642872
CCDC107	0.149465838	0.344792549
KLHDC3	0.149453556	0.344832647
ZNF518B	0.14929328	0.345356158
FAH	0.14901919	0.346252552
TRIM5	0.148952139	0.346472052
ENSG00000267787	0.148950739	0.346476636
MT-CO1	0.148882761	0.346699263
NXPE3	0.148861754	0.346768078
LINC01215	0.148768597	0.347073343
LINC01270	0.148705773	0.347279305
SLC35C1	0.148679933	0.347364038
ENO2	0.148654002	0.347449086
UBE2E1	0.148633605	0.347515991
SYDE2	0.148610674	0.347591217
RPS2P32	0.148594336	0.34764482
RRS1	0.148347769	0.348454404
GLI4	0.148303428	0.348600116
TTC9C	0.148302713	0.348602463
BIK	0.147995829	0.349611964
AQP3	0.147966443	0.349708722
GATB	0.147934215	0.349814857

HP	0.147917801	0.349868921
SPCS2	0.147891244	0.349956403
TCEAL3	0.147856945	0.35006941
STRN	0.147824571	0.350176092
CLN6	0.147800381	0.350255819
ZBTB24	0.147747816	0.350429106
TOR4A	0.147690218	0.350619046
TNIK	0.147687077	0.350629405
NAA30	0.147590731	0.350947268
CBR1	0.147570552	0.351013863
SECTM1	0.147470239	0.351345038
MEF2C-AS1	0.147352433	0.351734206
UBQLN1-AS1	0.14719648	0.352249796
IL17RA	0.147183945	0.352291259
DENND3-AS1	0.147095459	0.352584026
CLUH	0.147070744	0.352665823
GPR82	0.147050818	0.352731781
RGS16	0.146980364	0.352965051
MRPS18A	0.14697974	0.352967118
ADCK1	0.146919332	0.353167202
SH2D2A	0.146712937	0.353851346
ENSG00000272155	0.146696105	0.353907175
MAP3K21	0.146681614	0.353955244
EXOC3	0.14654705	0.354401798
OXCT1	0.14645596	0.354704281
MRPL47	0.146432161	0.354783334
PTPDC1	0.146335306	0.355105172
IGKJ1	0.146317391	0.355164722
DENND6B	0.146309968	0.3551894
TUBB2A	0.146137705	0.355762332
CACNA1A	0.146118956	0.355824723
TRBJ2-1	0.146112927	0.355844788
EML4	0.146086129	0.355933977
ZNF264	0.146063639	0.356008842

FUOM	0.145985536	0.3562689
ENSG00000239415	0.145912595	0.356511874
CDC40	0.145893012	0.356577124
CEP85L	0.145892043	0.356580351
PKN1	0.145889858	0.356587632
SCAMP2	0.145756155	0.357033331
LYAR	0.145613642	0.357508769
PPP1R14A	0.145611221	0.357516846
ADGRE1	0.145573531	0.35764265
CNIH4	0.145486442	0.357933442
GOLM1	0.145446592	0.35806655
METTL25	0.145425095	0.358138367
EPHB6	0.145307376	0.358531795
TRBV29-1	0.145217409	0.358832651
ILF3	0.145190342	0.358923195
CUBN	0.145169783	0.358991977
PDZK1IP1	0.145106385	0.359204134
NHS	0.145092192	0.359251638
MIEF2	0.145042203	0.359418989
MICOS10	0.145036906	0.359436724
TXNDC16	0.144901235	0.359891169
CLEC4F	0.144809953	0.360197122
LINC00663	0.1447723	0.360323368
ATAD2	0.144758237	0.360370528
VASH1	0.144666448	0.360678429
RRAS	0.144656133	0.360713038
IGIP	0.144594173	0.360920982
DMPK	0.144590561	0.360933106
RPF1	0.144528324	0.361142056
PATL1	0.144520448	0.361168503
SELENOH	0.144488979	0.361274186
TRBV20-1	0.144358699	0.36171191
FDXR	0.144324576	0.36182661
SH3BP1	0.144323929	0.361828784

MRPL15	0.1442607	0.362041382
RABEPK	0.144224567	0.362162907
RDH10	0.144036961	0.362794274
ENSG00000273319	0.143951301	0.363082774
SLC50A1	0.143911445	0.363217054
PEF1	0.143830259	0.363490672
EMC8	0.143827419	0.363500246
C8orf33	0.143701085	0.363926279
E2F3	0.143697572	0.36393813
SSB	0.143657624	0.364072911
C4orf33	0.14357702	0.364344954
TBC1D31	0.143509231	0.364573839
LILRB4	0.143382945	0.365000465
NUDT18	0.14329288	0.365304914
CIRBP	0.143202075	0.365612015
EXOSC4	0.143121058	0.365886145
CAMSAP1	0.143101838	0.365951197
AKR1B1	0.143061066	0.366089214
CMTM4	0.143052219	0.366119167
FBRSL1	0.142908049	0.366607474
MON1A	0.142901296	0.366630356
MKI67	0.142870239	0.366735601
XPO6	0.142743072	0.367166732
ING5	0.142698339	0.367318462
USP22	0.142693614	0.367334491
SRRT	0.142687766	0.36735433
TOLLIP	0.142333226	0.368558293
PPIH	0.142326311	0.368581799
DUSP2	0.142303038	0.368660914
PGAP6	0.142212721	0.368968045
PRICKLE1	0.142198953	0.369014879
RTP4	0.142141565	0.369210123
ACBD5	0.142113981	0.369303993
SH3GLB2	0.142030215	0.369589137

ZSWIM6	0.141987504	0.369734581
CTSA	0.141819644	0.370306518
RRP15	0.141752103	0.370536792
FBXO30	0.141604776	0.37103939
RIMS3	0.141526353	0.371307093
SNAPC2	0.141484505	0.371449991
GLO1	0.141469534	0.37150112
CALD1	0.141374916	0.371824359
FBXO45	0.141303541	0.372068303
ENSG00000282988	0.141271291	0.37217856
ADORA3	0.141246364	0.372263793
ATP2A2	0.141196196	0.372435369
SYNCRIP	0.141116165	0.372709175
NAP1L4	0.141015555	0.373053552
SH2D1A	0.140963659	0.373231263
PCGF1	0.140861789	0.37358025
ZNF433-AS1	0.140829248	0.373691767
ENSG00000259351	0.140798736	0.373796351
UBE2D2	0.140788825	0.373830328
MAP7D3	0.140631662	0.374369328
UBE2CP5	0.140598159	0.374484289
GGA1	0.140504159	0.374806949
ETF1	0.140481219	0.374885719
ACOT4	0.140306594	0.37548564
RBM43	0.140262048	0.37563877
MARVELD1	0.140221671	0.3757776
KLHDC4	0.140185295	0.375902698
FAM89A	0.140145029	0.376041206
RAB39B	0.14010467	0.376180059
ATAD5	0.139991088	0.376571006
TRBV6-6	0.139982548	0.376600409
ATF7	0.139862951	0.377012338
ENSG00000234292	0.139772713	0.377323321
STX8	0.139765346	0.377348716

DMWD	0.139716121	0.377518429
TBC1D10A	0.139674858	0.377660726
NUDT3	0.139671802	0.377671264
TAF9	0.13963603	0.377794655
IGHV3-72	0.139581806	0.377981737
SLC43A2	0.139520026	0.378194952
GZMB	0.139386314	0.37865667
RXRB	0.139373968	0.378699318
EXOC8	0.139351321	0.378777559
ATP6V0E2-AS1	0.139345764	0.378796759
GPATCH11	0.139342887	0.378806696
DHX33	0.139321113	0.378881933
ENSG00000276136	0.139314339	0.378905344
ZNF527	0.139278319	0.379029826
TNFSF13B	0.139113912	0.379598324
EPHX1	0.139046074	0.379833045
PFDN6	0.139004388	0.379977323
MRPL37	0.1389948	0.380010511
ZNF37A	0.138941239	0.380195947
IP6K1	0.138933316	0.380223379
SLC39A3	0.138927061	0.380245041
ELP5	0.138906005	0.380317961
FLVCR1-DT	0.138795772	0.38069984
WRAP53	0.138701123	0.381027913
SLC25A28	0.138693812	0.38105326
FAM229B	0.138603173	0.381367602
GRB2	0.13854622	0.381565199
GALK1	0.138509254	0.38169348
PAIP2B	0.138240348	0.382627437
R3HDM4	0.138234879	0.382646447
PPM1K	0.138103854	0.383102019
JAGN1	0.138022018	0.383386723
SKIC8	0.137949576	0.383638852
CLCN6	0.137947134	0.383647353

ENSG00000274422	0.137947109	0.383647439
ENSG00000257176	0.137817101	0.384100172
TENT4A	0.137773317	0.384252713
SLC12A9	0.137665638	0.384628013
NLE1	0.137649759	0.384683374
IGKV4-1	0.137552607	0.385022196
USP13	0.137502938	0.38519549
CABIN1	0.137465055	0.385327692
MAP2K3	0.137429947	0.385450232
ATP6V1E2	0.137298888	0.385907886
TMEM234	0.137270506	0.386007036
UBAC1	0.137128109	0.386504716
SUPT16H	0.137055609	0.386758249
EFCAB2	0.137049872	0.386778317
TRAV9-2	0.137030549	0.386845908
WASL	0.136963932	0.387078985
TUBGCP2	0.136800745	0.387650288
LINC01765	0.136743961	0.387849202
KCTD15	0.136719482	0.387934969
GP9	0.136653292	0.388166935
PHOSPHO1	0.136420185	0.388984516
ZNF682	0.136372315	0.389152536
RPL11P3	0.136289783	0.389442318
HNRNPA3	0.136259681	0.389548039
FKBP11	0.136243339	0.389605445
FDFT1	0.136209602	0.389723964
INSR	0.136118211	0.390045133
DPM2	0.13610257	0.390100116
LENG1	0.136012229	0.39041777
ZNF213	0.135989884	0.390496363
NUDT4	0.135984497	0.390515314
SIK2	0.135975466	0.390547081
KDM7A-DT	0.13592184	0.390735749
KLHL7	0.135745146	0.391357775

ENSG00000280248	0.135732529	0.391402211
MYOM1	0.135713834	0.391468063
MUL1	0.135690537	0.391550134
IKZF1	0.135663073	0.391646896
ALAD	0.135620092	0.391798358
NCOA5	0.135547379	0.39205467
ZBTB5	0.135475194	0.392309216
MRPL4	0.135417442	0.392512936
LMLN	0.135360615	0.392713454
GPATCH3	0.135348381	0.39275663
MYOM2	0.135318163	0.392863289
USP7-AS1	0.135293258	0.392951205
PAFAH2	0.135208136	0.393251784
NFATC2IP-AS1	0.135140319	0.393491348
POP4	0.135137607	0.393500932
ZNF815P	0.135048029	0.393817504
TRBV7-9	0.134966629	0.394105304
GOSR1	0.134878943	0.394415468
STARD7-AS1	0.13470659	0.395025528
KNSTRN	0.13465492	0.395208524
TEX30	0.134631983	0.395289774
WHAMMP1	0.13442012	0.396040729
RABEP2	0.134388814	0.396151762
C12orf42	0.134332643	0.396351034
ELMO3	0.134267664	0.396581623
REEP6	0.134218851	0.396754897
MRPL1	0.134177133	0.39690302
DDX10	0.134057263	0.397328802
PHF13	0.133946229	0.397723439
PDCD11	0.133923308	0.397804934
CYC1	0.133890322	0.397922228
RP9	0.133851997	0.398058536
LINC01089	0.133828012	0.398143852
FAM234B	0.133822792	0.398162423

ZNF879	0.133806663	0.398219804
PLGRKT	0.133769227	0.398353005
NDUFB5	0.133659758	0.398742659
FAM151B	0.133503213	0.399300259
ANXA9	0.133400417	0.399666656
MOB3A	0.133336685	0.399893917
LGALS12	0.133302091	0.400017303
ZSWIM3	0.13309259	0.400765011
CMTM3	0.133091736	0.400768062
EBLN3P	0.133078389	0.400815724
GDPGP1	0.133050525	0.40091524
ENSG00000242861	0.133022764	0.401014399
PLXNB2	0.132933636	0.401332856
KMT5C	0.132827414	0.401712582
CACNG8	0.132779472	0.401884033
BFAR	0.132755599	0.401969424
DDOST	0.132712185	0.402124738
PRMT1	0.132704147	0.402153498
AMBRA1	0.132698584	0.402173402
FBXO31	0.132670359	0.402274402
FITM2	0.132644975	0.402365249
GLYCTK	0.132614959	0.402472689
CRADD	0.13256176	0.402663152
SPTBN1	0.132483334	0.402944028
DEFA4	0.132368874	0.403354154
SRSF9	0.132363231	0.403374381
TGS1	0.132335756	0.403472868
CCHCR1	0.132323895	0.403515391
ANKRD36BP2	0.132311762	0.403558889
RAD9A	0.132308453	0.403570752
VPS36	0.132213533	0.403911153
CDCA7	0.132140824	0.404172013
LINC00513	0.132139009	0.404178526
ZNF132	0.131952824	0.404846954

ENSG00000260274	0.131873206	0.40513299
AKIP1	0.131732313	0.405639441
HNRNPA2B1	0.13171817	0.405690302
ENSG00000224376	0.131649113	0.405938685
FCGR3B	0.131638663	0.405976278
KRT10-AS1	0.131579131	0.406190481
IGHJ6	0.131518457	0.406408863
ENSG00000279433	0.131469176	0.406586287
SLC26A2	0.131418476	0.406768865
IGKV3-20	0.131395799	0.406850544
DMAC2L	0.131376363	0.406920558
WDR54	0.131330992	0.40708402
ZMYND11	0.131323292	0.407111766
ENSG00000280063	0.131226242	0.407461559
MIIP	0.131199152	0.407559229
PTCRA	0.13119248	0.407583288
NTNG2	0.131186916	0.407603351
SPCS3	0.131035041	0.408151202
ATXN7L2	0.130966199	0.408399672
REM2	0.130956489	0.408434723
SPDL1	0.130869079	0.408750351
SESN3	0.130859158	0.408786185
GFUS	0.130858259	0.408789432
AKAP12	0.130842692	0.408845658
APOL1	0.130821302	0.408922926
TDG	0.130803917	0.408985734
SHROOM1	0.130718456	0.409294562
PPP5D1P	0.130659904	0.409506225
CDK19	0.13064308	0.409567056
TXLNG	0.130631075	0.409610461
TTYH3	0.130508334	0.410054436
ZNF605	0.13048649	0.410133476
ENSG00000273576	0.130485372	0.410137522
PTPMT1	0.130418362	0.410380053

BLOC1S5	0.130340012	0.410663728
ENSG00000224950	0.130251211	0.410985377
SSR3	0.130242301	0.411017658
WDR89	0.130163966	0.411301534
RHEX	0.13014772	0.41136042
DESI2	0.130141024	0.411384693
LSM2	0.130117097	0.411471431
PPP1R13B	0.13010321	0.411521781
MOB3C	0.130097823	0.411541312
DYNC1H1	0.130065705	0.411657774
TIPARP	0.130061303	0.411673735
LILRA6	0.130056105	0.411692587
WDR13	0.129937219	0.412123858
NFRKB	0.129915268	0.412203514
TCEA2	0.12990766	0.412231128
RILPL2	0.129869468	0.412369747
SLC22A1	0.129860032	0.412404
CIC	0.12985541	0.412420779
ZNF480	0.129798814	0.412626259
PES1	0.129790883	0.412655059
SEPSECS-AS1	0.12974525	0.412820787
SAYSD1	0.129718543	0.412917799
CD24	0.129667619	0.413102812
HAGH	0.129667116	0.41310464
MROH6	0.129642036	0.413195778
ZDHHC8	0.129530448	0.413601408
QSER1	0.129491737	0.413742178
KLF9	0.129438106	0.41393725
XRCC1	0.129403791	0.41406209
AFAP1L2	0.129291605	0.414470384
VSIG10	0.129237721	0.41466657
OTUD7B	0.129203757	0.414790259
NRL	0.129160528	0.414947716
HS6ST1	0.12915336	0.414973828

MIS18A	0.129134202	0.415043623
STEEP1	0.12910085	0.415165147
TYW3	0.129096622	0.415180553
ACVR1B	0.129075602	0.415257152
H3-3A	0.12896273	0.415668616
GPX1P1	0.128949454	0.415717026
AKAP8	0.128945838	0.415730212
ZNF484	0.128942596	0.415742037
IGLC3	0.128869893	0.416007216
CKLF	0.128844809	0.41609873
ENSG00000267174	0.128839414	0.416118416
ENSG00000277203	0.128837852	0.416124116
CLEC2B	0.128772413	0.416362922
PHAX	0.128709949	0.416590943
RSBN1	0.128694784	0.416646312
C1D	0.128580234	0.417064684
OSBPL8	0.12852886	0.417252398
HP1BP3	0.128516609	0.417297166
PSMG1	0.128487774	0.417402553
NKIRAS2	0.128430192	0.417613047
CFLAR-AS1	0.128398405	0.417729271
FAM168B	0.128393637	0.41774671
PNKP	0.128387162	0.417770386
KRI1	0.128252494	0.418263014
ATG2A	0.128225257	0.418362691
ZBTB45	0.128199116	0.418458366
PTDSS2	0.12815864	0.418606534
TANGO6	0.128141439	0.41866951
DBF4	0.127902353	0.4195454
PCED1A	0.127862997	0.419689679
OPLAH	0.127841122	0.419769885
PKD1	0.12782575	0.419826254
TRIB2	0.127790183	0.419956691
SPTLC2	0.127708839	0.420255093

DHRS4	0.127673021	0.420386527
NR2C2AP	0.127634081	0.420529444
ARHGAP18	0.127620054	0.420580933
WNK1	0.127529288	0.420914188
RMDN3	0.127496264	0.421035476
DPEP2	0.127495103	0.42103974
SGK3	0.12737553	0.421479062
HUWE1	0.127336839	0.421621275
THAP3	0.127282069	0.421822631
ZNF257	0.127219552	0.422052533
SAMD1	0.127211658	0.422081565
CENPK	0.127163851	0.422257428
CLCF1	0.127029759	0.422750917
SKA3	0.126911718	0.423185605
LSM6	0.126847821	0.423421012
TPP1	0.126781462	0.423665564
VWF	0.126748772	0.423786069
ENSG00000262089	0.126653633	0.424136879
SCAF11	0.126596822	0.424346441
TIGD6	0.12656897	0.4244492
ENSG00000280067	0.126550365	0.424517852
MORN2	0.126400337	0.425071671
UQCC2	0.126389412	0.425112018
APOBEC3H	0.126387908	0.425117572
RAB14	0.126356305	0.425234291
GRAMD1B	0.12630676	0.42541731
ZFPM1	0.12625818	0.42559681
KLHDC7B	0.126222926	0.425727098
LINGO3	0.126204718	0.425794398
LRIF1	0.126161014	0.425955959
ENSG00000279355	0.126148843	0.426000958
ENSG00000260257	0.126026436	0.426453672
SECISBP2L	0.125984063	0.426610448
NFIC	0.125815366	0.427234932

CCDC9	0.125802296	0.427283336
GPR160	0.125757804	0.427448133
KTN1	0.125715561	0.427604633
IRF2BP2	0.125539414	0.428257558
COQ4	0.12549667	0.428416083
SYT17	0.125483575	0.428464654
DOK1	0.125458153	0.428558959
HELLS	0.125437965	0.428633854
TNFAIP8L1	0.12542649	0.428676428
RAB3GAP1	0.12538022	0.428848125
ALKBH2	0.125234058	0.429390746
CORO2A	0.125233262	0.429393702
CLDN9	0.125212989	0.429468995
ZNF738	0.125212757	0.429469858
PELO	0.125138133	0.429747072
SLAMF8	0.125127585	0.429786266
ZFYVE9	0.12512053	0.42981248
TIGD5	0.125118874	0.429818632
NISCH	0.125111964	0.429844312
TIMM29	0.124901557	0.430626574
CALU	0.124890127	0.43066909
SPP1	0.124880501	0.4307049
CAMK1D	0.124785121	0.431059806
NUF2	0.1247847	0.431061375
TMEM175	0.124684243	0.431435349
FARSA	0.124530572	0.432007779
PTRH2	0.124397731	0.432502953
TMEM14C	0.124391134	0.432527551
RAMP1	0.124352361	0.432672145
ALDOC	0.124349741	0.432681917
ASB1	0.124114162	0.433561035
PABPC4	0.124070658	0.433723489
RNF130	0.124067479	0.433735364
SLC25A30	0.124046376	0.433814179

ASL	0.12402199	0.433905269
ZC3H8	0.123872441	0.434464107
OSBPL10	0.123871602	0.434467244
RBM27	0.123801584	0.434729029
IGKV1D-8	0.123785649	0.434788618
TIMM17A	0.123764477	0.434867798
SPOP	0.123757628	0.434893415
RPN2	0.123703954	0.435094196
OGFRL1	0.123663824	0.435244342
TIMM9	0.123657078	0.435269586
PCYT1A	0.123597408	0.435492903
TESMIN	0.123543264	0.435695593
USP3	0.123528094	0.435752395
EXTL2	0.12351437	0.435803784
ZDHHC24	0.123500978	0.435853932
NTPCR	0.123479023	0.435936153
ASB14	0.123383086	0.43629554
PRDM1	0.123307581	0.436578498
EXOC6B	0.12330232	0.436598219
CREBBP	0.123166665	0.437106863
GNAS	0.123057886	0.437514975
COA7	0.12302277	0.437646764
SERPINB1	0.123009201	0.437697698
CYLD	0.123008046	0.437702031
FCGR1BP	0.122970721	0.437842147
OSBPL7	0.12294699	0.437931244
SURF6	0.122913075	0.438058597
NEU3	0.122900345	0.438106402
MRTFA	0.122876038	0.438197692
SUPT3H	0.122862968	0.438246783
NSUN5	0.12283773	0.438341587
TRIM7	0.122741388	0.43870359
SIGLEC8	0.122702628	0.438849279
MCM4	0.122580117	0.439309932

MTF2	0.122508268	0.439580217
ENSG00000273759	0.12246357	0.439748412
SMYD5	0.122450046	0.439799309
PRAF2	0.122348176	0.440182789
ZNF597	0.122145265	0.440947182
ACTR2	0.121821735	0.442167477
LTF	0.121810397	0.442210277
THAP7	0.121784371	0.442308528
APOBEC2	0.12177941	0.442327255
RAB2A	0.121776541	0.442338087
AKAP7	0.121663805	0.442763834
TOR3A	0.121617999	0.442936882
AIRIM	0.121600315	0.443003701
HDHD3	0.121541992	0.443224111
SIGLEC15	0.121538331	0.443237948
TXNRD2	0.121496195	0.443397225
HIC2	0.121477832	0.443466648
TOP1MT	0.121465364	0.443513791
TNFAIP6	0.121427395	0.443657364
VCPKMT	0.121407765	0.443731602
PIP5K1B	0.121369844	0.443875032
NME6	0.121354804	0.443931926
MARK4	0.121174081	0.44461588
BOD1L1	0.121165848	0.444647054
HIP1	0.121045108	0.445104341
TOMM40	0.121003639	0.445261457
NOD1	0.120991625	0.445306981
SLC2A8	0.120948476	0.445470504
WAC-AS1	0.120942513	0.445493108
PROCA1	0.120898398	0.445660329
ACOT2	0.12078499	0.446090378
RBM15B	0.120773282	0.446134787
PALB2	0.12072629	0.446313057
CENPP	0.120598301	0.446798805

CENPO	0.120545804	0.446998123
PLOD1	0.120484848	0.44722962
NBR2	0.120405995	0.447529186
MAST2	0.12036237	0.447694964
ETV7	0.120308322	0.447900403
RBM3	0.120233992	0.44818301
ACAP3	0.120155317	0.448482249
C10orf95-AS1	0.120146017	0.448517626
TPM3	0.120142522	0.448530922
MRPL2	0.120045497	0.448900125
DNPEP	0.120036241	0.448935354
ENSG00000262823	0.120023697	0.4489831
PILRA	0.119970305	0.44918636
ALOX15	0.119917912	0.449385866
TRMT6	0.119883328	0.449517583
LIN7A	0.119871647	0.449562079
GSTCD	0.119505907	0.450956408
VMAC	0.119453853	0.451155048
ATF1	0.119431034	0.451242141
SLPI	0.11936771	0.451483878
HMGCL	0.1193509	0.451548062
BID	0.119294448	0.45176364
RAB28	0.119254241	0.451917215
NEIL2	0.118983715	0.452951266
GNAQ	0.118890189	0.453309055
EPG5	0.11872059	0.453958258
TMEM203	0.118622756	0.454332985
RAB35	0.118576215	0.454511304
AGPAT2	0.118552108	0.454603685
ENSG00000267279	0.11854958	0.454613375
PPCDC	0.118436045	0.455048595
GLG1	0.118428912	0.455075945
SYT2	0.118420046	0.455109942
ZNF562	0.118416704	0.455122757

TPX2	0.118391562	0.455219175
MFGE8	0.118390509	0.455223211
CETN2	0.118363885	0.455325324
SMIM13	0.118329656	0.455456622
TCN1	0.118256956	0.455735561
ENSG00000277978	0.118243792	0.455786078
EXOSC5	0.118081457	0.456409302
NEK3	0.118006238	0.456698235
ERICH1	0.117990463	0.456758843
MICU1	0.117895631	0.457123277
LITATS1	0.117861709	0.457253675
RARS1	0.117738304	0.457728227
ANXA1	0.117661234	0.45802473
PIM2	0.117460448	0.45879768
ASB8	0.117455321	0.458817426
ENSG00000254531	0.117416275	0.458967827
PYROXD1	0.117381836	0.459100499
CDPF1	0.117350325	0.459221913
RFX1	0.117319945	0.459338983
MORN1	0.117246736	0.459621165
ENSG00000272211	0.117196007	0.459816755
GTF3C1	0.11713365	0.460057235
PRORP	0.117082752	0.460253577
VPS37C	0.116848893	0.461156269
KIR2DS4	0.11679318	0.461371461
SIGLEC5	0.116766298	0.461475312
TMEM65	0.116728212	0.46162247
USB1	0.116594963	0.462137513
TNNC2	0.116591029	0.462152726
AP3S1	0.116527638	0.462397862
SBF2-AS1	0.116435037	0.46275608
CRTAP	0.116398456	0.46289763
SLC5A3	0.116377964	0.462976936
ALS2	0.116330784	0.463159551
