# Supplementary material for: Neutrophil-enriched gene signature correlates with teplizumab therapy resistance in different stages of type 1 diabetes
Source: J Clin Invest. 2025 Sep 30;135(23):e176403. doi: 10.1172/JCI176403 (PMC12646666; doi:10.1172/JCI176403)
Supplement: Supplemental table 8 [file jci-135-176403-s296.pdf]

Supplemental Table 8: Predictive response score

**Symbol**	**Coefficient**
ZNF667	0.005216149
TRBV10-2	0.002906216
C8orf58	0.002405641
DUSP5	0.001530691
NR1H3	0.001396412
ABHD14A	0.001362636
FUT11	0.000812976
TRAPPC4	0.000563408
NCR3	0.000311761
DCXR	0.000258884
HLA-DRB5	8.27E-05
IL2RG	7.43E-05
ACTG1	1.91E-05
GAS7	-0.000201274
RBBP6	-0.000434968
TRAPPC6B	-0.00106405
PIGM	-0.001091661
NHLRC3	-0.00112606
ZNF141	-0.001483622
CARF	-0.001507113
KANSL1L	-0.001831619
SCRN3	-0.001957425
KIF28P	-0.00592505
P2RX5-TAX1BP3	-0.006638634
ZNF790-AS1	-0.008498776
CCDC13	-0.009325166
